# Organocatalytic Asymmetric Synthesis of Si-Stereogenic Silyl Ethers

**DOI:** 10.1021/jacs.2c04261

**Published:** 2022-06-01

**Authors:** Hui Zhou, Jung Tae Han, Nils Nöthling, Monika M. Lindner, Judith Jenniches, Clemens Kühn, Nobuya Tsuji, Li Zhang, Benjamin List

**Affiliations:** † Max-Planck-Institut für Kohlenforschung, Kaiser-Wilhelm-Platz 1, 45470 Mülheim an der Ruhr, Germany; ‡ Innovation Center, Merck KGaA, Frankfurter Straße 250, 64293 Darmstadt, Germany; § Institute for Chemical Reaction Design and Discovery (WPI-ICReDD), Hokkaido University, Sapporo 001-0021, Japan

## Abstract

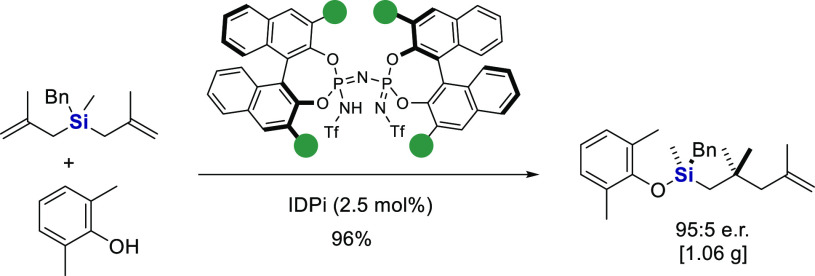

Functionalized enantiopure organosilanes are important building blocks with applications in various fields of chemistry; nevertheless, asymmetric synthetic methods for their preparation are rare. Here we report the first organocatalytic enantioselective synthesis of tertiary silyl ethers possessing “central chirality” on silicon. The reaction proceeds via a desymmetrizing carbon–carbon bond forming silicon–hydrogen exchange reaction of symmetrical bis(methallyl)silanes with phenols using newly developed imidodiphosphorimidate (IDPi) catalysts. A variety of enantiopure silyl ethers was obtained in high yields with good chemo- and enantioselectivities and could be readily derivatized to several useful chiral silicon compounds, leveraging the olefin functionality and the leaving group nature of the phenoxy substituent.

Chiral molecules bearing a carbon stereogenic center are ubiquitous in nature and have been the focus of fundamental and applied studies during the past decades.^1^ In contrast, the asymmetric synthesis of their heavier congeners bearing a stereogenic *silicon* atom has been far less investigated. As enantiopure organosilanes are currently gaining substantial importance in material science, in polymer synthesis, as chiral ligands, and in scent and medicinal chemistry, an expansion of the synthetic toolbox to enable their preparation is highly desirable.^2−8^ A literature survey reveals that Si-stereogenic silanes have been created either via desymmetrization and diastereoselective synthesis from achiral silicon compounds or via the kinetic resolution of racemic silanes by means of transition metal and enzyme catalysis.^9−13^ Indeed, the catalytic construction of Si-stereogenic centers appears to be a topic of high current relevance in asymmetric catalysis.^14−24^ However, to the best of our knowledge, organocatalytic asymmetric approaches to enantiopure silanes constitute an unmet challenge.
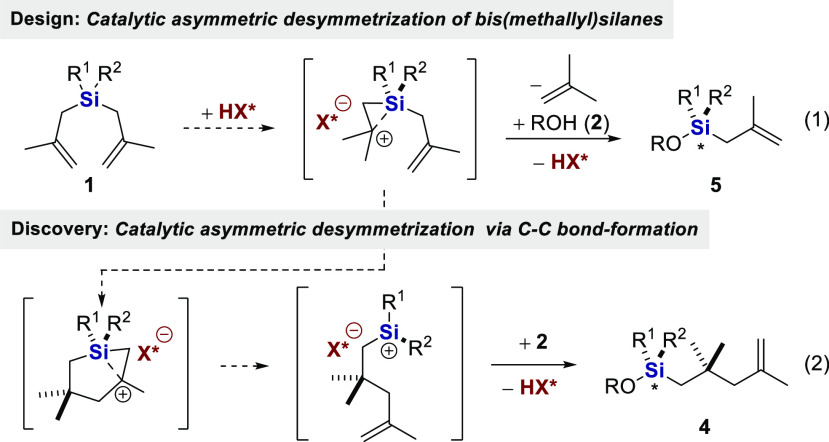
 Inspired by previous work on asymmetric catalysis of the silicon–hydrogen exchange reaction,^25,26^ we considered a catalytic desymmetrizing silyl ether formation from bis(methallyl)silanes **1** with alcohols **2**, in which olefin protonation occurs from an acid catalyst HX* (**3**) to form a β-silyl-stabilized cationic intermediate, followed by nucleophilic attack of the alcohol to generate the chiral silyl ether **5** (eq 1).

We report here that instead of the intermolecular addition of the alcohol, an *intramolecular* cation−π cyclization with the second olefin occurs, ultimately leading to a silylium ion equivalent, which reacts with the alcohol to form an enantioenriched silyl ether **4**, featuring a new C–C σ-bond (eq 2). We have discovered a highly enantioselective, desymmetrizing, carbon–carbon bond forming reaction of symmetrical bis(methallyl)silanes with phenols. The perfectly atom-economic reaction is catalyzed by newly developed imidodiphosphorimidates (IDPi) and delivers several structurally distinct silyl ethers. We also describe the utilization of the obtained products in the synthesis of various Si-stereogenic silanes and suggest a mechanism of this unusual transformation.

As the starting point, bis(methallyl)silane **1a** and 2,6-dimethyl-phenol **2a** were reacted with IDPi catalyst **3a**, featuring a 1-naphthyl substituent at the 3,3′-position of the BINOL backbone and a trifluoromethyl sulfonyl group in the inner core. Product **4a** was obtained as the main product with only small amounts of ether **5a** (Table 1). Notably, we obtained the new chiral organosilane **4a** in 73% yield and a promising enantioselectivity of 64:36 e.r. (Table 1, entry 1). A subsequent investigation of the reaction conditions revealed toluene as the optimal solvent, affording the desired product in 86% yield and 86:14 e.r. (entries 2–4). Lowering the temperature to −20 °C suppressed the formation of side product **5a** and led to an increased yield without significantly affecting the enantioselectivity (entry 5). Spirocyclic-fluorenyl-substituted IDPi catalysts, which have been preferred motifs in our previous Si-ACDC studies, were subsequently investigated (entries 6–9).^27−36^ We eventually identified IDPi **3e** as the optimal catalyst, which enabled quantitative formation of product **4a** with 97:3 e.r. (entry 9).

**Table 1:**
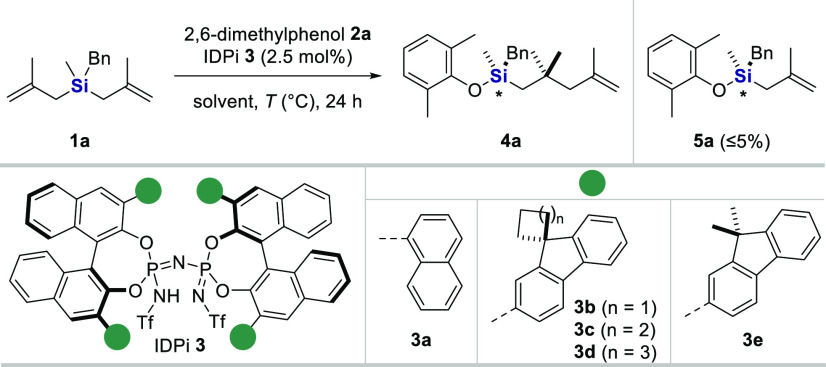
Reaction Development^a^

entry	catalyst	solvent	*T* (°C)	yield (%)^b^	e.r.^c^
1	**3a**	diethyl ether	25	73	64:36
2	**3a**	dichloromethane	25	76	86:14
3	**3a**	cyclohexane	25	90	79:21
4	**3a**	toluene	25	86	86:14
5	**3a**	toluene	–20	94	86.5:13.5
6	**3b**	toluene	–20	>95	85:15
7	**3c**	toluene	–20	>95	89.5:10.5
8	**3d**	toluene	–20	>95	95:5
9	**3e**	toluene	–20	>95	97:3

a Performed with 2,6-dimethylphenol **2a** (0.025 mmol), **1a** (1.5 equiv), and IDPi catalysts **3a**–**3e** (2.5 mol %) in solvent (0.25 mL, 0.1 M).

b Yields determined by ^1^H NMR spectroscopy using 1,3,5-trimethoxybenzene as internal standard.

c Enantiomeric ratios (e.r.) determined by HPLC.

Having identified these optimized conditions, we investigated other substrates for this new catalytic asymmetric approach to Si-stereogenic organosilanes. As illustrated in Table 2, benzyl-substituted chiral silane **4a** was isolated in 92% yield with 97:3 e.r. on a 0.2 mmol scale. Related silanes **4b**–**4d** were prepared in similarly good yields and enantioselectivities (87–96% yield and 96:4 to 98:2 e.r., respectively) from the corresponding benzyl silanes with electron-donating or -withdrawing groups at the *para-*position of the phenyl ring. In addition, substituents with diverse electronic properties in *meta*- and *ortho*-positions of the phenyl group were well tolerated, affording chiral organosilanes **4e**–**4i** with comparable outcome. 3,4-Dimethyl-substituted product **4j** was obtained in 93% yield and an e.r. of 93.5:6.5. The use of substrate **1k** bearing a bulkier 2-naphthylmethyl group on the silicon atom delivered **4k** with good yield and 95:5 e.r. While the reactions of benzyl-substituted silanes (**1a**–**1k**) occurred with good chemoselectivity, aryl-substituted substrates (**1l**–**1n**) initially led to the formation of a side product and required further optimization of the reaction conditions (see the Supporting Information, Table S3). Ultimately, this gave products **4l**–**4n** in 78–84% yields with 95:5 e.r. The silicon-stereogenic silane **4o**, bearing a thiophenyl moiety, could also be obtained in satisfactory yield and with high enantioselectivity. Product **4p** was generated from silane **1p**, bearing three methallyl groups, with 76:24 e.r., and no further substitution was detected. Submitting silane **1q** to the reaction conditions revealed the key role of the methyl substituent on the silicon atom; indeed, its replacement with an ethyl group delivered product **4q** in quantitative yield but with a significantly decreased enantioselectivity (70.5:29.5 e.r.).

**Table 2:**
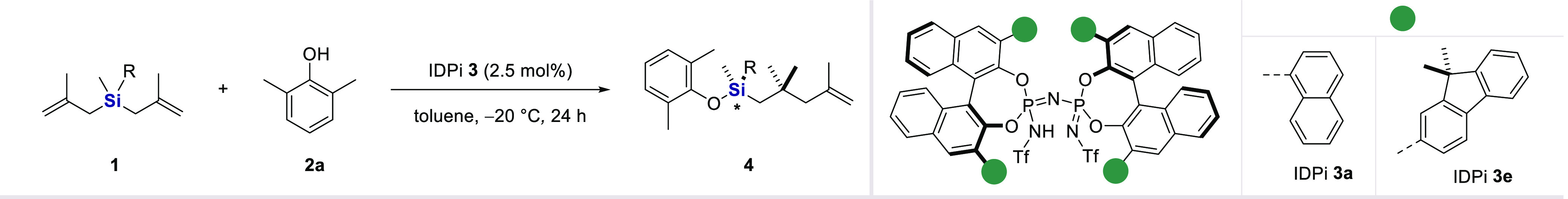

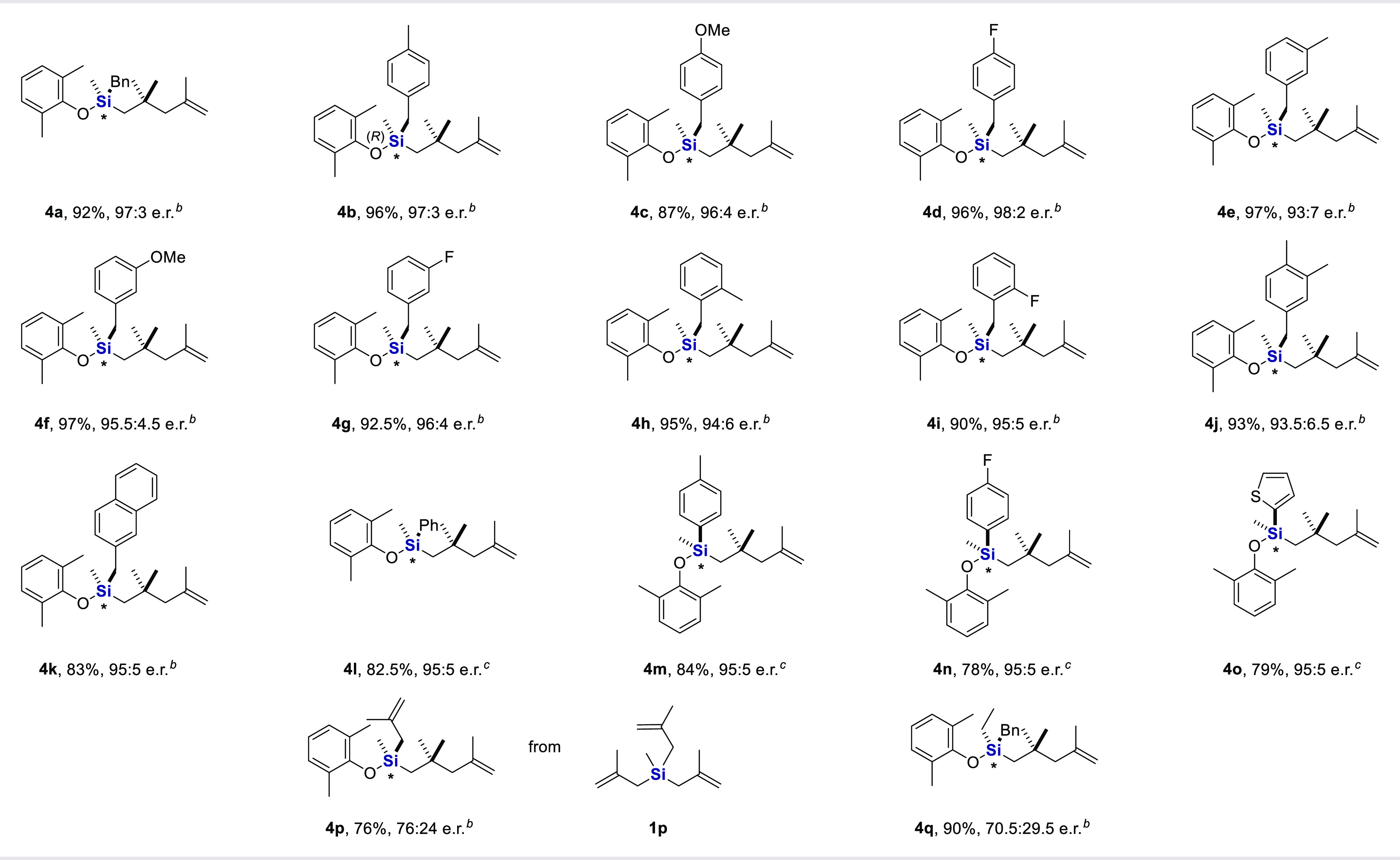
Substrate Scope^a^

a Performed with 2,6-dimethylphenol **2a** (0.2 mmol), **1** (1.5 equiv), and IDPi catalyst **3a** or **3e** (2.5 mol %) in toluene (2.0 mL, 0.1 M) at −20 °C for 24 h. Isolated yields with enantiomeric ratio (e.r.) determined by HPLC analysis.

b With IDPi **3e**.

c With IDPi **3a**.

Despite extensive attempts, we were unable to obtain suitable single crystals of our new organosilicon compounds for the determination of their absolute configuration by single-crystal X-ray diffraction. Therefore, the “crystalline sponge method” was applied to elucidate the absolute structure by X-ray analysis (CS-XRD).^37−40^

In this method, single crystals of a flexible MOF, the crystalline sponge (CS), are used as a scaffold to arrange the analyte molecules into the pores and apply X-ray crystallographic analysis to determine the structure. With this method, we could unequivocally determine the absolute structure of the analyte molecules. The space group symmetry of the original CS changed from centrosymmetric (*C*2/*c*) to non-centrosymmetric (*C*2), to accommodate enantiopure molecules.

As depicted in Figure 1a, the absolute configuration of product **4b** was assigned to be *R* by CS-XRD. Several independent additional experiments using the same and the opposite enantiomer confirmed the correctness of this assignment (see the Supporting Information, Figures S13–S30 and Tables S4–S6).

**Figure 1:**
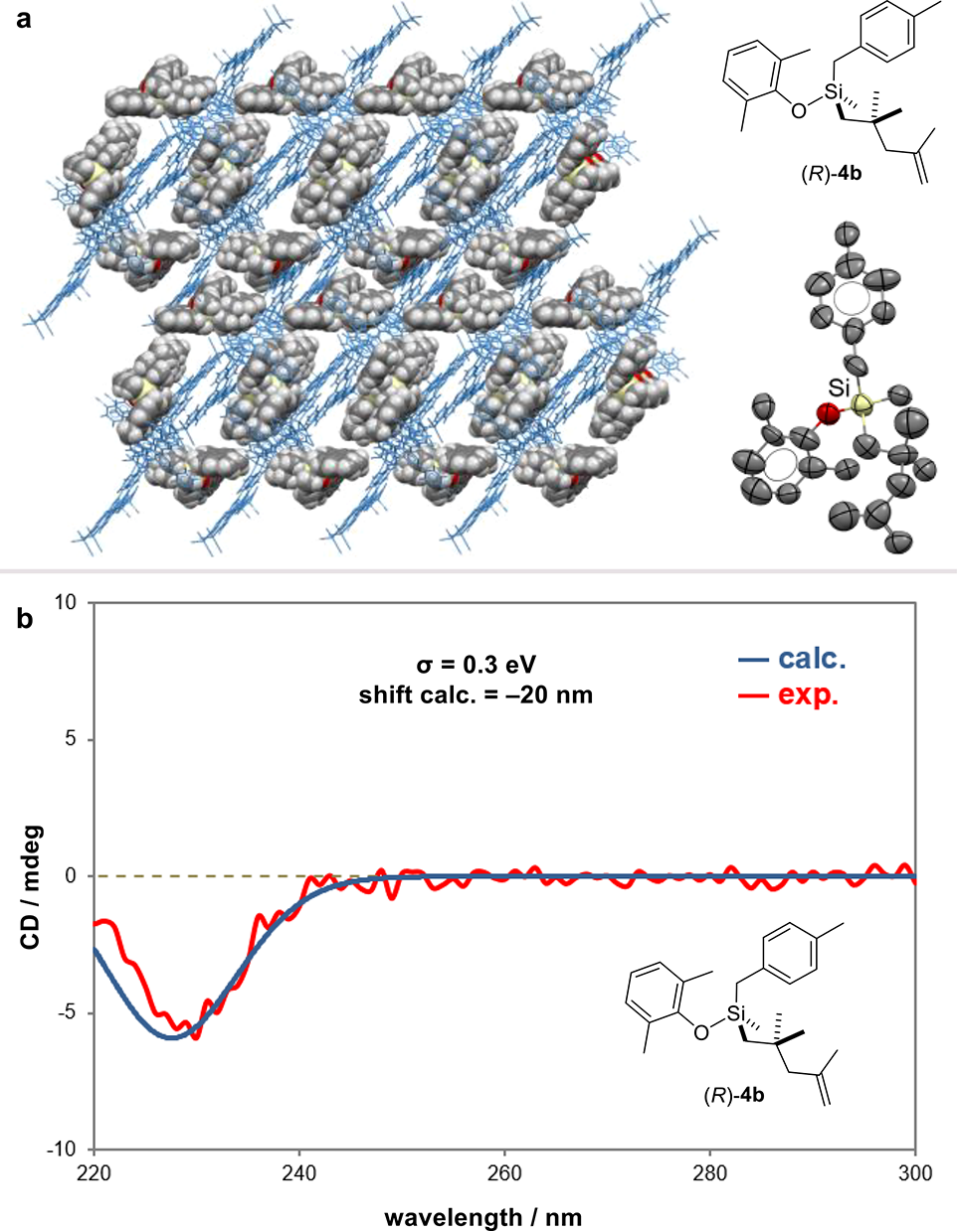
Absolute configuration determination of (*R***)-4b**. (a) The crystalline sponge method was employed. (b) Calculated (blue curve) and experimental (red curve) CD spectra of **4b**.

The absolute configuration of products **4b** and **4d** was furthermore confirmed to be *R* by computational and experimental CD spectroscopy (Figure 1b and Figure S33 in the Supporting Information).^41^

To illustrate the practical utility of our methodology and the synthetic value of the newly obtained enantiopure organosilicon products, a preparative-scale reaction was performed and readily delivered 1.06 g of product **4a** in 96% yield and 95:5 e.r. (Figure 2). Hydroboration/oxidation of olefin **4a** gave compound **6**, with moderate diastereoselectivity and without erosion of enantiopurity.^42^ Upon cyclopropanation or epoxidation, silane products **7** and **8** could be obtained in 40% and 55% yield, respectively, with identical e.r.^43,44^ Hydrogenation of **4a** led to product **9** in 88% yield. Moreover, a sequential strategy was employed for the conversion of the Si–O bond into a Si–C bond. Accordingly, in situ treatment of product **9** with diisobutylaluminum hydride gave hydrosilane **10**.^45^ A subsequent Pt-catalyzed hydrosilylation with 1-octene provided quaternary silane **11**.^18^ While the absolute stereochemistry of this product remains to be confirmed, it has previously been shown that the reduction of alkoxysilanes with diisobutylaluminum hydride and also the hydrosilylation of 1-octene, respectively, proceed via retention.^46,47^ The direct construction of a Si–C bond by treatment of silyl ether **4a** with *n*-BuLi occurred with substantial loss of enantioselectivity and gave quaternary silane **12**, mostly with retention of configuration at the silicon stereocenter.^48^ A Pt-catalyzed intramolecular hydrosilylation of unsaturated hydrosilane **13**, which is readily obtained via reduction of **4a**, gave product **14** with moderate d.r. and retention of configuration.^46^ The Si–H bond in silane **13** could be converted into a Si–OH group via dehydrogenative coupling with water in the presence of Pd/C.^49^ This transformation has been shown to proceed with inversion of configuration, furnishing chiral silanol **15**.^50^ Finally, boronate **16** was prepared in 90% yield as a 1:1 mixture of diastereomers via hydroboration.^51^

**Figure 2:**
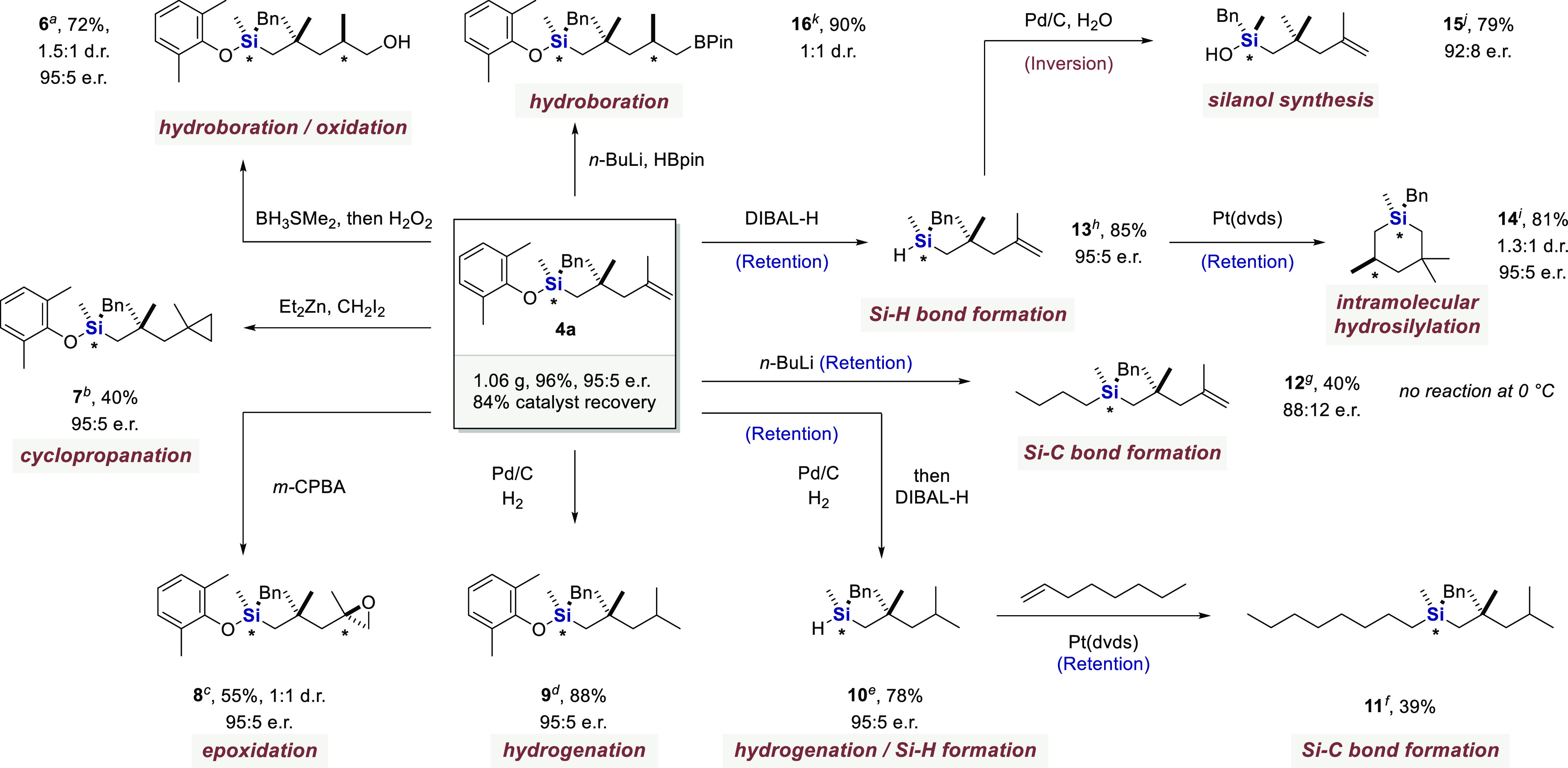
Derivatizations. Isolated yields with e.r. determined by HPLC and d.r. measured by ^1^H NMR spectroscopy or HPLC. (a) BH_3_SMe_2_ (1.0 equiv), 0 °C, THF, 1 h, then H_2_O_2_ (7.8 equiv), rt, EtOH, 3 h. (b) Et_2_Zn (2.0 equiv), CH_2_I_2_ (4.0 equiv), 0 °C–rt, DCE, 18 h. (c) *m*-CPBA (2.0 equiv), 0 °C–rt, DCM, 7 h. (d) Pd/C (0.1 equiv), H_2_ (balloon), rt, MeOH, 12 h. (e) Pd/C (0.1 equiv), H_2_ (balloon), rt, MeOH, 12 h, then DIBAL-H (2.0 equiv), 0 °C–rt, hexanes, 18 h. (f) Pt(dvds) (0.05 equiv), 1-octene (2.0 equiv), 50 °C, hexanes, 48 h. (g) *n*-BuLi (3.0 equiv), 0–35 °C, Et_2_O, 48 h. (h) DIBAL-H (2.0 equiv), 0 °C–rt, hexanes, 18 h. (i) Pt(dvds) (0.05 equiv), 50 °C, hexanes, 48 h. (j) Pd/C (0.1 equiv), H_2_O (3.0 equiv), 0 °C, ethyl acetate, 24 h. (k) *n*-BuLi (0.1 equiv), HBPin (3.0 equiv), 130 °C, toluene, 18 h.

To elucidate the reaction mechanism, we conducted several additional experiments. Using phenyl-substituted silane **1l** as starting material, we could isolate side product **5b**, which enabled us to rule out its possible role as an intermediate, undergoing an intermolecular hydroallylation. As expected, mixing compounds **5b** and **1l** with 1.1 equiv of phenol **2b** and IDPi **3a** as catalyst in toluene at room temperature for 24 h did not lead to any detectable amounts of product **4l** (Figure 3, eq 1). Further, when the reaction between substrates **1a** and **2a** was conducted for only 12 h at a reduced catalyst loading (1 mol %), cyclic Si-stereogenic silane **17a** was isolated in 20% yield. Reaction progress kinetic studies suggested silane **17a** to be a (“parasitic”) intermediate of the reaction (see the Supporting Information, Figure S5). Interestingly, the e.r. of this six-membered silane product was determined to be 53:47 (eq 2). That cyclization is indeed not the enantio-determining step was then confirmed when we reacted racemic silane **17a** with phenol **2a**, which cleanly furnished product **4a** in 75% yield and 96:4 e.r. (Figure 3, eq 3). As such, the enantio-determining step is supposed to be the Si–O bond formation, and steric effects on enantiocontrol in the corresponding transition states were elucidated by DFT studies (see the Supporting Information, Figures S31 and S32 and Table S7). Furthermore, in light of the formation of product **4p** from the corresponding tris-methallyl silane **1p**, with significant enantioselectivity, olefin protonation as the enantio-determining step is also unlikely.

**Figure 3:**
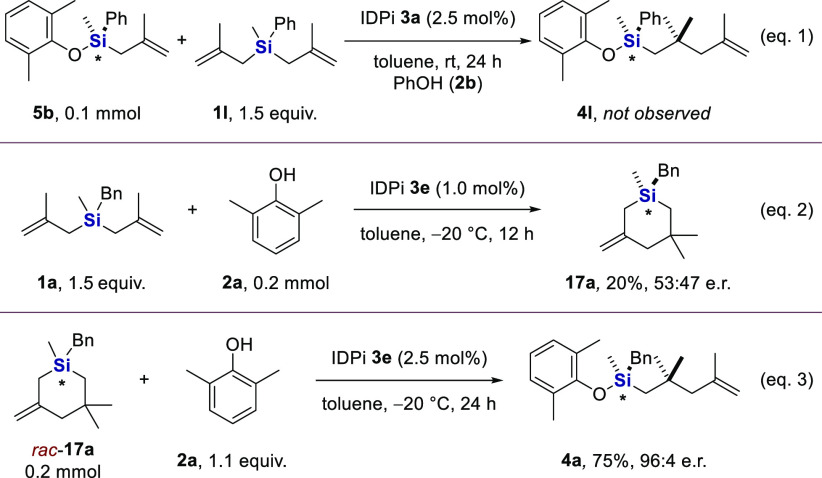
Control experiments. Reaction of eq 1 was performed with **5b** (0.1 mmol), silane **1l** (1.5 equiv), phenol **2b** (1.1 equiv), and IDPi **3a** (2.5 mol %) in toluene (1.0 mL, 0.1 M) at rt for 24 h. Reaction of eq 2 was performed with **2a** (0.2 mmol), silane **1a** (1.5 equiv), and IDPi **3e** (1.0 mol %) in toluene (2.0 mL, 0.1 M) at −20 °C for 12 h and terminated by the addition of Et_3_N. Reaction of eq 3 was performed with *rac*-**17a** (0.2 mmol), **2a** (1.1 equiv), and IDPi **3e** (2.5 mol %) in toluene (2.0 mL, 0.1 M) at −20 °C for 24 h.

Based on these results, a plausible reaction mechanism can be proposed (Figure 4). Accordingly, the catalytic cycle commences with the protonation of symmetrical silane **1** by IDPi **3** to provide ion pair **I**, the carbocation of which is stabilized by silicon hyperconjugation. Subsequent cation−π cyclization takes place to afford the ion pair **II**. Deprotonation of its cyclic cation gives compound **17**, an isolable intermediate that can reversibly be protonated to regenerate ion pair **II**. Alternatively, Si–C bond cleavage would lead to silylium-based ion pair **III**. Finally, reaction of this intermediate with phenol **2a** furnishes product **4** and regenerates catalyst **3**.

**Figure 4:**
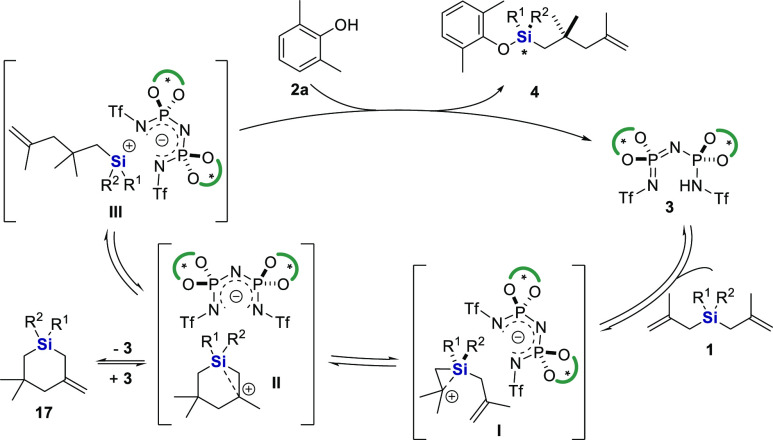
Proposed mechanism.

In conclusion, we have realized an organocatalytic asymmetric synthesis of Si-stereogenic silyl ethers that proceeds via a C–C bond forming desymmetrization and is enabled by our IDPi catalysts. Various non-natural, enantioenriched silane products could be generated and were utilized in the synthesis of valuable silane derivatives with potential application in material and medicinal chemistry. Our approach features scalability, broad substrate scope, operational simplicity, and mechanistic novelty. Particularly, we observed and characterized an unprecedented six-membered cyclic chiral silane, the protonated form of which may act as an intermediate in the catalytic cycle. Our newly developed strategy provides a practical and efficient access to Si-stereogenic compounds, which may find utilization in the synthesis of silicon-containing materials, pharmaceuticals, and chiral ligands for transition-metal-catalyzed reactions.

## Supporting Information Available

Experimental details and analytical data for all new compounds (PDF)

## Accession Codes

CCDC 2115183 and 2166402–2166408 contain the supplementary crystallographic data for this paper. These data can be obtained free of charge via www.ccdc.cam.ac.uk/data_request/cif, or by emailing data_request@ccdc.cam.ac.uk, or by contacting The Cambridge Crystallographic Data Centre, 12 Union Road, Cambridge CB2 1EZ, UK; fax: +44 1223 336033.
