# Supplementary material for: Organocatalytic Asymmetric Synthesis of Si-Stereogenic Silyl Ethers
Source: J Am Chem Soc. 2022 Jun 1;144(23):10156–61. doi: 10.1021/jacs.2c04261 (PMC9490845; doi:10.1021/jacs.2c04261)
Supplement: Supplementary file 1 — ja2c04261_si_001.pdf [file ja2c04261_si_001.pdf]

Organocatalytic Asymmetric Synthesis of *Si*-Stereogenic Silyl Ethers

Hui Zhou,† Jung Tae Han,† Nils Nöthling,† Monika M. Lindner,† Judith Jenniches,‡ Clemens Kühn,‡ Nobuya Tsuji,§ Li Zhang,† and Benjamin List*†§

† Max-Planck-Institut für Kohlenforschung, Kaiser-Wilhelm-Platz 1, D-45470 Mülheim an der Ruhr, Germany

‡ Innovation Center, Merck KGaA, Frankfurter Straße 250, 64293 Darmstadt, Germany

§ Institute for Chemical Reaction Design and Discovery (WPI-ICReDD), Hokkaido University, Sapporo 001-0021, Japan

*Email: list@kofo.mpg.de

Content

1. General considerations S2

2. Initial investigations of a suitable nucleophile S4

3. Development of suitable reaction conditions and catalyst identification S5

4. The preparation of different Grignard reagents S7

5. The preparation and characterization of the silicon starting materials S8

6. Substrate scope for the organocatalytic asymmetric synthesis of tertiary silyl ethers S12

7. Optimization of the reaction with aromatic silane **1l** with **2a** S20

8. Gram-scale reaction and derivatizations of **4a** S21

9. Mechanistic studies S28

10. Inclusion and co-crystallization experiments for single crystal X-ray structure determination S30

11. DFT calculations and CD experiments S61

12. References S135

13. Copies of NMR spectra S138

14. Copies of HPLC traces S199

1. General considerations

Chemicals

Unless otherwise indicated, starting materials were obtained from Sigma-Aldrich, ABCR-GmbH, TCI, or Acros Co. Ltd. Moreover, commercially available reagents were used without additional purification. Symmetrical silanes **1a**–**1q** were synthesized according to literature procedure with modifications.[1] 2,6-Dimethylphenol **2a** was obtained from commercial suppliers and used after stepwise chromatographic purification and recrystallization. The chiral imidodiphosphorimidate acids (IDPis) **3a**[2] and **3e**[3] were known compounds and synthesized according to literature procedures.

Solvents

Solvents (diethylether, cyclohexane, dichloromethane, and toluene) were dried by distillation from an appropriate drying agent in the technical department of the Max-Planck-Institut für Kohlenforschung and received in Schlenk flasks under argon. In addition, CH_3CN was purchased from commercial suppliers and dried over molecular sieves.

Inert Gas

Dry argon was purchased from Air Liquide with >99.5% purity.

Thin Layer Chromatography

Thin-layer chromatography (TLC) was performed using silica gel pre-coated plastic sheets (Polygram SIL G/UV_{254}, 0.2 mm, with fluorescent indicator; Macherey-Nagel) which was visualized with a UV lamp (254 nm) and/or phosphomolybdic acid (PMA). PMA stain: PMA (20 g) in EtOH (200 mL).

Column Chromatography

Column chromatography (CC) was carried out using Merck silica gel (60 Å, 230−400 mesh, particle size 0.040−0.063 mm) using technical grade solvents. Elution was accelerated using compressed argon. All reported yields, unless otherwise specified, refer to spectroscopically and chromatographically pure compounds.

Nomenclature

Nomenclature follows the suggestions proposed by the computer program ChemBioDraw (12.0.3.1216) of CBD/cambridgesoft.

Nuclear Magnetic Resonance Spectroscopy

^{1}H, ^{13}C, ^{19}F, ^{31}P Nuclear magnetic resonance (NMR) spectra for compound characterization were recorded on Bruker AVIII-500 MHz, NMR spectrometer in a suitable deuterated solvent. The solvent employed and the respective measuring frequency are indicated for each experiment. Chemical shifts are reported with tetramethylsilane (TMS) serving as a universal reference of all nuclides. The resonance multiplicity is described as s (singlet), d (doublet), t (triplet), q (quadruplet), m (multiplet), and b (broad). All spectra were recorded at 298 K, processed with MestReNova 14.2.3 suits of program, and coupling constants are reported as observed. The residual deuterated solvent signal relative to tetramethylsilane was used as the internal reference in ^{1}H and ^{13}C NMR spectra (e.g. CD_2Cl_2 = 5.32 ppm in ^{1}H NMR and 53.8 ppm in ^{13}C NMR).[4] Signals are reported as follows: chemical shift δ in ppm (multiplicity, coupling constant *J* in Hz, number of protons). All X-nuclei spectra were acquired proton decoupled unless otherwise noted.

Kinetic NMR measurements were performed at a Bruker AVIII- 300MHz WB NMR spectrometer. The temperature of low temperature experiments was calibrated against a 4% MeOH in MeOD-d_4 sample.[5] Further details are mentioned in the corresponding section.

Mass Spectrometry

Electrospray ionization (ESI) mass spectrometry was conducted on a Bruker ESQ 3000 spectrometer. High resolution mass spectra were determined on a Bruker APEX III FTMS (7 T magnet). The ionization method and mode of detection employed is indicated for the respective experiment and all masses are reported in atomic units per elementary charge (*m/z*) with an intensity normalized to the most intense peak.

Specific Rotations

Specific rotations ($[\boldsymbol{\alpha}]_{\mathbf{D}}^{\mathbf{T}}$) were measured with a Rudolph RA Autopol IV Automatic Polarimeter at the indicated temperature with a sodium lamp (sodium D line, $\boldsymbol{\lambda}$ = 589 nm). Measurements were performed in an acid resistant 1 mL cell (50 mm length) with concentrations (g/(100 mL)) reported in the corresponding solvent.

High Performance Liquid Chromatography

High performance liquid chromatography (HPLC) was performed on a Shimadzu LC-20AD liquid chromatograph SIL-20AC auto sampler, CMB-20A using Daicel columns with a chiral stationary phase. All solvents used were HPLC-grade solvents purchased from Sigma-Aldrich. The column employed and the respective solvent mixture are indicated for each experiment.

Melting Points

Melting points (m.p.) were measured on a Büchi 540 Melting Point apparatus in open glass capillaries and are uncorrected.

Concentration Determination of Grignard Reagents

Titration was performed on a Metrohm volumetric titrator (DE).

CD(Circular Dichroism) Spectra

CD spectra were recorded at 20 °C in hexane (HPLC grade) using a JASCO J-810 spectropolarimeter and precision cells (quartz suprasil, 2mm, Hellma).

Abbreviations

e.r. = enantiomeric ratio, TLC = thin layer chromatography, CH_3CN = acetonitrile, TBS = $SiMe_2{}^tBu$, Tf = SO_2CF_3, MOM = methoxymethyl ether.

2. Initial investigations of a suitable nucleophile

Based on our previously developed silicon-hydrogen exchange reaction, which affords enol silane triggered by the release of propene, we sought to change the silyl source from allyl(*tert*-butyl) dimethylsilane to a symmetrical diallyl silane in order to obtain the enol silane bearing a silicon-stereogenic center. As we designed in **Figure S1, eq. 1**, the Si-H exchange reaction of cyclohexanone (0.1 mmol) and allyl(*tert*-butyl) dimethylsilane (1.5 equiv.) using *rac*-IDPi was carried out in toluene (0.1 M) at room temperature for 24 h, providing the desired enol silane product in 85% NMR yield with 1,3,5-trimethoxybenzene as internal standard. While the reaction between 3,3-dimethylbutan-2-one and benzyl(methyl)bis(2-methylallyl)silane under the same reaction conditions didn't work, no any desired chiral silane product bearing a Si-stereogenic center was detected (**eq. 2**).

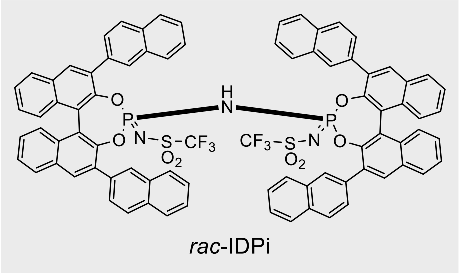

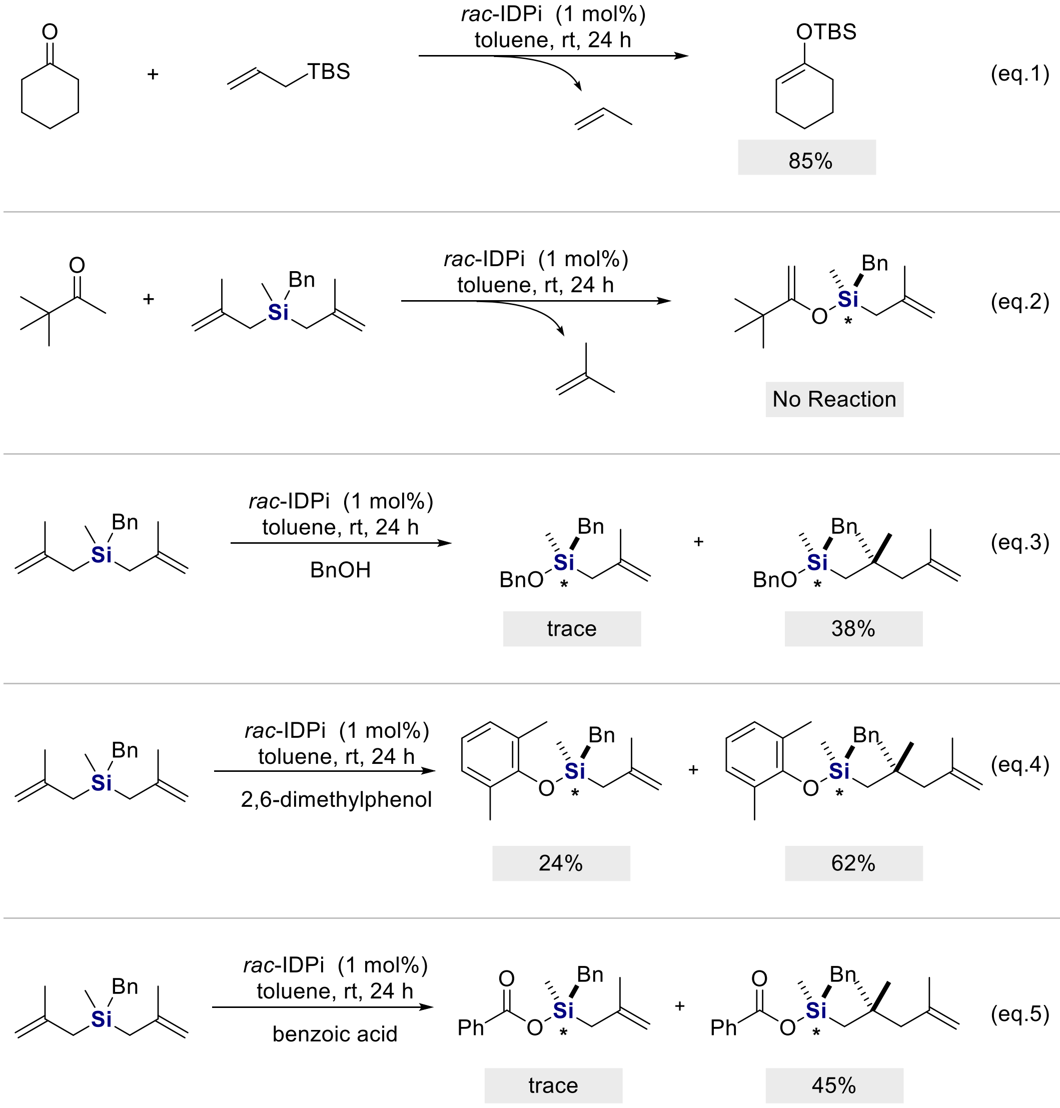

Figure S1. Initial investigations of suitable nucleophiles. For eq. 1, the reaction was performed with cyclohexanone (0.1 mmol), allyl(*tert*-butyl) dimethylsilane (1.5 equiv.), and *rac*-IDPi catalyst (1 mol%) in toluene (1.0 mL, 0.1 M) at room temperature for 24 h. For eq. 2, the reaction was performed with 3,3-dimethylbutan-2-one (0.1 mmol), benzyl(methyl)bis(2-methylallyl)silane (1.5 equiv.), and *rac*-IDPi catalyst (1 mol%) in toluene (1.0 mL, 0.1 M) at room temperature for 24 h. For eq. 3, the reaction was performed with phenylmethanol (0.1 mmol), benzyl(methyl)bis(2-methylallyl)silane (1.5 equiv.), and *rac*-IDPi catalyst (1 mol%) in toluene (1.0 mL, 0.1 M) at room temperature for 24 h. For eq. 4, the reaction was performed with 2,6-dimethylphenol (0.1 mmol), benzyl(methyl)bis(2-methylallyl)silane (1.5 equiv.), and *rac*-IDPi catalyst (1 mol%) in toluene (1.0 mL, 0.1 M) at room temperature for 24 h. For eq. 5, the reaction was performed with benzoic acid (0.1 mmol), benzyl(methyl)bis(2-methylallyl)silane (1.5 equiv.), and *rac*-IDPi catalyst (1 mol%) in toluene (1.0 mL, 0.1 M) at room temperature for 24 h. All yields were determined by NMR analysis using 1,3,5-trimethoxybenzene as internal standard.

Continuous endeavors were focused on the screening of smaller but stronger nucleophiles, such as phenylmethanol, 2,6-dimethylphenol, and benzoic acid. Interestingly, when using phenylmethanol, only trace amount of Si-H exchange products enabled by the release of isobutene was detected, while the unexpected chiral organosilanes were obtained with perfect atom economy in 38% yield, implying an entirely different mechanism from the previously established system (**eq. 3**). Similarly, reactions with 2,6-dimethylphenol and benzoic acid were conducted, which also afforded the similar major products. It is noteworthy that reaction with 2,6-dimethylphenol provided the new chiral silicon product in 62% yield (**eq. 4** and **eq. 5**).

3. Development of suitable reaction conditions and catalyst identification

General procedure for the optimization of the organocatalytic asymmetric synthesis of chiral Si-stereogenic silane.

On the basis of the results from **Figure S1**, **eq. 4** that the new chiral silane could be obtained with promising reactivity and chemoselectivity using 2,6-dimethylphenol as nucleophile, we performed the reaction optimization as shown in **Table S1**.

Table S1. Screening of the reaction conditions:[a]

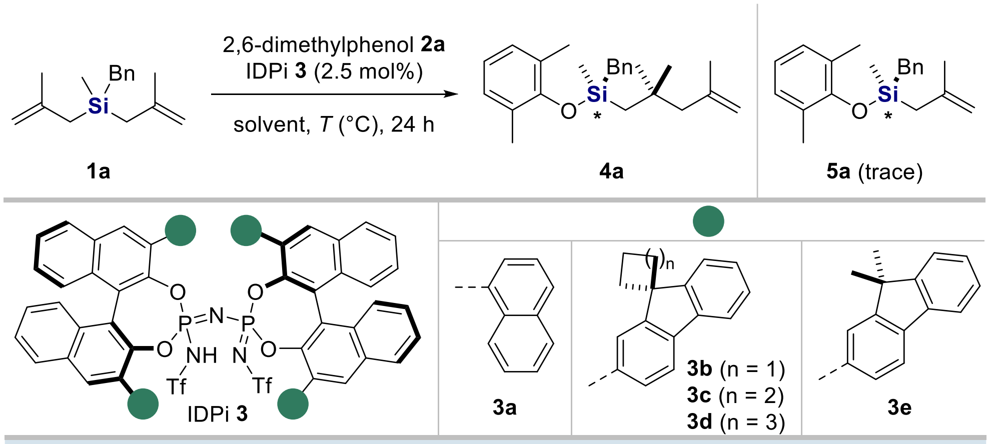

entry	catalyst	solvent	T (ºC)	yield (%)[b]	e.r.[c]
1	**3a**	diethylether	25	73	64:36
2	**3a**	dichloromethane	25	76	86:14
3	**3a**	cyclohexane	25	90	79:21
4	**3a**	tetrahydrofuran	25	<5	--
5	**3a**	toluene	25	86	86:14
6	**3a**	toluene	–20	94	86.5:13.5
7	**3b**	toluene	–20	>95	85:15
8	**3c**	toluene	–20	>95	89.5:10.5
9	**3d**	toluene	–20	>95	95:5
10	**3e**	toluene	–20	>95	97:3

[a]Reactions were performed with 2,6-dimethylphenol **2a** (0.025 mmol), symmetrical silane **1a** (1.5 equiv.), and IDPi catalysts **3a**–**3e** (2.5 mol%) in solvent (0.25 mL, 0.1 M). [b]Yields were determined by ^{1}H NMR using 1,3,5-trimethoxybenzene as internal standard. [c]Enantiomeric ratios (e.r.) were determined by HPLC analysis.

Prochiral diallyl silane starting material **1a** (0.0375 mmol, 1.5 equiv.) was placed in a 1.5 mL GC vial, which was equipped with a teflon-coated magnetic stirring bar. IDPi **3a–3e** (2.5 mol%) and solvent (0.1 M, 0.25 mL) were added, and the resultant solution was stirred for 5 min at 25 °C. 2,6-Dimethylphenol **2a** (0.025 mmol, 1.0 equiv.) was slowly added and the reaction mixture was stirred for an additional 24 h at the indicated temperature. After the ketone was fully consumed, as monitored by TLC, the reaction mixture was treated with triethylamine. Volatiles were removed in vacuo and the yield of **4a** was determined by NMR analysis with 1,3,5-trimethoxybenzene as internal standard. The enantiomeric ratio was determined by HPLC analysis after purification by prep. TLC.

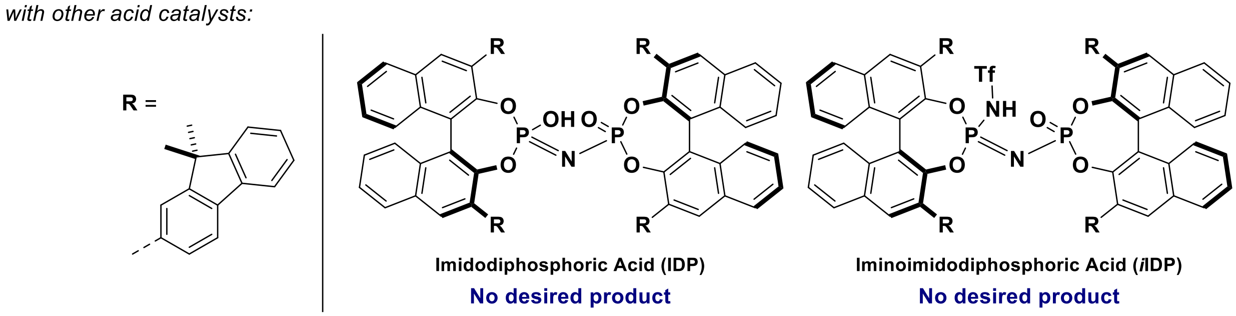

Figure S2. Reactivity examinations with IDP and *i*IDP under standard reaction conditions.

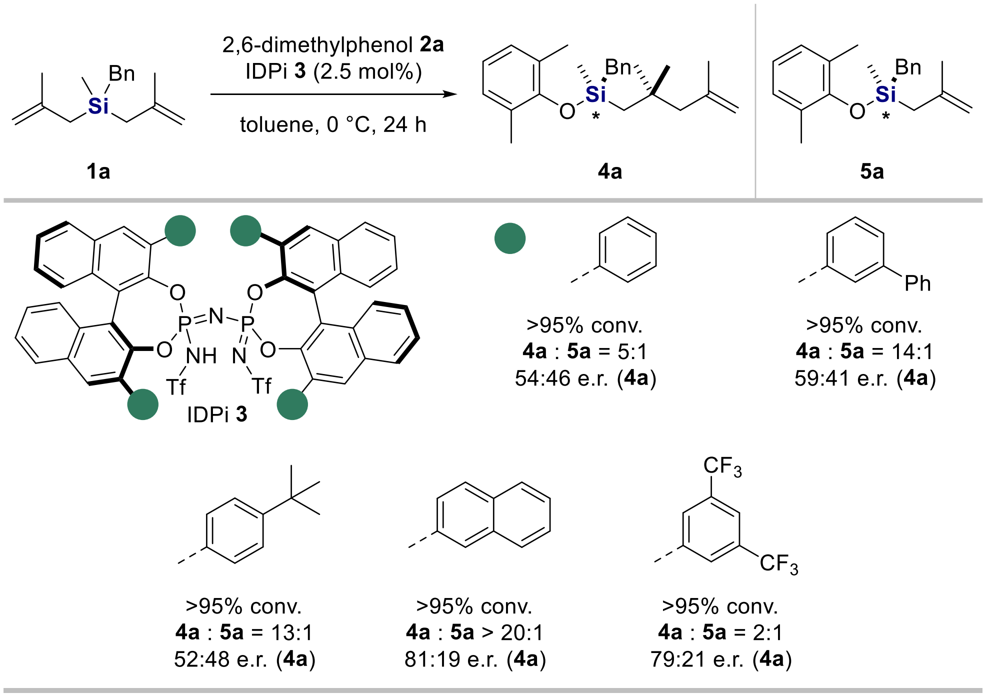

Figure S3. Reaction catalyzed by different IDPi catalysts bearing common substituents.

Table S2. **Screening of the different phenol nucleophiles:**

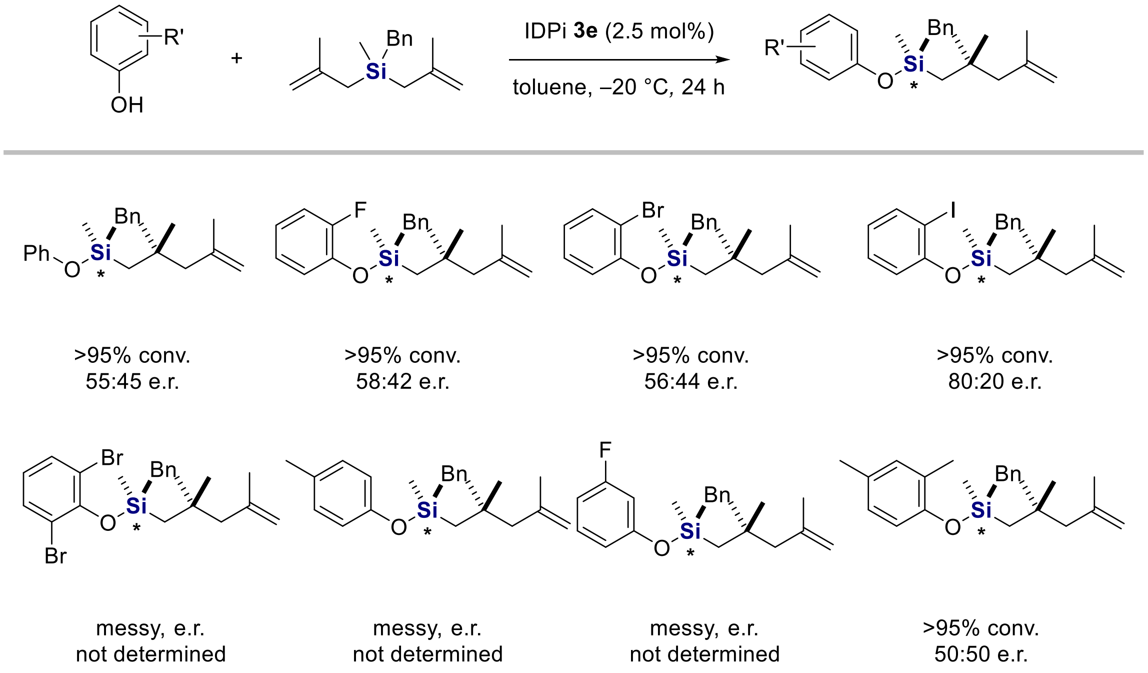

Meanwhile, we also studied several different phenol nucleophiles under the same reaction conditions, as shown above in **Table S2**.

4. The preparation of different Grignard reagents

Method A: preparation of aryl Grignard reagent

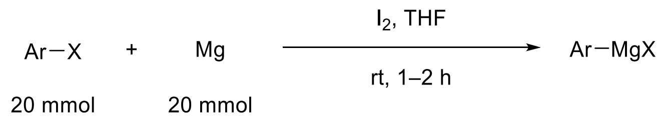

A 50 mL three-necked round-bottom flask, which was flame-dried and argon-flushed in prior to use, was equipped with a magnetic stir bar, a reflux condenser and a 50 mL pressure-equalizing dropping funnel and charged with Mg turnings (20 mmol, 1.0 equiv) and two grain of iodine at rt. The dropping funnel was charged with a solution of the corresponding aryl bromide or aryl chloride precursors (20 mmol) in anhydrous THF (20 mL). The aryl halide solution was added in small portion to the flask and the resultant solution was stirred until the Grignard reaction commenced, which could be indicated by the changed color from dark purple to colorless. When the initial vigorous reaction had subsided, the remained halide solution was added at a rate such that the mixture refluxed gently. After the addition was completed within 0.5 h, the generated dark brown solution was stirred at rt for 0.5 h, and the concentration was determined by titration.

Method B: preparation of thiophenyl magnesium chloride (0.15 M in THF)

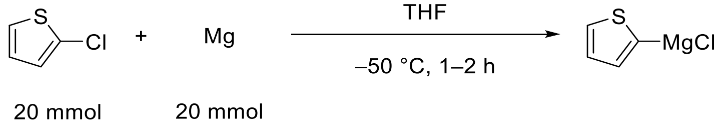

A 50 mL two-necked round-bottom flask, which was flame-dried and argon-flushed in prior to use, was equipped with a magnetic stir bar and a 50 mL pressure-equalizing dropping funnel and charged with Mg turnings (30 mmol, 2.0 equiv) at rt. The dropping funnel

was charged with a solution of the 2-chlorothiophene (30 mmol) in anhydrous THF (20 mL). The solution was added dropwise to the flask and the resultant solution was stirred at –50 ºC for 1 h, and the concentration of the Grignard reagent was determined by titration.

Method C: preparation of (2-methylallyl)magnesium chloride (0.75 M in THF)

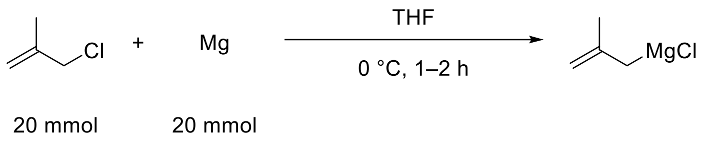

A 50 mL three-necked round-bottom flask, which was flame-dried and argon-flushed in prior to use, was equipped with a magnetic stir bar and a 50 mL pressure-equalizing dropping funnel and charged with Mg turnings (20 mmol, 1.0 equiv) at rt. The dropping funnel was charged with a solution of the 3-chloro-2-methylprop-1-ene (20 mmol) in anhydrous THF (20 mL). The solution was added in small portion to the flask at 0 ºC and the resultant solution was stirred until the Grignard reaction commenced, which could be indicated by the continuous formation of bubbles on the surface of magnesium turnings. When the initial vigorous reaction had subsided, the remained halide solution was added dropwise. After the addition was completed within 0.5 h, the resultant dark brown solution was gradually warmed up to rt and stirred at the indicated temperature for 0.5 h, and the concentration was determined by titration.

5. The preparation and characterization of the silicon starting materials[1]

Method A: To a solution of methyl trichlorosilane (10.0 mmol) in THF (20 mL), different Grignard reagents R^1-MgX (10.0 mmol) were added at 0 ºC under argon atmosphere. After the resulting mixture was stirred at 0 ºC for 2 h, a solution of (2-methylallyl)magnesium chloride (0.75 M in THF, 2.5 equivalent, 25 mmol) was added to the mixture at 0 °C. The reaction mixture was gradually warmed up to rt and stirred for 24 h. After the completion that was indicated by TLC, the reaction was quenched with sat. aq. NH_4Cl and extracted with hexanes. The combined organic phases were dried over Na_2SO_4, filtrated and dried under reduced pressure. Purification of the crude by silica gel chromatography with hexanes afforded the desired substituted bis(2-methylallyl)silane **1**.

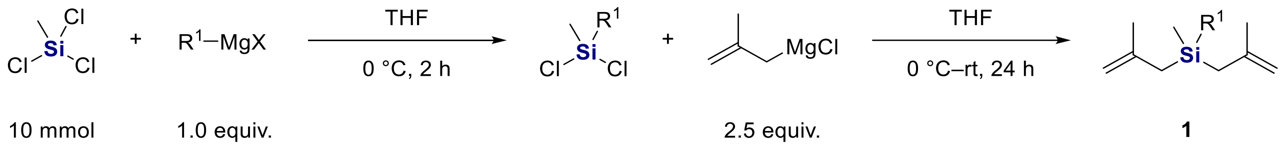

Method B: To a solution of dichloro(methyl)(phenyl)silane (10.0 mmol) in THF (20 mL), the solution of (2-methylallyl)magnesium chloride (0.75 M in THF, 2.5 equivalent, 25 mmol) prepared beforehand was added dropwise at 0 °C. After the addition, the resulting mixture was gradually warmed up to room temperature and stirred for 24 h. Utill the full conversion was confirmed by TLC, the reaction was quenched with sat. aq. NH_4Cl and extracted with hexanes. The combined organic phases were dried over Na_2SO_4, filtrated and dried under reduced pressure. Purification of the crude product by silica gel chromatography with hexanes afforded the desired product **1l**.

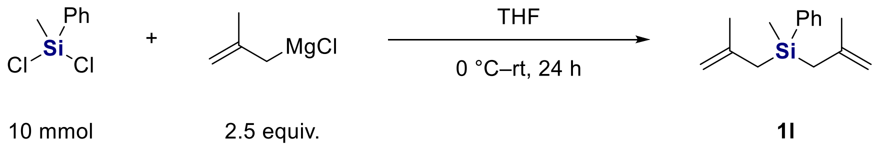

The starting material **1l** was prepared according to the reported procedure with minor modifications. This compound is known, and its spectra are in accordance with the reported structure.

Method C: To a solution of silicon tetrachloride (10.0 mmol) in THF (20 mL), ethyl magnesium chloride (10.0 mmol) was added at 0 ºC under argon, and the resulting mixture was stirred at 0 ºC for 2 h. Benzyl magnesium chloride was then added to the prepared ethyl trichlorosilane via syringe. After 2 h at 0 ºC, a solution of (2-methylallyl)magnesium chloride (0.75 M in THF, 2.5 equivalent, 25 mmol) prepared beforehand was added to the mixture at 0 °C. The reaction mixture was gradually warmed up to room temperature and stirred for 24 h. After the completion that was indicated by TLC, the reaction was quenched with sat. aq. NH_4Cl and extracted with hexanes. The combined organic phases were dried over Na_2SO_4, filtrated and dried under reduced pressure. Purification of the crude by silica gel chromatography with hexanes afforded the desired product **1q**.

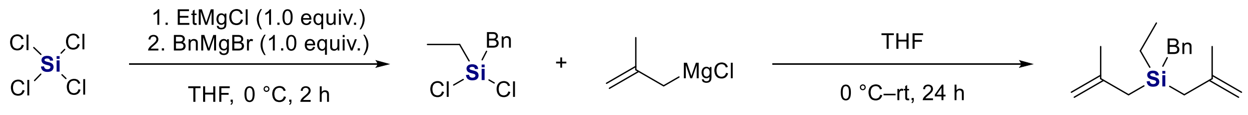

10 mmol 2.5 equiv. **1q**

benzyl(methyl)bis(2-methylallyl)silane (1a)

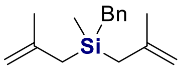

^{1}H NMR (501 MHz, CD_2Cl_2) δ 7.21 (dd, *J* = 8.4, 7.0 Hz, 2H), 7.09–7.05 (m, 1H), 7.05–7.00 (m, 2H), 4.63 (dt, *J* = 2.8, 1.4 Hz, 2H), 4.51 (dq, *J* = 2.0, 1.0 Hz, 2H), 2.18 (s, 2H), 1.70 (t, *J* = 1.1 Hz, 6H), 1.64–1.54 (m, 4H), 0.02 (s, 3H).

^{13}C NMR (126 MHz, CD_2Cl_2) δ 143.6, 140.2, 128.8, 128.6, 124.4, 109.4, 25.6, 25.5, 24.4, –4.3.

R_f = 0.32 (hexanes).

APPI-HRMS (*m/z*): calculated for $C_{16}H_{25}Si_1$ ($[M+H]^+$): 245.1720, found: 245.1718.

methylbis(2-methylallyl)(4-methylbenzyl)silane (1b)

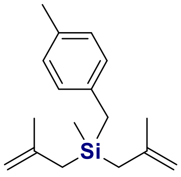

^{1}H NMR (501 MHz, CD_2Cl_2) δ 7.02 (d, *J* = 7.8 Hz, 2H), 6.94–6.89 (m, 2H), 4.63 (dd, *J* = 2.5, 1.4 Hz, 2H), 4.51 (dq, *J* = 1.9, 0.9 Hz, 2H), 2.28 (s, 3H), 2.13 (s, 2H), 1.70 (t, *J* = 1.1 Hz, 6H), 1.64–1.54 (m, 4H), 0.01 (s, 3H).

^{13}C NMR (126 MHz, CD_2Cl_2) δ 143.7, 136.8, 133.8, 129.2, 128.7, 109.4, 25.6, 25.5, 23.8, 21.0, –4.3.

R_f = 0.29 (hexanes).

EI-HRMS (*m/z*): calculated for $C_{17}H_{26}Si_1$ $[M^{+\bullet}]$: 258.1798, found: 258.1796.

(4-methoxybenzyl)(methyl)bis(2-methylallyl)silane (1c)

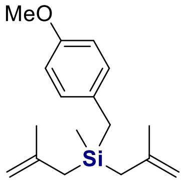

^{1}H NMR (501 MHz, CD_2Cl_2) δ 6.97–6.92 (m, 2H), 6.78–6.74 (m, 2H), 4.63 (dt, *J* = 2.5, 1.4 Hz, 2H), 4.50 (dq, *J* = 2.1, 1.0 Hz, 2H), 3.77–3.73 (m, 3H), 2.10 (s, 2H), 1.70 (t, *J* = 1.1 Hz, 6H), 1.63–1.54 (m, 4H), 0.01 (s, 3H).

^{13}C NMR (126 MHz, CD_2Cl_2) δ 157.2, 143.7, 131.8, 129.6, 114.0, 109.3, 55.5, 25.6, 25.6, 23.1, –4.3.

R_f = 0.35 (Ethyl acetate/hexanes = 1:20).

EI-HRMS (*m/z*): calculated for $C_{17}H_{26}O_1Si_1$ $[M^{+\bullet}]$: 274.1747, found: 274.1744.

(4-fluorobenzyl)(methyl)bis(2-methylallyl)silane (1d)

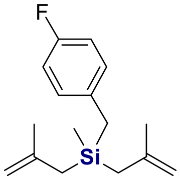

^{1}H NMR (501 MHz, CD_2Cl_2) δ 7.02–6.97 (m, 2H), 6.94–6.88 (m, 2H), 4.64 (dq, *J* = 2.8, 1.4 Hz, 2H), 4.51 (dq, *J* = 2.1, 1.1 Hz, 2H), 2.16 (s, 2H), 1.70 (t, *J* = 1.1 Hz, 6H), 1.64–1.54 (m, 4H), 0.02 (s, 3H).

^{13}C NMR (126 MHz, CD_2Cl_2) δ 160.8 (d, *J* = 240.6 Hz), 143.5, 135.9 (d, *J* = 3.2 Hz), 130.0 (d, *J* = 7.6 Hz), 115.2 (d, *J* = 21.1 Hz), 109.5, 25.6, 25.5, 23.5, –4.4.

^{19}F NMR (471 MHz, CD_2Cl_2) δ –120.9.

R_f = 0.35 (hexanes).

CI-HRMS (*m/z*): calculated for $C_{16}H_{24}F_1Si_1$ ($[M+H]^+$): 263.1626, found: 263.1626.

methylbis(2-methylallyl)(3-methylbenzyl)silane (1e)

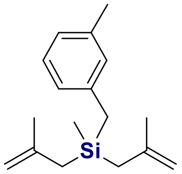

^{1}H NMR (501 MHz, CD_2Cl_2) δ 7.09 (t, *J* = 7.5 Hz, 1H), 6.88 (d, *J* = 7.6 Hz, 1H), 6.86 (d, *J* = 1.9 Hz, 1H), 6.84–6.80 (m, 1H), 4.63 (dq, *J* = 2.8, 1.4 Hz, 2H), 4.51 (dq, *J* = 2.1, 1.0 Hz, 2H), 2.28 (s, 3H), 2.14 (s, 2H), 1.70 (t, *J* = 1.1 Hz, 6H), 1.63–1.54 (m, 4H), 0.02 (s, 3H).

^{13}C NMR (126 MHz, CD_2Cl_2) δ 143.7, 140.0, 138.1, 129.6, 128.4, 125.8, 125.2, 109.4, 109.3, 25.6, 25.6, 24.3, 21.5, –4.3.

R_f = 0.31 (hexanes).

CI-HRMS (*m/z*): calculated for $C_{17}H_{27}Si_1$ ($[M+H]^+$): 259.1876, found: 259.1872.

(3-methoxybenzyl)(methyl)bis(2-methylallyl)silane (1f)

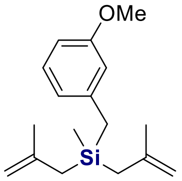

^{1}H NMR (501 MHz, CD_2Cl_2) δ 7.12 (t, *J* = 7.9 Hz, 1H), 6.65–6.55 (m, 3H), 4.64 (dq, *J* = 2.8, 1.4 Hz, 2H), 4.52 (dt, *J* = 2.3, 1.0 Hz, 2H), 3.76 (s, 3H), 2.16 (s, 2H), 1.71 (t, *J* = 1.1 Hz, 6H), 1.64–1.56 (m, 4H), 0.03 (s, 3H).

^{13}C NMR (126 MHz, CD_2Cl_2) δ 160.1, 143.6, 141.8, 129.4, 121.3, 114.4, 109.9, 109.5, 55.4, 25.6, 25.6, 24.6, –4.2.

R_f = 0.31 (Ethyl acetate/hexanes = 1:20).

EI-HRMS (*m/z*): calculated for $C_{17}H_{26}O_1Si_1$ $[M^{+\bullet}]$: 274.1747, found: 274.1744.

(3-fluorobenzyl)(methyl)bis(2-methylallyl)silane (1g)

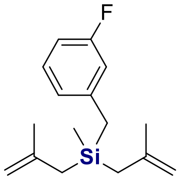

^{1}H NMR (501 MHz, CD_2Cl_2) δ 7.18 (td, *J* = 7.9, 6.2 Hz, 1H), 6.82 (dt, *J* = 7.6, 1.3 Hz, 1H), 6.80–6.71 (m, 2H), 4.65 (dd, *J* = 2.4, 1.3 Hz, 2H), 4.52 (dd, *J* = 2.2, 1.2 Hz, 2H), 2.20 (s, 2H), 1.70 (t, *J* = 1.1 Hz, 6H), 1.64–1.55 (m, 4H), 0.03 (s, 3H).

^{13}C NMR (126 MHz, CD_2Cl_2) δ 163.3 (d, *J* = 243.8 Hz), 143.4, 143.2 (d, *J* = 7.8 Hz), 130.0 (d, *J* = 8.6 Hz), 124.6 (d, *J* = 2.6 Hz), 115.4 (d, *J* = 21.0 Hz), 111.2 (d, *J* = 21.0 Hz), 109.6, 25.6, 25.5, 24.5, –4.3.

^{19}F NMR (471 MHz, CD_2Cl_2) δ –120.9.

R_f = 0.49 (hexanes).

EI-HRMS (*m/z*): calculated for $C_{16}H_{24}F_1Si_1$ ($[M+H]^+$): 263.1626, found: 263.1622.

methylbis(2-methylallyl)(2-methylbenzyl)silane (1h)

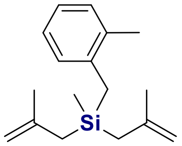

^{1}H NMR (501 MHz, CD_2Cl_2) δ 7.12–7.07 (m, 1H), 7.07–7.02 (m, 1H), 6.98 (ddd, *J* = 14.3, 7.3, 1.6 Hz, 2H), 4.64 (dt, *J* = 2.8, 1.4 Hz, 2H), 4.51 (dq, *J* = 1.9, 0.9 Hz, 2H), 2.22 (s, 3H), 2.19 (s, 2H), 1.70 (t, *J* = 1.1 Hz, 6H), 1.62 (qd, *J* = 13.5, 1.1 Hz, 4H), –0.00 (s, 3H).

^{13}C NMR (126 MHz, CD_2Cl_2) δ 143.6, 138.8, 135.2, 130.5, 129.2, 126.0, 124.6, 109.5, 26.1, 25.6, 20.9, 20.6, –4.0.

R_f = 0.35 (hexanes).

CI-HRMS (*m/z*): calculated for $C_{17}H_{27}Si_1$ ($[M+H]^+$): 259.1876, found: 259.1873.

(2-fluorobenzyl)(methyl)bis(2-methylallyl)silane (1i)

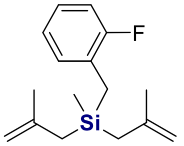

^{1}H NMR (501 MHz, CD_2Cl_2) δ 7.10–7.04 (m, 2H), 7.04–6.95 (m, 2H), 4.64 (dq, *J* = 2.8, 1.4 Hz, 2H), 4.51 (dq, *J* = 2.0, 0.9 Hz, 2H), 2.18 (d, *J* = 2.0 Hz, 2H), 1.70 (t, *J* = 1.1 Hz, 6H), 1.67–1.56 (m, 4H), 0.03 (d, *J* = 1.0 Hz, 3H).

^{13}C NMR (126 MHz, CD_2Cl_2) δ 160.8 (d, *J* = 242.3 Hz), 143.5, 131.1 (d, *J* = 4.9 Hz), 127.4 (d, *J* = 16.7 Hz), 126.2 (d, *J* = 7.6 Hz), 124.2 (d, *J* = 3.5 Hz), 115.4 (d, *J* = 21.7 Hz), 109.5, 25.7, 25.5, 17.1, 17.1, –4.4.

^{19}F NMR (471 MHz, CD_2Cl_2) δ –117.2.

R_f = 0.52 (hexanes).

CI-HRMS (*m/z*): calculated for $C_{16}H_{24}F_1Si_1$ ($[M+H]^+$): 263.1626, found: 263.1625.

(3,4-dimethylbenzyl)(methyl)bis(2-methylallyl)silane (1j)

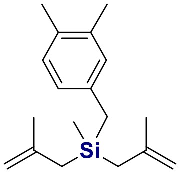

^{1}H NMR (501 MHz, CD_2Cl_2) δ 6.96 (d, *J* = 7.6 Hz, 1H), 6.81 (d, *J* = 2.0 Hz, 1H), 6.75 (dd, *J* = 7.6, 2.0 Hz, 1H), 4.63 (dt, *J* = 2.8, 1.4 Hz, 2H), 4.51 (dt, *J* = 2.0, 1.0 Hz, 2H), 2.20 (d, *J* = 4.1 Hz, 6H), 2.09 (s, 2H), 1.70 (t, *J* = 1.1 Hz, 6H), 1.63–1.54 (m, 4H), 0.01 (s, 3H).

^{13}C NMR (126 MHz, CD_2Cl_2) δ 143.8, 137.2, 136.6, 132.4, 130.2, 129.7, 126.2, 109.3, 25.6, 23.7, 19.9, 19.3, –4.3.

R_f = 0.44 (hexanes).

CI-HRMS (*m/z*): calculated for $C_{18}H_{27}Si_1$ ($[M–H]^–$): 271.1876, found: 271.1873.

methylbis(2-methylallyl)(naphthalen-2-ylmethyl)silane (1k)

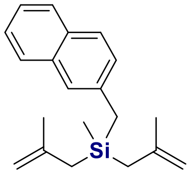

^{1}H NMR (501 MHz, CD_2Cl_2) δ 7.79 (dd, *J* = 8.2, 1.3 Hz, 1H), 7.76–7.71 (m, 2H), 7.52–7.48 (m, 1H), 7.44 (ddd, *J* = 8.1, 6.8, 1.4 Hz, 1H), 7.39 (ddd, *J* = 8.0, 6.8, 1.3 Hz, 1H), 7.23 (dd, *J* = 8.4, 1.8 Hz, 1H), 4.68 (dq, *J* = 2.8, 1.4 Hz, 2H), 4.56 (dq, *J* = 2.0, 0.9 Hz, 2H), 2.38 (s, 2H), 1.78–1.71 (m, 6H), 1.70–1.61 (m, 4H), 0.08 (s, 3H).

^{13}C NMR (126 MHz, CD_2Cl_2) δ 143.2, 137.6, 133.9, 131.1, 128.1, 127.5, 127.5, 126.9, 125.8, 125.7, 124.5, 109.2, 25.3, 25.2, 24.4, –4.6.

R_f = 0.32 (hexanes).

EI-HRMS (*m/z*): calculated for $C_{20}H_{26}Si_1$ $[M^{+\bullet}]$: 294.1798, found: 294.1794.

methylbis(2-methylallyl)(phenyl)silane (1l)

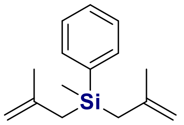

^{1}H NMR (501 MHz, CD_2Cl_2) δ 7.57–7.51 (m, 2H), 7.39–7.31 (m, 3H), 4.58 (dt, *J* = 2.8, 1.4 Hz, 2H), 4.48 (dq, *J* = 2.1, 1.0 Hz, 2H), 1.84 (d, *J* = 1.1 Hz, 4H), 1.57 (t, *J* = 1.1 Hz, 6H), 0.37 (s, 3H).

^{13}C NMR (126 MHz, CD_2Cl_2) δ 143.5, 138.1, 134.4, 129.4, 128.0, 109.4, 26.5, 25.5, –5.0.

R_f = 0.32 (hexanes).

EI-HRMS (*m/z*): calculated for $C_{15}H_{22}Si_1$ $[M^{+\bullet}]$: 230.1485, found: 230.1482.

methylbis(2-methylallyl)(p-tolyl)silane (1m)

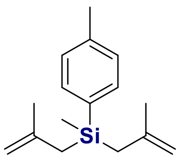

^{1}H NMR (501 MHz, CD_2Cl_2) δ 7.45–7.40 (m, 2H), 7.17 (d, *J* = 7.5 Hz, 2H), 4.58 (dd, *J* = 2.5, 1.4 Hz, 2H), 4.47 (dt, *J* = 2.0, 1.0 Hz, 2H), 2.34 (s, 3H), 1.82 (d, *J* = 1.0 Hz, 4H), 1.57 (t, *J* = 1.1 Hz, 6H), 0.35 (s, 3H).

^{13}C NMR (126 MHz, CD_2Cl_2) δ 143.7, 139.4, 134.4, 134.3, 128.8, 109.3, 26.6, 25.5, 21.6, –4.9.

R_f = 0.66 (hexanes).

EI-HRMS (*m/z*): calculated for $C_{16}H_{24}Si_1$ $[M^{+\bullet}]$: 244.1642, found: 244.1639.

(4-fluorophenyl)(methyl)bis(2-methylallyl)silane (1n)

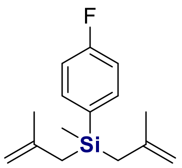

^{1}H NMR (501 MHz, CD_2Cl_2) δ 7.56–7.48 (m, 2H), 7.09–7.02 (m, 2H), 4.59 (dq, *J* = 2.8, 1.4 Hz, 2H), 4.47 (dt, *J* = 1.9, 1.0 Hz, 2H), 1.83 (d, *J* = 1.0 Hz, 4H), 1.57 (t, *J* = 1.1 Hz, 6H), 0.37 (s, 3H).

^{13}C NMR (126 MHz, CD_2Cl_2) δ 164.2 (d, *J* = 247.4 Hz), 143.3, 136.4 (d, *J* = 7.3 Hz), 133.7 (d, *J* = 4.1 Hz), 115.1 (d, *J* = 19.8 Hz), 109.6, 26.6, 25.4, –4.9.

^{19}F NMR (471 MHz, CD_2Cl_2) δ –112.8.

R_f = 0.34 (hexanes).

EI-HRMS (*m/z*): calculated for $C_{15}H_{21}Si_1F_1$ $[M^{+\bullet}]$: 248.1391, found: 248.1388.

methylbis(2-methylallyl)(thiophen-2-yl)silane (1o)

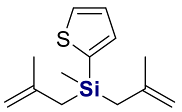

^{1}H NMR (501 MHz, CD_2Cl_2) δ 7.64 (dd, *J* = 4.6, 0.9 Hz, 1H), 7.32 (dd, *J* = 3.3, 0.9 Hz, 1H), 7.20 (dd, *J* = 4.6, 3.3 Hz, 1H), 4.62 (dq, *J* = 2.7, 1.4 Hz, 2H), 4.52 (dq, *J* = 2.1, 1.0 Hz, 2H), 1.87 (t, *J* = 1.1 Hz, 4H), 1.61 (t, *J* = 1.1 Hz, 6H), 0.41 (s, 3H).

^{13}C NMR (126 MHz, CD_2Cl_2) δ 143.1, 135.6, 131.3, 128.5, 109.8, 27.4, 25.4, –3.6.

R_f = 0.60 (hexanes).

CI-HRMS (*m/z*): calculated for $C_{13}H_{21}S_1Si_1$ ($[M+H]^+$): 237.1128, found: 237.1124.

methyltris(2-methylallyl)silane (1p)

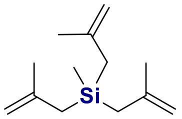

^{1}H NMR (501 MHz, CD_2Cl_2) δ 4.63 (dq, *J* = 2.8, 1.4 Hz, 3H), 4.52 (dq, *J* = 2.0, 0.9 Hz, 3H), 1.72 (t, *J* = 1.1 Hz, 9H), 1.63 (d, *J* = 1.1 Hz, 6H), 0.10 (s, 3H).

^{13}C NMR (126 MHz, CD_2Cl_2) δ 143.8, 109.3, 26.0, 25.6, –3.9.

R_f = 0.55 (hexanes).

CI-HRMS (*m/z*): calculated for $C_{13}H_{25}Si_1$ ($[M+H]^+$): 209.1720, found: 209.1716.

benzyl(ethyl)bis(2-methylallyl)silane (1q)

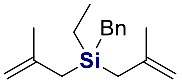

^{1}H NMR (501 MHz, CD_2Cl_2) δ 7.24–7.15 (m, 2H), 7.06 (ddq, *J* = 7.3, 3.5, 2.1, 1.6 Hz, 3H), 4.65 (dd, *J* = 2.5, 1.3 Hz, 2H), 4.54 (dq, *J* = 2.0, 1.0 Hz, 2H), 2.20 (s, 2H), 1.75–1.69 (m, 6H), 1.61 (d, *J* = 1.0 Hz, 4H), 0.95 (t, *J* = 7.9 Hz, 3H), 0.59 (q, *J* = 7.9 Hz, 2H).

^{13}C NMR (126 MHz, CD_2Cl_2) δ 143.7, 140.3, 128.9, 128.6, 124.4, 109.7, 25.7, 23.8, 22.6, 7.5, 4.8.

R_f = 0.28 (hexanes).

CI-HRMS (*m/z*): calculated for $C_{17}H_{27}Si_1$ ($[M+H]^+$): 259.1876, found: 259.1874.

6. Substrate scope for the organocatalytic asymmetric synthesis of tertiary silyl ethers

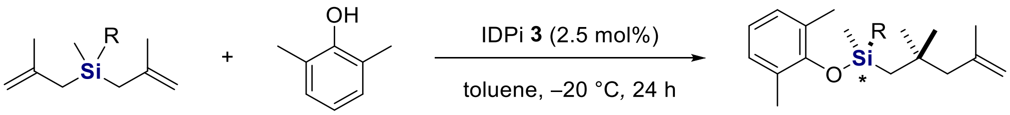

1 **2a** **4**

General procedure for organocatalytic asymmetric synthesis of silyl ethers

The silane starting material **1** (0.3 mmol, 1.5 equiv.) was placed in a 5 mL GC vial, which was equipped with a teflon-coated magnetic stirring bar. IDPi **3a** or **3e** (2.5 mol%) and toluene (0.1 M, 2.0 mL) were added, and the resultant solution was stirred at –20 °C for 10

min before 2,6-dimethylphenol **2a** (0.2 mmol, 1.0 equiv.) was slowly added. The reaction mixture was stirred for an additional 24 h at the indicated temperature. After **2a** was fully consumed, as monitored by TLC, the reaction mixture was treated with triethylamine. All volatiles were removed in vacuo and the crude residue was purified by column chromatography with silica gel, which was pre-neutralized by triethylamine, to afford the desired silyl ethers **4**.

Analytical data of products 4

benzyl(2,6-dimethylphenoxy)(methyl)(2,2,4-trimethylpent-4-en-1-yl)silane 4a

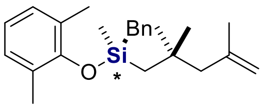

The titled product was purified by column chromatography with hexanes as eluent to afford **4a** as a colorless oil (67.4 mg, 92% yield).

^{1}H NMR (501 MHz, CD_2Cl_2) δ 7.14 (dd, *J* = 8.2, 6.9 Hz, 2H), 7.07–7.01 (m, 1H), 6.96 (d, *J* = 7.5 Hz, 2H), 6.92–6.87 (m, 2H), 6.80 (t, *J* = 7.5 Hz, 1H), 4.84 (dd, *J* = 2.7, 1.4 Hz, 1H), 4.60 (dq, *J* = 2.7, 0.8 Hz, 1H), 2.29– 2.20 (m, 2H), 2.16 (s, 6H), 2.01 (t, *J* = 1.1 Hz, 2H), 1.74 (t, *J* = 1.1 Hz, 3H), 1.06 (d, *J* = 14.8 Hz, 1H), 1.05 (s, 3H), 1.03 (s, 3H), 0.89 (d, *J* = 14.8 Hz, 1H), 0.36 (s, 3H).

^{13}C NMR (126 MHz, CD_2Cl_2) δ 153.0, 144.3, 139.0, 129.1, 129.0, 128.9, 128.5, 124.6, 121.8, 114.5, 54.2, 34.2, 31.4, 30.6, 30.5, 28.0, 25.6, 18.4, 1.6.

R_f = 0.36 (Hexanes).

ESI-HRMS (*m/z*): calculated for $C_{24}H_{34}O_1Si_1Na_1$ ($[M+Na]^+$): 389.2271, found: 389.2272.

HPLC (OD-3, *n*-Heptane = 100, 0.5 mL/min, 298 K, 220 nm): t_{R1}= 12.7 min, t_{R2} = 10.7 min, e.r. = 97:3.

$[\alpha]_D^{25}$ = –11.4 (*c* 1.02, $CHCl_3$).

(*R*)-(2,6-dimethylphenoxy)(methyl)(4-methylbenzyl)(2,2,4-trimethylpent-4-en-1-yl)silane 4b

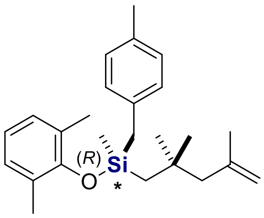

The titled product was purified by column chromatography with hexanes as eluent to afford (*R*)-**4b** as a colorless oil (73.1 mg, 96% yield).

^{1}H NMR (501 MHz, CD_2Cl_2) δ 6.96 (d, *J* = 7.5 Hz, 4H), 6.80 (d, *J* = 7.7 Hz, 3H), 4.84 (dd, *J* = 2.7, 1.4 Hz, 1H), 4.60 (dq, *J* = 2.6, 0.9 Hz, 1H), 2.26 (s, 3H), 2.20 (d, *J* = 4.2 Hz, 2H), 2.17 (s, 6H), 2.01 (s, 2H), 1.74 (t, *J* = 1.1 Hz, 3H), 1.05 (s, 3H), 1.04 (d, *J* = 15.0 Hz, 1H), 1.03 (s, 3H), 0.88 (d, *J* = 15.0 Hz, 1H), 0.35 (s, 3H).

^{13}C NMR (126 MHz, CD_2Cl_2) δ 153.0, 144.3, 135.6, 134.1, 129.2, 129.0, 128.9, 121.7, 114.4, 54.2, 34.2, 31.4, 30.6, 30.5, 27.3, 25.6, 21.0, 18.4, 1.6.

R_f = 0.35 (hexanes).

ESI-HRMS (*m/z*): calculated for $C_{25}H_{36}O_1Si_1Na_1$ ($[M+Na]^+$): 403.2428, found: 403.2429.

HPLC (OD-3, *n*-Heptane = 100, 0.5 mL/min, 298 K, 220 nm): t_{R1}= 12.9 min, t_{R2} = 10.9 min, e.r. = 97:3.

$[\alpha]_D^{25}$ = –15.4 (*c* 1.0, $CHCl_3$).

(2,6-dimethylphenoxy)(4-methoxybenzyl)(methyl)(2,2,4-trimethylpent-4-en-1-yl)silane 4c

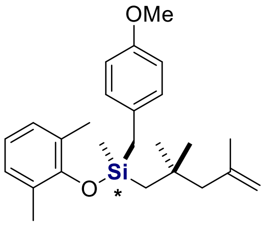

The titled product was purified by column chromatography with hexanes as eluent to afford **4c** as a colorless oil (69.0 mg, 87% yield).

^{1}H NMR (501 MHz, CD_2Cl_2) δ 6.96 (d, *J* = 7.4 Hz, 2H), 6.83–6.77 (m, 3H), 6.72–6.67 (m, 2H), 4.85 (dq, *J* = 2.9, 1.5 Hz, 1H), 4.61 (dq, *J* = 2.6, 0.8 Hz, 1H), 3.73 (s, 3H), 2.18 (d, *J* = 5.5 Hz, 2H), 2.17 (s, 6H), 2.02 (d, *J* = 1.0 Hz, 2H), 1.78–1.71 (m, 3H), 1.06 (s, 3H), 1.04 (d, *J* = 14.9 Hz, 1H), 1.03 (s, 3H), 0.89 (d, *J* = 14.9 Hz, 1H), 0.36 (s, 3H).

^{13}C NMR (126 MHz, CD_2Cl_2) δ 157.3, 153.0, 144.3, 130.7, 129.9, 129.0, 128.9, 121.7, 114.4, 114.0, 55.5, 54.2, 34.2, 31.4, 30.6, 30.6, 26.7, 25.6, 18.4, 1.6.

R_f = 0.61 (Ethyl acetate/hexanes = 1:20).

ESI-HRMS (*m/z*): calculated for $C_{25}H_{36}O_2Si_1Na_1$ ($[M+Na]^+$): 419.2377, found: 419.2380.

HPLC (IB-3, *n*-Heptane = 100, 0.5 mL/min, 298 K, 220 nm): t_{R1}= 18.2 min, t_{R2} = 15.9 min, e.r. = 96:4.

$[\alpha]_D^{25}$ = –17.7 (*c* 0.92, $CHCl_3$).

(2,6-dimethylphenoxy)(4-fluorobenzyl)(methyl)(2,2,4-trimethylpent-4-en-1-yl)silane 4d

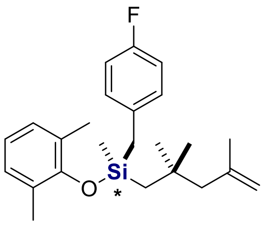

The titled product was purified by column chromatography with hexanes as eluent to afford **4d** as a colorless oil (73.8 mg, 96% yield).

^{1}H NMR (501 MHz, CD_2Cl_2) δ 6.96 (d, *J* = 7.5 Hz, 2H), 6.87–6.76 (m, 5H), 4.85 (dd, *J* = 2.7, 1.4 Hz, 1H), 4.61 (dq, *J* = 2.7, 0.9 Hz, 1H), 2.27–2.17 (m, 2H), 2.15 (s, 6H), 2.02 (t, *J* = 1.1 Hz, 2H), 1.75 (t, *J* = 1.1 Hz, 3H), 1.06 (s, 3H), 1.05 (d, *J* = 14.9 Hz, 1H), 1.04 (s, 3H), 0.89 (d, *J* = 14.9 Hz, 1H), 0.37 (s, 3H).

^{13}C NMR (126 MHz, CD_2Cl_2) δ 160.8 (d, *J* = 242.0 Hz), 152.9, 144.2, 134.7 (d, *J* = 3.3 Hz), 130.2 (d, *J* = 7.7 Hz), 129.0, 128.9, 121.9, 115.1 (d, *J* = 21.2 Hz), 114.5, 54.2, 34.2, 31.4, 30.6, 30.6, 27.0, 25.6, 18.4, 1.6.

^{19}F NMR (471 MHz, CD_2Cl_2) δ –120.7.

R_f = 0.32 (hexanes).

ESI-HRMS (*m/z*): calculated for $C_{24}H_{33}F_1O_1Si_1Na_1$ ($[M+Na]^+$): 407.2177, found: 407.2179.

HPLC (OD-3, *n*-Heptane = 100, 0.5 mL/min, 298 K, 220 nm): t_{R1}= 13.2 min, t_{R2} = 10.8 min, e.r. = 98:2.

$[\alpha]_D^{25}$ = –15.4 (*c* 1.02, $CHCl_3$).

(2,6-dimethylphenoxy)(methyl)(3-methylbenzyl)(2,2,4-trimethylpent-4-en-1-yl)silane 4e

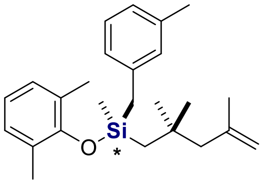

The titled product was purified by column chromatography with hexanes as eluent to afford **4e** as a colorless oil (73.8 mg, 97% yield).

^{1}H NMR (501 MHz, CD_2Cl_2) δ 7.02 (t, *J* = 7.6 Hz, 1H), 6.96 (d, *J* = 7.4 Hz, 2H), 6.85 (d, *J* = 7.6 Hz, 1H), 6.80 (t, *J* = 7.4 Hz, 1H), 6.73–6.68 (m, 1H), 6.61 (d, *J* = 1.9 Hz, 1H), 4.85 (dq, *J* = 2.9, 1.5 Hz, 1H), 4.61 (dq, *J* = 2.6, 0.9 Hz, 1H), 2.20 (s, 3H), 2.19 (s, 2H), 2.16 (s, 6H), 2.02 (d, *J* = 1.2 Hz, 2H), 1.75 (t, *J* = 1.1 Hz, 3H), 1.07 (s, 3H), 1.05 (d, *J* = 14.8 Hz, 1H), 1.04 (s, 3H), 0.90 (d, *J* = 14.8 Hz, 1H), 0.37 (s, 3H).

^{13}C NMR (126 MHz, CD_2Cl_2) δ 153.0, 144.3, 138.7, 138.0, 129.9, 129.0, 129.0, 128.3, 126.1, 125.4, 121.8, 114.4, 54.3, 34.2, 31.5, 30.6, 30.6, 27.8, 25.6, 21.5, 18.4, 1.7.

R_f = 0.26 (hexanes).

ESI-HRMS (*m/z*): calculated for $C_{25}H_{36}O_1Si_1Na_1$ ($[M+Na]^+$): 403.2428, found: 403.2427.

HPLC (OD-3, *n*-Heptane = 100, 0.5 mL/min, 298 K, 220 nm): t_{R1}= 13.5 min, t_{R2} = 11.8 min, e.r. = 93:7.

$[\alpha]_D^{25}$ = –12.7 (*c* 0.98, $CHCl_3$).

(2,6-dimethylphenoxy)(3-methoxybenzyl)(methyl)(2,2,4-trimethylpent-4-en-1-yl)silane 4f

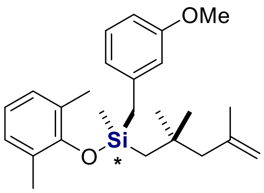

The titled product was purified by column chromatography with hexanes as eluent to afford **4f** as a colorless oil (77.0 mg, 97% yield).

^{1}H NMR (501 MHz, CD_2Cl_2) δ 7.06 (t, *J* = 7.9 Hz, 1H), 6.96 (d, *J* = 7.4 Hz, 2H), 6.80 (t, *J* = 7.5 Hz, 1H), 6.60 (ddd, *J* = 8.2, 2.6, 0.9 Hz, 1H), 6.51 (dt, *J* = 7.6, 1.3 Hz, 1H), 6.44 (t, *J* = 2.1 Hz, 1H), 4.84 (dq, *J* = 2.9, 1.5 Hz, 1H), 4.61 (dq, *J* = 2.6, 0.9 Hz, 1H), 3.69 (s, 3H), 2.25 (d, *J* = 13.9 Hz, 2H), 2.18 (s, 6H), 2.02 (t, *J* = 1.1 Hz, 2H), 1.75 (t, *J* = 1.2 Hz, 3H), 1.06 (d, *J* = 5.0 Hz, 3H), 1.05 (d, *J* = 14.9 Hz, 1H), 1.04 (s, 3H), 0.90 (d, *J* = 14.9 Hz, 1H), 0.37 (s, 3H).

^{13}C NMR (126 MHz, CD_2Cl_2) δ 160.0, 153.0, 144.3, 140.6, 129.4, 129.0, 128.9, 121.8, 121.6, 114.6, 114.5, 110.3, 55.3, 54.2, 34.2, 31.5, 30.6, 30.6, 28.1, 25.6, 18.4, 1.7.

R_f = 0.60 (Ethyl acetate/hexanes = 1:20).

ESI-HRMS (*m/z*): calculated for $C_{25}H_{36}O_2Si_1Na_1$ ($[M+Na]^+$): 419.2377, found: 419.2378.

HPLC (IB-3N, *n*-Heptane = 100, 1.0 mL/min, 298 K, 220 nm): t_{R1}= 11.2 min, t_{R2} = 12.6 min, e.r. = 95.5:4.5.

$[\boldsymbol{\alpha}]_{\mathbf{D}}^{\mathbf{25}}$ = –10.9 (*c* 0.98, $CHCl_3$).

(2,6-dimethylphenoxy)(3-fluorobenzyl)(methyl)(2,2,4-trimethylpent-4-en-1-yl)silane 4g

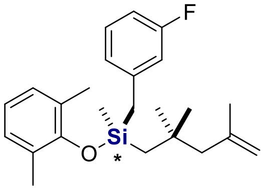

The titled product was purified by column chromatography with hexanes as eluent to afford **4g** as a colorless oil (71.2 mg, 92.5% yield).

^{1}H NMR (501 MHz, CD_2Cl_2) δ 7.11 (td, *J* = 7.9, 6.2 Hz, 1H), 6.97 (d, *J* = 7.4 Hz, 2H), 6.81 (t, *J* = 7.5 Hz, 1H), 6.78–6.72 (m, 1H), 6.69 (dt, *J* = 7.6, 1.3 Hz, 1H), 6.56 (dt, *J* = 10.5, 2.1 Hz, 1H), 4.85 (dq, *J* = 2.9, 1.5 Hz, 1H), 4.61 (dq, *J* = 2.7, 0.9 Hz, 1H), 2.31–2.21 (m, 2H), 2.16 (s, 6H), 2.02 (t, *J* = 1.2 Hz, 2H), 1.75 (t, *J* = 1.2 Hz, 3H), 1.06 (s, 3H), 1.05 (d, *J* = 15.0 Hz, 1H), 1.04 (s, 3H), 0.90 (d, *J* = 15.0 Hz, 1H), 0.39 (s, 3H).

^{13}C NMR (126 MHz, CD_2Cl_2) δ 163.2 (d, *J* = 244.0 Hz), 152.9, 144.2, 141.8 (d, *J* = 7.7 Hz), 129.8 (d, *J* = 9.6 Hz), 129.0, 128.9, 124.9 (d, *J* = 3.1 Hz), 121.9, 115.7 (d, *J* = 20.1 Hz), 114.5, 111.4 (d, *J* = 20.1 Hz), 54.2, 34.2, 31.5, 30.6, 30.6, 28.1, 28.0, 25.6, 18.3, 1.7.

^{19}F NMR (471 MHz, CD_2Cl_2) δ –114.9.

R_f = 0.31 (hexanes).

ESI-HRMS (*m/z*): calculated for $C_{24}H_{33}O_1Si_1F_1Na_1$ ($[M+Na]^+$): 407.2176, found: 407.2180.

HPLC (OD-3, *n*-Heptane = 100, 0.5 mL/min, 298 K, 220 nm): t_{R1}= 12.2 min, t_{R2} = 11.0 min, e.r. = 96:4.

$[\boldsymbol{\alpha}]_{\mathbf{D}}^{\mathbf{25}}$ = –15.7 (*c* 1.02, $CHCl_3$).

(2,6-dimethylphenoxy)(methyl)(2-methylbenzyl)(2,2,4-trimethylpent-4-en-1-yl)silane 4h

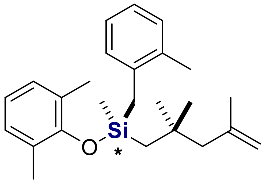

The titled product was purified by column chromatography with hexanes as eluent to afford **4h** as a colorless oil (72.3 mg, 95% yield).

^{1}H NMR (501 MHz, CD_2Cl_2) δ 7.07 (dd, *J* = 6.7, 2.3 Hz, 1H), 7.02–6.94 (m, 4H), 6.86 (dd, *J* = 6.8, 2.2 Hz, 1H), 6.80 (t, *J* = 7.5 Hz, 1H), 4.85 (dq, *J* = 2.9, 1.5 Hz, 1H), 4.61 (dq, *J* = 2.6, 0.9 Hz, 1H), 2.25 (s, 2H), 2.18 (s, 9H), 2.01 (d, *J* = 2.3 Hz, 2H), 1.74 (t, *J* = 1.1 Hz, 3H), 1.10 (d, *J* = 15.0 Hz, 1H), 1.05 (s, 3H), 1.03 (s, 3H), 0.90 (d, *J* = 15.0 Hz, 1H), 0.34 (s, 3H).

^{13}C NMR (126 MHz, CD_2Cl_2) δ 152.9, 144.3, 137.7, 135.5, 130.5, 129.5, 129.0, 128.9, 126.0, 124.9, 121.8, 114.5, 54.3, 34.3, 31.9, 30.6, 30.6, 25.6, 24.4, 20.6, 18.3, 2.1.

R_f = 0.26 (hexanes).

ESI-HRMS (*m/z*): calculated for $C_{25}H_{36}O_1Si_1Na_1$ ($[M+Na]^+$): 403.2428, found: 403.2431.

HPLC (OD-3, *n*-Heptane = 100, 0.5 mL/min, 298 K, 220 nm): t_{R1} = 12.1 min, t_{R2} = 11.0 min, e.r. = 94:6.

$[\alpha]_D^{25}$ = –11.2 (c 0.94, $CHCl_3$).

(2,6-dimethylphenoxy)(2-fluorobenzyl)(methyl)(2,2,4-trimethylpent-4-en-1-yl)silane 4i

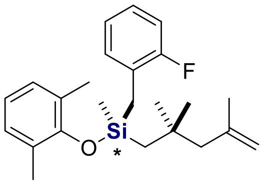

The titled product was purified by column chromatography with hexanes as eluent to afford **4i** as a colorless oil (69.2 mg, 90% yield).

^{1}H NMR (501 MHz, CD_2Cl_2) δ 7.05 (dddd, J = 8.8, 7.2, 5.3, 1.8 Hz, 1H), 6.99–6.96 (m, 2H), 6.96–6.90 (m, 2H), 6.87–6.78 (m, 2H), 4.84 (dq, J = 2.9, 1.5 Hz, 1H), 4.61 (dq, J = 2.6, 0.8 Hz, 1H), 2.29 (d, J = 1.8 Hz, 2H), 2.18 (s, 6H), 2.03–1.99 (m, 2H), 1.75 (t, J = 1.1 Hz, 3H), 1.10 (d, J = 15.0 Hz, 1H), 1.06 (s, 3H), 1.03 (s, 3H), 0.91 (d, J = 14.9 Hz, 1H), 0.38 (d, J = 1.2 Hz, 3H).

^{13}C NMR (126 MHz, CD_2Cl_2) δ 160.8 (d, J = 242.1 Hz), 152.9, 144.3, 131.3 (d, J = 4.9 Hz), 129.0, 128.9, 126.4 (d, J = 7.8 Hz), 126.2 (d, J = 16.6 Hz), 124.2 (d, J = 3.9 Hz), 121.9, 115.3 (d, J = 22.3 Hz), 114.5, 54.2, 34.2, 31.5, 30.6, 30.5, 25.6, 20.2, 18.3, 1.7, 1.6.

^{19}F NMR (471 MHz, CD_2Cl_2) δ –116.7.

R_f = 0.31 (hexanes).

CI-HRMS (m/z): calculated for $C_{24}H_{32}O_1F_1Si_1$ ($[M–H]^–$): 383.2201, found: 383.2201.

HPLC (OD-3, n-Heptane = 100, 0.5 mL/min, 298 K, 220 nm): t_{R1} = 9.9 min, t_{R2} = 8.5 min, e.r. = 95:5.

$[\alpha]_D^{25}$ = –11.7 (c 1.0, $CHCl_3$).

(3,4-dimethylbenzyl)(2,6-dimethylphenoxy)(methyl)(2,2,4-trimethylpent-4-en-1-yl)silane 4j

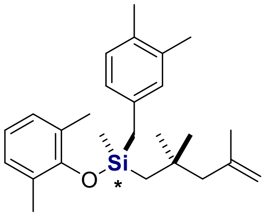

The titled product was purified by column chromatography with hexanes as eluent to afford **4j** as a colorless oil (73.4 mg, 93% yield).

^{1}H NMR (501 MHz, CD_2Cl_2) δ 6.96 (d, J = 7.4 Hz, 2H), 6.90 (d, J = 7.6 Hz, 1H), 6.80 (t, J = 7.4 Hz, 1H), 6.65 (dd, J = 7.7, 2.0 Hz, 1H), 6.59 (d, J = 2.0 Hz, 1H), 4.85 (dd, J = 2.7, 1.4 Hz, 1H), 4.62 (dd, J = 2.5, 1.1 Hz, 1H), 2.17 (s, 3H), 2.17 (s, 6H), 2.16 (s, 2H), 2.13 (s, 3H), 2.02 (s, 2H), 1.75 (t, J = 1.2 Hz, 3H), 1.07 (s, 3H), 1.04 (s, 3H), 1.04 (d, J = 15.0 Hz, 1H), 0.91 (d, J = 15.0 Hz, 1H), 0.36 (s, 3H).

^{13}C NMR (126 MHz, CD_2Cl_2) δ 153.1, 144.3, 136.5, 136.0, 132.7, 130.4, 129.6, 129.0, 126.4, 121.7, 114.4, 54.3, 34.2, 31.5, 30.6, 30.6, 27.2, 25.6, 19.8, 19.3, 18.4, 1.6.

R_f = 0.34 (hexanes).

ESI-HRMS (m/z): calculated for $C_{26}H_{38}O_1Na_1Si_1$ ($[M+Na]^+$): 417.2584, found: 417.2580.

HPLC (OD-3, n-Heptane = 100, 0.5 mL/min, 298 K, 220 nm): t_{R1} = 12.3 min, t_{R2} = 10.7 min, e.r. = 93.5:6.5.

$[\alpha]_D^{25}$ = –13.6 (c 1.02, $CHCl_3$).

(2,6-dimethylphenoxy)(methyl)(naphthalen-2-ylmethyl)(2,2,4-trimethylpent-4-en-1-yl)silane 4k

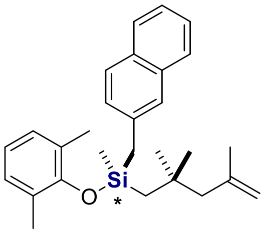

The titled product was purified by column chromatography with hexanes as eluent to afford **4k** as a colorless oil (69.2 mg, 83% yield).

^{1}H NMR (501 MHz, CD_2Cl_2) δ 7.75 (dd, J = 8.0, 1.3 Hz, 1H), 7.65 (d, J = 8.4 Hz, 1H), 7.61 (dd, J = 8.1, 1.3 Hz, 1H), 7.38 (dddd, J = 20.7, 8.1, 6.8, 1.4 Hz, 2H), 7.22 (d, J = 1.8 Hz, 1H), 7.08 (dd, J = 8.4, 1.8 Hz, 1H), 6.98 (d, J = 7.4 Hz, 2H), 6.84 (t, J = 7.4 Hz, 1H), 4.85 (dq, J = 2.9, 1.5 Hz, 1H), 4.62 (dt, J = 2.7, 0.9 Hz, 1H), 2.47–2.36 (m, 2H), 2.17 (s, 6H), 2.04 (s, 2H), 1.75 (t, J = 1.1 Hz, 3H), 1.13 (d, J = 14.9 Hz, 1H), 1.09 (s, 3H), 1.06 (s, 3H), 0.95 (d, J = 14.9 Hz, 1H), 0.39 (s, 3H).

^{13}C NMR (126 MHz, CD_2Cl_2) δ 153.0, 144.3, 136.8, 134.1, 131.6, 129.1, 129.0, 128.6, 127.8, 127.5, 126.5, 126.1, 124.9, 121.9, 114.5, 54.3, 34.3, 31.6, 30.7, 30.6, 28.2, 25.6, 18.4, 1.8.

R_f = 0.32 (hexanes).

ESI-HRMS (*m/z*): calculated for $C_{28}H_{36}O_1Si_1Na_1$ ($[M+Na]^+$): 439.2428, found: 439.2433.

HPLC (IB-3, *n*-Heptane = 100, 0.5 mL/min, 298 K, 220 nm): t_{R1} = 17.7 min, t_{R2} = 16.8 min, e.r. = 95:5.

$[\alpha]_D^{25}$ = –20.4 (*c* 0.90, $CHCl_3$).

(2,6-dimethylphenoxy)(methyl)(phenyl)(2,2,4-trimethylpent-4-en-1-yl)silane 4l

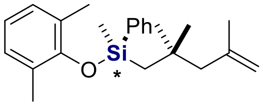

The titled product was purified by column chromatography with hexanes as eluent to afford **4l** as a colorless oil (58.2 mg, 82.5% yield).

^{1}H NMR (501 MHz, CD_2Cl_2) δ 7.62–7.55 (m, 2H), 7.42–7.36 (m, 1H), 7.34 (dd, *J* = 7.9, 6.3 Hz, 2H), 6.90 (d, *J* = 7.4 Hz, 2H), 6.74 (t, *J* = 7.4 Hz, 1H), 4.85 (dt, *J* = 3.0, 1.5 Hz, 1H), 4.64–4.59 (m, 1H), 2.05 (s, 6H), 2.00 (s, 2H), 1.75 (t, *J* = 1.1 Hz, 3H), 1.43–1.25 (m, 2H), 0.98 (s, 3H), 0.93 (s, 3H), 0.56 (s, 3H).

^{13}C NMR (126 MHz, CD_2Cl_2) δ 153.0, 144.3, 138.9, 134.0, 129.9, 129.0, 128.8, 128.1, 121.7, 114.4, 54.2, 34.1, 32.1, 30.6, 25.6, 18.5, 0.1.

R_f = 0.19 (hexanes).

CI-HRMS (*m/z*): calculated for $C_{23}H_{33}O_1Si_1$ ($[M+H]^+$): 353.2295, found: 353.2293.

HPLC (OJ-3R, MeOH : H_2O = 80:20, 1 mL/min, 298 K, 190 nm): t_{R1} = 29.5 min, t_{R2} = 34.0 min, e.r. = 95:5.

$[\alpha]_D^{25}$ = 32.8 (*c* 0.98, $CHCl_3$).

(2,6-dimethylphenoxy)(methyl)(p-tolyl)(2,2,4-trimethylpent-4-en-1-yl)silane 4m

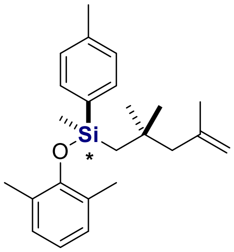

The titled product was purified by column chromatography with hexanes as eluent to afford **4m** as a colorless oil (61.6 mg, 84% yield).

^{1}H NMR (501 MHz, CD_2Cl_2) δ 7.51–7.44 (m, 2H), 7.16 (dt, *J* = 7.3, 0.8 Hz, 2H), 6.89 (d, *J* = 7.4 Hz, 2H), 6.73 (t, *J* = 7.4 Hz, 1H), 4.84 (dt, *J* = 2.9, 1.5 Hz, 1H), 4.61 (dq, *J* = 2.6, 0.9 Hz, 1H), 2.34 (s, 3H), 2.06 (s, 6H), 2.00 (s, 2H), 1.75 (t, *J* = 1.1 Hz, 3H), 1.32 (d, *J* = 9.4 Hz, 2H), 0.98 (s, 3H), 0.92 (s, 3H), 0.55 (s, 3H).

^{13}C NMR (126 MHz, CD_2Cl_2) δ 153.1, 144.4, 140.0, 135.2, 134.1, 129.0, 128.9, 128.8, 121.6, 114.4, 54.2, 34.1, 32.2, 30.6, 25.6, 21.6, 18.5, 0.2.

R_f = 0.17 (hexanes).

EI-HRMS (*m/z*): calculated for $C_{24}H_{34}O_1Si_1$ $[M^{+\bullet}]$: 366.2373, found: 366.2372.

HPLC (OJ-3R, MeOH : H_2O = 80:20, 1 mL/min, 298 K, 220 nm): t_{R1} = 33.0 min, t_{R2} = 39.9 min, e.r. = 95:5.

$[\alpha]_D^{25}$ = 31.0 (*c* 0.42, CH_3CN).

(2,6-dimethylphenoxy)(4-fluorophenyl)(methyl)(2,2,4-trimethylpent-4-en-1-yl)silane 4n

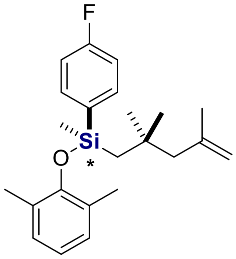

The titled product was purified by column chromatography with hexanes as eluent to afford **4n** as a colorless oil (57.8 mg, 78% yield).

^{1}H NMR (501 MHz, CD_2Cl_2) δ 7.62–7.55 (m, 2H), 7.09–7.00 (m, 2H), 6.90 (d, *J* = 7.4 Hz, 2H), 6.75 (t, *J* = 7.4 Hz, 1H), 4.85 (dd, *J* = 2.7, 1.4 Hz, 1H), 4.62 (dq, *J* = 2.6, 0.8 Hz, 1H), 2.04 (s, 6H), 2.00 (d, *J* = 0.8 Hz, 2H), 1.75 (dd, *J* = 1.4, 0.9 Hz, 3H), 1.33 (d, *J* = 7.5 Hz, 2H), 0.98 (s, 3H), 0.92 (s, 3H), 0.55 (s, 3H).

^{13}C NMR (126 MHz, CD_2Cl_2) δ 164.4 (d, *J* = 248.6 Hz), 152.9, 144.3, 136.2 (d, *J* = 7.7 Hz), 134.7 (d, *J* = 4.4 Hz), 129.0, 128.9, 121.8, 115.2 (d, *J* = 19.6 Hz), 114.5, 54.2, 34.1, 32.2, 30.6, 25.6, 18.5, 0.1.

^{19}F NMR (471 MHz, CD_2Cl_2) δ −111.7.

R_f = 0.17 (hexanes).

EI-HRMS (*m/z*): calculated for $C_{23}H_{31}O_1Si1F_1$ $[M^{+\bullet}]$: 370.2123, found: 370.2122.

HPLC (OJ-3R, MeOH : H_2O = 80:20, 1 mL/min, 298 K, 220 nm): t_{R1} = 30.3 min, t_{R2} = 33.4 min, e.r. = 95:5.

$[\alpha]_D^{25}$ = 27.5 (*c* 0.56, CH_3CN).

(2,6-dimethylphenoxy)(methyl)(thiophen-2-yl)(2,2,4-trimethylpent-4-en-1-yl)silane 4o

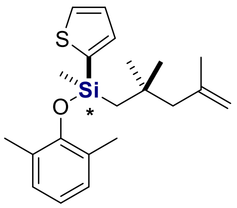

The titled product was purified by column chromatography with hexanes as eluent to afford **4o** as a colorless oil (56.7 mg, 79% yield).

^1H NMR (501 MHz, CD_2Cl_2) δ 7.63 (dd, *J* = 4.7, 0.9 Hz, 1H), 7.34 (dd, *J* = 3.4, 0.9 Hz, 1H), 7.17 (dd, *J* = 4.6, 3.4 Hz, 1H), 6.91 (d, *J* = 7.4 Hz, 2H), 6.75 (t, *J* = 7.4 Hz, 1H), 4.87 (dd, *J* = 2.7, 1.4 Hz, 1H), 4.64 (dq, *J* = 2.6, 0.9 Hz, 1H), 2.10 (s, 6H), 2.05 (d, *J* = 1.1 Hz, 2H), 1.77 (t, *J* = 1.1 Hz, 3H), 1.44–1.29 (m, 2H), 1.06 (s, 3H), 1.00 (s, 3H), 0.68 (s, 3H).

^{13}C NMR (126 MHz, CD_2Cl_2) δ 152.6, 144.3, 138.0, 135.9, 131.8, 129.1, 128.9, 128.4, 121.9, 114.6, 54.1, 34.2, 32.9, 30.6, 30.5, 25.6, 18.3, 2.1.

R_f = 0.43 (hexanes).

ESI-HRMS (*m/z*): calculated for $C_{21}H_{30}O_1S_1Si_1Na_1$ ($[M+Na]^+$): 381.1679, found: 381.1682.

HPLC (OD-3, *n*-Heptane = 100, 0.5 mL/min, 298 K, 220 nm): t_{R1} = 10.4 min, t_{R2} = 12.2 min, e.r. = 95:5.

$[\alpha]_D^{25}$ = 29.7 (*c* 1.04, $CHCl_3$).

(2,6-dimethylphenoxy)(methyl)(2-methylallyl)(2,2,4-trimethylpent-4-en-1-yl)silane 4p

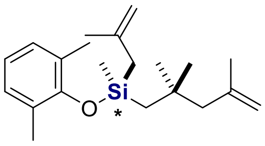

The titled product was purified by column chromatography with hexanes as eluent to afford **4p** as a colorless oil (50.2 mg, 76% yield).

^1H NMR (501 MHz, CD_2Cl_2) δ 6.96 (d, *J* = 7.4 Hz, 2H), 6.78 (t, *J* = 7.5 Hz, 1H), 4.85 (dd, *J* = 2.7, 1.4 Hz, 1H), 4.63 (ddd, *J* = 2.9, 2.1, 1.1 Hz, 2H), 4.48 (dq, *J* = 1.9, 0.9 Hz, 1H), 2.22 (s, 6H), 2.03 (dd, *J* = 3.0, 0.8 Hz, 2H), 1.76 (t, *J* = 1.1 Hz, 3H), 1.73 (d, *J* = 1.1 Hz, 2H), 1.71 (t, *J* = 1.1 Hz, 3H), 1.05 (s, 6H), 1.03 (d, *J* = 15.0 Hz, 1H), 0.93 (d, *J* = 14.8 Hz, 1H), 0.46 (s, 3H).

^{13}C NMR (126 MHz, CD_2Cl_2) δ 153.0, 144.3, 142.8, 128.9, 128.9, 121.7, 114.4, 110.1, 54.2, 34.1, 31.8, 30.6, 30.5, 29.7, 25.6, 25.5, 18.4, 1.8.

R_f = 0.22 (hexanes).

ESI-HRMS (*m/z*): calculated for $C_{21}H_{34}O_1Na_1Si_1$ ($[M+Na]^+$): 353.2271, found: 353.2276.

HPLC (OD-3, *n*-Heptane = 100, 0.5 mL/min, 298 K, 220 nm): t_{R1} = 6.2 min, t_{R2} = 6.6 min, e.r. = 76:24.

$[\alpha]_D^{25}$ = –8.8 (*c* 0.95, CH_3CN).

benzyl(2,6-dimethylphenoxy)(ethyl)(2,2,4-trimethylpent-4-en-1-yl)silane 4q

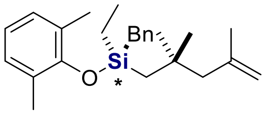

The titled product was purified by column chromatography with hexanes as eluent to afford **4q** as a colorless oil (68.5 mg, 90% yield).

^1H NMR (501 MHz, CD_2Cl_2) δ 7.12 (dd, *J* = 8.2, 6.9 Hz, 2H), 7.06–7.00 (m, 1H), 6.97–6.89 (m, 4H), 6.79 (t, *J* = 7.4 Hz, 1H), 4.84 (dq, *J* = 2.8, 1.5 Hz, 1H), 4.60 (dt, *J* = 2.7, 0.9 Hz, 1H), 2.41–2.28 (m, 2H), 2.16 (s, 6H), 2.00 (s, 2H), 1.74 (t, *J* = 1.1 Hz, 3H), 1.08–1.00 (m, 7H), 0.99–0.91 (m, 4H), 0.90–0.82 (m, 2H).

^{13}C NMR (126 MHz, CD_2Cl_2) δ 153.2, 144.3, 139.1, 129.2, 129.0, 128.8, 128.5, 124.6, 121.7, 114.5, 54.4, 34.2, 30.5, 30.5, 29.7, 26.3, 25.6, 18.3, 9.4, 7.2.

R_f = 0.24 (hexanes).

ESI-HRMS (*m/z*): calculated for $C_{25}H_{36}O_1Si_1Na_1$ ($[M+Na]^+$): 403.2428, found: 403.2433.

HPLC (OD-3, *n*-Heptane = 100, 1 mL/min, 293 K, 220 nm): t_{R1} = 5.0 min, t_{R2} = 5.4 min, e.r. = 70.5:29.5.

benzyl(2,6-dimethylphenoxy)(methyl)(2-methylallyl)silane 5a

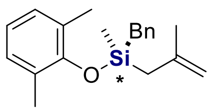

The titled product was purified by column chromatography with hexanes as eluent to afford **5a** as a colorless oil (trace).

^1H NMR (501 MHz, CD_2Cl_2) δ 7.16 (dd, *J* = 8.2, 6.9 Hz, 2H), 7.09–7.03 (m, 1H), 6.96 (td, *J* = 6.0, 2.7 Hz, 4H), 6.81 (t, *J* = 7.5 Hz, 1H), 4.67 (dq, *J* = 2.8, 1.5 Hz, 1H), 4.53 (dq, *J* = 2.0, 1.0 Hz, 1H), 2.34 (s, 2H), 2.15 (s, 6H), 1.83–1.74 (m, 2H), 1.71 (t, *J* = 1.1 Hz, 3H), 0.27 (s, 3H).

^{13}C NMR (126 MHz, CD_2Cl_2) δ 152.8, 142.4, 138.6, 129.1, 129.0, 128.9, 128.6, 124.8, 122.0, 110.5, 27.5, 26.1, 25.5, 18.2, –2.0.

R_f = 0.36 (hexanes).

EI-HRMS (*m/z*): calculated for $C_{20}H_{26}O_1Si_1$ $[M^{+\bullet}]$: 310.1747, found: 310.1747.

(2,6-dimethylphenoxy)(methyl)(2-methylallyl)(phenyl)silane 5b

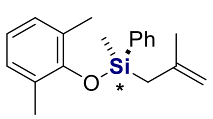

The titled product was purified by column chromatography with hexanes as eluent to afford **5b** as a colorless oil (5.9 mg, 10% yield).

^1H NMR (501 MHz, CD_2Cl_2) δ 7.65–7.58 (m, 2H), 7.44–7.38 (m, 1H), 7.38–7.31 (m, 2H), 6.92 (d, *J* = 7.4 Hz, 2H), 6.76 (t, *J* = 7.5 Hz, 1H), 4.63 (dt, *J* = 2.8, 1.4 Hz, 1H), 4.51 (dq, *J* = 2.1, 1.0 Hz, 1H), 2.10 (d, *J* = 2.5 Hz, 8H), 1.62 (t, *J* = 1.1 Hz, 3H), 0.52 (s, 3H).

^{13}C NMR (126 MHz, CD_2Cl_2) δ 152.8, 142.2, 137.0, 134.1, 130.2, 128.9, 128.9, 128.1, 121.9, 110.6, 28.3, 25.4, 18.2, –2.8.

R_f = 0.19 (hexanes).

CI-HRMS (*m/z*): calculated for $C_{19}H_{25}O_1Si_1$ ($[M+H]^+$): 297.1669, found: 297.1664.

1-benzyl-1,3,3-trimethyl-5-methylenesilinane 17a

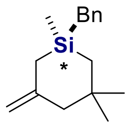

The titled product was purified by column chromatography with hexanes as eluent to afford **17a** as a colorless oil (9.8 mg, 20% yield).

^1H NMR (501 MHz, CD_2Cl_2) δ 7.23–7.16 (m, 2H), 7.08–7.04 (m, 1H), 7.02–6.98 (m, 2H), 4.67 (dt, *J* = 2.7, 1.3 Hz, 1H), 4.54 (dt, *J* = 2.8, 0.8 Hz, 1H), 2.19–2.07 (m, 2H), 1.97–1.84 (m, 2H), 1.60–1.46 (m, 2H), 1.01 (s, 3H), 0.93 (s, 3H), 0.71–0.51 (m, 2H), –0.01 (s, 3H).

^{13}C NMR (126 MHz, CD_2Cl_2) δ 146.5, 140.4, 128.6, 128.6, 124.4, 109.5, 52.1, 34.4, 33.8, 31.4, 26.8, 25.9, 25.3, –3.4.

R_f = 0.38 (hexanes).

EI-HRMS (*m/z*): calculated for $C_{16}H_{24}Si_1$ $[M^{+\bullet}]$: 244.1642, found: 244.1639.

HPLC (OJ-3R, Acetonitrile: Water = 50:50, 1.0 mL/min, 298 K, 220 nm): t_{R1} = 50.4 min, t_{R2} = 58.7 min, e.r. = 53:47.

1,3,3-trimethyl-5-methylene-1-phenylsilinane 17b

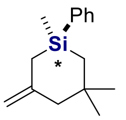

^{1}H NMR (501 MHz, CD_2Cl_2) δ 7.58–7.50 (m, 2H), 7.38–7.30 (m, 3H), 4.75 (dt, J = 2.7, 1.3 Hz, 1H), 4.58 (dt, J = 2.8, 0.8 Hz, 1H), 2.04–1.97 (m, 2H), 1.91 (d, J = 13.1 Hz, 1H), 1.77 (d, J = 13.2 Hz, 1H), 1.00 (s, 3H), 0.94 (d, J = 14.8 Hz, 1H), 0.91 (s, 3H), 0.85–0.79 (m, 1H), 0.30 (s, 3H).

^{13}C NMR (126 MHz, CD_2Cl_2) δ 146.4, 139.5, 134.0, 129.3, 128.1, 109.9, 52.2, 34.5, 33.9, 31.3, 27.4, 25.5, –2.1.

R_f = 0.66 (hexanes).

EI-HRMS (m/z): calculated for $C_{15}H_{22}Si_1$ [$M^{+\bullet}$]: 230.1485, found: 230.1482.

Limitations of the method:

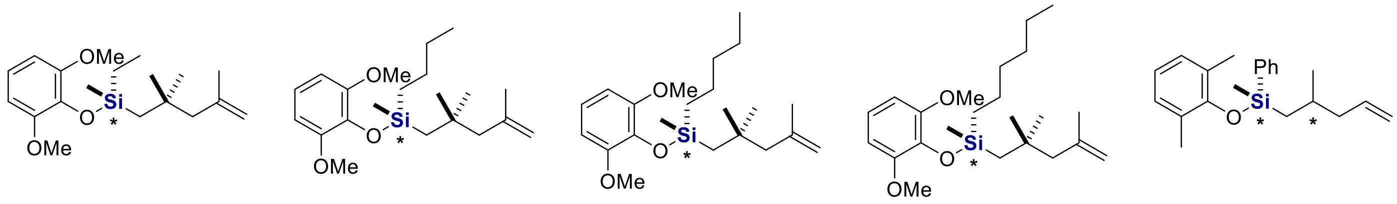

62%, 63:37 e.r. | 54%, 74:26 e.r. | 61%, 73:27 e.r. | 49%, 71:29 e.r. | Not detected

Procedure: The silicon starting material (0.3 mmol, 1.5 equiv.) was placed in a 5 mL GC vial, which was equipped with a teflon-coated magnetic stirring bar. IDPi **3a** (2.5 mol%) and toluene (0.1 M, 2.0 mL) were added, and the resultant solution was stirred at −20 °C for 10 min before 2,6-dimethoxyphenol (0.2 mmol, 1.0 equiv.) was slowly added. The reaction mixture was stirred for an additional 24 h at the indicated temperature. After 2,6-dimethoxyphenol was fully consumed, as monitored by TLC, the reaction mixture was treated with triethylamine. All volatiles were removed in vacuo and the crude residue was purified by column chromatography with silica gel, which was pre-neutralized by triethylamine, to afford the desired silyl ethers.

7. Optimization of the reaction with aromatic silane 1l with 2a

Table S3. **Screening of the reaction conditions:**[a]

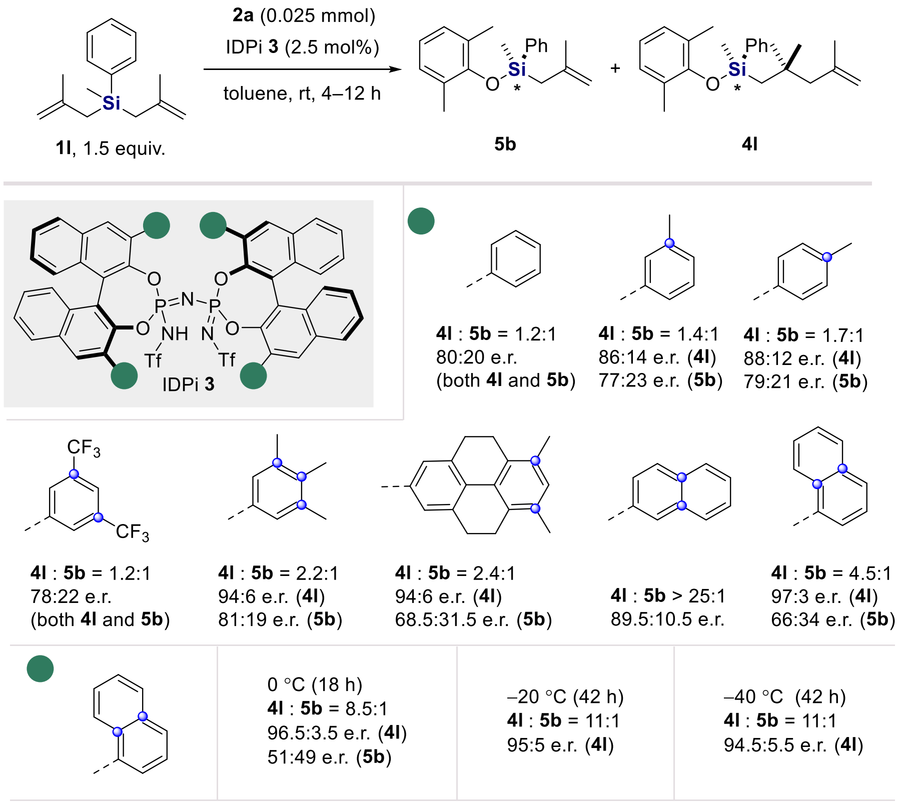

[a]Reactions were performed with 2,6-dimethylphenol **2a** (0.025 mmol), symmetrical silane **1l** (1.5 equiv.), and IDPi catalysts (2.5 mol%) in toluene (0.25 mL, 0.1 M). The ratios of **4l**:**5b** were determined by NMR analysis. Enantiomeric ratios (e.r.) were determined by HPLC.

Prochiral diallyl silane starting material **1l** (0.0375 mmol, 1.5 equiv.) was placed in a 1.5 mL GC vial, which was equipped with a teflon-coated magnetic stirring bar. IDPi catalysts (2.5 mol%) and toluene (0.1 M, 0.25 mL) were added, and the resultant solution was stirred for 5 min at rt. 2,6-Dimethylphenol **2a** (0.025 mmol, 1.0 equiv.) was slowly added and the reaction mixture was stirred for 4–42 h at the indicated temperature. After the ketone was fully consumed, as monitored by TLC, the reaction mixture was treated with triethylamine. Volatiles were removed in vacuo, then the ratio of the desired product and the side product was determined by NMR analysis and the enantiomeric ratio was determined by HPLC after purification by prep. TLC.

*Note: in all cases, full conversion of **2a** was observed.*

8. Gram-scale reaction and derivatizations of 4a

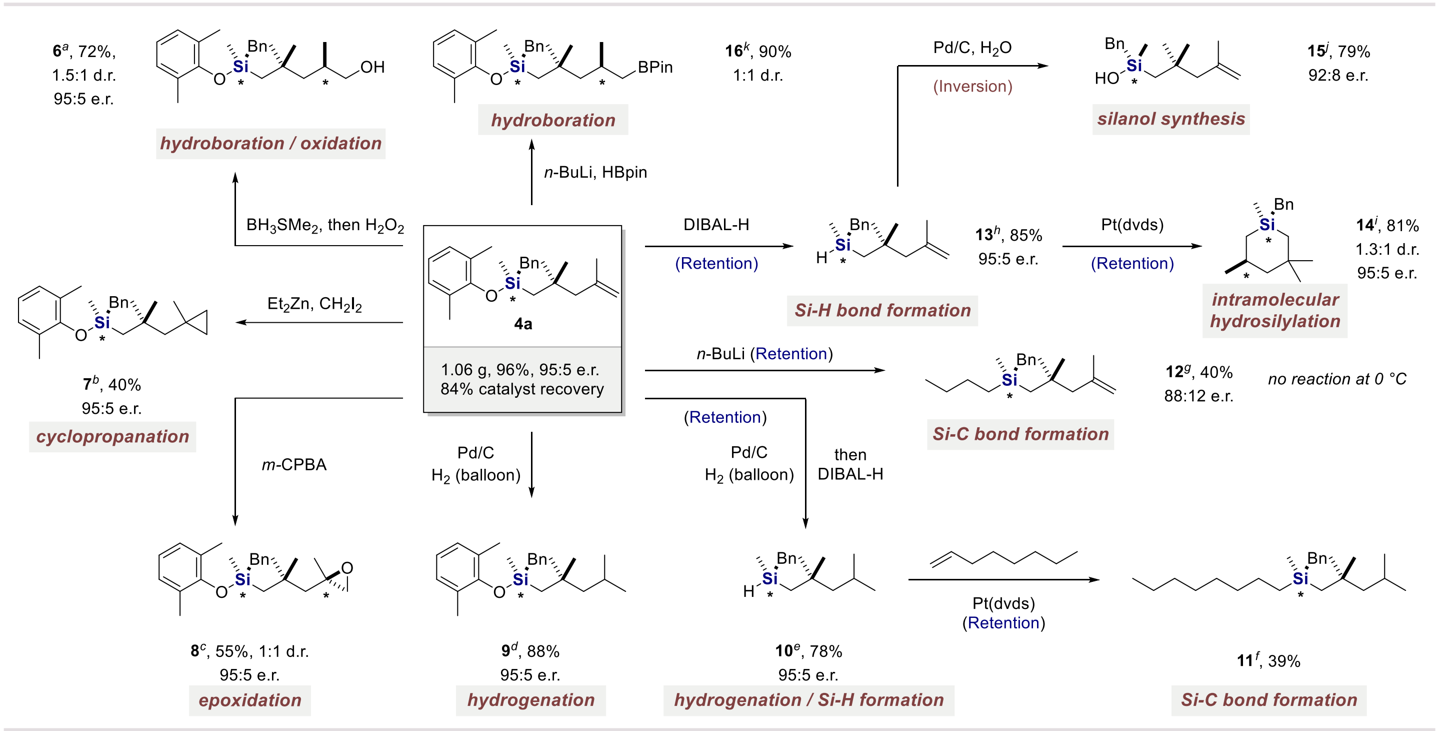

Figure S4. Gram-scale reaction and derivatizations of 4a. Reactions were conducted on a 0.1 mmol scale on **4a**. Isolated yields with e.r. determined by HPLC and d.r. measured by ^{1}H NMR or HPLC. [a]BH_3SMe_2 (1.0 equiv.), 0 °C, THF, 1 h. [b]Et_2Zn (2.0 equiv.), CH_2I_2 (4.0 equiv.), 0 °C–rt, DCE, 18 h. [c]*m*-CPBA (2.0 equiv.), 0 °C–rt, DCM, 7 h. [d]Pd/C (0.1 equiv.), H_2 (balloon), rt, MeOH, 12 h. [e]Pd/C (0.1 equiv.), H_2 (balloon), rt, MeOH, 12 h, then DIBAL-H (2.0 equiv.), 0 °C–rt, hexanes, 18 h. [f]Pt(dvds) (0.05 equiv.), 1-octene (2.0 equiv.), 50 °C, hexanes, 48 h. [g]*n*-BuLi (3.0 equiv.), 0–35 °C, THF, 48 h. [h]DIBAL-H (2.0 equiv.), 0 °C–rt, hexanes, 18 h. [i]Pt(dvds) (0.05 equiv.), 50 °C, hexanes, 48 h. [j]Pd/C (0.1 equiv.), H_2O (3.0 equiv.), 0 °C, ethyl acetate, 24 h. [k]*n*-BuLi (0.1 equiv.), HBPin (3.0 equiv.), 130 °C, toluene, 18 h.

Gram-scale catalytic reaction of silane 1a with 2,6-dimethylphenol 2a.

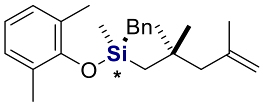

The silane starting material **1a** (0.99 mL, 3.6 mmol) was placed in a round-bottom flask, which was equipped with a teflon-coated magnetic stirring bar. IDPi **3e** (51 mg, 0.03 mmol, 1.0 mol%) and solvent (0.2 M, 15.0 mL) were added, and the resultant solution was stirred at –20 °C for 10 min before 2,6-dimethylphenol **2a** (366 mg, 3 mmol) was slowly added. The reaction mixture was stirred for an additional 42 h at the indicated temperature. After the phenol nucleophile was fully consumed, as monitored by TLC, the reaction mixture was treated with triethylamine. All volatiles were removed in vacuo and the crude residue was purified by column chromatography with silica gel, which was pre-neutralized by

triethylamine, to afford the desired silane product **4a** (1.06 g, 96% yield, 95:5 e.r.), and catalyst **3e** could be recovered (43mg, 84% yield).

i. Preparation of 5-(benzyl(2,6-dimethylphenoxy)(methyl)silyl)-2,4,4-trimethylpentan-1-ol 6

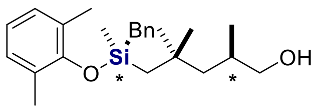

According to a known procedure with minor modification[6]

To a solution of silane **4a** (37 μL, 0.1 mmol) in THF at 0 °C, $BH_3{\cdot}SMe_2$ (10 μL, 0.1 mmol, 1 equiv.) was slowly added and the mixture was stirred at this temperature. After 1 h, EtOH (48 μL), NaOH (50 μL, 1M in H_2O) and H_2O_2 (24 μL, 30% v/v in H_2O) were added subsequently. The resulting mixture was stirred for 3 h at the room temperature. After the reaction completed (monitored by TLC), the reaction was quenched with aqueous NH_4Cl (1 mL) and extracted with diethyl ether (3×3 mL). The organic layers were combined, dried over anhydrous Na_2SO_4. The solvent was removed under reduced pressure, and purified by column chromatography (ethyl acetate/hexanes 1:5) affording product **6** as colorless oil (27.7 mg, 72% yield, 95:5 e.r.).

There were two diastereomers formed, d.r. = 1.5:1

^{1}H NMR (501 MHz, CD_2Cl_2) δ 7.14 (t, *J* = 7.6 Hz, 2H), 7.04 (t, *J* = 7.4 Hz, 1H), 6.96 (d, *J* = 7.4 Hz, 2H), 6.91 (d, *J* = 8.2 Hz, 2H), 6.80 (t, *J* = 7.4 Hz, 1H), 3.37 (dtd, *J* = 11.1, 5.7, 2.3 Hz, 1H), 3.24 (dddd, *J* = 10.4, 7.2, 5.4, 3.4 Hz, 1H), 2.28 (d, *J* = 14.0 Hz, 1H), 2.23 (d, *J* = 14.0 Hz, 1H), 2.17 (s, 6H), 1.60 (qd, *J* = 6.5, 3.8 Hz, 1H), 1.32–1.26 (m, 3H), 1.03 (d, *J* = 3.8 Hz, 3H), 1.01 (d, *J* = 4.9 Hz, 3H), 0.92 (dd, *J* = 6.7, 2.2 Hz, 3H), 0.88–0.84 (m, 2H), 0.36 (s, 3H).

^{13}C NMR (126 MHz, CD_2Cl_2) δ 153.0, 139.0, 139.0, 129.1, 129.0, 128.9, 128.5, 124.6, 121.8, 69.6, 49.7, 33.8, 32.6, 31.6, 31.6, 30.5, 30.4, 30.4, 28.0, 19.7, 18.4, 1.7.

R_f = 0.52 (Ethyl acetate/hexanes = 1:4).

ESI-HRMS (*m/z*): calculated for $C_{24}H_{36}O_2Si_1Na_1$ ($[M+Na]^+$): 407.2381, found: 407.2377.

HPLC (AD-3, *n*-heptane/*i*-PrOH = 99.5:0.5, 1.0 mL/min, 298 K, 220 nm): for major diastereomer, t_{R1} = 11.7 min, t_{R2} = 14.0 min, e.r. = 95:5. for minor diastereomer, t_{R1} = 10.7 min, t_{R2} = 12.6 min, e.r. = 95:5.

$[\alpha]_D^{25}$ = –8.2 (*c* 0.71, $CHCl_3$).

ii. Preparation of benzyl(2,2-dimethyl-3-(1-methylcyclopropyl)propyl)(2,6-dimethylphenoxy)(methyl)silane 7

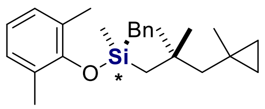

According to a known procedure with minor modification[7]

To a solution of silane **4a** (37 μL, 0.1 mmol) in 1,2-dichloroethane (1 mL), diethyl zinc (1 M in hexane, 0.2 mL, 0.2 mmol, 2 equiv.) and diiodomethane (32 μL, 0.4 mmol, 4 equiv.) were added subsequently at 0 °C. The resultant mixture was stirred for 1 h at 0 °C and then for 17 h at room temperature. After the reaction completed (monitored by TLC), the reaction was quenched with aqueous NH_4Cl (1 mL) and extracted with diethyl ether (3 × 3 mL). The organic layers were combined, dried over anhydrous Na_2SO_4. The solvent was removed under reduced pressure and purified by column chromatography (ethyl acetate/hexanes = 1:20) affording product **7** as a colorless oil (15.0 mg, 40% yield, 95:5 e.r.).

^{1}H NMR (501 MHz, CD_2Cl_2) δ 7.14 (dd, *J* = 8.3, 6.9 Hz, 2H), 7.07–7.02 (m, 1H), 6.96 (d, *J* = 7.4 Hz, 2H), 6.92 (d, *J* = 6.6 Hz, 2H), 6.79 (t, *J* = 7.4 Hz, 1H), 2.27 (d, *J* = 14.0 Hz, 1H), 2.23 (d, *J* = 14.0 Hz, 1H), 2.17 (s, 6H), 1.27 (s, 2H), 1.12–1.05 (m, 7H), 1.04 (s, 3H), 0.93 (d, *J* = 15.0 Hz, 1H), 0.35 (s, 3H), 0.25–0.20 (m, 2H), 0.20–0.15 (m, 2H).

^{13}C NMR (126 MHz, CD_2Cl_2) δ 153.0, 139.1, 129.1, 129.0, 128.9, 128.5, 124.6, 121.7, 55.6, 35.5, 32.7, 31.4, 31.3, 28.1, 25.3, 18.4, 14.6, 14.2, 1.8.

R_f = 0.12 (Hexanes).

ESI-HRMS (*m/z*): calculated for $C_{25}H_{36}O_1Si_1Na_1$ ($[M+Na]^+$): 403.2430, found: 403.2428.

HPLC (IB-3, *n*-heptane, 1.0 mL/min, 298 K, 220 nm): t_{R1} = 7.6 min, t_{R2} = 8.3 min, e.r. = 95:5.

$[\alpha]_D^{25}$ = –8.3 (*c* 0.75, $CHCl_3$).

iii. Preparation of benzyl(2,2-dimethyl-3-(2-methyloxiran-2-yl)propyl)(2,6-dimethylphenoxy)(methyl)silane 8

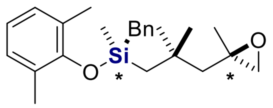

According to a known procedure with minor modification[1, 8]

To a solution of silane **4a** (37 μL, 0.1 mmol) in DCM (1 mL), *m*-CPBA (34 mg, 0.2 mmol, 2 equiv.) was added at 0 °C. The reaction mixture was stirred for 1 h at this temperature and then for 6 h at room temperature. After the reaction completed (monitored by TLC), the reaction mixture was quenched with aqueous 1M NaOH (0.2 mL) and extracted with DCM (3 × 3 mL). The organic layers were combined and dried over Na_2SO_4. The solvent was removed under reduced pressure and purified by column chromatography (ethyl acetate/hexanes 1:10) affording product **8** as a colorless oil (21.0 mg, 55% yield, 95:5 e.r.).

There were two diastereomers formed, d.r. = 1:1

^{1}H NMR (501 MHz, CD_2Cl_2) δ 7.14 (dd, *J* = 8.2, 6.9 Hz, 2H), 7.08–7.01 (m, 1H), 6.96 (d, *J* = 7.6 Hz, 2H), 6.90 (d, *J* = 6.8 Hz, 2H), 6.80 (t, *J* = 7.4 Hz, 1H), 2.52 (t, *J* = 4.9 Hz, 1H), 2.48 (dt, *J* = 5.0, 1.7 Hz, 1H), 2.27 (dd, *J* = 14.0, 2.7 Hz, 1H), 2.23 (d, *J* = 12.3 Hz, 1H), 2.17 (s, 6H), 1.77 (ddd, *J* = 14.0, 2.3, 1.4 Hz, 1H), 1.34 (d, *J* = 14.0 Hz, 1H), 1.30 (s, 3H), 1.13 (d, *J* = 4.6 Hz, 3H), 1.10 (d, *J* = 5.0 Hz, 3H), 0.96–0.87 (m, 2H), 0.36 (s, 3H).

^{13}C NMR (126 MHz, CD_2Cl_2) δ 152.9, 138.9, 129.1, 129.0, 128.9, 128.5, 124.7, 121.9, 55.7, 54.8, 53.4, 33.8, 32.4, 32.4, 31.3, 31.3, 31.1, 31.0, 27.9, 23.8, 23.7, 22.8, 18.4, 14.2, 1.7. (due to the inseparable carbon signals of these two diastereomers, all the peaks are presented in this case)

R_f = 0.38 (Ethyl acetate/hexanes = 1:10).

ESI-HRMS (*m/z*): calculated for $C_{24}H_{34}O_2Si_1Na_1$ ($[M+Na]^+$): 405.2221, found: 405.2220.

SFC (OJ-3, CO_2:*i*-PrOH = 99:1, 1.0 mL/min, 313 K, 220 nm): for major diastereomer, t_{R1} = 11.9 min, t_{R2} = 14.1 min, e.r. = 95:5. for minor diastereomer, t_{R1} = 11.9 min, t_{R2} = 14.1 min, e.r. = 95:5.

$[\alpha]_D^{25}$ = –6.2 (*c* 0.78, $CHCl_3$).

iv. Preparation of benzyl(2,6-dimethylphenoxy)(methyl)(2,2,4-trimethylpentyl)silane 9

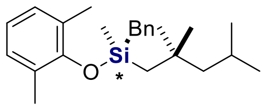

To a solution of silane **4a** (37 μL, 0.1 mmol) in methanol (1 mL), Pd/C (10 wt%, 10 mg, 0.01 mmol, 0.1 equiv.) was added. The flask was filled with hydrogen gas (balloon). The resulting mixture was stirred for 12 h at room temperature. After the reaction completed (monitored by TLC), the reaction mixture was filtered through silica gel and the solvent was removed under reduced pressure. Subsequently, the residue was purified by column chromatography (hexane) affording product **9** as a colorless oil (32.3 mg, 88% yield, 95:5 e.r.).

^{1}H NMR (501 MHz, CD_2Cl_2) δ 7.14 (dd, *J* = 8.3, 6.9 Hz, 2H), 7.08–7.00 (m, 1H), 6.96 (d, *J* = 7.6 Hz, 2H), 6.91 (d, *J* = 6.8 Hz, 2H), 6.80 (t, *J* = 7.4 Hz, 1H), 2.28 (d, *J* = 14.1 Hz, 1H), 2.23 (d, *J* = 14.0 Hz, 1H), 2.17 (s, 6H), 1.63 (dtd, *J* = 11.9, 6.7, 1.5 Hz, 1H), 1.37–1.21 (m, 2H), 1.20 (d, *J* = 1.8 Hz, 1H), 1.19 (d, *J* = 2.0 Hz, 1H), 1.02 (s, 3H), 1.00 (s, 3H), 0.89 (d, *J* = 2.2 Hz, 3H), 0.88 (d, *J* = 2.3 Hz, 3H), 0.35 (s, 3H).

^{13}C NMR (126 MHz, $CDCl_3$) δ 153.0, 139.1, 129.1, 129.0, 128.9, 128.5, 124.6, 121.7, 55.5, 34.0, 31.7, 30.7, 30.6, 28.1, 25.7, 24.9, 18.4, 1.7.

R_f = 0.30 (Hexanes).

ESI-HRMS (*m/z*): calculated for $C_{24}H_{36}O_1Si_1Na_1$ ($[M+Na]^+$): 391.2430, found: 391.2428.

HPLC (OD-3, *n*-heptane, 0.5 mL/min, 298 K, 220 nm): t_{R1} = 8.8 min, t_{R2} = 10.6 min, e.r. = 95:5.

$[\alpha]_D^{25}$ = –14.9 (*c* 1.0, $CHCl_3$).

v. Preparation of benzyl(methyl)(2,2,4-trimethylpentyl)silane 10

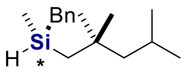

According to a known procedure with minor modification[9]

To a solution of silane **4a** (75 µL, 0.2 mmol) in methanol (1 mL), Pd/C (10 wt%, 10 mg, 0.01 mmol, 0.1 equiv.) was added. The flask was filled with hydrogen gas (balloon). The resulting mixture was stirred for 12 h at room temperature. After the reaction completed (monitored by TLC), the reaction mixture was filtered through silica gel and the solvent was removed under reduced pressure. To a solution of resulting crude in hexane (1 mL), DIBAL-H (1 M in hexane, 0.6 mL, 0.6 mmol, 3 equiv.) was added dropwise at 0 °C and stirred for 18 h at room temperature. After the reaction completed (monitored by TLC), the reaction was quenched with aqueous NH_4Cl (1 mL) and extracted with hexanes (3 × 3 mL). The organic layers were combined, dried over anhydrous Na_2SO_4. The solvent was removed under reduced pressure and purified by column chromatography (hexanes) affording product **10** as a colorless oil (38.8 mg, 78% yield, 95:5 e.r.).

^{1}H NMR (501 MHz, CD_2Cl_2) δ 7.21 (dd, *J* = 8.4, 6.9 Hz, 2H), 7.08–7.03 (m, 3H), 4.00 (dtd, *J* = 6.5, 3.9, 2.7 Hz, 1H), 2.19 (dd, *J* = 13.7, 2.8 Hz, 1H), 2.10 (dd, *J* = 13.6, 4.0 Hz, 1H), 1.64 (dddd, *J* = 13.4, 11.9, 6.7, 5.2 Hz, 1H), 1.19 (d, *J* = 5.3 Hz, 2H), 0.96 (s, 3H), 0.95 (s, 3H), 0.90 (d, *J* = 2.2 Hz, 3H), 0.89 (d, *J* = 2.2 Hz, 3H), 0.81 (dd, *J* = 14.7, 2.5 Hz, 1H), 0.68 (dd, *J* = 14.7, 4.1 Hz, 1H), 0.05 (d, *J* = 3.7 Hz, 3H).

^{13}C NMR (126 MHz, $CDCl_3$) δ 140.9, 128.7, 128.6, 124.5, 54.6, 33.9, 30.5, 30.4, 29.0, 25.7, 25.6, 24.9, 24.6, –4.1

R_f = 0.80 (Hexanes).

EI-HRMS (*m/z*): calculated for $C_{16}H_{28}Si_1$ $[M^{+\bullet}]$: 248.1950, found: 248.1955.

HPLC (OJ-3R, Acetonitrile/Water = 70:30, 1.0 mL/min, 298 K, 220 nm): t_{R1} = 9.7 min, t_{R2} = 10.6 min, e.r. = 95:5.

$[\alpha]_D^{25}$ = 4.5 (*c* 1.0, $CHCl_3$).

vi. Preparation of benzyl(methyl)(octyl)(2,2,4-trimethylpentyl)silane 11

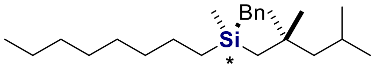

According to a known procedure with minor modification[10]

To a solution of hydrosilane **10** (24.8 mg, 0.1 mmol) in hexane (1 mL), 1-octene (31 µL, 0.2 mmol, 2 equiv.) Pt(dvds) (ca. Pt, 3% in xylene, 20 µL, 0.005 mmol, 0.05 equiv.) were added and stirred for 48 h at room temperature. After the reaction completed (monitored by TLC), the solvent was removed under reduced pressure, and purified by column chromatography (hexanes) affording product **11** as colorless oil (14.1 mg, 39% yield).

Note: For this compound, no separation method was available for HPLC or GC analysis. From Hou's work, we speculate the e.r. of the product could be the same as the starting material.[10]

^{1}H NMR (501 MHz, CD_2Cl_2) δ 7.18 (t, *J* = 7.7 Hz, 2H), 7.06–7.02 (m, 1H), 7.00 (d, *J* = 6.8 Hz, 2H), 2.10 (s, 2H), 1.71–1.55 (m, 2H), 1.35–1.22 (m, 14H), 1.16 (d, *J* = 5.3 Hz, 2H), 0.95 (d, *J* = 4.0 Hz, 6H), 0.91–0.87 (m, 8H), 0.70 (d, *J* = 5.0 Hz, 2H), 0.00 (s, 3H).

^{13}C NMR (126 MHz, $CDCl_3$) δ 128.7, 128.4, 124.1, 55.5, 34.5, 34.2, 32.4, 30.8, 29.7, 29.7, 29.6, 26.3, 25.7, 24.9, 24.2, 23.1, 15.8, 14.3, –2.5.

R_f = 0.63 (Hexanes).

EI-HRMS (*m/z*): calculated for $C_{24}H_{45}Si_1$ ($[M+H]^+$): 361.3282, found: 361.3285.

$[\alpha]_D^{25}$ = –8.6 (*c* 0.65, $CHCl_3$).

vii. Preparation of benzyl(butyl)(methyl)(2,2,4-trimethylpent-4-en-1-yl)silane 12

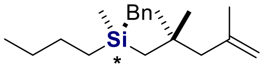

According to a known procedure with minor modification[11]

To a solution of silane **4a** (37 μL, 0.1 mmol) in Et_2O (1 mL), *n*-BuLi (2.5 M in hexanes, 120 μL, 0.3 mmol, 3 equiv.) was added dropwise at 0 °C and stirred for 48 h at 35 °C. After the reaction completed (monitored by TLC), the reaction was quenched with aqueous NH_4Cl (1 mL) and extracted with ethyl acetate (3 × 3 mL). The organic layers were combined, dried over anhydrous Na_2SO_4. The solvent was removed under reduced pressure and purified by column chromatography (hexanes) affording product **12** as a colorless oil (12.1 mg, 40% yield).

^{1}H NMR (501 MHz, CD_2Cl_2) δ 7.23–7.12 (m, 2H), 7.04 (td, *J* = 7.4, 1.4 Hz, 1H), 7.02–6.97 (m, 2H), 4.84 (dq, *J* = 2.9, 1.5 Hz, 1H), 4.61 (dq, *J* = 2.7, 0.8 Hz, 1H), 2.11 (s, 2H), 1.98 (s, 2H), 1.76 (s, 3H), 1.32–1.22 (m, 4H), 0.98 (s, 3H), 0.97 (s, 3H), 0.87 (t, *J* = 7.1 Hz, 3H), 0.74 (d, *J* = 4.5 Hz, 2H), 0.56–0.50 (m, 2H), 0.02 (s, 3H).

^{13}C NMR (126 MHz, $CDCl_3$) δ 144.5, 141.1, 128.7, 128.4, 124.2, 114.2, 54.1, 34.7, 30.6, 29.7, 27.1, 26.5, 26.2, 25.6, 15.4, 13.9, –2.5.

R_f = 0.58 (Hexanes).

CI-HRMS (*m/z*): calculated for $C_{20}H_{38}Si_1N_1$ ($[M+NH_4]^+$): 320.2764, found: 320.2768.

HPLC (OJ-3R, MeOH /Water = 85:15, 1.0 mL/min, 298 K, 220 nm): t_{R1} = 15.7 min, t_{R2} = 14.5 min, e.r. = 88:12.

$[\alpha]_D^{25}$ = –3.7 (*c* 0.60, $CHCl_3$).

viii. Preparation of benzyl(methyl)(2,2,4-trimethylpent-4-en-1-yl)silane 13

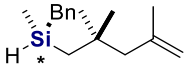

According to a known procedure with minor modification[9]

To a solution of silane **4a** (75 μL, 0.2 mmol) in hexane (1 mL), DIBAL-H (1 M in hexane, 0.6 mL, 0.6 mmol, 3 equiv.) was added dropwise at 0 °C and stirred for 18 h at room temperature. After the reaction completed (monitored by TLC), the reaction was quenched with aqueous NH_4Cl (1 mL) and extracted with hexanes (3 × 3 mL). The organic layers were combined, dried over anhydrous Na_2SO_4. The solvent was removed under reduced pressure and purified by column chromatography (hexanes) affording product **13** as a colorless oil (41.9 mg, 85% yield, 95:5 e.r.).

^{1}H NMR (501 MHz, CD_2Cl_2) δ 7.21 (dd, *J* = 8.4, 6.9 Hz, 2H), 7.12–6.97 (m, 3H), 4.84 (dq, *J* = 2.9, 1.4 Hz, 1H), 4.63–4.60 (m, 1H), 4.03 (dtd, *J* = 6.5, 3.9, 2.7 Hz, 1H), 2.20 (dd, *J* = 13.7, 2.9 Hz, 1H), 2.11 (dd, *J* = 13.7, 4.0 Hz, 1H), 2.00 (s, 2H), 1.76 (s, 3H), 0.98 (s, 3H), 0.98 (s, 3H), 0.84 (dd, *J* = 14.6, 2.5 Hz, 1H), 0.70 (dd, *J* = 14.6, 4.1 Hz, 1H), 0.06 (d, *J* = 3.7 Hz, 3H).

^{13}C NMR (126 MHz, $CDCl_3$) δ 144.4, 140.8, 128.6, 128.6, 124.5, 114.3, 53.2, 34.1, 30.4, 30.3, 28.8, 25.6, 24.5, –4.1.

R_f = 0.80 (Hexanes).

CI-HRMS (*m/z*): calculated for $C_{16}H_{25}Si_1$ ($[M–H]^–$): 245.1716, found: 245.1720.

HPLC (OJ-3R, MeOH/Water = 85:15, 1.0 mL/min, 298 K, 220 nm): t_{R1} = 17.8 min, t_{R2} = 19.5 min, e.r. = 95:5.

$[\alpha]_D^{25}$ = 7.9 (*c* 0.92, $CHCl_3$).

ix. Preparation of 1-benzyl-1,3,3,5-tetramethylsilinane 14

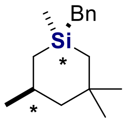

According to a known procedure with minor modification[10]

To a solution of hydrosilane **13** (24.6 mg, 0.1 mmol) in hexane (1 mL), Pt(dvds) (ca. Pt, 3% in xylene, 20 μL, 0.005 mmol, 0.05 equiv.) was added and stirred for 48 h at room temperature. After the reaction completed (monitored by TLC), the

solvent was removed under reduced pressure, and purified by column chromatography (hexanes) affording product **14** as colorless oil (20.0 mg, 81% yield, 95:5 e.r.).

There were two diastereomers formed, d.r. = 1.3:1, for the major product:

^{1}H NMR (501 MHz, CD_2Cl_2) δ 7.23–7.18 (m, 2H), 7.08–7.03 (m, 2H), 6.98 (d, J = 6.5 Hz, 1H), 2.23 (d, J = 2.5 Hz, 2H), 1.89–1.80 (m, 1H), 1.34 (dq, J = 13.5, 2.0 Hz, 1H), 1.30–1.26 (m, 1H), 1.06 (s, 3H), 0.98 (d, J = 6.5 Hz, 3H), 0.91 (s, 3H), 0.90–0.80 (m, 1H), 0.71–0.66 (m, 2H), 0.30 (d, J = 14.5 Hz, 1H), –0.16 (s, 3H).

^{13}C NMR (126 MHz, CD_2Cl_2) δ 141.0, 128.6, 128.5, 124.2, 78.0, 51.9, 38.0, 33.1, 28.8, 28.1, 26.9, 26.7, 25.9, 21.4, –2.6.

R_f = 0.57 (Hexanes).

EI-HRMS (*m/z*): calculated for $C_{16}H_{26}Si_1$ [$M^{+\bullet}$]: 246.1795, found: 246.1798.

HPLC (OJ-3R, Acetonitrile/Water = 65:35, 1.0 mL/min, 298 K, 220 nm): for major diastereomer, t_{R1} = 10.0 min, t_{R2} = 10.7 min, e.r. = 95:5, for minor diastereomer, t_{R1} = 11.8 min, t_{R2} = 12.7 min, e.r. = 95:5.

$[\alpha]_D^{25}$ = –4.2 (*c* 1.0, $CHCl_3$)

For the minor product:

^{1}H NMR (501 MHz, CD_2Cl_2) δ 7.23–7.18 (m, 2H), 7.08–7.03 (m, 2H), 6.98 (d, J = 6.5 Hz, 1H), 2.03 (s, 2H), 1.78–1.68 (m, 1H), 0.99 (s, 3H), 0.97 (s, 3H), 0.95 (d, J = 6.5 Hz, 3H), 0.90–0.80 (m, 2H), 0.57 (dt, J = 14.5, 2.0 Hz, 1H), 0.39 (d, J = 14.5 Hz, 1H), 0.81 (d, J = 13.0 Hz, 1H), 0.06 (s, 3H), 0.02 (d, J = 14.5 Hz, 1H).

^{13}C NMR (126 MHz, CD_2Cl_2) δ 140.8, 128.6, 128.5, 124.2, 78.0, 51.8, 38.0, 33.1, 28.8, 28.1, 26.9, 26.6, 26.0, 21.6, 2.7.

x. Preparation of benzyl(methyl)(2,2,4-trimethylpent-4-en-1-yl)silanol 15

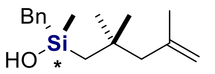

According to a known procedure with minor modification[12]

To a solution of hydrosilane **13** (24.6 mg, 0.1 mmol) in ethyl acetate (1 mL), Pd/C (10 wt%, 10 mg, 0.01 mmol, 0.1 equiv.) and water (5 μL, 0.3 mmol, 3 equiv.) was added subsequently at 0 °C. The reaction mixture was stirred for 24 h at this temperature. After the reaction completed (monitored by TLC), the reaction mixture was filtered through silica gel and dried over Na_2SO_4. The solvent was removed under vacuo and purified by column chromatography (ethyl acetate/hexanes 1:5) affording product **15** as a colorless oil (20.7 mg, 79% yield, 92:8 e.r.).

^{1}H NMR (501 MHz, CD_2Cl_2) δ 7.23–7.20 (m, 2H), 7.09–7.05 (m, 3H), 4.85–4.84 (m, 1H), 4.63–4.62 (m, 1H), 2.19 (d, J = 13.5 Hz, 1H), 2.12 (d, J = 13.5 Hz, 1H), 2.02 (s, 2H), 1.77 (s, 3H), 1.61 (s, 1H) 1.03 (s, 6H), 0.84 (d, J = 15.0 Hz, 1H), 0.76 (d, J = 15.0 Hz, 1H), 0.14 (s, 3H).

^{13}C NMR (126 MHz, $CDCl_3$) δ 144.5, 139.8, 128.7, 128.6, 124.6, 114.3, 34.3, 31.9, 30.7, 30.6, 29.0, 25.6, 0.5.

R_f = 0.45 (Ethyl acetate/hexanes = 1:5).

EI-HRMS (*m/z*): calculated for $C_{16}H_{26}O_1Si_1Na_1$ ($[M+Na]^+$): 285.1648, found: 285.1645.

HPLC (IE-3, *n*-heptane/*i*-PrOH = 99:1, 1.0 mL/min, 298 K, 220 nm): t_{R1} = 4.2 min, t_{R2} = 8.3 min, e.r. = 92:8.

$[\alpha]_D^{25}$ = 7.5 (*c* 1.04, $CHCl_3$).

xi. benzyl(2,6-dimethylphenoxy)(methyl)(2,2,4-trimethyl-5-(4,4,5,5-tetramethyl-1,3,2-dioxaborolan-2-yl)pentyl)silane 16

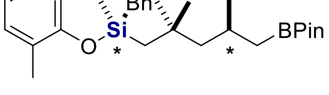

According to a known procedure with minor modification[13]

HBpin (87 μL, 0.6 mmol, 3 equiv.) and toluene (1 mL) were placed in a flame-dried Schlenk flask, equipped with a Teflon-coated magnetic stirring bar. *n*-BuLi (2.5 M in hexane, 8 μL, 0.02 mmol, 0.1

equiv.) was added and stirred for 5 min at 25 °C. Silane **4a** (75 μL, 0.2 mmol) was then added and stirred at 130 °C for 18 h. After the reaction completed (monitored by TLC), the reaction was quenched with aqueous NH_4Cl (1 mL) and extracted with ethyl acetate (3 × 3 mL). The organic layers were combined, dried over anhydrous Na_2SO_4. The solvent was removed under reduced pressure, and purified by column chromatography (ethyl acetate/hexanes 1:20) affording product **16** as colorless oil (89.5 mg, 90% yield).

Note: For this compound, no separation method was available for HPLC analysis.

There were two diastereomers formed, d.r. = 1:1

^{1}H NMR (501 MHz, CD_2Cl_2) δ 7.14 (dd, *J* = 8.3, 6.9 Hz, 2H), 7.07–6.98 (m, 1H), 6.96 (d, *J* = 7.4 Hz, 2H), 6.93–6.83 (m, 2H), 6.79 (t, *J* = 7.4 Hz, 1H), 2.28 (d, *J* = 13.9 Hz, 1H), 2.24 (d, *J* = 14.0 Hz, 1H), 2.17 (s, 6H), 1.77 (ddtd, *J* = 8.6, 7.1, 5.8, 4.5 Hz, 1H), 1.29 (t, *J* = 3.9 Hz, 1H), 1.24 (s, 1H), 1.21 (s, 12H), 1.02 (s, 3H), 1.00 (s, 3H), 0.92 (dd, *J* = 6.6, 2.4 Hz, 3H), 0.90–0.83 (m, 2H), 0.79 (dd, *J* = 14.7, 4.8 Hz, 1H), 0.65 (dd, *J* = 15.4, 8.2 Hz, 1H), 0.35 (s, 3H).

^{13}C NMR (126 MHz, CD_2Cl_2) δ 153.1, 139.2, 129.1, 129.0, 128.9, 128.5, 124.6, 121.8, 83.1, 56.2, 34.1, 32.1, 30.8, 30.7, 28.1, 26.4, 25.4, 25.2, 25.1, 23.2 (C–B), 18.5, 1.8, 1.7.

R_f = 0.42 (Ethyl acetate/hexanes = 1:20).

ESI-HRMS (*m/z*): calculated for $C_{30}H_{47}O_3B_1Si_1Na_1$ ([M+Na]$^+$): 517.3287, found: 517.3280.

$[\alpha]_D^{25}$ = –11.1 (*c* 0.99, $CHCl_3$).

N-((11b*S*)-4-(((11b*S*)-2,6-bis(9,9-dimethyl-9H-fluoren-2-yl)-4-oxidodinaphtho[2,1-d:1',2'-f][1,3,2]dioxaphosphepin-4-yl)imino)-2,6-bis(9,9-dimethyl-9H-fluoren-2-yl)-4l5-dinaphtho[2,1-d:1',2'-f][1,3,2]dioxaphosphepin-4-yl)-1,1,1-trifluoromethanesulfonamide

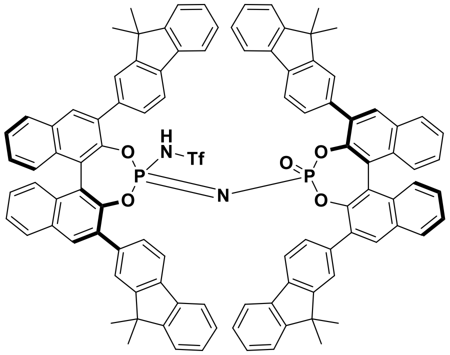

^{1}H NMR (501 MHz, CD_2Cl_2) δ 8.16 (d, *J* = 8.2 Hz, 1H), 8.12 (d, *J* = 8.2 Hz, 1H), 8.07 (t, *J* = 9.0 Hz, 4H), 7.90 (dd, *J* = 8.2, 4.8 Hz, 2H), 7.88–7.82 (m, 2H), 7.72 (dddd, *J* = 19.1, 8.3, 6.8, 1.3 Hz, 2H), 7.66 (d, *J* = 7.5 Hz, 1H), 7.60 (dddd, *J* = 13.7, 8.2, 6.6, 1.2 Hz, 2H), 7.54 (dd, *J* = 7.6, 6.0 Hz, 2H), 7.52–7.49 (m, 1H), 7.49–7.44 (m, 3H), 7.42 (dt, *J* = 7.9, 1.3 Hz, 3H), 7.40–7.37 (m, 2H), 7.36 (d, *J* = 1.7 Hz, 2H), 7.35–7.18 (m, 14H), 6.91–6.84 (m, 2H), 6.74 (dd, *J* = 8.0, 1.6 Hz, 1H), 6.68 (d, *J* = 7.9 Hz, 1H), 6.29 (dd, *J* = 7.9, 1.6 Hz, 1H), 6.15 (dd, *J* = 7.9, 1.7 Hz, 1H), 1.52 (s, 3H), 1.39 (s, 3H), 1.35 (s, 3H), 1.32 (s, 3H), 1.29 (s, 3H), 1.23 (s, 3H), 1.19 (s, 3H), 1.14 (s, 3H).

^{13}C NMR (126 MHz, CD_2Cl_2) δ 154.8, 154.7, 154.4, 154.2, 154.2, 153.9, 153.8, 153.7, 144.8, 144.7, 144.6, 144.6, 144.2, 144.1, 143.3, 143.2, 139.2, 139.2, 139.1, 139.0, 139.0, 138.6, 135.6, 135.5, 135.3, 135.1, 135.0, 135.0, 134.9, 134.9, 134.8, 134.7, 134.3, 134.3, 132.3, 132.3, 132.2, 132.2, 132.1, 132.1, 132.0, 131.2, 131.1, 129.4, 129.4, 129.3, 129.0, 128.9, 128.8, 127.9, 127.9, 127.9, 127.6, 127.5, 127.5, 127.4, 127.4, 127.4, 127.2, 127.1, 127.1, 127.1, 126.9, 126.8, 126.6, 124.5, 124.1, 124.1, 124.0, 124.0, 123.8, 123.8, 123.2, 123.0, 123.0, 122.9, 122.9, 122.8, 122.8, 122.4, 122.4, 120.7, 120.5, 120.2, 120.1, 119.7, 119.6, 119.5, 27.9, 27.4, 27.2, 27.0, 27.0, 26.9, 26.8, 26.7.

^{31}P NMR (203 MHz, CD_2Cl_2) δ –6.5 (d, *J* = 129.3 Hz, 1P), –10.8 (d, *J* = 129.3 Hz, 1P).

^{19}F NMR (471 MHz, CD_2Cl_2) δ –80.4.

R_f = 0.21 (Ethyl acetate/hexanes = 1:4).

ESI-HRMS (*m/z*): calculated for $C_{101}H_{72}N_2O_7S_1P_2F_3$ ([M-H]$^-$): 1575.4493, found: 1575.4500.

$[\alpha]_D^{25}$ = 308.2 (*c* 0.34, $CHCl_3$).

(11b*S*,11b'*S*)-4,4'-iminobis(2,6-bis(9,9-dimethyl-9H-fluoren-2-yl)-4l4-dinaphtho[2,1-d:1',2'-f][1,3,2]dioxaphosphepine 4-oxide)

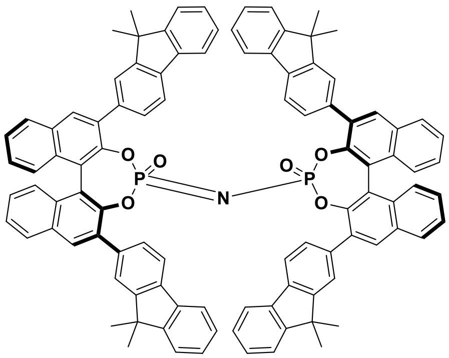

^{1}H NMR (501 MHz, CD_2Cl_2) δ 8.23 (d, *J* = 8.1 Hz, 2H), 8.16 (d, *J* = 10.6 Hz, 2H), 7.97 (d, *J* = 8.3 Hz, 2H), 7.92 (s, 2H), 7.86 (s, 2H), 7.80 (t, *J* = 7.5 Hz, 2H), 7.74 (d, *J* = 7.7 Hz, 2H), 7.67 (t, *J* = 7.9 Hz, 2H), 7.64–7.52 (m, 7H), 7.47–7.39 (m, 6H), 7.37–7.29 (m, 9H), 7.25 (t, *J* = 7.4 Hz, 2H), 7.20 (d, *J* = 7.8 Hz, 2H), 6.50 (s, 2H), 6.18 (s, 2H), 5.84 (s, 2H), 1.64–1.42 (m, 12H), 1.15 (s, 6H), 0.71–0.49 (m, 6H).

^{13}C NMR (126 MHz, CD_2Cl_2) δ 155.3, 154.5, 154.3, 153.9, 144.5, 144.3, 139.3, 139.3, 138.8, 138.6, 135.7, 134.9, 133.3, 133.0, 132.8, 132.6, 132.0, 131.8, 130.8, 129.4, 129.2, 128.7, 127.7, 127.6, 127.5, 127.3, 127.2, 127.1, 127.0, 126.9, 126.5, 124.9, 124.8, 124.3, 123.6, 123.0, 122.1, 120.2, 120.1, 120.1, 120.0, 47.6, 46.9, 28.0, 27.1, 26.4, 26.3.

^{31}P NMR (203 MHz, CD_2Cl_2) δ –0.1.

R_f = 0.30 (Ethyl acetate/hexanes = 1:2.3).

ESI-HRMS (*m/z*): calculated for $C_{100}H_{72}N_1O_6P_2$ ($[M-H]^-$): 1444.4840, found: 1444.4855.

$[\alpha]_D^{25}$ = 586.5 (*c* 0.31, $CHCl_3$).

Note: these two catalysts were synthesized according to the procedure described in our recently published work.[14]

9. Mechanistic studies

At first, we conducted a reaction for reaction progress kinetic analysis:

The solution of silane starting material **1l** (0.075 mmol, 1.5 equiv.) and IDPi **3a** (1.0 mol%) in toluene-d_8 (0.1 M, 0.5 mL) was placed in a NMR tube, which was treated with dry-ice bath in ethanol at –20 °C for 10 min before 2,6-dimethylphenol **2a** (0.05 mmol, 1.0 equiv.) was added. The resultant mixture was kept at this temperature for an additional 16.5 h, in which ^{1}H NMR spectra were taken at different time points.

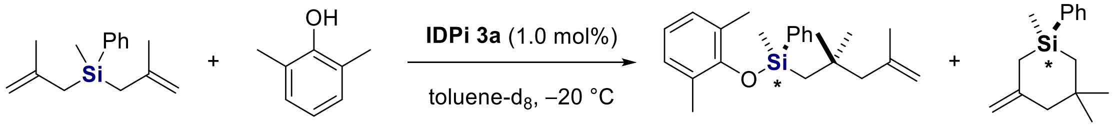

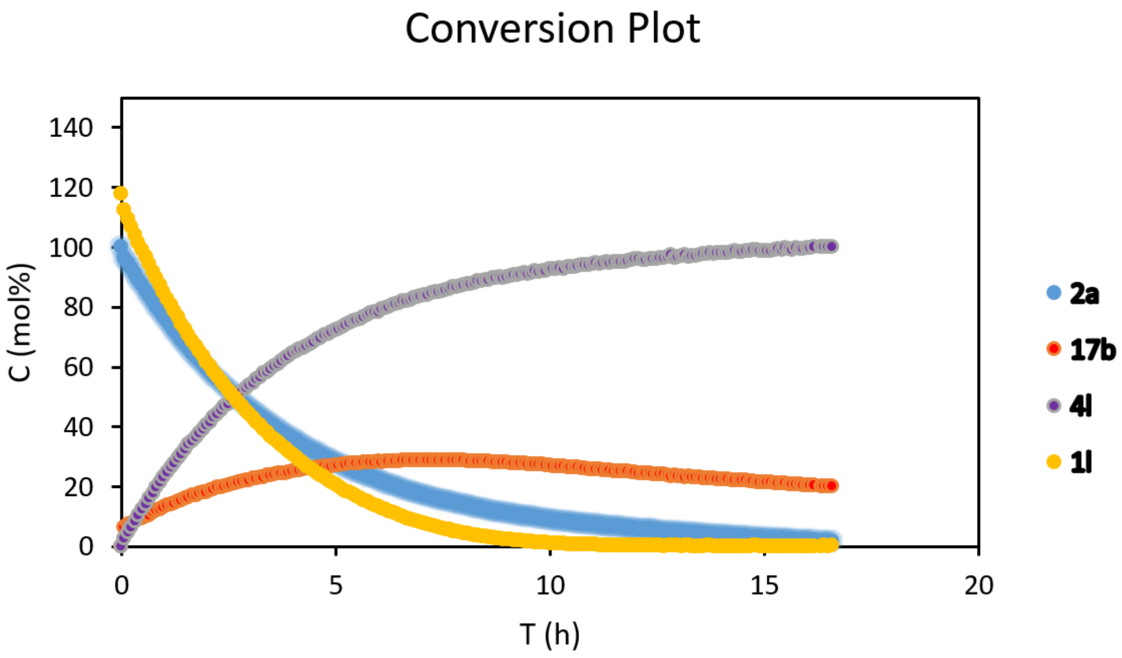

Figure S5. Reaction progress kinetic analysis. Reaction was performed with **1l** (0.075 mmol), **2a** (0.05 mmol), and IDPi **3a** (1 mol%) in toluene-d_8 (0.5 mL, 0.1 M) at −20 °C for ~16.5 h.

From the kinetic plots, the silane starting material **1l** and 2,6-dimethylphenol **2a** were consumed with the concomitant formation of product **4l**, while the formation of the six-membered silane product **17b** presented as a parabola. We speculated that the cyclic silane could be the intermediate of the reaction.

Next, several control experiments were performed to elucidate the possible reaction mechanism.

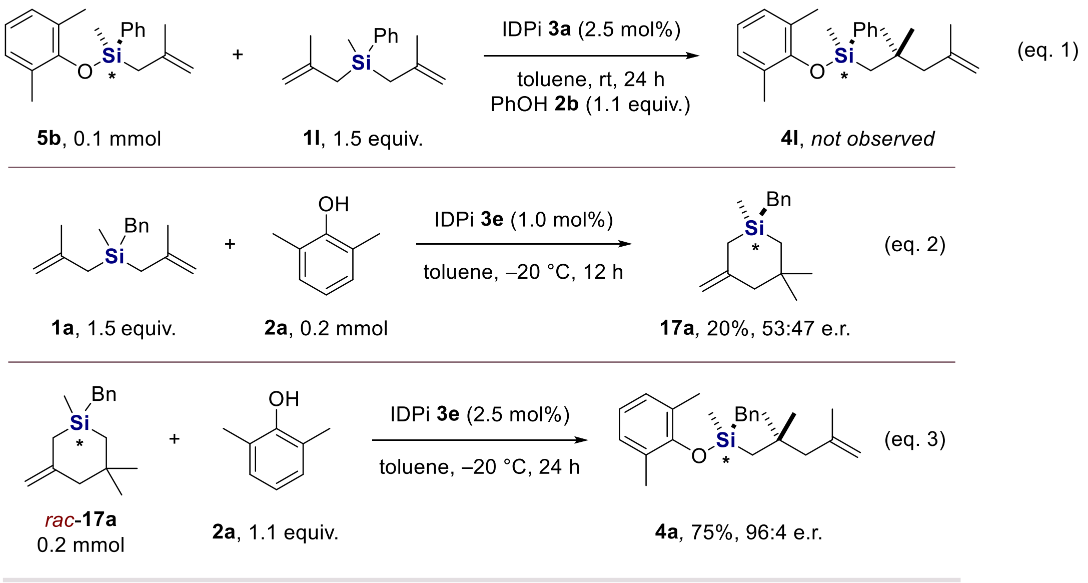

Figure S6. Control experiments. Reaction of eq. 1 was performed with **5b** (0.1 mmol), silane **1l** (1.5 equiv.), phenol **2b** (1.1 equiv.) and IDPi **3a** (2.5 mol%) in toluene (1.0 mL, 0.1 M) at rt for 24 h. Reaction of eq. 2 was performed with **2a** (0.2 mmol), silane **1a** (1.5 equiv.), and IDPi **3e** (1.0 mol%) in toluene (2.0 mL, 0.1 M) at −20 °C for 12 h and terminated by the addition of Et_3N. Reaction of eq. 3 was performed with *rac*-**17a** (0.2 mmol), **2a** (1.1 equiv.), and IDPi **3e** (2.5 mol%) in toluene (2.0 mL, 0.1 M) at −20 °C for 24 h.

Procedure for eq.1: The side product **5b** (0.1 mmol) and silane **1l** (1.5 equiv.) were placed in a 1.5 mL GC vial, which was equipped with a teflon-coated magnetic stirring bar. IDPi **3a** (2.5 mol%) and phenol **2b** (1.1 equiv.) in toluene (0.1 M, 1.0 mL) were added, and the resultant solution was stirred at rt for 24 h. From TLC analysis, no desired product **4l** was detected. The reaction route of intermolecular hydroallylation could be excluded.

Procedure for eq.2: The silane starting material **1a** (0.3 mmol, 1.5 equiv.) was placed in a 5 mL GC vial, which was equipped with a teflon-coated magnetic stirring bar. IDPi **3e** (1.0 mol%) and toluene (0.1 M, 2.0 mL) were added, and the resultant solution was stirred at −20 °C for 10 min before 2,6-dimethylphenol **2a** (0.2 mmol, 1.0 equiv.) was slowly added. The reaction mixture was stirred for an additional 12 h at the indicated temperature. Then the reaction mixture was intentionally quenched by triethylamine, from TLC analysis, a new spot that is less polar than the starting silane appeared. After all volatiles were removed in vacuo, the crude residue was purified by column chromatography with silica gel, which was pre-neutralized by triethylamine. As such, the possible intermediate **17a** was captured in 20% yield with 53:47 e.r..

Procedure for eq.3: *rac*-**17a** (0.2 mmol) was placed in a 5 mL GC vial, which was equipped with a teflon-coated magnetic stirring bar. IDPi **3e** (2.5 mol%) and toluene (0.1 M, 2.0 mL) were added, and the resultant solution was stirred at −20 °C for 10 min before 2,6-dimethylphenol **2a** (1.1 equiv.) was slowly added. The reaction mixture was stirred for an additional 24 h at the indicated temperature.

Then the reaction mixture was quenched by triethylamine. After all volatiles were removed in vacuo, the crude residue was purified by column chromatography with silica gel, which was pre-neutralized by triethylamine. The silane product **4a** was obtained in 75% yield with 96:4 e.r..

10. Inclusion and co-crystallization experiments for single crystal X-ray structure determination

Compound **4b** and structure analogs are oily liquids at room temperature. Initial crystallization attempts from various solvents failed. Therefore several different techniques and co-crystallization methods have been tried. A detailed description is given in the following.

In situ crystallization

In situ crystallization has been successfully used in various cases.[15–17] Therefore, the possibility of *in situ* crystal growth of the neat compound in a glass capillary was checked by low temperature DSC. The experiments do not show any significant phase transition. As already indicated by the DSC curves, no crystalline solid could be obtained during cooling on the diffractometer.

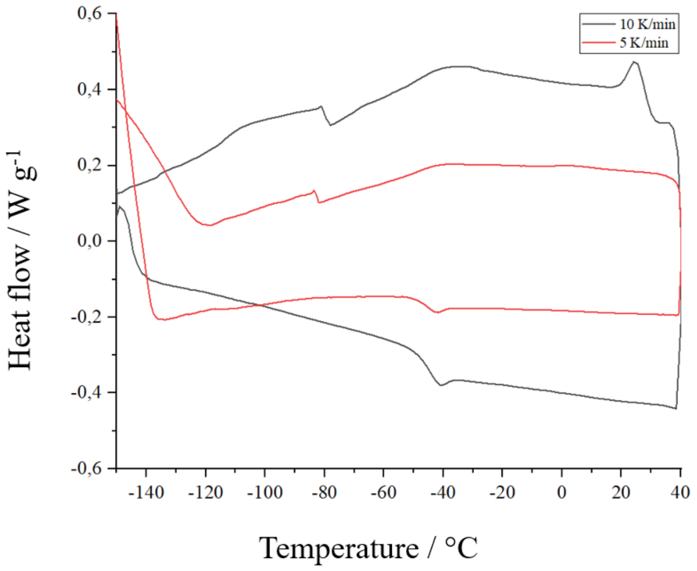

Figure S7. Low temperature DSC curves of **4b** of two cycles with different temperature gradients.

Co-crystallization with chaperone compounds

According to the literature procedure[18], enantiopure compound **4b** was mixed with a sub-stoichiometric amount of different tetraaryladamantanes, the so-called "chaperone" compounds [1,3,5,7-tetrakis(2,4-diethoxyphenyl)adamantane (TEO); 1,3,5,7-tetrakis(2,4-dimethoxyphenyl)adamantane (TDA); 1,3,5,7-tetrakis(2-bromo-4-methoxyphenyl)-adamantane (TBro)].

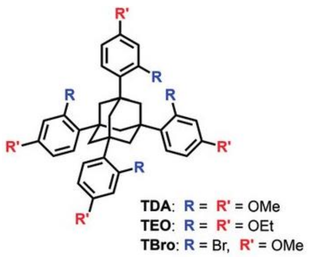

Figure S8: General molecular structure of various chaperone compounds for co-crystallizations.

The mixtures of the liquid target compound and the co-crystallization agents were prepared on a hot stage microscope. Initially the "chaperones" show a poor solubility in the liquid phase at room temperature. During heating, it was observed that the solid completely transformed into a liquid phase at ~160°C. This was continuously checked with crossed polarization filters. During cooling, small single crystals occur at the outer regions of the liquid mixture. These crystals were investigated by XRD. After structure solution and refinement it turned out that only the pure co-former crystallized from the melt. It is expected that the molecular weight of the target compound is too high (~340 g/mol) for this method (should be <250 g/mol according to ref[19]). Therefore no further information could be gained from these experiments regarding the target's structure and absolute configuration.

Crystallization with cyclodextrin compounds

Following literature procedures, enantiopure compound **4b** was treated with β- and γ-cyclodextrin to possibly form an inclusion or co-crystalline compound.

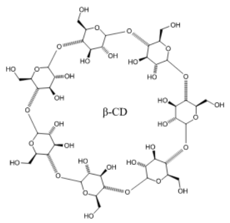

Figure S9: Molecular structure of β-cyclodextrin (β-CD).

Procedure A[20]:

A solution of β-cyclodextrin (33.3 mg, 0.0288 mmol, p.A. grade) and the enantiopure target compound **4b** (10.14 mg, 0.0288 mmol) in Millipore water (2.0 mL) in a 4 mL vial was prepared. After stirring at 70 °C for 4 h, the stirring bar was removed and the mixture gradually cooled to 25 °C over a period of 7 d. After this procedure a crystalline solid could be observed which consisted of several small (edges less than 50 µm) multi-domain crystals which were not suitable for single crystal diffraction analysis.

Procedure B[21]:

A solution of β-cyclodextrin (37.0 mg, 0.032 mmol, p.A. grade) and the enantiopure target compound **4b** (5.64 mg, 0.016 mmol) in Millipore water (0.5 mL) in a 4 mL vial was prepared. After stirring at 60 °C for 24 h, a milky liquid phase was observed. The stirring bar was then removed and the mixture gradually cooled to 25 °C overnight. After this procedure, XRD suitable single crystals could be obtained at the liquid-gas-phase boundary inside the GC vials. The X-ray dataset revealed only a structure of pure β-cyclodextrin hydrate clathrate and no target intercalation or co-crystallization could be observed.

Procedure C:

Three solutions a), b) and c) of β-cyclodextrin (33 mg each, 0.029 mmol, p.A. grade) and the enantiopure target compound **4b** (10 mg each, 0.029 mmol) in Millipore water (a) 1.0 mL; b) 0.75 mL; c) 0.5 mL) in GC vials were prepared. After stirring at 70 °C overnight, the stirring bar was removed and the mixtures gradually cooled to 25 °C over a period of 7 d. After the procedure a crystalline solid could be observed which consisted of several small (edges less than 50 µm) multi-domain crystals which were not suitable for single crystal diffraction analysis.

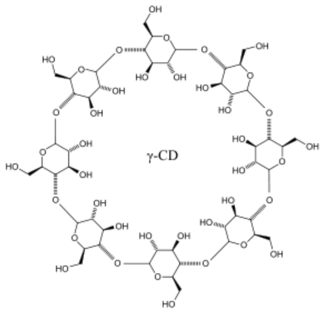

Figure S10: Molecular structure of γ-cyclodextrin (γ-CD).

Procedure D:

Two solutions a) and b) of γ-cyclodextrin (37 mg each, 0.029 mmol, p.A. grade) and the enantiopure target compound **4b** (10 mg each, 0.029 mmol) in Millipore water (a) 1.0 mL; b) 0.5 mL) in GC vials were prepared. After stirring at 70 °C for 4 h, the stirring bar was removed and the mixtures gradually cooled to 25 °C over a period of 3 d. After this procedure, the remaining solid was inspected optically by polarized light microscope. It was found that the solid only consists of an amorphous phase. Therefore no XRD dataset could be recorded.

We used the β- and γ-cyclodextrin because of its bigger inner molecular diameter. All attempts to get a co-crystal or a clathrate containing the target compound failed.

Solvent co-crystallization

Various common and non-standard solvents have been tested in a solvent screening experiment. Here, one droplet of the target compound (purified and enantiopure) was mixed with one droplet of solvent at room temperature in a GC vial with insert. In all tested solvents, no direct formation of a solid phase was observed. The mixtures were transferred into the fridge and stored at 8 °C for 7 d. Even here, no solidification could be observed.

Tested solvents were toluene, mesitylene, benzene, hexafluorobenzene, fluorobenzene, cyclohexane and DMSO.

In some metal-organic compounds[22] tetramethyldisiloxane (TMDS) becomes a suitable co-former. The compound of interest was mixed 1:1 with TMDS at room temperature and no solid was observed. Furthermore, a dilute mixture of target compound and TMDS in toluene was allowed to evaporate over 1 week in a fridge at 4 °C. With this procedure, no suitable single crystals could be observed.

Derivatization

A common method in crystal structure determination is to investigate different derivative forms of the original molecule. Because all initial crystallization attempts failed, two different derivatives were synthesized. Both structures are shown below:

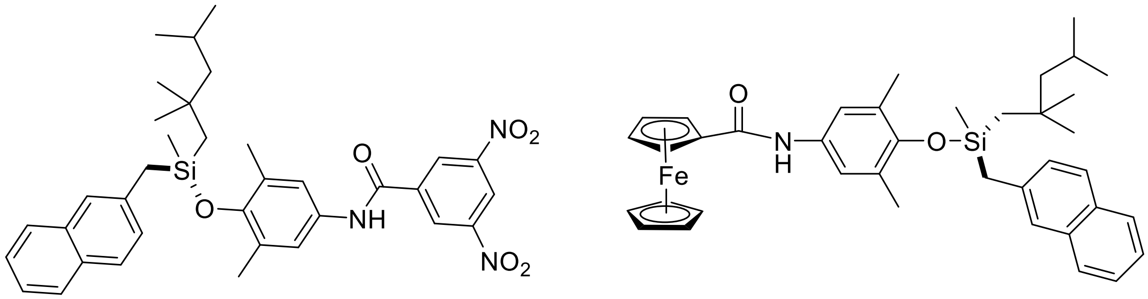

Figure S11: Molecular structures of two derivatives: nitro- (left) and ferrocenyl substituted (right).

The ferrocenyl substitution led to a red compound with a very high viscosity. It was dissolved in toluene in a small glass container at room temperature until a clear solution was obtained. The container was sealed with a pierced polymer lid and placed in the fridge for 1.5 weeks at 4 °C. After this period, an amorphous precipitate was observed.

The yellow NO_2 substituted derivative was handled in a similar way. It appears to be initially in a polycrystalline solid state. It was dissolved in toluene in a small glass container at room temperature until no solid was remained. The container was sealed with a pierced polymer lid and placed in the fridge for 1.5 weeks at 4 °C. After this period, bigger crystals could be observed at the upper inner wall of the glass container. Careful inspection of the crystals by polarized light microscope indicates that some of these crystals may be suitable for XRD.

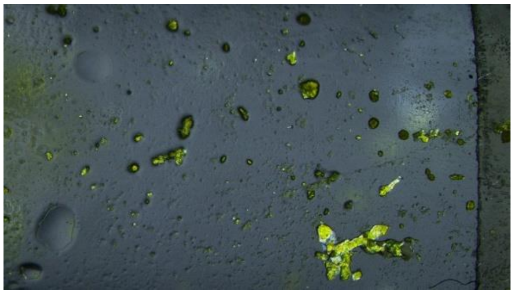

Figure S12: Optical light microscope image (crossed polarization filters) of the recrystallized NO_2 derivative from toluene after 1.5 weeks of solvent evaporation.

Investigation of these crystals on a single crystal diffractometer show only two very poor reflections at low 2Θ angle. No suitable XRD dataset could be recorded.

Crystalline sponge method

All of the tried techniques and co-crystallization methods failed and no further structural support could be gained by these experiments. In order to obtain a X-ray crystal structure of the compound despite the difficulties, the method of Fujita becomes a favorable choice.[23] Here a flexible and porous coordination polymer {$[(ZnX_2)_3\cdot(tpt)_2\cdot x(solvent)]_n$ (X = Cl, I; tpt = 2,4,6-tris(4-pyridyl)-1,3,5-triazine)} is used as “host” for small molecule (“guest”) inclusion. The so-called crystalline sponge (CS) method was successfully applied to several compounds, which are difficult to crystallize, and limited in amount. Additionally, the assignment of the absolute configuration of the major enantiomer of a low enantiomeric excess sample becomes possible.[24]

Sample preparation for CS-XRD of 4b (“guest” inclusion)

Hosting CS was obtained from Merck KGaA Darmstadt, Germany (Crystal-Do project) and used as received. Solvents (dichloromethane and cyclohexane) used have p. A. grade and were stored under inert gas (Ar). Dichloromethane (DCM) was purchased from Fisher Scientific in 99% purity, dried over calcium hydride and stored over molecular sieves. Cyclohexane was obtained from Sigma-Aldrich in >99% purity, dried and distilled before use. The following routine was performed three times with four different types of CS ($[(ZnCl_2)_3\cdot(tpt)_2\cdot x(n\text{-hexane})]_n$; $[(ZnI_2)_3\cdot(tpt)_2\cdot x(n\text{-hexane})]_n$; $[(ZnCl_2)_3\cdot(tpt)_2\cdot x(\text{cyclohexane})]_n$; $[(ZnI_2)_3\cdot(tpt)_2\cdot x(\text{cyclohexane})]_n$). The solvent above each CS was removed, the crystal was subsequently soaked in a mixture of cyclohexane (50 µL) and 1 µL of compound **4b** (1 mg/mL in DCM, 97:3 e.r.) in a glass micro vial. The septum in the screw cap of the micro vial was pierced with a syringe needle (diameter 0.8 mm) and the solvent was slowly evaporated over 20 h at 50 °C in an oven with a gentle Ar flow (0.15 bar, inlet valve 46%, outlet valve 100%). The resulting twelve crystals were subjected to single crystal X-ray analysis using synchrotron radiation. Samples were mounted on MiTeGen-Loops using perfluoropolyether PFO-XR75 and shipped under cryogenic conditions in liquid nitrogen to the synchrotron facility for diffraction experiments. The data set of best quality was used for structure determination. Additional soakings with $[(ZnI_2)_3\cdot(tpt)_2\cdot x(n\text{-hexane})]_n$ show guest inclusions as well but probably due to loss of solvent during mounting delivered diffraction data of minor quality.

Single crystal structure analysis of 4b $(ZnI_2)_3(tpt)_2$ cyclohexane solvate

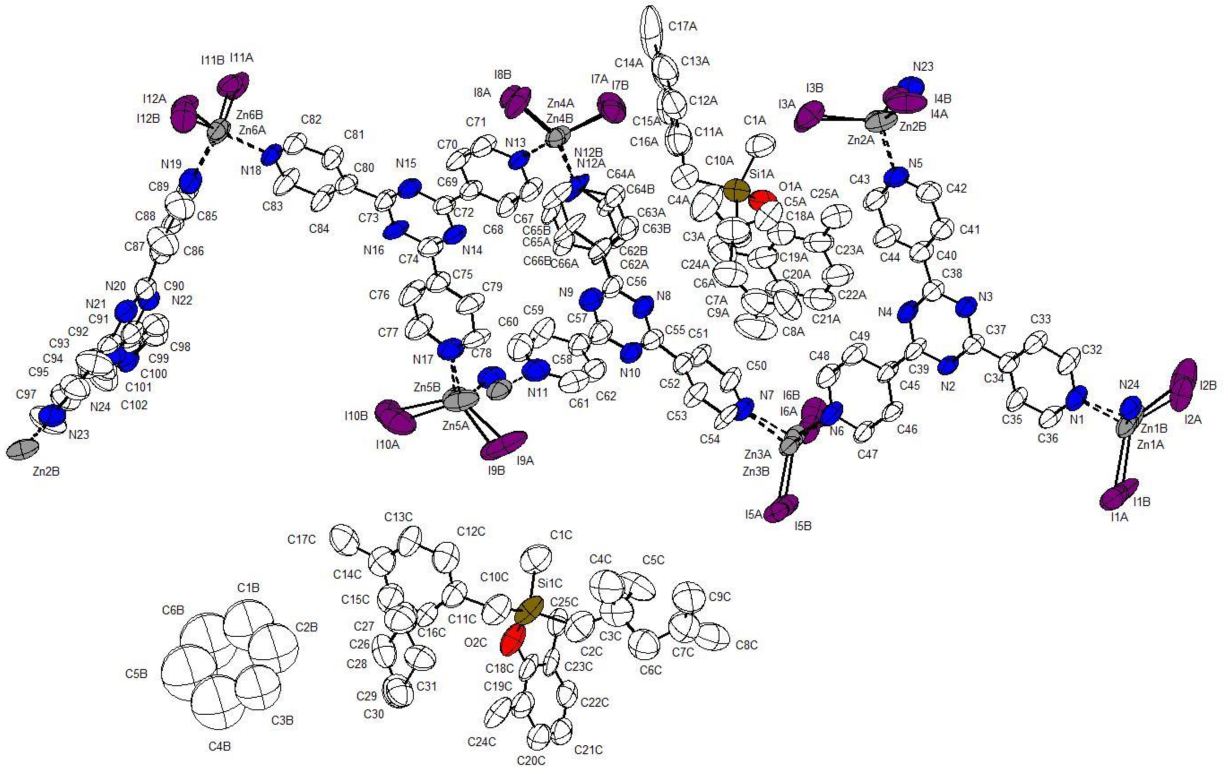

Figure S13. The molecular structure of complex **4b $(ZnI_2)_3(tpt)_2$ cyclohexane solvate**. H atoms have been omitted for clarity. Color code: Zn (grey), I (purple), N (blue), C (white), O (red) and Si (brown-green).

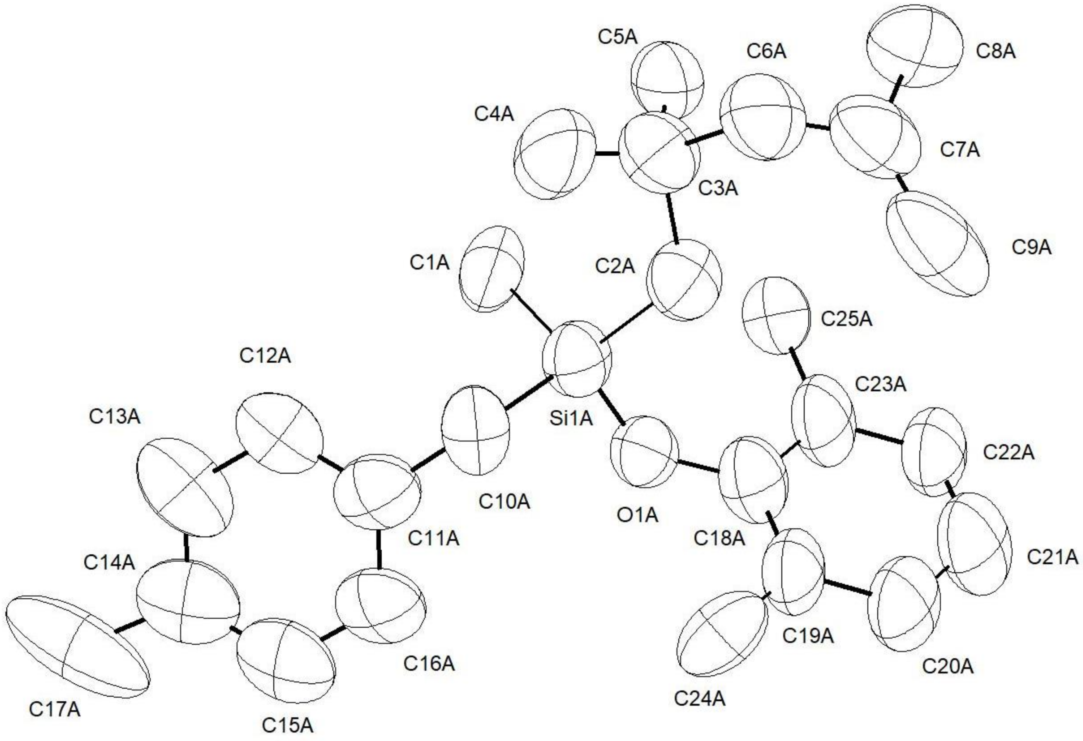

Figure S14. The molecular structure of **4b**. H atoms, MOF, solute and second analyte have been omitted for clarity.

X-ray Crystal Structure Analysis of 4b $(ZnI_2)_3(TPT)_2$ cyclohexane solvate: $C_{93.04}\,H_{80.98}\,I_{12}\,N_{24}\,O_{0.65}\,Si_{0.65}\,Zn_6$, M_r = 3478.87 g mol^{-1}, colourless block, crystal size 0.15 x 0.075 x 0.06 mm^3, monoclinic, *C*2 [5], *a* = 35.072(18) Å, *b* = 14.875(5) Å, *c* = 31.257(13) Å, β = 102.044(9)°, *V* = 15948(12) Å^3, *T* = 100(2) K, *Z* = 4, D_{calc} = 1.449 g·cm^3, λ = 0.56360 Å, $\mu(\lambda)$ = 1.699 mm^{-1}, no absorption correction, P11 beamline at PETRAIII (DESY, Hamburg) synchrotron facility equipped with a single φ-axis goniometer and Pilatus 6M detector, 0.941 < θ < 22.592°, 141505 measured reflections, 39770 independent reflections, 33284 reflections with $I > 2\sigma(I)$, R_{int} = 0.0187. The structure was solved by *SHELXT* and refined by full-matrix least-squares (*SHELXL*) against F^2 to R_1 = 0.0590 [$I > 2\sigma(I)$], wR_2 = 0.1948, 1732 parameters, 683 restraints, absolute structure factor Parsons (z) = 0.101(7).

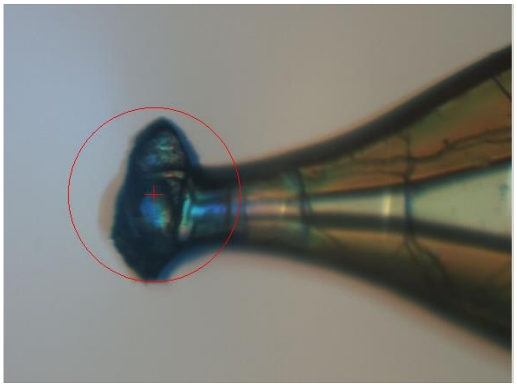
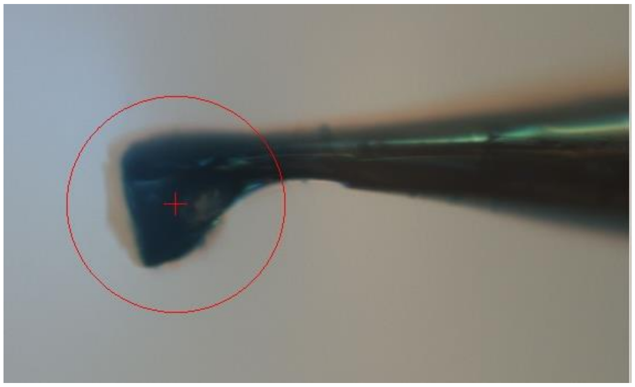

Figure S15. Images of the mounted crystal on a 75 µm MiTeGen loop at 0° and 90° φ-angle. Red circle (size) and cross (center) indicate the profile of the 200 µm primary beam.

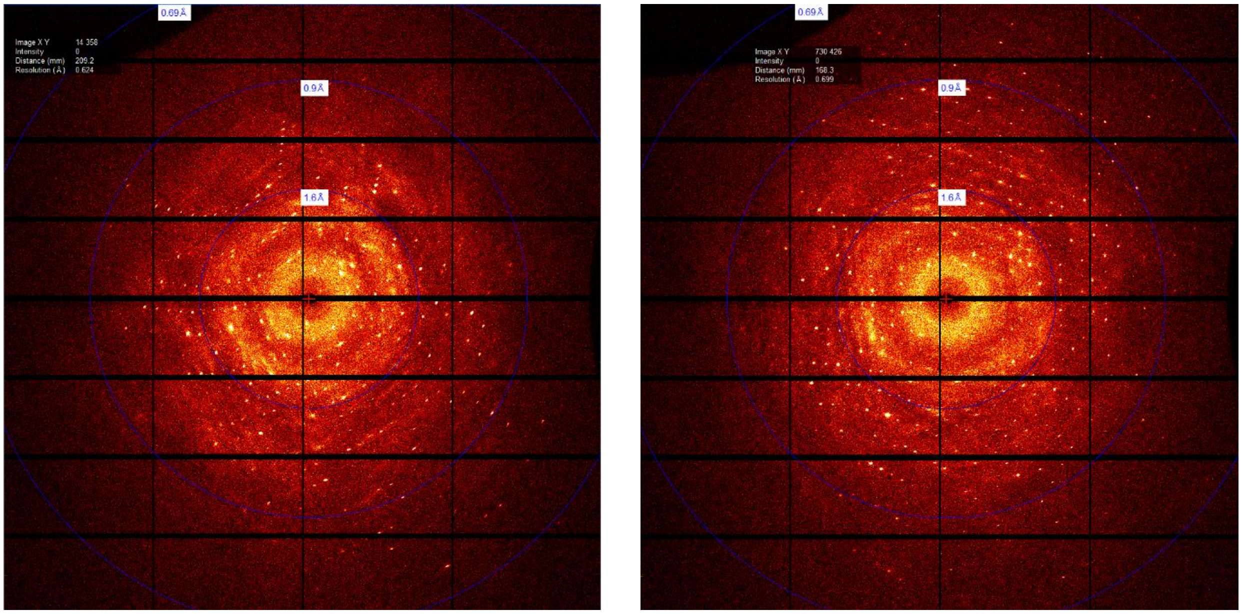

Figure S16. Diffraction pattern of investigated crystal at φ = 0° (left) and φ = 90° (right).

Reciprocal space was explored using spot2pdb routine implemented in XDS software package and Mercury program was used for visualization.[25–26]

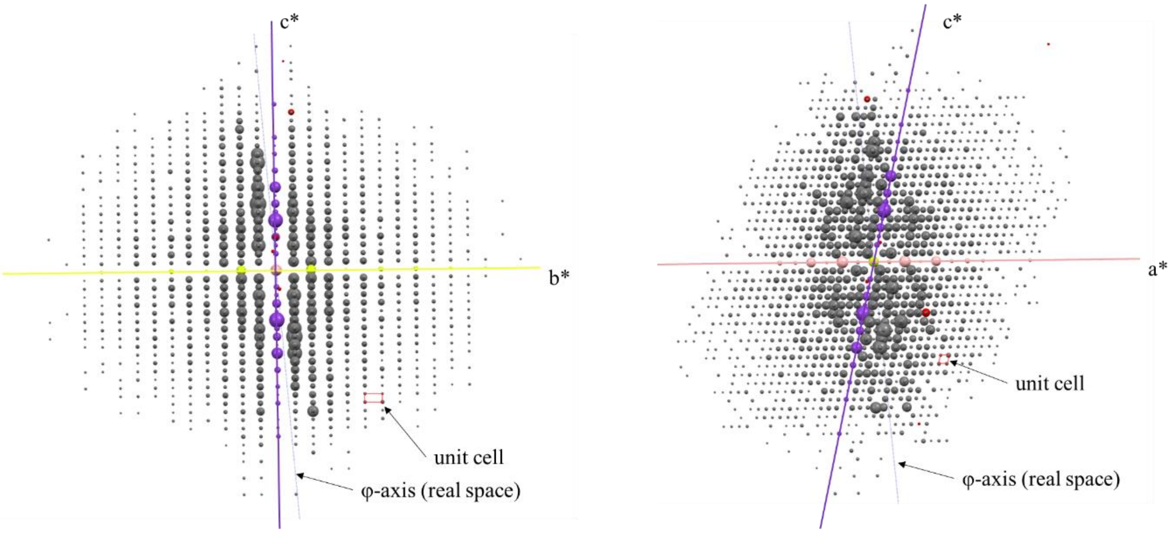

Figure S17. Reciprocal space view along a* (left) and b* (right). Spotsize is related to reflection intensity; unit cell (red box) and axis of rotation (blue line) in the real space are shown. Red spots are reflections, which do not fit to the chosen lattice.

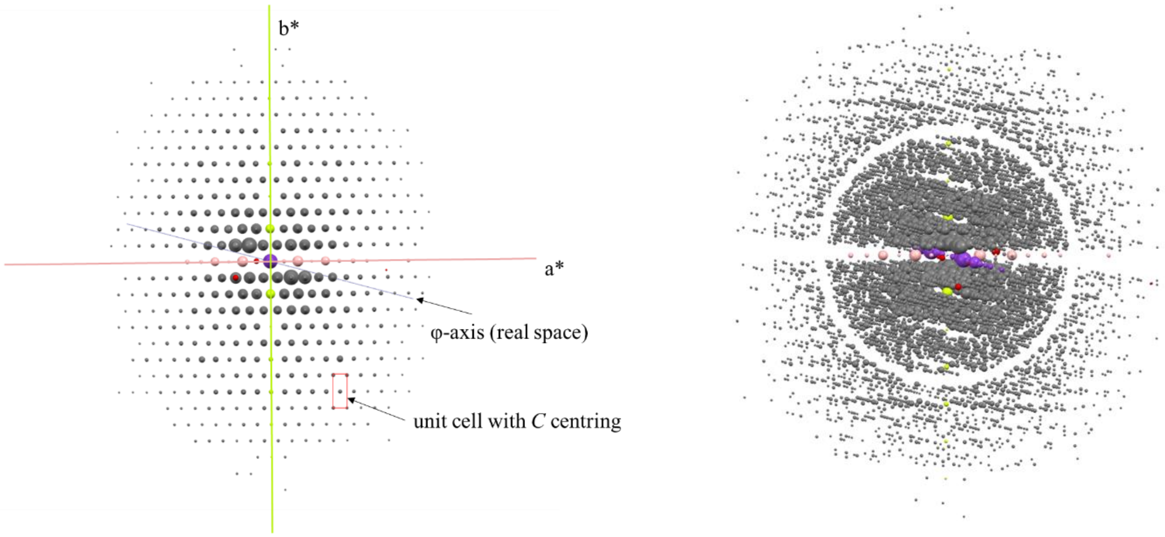

Figure S18. Reciprocal space view along c* (left) and axis of rotation (right). Spotsize is related to reflection intensity; unit cell (red box) and axis of rotation (blue line) in the real space are shown. Red spots are reflections, which do not fit to the chosen lattice.

INTENSITY STATISTICS FOR DATASET

Resolution	#Data	#Theory	%Complete	Redundancy	Mean I	Mean I/s	Rmerge	Rsigma
Inf - 3.11	313	320	97.8	6.22	91.96	92.82	0.0160	0.0104
3.11 - 2.03	737	787	93.6	6.14	41.50	91.63	0.0157	0.0102
2.03 - 1.60	1055	1095	96.3	6.20	23.56	84.88	0.0178	0.0104
1.60 - 1.40	1036	1063	97.5	6.63	11.60	78.39	0.0194	0.0106
1.40 - 1.27	1041	1071	97.2	6.67	7.19	72.00	0.0208	0.0106
1.27 - 1.18	1021	1053	97.0	6.68	5.04	65.05	0.0239	0.0113
1.18 - 1.10	1125	1237	90.9	5.72	3.80	54.25	0.0266	0.0133
1.10 - 1.04	1153	1197	96.3	6.34	2.59	47.24	0.0286	0.0143
1.04 - 1.00	908	946	96.0	6.51	1.84	41.39	0.0299	0.0153
1.00 - 0.96	1075	1129	95.2	6.66	1.39	37.52	0.0343	0.0171
0.96 - 0.93	936	969	96.6	6.83	1.05	31.18	0.0389	0.0196
0.93 - 0.90	1065	1115	95.5	6.76	0.93	28.94	0.0408	0.0212
0.90 - 0.87	1201	1250	96.1	6.88	0.67	23.26	0.0482	0.0259
0.87 - 0.85	866	971	89.2	5.81	0.53	19.60	0.0598	0.0339
0.85 - 0.82	1482	1580	93.8	6.21	0.37	15.12	0.0745	0.0434
0.82 - 0.80	1139	1201	94.8	6.61	0.34	14.82	0.0765	0.0449
0.80 - 0.79	575	619	92.9	6.44	0.22	11.09	0.1027	0.0641
0.79 - 0.77	1340	1420	94.4	6.67	0.20	10.49	0.1121	0.0706
0.77 - 0.76	687	728	94.4	6.65	0.19	9.66	0.1138	0.0743
0.76 - 0.74	1531	1629	94.0	6.56	0.15	8.33	0.1371	0.0914
0.74 - 0.73	509	559	91.1	5.87	0.12	6.35	0.1667	0.1277
0.83 - 0.73	6323	6729	94.0	6.51	0.22	10.75	0.1037	0.0657
Inf - 0.73	20795	21939	94.8	6.45	5.98	37.96	0.0203	0.0118

Complete .cif-data of the compound are available under the CCDC number **CCDC-2115183**.

Seventeen reflections show high I/σI and where excluded from dataset before final refinement cycles. The initially used CS crystallizes in the centrosymmetric space group *C*2/c. By soaking of an enantiopure chiral molecule into the CS the space group then changes to a non-centrosymmetric space group (e.g. *C*2 or $P2_1$). As the initial compound **4b** is enantiopure, the observed space group of the CS within inclusion is non-centrosymmetric (*C*2). Lattice constants are in agreement with other literature examples.[24]

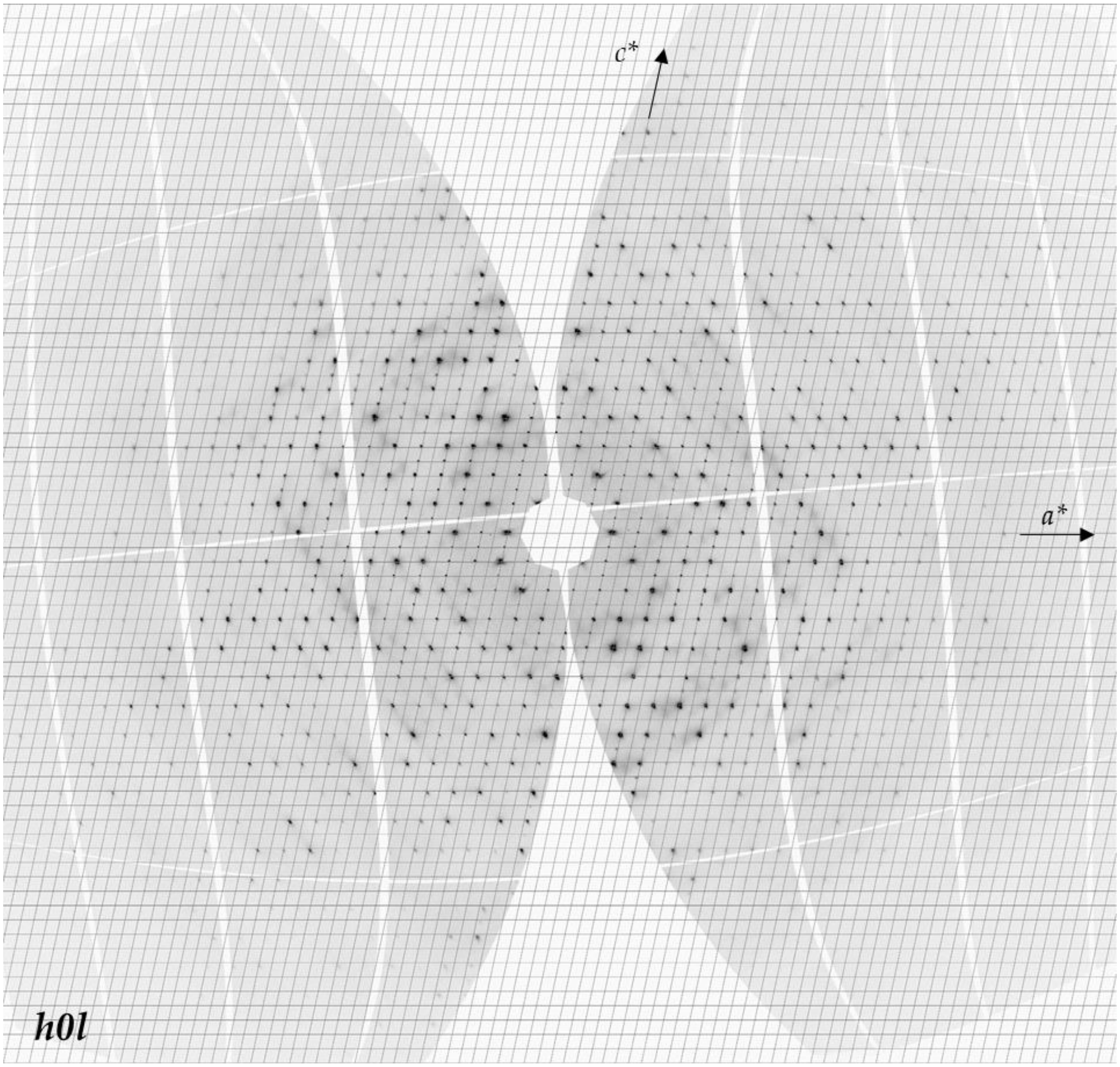

Figure S19. Reciprocal lattice layer (*h*0*l*). Bragg reflections (black spots) and reciprocal lattice grid (horizontal axis *a** and vertical axis *c**) are shown.

Reciprocal *h*0*l*-layer was inspected using *CrysAlisPro* unwarp routine to prove the correct space group assignment.[27] Additional weak *l*=odd reflections along axis *c** are present. This proves the absence of the *c*-glide plane (reflection condition is *h*0*l*: *l*=2n) and thus the indication of the correct space group assignment (*C*2).

Hydrogen atoms are refined using riding model. The inclusion compound consist of a CS backbone $[(ZnI_2)_3{\cdot}(tpt)_2]_n$, two independent molecules of **4b** (called A and C with occupancies of ~0.44 and ~0.4) and two independent solute molecules (cyclohexane, with occupancy of ~0.55 and ~0.25). It was decided to use a model without solvent mask, to show all refinable details of the complex

structure. Not applying a solvent mask has a positive effect on the absolute structure factor which refines closer to zero. Caused by the missing solvent mask, the given model contains solvent accessible voids of 2472.54 Å³ (15.5% of unit cell volume at 1.2 Å probe radius). These voids belong to positions where the disordered solute molecules could not be refined in a proper way.

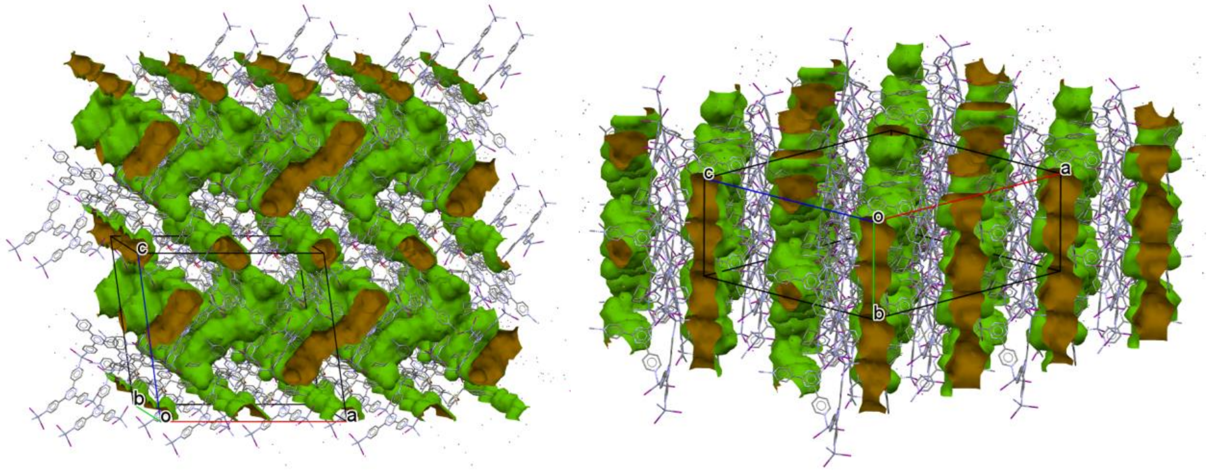

Figure S20. Packing of **4b $(ZnI_2)_3(tpt)_2$ cyclohexane solvate** and the unit cell in two random orientations. Calculated solvent accessible voids (green) are shown respectively.

In addition to the solvent, there is also disorder in the framework structure. The connectors where treated by splitting initial Zn and I atom positions into two parts. One pyridine ring (C62 – C66 and N12) of the framework shows disorder over two positions with occupancy of ~0.6:0.4. Thermal ellipsoids are refined using SIMU instruction.

The first molecule (called A, occupancy ~0.44, PART 1) of **4b** could be identified after full refinement of the framework by residual electron density map. For geometrical optimization some parameters are treated with constraints and restraints. The anisotropic displacement parameters have been equalized by SIMU instruction for all atoms. Aromatic ring systems are constrained with AFIX66. Terminal olefinic bond between C7A and C8A is restrained by DFIX.

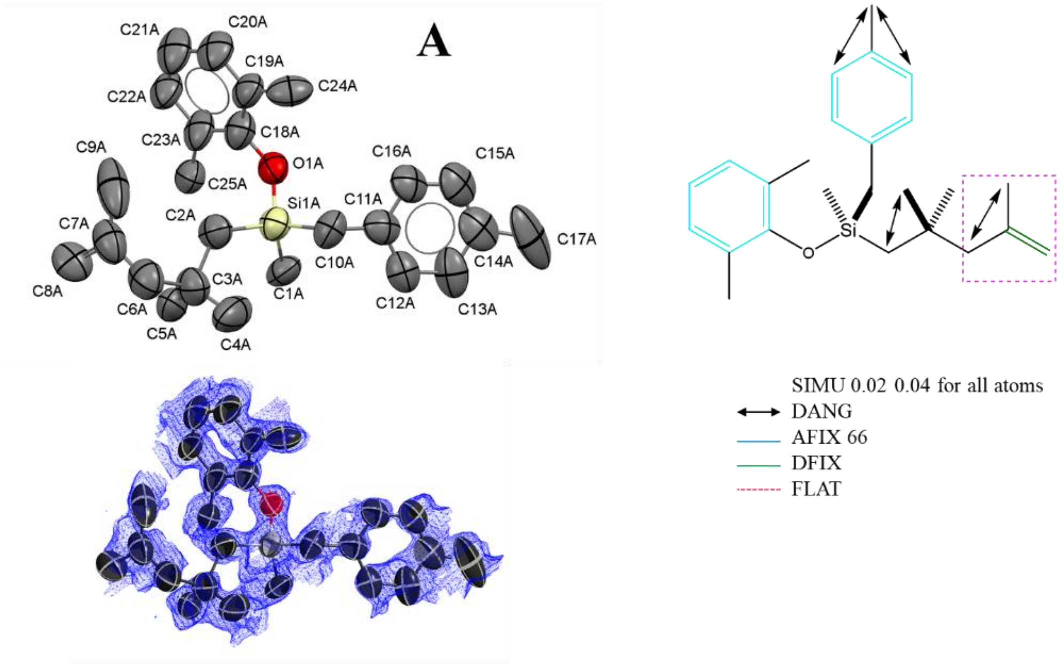

Figure S21. Restraints and constraints applied in the refinement of first independent molecule (A) of compound **4b** and electron density map F_o (contoured at the 0.63σ level).

In addition, the second molecule (called C, occupancy ~0.4, PART -1) of **4b** in the asymmetric unit is located and disordered on a crystallographic special position (twofold rotational axis). Therefore, an intense crystallographic treatment with several constraints and restraints becomes necessary.

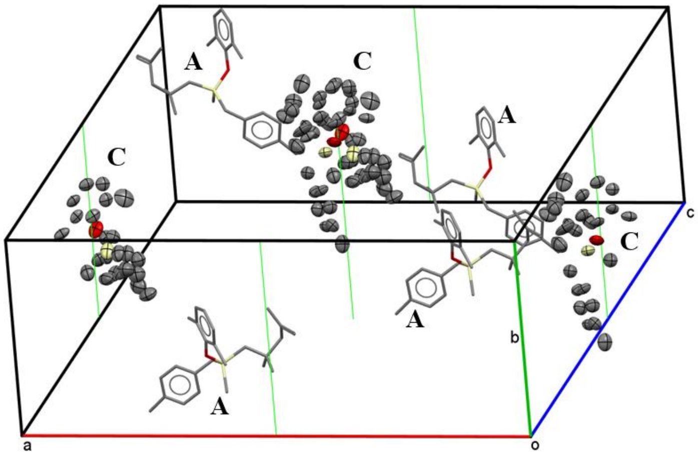

Figure S22. Packing of **4b** in the unit cell. MOF, solute molecules and hydrogen atoms are omitted for clarity. Crystallographic twofold axis are shown in green.

The anisotropic displacement parameters have been equalized by SIMU instruction for all atoms. Aromatic ring systems are constrained with AFIX66. Terminal olefinic bond between C7C and C8C is restrained by DFIX. SAME instruction was used to modulate the sphere around Si central atom in molecule C. Because of high disorder, several other atoms are restrained in their theoretical positions using DANG, AFIX, DFIX and FLAT instructions. This allows the convergence of the refinement and a meaningful connectivity.

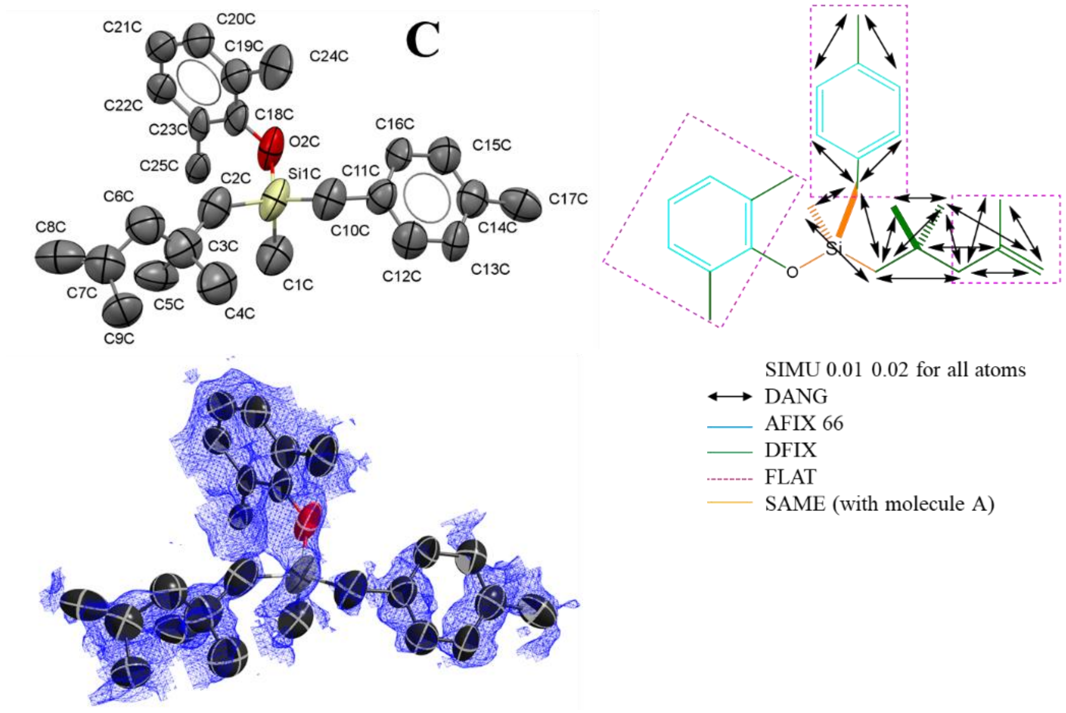

Figure S23. Restraints and constraints applied in the refinement of second independent molecule (C) of compound **4b** and electron density map F_o (contoured at the 0.63σ level).

Within the pores of the CS backbone, two cyclohexane molecules could be found coexisting with the two independent molecules of **4b**.

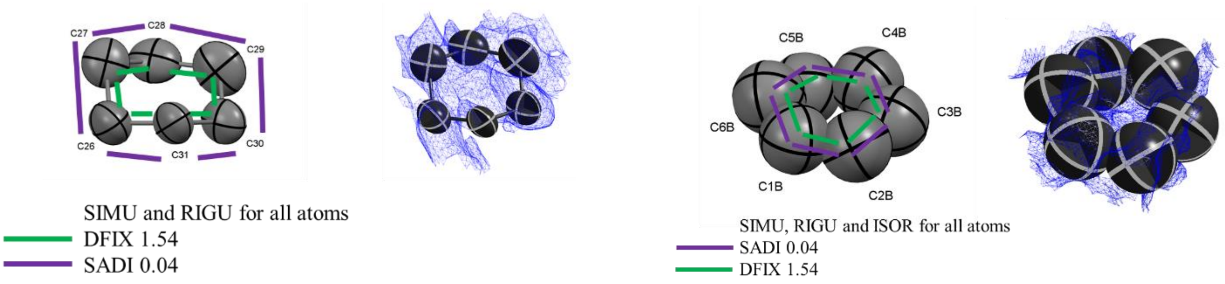

Figure S24. Treatment of both cyclohexane molecules and the corresponding electron density map F_o (contoured at the 0.63σ level), hydrogen atoms are omitted for clarity.

Furthermore, short contacts between one molecule of **4b** and a triazine ring of the CS framework (centroid distance of 3.386 Å) could be found. This π-π-stacking indicates a gentle host-guest interaction of CS and **4b**.

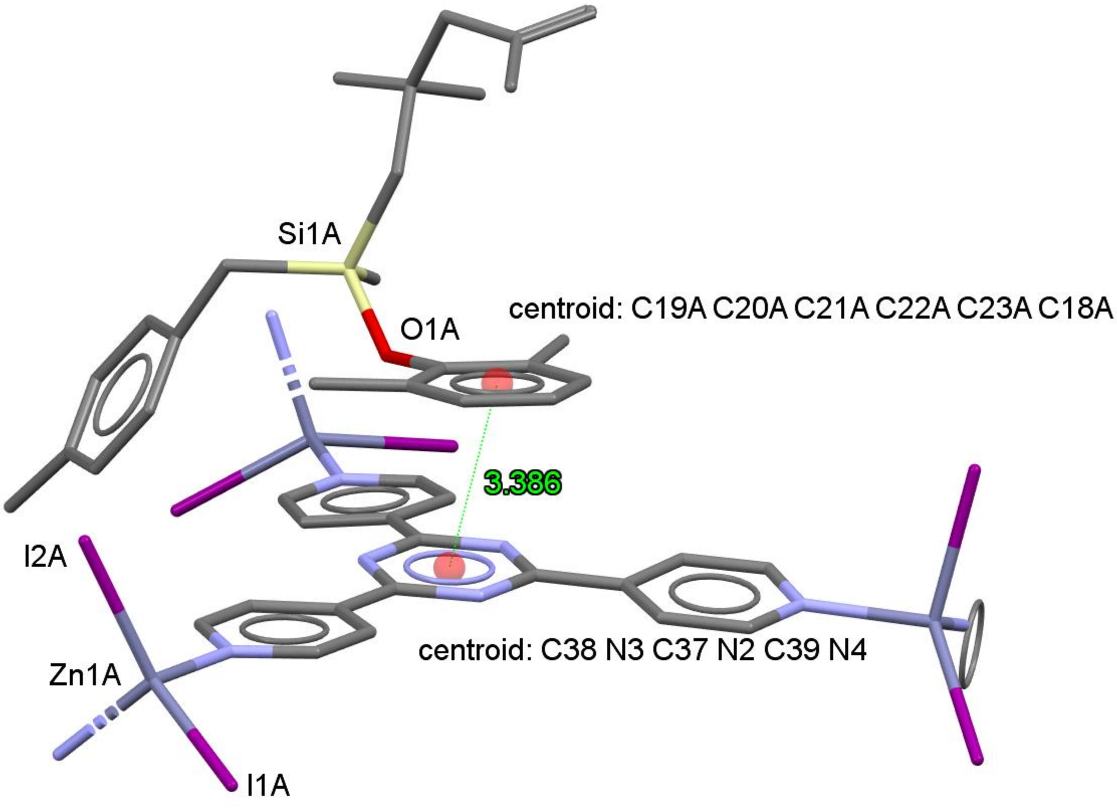

Figure S25. Short contacts between host CS framework and guest **4b**.

Table S4. Crystal data and structure refinement.

Identification code	13855c

Empirical formula	$C_{93.04} H_{80.98} I_{12} N_{24} O_{0.65} Si_{0.65} Zn_6$	
Color	colourless	
Formula weight	3478.87 g·mol^{-1}	
Temperature	100(2) K	
Wavelength	0.563600 Å	
Crystal system	Monoclinic	
Space group	*C*2, (No. 5)	
Unit cell dimensions	a = 35.072(18) Å	α= 90°.
	b = 14.875(5) Å	β= 102.044(9)°.
	c = 31.257(13) Å	γ = 90°.
Volume	15948(12) Å^3	
Z	4	
Density (calculated)	1.449 Mg·m^{-3}	
Absorption coefficient	1.699 mm^{-1}	
F(000)	6550 e	
Crystal size	0.15 x 0.075 x 0.06 mm^3	
θ range for data collection	0.941 to 22.592°.	
Index ranges	$-47 \le h \le 47$, $-20\le k \le 20$, $-39 \le l \le 40$	
Reflections collected	141505	
Independent reflections	39770 [R_{int} = 0.0187]	
Reflections with I>2σ(I)	33284	
Completeness to θ = 19.765°	95.1 %	
Absorption correction	None	
Refinement method	Full-matrix least-squares on F^2	
Data / restraints / parameters	39770 / 683 / 1732	
Goodness-of-fit on F^2	1.046	
Final R indices [I>2σ(I)]	R_1 = 0.0590	wR^2 = 0.1948
R indices (all data)	R_1 = 0.0646	wR^2 = 0.2045
Absolute structure parameter	0.101(7)	
Extinction coefficient	0.00091(7)	
Largest diff. peak and hole	1.275 and -0.566 e·Å^{-3}	

Table S5. Bond lengths [Å] and angles [°].

I(1A)-Zn(1A)	2.547(7)	I(8A)-Zn(4A)	2.605(8)
Zn(4A)-I(7A)	2.541(7)	Zn(4A)-N(12A)	2.051(13)
Zn(4A)-N(13)	2.055(10)	Zn(1A)-I(2A)	2.524(6)
Zn(1A)-N(1)	2.054(9)	Zn(1A)-N(24)#1	2.065(9)
I(5A)-Zn(3A)	2.521(4)	I(11A)-Zn(6A)	2.77(2)
Zn(3A)-I(6A)	2.527(4)	Zn(3A)-N(7)	2.064(9)
Zn(3A)-N(6)	2.081(8)	Zn(6A)-I(12A)	2.490(18)
Zn(6A)-N(18)	1.993(14)	Zn(6A)-N(19)	2.030(16)
I(9A)-Zn(5A)	2.561(8)	I(4A)-Zn(2A)	2.494(11)
I(10B)-Zn(5B)	2.535(7)	I(3A)-Zn(2A)	2.551(8)
Zn(2A)-N(5)	2.107(11)	Zn(2A)-N(23)#2	2.023(13)
Zn(5A)-N(17)	2.117(10)	Zn(5A)-N(11)#3	2.081(11)
Zn(5A)-I(10A)	2.46(2)	N(2)-C(39)	1.347(2)
N(2)-C(37)	1.349(2)	C(39)-N(4)	1.346(2)
C(39)-C(45)	1.436(7)	N(4)-C(38)	1.347(2)
C(38)-N(3)	1.347(2)	C(38)-C(40)	1.432(9)
N(3)-C(37)	1.348(2)	C(37)-C(34)	1.438(7)
N(14)-C(74)	1.338(3)	N(14)-C(72)	1.334(3)
C(74)-N(16)	1.335(3)	C(74)-C(75)	1.449(8)
N(16)-C(73)	1.336(3)	C(73)-N(15)	1.335(3)
C(73)-C(80)	1.455(8)	N(15)-C(72)	1.335(3)
C(72)-C(69)	1.445(8)	C(46)-H(46)	0.9500
C(46)-C(45)	1.397(10)	C(46)-C(47)	1.380(10)
C(81)-H(81)	0.9500	C(81)-C(80)	1.343(11)
C(81)-C(82)	1.390(10)	N(8)-C(55)	1.329(3)
N(8)-C(56)	1.329(3)	C(55)-N(10)	1.329(3)
C(55)-C(52)	1.464(10)	N(10)-C(57)	1.327(3)
C(57)-N(9)	1.327(3)	C(57)-C(58)	1.430(11)
N(9)-C(56)	1.328(3)	C(56)-C(62A)	1.521(12)
C(56)-C(62B)	1.486(13)	C(80)-C(84)	1.382(11)
N(17)-C(78)	1.326(12)	N(17)-C(77)	1.369(13)
N(17)-Zn(5B)	2.063(10)	C(45)-C(49)	1.395(10)
C(62A)-C(66A)	1.359(7)	C(62A)-C(63A)	1.360(7)
C(66A)-H(66A)	0.9500	C(66A)-C(65A)	1.363(7)
C(65A)-H(65A)	0.9500	C(65A)-N(12A)	1.358(7)
N(12A)-C(64A)	1.360(7)	C(64A)-H(64A)	0.9500
C(64A)-C(63A)	1.358(7)	C(63A)-H(63A)	0.9500

C(78)-H(78)	0.9500	C(78)-C(79)	1.417(11)
N(5)-C(42)	1.334(13)	N(5)-C(43)	1.284(12)
N(5)-Zn(2B)	2.034(11)	C(98)-C(92)	1.406(9)
C(98)-C(99)	1.340(15)	C(98)-C(102)	1.441(14)
C(90)-N(22)	1.344(3)	C(90)-N(20)	1.338(3)
C(90)-C(87)	1.439(9)	N(22)-C(92)	1.340(3)
C(92)-N(21)	1.341(3)	N(21)-C(91)	1.341(3)
C(91)-N(20)	1.342(3)	C(91)-C(93)	1.431(12)
N(7)-C(50)	1.293(14)	N(7)-C(54)	1.381(19)
N(7)-Zn(3B)	2.170(10)	C(83)-H(83)	0.9500
C(83)-N(18)	1.353(11)	C(83)-C(84)	1.371(11)
C(70)-H(70)	0.9500	C(70)-C(71)	1.381(11)
C(70)-C(69)	1.409(11)	C(48)-H(48)	0.9500
C(48)-C(49)	1.364(11)	C(48)-N(6)	1.326(13)
C(87)-C(88)	1.398(13)	C(87)-C(86)	1.369(13)
C(33)-H(33)	0.9500	C(33)-C(34)	1.390(9)
C(33)-C(32)	1.378(10)	C(71)-H(71)	0.9500
C(71)-N(13)	1.323(10)	N(18)-C(82)	1.322(9)
N(18)-Zn(6B)	2.092(6)	C(34)-C(35)	1.337(11)
C(35)-H(35)	0.9500	C(35)-C(36)	1.383(9)
C(88)-H(88)	0.9500	C(88)-C(89)	1.337(12)
C(68)-H(68)	0.9500	C(68)-C(69)	1.366(10)
C(68)-C(67)	1.384(10)	C(93)-C(97)	1.376(14)
C(93)-C(94)	1.369(14)	N(13)-C(67)	1.362(10)
N(13)-Zn(4B)	2.052(7)	C(42)-H(42)	0.9500
C(42)-C(41)	1.371(12)	C(84)-H(84)	0.9500
N(1)-C(36)	1.348(8)	N(1)-C(32)	1.318(12)
N(1)-Zn(1B)	2.087(14)	C(36)-H(36)	0.9500
C(41)-H(41)	0.9500	C(41)-C(40)	1.397(10)
C(79)-H(79)	0.9500	C(79)-C(75)	1.335(12)
C(49)-H(49)	0.9500	C(44)-H(44)	0.9500
C(44)-C(40)	1.389(12)	C(44)-C(43)	1.391(13)
C(76)-H(76)	0.9500	C(76)-C(75)	1.390(12)
C(76)-C(77)	1.377(12)	N(6)-C(47)	1.361(9)
N(6)-Zn(3B)	2.092(8)	C(47)-H(47)	0.9500
C(32)-H(32)	0.9500	C(82)-H(82)	0.9500
C(97)-H(97)	0.9500	C(97)-C(96)	1.370(18)
C(67)-H(67)	0.9500	C(94)-H(94)	0.9500
C(94)-C(95)	1.418(18)	C(85)-H(85)	0.9500

C(85)-C(86)	1.418(17)	C(85)-N(19)	1.254(16)
C(89)-H(89)	0.9500	C(89)-N(19)	1.374(12)
C(77)-H(77)	0.9500	C(43)-H(43)	0.9500
C(96)-H(96)	0.9500	C(96)-N(23)	1.362(15)
C(95)-H(95)	0.9500	C(95)-N(23)	1.300(13)
C(53)-H(53)	0.9500	C(53)-C(52)	1.393(14)
C(53)-C(54)	1.406(14)	C(50)-H(50)	0.9500
C(50)-C(51)	1.433(11)	C(86)-H(86)	0.9500
C(62)-H(62)	0.9500	C(62)-C(58)	1.376(13)
C(62)-C(61)	1.316(16)	C(52)-C(51)	1.331(15)
N(19)-Zn(6B)	2.105(9)	C(54)-H(54)	0.9500
C(58)-C(59)	1.379(14)	N(23)-Zn(2B)#4	2.077(14)
N(11)-C(61)	1.384(15)	N(11)-C(60)	1.284(15)
N(11)-Zn(5B)#5	2.067(13)	C(51)-H(51)	0.9500
C(61)-H(61)	0.9500	C(59)-H(59)	0.9500
C(59)-C(60)	1.419(18)	C(60)-H(60)	0.9500
C(100)-H(100)	0.9500	C(100)-C(99)	1.363(13)
C(100)-N(24)	1.357(11)	C(99)-H(99)	0.9500
C(102)-H(102)	0.9500	C(102)-C(101)	1.360(19)
I(8B)-Zn(4B)	2.525(5)	Zn(4B)-I(7B)	2.505(6)
Zn(4B)-N(12B)	2.101(14)	I(2B)-Zn(1B)	2.521(17)
Zn(1B)-I(1B)	2.545(16)	Zn(1B)-N(24)#1	2.127(16)
I(5B)-Zn(3B)	2.528(5)	Zn(3B)-I(6B)	2.524(5)
Zn(5B)-I(9B)	2.545(9)	I(3B)-Zn(2B)	2.547(9)
Zn(2B)-I(4B)	2.546(10)	N(24)-C(101)	1.311(17)
C(22A)-H(22A)	0.9500	C(22A)-C(23A)	1.3900
C(22A)-C(21A)	1.3900	C(23A)-C(18A)	1.3900
C(23A)-C(25A)	1.54(3)	C(18A)-C(19A)	1.3900
C(18A)-O(1A)	1.364(19)	C(19A)-C(20A)	1.3900
C(19A)-C(24A)	1.50(3)	C(20A)-H(20A)	0.9500
C(20A)-C(21A)	1.3900	C(21A)-H(21A)	0.9500
C(101)-H(101)	0.9500	C(1A)-H(1AA)	0.9800
C(1A)-H(1AB)	0.9800	C(1A)-H(1AC)	0.9800
C(1A)-Si(1A)	1.92(2)	Si(1A)-O(1A)	1.658(16)
Si(1A)-C(2A)	1.890(18)	Si(1A)-C(10A)	1.90(2)
C(2A)-H(2AA)	0.9900	C(2A)-H(2AB)	0.9900
C(2A)-C(3A)	1.62(4)	C(10A)-H(10A)	0.9900
C(10A)-H(10B)	0.9900	C(10A)-C(11A)	1.55(3)
C(24A)-H(24A)	0.9800	C(24A)-H(24B)	0.9800

C(24A)-H(24C)	0.9800	C(25A)-H(25A)	0.9800
C(25A)-H(25B)	0.9800	C(25A)-H(25C)	0.9800
C(17A)-H(17A)	0.9800	C(17A)-H(17B)	0.9800
C(17A)-H(17C)	0.9800	C(17A)-C(14A)	1.54(4)
C(11A)-C(16A)	1.3900	C(11A)-C(12A)	1.3900
C(16A)-H(16A)	0.9500	C(16A)-C(15A)	1.3900
C(15A)-H(15A)	0.9500	C(15A)-C(14A)	1.3900
C(14A)-C(13A)	1.3900	C(13A)-H(13A)	0.9500
C(13A)-C(12A)	1.3900	C(12A)-H(12A)	0.9500
C(3A)-C(6A)	1.62(4)	C(3A)-C(4A)	1.32(5)
C(3A)-C(5A)	1.43(4)	C(6A)-H(6AA)	0.9900
C(6A)-H(6AB)	0.9900	C(6A)-C(7A)	1.46(5)
C(4A)-H(4AA)	0.9800	C(4A)-H(4AB)	0.9800
C(4A)-H(4AC)	0.9800	C(5A)-H(5AA)	0.9800
C(5A)-H(5AB)	0.9800	C(5A)-H(5AC)	0.9800
C(7A)-C(9A)	1.47(5)	C(7A)-C(8A)	1.33(2)
C(9A)-H(9AA)	0.9800	C(9A)-H(9AB)	0.9800
C(9A)-H(9AC)	0.9800	C(8A)-H(8AA)	0.9500
C(8A)-H(8AB)	0.9500	N(12B)-C(64B)	1.371(9)
N(12B)-C(65B)	1.372(9)	C(64B)-H(64B)	0.9500
C(64B)-C(63B)	1.375(9)	C(63B)-H(63B)	0.9500
C(63B)-C(62B)	1.370(9)	C(62B)-C(66B)	1.369(9)
C(66B)-H(66B)	0.9500	C(66B)-C(65B)	1.370(9)
C(65B)-H(65B)	0.9500	I(12B)-Zn(6B)	2.582(5)
Zn(6B)-I(11B)	2.449(6)	C(1C)-H(1CA)	0.9800
C(1C)-H(1CB)	0.9800	C(1C)-H(1CC)	0.9800
C(1C)-Si(1C)	1.97(3)	Si(1C)-O(2C)	1.62(2)
Si(1C)-C(10C)	1.85(2)	Si(1C)-C(2C)	1.91(3)
O(2C)-C(18C)	1.24(3)	C(10C)-H(10C)	0.9900
C(10C)-H(10D)	0.9900	C(10C)-C(11C)	1.55(2)
C(2C)-H(2CA)	0.9900	C(2C)-H(2CB)	0.9900
C(2C)-C(3C)	1.54(3)	C(18C)-C(23C)	1.3900
C(18C)-C(19C)	1.3900	C(23C)-C(22C)	1.3900
C(23C)-C(25C)	1.52(3)	C(22C)-H(22C)	0.9500
C(22C)-C(21C)	1.3900	C(21C)-H(21C)	0.9500
C(21C)-C(20C)	1.3900	C(20C)-H(20C)	0.9500
C(20C)-C(19C)	1.3900	C(19C)-C(24C)	1.53(3)
C(25C)-H(25D)	0.9800	C(25C)-H(25E)	0.9800
C(25C)-H(25F)	0.9800	C(24C)-H(24D)	0.9800

C(24C)-H(24E)	0.9800	C(24C)-H(24F)	0.9800
C(2B)-H(2BA)	0.9900	C(2B)-H(2BB)	0.9900
C(2B)-C(1B)	1.54(3)	C(2B)-C(3B)	1.54(3)
C(1B)-H(1BA)	0.9900	C(1B)-H(1BB)	0.9900
C(1B)-C(6B)	1.57(3)	C(6B)-H(6BA)	0.9900
C(6B)-H(6BB)	0.9900	C(6B)-C(5B)	1.55(3)
C(5B)-H(5BA)	0.9900	C(5B)-H(5BB)	0.9900
C(5B)-C(4B)	1.56(3)	C(4B)-H(4BA)	0.9900
C(4B)-H(4BB)	0.9900	C(4B)-C(3B)	1.54(3)
C(3B)-H(3BA)	0.9900	C(3B)-H(3BB)	0.9900
C(29)-H(29A)	0.9900	C(29)-H(29B)	0.9900
C(29)-C(30)	1.53(3)	C(29)-C(28)	1.54(3)
C(30)-H(30A)	0.9900	C(30)-H(30B)	0.9900
C(30)-C(31)	1.53(2)	C(31)-H(31A)	0.9900
C(31)-H(31B)	0.9900	C(31)-C(26)	1.57(3)
C(26)-H(26A)	0.9900	C(26)-H(26B)	0.9900
C(26)-C(27)	1.57(3)	C(27)-H(27A)	0.9900
C(27)-H(27B)	0.9900	C(27)-C(28)	1.53(3)
C(28)-H(28A)	0.9900	C(28)-H(28B)	0.9900
C(14C)-C(13C)	1.3900	C(14C)-C(15C)	1.3900
C(14C)-C(17C)	1.53(2)	C(13C)-H(13C)	0.9500
C(13C)-C(12C)	1.3900	C(12C)-H(12C)	0.9500
C(12C)-C(11C)	1.3900	C(11C)-C(16C)	1.3900
C(16C)-H(16C)	0.9500	C(16C)-C(15C)	1.3900
C(15C)-H(15C)	0.9500	C(17C)-H(17D)	0.9800
C(17C)-H(17E)	0.9800	C(17C)-H(17F)	0.9800
C(3C)-C(6C)	1.55(3)	C(3C)-C(4C)	1.54(3)
C(3C)-C(5C)	1.54(3)	C(6C)-H(6CA)	0.9900
C(6C)-H(6CB)	0.9900	C(6C)-C(7C)	1.52(3)
C(4C)-H(4CA)	0.9800	C(4C)-H(4CB)	0.9800
C(4C)-H(4CC)	0.9800	C(5C)-H(5CA)	0.9800
C(5C)-H(5CB)	0.9800	C(5C)-H(5CC)	0.9800
C(7C)-C(9C)	1.53(3)	C(7C)-C(8C)	1.33(3)
C(9C)-H(9CA)	0.9800	C(9C)-H(9CB)	0.9800
C(9C)-H(9CC)	0.9800	C(8C)-H(8CA)	0.9500
C(8C)-H(8CB)	0.9500		

I(7A)-Zn(4A)-I(8A)	125.5(3)	N(12A)-Zn(4A)-I(8A)	115.7(5)
N(12A)-Zn(4A)-I(7A)	102.4(5)	N(12A)-Zn(4A)-N(13)	100.6(5)

N(13)-Zn(4A)-I(8A)	104.7(4)	N(13)-Zn(4A)-I(7A)	104.7(3)
I(2A)-Zn(1A)-I(1A)	125.7(3)	N(1)-Zn(1A)-I(1A)	109.0(4)
N(1)-Zn(1A)-I(2A)	106.3(3)	N(1)-Zn(1A)-N(24)#1	101.5(4)
N(24)#1-Zn(1A)-I(1A)	104.1(3)	N(24)#1-Zn(1A)-I(2A)	107.7(3)
I(5A)-Zn(3A)-I(6A)	127.86(19)	N(7)-Zn(3A)-I(5A)	111.1(4)
N(7)-Zn(3A)-I(6A)	102.1(4)	N(7)-Zn(3A)-N(6)	97.8(3)
N(6)-Zn(3A)-I(5A)	114.8(3)	N(6)-Zn(3A)-I(6A)	98.6(3)
I(12A)-Zn(6A)-I(11A)	120.7(6)	N(18)-Zn(6A)-I(11A)	102.7(8)
N(18)-Zn(6A)-I(12A)	112.1(7)	N(18)-Zn(6A)-N(19)	102.0(7)
N(19)-Zn(6A)-I(11A)	110.5(7)	N(19)-Zn(6A)-I(12A)	107.3(8)
I(4A)-Zn(2A)-I(3A)	123.4(4)	N(5)-Zn(2A)-I(4A)	109.1(5)
N(5)-Zn(2A)-I(3A)	105.8(4)	N(23)#2-Zn(2A)-I(4A)	101.7(4)
N(23)#2-Zn(2A)-I(3A)	111.7(5)	N(23)#2-Zn(2A)-N(5)	103.4(5)
N(17)-Zn(5A)-I(9A)	108.6(4)	N(17)-Zn(5A)-I(10A)	106.6(7)
N(11)#3-Zn(5A)-I(9A)	109.4(4)	N(11)#3-Zn(5A)-N(17)	99.2(5)
N(11)#3-Zn(5A)-I(10A)	105.3(5)	I(10A)-Zn(5A)-I(9A)	124.7(7)
C(39)-N(2)-C(37)	120.0	N(2)-C(39)-C(45)	119.9(4)
N(4)-C(39)-N(2)	120.1	N(4)-C(39)-C(45)	120.0(4)
C(39)-N(4)-C(38)	120.0	N(4)-C(38)-C(40)	117.0(4)
N(3)-C(38)-N(4)	120.0	N(3)-C(38)-C(40)	122.7(4)
C(38)-N(3)-C(37)	120.2	N(2)-C(37)-C(34)	117.9(4)
N(3)-C(37)-N(2)	119.8	N(3)-C(37)-C(34)	122.3(4)
C(72)-N(14)-C(74)	120.0	N(14)-C(74)-C(75)	120.9(4)
N(16)-C(74)-N(14)	120.0	N(16)-C(74)-C(75)	118.4(4)
C(74)-N(16)-C(73)	120.0	N(16)-C(73)-C(80)	119.3(4)
N(15)-C(73)-N(16)	120.0	N(15)-C(73)-C(80)	120.6(4)
C(72)-N(15)-C(73)	120.1	N(14)-C(72)-N(15)	119.9
N(14)-C(72)-C(69)	120.8(4)	N(15)-C(72)-C(69)	119.1(4)
C(45)-C(46)-H(46)	120.2	C(47)-C(46)-H(46)	120.2
C(47)-C(46)-C(45)	119.6(7)	C(80)-C(81)-H(81)	120.3
C(80)-C(81)-C(82)	119.4(7)	C(82)-C(81)-H(81)	120.3
C(55)-N(8)-C(56)	120.0	N(8)-C(55)-N(10)	119.9
N(8)-C(55)-C(52)	119.9(5)	N(10)-C(55)-C(52)	119.8(5)
C(57)-N(10)-C(55)	120.0	N(10)-C(57)-C(58)	120.9(5)
N(9)-C(57)-N(10)	120.1	N(9)-C(57)-C(58)	118.9(5)
C(57)-N(9)-C(56)	119.9	N(8)-C(56)-C(62A)	114.8(7)
N(8)-C(56)-C(62B)	124.3(7)	N(9)-C(56)-N(8)	120.1
N(9)-C(56)-C(62A)	124.7(6)	N(9)-C(56)-C(62B)	115.7(7)
C(81)-C(80)-C(73)	122.5(7)	C(81)-C(80)-C(84)	119.3(7)

C(84)-C(80)-C(73)	118.2(7)	C(78)-N(17)-Zn(5A)	119.3(7)
C(78)-N(17)-C(77)	119.2(8)	C(78)-N(17)-Zn(5B)	127.0(7)
C(77)-N(17)-Zn(5A)	121.5(7)	C(77)-N(17)-Zn(5B)	113.9(6)
C(46)-C(45)-C(39)	123.2(6)	C(49)-C(45)-C(39)	119.6(6)
C(49)-C(45)-C(46)	117.1(7)	C(66A)-C(62A)-C(56)	114.0(7)
C(66A)-C(62A)-C(63A)	120.0	C(63A)-C(62A)-C(56)	125.9(7)
C(62A)-C(66A)-H(66A)	120.0	C(62A)-C(66A)-C(65A)	120.0
C(65A)-C(66A)-H(66A)	120.0	C(66A)-C(65A)-H(65A)	120.0
N(12A)-C(65A)-C(66A)	119.9	N(12A)-C(65A)-H(65A)	120.0
C(65A)-N(12A)-Zn(4A)	117.1(7)	C(65A)-N(12A)-C(64A)	120.0
C(64A)-N(12A)-Zn(4A)	122.6(6)	N(12A)-C(64A)-H(64A)	119.9
C(63A)-C(64A)-N(12A)	120.2	C(63A)-C(64A)-H(64A)	119.9
C(62A)-C(63A)-H(63A)	120.0	C(64A)-C(63A)-C(62A)	119.9
C(64A)-C(63A)-H(63A)	120.0	N(17)-C(78)-H(78)	119.7
N(17)-C(78)-C(79)	120.6(9)	C(79)-C(78)-H(78)	119.7
C(42)-N(5)-Zn(2A)	126.2(6)	C(42)-N(5)-Zn(2B)	119.5(7)
C(43)-N(5)-Zn(2A)	117.5(7)	C(43)-N(5)-C(42)	116.3(7)
C(43)-N(5)-Zn(2B)	124.3(7)	C(92)-C(98)-C(102)	121.1(9)
C(99)-C(98)-C(92)	122.3(7)	C(99)-C(98)-C(102)	116.2(9)
N(22)-C(90)-C(87)	119.6(5)	N(20)-C(90)-N(22)	120.0
N(20)-C(90)-C(87)	120.3(5)	C(92)-N(22)-C(90)	120.0
N(22)-C(92)-C(98)	120.5(5)	N(22)-C(92)-N(21)	120.1
N(21)-C(92)-C(98)	119.0(5)	C(92)-N(21)-C(91)	120.0
N(21)-C(91)-N(20)	120.0	N(21)-C(91)-C(93)	120.9(4)
N(20)-C(91)-C(93)	119.0(4)	C(90)-N(20)-C(91)	120.1
C(50)-N(7)-Zn(3A)	130.1(9)	C(50)-N(7)-C(54)	119.7(8)
C(50)-N(7)-Zn(3B)	114.5(9)	C(54)-N(7)-Zn(3A)	109.9(7)
C(54)-N(7)-Zn(3B)	125.2(7)	N(18)-C(83)-H(83)	117.9
N(18)-C(83)-C(84)	124.1(8)	C(84)-C(83)-H(83)	117.9
C(71)-C(70)-H(70)	120.4	C(71)-C(70)-C(69)	119.1(8)
C(69)-C(70)-H(70)	120.4	C(49)-C(48)-H(48)	118.9
N(6)-C(48)-H(48)	118.9	N(6)-C(48)-C(49)	122.3(9)
C(88)-C(87)-C(90)	121.8(7)	C(86)-C(87)-C(90)	121.9(9)
C(86)-C(87)-C(88)	116.3(9)	C(34)-C(33)-H(33)	121.4
C(32)-C(33)-H(33)	121.4	C(32)-C(33)-C(34)	117.2(7)
C(70)-C(71)-H(71)	118.7	N(13)-C(71)-C(70)	122.7(9)
N(13)-C(71)-H(71)	118.7	C(83)-N(18)-Zn(6A)	116.1(7)
C(83)-N(18)-Zn(6B)	119.5(6)	C(82)-N(18)-Zn(6A)	127.7(7)
C(82)-N(18)-C(83)	116.0(7)	C(82)-N(18)-Zn(6B)	124.3(6)

C(33)-C(34)-C(37)	120.1(6)	C(35)-C(34)-C(37)	121.2(6)
C(35)-C(34)-C(33)	118.7(7)	C(34)-C(35)-H(35)	119.7
C(34)-C(35)-C(36)	120.7(7)	C(36)-C(35)-H(35)	119.7
C(87)-C(88)-H(88)	120.6	C(89)-C(88)-C(87)	118.7(9)
C(89)-C(88)-H(88)	120.6	C(69)-C(68)-H(68)	119.8
C(69)-C(68)-C(67)	120.4(8)	C(67)-C(68)-H(68)	119.8
C(97)-C(93)-C(91)	120.9(8)	C(94)-C(93)-C(91)	122.1(9)
C(94)-C(93)-C(97)	117.0(11)	C(71)-N(13)-Zn(4A)	121.2(6)
C(71)-N(13)-C(67)	118.7(7)	C(71)-N(13)-Zn(4B)	123.0(6)
C(67)-N(13)-Zn(4A)	120.1(5)	C(67)-N(13)-Zn(4B)	118.2(5)
C(70)-C(69)-C(72)	119.0(6)	C(68)-C(69)-C(72)	123.1(7)
C(68)-C(69)-C(70)	117.7(7)	N(5)-C(42)-H(42)	117.5
N(5)-C(42)-C(41)	125.0(8)	C(41)-C(42)-H(42)	117.5
C(80)-C(84)-H(84)	121.1	C(83)-C(84)-C(80)	117.8(8)
C(83)-C(84)-H(84)	121.1	C(36)-N(1)-Zn(1A)	119.8(6)
C(36)-N(1)-Zn(1B)	124.4(7)	C(32)-N(1)-Zn(1A)	123.5(6)
C(32)-N(1)-C(36)	116.4(6)	C(32)-N(1)-Zn(1B)	117.8(7)
C(35)-C(36)-H(36)	119.1	N(1)-C(36)-C(35)	121.8(7)
N(1)-C(36)-H(36)	119.1	C(42)-C(41)-H(41)	120.9
C(42)-C(41)-C(40)	118.1(8)	C(40)-C(41)-H(41)	120.9
C(78)-C(79)-H(79)	119.8	C(75)-C(79)-C(78)	120.3(9)
C(75)-C(79)-H(79)	119.8	C(45)-C(49)-H(49)	119.8
C(48)-C(49)-C(45)	120.4(8)	C(48)-C(49)-H(49)	119.8
C(40)-C(44)-H(44)	120.5	C(40)-C(44)-C(43)	119.0(9)
C(43)-C(44)-H(44)	120.5	C(75)-C(76)-H(76)	120.4
C(77)-C(76)-H(76)	120.4	C(77)-C(76)-C(75)	119.3(9)
C(48)-N(6)-Zn(3A)	121.2(6)	C(48)-N(6)-C(47)	119.0(7)
C(48)-N(6)-Zn(3B)	113.7(6)	C(47)-N(6)-Zn(3A)	119.8(6)
C(47)-N(6)-Zn(3B)	124.4(6)	C(41)-C(40)-C(38)	122.5(7)
C(44)-C(40)-C(38)	120.8(7)	C(44)-C(40)-C(41)	116.6(8)
C(46)-C(47)-H(47)	119.3	N(6)-C(47)-C(46)	121.4(8)
N(6)-C(47)-H(47)	119.3	C(33)-C(32)-H(32)	117.6
N(1)-C(32)-C(33)	124.9(7)	N(1)-C(32)-H(32)	117.6
C(81)-C(82)-H(82)	118.3	N(18)-C(82)-C(81)	123.4(8)
N(18)-C(82)-H(82)	118.3	C(93)-C(97)-H(97)	120.7
C(96)-C(97)-C(93)	118.6(11)	C(96)-C(97)-H(97)	120.7
C(79)-C(75)-C(74)	122.6(7)	C(79)-C(75)-C(76)	119.2(7)
C(76)-C(75)-C(74)	118.2(7)	C(68)-C(67)-H(67)	119.4
N(13)-C(67)-C(68)	121.3(7)	N(13)-C(67)-H(67)	119.4

C(93)-C(94)-H(94)	119.6	C(93)-C(94)-C(95)	120.9(11)
C(95)-C(94)-H(94)	119.6	C(86)-C(85)-H(85)	119.5
N(19)-C(85)-H(85)	119.5	N(19)-C(85)-C(86)	121.0(11)
C(88)-C(89)-H(89)	118.1	C(88)-C(89)-N(19)	123.8(10)
N(19)-C(89)-H(89)	118.1	N(17)-C(77)-C(76)	121.3(10)
N(17)-C(77)-H(77)	119.4	C(76)-C(77)-H(77)	119.4
N(5)-C(43)-C(44)	124.7(9)	N(5)-C(43)-H(43)	117.6
C(44)-C(43)-H(43)	117.6	C(97)-C(96)-H(96)	117.4
N(23)-C(96)-C(97)	125.2(10)	N(23)-C(96)-H(96)	117.4
C(94)-C(95)-H(95)	118.8	N(23)-C(95)-C(94)	122.4(12)
N(23)-C(95)-H(95)	118.8	C(52)-C(53)-H(53)	122.5
C(52)-C(53)-C(54)	115.1(12)	C(54)-C(53)-H(53)	122.5
N(7)-C(50)-H(50)	120.0	N(7)-C(50)-C(51)	120.0(11)
C(51)-C(50)-H(50)	120.0	C(87)-C(86)-C(85)	121.1(12)
C(87)-C(86)-H(86)	119.4	C(85)-C(86)-H(86)	119.5
C(58)-C(62)-H(62)	119.7	C(61)-C(62)-H(62)	119.7
C(61)-C(62)-C(58)	120.6(10)	C(53)-C(52)-C(55)	116.3(9)
C(51)-C(52)-C(55)	121.8(8)	C(51)-C(52)-C(53)	121.8(9)
C(85)-N(19)-Zn(6A)	120.0(9)	C(85)-N(19)-C(89)	118.8(9)
C(85)-N(19)-Zn(6B)	118.6(7)	C(89)-N(19)-Zn(6A)	120.4(10)
C(89)-N(19)-Zn(6B)	122.3(8)	N(7)-C(54)-C(53)	123.1(11)
N(7)-C(54)-H(54)	118.5	C(53)-C(54)-H(54)	118.5
C(62)-C(58)-C(57)	121.4(8)	C(62)-C(58)-C(59)	116.4(10)
C(59)-C(58)-C(57)	122.0(8)	Zn(2A)#4-N(23)-Zn(2B)#4	7.3(5)
C(96)-N(23)-Zn(2A)#4	118.0(8)	C(96)-N(23)-Zn(2B)#4	123.5(8)
C(95)-N(23)-Zn(2A)#4	125.6(9)	C(95)-N(23)-C(96)	115.9(10)
C(95)-N(23)-Zn(2B)#4	120.6(9)	C(61)-N(11)-Zn(5A)#5	119.2(8)
C(61)-N(11)-Zn(5B)#5	126.9(8)	C(60)-N(11)-Zn(5A)#5	122.3(9)
C(60)-N(11)-C(61)	118.4(10)	C(60)-N(11)-Zn(5B)#5	114.5(8)
C(50)-C(51)-H(51)	119.9	C(52)-C(51)-C(50)	120.3(9)
C(52)-C(51)-H(51)	119.9	C(62)-C(61)-N(11)	123.2(10)
C(62)-C(61)-H(61)	118.4	N(11)-C(61)-H(61)	118.4
C(58)-C(59)-H(59)	119.6	C(58)-C(59)-C(60)	120.7(10)
C(60)-C(59)-H(59)	119.6	N(11)-C(60)-C(59)	120.4(11)
N(11)-C(60)-H(60)	119.8	C(59)-C(60)-H(60)	119.8
C(99)-C(100)-H(100)	119.2	N(24)-C(100)-H(100)	119.2
N(24)-C(100)-C(99)	121.6(10)	C(98)-C(99)-C(100)	121.2(9)
C(98)-C(99)-H(99)	119.4	C(100)-C(99)-H(99)	119.4
C(98)-C(102)-H(102)	120.6	C(101)-C(102)-C(98)	118.8(12)

C(101)-C(102)-H(102)	120.6	N(13)-Zn(4B)-I(8B)	106.4(3)
N(13)-Zn(4B)-I(7B)	107.3(3)	N(13)-Zn(4B)-N(12B)	104.7(5)
I(7B)-Zn(4B)-I(8B)	126.1(2)	N(12B)-Zn(4B)-I(8B)	98.4(5)
N(12B)-Zn(4B)-I(7B)	111.8(5)	N(1)-Zn(1B)-I(2B)	110.9(7)
N(1)-Zn(1B)-I(1B)	101.4(6)	N(1)-Zn(1B)-N(24)#1	98.4(7)
I(2B)-Zn(1B)-I(1B)	124.8(6)	N(24)#1-Zn(1B)-I(2B)	112.1(7)
N(24)#1-Zn(1B)-I(1B)	105.7(6)	N(7)-Zn(3B)-I(5B)	100.8(4)
N(7)-Zn(3B)-I(6B)	114.2(4)	N(6)-Zn(3B)-N(7)	94.3(3)
N(6)-Zn(3B)-I(5B)	103.0(4)	N(6)-Zn(3B)-I(6B)	110.4(4)
I(6B)-Zn(3B)-I(5B)	128.4(2)	I(10B)-Zn(5B)-I(9B)	121.2(4)
N(17)-Zn(5B)-I(10B)	109.1(4)	N(17)-Zn(5B)-N(11)#3	101.5(5)
N(17)-Zn(5B)-I(9B)	109.4(4)	N(11)#3-Zn(5B)-I(10B)	110.4(4)
N(11)#3-Zn(5B)-I(9B)	103.4(4)	N(5)-Zn(2B)-N(23)#2	104.1(5)
N(5)-Zn(2B)-I(3B)	105.3(4)	N(5)-Zn(2B)-I(4B)	111.3(5)
N(23)#2-Zn(2B)-I(3B)	103.2(5)	N(23)#2-Zn(2B)-I(4B)	107.4(4)
I(4B)-Zn(2B)-I(3B)	123.8(4)	Zn(1A)#6-N(24)-Zn(1B)#6	8.5(6)
C(100)-N(24)-Zn(1A)#6	125.4(7)	C(100)-N(24)-Zn(1B)#6	117.2(8)
C(101)-N(24)-Zn(1A)#6	115.8(7)	C(101)-N(24)-C(100)	118.8(8)
C(101)-N(24)-Zn(1B)#6	124.0(8)	C(23A)-C(22A)-H(22A)	120.0
C(23A)-C(22A)-C(21A)	120.0	C(21A)-C(22A)-H(22A)	120.0
C(22A)-C(23A)-C(18A)	120.0	C(22A)-C(23A)-C(25A)	122.2(15)
C(18A)-C(23A)-C(25A)	117.6(15)	C(19A)-C(18A)-C(23A)	120.0
O(1A)-C(18A)-C(23A)	121.1(13)	O(1A)-C(18A)-C(19A)	118.8(13)
C(18A)-C(19A)-C(20A)	120.0	C(18A)-C(19A)-C(24A)	121.4(15)
C(20A)-C(19A)-C(24A)	118.5(15)	C(19A)-C(20A)-H(20A)	120.0
C(21A)-C(20A)-C(19A)	120.0	C(21A)-C(20A)-H(20A)	120.0
C(22A)-C(21A)-H(21A)	120.0	C(20A)-C(21A)-C(22A)	120.0
C(20A)-C(21A)-H(21A)	120.0	C(102)-C(101)-H(101)	118.8
N(24)-C(101)-C(102)	122.4(11)	N(24)-C(101)-H(101)	118.8
H(1AA)-C(1A)-H(1AB)	109.5	H(1AA)-C(1A)-H(1AC)	109.5
H(1AB)-C(1A)-H(1AC)	109.5	Si(1A)-C(1A)-H(1AA)	109.5
Si(1A)-C(1A)-H(1AB)	109.5	Si(1A)-C(1A)-H(1AC)	109.5
O(1A)-Si(1A)-C(1A)	107.1(8)	O(1A)-Si(1A)-C(2A)	107.1(9)
O(1A)-Si(1A)-C(10A)	110.2(11)	C(2A)-Si(1A)-C(1A)	114.6(11)
C(2A)-Si(1A)-C(10A)	109.9(10)	C(10A)-Si(1A)-C(1A)	107.9(11)
C(18A)-O(1A)-Si(1A)	134.1(12)	Si(1A)-C(2A)-H(2AA)	107.6
Si(1A)-C(2A)-H(2AB)	107.6	H(2AA)-C(2A)-H(2AB)	107.0
C(3A)-C(2A)-Si(1A)	118.8(16)	C(3A)-C(2A)-H(2AA)	107.6
C(3A)-C(2A)-H(2AB)	107.6	Si(1A)-C(10A)-H(10A)	108.2

Si(1A)-C(10A)-H(10B)	108.2	H(10A)-C(10A)-H(10B)	107.4
C(11A)-C(10A)-Si(1A)	116.3(15)	C(11A)-C(10A)-H(10A)	108.2
C(11A)-C(10A)-H(10B)	108.2	C(19A)-C(24A)-H(24A)	109.5
C(19A)-C(24A)-H(24B)	109.5	C(19A)-C(24A)-H(24C)	109.5
H(24A)-C(24A)-H(24B)	109.5	H(24A)-C(24A)-H(24C)	109.5
H(24B)-C(24A)-H(24C)	109.5	C(23A)-C(25A)-H(25A)	109.5
C(23A)-C(25A)-H(25B)	109.5	C(23A)-C(25A)-H(25C)	109.5
H(25A)-C(25A)-H(25B)	109.5	H(25A)-C(25A)-H(25C)	109.5
H(25B)-C(25A)-H(25C)	109.5	H(17A)-C(17A)-H(17B)	109.5
H(17A)-C(17A)-H(17C)	109.5	H(17B)-C(17A)-H(17C)	109.5
C(14A)-C(17A)-H(17A)	109.5	C(14A)-C(17A)-H(17B)	109.5
C(14A)-C(17A)-H(17C)	109.5	C(16A)-C(11A)-C(10A)	121.7(15)
C(16A)-C(11A)-C(12A)	120.0	C(12A)-C(11A)-C(10A)	117.9(15)
C(11A)-C(16A)-H(16A)	120.0	C(15A)-C(16A)-C(11A)	120.0
C(15A)-C(16A)-H(16A)	120.0	C(16A)-C(15A)-H(15A)	120.0
C(16A)-C(15A)-C(14A)	120.0	C(14A)-C(15A)-H(15A)	120.0
C(15A)-C(14A)-C(17A)	129.1(19)	C(13A)-C(14A)-C(17A)	110.9(19)
C(13A)-C(14A)-C(15A)	120.0	C(14A)-C(13A)-H(13A)	120.0
C(14A)-C(13A)-C(12A)	120.0	C(12A)-C(13A)-H(13A)	120.0
C(11A)-C(12A)-H(12A)	120.0	C(13A)-C(12A)-C(11A)	120.0
C(13A)-C(12A)-H(12A)	120.0	C(2A)-C(3A)-C(6A)	105(2)
C(4A)-C(3A)-C(2A)	104(3)	C(4A)-C(3A)-C(6A)	126(4)
C(4A)-C(3A)-C(5A)	107(3)	C(5A)-C(3A)-C(2A)	111(2)
C(5A)-C(3A)-C(6A)	104(3)	C(3A)-C(6A)-H(6AA)	107.5
C(3A)-C(6A)-H(6AB)	107.5	H(6AA)-C(6A)-H(6AB)	107.0
C(7A)-C(6A)-C(3A)	119(2)	C(7A)-C(6A)-H(6AA)	107.5
C(7A)-C(6A)-H(6AB)	107.5	C(3A)-C(4A)-H(4AA)	109.5
C(3A)-C(4A)-H(4AB)	109.5	C(3A)-C(4A)-H(4AC)	109.5
H(4AA)-C(4A)-H(4AB)	109.5	H(4AA)-C(4A)-H(4AC)	109.5
H(4AB)-C(4A)-H(4AC)	109.5	C(3A)-C(5A)-H(5AA)	109.5
C(3A)-C(5A)-H(5AB)	109.5	C(3A)-C(5A)-H(5AC)	109.5
H(5AA)-C(5A)-H(5AB)	109.5	H(5AA)-C(5A)-H(5AC)	109.5
H(5AB)-C(5A)-H(5AC)	109.5	C(6A)-C(7A)-C(9A)	125(3)
C(8A)-C(7A)-C(6A)	109(4)	C(8A)-C(7A)-C(9A)	126(4)
C(7A)-C(9A)-H(9AA)	109.5	C(7A)-C(9A)-H(9AB)	109.5
C(7A)-C(9A)-H(9AC)	109.5	H(9AA)-C(9A)-H(9AB)	109.5
H(9AA)-C(9A)-H(9AC)	109.5	H(9AB)-C(9A)-H(9AC)	109.5
C(7A)-C(8A)-H(8AA)	120.0	C(7A)-C(8A)-H(8AB)	120.0
H(8AA)-C(8A)-H(8AB)	120.0	C(64B)-N(12B)-Zn(4B)	118.2(8)

C(64B)-N(12B)-C(65B)	120.0	C(65B)-N(12B)-Zn(4B)	121.7(8)
N(12B)-C(64B)-H(64B)	120.1	N(12B)-C(64B)-C(63B)	119.9
C(63B)-C(64B)-H(64B)	120.1	C(64B)-C(63B)-H(63B)	120.0
C(62B)-C(63B)-C(64B)	119.9	C(62B)-C(63B)-H(63B)	120.0
C(63B)-C(62B)-C(56)	115.1(10)	C(66B)-C(62B)-C(56)	124.4(10)
C(66B)-C(62B)-C(63B)	120.1	C(62B)-C(66B)-H(66B)	120.0
C(62B)-C(66B)-C(65B)	120.1	C(65B)-C(66B)-H(66B)	120.0
N(12B)-C(65B)-H(65B)	120.0	C(66B)-C(65B)-N(12B)	120.0
C(66B)-C(65B)-H(65B)	120.0	N(18)-Zn(6B)-N(19)	96.3(3)
N(18)-Zn(6B)-I(12B)	107.1(3)	N(18)-Zn(6B)-I(11B)	104.5(3)
N(19)-Zn(6B)-I(12B)	103.1(3)	N(19)-Zn(6B)-I(11B)	110.5(3)
I(11B)-Zn(6B)-I(12B)	130.28(19)	H(1CA)-C(1C)-H(1CB)	109.5
H(1CA)-C(1C)-H(1CC)	109.5	H(1CB)-C(1C)-H(1CC)	109.5
Si(1C)-C(1C)-H(1CA)	109.5	Si(1C)-C(1C)-H(1CB)	109.5
Si(1C)-C(1C)-H(1CC)	109.5	O(2C)-Si(1C)-C(1C)	107.4(16)
O(2C)-Si(1C)-C(10C)	110.7(16)	O(2C)-Si(1C)-C(2C)	109.0(15)
C(10C)-Si(1C)-C(1C)	113.4(15)	C(10C)-Si(1C)-C(2C)	110.1(14)
C(2C)-Si(1C)-C(1C)	105.9(15)	C(18C)-O(2C)-Si(1C)	130.7(16)
Si(1C)-C(10C)-H(10C)	108.1	Si(1C)-C(10C)-H(10D)	108.1
H(10C)-C(10C)-H(10D)	107.3	C(11C)-C(10C)-Si(1C)	116.6(19)
C(11C)-C(10C)-H(10C)	108.1	C(11C)-C(10C)-H(10D)	108.1
Si(1C)-C(2C)-H(2CA)	101.7	Si(1C)-C(2C)-H(2CB)	101.7
H(2CA)-C(2C)-H(2CB)	104.7	C(3C)-C(2C)-Si(1C)	141(3)
C(3C)-C(2C)-H(2CA)	101.7	C(3C)-C(2C)-H(2CB)	101.7
O(2C)-C(18C)-C(23C)	119(3)	O(2C)-C(18C)-C(19C)	121(3)
C(23C)-C(18C)-C(19C)	120.0	C(18C)-C(23C)-C(22C)	120.0
C(18C)-C(23C)-C(25C)	129(2)	C(22C)-C(23C)-C(25C)	111(2)
C(23C)-C(22C)-H(22C)	120.0	C(23C)-C(22C)-C(21C)	120.0
C(21C)-C(22C)-H(22C)	120.0	C(22C)-C(21C)-H(21C)	120.0
C(20C)-C(21C)-C(22C)	120.0	C(20C)-C(21C)-H(21C)	120.0
C(21C)-C(20C)-H(20C)	120.0	C(21C)-C(20C)-C(19C)	120.0
C(19C)-C(20C)-H(20C)	120.0	C(18C)-C(19C)-C(24C)	123(3)
C(20C)-C(19C)-C(18C)	120.0	C(20C)-C(19C)-C(24C)	117(3)
C(23C)-C(25C)-H(25D)	109.5	C(23C)-C(25C)-H(25E)	109.5
C(23C)-C(25C)-H(25F)	109.5	H(25D)-C(25C)-H(25E)	109.5
H(25D)-C(25C)-H(25F)	109.5	H(25E)-C(25C)-H(25F)	109.5
C(19C)-C(24C)-H(24D)	109.5	C(19C)-C(24C)-H(24E)	109.5
C(19C)-C(24C)-H(24F)	109.5	H(24D)-C(24C)-H(24E)	109.5
H(24D)-C(24C)-H(24F)	109.5	H(24E)-C(24C)-H(24F)	109.5

H(2BA)-C(2B)-H(2BB)	107.0	C(1B)-C(2B)-H(2BA)	107.5
C(1B)-C(2B)-H(2BB)	107.5	C(1B)-C(2B)-C(3B)	119(2)
C(3B)-C(2B)-H(2BA)	107.5	C(3B)-C(2B)-H(2BB)	107.5
C(2B)-C(1B)-H(1BA)	107.4	C(2B)-C(1B)-H(1BB)	107.4
C(2B)-C(1B)-C(6B)	120(3)	H(1BA)-C(1B)-H(1BB)	106.9
C(6B)-C(1B)-H(1BA)	107.4	C(6B)-C(1B)-H(1BB)	107.4
C(1B)-C(6B)-H(6BA)	108.5	C(1B)-C(6B)-H(6BB)	108.5
H(6BA)-C(6B)-H(6BB)	107.5	C(5B)-C(6B)-C(1B)	115(3)
C(5B)-C(6B)-H(6BA)	108.5	C(5B)-C(6B)-H(6BB)	108.5
C(6B)-C(5B)-H(5BA)	108.7	C(6B)-C(5B)-H(5BB)	108.7
C(6B)-C(5B)-C(4B)	114(4)	H(5BA)-C(5B)-H(5BB)	107.6
C(4B)-C(5B)-H(5BA)	108.7	C(4B)-C(5B)-H(5BB)	108.7
C(5B)-C(4B)-H(4BA)	107.9	C(5B)-C(4B)-H(4BB)	107.9
H(4BA)-C(4B)-H(4BB)	107.2	C(3B)-C(4B)-C(5B)	118(3)
C(3B)-C(4B)-H(4BA)	107.9	C(3B)-C(4B)-H(4BB)	107.9
C(2B)-C(3B)-H(3BA)	107.6	C(2B)-C(3B)-H(3BB)	107.6
C(4B)-C(3B)-C(2B)	119(3)	C(4B)-C(3B)-H(3BA)	107.6
C(4B)-C(3B)-H(3BB)	107.6	H(3BA)-C(3B)-H(3BB)	107.0
H(29A)-C(29)-H(29B)	107.8	C(30)-C(29)-H(29A)	109.1
C(30)-C(29)-H(29B)	109.1	C(30)-C(29)-C(28)	113(3)
C(28)-C(29)-H(29A)	109.1	C(28)-C(29)-H(29B)	109.1
C(29)-C(30)-H(30A)	108.4	C(29)-C(30)-H(30B)	108.4
H(30A)-C(30)-H(30B)	107.5	C(31)-C(30)-C(29)	115(2)
C(31)-C(30)-H(30A)	108.4	C(31)-C(30)-H(30B)	108.4
C(30)-C(31)-H(31A)	110.0	C(30)-C(31)-H(31B)	110.0
C(30)-C(31)-C(26)	108(3)	H(31A)-C(31)-H(31B)	108.4
C(26)-C(31)-H(31A)	110.0	C(26)-C(31)-H(31B)	110.0
C(31)-C(26)-H(26A)	110.5	C(31)-C(26)-H(26B)	110.5
H(26A)-C(26)-H(26B)	108.7	C(27)-C(26)-C(31)	106(2)
C(27)-C(26)-H(26A)	110.5	C(27)-C(26)-H(26B)	110.5
C(26)-C(27)-H(27A)	109.7	C(26)-C(27)-H(27B)	109.7
H(27A)-C(27)-H(27B)	108.2	C(28)-C(27)-C(26)	110(3)
C(28)-C(27)-H(27A)	109.7	C(28)-C(27)-H(27B)	109.7
C(29)-C(28)-H(28A)	109.1	C(29)-C(28)-H(28B)	109.1
C(27)-C(28)-C(29)	113(2)	C(27)-C(28)-H(28A)	109.1
C(27)-C(28)-H(28B)	109.1	H(28A)-C(28)-H(28B)	107.8
C(13C)-C(14C)-C(15C)	120.0	C(13C)-C(14C)-C(17C)	116(2)
C(15C)-C(14C)-C(17C)	123(2)	C(14C)-C(13C)-H(13C)	120.0
C(12C)-C(13C)-C(14C)	120.0	C(12C)-C(13C)-H(13C)	120.0

C(13C)-C(12C)-H(12C)	120.0	C(13C)-C(12C)-C(11C)	120.0
C(11C)-C(12C)-H(12C)	120.0	C(12C)-C(11C)-C(10C)	117(2)
C(16C)-C(11C)-C(10C)	122(2)	C(16C)-C(11C)-C(12C)	120.0
C(11C)-C(16C)-H(16C)	120.0	C(15C)-C(16C)-C(11C)	120.0
C(15C)-C(16C)-H(16C)	120.0	C(14C)-C(15C)-H(15C)	120.0
C(16C)-C(15C)-C(14C)	120.0	C(16C)-C(15C)-H(15C)	120.0
C(14C)-C(17C)-H(17D)	109.5	C(14C)-C(17C)-H(17E)	109.5
C(14C)-C(17C)-H(17F)	109.5	H(17D)-C(17C)-H(17E)	109.5
H(17D)-C(17C)-H(17F)	109.5	H(17E)-C(17C)-H(17F)	109.5
C(2C)-C(3C)-C(6C)	112(3)	C(2C)-C(3C)-C(4C)	112(3)
C(2C)-C(3C)-C(5C)	105(3)	C(4C)-C(3C)-C(6C)	104(3)
C(5C)-C(3C)-C(6C)	112(3)	C(5C)-C(3C)-C(4C)	112(3)
C(3C)-C(6C)-H(6CA)	110.7	C(3C)-C(6C)-H(6CB)	110.7
H(6CA)-C(6C)-H(6CB)	108.8	C(7C)-C(6C)-C(3C)	105(2)
C(7C)-C(6C)-H(6CA)	110.7	C(7C)-C(6C)-H(6CB)	110.7
C(3C)-C(4C)-H(4CA)	109.5	C(3C)-C(4C)-H(4CB)	109.5
C(3C)-C(4C)-H(4CC)	109.5	H(4CA)-C(4C)-H(4CB)	109.5
H(4CA)-C(4C)-H(4CC)	109.5	H(4CB)-C(4C)-H(4CC)	109.5
C(3C)-C(5C)-H(5CA)	109.5	C(3C)-C(5C)-H(5CB)	109.5
C(3C)-C(5C)-H(5CC)	109.5	H(5CA)-C(5C)-H(5CB)	109.5
H(5CA)-C(5C)-H(5CC)	109.5	H(5CB)-C(5C)-H(5CC)	109.5
C(6C)-C(7C)-C(9C)	113(3)	C(8C)-C(7C)-C(6C)	119(3)
C(8C)-C(7C)-C(9C)	129(4)	C(7C)-C(9C)-H(9CA)	109.5
C(7C)-C(9C)-H(9CB)	109.5	C(7C)-C(9C)-H(9CC)	109.5
H(9CA)-C(9C)-H(9CB)	109.5	H(9CA)-C(9C)-H(9CC)	109.5
H(9CB)-C(9C)-H(9CC)	109.5	C(7C)-C(8C)-H(8CA)	120.0
C(7C)-C(8C)-H(8CB)	120.0	H(8CA)-C(8C)-H(8CB)	120.0

Symmetry transformations used to generate equivalent atoms:

#1 x+1,y,z+1 #2 x+1/2,y-1/2,z+1 #3 x-1/2,y+1/2,z

#4 x-1/2,y+1/2,z-1 #5 x+1/2,y-1/2,z #6 x-1,y,z-1

HPLC analysis of inclusion crystal extract

After single crystal X-ray analysis of the inclusion crystal, the presence of **4b** as a guest in the CS was proven by HPLC analysis. This ensures a correct and meaningful structure model. One of the three crystals, from the same CS inclusion (**4b** $(ZnI_2)_3(tpt)_2$ cyclohexane solvate), was lost during cryo transportation to the synchrotron facility. A second crystal does not show any diffraction, therefore only the remaining third crystal was investigated.

To verify the retention times the original DCM solution (1 mg / mL) of **4b** was used as a reference. 0.5 µL were injected, 150 mm YMC Cellulose SJ stationary phase with 1.8 µm and 4.6 mm i.D., acetonitrile/water = 60:40 mobile phase, 1.0 mL / min flow rate, 19.5 MPa pressure, 298 K temperature, UV detection at 220 nm.

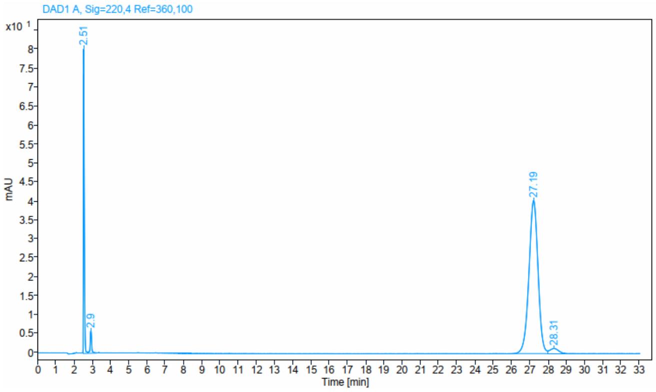

Signal: DAD1 A, Sig=220,4 Ref=360,100

RT [min]	Area	Area%	Name
2.51	269.96	15.42	
2.90	33.94	1.94	
27.19	1400.93	80.04	1. Enantiomer
28.31	45.39	2.59	2. Enantiomer
Sum	1750.2193		

Figure S26. HPLC-UV/VIS chromatogram of neat **4b** with optimized separation conditions.

The optimized HPLC routine was then used to analyze the CS inclusions.[28] Initially the hosting CS was transferred to a new vial and washed with 1 mL of water (gentle shaking). To dissolve the framework of the investigated crystals, 250 µL of dissolution medium (acetone:acetonitrile:methanol:water; 25% V/V each) was added and the solution placed in an oven at 25 °C for 12 h. After complete CS dissolution and release of guest molecules, the sample solution (100 µL) was diluted with acetonitrile:water (900 µL, 50:50) and analyzed using HPLC-UV/VIS with a chiral stationary phase and the optimized separation method.

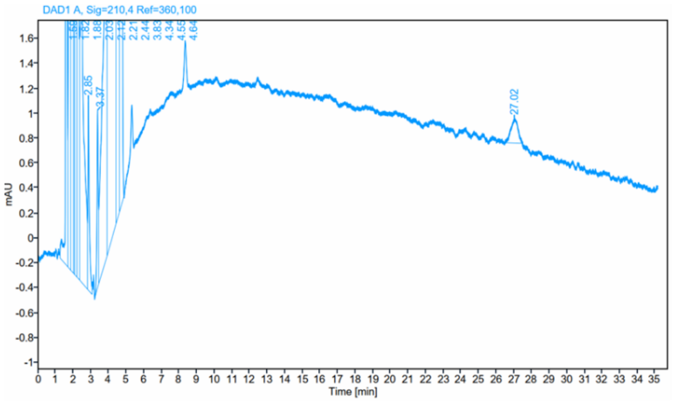

Signal: DAD1 A, Sig=210,4 Ref=360,100

RT [min]	Area	Area%	Name
1.59	73.63	7.91	
1.82	159.65	17.14	
1.88	258.21	27.72	
2.03	24.13	2.59	
2.12	76.30	8.19	
2.21	30.24	3.25	
2.44	56.66	6.08	
2.85	8.50	0.91	
3.37	6.98	0.75	
3.83	46.50	4.99	
4.34	101.80	10.93	
4.55	51.08	5.48	
4.64	32.43	3.48	
27.02	5.33	0.57	1. Enantiomer
Sum	931.4259		

Figure S27. HPLC-UV/VIS chromatogram of dissolved CS with inclusions of **4b**.

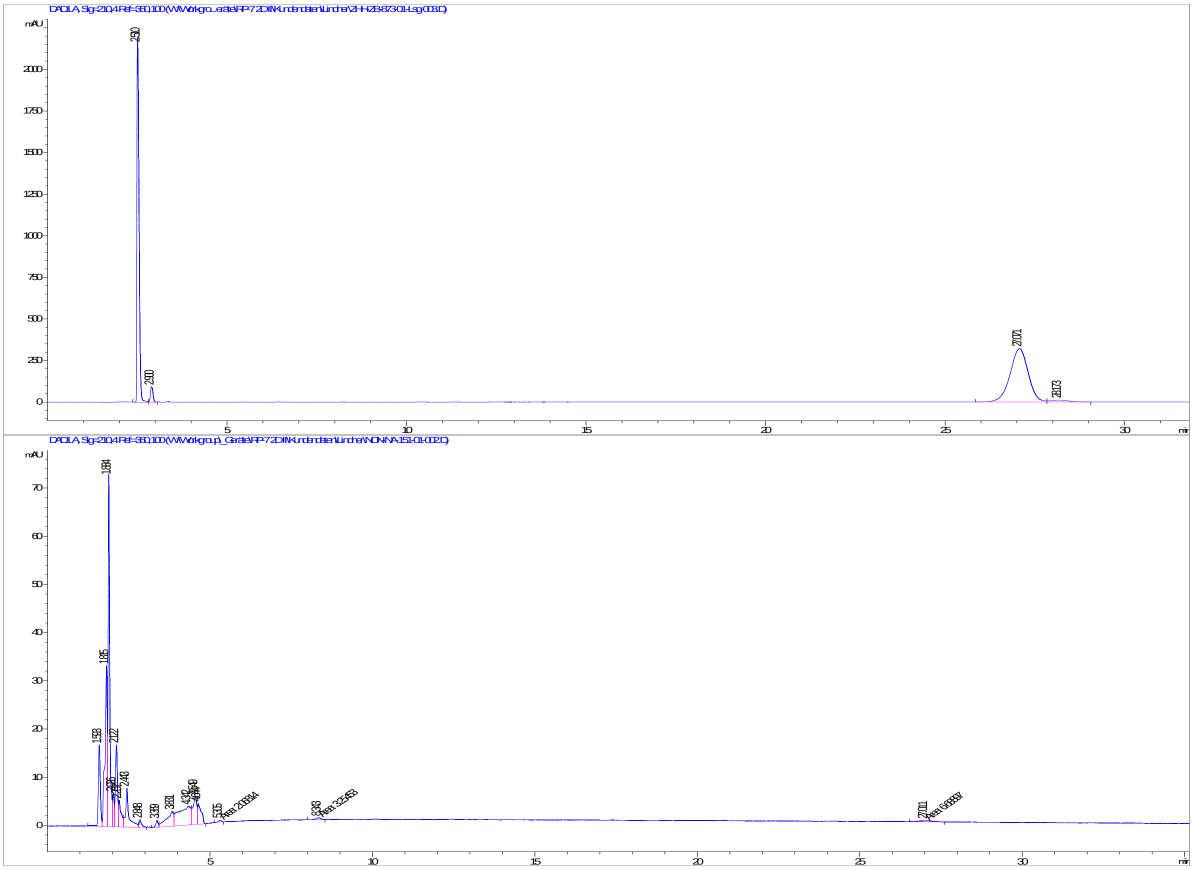

Figure S28. Comparison of HPLC-UV/VIS for neat **4b** (top) and dissolved CS with inclusions of **4b** (bottom).

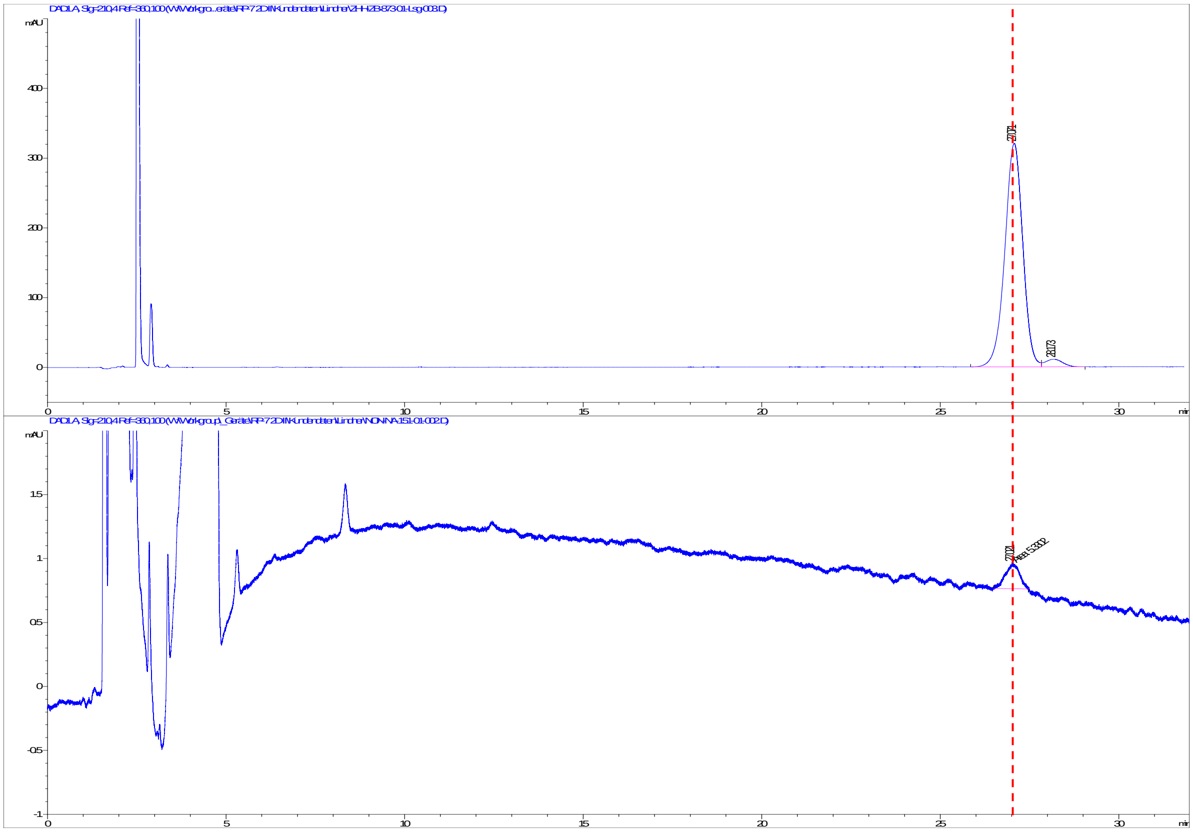

Figure S29. Comparison of intensity scaled HPLC-UV/VIS for neat **4b** (top) and dissolved CS with inclusions of **4b** (bottom). Red line indicating the identical retention times.

The retention times found here ($\overline{RT}$ = 27.02 min) are in good agreement with those from the neat compound. This clearly proves the presence of **4b** as an inclusion guest in the CS and supports the findings of the single crystal X-ray structure determination.

Additional confirming experiments

To confirm the results of the absolute structure determination, the soaking experiments were repeated. Here the same enantiomer as before of compound **4b** was used three times, the opposite enantiomer also three times and finally an experiment using a racemic mixture was executed. All soaking experiments have been re-executed using the same experimental conditions as reported above for the initial experiments. The results are shown in the following table S6.

Workflow of the method

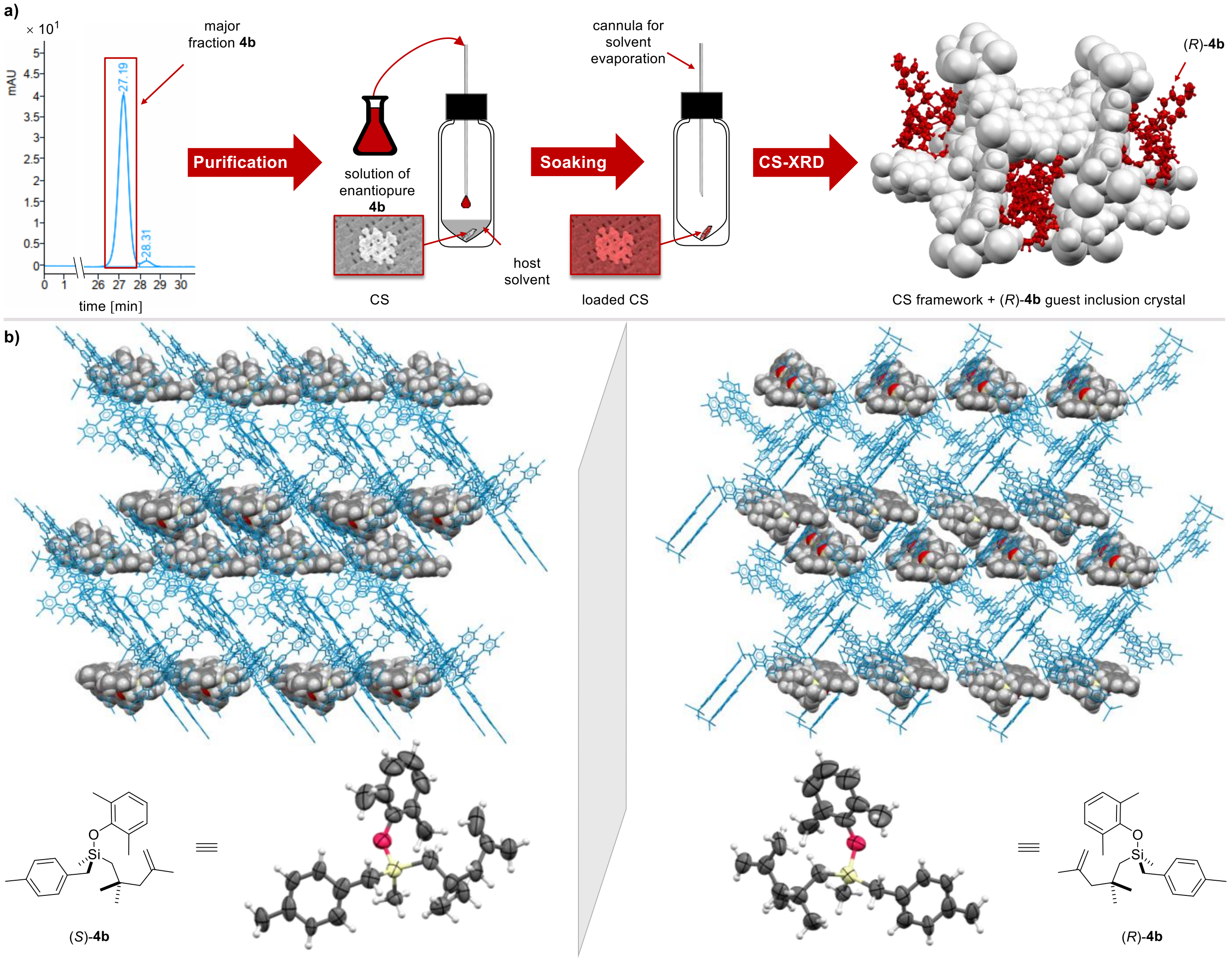

Figure S30. a) schematic workflow of absolute structure determination of product **4b** using the crystalline sponge method (CS-XRD); b) crystal structure and ellipsoid plot of both enantiomers.

Table S6. Additional confirming experiments

Additional soaking experiments were performed with the enantiopure enantiomer **4b**, the opposite enantiomer and a racemic mixture of both (as the structure could be confirmed from molecules clearly assignable on position 1, not all highly disordered moieties on the special position 2 were refined).

Exp. No.	Analyte	Absolute configuration determined by CS-XRD	R_1 (%)	R_{int} (%)	Flack (Parsons) x	Analyte occupancy position 1/ position 2 (%)	Soaking Experiment Details
1	Enantiopure compound **4b** (same enantiomer as before)	*(R)*	5.65	2.29	0.071(5)	61.2 / not assigned	$[(ZnI_2)_3{\cdot}(tpt)_2{\cdot}x(cyclohexane)]_n$-sponge, 50 °C, 3 µg analyte
2		*(R)*	5.61	2.47	0.066(4)	55.5 / not assigned	
3		*(R)*	5.27	3.06	0.032(5)	60.0 / 52.4	$[(ZnI_2)_3{\cdot}(tpt)_2{\cdot}x(n\text{-hexane})]_n$-sponge, 50 °C, 3 µg analyte
4	Opposite enantiomer of **4b**, enantiopure	*(S)*	5.94	2.88	0.058(4)	63.3 / not assigned	$[(ZnI_2)_3{\cdot}(tpt)_2{\cdot}x(cyclohexane)]_n$-sponge, 50 °C, 3 µg analyte
5		*(S)*	6.30	2.74	0.063(5)	49.9 / not assigned	
6		*(S)*	5.19	2.66	0.030(5)	53.6 / not assigned	
7	Racemic mixture of both enantiomers of **4b**	*(R/S)*	6.79	2.95	0.483(7)	52.9 / 51.0	$[(ZnI_2)_3{\cdot}(tpt)_2{\cdot}x(cyclohexane)]_n$-sponge, 50 °C, 1 µg of each enantiomer

Instrument parameters: Rigaku XtaLAB Synergy-R, HyPix-Arc 150; Cu K_α (1.54184 Å); $T_{measurement}$ = 100 K; .cif-files of the experiments 1 to 7 are available under the CCDC numbers **CCDC-2166402-2166408.**

In all experiments, the molecules in position 1 could directly and unequivocally be found in the difference electron density. Molecules on position 2 were mostly disordered due to the 2-fold rotational axis and therefore mostly not modelled to avoid introducing a bias. In all experiments with **4b**, the *(R)*-configuration was confirmed with R_1 well below 10% and Flack-x parameter < 0.1. Correspondingly, all experiments with the opposite enantiomer consistently yielded (*S*)-configuration with similar quality. There is no clear correlation between the occupation of the analyte molecules and the Flack-x parameter values.

The racemic mixture of both enantiomers in experiment no. 7 yielded both configurations present in the structure. The structure has forcefully been described in space group *C*2 to enable the comparison of the Flack-x parameters from all experiments despite the fact, that the diffraction pattern of experiment no. 7 clearly indicated presence of the c glide plane and thus space group *C*2/c (please note that the deposited structure of the included racemate is described in *C*2/c, **CCDC-2166408**). For all other experiments (1-6; enantiopure compounds), significant reflections clearly indicate absence of the c glide plane (see also Figure S19). Consequently, the Flack-x parameter of the structure model of no. 7 is ~0.5 which is in consistency with the expected value for racemic compounds. Additionally, the occupation of both enantiomers in the structure is equal within the errors of the determination.[29]

The experiments thus prove, that it is possible to discern between the two enantiomerically pure analytes as well as a racemic mixture. The absolute configuration has thus been reproducibly confirmed by six independent experiments with both enantiomers, proving that the absolute structure can reliably be determined by CS-XRD.

11. DFT calculations and CD experiments

To gain insights into the origin of the high enantioselectivities, a preliminary DFT calculation was performed. Possible conformations were explored and optimized by the artificial force induced reaction (AFIR) method[30] implemented in the global route reaction mapping (GRRM) program.[31] Conformational searches on the transition states and the pre-reaction complex have been performed at GFN2-xTB level of theory,[32, 33] using SC-AFIR[34] in combination with ORCA program version 4.2.1.[35] Geometry optimization of pre-reaction complexes was performed by Gaussian 16[36] program at B3LYP-D3/6-31G(d) level of theory.[37, 38] Other molecular geometries were optimized using GRRM program at the B3LYP-D3/6-31G(d) level of theory. Thermal free energy corrections have been performed at 253.150 K at the B3LYP-D3/6-31G(d) level of theory, using Gaussian16 program. Transition state structures were verified by the presence of a single imaginary vibrational frequency. All Single point calculations presented in this paper were carried out using Gaussian16 program at the wB97XD/6-311+G(d,p) level of theory.[39] Solvation effect has been accounted by using CPCM (toluene) solvation model,[40] as implemented in Gaussian16. The molecular structures were visualized with the UCSF Chimera program.[41]

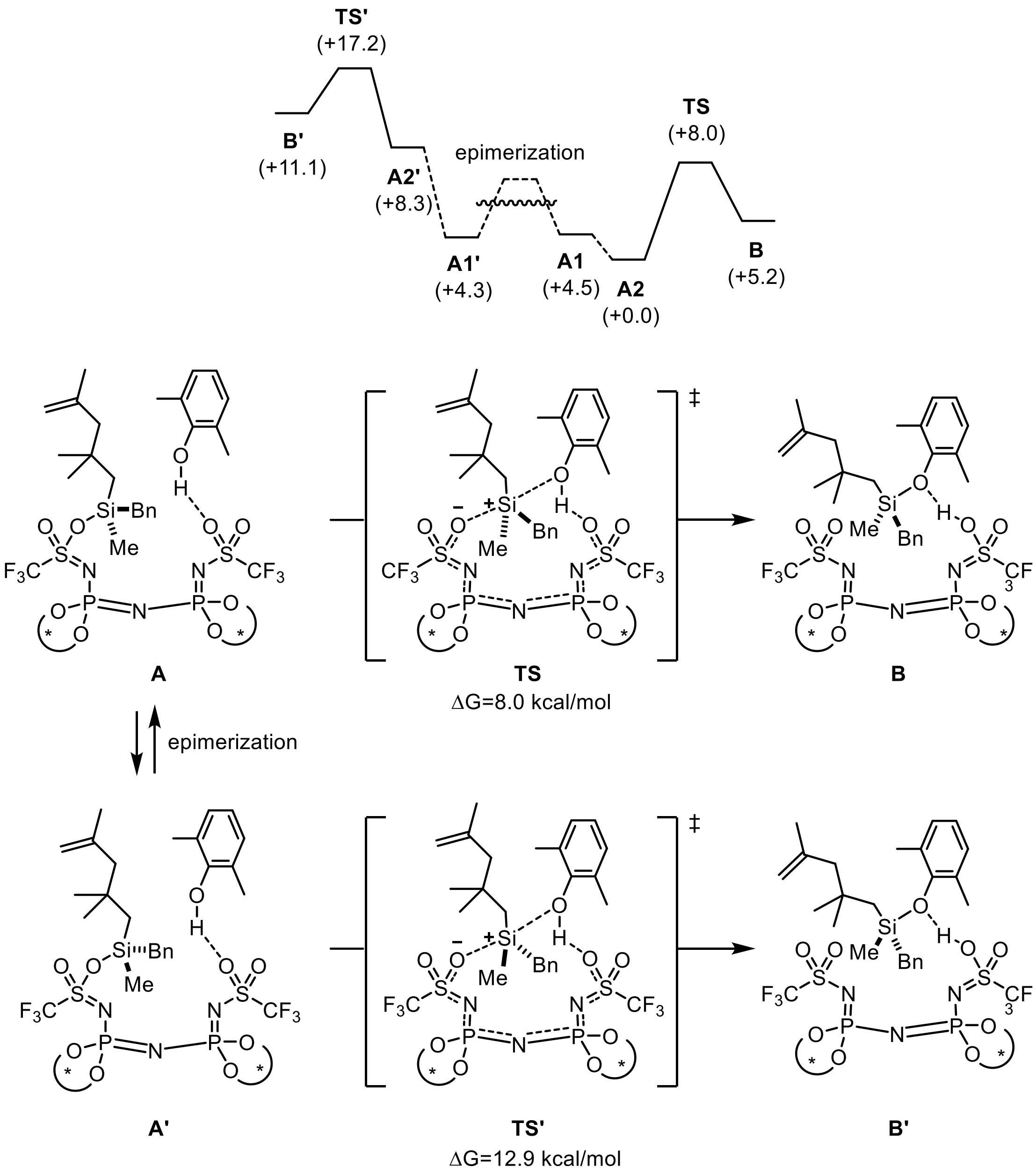

Figure S31. Summary of the computational details. The calculation was performed at the wB97XD-CPCM(Toluene)/6-311+G(d,p)//B3LYP-D3/6-31G(d) level of theory.

Based on the experimental observations (Figure 3), we have hypothesized that the enantio-determining step would be the Si–O bond formation step and the epimerization should be faster than that. Thus, two Si–O bond forming transition states leading to each enantiomer and their intrinsic reaction coordinates are calculated (Fig S1, **A2-TS-B** and **A2’-TS’-B’**). Additionally, pre-reaction complexes were separately sampled and optimized (**A1** and **A1’**). Although the absolute values of the imaginary frequencies of these transition states were relatively small, the optimization procedures are convergent, as the root-mean-square and the maximum force become smaller than 0.0002 and 0.0003 hartree Å^{-1}, respectively. The corresponding vibrations were also confirmed.

side view: top view:

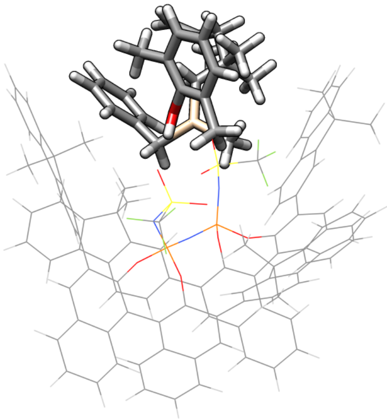

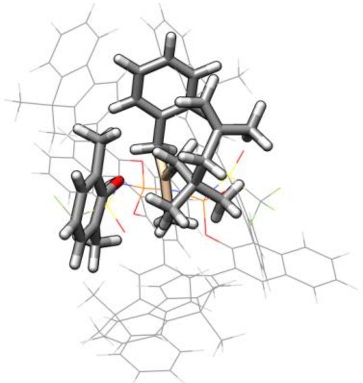

TS
$\Delta\Delta G^{calc}$ = +0.0 kcal/mol
$\Delta\Delta G^{exp}$ = +0.0 kcal/mol

side view: top view:

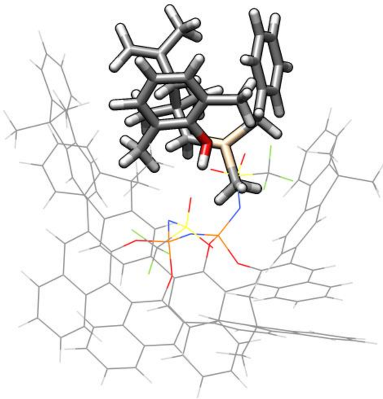

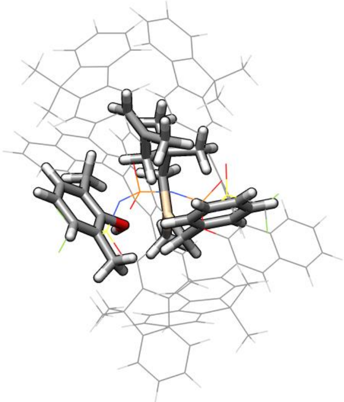

TS'
$\Delta\Delta G^{calc}$ = +4.9 kcal/mol
$\Delta\Delta G^{exp}$ = +1.7 kcal/mol

Figure S32. Comparison between the transition states leading to the products of each enantiomer. **TS** leads to the major enantiomer and **TS'** leads to the minor isomer.

The calculation results qualitatively describe the observed enantioselectivity, even though the energy difference was slightly overestimated. According to the calculated transition states, in addition to the catalyst substituents, the steric parameters on the substrates, both nucleophile and electrophile, seem to play important roles (Fig. S2). The bulky nucleophile seems to restrict the conformation of the silylium group, and it approaches to the Si center in a manner avoiding the steric repulsion from the sterically demanding *gem*-dimethyl group of the silyl substituent. By comparing two transition state structures, 2,6-positions of phenol would then induce the steric hindrance against the dimethyl fluorenyl substituents of the catalyst, especially in **TS'**, which leads to the minor enantiomer; this explains the relatively poor enantioselectivity of the sterically less demanding nucleophiles (Table S2).

Table S7. Summary of the computational study.

	Thermal corrections (in hartree)	wB97XD/6-311+G(d,p) single point (toluene solv.)	Imaginary freq.	Gibbs free energy (in hartree)	Relative energy (in kcal/mol)
**A1**	1.913133	-8074.695112	-	-8072.781979	+4.5

**A1’**	1.91122	-8074.693427	-	-8072.782207	+4.3
**A2**	1.915414	-8074.704542	-	-8072.789128	+0.0
**A2’**	1.910727	-8074.686704	-	-8072.775977	+8.3
**TS**	1.915858	-8074.692314	-21.01157938	-8072.776456	+8.0
**TS’**	1.914077	-8074.67572	-52.52308072	-8072.761643	+17.2
**B**	1.914288	-8074.695088	-	-8072.780800	+5.2
**B’**	1.910409	-8074.681897	-	-8072.771488	+11.1

A1:

O 0.388476 1.813065 2.659359

O 2.371032 1.470659 1.102544

O -0.082698 2.565106 -2.446991

O -2.173981 2.114308 -1.073187

N 0.675999 -0.465038 1.422862

S 0.547492 -0.755984 -2.246687

S -0.614867 -1.259171 1.714961

O 1.824928 -0.085165 -1.954171

O 0.411151 -2.162974 -1.828029

O -1.936666 -0.706725 1.426815

O -0.392242 -2.695209 1.186212

N -0.758630 0.077657 -1.869943

P -0.673322 1.572348 -1.297532

N 0.059123 1.851547 0.069082

P 0.805858 1.150688 1.234597

Si -1.446405 -3.902549 0.351403

C -2.474050 -4.638823 1.747444

H -2.760013 -3.780713 2.369204

H -3.414431 -4.963817 1.279787

C -0.237094 -5.011901 -0.525603

H 0.302703 -4.454493 -1.293505

H 0.493189 -5.476041 0.139668

H -0.808726 -5.802302 -1.025337

O	2.883471	-3.778524	-1.783834
H	2.378959	-3.039664	-1.404149
C	-1.951072	-5.782038	2.653438
C	3.110873	-4.759441	-0.855211
C	-2.981941	-6.022260	3.810103
H	-2.613991	-6.866925	4.410498
H	-3.914530	-6.358628	3.339201
C	3.691074	-7.070353	-0.492502
C	3.564691	-6.893647	0.886352
C	3.207191	-5.641512	1.382081
C	2.986830	-4.550071	0.530075
C	3.456649	-6.018951	-1.379860
H	3.958850	-8.045222	-0.893771
H	3.736993	-7.724233	1.565406
H	3.100674	-5.490812	2.454628
C	3.260122	1.657745	2.164335
C	0.590513	3.207690	2.695423
C	-0.093144	3.924202	-2.094091
C	-2.945840	2.687111	-2.082269
C	4.437816	0.841483	2.202573
C	-4.169425	2.037752	-2.429361
C	-0.539351	4.056082	2.530351
C	1.133783	4.525241	-1.693096
C	4.486598	3.909325	6.294079
C	3.608217	3.771135	5.242264
C	3.877003	2.862983	4.182300
C	5.064511	2.071788	4.248656
C	5.962418	2.253721	5.336741
C	5.683135	3.153651	6.338281
H	4.257673	4.602492	7.098747
H	2.694218	4.353655	5.222411
C	3.012300	2.698344	3.049189
C	5.323792	1.124888	3.233507

H	6.868986	1.654100	5.368077
H	6.270447	0.597925	3.275611
C	2.520786	7.855942	2.372209
C	1.244266	7.343802	2.390653
C	1.018971	5.946566	2.521146
C	2.136917	5.067987	2.688925
C	3.443751	5.628982	2.647855
C	3.629797	6.984180	2.485836
H	-1.126724	6.098339	2.351047
H	0.382687	7.995597	2.278671
C	-0.291350	5.414015	2.467513
C	1.890795	3.657111	2.824312
H	4.304207	4.975979	2.729712
H	4.638142	7.386296	2.445172
C	-2.608920	7.878543	-0.835636
C	-2.594813	6.615689	-1.386014
C	-1.373061	5.910843	-1.571302
C	-0.157538	6.534909	-1.142509
C	-0.204022	7.852242	-0.610817
C	-1.401361	8.512370	-0.456811
H	-3.555450	8.392506	-0.693300
H	-3.526924	6.142550	-1.672264
C	-1.311186	4.579933	-2.107892
C	1.066548	5.830341	-1.241653
H	0.729071	8.316976	-0.305752
H	1.980376	6.330216	-0.933525
C	-4.875354	5.515316	-5.795942
C	-5.300419	4.393098	-5.124081
C	-4.518972	3.827696	-4.079385
C	-3.289495	4.457042	-3.705469
C	-2.869857	5.604036	-4.432844
C	-3.641531	6.117549	-5.451639
H	-5.899445	2.232643	-3.666148

H	-6.237882	3.910667	-5.390332
H	-1.924208	6.071839	-4.184553
H	-3.297420	6.990651	-5.999098
C	-4.934211	2.655850	-3.405269
C	-2.530257	3.892126	-2.626102
H	6.371356	3.278845	7.169565
H	2.681286	8.924858	2.261271
H	-1.424383	9.515565	-0.039780
H	-5.477032	5.934575	-6.597552
C	0.433419	-0.811386	-4.110655
F	0.490522	0.432352	-4.603219
F	1.451342	-1.527552	-4.599412
F	-0.718554	-1.375228	-4.493555
C	2.430060	3.805603	-1.737137
C	2.822447	3.053689	-2.862497
C	3.309583	3.918710	-0.646100
C	4.921132	2.530205	-1.757317
C	4.065430	2.439924	-2.874414
C	4.547409	3.280018	-0.642726
H	5.189609	3.338857	0.232093
C	6.091244	1.681438	-1.994131
C	5.966873	1.098664	-3.270496
C	7.148290	1.336941	-1.151529
C	8.075095	0.389379	-1.590694
C	7.948318	-0.199599	-2.853839
C	6.895175	0.160079	-3.704117
H	2.146394	2.950426	-3.705370
H	2.995178	4.479717	0.225975
H	7.224307	1.764182	-0.155213
H	6.796308	-0.310355	-4.679375
H	8.660263	-0.956884	-3.169481
H	8.893551	0.094265	-0.939576
C	4.722185	1.620696	-3.985670

C	5.132336	2.555390	-5.150687
H	5.773806	3.368183	-4.794827
H	5.680479	1.993332	-5.915800
H	4.243552	2.995513	-5.617652
C	3.826870	0.493050	-4.521921
H	2.930735	0.904210	-4.996499
H	4.363502	-0.097677	-5.273610
H	3.499375	-0.169466	-3.721440
C	-1.924193	3.540006	2.412159
C	-2.434582	2.576727	3.304653
C	-2.763172	4.069176	1.415398
C	-4.579492	2.692498	2.170922
C	-3.755760	2.170847	3.189419
C	-4.085058	3.650253	1.285375
H	-4.702603	4.038654	0.479766
C	-5.869404	1.998109	2.211278
C	-5.845243	1.076025	3.275522
C	-6.965011	2.064289	1.350022
C	-8.033284	1.188459	1.553388
C	-8.008126	0.266158	2.605715
C	-6.913474	0.210694	3.477954
H	-1.790702	2.158645	4.072457
H	-2.357045	4.786648	0.711182
H	-6.970036	2.756695	0.512543
H	-6.896959	-0.512831	4.289714
H	-8.840182	-0.419347	2.741290
H	-8.884819	1.211531	0.878770
C	-4.535343	1.178639	4.052252
C	-4.787920	1.779838	5.456031
H	-5.294868	2.747244	5.382566
H	-5.414470	1.106835	6.052919
H	-3.839163	1.926507	5.985206
C	-3.831740	-0.180069	4.191714

H	-2.891746	-0.070142	4.739515
H	-4.460281	-0.884292	4.748086
H	-3.605140	-0.614862	3.216457
C	-0.537855	-1.590016	3.563435
F	0.564276	-2.275207	3.864918
F	-0.519230	-0.418110	4.199368
F	-1.612190	-2.288634	3.943569
C	4.776060	-0.220677	1.216227
C	5.706771	-1.227666	1.577803
C	4.258696	-0.243174	-0.093336
C	5.625754	-2.135037	-0.667864
C	6.137240	-2.152482	0.643624
C	4.662902	-1.198689	-1.022474
H	4.234890	-1.187144	-2.016157
C	6.276856	-3.191189	-1.440046
C	7.180628	-3.866999	-0.597888
C	6.143255	-3.548483	-2.781799
C	6.924185	-4.596364	-3.274961
C	7.815805	-5.277575	-2.437393
C	7.948512	-4.914164	-1.091439
H	6.076543	-1.294663	2.596437
H	3.551763	0.500729	-0.415212
H	5.438882	-3.026113	-3.421768
H	8.644065	-5.445659	-0.446030
H	8.411016	-6.094942	-2.836140
H	6.834134	-4.891103	-4.317358
C	7.161896	-3.272813	0.809239
C	6.705736	-4.312419	1.853509
H	5.735155	-4.737741	1.584157
H	7.432870	-5.130235	1.922651
H	6.621044	-3.849094	2.844139
C	8.544739	-2.702146	1.188440
H	8.505881	-2.220210	2.172682

H	9.294841	-3.500432	1.230120
H	8.871624	-1.958886	0.454004
C	-4.655873	0.794427	-1.778666
C	-5.451956	-0.109542	-2.517410
C	-4.416524	0.526074	-0.417707
C	-5.757363	-1.460281	-0.531267
C	-5.999307	-1.219267	-1.896516
C	-4.968012	-0.586366	0.208361
H	-4.775429	-0.760717	1.260871
C	-6.369263	-2.735803	-0.164853
C	-6.994329	-3.276229	-1.306282
C	-6.345784	-3.435388	1.042620
C	-6.943916	-4.697350	1.097398
C	-7.563803	-5.239096	-0.035973
C	-7.598867	-4.525104	-1.241756
H	-5.609070	0.050455	-3.580466
H	-3.803044	1.194248	0.164704
H	-5.861783	-3.008243	1.916869
H	-8.076668	-4.956046	-2.118233
H	-8.021993	-6.222969	0.020440
H	-6.929245	-5.262569	2.026140
C	-6.876239	-2.318234	-2.492395
C	-6.230378	-2.969279	-3.729949
H	-5.222947	-3.326878	-3.511968
H	-6.830185	-3.818260	-4.077295
H	-6.169991	-2.243098	-4.549197
C	-8.269714	-1.754894	-2.859146
H	-8.180648	-1.003159	-3.652094
H	-8.925539	-2.556165	-3.219237
H	-8.743327	-1.285233	-1.990691
C	3.527173	-6.202478	-2.873316
H	3.740150	-7.244685	-3.132280
H	2.582516	-5.908355	-3.346151

H	4.307704	-5.571189	-3.309984
C	2.637980	-3.201319	1.103564
H	2.124369	-3.308201	2.062915
H	3.535358	-2.602406	1.272223
H	1.978707	-2.612929	0.462807
C	-2.597160	-2.989534	-0.817171
H	-2.090965	-2.129304	-1.261159
H	-3.444047	-2.607457	-0.243977
C	-3.047332	-3.976908	-1.870265
C	-4.049975	-4.922175	-1.598917
C	-2.417253	-4.015939	-3.124455
C	-4.409340	-5.879272	-2.548904
H	-4.579205	-4.890215	-0.651612
C	-2.782197	-4.967994	-4.076524
H	-1.638131	-3.294429	-3.347001
C	-3.776327	-5.908169	-3.792041
H	-5.200065	-6.587810	-2.317226
H	-2.286615	-4.975116	-5.044071
H	-4.058629	-6.649375	-4.535027
C	-1.879276	-7.099067	1.853482
H	-1.576601	-7.930237	2.502174
H	-2.852958	-7.350096	1.415298
H	-1.151198	-7.036095	1.040027
C	-0.549038	-5.469566	3.210603
H	0.207534	-5.483057	2.418525
H	-0.506145	-4.488808	3.684325
H	-0.252029	-6.218401	3.954836
C	-3.303801	-4.863310	4.734456
C	-4.386831	-4.102178	4.532439
H	-5.063194	-4.285147	3.701225
H	-4.640026	-3.275298	5.191243
C	-2.397934	-4.631482	5.920650
H	-2.349317	-5.531818	6.547941

H	-1.370356	-4.408485	5.616607
H	-2.752610	-3.802296	6.541161

A1’

O	-1.187286	-1.829476	2.655344
O	-2.905118	-0.830459	1.072637
O	-0.792318	-2.350350	-2.437432
O	1.349905	-2.494635	-1.061169
N	-0.840618	0.565655	1.796853
S	-0.517160	0.863241	-2.558373
S	0.569986	0.862972	2.360239
O	-1.918927	0.627146	-2.175877
O	-0.002375	2.241290	-2.461219
O	1.683769	-0.008164	1.995636
O	0.845084	2.378060	2.222408
N	0.529827	-0.159383	-1.928072
P	0.058726	-1.559831	-1.297064
N	-0.684980	-1.598999	0.084461
P	-1.323419	-0.920688	1.314960
Si	0.698515	3.575029	0.871291
C	1.993732	3.079212	-0.372998
H	1.656388	2.071569	-0.653796
H	1.774734	3.681110	-1.264409
C	-1.038991	3.352643	0.249108
H	-1.231686	3.997023	-0.611389
H	-1.239416	2.332013	-0.072558
H	-1.761111	3.590922	1.035797
O	-0.965000	4.824157	-2.839593
H	-0.745433	3.874790	-2.932560
C	3.528552	3.028215	-0.152577
C	0.196148	5.542505	-2.681646
C	4.116808	4.470612	-0.208750
H	3.628683	5.074079	0.564506

H	3.829247	4.906374	-1.170790
C	1.333533	7.357139	-1.573230
C	2.496951	7.074457	-2.289073
C	2.491347	6.039047	-3.221356
C	1.348787	5.259419	-3.441149
C	0.166149	6.609504	-1.761358
H	1.320424	8.179748	-0.860804
H	3.398341	7.658332	-2.125972
H	3.389039	5.824587	-3.797217
C	-3.800102	-0.721218	2.141340
C	-1.833049	-3.074048	2.608572
C	-1.125767	-3.679506	-2.157112
C	1.965157	-3.220550	-2.081048
C	-4.530146	0.498013	2.262924
C	3.337002	-2.933152	-2.361915
C	-1.026033	-4.227459	2.410851
C	-2.474299	-3.970127	-1.807149
C	-5.817839	-2.523904	6.173014
C	-4.956125	-2.688677	5.111268
C	-4.829922	-1.687547	4.109869
C	-5.591923	-0.483571	4.252309
C	-6.485648	-0.353397	5.350757
C	-6.601296	-1.350929	6.290369
H	-5.893710	-3.300008	6.929504
H	-4.358130	-3.589633	5.037693
C	-3.957899	-1.817017	2.976647
C	-5.437301	0.559152	3.308547
H	-7.068167	0.560432	5.439736
H	-6.059717	1.442586	3.414823
C	-5.134298	-6.834659	2.025975
C	-3.761412	-6.758012	2.069733
C	-3.103923	-5.516986	2.290017
C	-3.888978	-4.338873	2.513870

C	-5.305627	-4.452182	2.450918
C	-5.910724	-5.665118	2.206634
H	-1.113978	-6.337408	2.109699
H	-3.150694	-7.643470	1.916838
C	-1.690291	-5.430436	2.268021
C	-3.212667	-3.086940	2.715880
H	-5.913333	-3.564508	2.581268
H	-6.993995	-5.723348	2.150146
C	0.267538	-8.216103	-1.155258
C	0.584532	-6.966665	-1.641012
C	-0.408374	-5.956003	-1.770649
C	-1.746966	-6.266374	-1.364397
C	-2.045853	-7.574499	-0.895290
C	-1.062549	-8.530759	-0.787920
H	1.046851	-8.965971	-1.052464
H	1.608633	-6.740029	-1.912987
C	-0.115266	-4.620858	-2.216579
C	-2.750289	-5.268531	-1.424011
H	-3.069363	-7.794946	-0.605177
H	-3.766478	-5.533366	-1.146088
C	3.125188	-6.261582	-5.943795
C	3.830842	-5.349761	-5.194218
C	3.211289	-4.651035	-4.121802
C	1.845588	-4.928613	-3.801550
C	1.139543	-5.863092	-4.607292
C	1.762424	-6.509497	-5.651922
H	4.978739	-3.565519	-3.574711
H	4.873370	-5.137269	-5.418724
H	0.093694	-6.057916	-4.399752
H	1.200926	-7.212958	-6.260422
C	3.922377	-3.694113	-3.362538
C	1.248156	-4.239347	-2.693019
H	-7.282666	-1.237068	7.128858

H	-5.624723	-7.787363	1.846320
H	-1.301490	-9.524161	-0.418186
H	3.606011	-6.783683	-6.766333
C	-0.403706	0.548377	-4.401403
F	-0.920974	-0.641623	-4.728951
F	-1.084571	1.512632	-5.041289
F	0.872427	0.591625	-4.792318
C	-3.542994	-2.941694	-1.840590
C	-3.716602	-2.092214	-2.951585
C	-4.431184	-2.848292	-0.756411
C	-5.598660	-1.047498	-1.828108
C	-4.753305	-1.171692	-2.950512
C	-5.450019	-1.899038	-0.734215
H	-6.090247	-1.806496	0.139245
C	-6.487684	0.097919	-2.037257
C	-6.228036	0.634943	-3.313242
C	-7.395727	0.712325	-1.174493
C	-8.040273	1.876919	-1.596803
C	-7.783376	2.413086	-2.863628
C	-6.877995	1.789376	-3.731649
H	-3.030653	-2.152653	-3.790363
H	-4.286713	-3.498299	0.097316
H	-7.571425	0.312595	-0.179082
H	-6.676453	2.216643	-4.710990
H	-8.282319	3.327682	-3.172528
H	-8.736564	2.379728	-0.930934
C	-5.186371	-0.202203	-4.051429
C	-5.863814	-0.997434	-5.196475
H	-6.689260	-1.607892	-4.816126
H	-6.262006	-0.311566	-5.953247
H	-5.137697	-1.660719	-5.680534
C	-4.041011	0.644296	-4.625710
H	-3.302526	0.006475	-5.119249

H	-4.425048	1.349506	-5.372068
H	-3.526677	1.202046	-3.842947
C	0.454393	-4.154518	2.323248
C	1.226927	-3.467765	3.281736
C	1.100116	-4.822857	1.270177
C	3.241866	-4.113054	2.087260
C	2.609922	-3.465711	3.169999
C	2.486402	-4.799289	1.137470
H	2.960104	-5.286328	0.289234
C	4.682370	-3.855407	2.162386
C	4.936277	-3.080850	3.310508
C	5.709518	-4.170124	1.271968
C	6.993197	-3.685116	1.528839
C	7.246425	-2.906103	2.663638
C	6.217837	-2.607785	3.566514
H	0.735731	-2.944957	4.096607
H	0.500255	-5.333041	0.527627
H	5.505406	-4.742828	0.371259
H	6.420522	-1.997606	4.443565
H	8.245857	-2.517661	2.838382
H	7.799128	-3.897073	0.831454
C	3.653790	-2.856019	4.108057
C	3.699280	-3.660301	5.430641
H	3.877896	-4.722887	5.237300
H	4.502482	-3.287676	6.076932
H	2.749914	-3.562428	5.969975
C	3.410652	-1.370109	4.418150
H	2.471944	-1.242522	4.964396
H	4.217625	-0.974839	5.046191
H	3.353734	-0.774506	3.505546
C	0.450058	0.879634	4.234549
F	0.193409	-0.354295	4.669308
F	1.610810	1.302069	4.743101

F	-0.527433	1.699779	4.610623
C	-4.376205	1.649501	1.336135
C	-4.538886	2.961828	1.835663
C	-4.155238	1.476763	-0.043263
C	-4.350263	3.842201	-0.416169
C	-4.531316	4.040051	0.966626
C	-4.146127	2.560765	-0.917371
H	-3.983574	2.390334	-1.974867
C	-4.407215	5.141700	-1.087474
C	-4.625807	6.137514	-0.114705
C	-4.291430	5.473952	-2.437408
C	-4.418269	6.813926	-2.809890
C	-4.648147	7.804045	-1.846838
C	-4.746422	7.469696	-0.489837
H	-4.657866	3.123296	2.904101
H	-4.002718	0.489138	-0.449514
H	-4.096323	4.707437	-3.181226
H	-4.915646	8.244278	0.254675
H	-4.742117	8.842380	-2.153884
H	-4.329043	7.091846	-3.856565
C	-4.696005	5.525028	1.283296
C	-3.543036	6.038460	2.173436
H	-2.572746	5.854215	1.701842
H	-3.638467	7.116888	2.344945
H	-3.554626	5.535168	3.147511
C	-6.053505	5.809248	1.957961
H	-6.110790	5.311953	2.933536
H	-6.189182	6.885326	2.117119
H	-6.879344	5.446241	1.337759
C	4.163500	-1.952799	-1.607287
C	5.284008	-1.365502	-2.240569
C	3.954084	-1.684904	-0.239751
C	5.981064	-0.394820	-0.132871

C	6.183626	-0.610642	-1.509253
C	4.853997	-0.915347	0.494019
H	4.681532	-0.754620	1.551864
C	7.069625	0.440599	0.375098
C	7.933298	0.748255	-0.695085
C	7.316782	0.936183	1.656619
C	8.424516	1.763972	1.854383
C	9.274429	2.083306	0.788962
C	9.035205	1.569102	-0.492064
H	5.437639	-1.502037	-3.307279
H	3.103011	-2.108667	0.271402
H	6.654988	0.685634	2.481175
H	9.702008	1.816749	-1.314669
H	10.128507	2.733880	0.957720
H	8.625614	2.168124	2.842958
C	7.475704	0.056388	-1.977912
C	7.251634	1.048907	-3.135049
H	6.547488	1.835232	-2.853793
H	8.198341	1.522363	-3.420460
H	6.855794	0.528867	-4.015180
C	8.503764	-1.018754	-2.402199
H	8.144143	-1.566713	-3.281202
H	9.464179	-0.555516	-2.656546
H	8.670729	-1.737594	-1.593170
C	-1.108830	6.949981	-1.031898
H	-0.937316	7.734949	-0.287606
H	-1.881713	7.299964	-1.723490
H	-1.539919	6.082113	-0.526538
C	1.361265	4.150057	-4.466867
H	1.609368	3.182929	-4.016804
H	0.382383	4.034264	-4.944644
H	2.099538	4.358906	-5.247899
C	0.757116	5.187372	1.854504

H	-0.139852	5.147486	2.489951
H	0.558597	5.967656	1.107527
C	1.964116	5.551378	2.683512
C	2.359548	4.768856	3.780254
C	2.706691	6.703242	2.388067
C	3.466285	5.126417	4.550342
H	1.802337	3.870452	4.027561
C	3.809859	7.066383	3.162078
H	2.419863	7.314078	1.535116
C	4.195909	6.277950	4.246827
H	3.758182	4.502760	5.391542
H	4.369710	7.964192	2.912906
H	5.057560	6.555660	4.847680
C	3.880211	2.339602	1.174260
H	4.959678	2.186709	1.248894
H	3.559748	2.935362	2.036657
H	3.403347	1.360707	1.236347
C	4.092555	2.190895	-1.316338
H	3.676734	1.178245	-1.308794
H	3.847456	2.650993	-2.282591
H	5.177187	2.110859	-1.237976
C	5.623165	4.574683	-0.040565
C	6.452113	4.490048	-1.088079
H	6.078458	4.353385	-2.100250
H	7.531904	4.538729	-0.970712
C	6.150343	4.823933	1.352334
H	5.843487	5.818329	1.700905
H	5.742091	4.113796	2.078422
H	7.242669	4.760449	1.388898

A2

O	1.189184391761	2.701614856787	-2.763234322084
O	-0.223549604336	1.745468088358	-0.896382131997

O	2.543514127776	-1.315153018598	-0.040166599954
O	3.570907844943	-1.277611293670	-2.378427940347
N	-0.582543224937	0.940465261836	-3.382385893641
S	-0.070601834263	-2.938664066961	-1.442118138039
S	-0.926661556033	1.607754867820	-4.729260298747
O	-0.748853485401	-1.936200714076	-0.602208393169
O	-0.869409667539	-3.626191230108	-2.467790222368
O	0.119076518558	2.154421923875	-5.587162770920
O	-1.873122615804	0.640987936519	-5.481466494804
N	1.324643634935	-2.495943592877	-2.042450952728
P	2.147734946704	-1.193086913847	-1.618728709547
N	1.578763273649	0.234855473211	-1.930073910029
P	0.532485261371	1.314458519699	-2.255294771524
Si	-2.412880497889	-1.029945306389	-5.039345062056
C	-3.658786562953	-1.403818267999	-6.381896251103
H	-3.079569668593	-1.561796727937	-7.297322927317
H	-4.018019325667	-2.400391039736	-6.085823410296
C	-3.065355741850	-0.965043643367	-3.294780182461
H	-2.295188854675	-1.205859504762	-2.560657139325
H	-3.458888368576	0.023364837232	-3.044508257851
H	-3.882694925889	-1.685782294339	-3.193279007063
O	-2.817696281921	-4.821595114902	-4.036699847882
H	-2.261504018767	-4.512937174600	-3.297305758351
C	-4.889919573277	-0.524737438469	-6.710218782174
C	-4.132877922024	-4.495941973450	-3.842856077148
C	-5.778331805755	-1.298299867075	-7.744462983686
H	-6.715577265967	-0.736070188508	-7.862469148641
H	-6.047043059717	-2.258454380053	-7.286033698372
C	-6.297822671323	-4.213542273583	-4.861346699867
C	-6.814571674228	-3.763963057346	-3.643480913345
C	-5.993050098889	-3.733629829981	-2.517882234925
C	-4.644386205966	-4.110364100860	-2.589499402017
C	-4.960095998160	-4.597705599855	-4.978723356207

H	-6.939867393597	-4.274036893811	-5.737478477886
H	-7.855687398416	-3.463506206204	-3.565122792305
H	-6.399227342648	-3.424246343288	-1.559403760874
C	-0.957729381676	2.939948808335	-0.853527522837
C	1.944367622078	3.448959732964	-1.848080448917
C	3.476756644924	-0.384482751627	0.438256162980
C	4.560429202822	-2.130095409047	-1.862665477893
C	-2.379092152848	2.852535967323	-0.728811437346
C	4.857679008121	-3.317054007184	-2.581742004974
C	3.365845247794	3.441211792178	-1.968171591593
C	3.010362312206	0.654656122247	1.293739703663
C	-1.140255694605	7.778622436892	-1.271963279262
C	-0.384678786544	6.628997823827	-1.209303865107
C	-0.995101941423	5.365708041695	-0.978940506970
C	-2.418266929043	5.312869832283	-0.853235208069
C	-3.167860960298	6.520735197737	-0.899585769291
C	-2.544944571163	7.729547319611	-1.102129515650
H	-0.653701276718	8.731912006485	-1.458235331013
H	0.688398793756	6.682594113541	-1.349685225994
C	-0.256229313747	4.137837035284	-0.899146020702
C	-3.055647076774	4.063359255420	-0.690186508352
H	-4.248002916432	6.466183576192	-0.787145043565
H	-4.127908749865	4.062813486951	-0.525480367396
C	3.526238371564	6.269557445892	2.016480580166
C	4.147330941899	5.667858462719	0.946710061090
C	3.398306512855	4.941835623656	-0.020633660296
C	1.973505299637	4.883419034669	0.100311879283
C	1.363854125732	5.506197597077	1.223922347915
C	2.121039075097	6.174086931739	2.160899851950
H	5.125421437057	4.320942267991	-1.131483538456
H	5.226930922144	5.719255900198	0.831520308970
C	4.045392516810	4.246022535082	-1.067431200012
C	1.236273913731	4.157746893560	-0.895190471846

H	0.289081639380	5.439865158724	1.347233659889
H	1.635369205156	6.630670600666	3.018582280575
C	7.905807358855	1.577206905942	0.204970638293
C	7.067390985134	0.522954339410	-0.082092469545
C	5.721980829103	0.512462839199	0.377430534369
C	5.256874463840	1.623292792387	1.152409160248
C	6.152169659618	2.688777001101	1.443440201161
C	7.446695893897	2.672560915859	0.976924229391
H	8.925885118499	1.571827543192	-0.168837035842
H	7.422633832380	-0.303153450144	-0.686918815942
C	4.796339676499	-0.530754565287	0.045583799041
C	3.921312935599	1.641380050727	1.623396794356
H	5.783810032464	3.524921143304	2.031610519520
H	3.609712623092	2.456349980579	2.270382703818
C	8.138314811031	-4.458067749945	0.847460115970
C	7.530920798876	-4.709678398677	-0.360096063279
C	6.539587843129	-3.831378314896	-0.876154411492
C	6.191203053185	-2.651842293074	-0.142588540443
C	6.816483970452	-2.440538207063	1.118705307572
C	7.763999741906	-3.317426291335	1.597219405576
H	6.112171052745	-5.033683660321	-2.621523142946
H	7.789780962305	-5.594198082165	-0.936998895853
H	6.535984493480	-1.579931999273	1.713751823205
H	8.223971240071	-3.134734002869	2.564536874670
C	5.859443960069	-4.124548683544	-2.082565140854
C	5.203485899847	-1.763290203261	-0.689555395613
H	-3.127369201824	8.645591961976	-1.145378019915
H	4.110277515979	6.810452807814	2.755802447072
H	8.118996833415	3.497030178009	1.198157945197
H	8.891749828312	-5.138067343361	1.235123816398
C	0.410636591792	-4.324940898655	-0.282379457096
F	1.247055263765	-3.876352274478	0.661044066447
F	-0.690653375918	-4.818532276614	0.300135445171

F	1.017234230884	-5.314133571531	-0.955973507884
C	1.633998806434	0.666294080824	1.846855733872
C	1.042910827902	-0.511230071871	2.349952200612
C	0.925402673198	1.876782735209	1.931524101622
C	-0.952713181571	0.751425894057	2.916400823821
C	-0.228513391837	-0.458501726560	2.898418140894
C	-0.366761733582	1.927809126249	2.450066710673
H	-0.916788677758	2.865241911994	2.450711725316
C	-2.314267081111	0.471796148268	3.379594257819
C	-2.404357658358	-0.898874494085	3.694084136949
C	-3.443021745028	1.290127672159	3.426106756292
C	-4.670058430601	0.723526818254	3.778233835832
C	-4.762583413001	-0.637328662802	4.090294299438
C	-3.624666324508	-1.453948762273	4.058470771356
H	1.579675465402	-1.452536707213	2.293494248516
H	1.377251337816	2.779001985946	1.538623397186
H	-3.377218440219	2.339809417455	3.151914502155
H	-3.705782708917	-2.512718692464	4.293130510539
H	-5.727605404766	-1.068667622269	4.340770117249
H	-5.564397921491	1.340722191794	3.794883391447
C	-1.047578128394	-1.581555703777	3.533882152144
C	-0.465384578288	-1.926586761255	4.928006993446
H	-0.417499790121	-1.037130846550	5.564477494445
H	-1.091522107741	-2.674976064054	5.427631213242
H	0.546618076071	-2.335404494531	4.827842556518
C	-1.107261887141	-2.854969746832	2.676001384283
H	-0.119691982927	-3.320217579081	2.613818618986
H	-1.791257589120	-3.587045396169	3.121073219850
H	-1.431931313214	-2.642423979175	1.656531555434
C	4.099648486612	2.578835434945	-2.928272263982
C	3.554056921335	2.157965802621	-4.159506785892
C	5.386162050888	2.121828304874	-2.573897973898
C	5.556470335107	0.851783050598	-4.601671484893

C	4.265294876173	1.281049542622	-4.959414934445
C	6.122071316631	1.279413896534	-3.401344189488
H	7.107238565889	0.943539810131	-3.089597810881
C	6.058408878286	-0.036807271867	-5.651288820610
C	5.052802049124	-0.178252055079	-6.630314895832
C	7.282040751503	-0.696092919375	-5.784476662717
C	7.495951632172	-1.495079226862	-6.910824434061
C	6.503489345522	-1.625646952283	-7.889556238675
C	5.274812651240	-0.966802737470	-7.751652597587
H	2.579716397075	2.498836886178	-4.481310196078
H	5.808670853237	2.398889016563	-1.616805797240
H	8.053505147347	-0.592852521104	-5.025544222954
H	4.501672860313	-1.082368848102	-8.507471592460
H	6.687043486562	-2.247496625210	-8.761767925869
H	8.441119935185	-2.017918888358	-7.029843984443
C	3.795263679065	0.585360595204	-6.230349488439
C	3.312417718770	1.559967971174	-7.318906882512
H	4.095935095313	2.277584512896	-7.584341072474
H	3.027324666893	1.009899516961	-8.223442209596
H	2.431849880675	2.111126490427	-6.972319876137
C	2.661581758792	-0.405748289668	-5.863628601045
H	1.778459198525	0.155019327250	-5.542618779075
H	2.385302749469	-1.019914616445	-6.725639420122
H	2.967274385048	-1.071618036175	-5.053206679263
C	-2.160783833357	2.995914577634	-4.435469319836
F	-1.571137689180	3.962412234503	-3.735923579888
F	-2.572181030952	3.472892777122	-5.608627531636
F	-3.208224440204	2.518346934341	-3.757893574658
C	-3.144475517151	1.588032704506	-0.570087543293
C	-4.511048798889	1.556358796857	-0.943913421120
C	-2.599300876165	0.452869565277	0.056766734206
C	-4.759814862018	-0.596314083537	0.137557704130
C	-5.306855900511	0.483677357553	-0.581997971690

C	-3.397428832737	-0.633783836453	0.405987239676
H	-2.953869699234	-1.477922996905	0.915535525809
C	-5.835766863441	-1.525657732619	0.480443054883
C	-7.036735773604	-1.040707645614	-0.072277127097
C	-5.820161585850	-2.701229047895	1.233411172366
C	-7.013165796109	-3.409309872507	1.397836908881
C	-8.203011001620	-2.943163021171	0.825329520848
C	-8.220706800973	-1.749697326618	0.091773668500
H	-4.939073137858	2.378779373867	-1.509975684094
H	-1.553190483706	0.421257350703	0.314649113638
H	-4.897076801780	-3.051885840706	1.685621539020
H	-9.149863664829	-1.386858992754	-0.341377501913
H	-9.121478388879	-3.508778575802	0.956770149143
H	-7.018202764867	-4.331037333150	1.973437894375
C	-6.802249613865	0.273766421137	-0.813748497764
C	-7.122495791271	0.118828678022	-2.313866675965
H	-6.568396667688	-0.719982850900	-2.742291382560
H	-8.192781780292	-0.067838392534	-2.461898092528
H	-6.855998905869	1.028125193231	-2.864277463935
C	-7.630368732904	1.420710092828	-0.199535374397
H	-7.410419795566	2.367559144115	-0.706837519229
H	-8.703413363306	1.222443642762	-0.303025054984
H	-7.402142372952	1.539253688251	0.864864903509
C	4.129630901494	-3.755093213629	-3.810829719775
C	3.116048751046	-4.724097569234	-3.700947696226
C	4.547691360218	-3.322655136429	-5.077161290133
C	2.982046904089	-4.818612499898	-6.121493853292
C	2.554239380934	-5.256633595328	-4.852724126319
C	3.970830107083	-3.843842694719	-6.236676118831
H	4.311134880663	-3.500345324337	-7.207924230947
C	2.255318940820	-5.573550727660	-7.146924935406
C	1.384369156479	-6.473713571516	-6.502632767189
C	2.327691654708	-5.525628607191	-8.539200304332

C	1.521248192609	-6.388167406572	-9.285206237063
C	0.647373579051	-7.276323987979	-8.647285246669
C	0.573614872996	-7.320265393987	-7.248916388592
H	2.786569468890	-5.045197173189	-2.718209084968
H	5.341077168881	-2.588387136847	-5.156361283770
H	2.993866956747	-4.824931828863	-9.035606914510
H	-0.105869207346	-8.012261305256	-6.756875378368
H	0.022335518066	-7.938117092044	-9.241413812096
H	1.565135527058	-6.362685642175	-10.371007832784
C	1.508624394481	-6.362132813187	-4.985366270906
C	0.170475880611	-5.975817463149	-4.331602854006
H	-0.185204655330	-5.019388084220	-4.714191987332
H	-0.597549014023	-6.730537004423	-4.534019762992
H	0.281179622479	-5.876815741434	-3.249137428271
C	2.028980489239	-7.683126623528	-4.376361644966
H	2.182211081421	-7.569548244276	-3.296937119660
H	1.303862848923	-8.489953459386	-4.534271921415
H	2.979608056117	-7.980007226537	-4.831590173890
C	-4.386315248774	-5.127108284374	-6.269313175749
H	-5.133227191234	-5.108952578703	-7.069230543974
H	-3.516728742593	-4.546905184306	-6.596134892461
H	-4.035082538374	-6.159247883327	-6.147555018025
C	-3.775082792987	-4.099008400728	-1.353051721363
H	-4.396923043985	-4.196621277517	-0.460514862799
H	-3.051056510349	-4.920962052034	-1.349538974816
H	-3.201815243360	-3.170377369443	-1.257177254897
C	-0.849307532355	-2.052163824306	-5.301124598293
H	-0.969844654609	-2.981621890136	-4.741105114778
H	-0.008400019979	-1.527750927589	-4.829956808811
C	-0.599767885729	-2.326672814508	-6.764572601704
C	-0.186353007402	-1.307026168054	-7.637215267180
C	-0.868125198537	-3.593124496843	-7.302368430487
C	-0.040413678779	-1.550788591111	-9.003183943398

H	0.008512690283	-0.312898366094	-7.245447389338
C	-0.730614324237	-3.836461844643	-8.669262817573
H	-1.186211591538	-4.395567171296	-6.643742185334
C	-0.314744030172	-2.816605585245	-9.526056668123
H	0.281092458803	-0.745803324302	-9.659769132166
H	-0.928717153103	-4.830917986310	-9.055474885323
H	-0.202346093672	-3.006839505408	-10.590222154731
C	-5.744247083334	-0.299284759113	-5.451836529072
H	-6.660515686318	0.254640644122	-5.692447963794
H	-6.034036620797	-1.251730298531	-4.994385218333
H	-5.199040241900	0.283302509527	-4.701587871414
C	-4.458820424203	0.836347292273	-7.285273499733
H	-3.963098155516	1.451039367885	-6.529793213622
H	-3.759230836539	0.712147804267	-8.118708297969
H	-5.330980946478	1.394691274237	-7.647696287162
C	-5.174581829149	-1.540569139221	-9.117566868270
C	-4.392545835767	-2.596184538827	-9.373193318110
H	-4.155187916471	-3.334630449770	-8.613653440187
H	-3.947983064876	-2.754769335128	-10.352689679938
C	-5.524874478912	-0.545230731730	-10.199025259819
H	-6.609970713297	-0.534669993987	-10.374427155358
H	-5.246930539754	0.477234581032	-9.917908840227
H	-5.030886053434	-0.783782228238	-11.146525059200

A2’

O	0.963672977509	2.364159752829	-2.627781756429
O	-0.271958180873	1.918577059888	-0.458294314148
O	1.854337992559	-1.106814462813	0.553976156928
O	2.774619385638	-1.561919195789	-1.796792936527
N	-1.347165585480	1.235007468723	-2.701703517244
S	-0.512564498565	-3.135632171830	0.091108415696
S	-1.676301783138	1.233189293955	-4.198477293063
O	-1.055238624458	-1.995572075118	0.844821470629

O	-1.465361781734	-4.113084236744	-0.470062235848
O	-0.610260551514	1.141873041629	-5.193250534561
O	-2.863103335417	0.262062129020	-4.467354796739
N	0.595305461967	-2.757400576852	-0.993134381343
P	1.394713797893	-1.367190591709	-0.983153286031
N	0.738558207870	-0.118913994747	-1.670549753903
P	0.053031516271	1.243535575789	-1.881471546904
Si	-2.936799500822	-1.523908004660	-4.123834102120
C	-1.643918978588	-2.293517809869	-5.243627491339
H	-0.745409119126	-1.680859641089	-5.066249305889
H	-1.432507042233	-3.260314893999	-4.766188932572
C	-2.432881400046	-1.663673980675	-2.347423056666
H	-2.949225259851	-2.503725944677	-1.880074282938
H	-1.358780124076	-1.832635869024	-2.269897987174
H	-2.680222550906	-0.760254618045	-1.792325272320
O	-2.474025674208	-4.662492878911	-3.015692666677
H	-1.934674156306	-4.477604344700	-2.218354794695
C	-1.823412929649	-2.494616557267	-6.769903220328
C	-2.404056238391	-5.990954892297	-3.360812764797
C	-2.921374797807	-3.581505689349	-6.993024960541
H	-3.838268164130	-3.248336858796	-6.492968398100
H	-2.589871183652	-4.490528156173	-6.474727509661
C	-3.498158998252	-7.876172489170	-4.385224241407
C	-2.375371854915	-8.665371260108	-4.139290221215
C	-1.267328909259	-8.103351965688	-3.507537844387
C	-1.248408996321	-6.757362736104	-3.121621234136
C	-3.534233339218	-6.534280698315	-3.996693562894
H	-4.369093360215	-8.305409277361	-4.875559209166
H	-2.363496476993	-9.710210360161	-4.437271823727
H	-0.387907224026	-8.712807233010	-3.314870843503
C	-0.788893678270	3.221592650170	-0.454498193323
C	1.941832218043	3.052845516123	-1.894166467680
C	2.952999635323	-0.315182376970	0.871354704189

C	3.735660119430	-2.448378483515	-1.299204385167
C	-2.175967847554	3.383234165578	-0.167340198298
C	3.993337602326	-3.650704151159	-2.027020565412
C	3.303690251574	2.756289585529	-2.183611047726
C	2.711317900563	0.842099804946	1.665700088668
C	-0.261104270558	7.971899905953	-1.292180546661
C	0.295844999722	6.712891700029	-1.250932445235
C	-0.459913036743	5.592127231325	-0.811326680488
C	-1.833449888914	5.795896562948	-0.458392835621
C	-2.370899099861	7.111984288413	-0.491310651093
C	-1.603249272601	8.179682343843	-0.894111084664
H	0.334434163965	8.812261690607	-1.637743987838
H	1.323363386693	6.567996344419	-1.563651556289
C	0.080799485237	4.262939069245	-0.732021540512
C	-2.645272954564	4.683882966165	-0.127817554288
H	-3.410706775196	7.254381262159	-0.207011308272
H	-3.686534202989	4.860170414407	0.127497323214
C	4.604148888858	5.893054836849	1.324794660436
C	4.930043741383	5.040529702832	0.296131160071
C	3.920206369925	4.377509535967	-0.453121781640
C	2.543075757658	4.643712845970	-0.167558829193
C	2.242496710212	5.513101618176	0.918990691822
C	3.244221847895	6.114771650522	1.647931191036
H	5.310029470313	3.260089818913	-1.665519191428
H	5.968733566879	4.835634189876	0.053209414641
C	4.259775484441	3.445731351470	-1.461128871236
C	1.532295077907	3.988054222005	-0.954092601528
H	1.207800345843	5.694838770753	1.183081609206
H	2.986175466318	6.766556546087	2.477811882993
C	7.637200986086	0.831109433296	0.355802951557
C	6.613751566761	-0.068215379694	0.152354832910
C	5.312258124948	0.172114099782	0.674065278956
C	5.085578971715	1.384643269209	1.401452201464

C	6.171751590231	2.273932360729	1.622804851360
C	7.420694668993	2.009051947475	1.110000784105
H	8.619331359748	0.632112304575	-0.063865175345
H	6.792579158096	-0.967445747589	-0.425943738766
C	4.210208229537	-0.724975428860	0.462282436318
C	3.793290842041	1.668053363644	1.907281249368
H	5.986156061478	3.181294399794	2.189717566553
H	3.655771004118	2.556717547272	2.516468950484
C	7.094451917621	-4.926492518554	1.537778605578
C	6.495307329972	-5.169983014602	0.324161551756
C	5.585002960432	-4.235315872218	-0.241330323750
C	5.319441292759	-3.011060571109	0.449232813808
C	5.940203469715	-2.799020760802	1.710224324999
C	6.801960500479	-3.733453191939	2.240771777073
H	5.232311382199	-5.391866117718	-2.013075840797
H	6.697246499481	-6.090762460791	-0.217815405402
H	5.720033873502	-1.894576749311	2.264963609998
H	7.257572950696	-3.554678666318	3.210741161557
C	4.945984105048	-4.493597185336	-1.475859017364
C	4.417265263448	-2.070182308313	-0.154139286916
H	-2.027584062493	9.179376759498	-0.922624640216
H	5.384797534555	6.387335999295	1.896405884896
H	8.240413775467	2.702152762364	1.278570743714
H	7.781578667459	-5.651675839354	1.964751922331
C	0.420315980226	-4.157842116206	1.354908265555
F	1.353784486863	-3.422624525854	1.973894598182
F	-0.445852644478	-4.606601198055	2.272636571113
F	1.013615302027	-5.199229649561	0.763631920482
C	1.390515630674	1.118951677154	2.285638833369
C	0.669496441985	0.105833121949	2.951077994208
C	0.897944097008	2.434292498707	2.300829912317
C	-0.996157812771	1.750167729608	3.604017461862
C	-0.496292788739	0.431491863122	3.628979790111

C	-0.299743001943	2.755750665702	2.934120844370
H	-0.680448342859	3.773194995543	2.899938943989
C	-2.250235959356	1.791331289585	4.358316810748
C	-2.485145319816	0.512391606429	4.899615456035
C	-3.153322289068	2.834197315770	4.569610395372
C	-4.296604222525	2.587766157195	5.333046942681
C	-4.529424597018	1.318835648868	5.876232920033
C	-3.622122549923	0.273906481283	5.661636634522
H	1.032659511864	-0.916377868005	2.940854911308
H	1.451753583535	3.205035837833	1.780676606572
H	-2.975179827432	3.817158532958	4.140619330643
H	-3.811719233905	-0.711039680219	6.081452177629
H	-5.425122045771	1.141922521301	6.465840095211
H	-5.013556392273	3.386456734022	5.504492671408
C	-1.364536271737	-0.453676333876	4.524267687562
C	-0.561696198570	-0.869299795279	5.780389813033
H	-0.167881739983	0.007603004700	6.304643057558
H	-1.200299448826	-1.428235873501	6.474118687159
H	0.280872197882	-1.510783209197	5.497779441793
C	-1.906650039705	-1.708656573897	3.818726721444
H	-1.093557395197	-2.359629277057	3.487946296547
H	-2.544360864323	-2.278162349687	4.505296884636
H	-2.494572056318	-1.442139259955	2.939694790148
C	3.719090651267	1.760561383590	-3.203869874462
C	3.171169191257	1.746694318252	-4.501964314898
C	4.742503991120	0.851726526492	-2.879573172485
C	4.679942380855	-0.064710013331	-5.096374402445
C	3.664256360162	0.851209517371	-5.439782863913
C	5.220863279014	-0.066433105936	-3.810938336660
H	5.986793742653	-0.783179547234	-3.526507882230
C	4.935291574503	-0.934835382891	-6.246993010462
C	4.090961790756	-0.536531936645	-7.301229083326
C	5.766839877332	-2.047286682791	-6.380484389184

C	5.738594302526	-2.767905564501	-7.575934825040
C	4.898322625454	-2.375926987405	-8.624330037162
C	4.073366033229	-1.251529032956	-8.493072929864
H	2.373307127738	2.435108932194	-4.761495712198
H	5.145409388310	0.850495873056	-1.874023091555
H	6.397738468873	-2.369334286998	-5.556363615336
H	3.416642546117	-0.956282550031	-9.308148034299
H	4.873377871574	-2.959267303703	-9.540290272120
H	6.361158094547	-3.651154529737	-7.688450833228
C	3.255082064599	0.676022617009	-6.901390669437
C	3.659141834341	1.914895220977	-7.733618918038
H	4.732115382023	2.113943880958	-7.645712146164
H	3.423518123125	1.757147586141	-8.792503631194
H	3.113318043714	2.800847916475	-7.389549941945
C	1.746380543134	0.414610865098	-7.055110414578
H	1.169423387679	1.303261433107	-6.789364756301
H	1.503291546054	0.145683855806	-8.089556759931
H	1.414977333243	-0.394813873629	-6.399159853051
C	-2.607762947937	2.825293822985	-4.509289424805
F	-1.744117991111	3.829935085329	-4.373648348366
F	-3.103723635281	2.811464818421	-5.746837326355
F	-3.598412855416	2.962134468737	-3.631355186368
C	-3.099094038526	2.226553167174	-0.056999135019
C	-4.338802656638	2.305097411512	-0.726604679603
C	-2.745947150777	1.025598969490	0.587706241444
C	-4.743170172049	-0.029461632675	-0.236437886493
C	-5.152676256771	1.187717876295	-0.811581357254
C	-3.562651914831	-0.099896680217	0.501886957371
H	-3.226986654376	-1.035281268912	0.932762076126
C	-5.662296835606	-1.083269508768	-0.673045475313
C	-6.668387692191	-0.496422814743	-1.468892741683
C	-5.598533301901	-2.468321453180	-0.506528711918
C	-6.561173759977	-3.262881007546	-1.135877353864

C	-7.571436467816	-2.680841524724	-1.911301618549
C	-7.631446703845	-1.291185610025	-2.079339190642
H	-4.607601622441	3.216601054543	-1.251677072518
H	-1.808348377749	0.950676314230	1.121484126489
H	-4.798706764770	-2.921506473323	0.073248286541
H	-8.401802416155	-0.849282016230	-2.706350080718
H	-8.306975701181	-3.313579783323	-2.400573896772
H	-6.518695173071	-4.343498873812	-1.029240796150
C	-6.467915036829	1.015961343073	-1.569042822716
C	-6.356718557209	1.487106451430	-3.032279831466
H	-5.506410822454	1.020398724635	-3.534073401638
H	-7.263816551238	1.237599310979	-3.593198050073
H	-6.216557742956	2.572841511300	-3.077686265257
C	-7.614660853208	1.769933341710	-0.858370146808
H	-7.425210855946	2.849710801965	-0.865473180181
H	-8.567940642178	1.585537282262	-1.367312511261
H	-7.710777324650	1.444128169043	0.182237903510
C	3.387136515229	-3.982732691261	-3.344182031005
C	3.306161558292	-5.334056795662	-3.754562768165
C	3.027322533867	-2.983271363563	-4.270287343139
C	2.665717464955	-4.631911377990	-5.984870987451
C	2.967482239930	-5.649921910622	-5.060109563416
C	2.664828388266	-3.301093428228	-5.577184895756
H	2.446630186908	-2.508544991092	-6.282319070114
C	2.420416032238	-5.240957703720	-7.293520763139
C	2.554486440242	-6.636892119789	-7.166038494991
C	2.116531887680	-4.663581409529	-8.528044610576
C	1.923025509915	-5.498873580179	-9.630260972602
C	2.041476572118	-6.888093936561	-9.501709375142
C	2.364053595777	-7.463742534644	-8.266539819052
H	3.522105588765	-6.129478824356	-3.046948050614
H	3.080031322438	-1.942502209945	-3.986623733656
H	2.033116816726	-3.586208552962	-8.625711209086

H	2.461241532703	-8.542900394247	-8.173520801350
H	1.884527823040	-7.524694045561	-10.368462034212
H	1.676222212639	-5.066638101711	-10.596416828096
C	2.922337920980	-7.020634351857	-5.734765175506
C	1.855498759093	-7.940108616448	-5.112243647900
H	0.869145018425	-7.470304567229	-5.129181527479
H	1.794454961725	-8.886049511190	-5.663114492395
H	2.107445826041	-8.172467587221	-4.070933532551
C	4.307203016189	-7.701977343961	-5.684699541534
H	4.592488469973	-7.916263143637	-4.647910196600
H	4.290740195137	-8.649507977825	-6.235566860284
H	5.075274115455	-7.059420703742	-6.127394699542
C	-4.753649300468	-5.677116958353	-4.224476291042
H	-5.598910480507	-6.280382104503	-4.571799136494
H	-5.048908365004	-5.158069732356	-3.304820709350
H	-4.562615797841	-4.905294013314	-4.978886260657
C	-0.032263685441	-6.147918209690	-2.465789098780
H	-0.177062622482	-5.995519760046	-1.391917911623
H	0.840367877689	-6.792218515294	-2.596706829164
H	0.216203404119	-5.168184919145	-2.890257814350
C	-4.786668177233	-1.816898987780	-4.229741476081
H	-5.169672046376	-1.227918595652	-3.386776874307
H	-4.910464185618	-2.855865676424	-3.903784432766
C	-5.631274162135	-1.545044142514	-5.449380238409
C	-5.574274705970	-0.326038173645	-6.144791037721
C	-6.559384130640	-2.505762285734	-5.877828489303
C	-6.409539038470	-0.085317520507	-7.236588093142
H	-4.870372241292	0.440105184492	-5.835667776111
C	-7.398690814095	-2.265258929540	-6.966752190414
H	-6.627258463109	-3.450196740059	-5.343861732399
C	-7.325240128942	-1.053021701287	-7.654343543399
H	-6.342697275318	0.863754744882	-7.762427809297
H	-8.108244502134	-3.027746052243	-7.278103612130

H	-7.973656157994	-0.863999061096	-8.505494016786
C	-2.179614214095	-1.163531166633	-7.455477215510
H	-2.156452449954	-1.271475699191	-8.545363058549
H	-3.178970997458	-0.815399749511	-7.175569530556
H	-1.460369759608	-0.385050109897	-7.177285891778
C	-0.482403591442	-2.992094734384	-7.332805298215
H	0.293519842443	-2.231159334331	-7.198560883167
H	-0.147717367012	-3.903196153645	-6.825509965879
H	-0.560174001944	-3.212481633370	-8.401018702243
C	-3.259965643340	-3.919367309689	-8.433901258788
C	-2.590998107941	-4.861287179684	-9.109526295553
H	-1.777610223382	-5.423346010834	-8.657160296721
H	-2.834521541731	-5.105357775445	-10.141328975650
C	-4.422785148630	-3.177768250289	-9.048122033902
H	-5.350574305325	-3.411004588531	-8.510683870236
H	-4.303271044440	-2.091305833946	-8.971658814393
H	-4.560690106817	-3.437548231385	-10.103097892889

TS

O	1.220243030884	2.674779672633	-2.953612180757
O	-0.129575862146	1.810672886514	-0.993069694593
O	2.329679398957	-1.154111846444	-0.019509169032
O	3.553455617307	-1.236123273058	-2.260599595728
N	-0.734362319713	1.029772721600	-3.430802644132
S	0.000061464475	-3.098226102223	-1.457727707876
S	-1.184506852674	1.598372916925	-4.818552980252
O	-0.860114509584	-2.149973330659	-0.728432059281
O	-0.650429346150	-3.959122186201	-2.482906804447
O	-0.146128946217	2.169305974511	-5.683690997695
O	-2.110933843519	0.606242312126	-5.439567238011
N	1.353738299207	-2.538630095292	-2.009008016097
P	2.067208427782	-1.146209889067	-1.635855645026
N	1.464152944344	0.189854934552	-2.143847726707

P	0.466618548862	1.341311133173	-2.427376377295
Si	-2.487717211360	-1.620050924585	-4.738640525760
C	-3.701019147111	-1.629839699577	-6.170365200962
H	-3.145651392330	-1.110448222597	-6.955904618091
H	-3.774507744363	-2.656303649222	-6.536644545261
C	-3.015349347296	-0.971467916563	-3.073988909931
H	-2.261856956173	-1.155520203987	-2.308527775506
H	-3.186199151818	0.102570078205	-3.098900920262
H	-3.946356416923	-1.456104719310	-2.768870848406
O	-2.603118773856	-3.585098360033	-4.196597561174
H	-1.902437757067	-3.753783342494	-3.498450654021
C	-5.132554676364	-1.035615252777	-6.093054413150
C	-3.764429417798	-4.374953368789	-3.991417977943
C	-5.749825756840	-1.119642299439	-7.533626650560
H	-6.669968289019	-0.515370299374	-7.539684117383
H	-6.061213613093	-2.158606896359	-7.700542378189
C	-5.508539602960	-5.717455761188	-4.932506425826
C	-6.153899086805	-5.738856299443	-3.701090733905
C	-5.551952064304	-5.139473192362	-2.595976268596
C	-4.329883998776	-4.466257761671	-2.706979153397
C	-4.289170201825	-5.046698712761	-5.102657058226
H	-5.942460818385	-6.231512177109	-5.786868300711
H	-7.108360070715	-6.246336365221	-3.592663600316
H	-6.028526403964	-5.190081754775	-1.620628971555
C	-0.805361119303	3.031671354112	-0.919135396701
C	2.053753022179	3.352922357116	-2.059865324104
C	3.270113640334	-0.254057794071	0.498677750353
C	4.442991089463	-2.123216917998	-1.638693528261
C	-2.220342881208	3.012744214723	-0.724330586356
C	4.668504433166	-3.381702761255	-2.253164672183
C	3.465103749873	3.219536966264	-2.217281914858
C	2.802117112903	0.797236374112	1.339377314108
C	-0.772737955486	7.871571953371	-1.341357333043

C	-0.073191993932	6.685529035356	-1.324579930333
C	-0.730008787122	5.454264729359	-1.053571227312
C	-2.143875634378	5.471319164555	-0.838428659332
C	-2.833079035129	6.715400110018	-0.839591762137
C	-2.164635865519	7.892078545324	-1.082943439412
H	-0.252257242596	8.799794224385	-1.560084309050
H	0.990536574732	6.683686439374	-1.531930053952
C	-0.049141389086	4.192665944045	-1.012222009281
C	-2.833641972421	4.254105078944	-0.641205647500
H	-3.905731017938	6.714908699471	-0.660914943393
H	-3.895472905051	4.306424376687	-0.423983323497
C	4.001380423283	6.150293336744	1.658378774513
C	4.530689268401	5.464614346553	0.589589458600
C	3.692965677575	4.751853692195	-0.311457928684
C	2.273353478308	4.802756087891	-0.133720845577
C	1.760283920100	5.501191099965	0.994336937546
C	2.601863356092	6.150385049292	1.870771837905
H	5.325292817478	3.944195175193	-1.454681771256
H	5.604809324411	5.432228975213	0.429285256644
C	4.244978535564	3.969565897625	-1.352849087175
C	1.441116139998	4.120240918095	-1.084287005732
H	0.690384445015	5.515551191509	1.166332665807
H	2.186590443809	6.670265759063	2.729557035782
C	7.780012273561	1.529368835484	0.521809976722
C	6.912924085693	0.516268739096	0.176656071100
C	5.547910618029	0.558435923197	0.572341894906
C	5.087292767765	1.682203380363	1.330266677965
C	6.015766111982	2.693674247010	1.697054947599
C	7.331143247098	2.625359145061	1.298432475933
H	8.816214439991	1.486477821856	0.198065913286
H	7.260682102055	-0.321470320373	-0.418201918253
C	4.608143745769	-0.455790534514	0.200919447148
C	3.728466228946	1.748555430413	1.726186867394

H	5.657039400214	3.534422697737	2.282980852571
H	3.411724399705	2.565093615225	2.368853033687
C	7.560542794517	-4.568622463844	1.494864542730
C	7.002914957016	-4.859374492727	0.271850733901
C	6.145325036523	-3.932213734635	-0.380053509775
C	5.881956167655	-2.664440061295	0.235016793575
C	6.450941884999	-2.410235917361	1.513900062186
C	7.269296722769	-3.335110720311	2.123787489786
H	5.722101525094	-5.221562235495	-2.065452947478
H	7.201285220006	-5.811350963577	-0.214689396792
H	6.231742615696	-1.476941794796	2.017889971688
H	7.690633477229	-3.116573884866	3.101217716782
C	5.524374669101	-4.253939211711	-1.611862903340
C	5.017287464428	-1.736891578079	-0.442000039625
H	-2.702528510278	8.835987831495	-1.091614946937
H	4.653417658068	6.682941456166	2.345241134634
H	8.028223202564	3.411085868633	1.576873445052
H	8.212137259706	-5.286528206353	1.985235939877
C	0.518175601163	-4.376314508516	-0.195722762526
F	1.292960687616	-3.814865781748	0.738680829017
F	-0.574359456663	-4.888513446793	0.388400135512
F	1.197411428197	-5.368619220928	-0.785154320282
C	1.414436869076	0.818625307711	1.861935285219
C	0.825904321445	-0.364040300489	2.355591715636
C	0.697774509888	2.024149186640	1.938718792364
C	-1.188938461479	0.878920435603	2.889481917995
C	-0.454119146195	-0.324309284991	2.881617045301
C	-0.603798273882	2.061987831778	2.437621531267
H	-1.164571407877	2.992732265033	2.425305755612
C	-2.561427776575	0.579027058462	3.306568223185
C	-2.647605464900	-0.797629061900	3.599258730753
C	-3.700362373018	1.383857923694	3.322532181251
C	-4.933023666357	0.799127174568	3.622771196418

C	-5.021412411992	-0.566555493735	3.913437506439
C	-3.873756678254	-1.370368214033	3.912094348633
H	1.372179492688	-1.300382882100	2.306569165685
H	1.150061229385	2.929776007861	1.551296420914
H	-3.636698253424	2.437418079651	3.063477872593
H	-3.951387312835	-2.433165931021	4.129272419147
H	-5.990045699151	-1.011789838917	4.122132592444
H	-5.834198699465	1.406734509096	3.615992700280
C	-1.275846980097	-1.461092590949	3.486319148187
C	-0.740555615086	-1.792777068855	4.903039326548
H	-0.734205820771	-0.901159980142	5.538145363864
H	-1.371191862264	-2.551827562331	5.380530598955
H	0.282281343940	-2.182065481380	4.842687666516
C	-1.272795351454	-2.739224116033	2.631591523077
H	-0.272627180559	-3.182474821197	2.611775677485
H	-1.957096531756	-3.485331347096	3.052382264531
H	-1.558623653729	-2.542583212555	1.597307931853
C	4.088689875377	2.305415536143	-3.208185823464
C	3.522741270548	2.053711721571	-4.475312313086
C	5.296815723426	1.662324005528	-2.867792282405
C	5.384301110676	0.581125507894	-5.010130922989
C	4.159533615019	1.187299930675	-5.348562191165
C	5.952026800241	0.814126255113	-3.757044216749
H	6.880729632058	0.331890765151	-3.461998096182
C	5.821126274684	-0.246210867141	-6.139716433063
C	4.845119317505	-0.162671117223	-7.153916053624
C	6.956490219923	-1.041632875817	-6.309734021200
C	7.109864106191	-1.751053733177	-7.503320539915
C	6.144452987180	-1.663527470959	-8.513497858929
C	5.004906327844	-0.866994188293	-8.341537524858
H	2.592521307240	2.524078796825	-4.768465341459
H	5.716504336430	1.805665894246	-1.879031719209
H	7.705303064956	-1.114301205010	-5.524898976825

H	4.252026446711	-0.807750641431	-9.124028371962
H	6.279588344253	-2.220427198826	-9.436996874899
H	7.985744263907	-2.377606651508	-7.648818557523
C	3.681291554899	0.718970127773	-6.717339909971
C	3.429568734703	1.889277022780	-7.686998255268
H	4.324575039841	2.510309848222	-7.799383054380
H	3.144126080197	1.512029064370	-8.675830357593
H	2.610611149034	2.516500481899	-7.318196502292
C	2.394816665777	-0.125035453225	-6.566689489026
H	1.574912503244	0.504604027855	-6.210773788887
H	2.099892737039	-0.553339415410	-7.529671774773
H	2.541518174535	-0.947194524031	-5.859702920779
C	-2.347967118149	3.012930732650	-4.435936383337
F	-1.690433714378	3.990065624811	-3.805559403883
F	-2.862234143824	3.483695071049	-5.575556755147
F	-3.349698910815	2.583067551313	-3.655393453769
C	-3.037959723113	1.779861697614	-0.571043037223
C	-4.410549392389	1.822833953044	-0.915271176686
C	-2.530370660558	0.590335912285	-0.015158938690
C	-4.727771879463	-0.386313146333	0.025675622687
C	-5.240625781823	0.759052411560	-0.611731001087
C	-3.365684129700	-0.483874316286	0.283400590251
H	-2.946788866236	-1.376789480379	0.726515628353
C	-5.830875545062	-1.312446964280	0.283645857805
C	-7.013335532094	-0.756413265065	-0.243474540737
C	-5.856462532891	-2.539070593779	0.950124969632
C	-7.068378180126	-3.228351978377	1.045470972684
C	-8.237815110565	-2.691917270944	0.492922205574
C	-8.216008285776	-1.445599665212	-0.146392776148
H	-4.813045221027	2.689220591173	-1.430745395480
H	-1.481205421480	0.502851327206	0.217672495394
H	-4.952449238712	-2.936733862852	1.402425800830
H	-9.131251777461	-1.024658225053	-0.556138114714

H	-9.172482501519	-3.240684334015	0.572643135838
H	-7.105498152527	-4.185123584205	1.560191035781
C	-6.743464201203	0.622394314635	-0.844063686177
C	-7.096340461977	0.673973238625	-2.343255264283
H	-6.574036406844	-0.110750683930	-2.895605104931
H	-8.174085125116	0.538143411154	-2.492244926335
H	-6.811302401450	1.640175934872	-2.775126926332
C	-7.517632570835	1.714117649776	-0.073699437237
H	-7.269523922280	2.707693115022	-0.465190599284
H	-8.599279146435	1.567914215939	-0.175029053835
H	-7.263190225509	1.690208154966	0.991118445685
C	4.011994788618	-3.775817016750	-3.524586138704
C	3.182084960296	-4.908816355918	-3.555938244657
C	4.279438961841	-3.071546968873	-4.707150551353
C	3.003970182382	-4.670898023376	-5.962913125615
C	2.669638978526	-5.340429311365	-4.769720543461
C	3.791522597862	-3.519277096486	-5.934410430382
H	4.040627041497	-2.981885248890	-6.843630796907
C	2.419605766336	-5.407187173197	-7.087064407393
C	1.677668940886	-6.491120212819	-6.574805624239
C	2.532393205884	-5.206795893100	-8.462860000947
C	1.893626262998	-6.099576631661	-9.326895451689
C	1.136983973763	-7.163135253244	-8.821244673595
C	1.023631951467	-7.361474638030	-7.438153054837
H	2.935440833684	-5.415485486908	-2.627932030950
H	4.901120985887	-2.185902685247	-4.666615317184
H	3.105030096713	-4.369943281696	-8.853612835485
H	0.444096139043	-8.196143713971	-7.050215665099
H	0.639798558373	-7.845077970398	-9.506222996439
H	1.973815364297	-5.959319484768	-10.401734353196
C	1.762246050666	-6.535111252877	-5.049972391672
C	0.379209723896	-6.357729198167	-4.386367177540
H	-0.098079654820	-5.426200574317	-4.701369732470

H	-0.281389612742	-7.193092722223	-4.648274809297
H	0.474975426237	-6.315963234762	-3.297025750270
C	2.406220494644	-7.852746591439	-4.568792081574
H	2.528934962449	-7.842752685165	-3.479560454569
H	1.775263042369	-8.709844066411	-4.831520309512
H	3.390547182647	-7.997284520935	-5.025755583842
C	-3.580488146014	-5.133738832612	-6.433476547721
H	-3.346821607334	-6.181663499885	-6.658742806128
H	-4.211569126185	-4.762738040808	-7.249561064663
H	-2.646900613060	-4.575794844245	-6.452455890760
C	-3.632496611493	-3.988357879357	-1.456657180425
H	-4.343292985042	-3.947316593496	-0.630110543053
H	-2.830010540455	-4.682123074696	-1.180866432118
H	-3.172428691919	-3.007200125947	-1.546439431206
C	-0.664119971752	-1.890132218099	-5.239519801541
H	-0.248372813514	-2.735228520025	-4.682611226282
H	-0.085373143887	-1.016620108792	-4.934847650090
C	-0.561181633775	-2.135335846169	-6.730090806535
C	-0.621936401061	-1.077742880859	-7.654197515502
C	-0.483320892593	-3.441147461662	-7.229258685575
C	-0.637998146594	-1.327437596352	-9.025604191471
H	-0.669165651067	-0.057021949434	-7.293898960819
C	-0.514437232058	-3.695492242565	-8.601686209328
H	-0.387330713682	-4.273576126041	-6.539265424047
C	-0.597673497519	-2.638575309346	-9.507106839810
H	-0.691441191272	-0.491720675765	-9.718980900817
H	-0.456256255070	-4.720040211266	-8.952981137282
H	-0.622701827312	-2.832307868444	-10.576383524499
C	-6.030951934646	-1.885067604562	-5.172284792412
H	-7.056098880994	-1.493923139791	-5.165517218419
H	-6.070015387878	-2.929632240508	-5.497075447179
H	-5.669883157575	-1.881513049554	-4.138797974851
C	-5.156058050497	0.417395994671	-5.576842081439

H	-4.851185075444	0.470986902779	-4.529527519778
H	-4.492174563151	1.077339100268	-6.134477102313
H	-6.175569293244	0.818187665654	-5.640861766545
C	-4.868502464086	-0.708566554960	-8.696841965240
C	-4.468466794104	-1.611440163668	-9.599483028986
H	-4.745224125449	-2.660445214368	-9.522794167245
H	-3.833093803643	-1.340979750449	-10.439142910675
C	-4.438039564627	0.736119742591	-8.803264105232
H	-5.273921003644	1.419895909241	-8.608941507575
H	-3.657372688826	0.971653567684	-8.069141579539
H	-4.032827524209	0.958422555579	-9.795710329164

TS’

O	0.975792166226	2.503440603643	-2.719570283277
O	-0.282562178195	1.973486810064	-0.586541095616
O	1.689280769839	-1.043002791858	0.361394036640
O	2.688330153269	-1.547572864449	-1.964177637172
N	-1.393302169263	1.426835773266	-2.844317379381
S	-0.529118445832	-3.130165325981	-0.065132093266
S	-1.814520112204	1.159556903430	-4.326970185167
O	-1.022842982613	-2.109943362461	0.867276213675
O	-1.530907860123	-4.083438472541	-0.614338822750
O	-0.729045436515	1.039356646630	-5.309279508674
O	-2.909875232228	0.141887270914	-4.424510810916
N	0.412445178555	-2.620148026199	-1.234391185454
P	1.286902464461	-1.264779293752	-1.200303326035
N	0.706460886763	-0.004452566028	-1.908918981021
P	-0.001464269353	1.373178484279	-2.064036862949
Si	-2.960251012713	-2.089224901943	-3.738599344843
C	-1.476218404045	-2.394796835991	-4.862606181521
H	-0.888690859622	-1.473168190249	-4.790650328064
H	-0.885063149549	-3.145611263642	-4.332185641880
C	-2.665849415794	-1.363348075884	-2.048701226373

H	-3.091600444842	-1.979396854273	-1.255309197449
H	-1.606633869516	-1.220036967832	-1.853549375447
H	-3.137140060772	-0.384884175156	-2.000665584573
O	-2.945451727104	-3.961111379694	-2.833650799664
H	-2.410663431429	-3.905930406082	-1.985165748624
C	-1.542052093986	-2.794600111923	-6.365775413046
C	-2.969651987181	-5.310576957621	-3.267156082792
C	-2.294468382349	-4.153088340991	-6.504407879134
H	-3.320715599238	-4.022407709080	-6.136294886955
H	-1.810689396855	-4.865077722123	-5.828795389098
C	-4.255504091091	-7.242729115434	-3.870619016593
C	-3.081180857936	-7.929823356261	-4.161504881055
C	-1.852508487813	-7.315687631459	-3.941523152587
C	-1.762154271493	-5.991613170470	-3.490819738006
C	-4.223239974694	-5.929246995537	-3.388282632857
H	-5.218217427785	-7.735692584941	-3.981825298949
H	-3.121833867084	-8.952771889842	-4.525121407645
H	-0.935053020724	-7.869504299975	-4.111544218644
C	-0.772204325712	3.283380433933	-0.515736473281
C	1.955832217513	3.121220167553	-1.933499803229
C	2.809082174536	-0.308650617741	0.734509621536
C	3.578703365817	-2.475295560697	-1.410149519825
C	-2.151285716561	3.462688560344	-0.213903325664
C	3.816417421358	-3.699707868968	-2.106829574095
C	3.319266950614	2.807284801309	-2.203058364965
C	2.598462425891	0.835502065705	1.554703909350
C	-0.170531022068	8.051651923935	-1.199334385013
C	0.368947533050	6.784522166410	-1.184500560082
C	-0.409411297396	5.661919859054	-0.790931082015
C	-1.785054506714	5.875090673875	-0.453473639381
C	-2.305122259645	7.198232077261	-0.459680247825
C	-1.516928274086	8.266350274990	-0.819681220145
H	0.442007501820	8.893278367118	-1.510616840799

H	1.399436893208	6.633921219705	-1.484727942387
C	0.110852969185	4.323369280633	-0.749970996943
C	-2.610941923235	4.764895126318	-0.152912890626
H	-3.347684625480	7.346545380290	-0.188612545650
H	-3.651326709319	4.944032984474	0.104693739981
C	4.622862680837	5.802926081332	1.427111106362
C	4.949940909638	4.979901138930	0.374918050614
C	3.940518173623	4.356936280434	-0.408371161144
C	2.564448844838	4.636010635082	-0.134801791304
C	2.261657691445	5.473645859329	0.976043391794
C	3.261843233126	6.034471240558	1.739077934718
H	5.326179862550	3.259712435324	-1.639858565180
H	5.988849593560	4.768974826026	0.137513917961
C	4.276282767518	3.453254082645	-1.441708606346
C	1.557043768891	4.030419505786	-0.963514720818
H	1.226171817504	5.665776821016	1.229638901182
H	3.001826656018	6.663124020012	2.586083659156
C	7.549187094649	0.628989846369	0.361357405160
C	6.493055596392	-0.223925945834	0.126361847687
C	5.189791058733	0.077477671468	0.608422226107
C	4.994176849290	1.295666235996	1.335780132646
C	6.112149554094	2.134749687781	1.589967808920
C	7.362011588925	1.815152325327	1.110641636755
H	8.534112654656	0.386884438011	-0.028111700230
H	6.645729034324	-1.132141350911	-0.446178005203
C	4.057445758217	-0.767918843047	0.360730967540
C	3.703408418126	1.621191546755	1.823347686377
H	5.952419583572	3.046779011046	2.156940512978
H	3.587657110764	2.501400917272	2.449253243395
C	6.525719778042	-5.148043438452	1.699602338550
C	5.982915291161	-5.378431477102	0.456957141746
C	5.202039829188	-4.384803595750	-0.193640764248
C	5.004915394554	-3.119609177789	0.444252749276

C	5.567946182404	-2.918657556217	1.733705288931
C	6.305638506568	-3.908284479677	2.345402944589
H	4.889204990249	-5.540907136624	-1.978215362256
H	6.133836401616	-6.331181206632	-0.044932980719
H	5.400597234522	-1.978392510253	2.245643194485
H	6.718601178754	-3.736364379602	3.335511606474
C	4.637640773461	-4.615079328038	-1.470008422835
C	4.216127980308	-2.128011752655	-0.232247820026
H	-1.927905742113	9.272045613002	-0.828246645791
H	5.402810494368	6.267080898599	2.024477874767
H	8.206352284741	2.471174674725	1.304215730450
H	7.114622532119	-5.917356871258	2.191260080848
C	0.562224457487	-4.265800189070	0.948836158741
F	1.595254031703	-3.587374087573	1.462821792533
F	-0.155342772253	-4.784022838521	1.952471863491
F	1.032037025719	-5.253999480103	0.181778049477
C	1.294444821982	1.097077752381	2.214007223953
C	0.618488410031	0.054619297005	2.881082076193
C	0.790785790055	2.405032820944	2.293258108359
C	-1.020047498578	1.658048026409	3.681661493426
C	-0.509868182859	0.344157460361	3.632834538593
C	-0.372941087738	2.691813182667	3.003962482518
H	-0.765913761651	3.704956677249	3.022220167383
C	-2.229424399368	1.659260814760	4.507666272002
C	-2.423458089823	0.359147336624	5.014718551649
C	-3.126899999749	2.684512706943	4.809206773354
C	-4.223336674751	2.399015953384	5.626099109719
C	-4.415835252896	1.108907829034	6.133658576102
C	-3.513542023954	0.081743092116	5.830090718340
H	0.992827210770	-0.962098794418	2.817368872943
H	1.311006208740	3.197288773733	1.770333432199
H	-2.980763034164	3.684548911045	4.408750023645
H	-3.670651557310	-0.919550782820	6.224059409173

H	-5.275540756284	0.901712877015	6.765490875239
H	-4.935278992615	3.184081517619	5.867127005898
C	-1.314617687153	-0.580069498097	4.547116490466
C	-0.436164759173	-1.017362087259	5.744395144425
H	-0.025909966741	-0.148676699638	6.269585417100
H	-1.026661390423	-1.604436814289	6.457293104840
H	0.398280984845	-1.637517957337	5.397302989338
C	-1.874774802184	-1.825598928815	3.838260267728
H	-1.072399133402	-2.447888281766	3.432736358546
H	-2.456711476302	-2.429012113235	4.545192339195
H	-2.523917658687	-1.545948054332	3.007345770132
C	3.745389439978	1.861044457449	-3.262870150443
C	3.196042193065	1.905417216555	-4.558468764092
C	4.792684530409	0.960875080959	-2.989094709445
C	4.774864756518	0.198120319140	-5.265001552394
C	3.718350977514	1.086566740703	-5.547511787158
C	5.310012997062	0.127926013226	-3.978105467657
H	6.105442337506	-0.573595572976	-3.739789724113
C	5.080475399309	-0.564422822687	-6.478174765804
C	4.210528398528	-0.138106011166	-7.501701648939
C	5.986594272302	-1.602730373183	-6.699090015319
C	6.010277308977	-2.220165053689	-7.951380004186
C	5.145265020960	-1.800543050335	-8.968777284154
C	4.241951338155	-0.752376386144	-8.748363134012
H	2.376542522033	2.581369342975	-4.777685368723
H	5.191352766539	0.904893213854	-1.982345512919
H	6.642204059217	-1.944616720103	-5.902714360719
H	3.567385736346	-0.434135364946	-9.539759405150
H	5.168427070786	-2.299173147007	-9.933880073268
H	6.696832724081	-3.041985032589	-8.134873598036
C	3.294384617949	0.977865882824	-7.009344784569
C	3.568017588423	2.295114164238	-7.768357778709
H	4.621737526847	2.582787880780	-7.691496596328

H	3.317088595956	2.184340373966	-8.829663604720
H	2.956654083067	3.105939938830	-7.356499143160
C	1.808087582138	0.594624248765	-7.134978091831
H	1.163719149484	1.393269420949	-6.760455777807
H	1.544953311955	0.399333826246	-8.181125293805
H	1.585739169917	-0.300547896415	-6.549093995382
C	-2.745091766994	2.724129326754	-4.736116341427
F	-1.894069680753	3.750364303045	-4.777557582897
F	-3.336792073867	2.584857934736	-5.928091808387
F	-3.683081026760	2.965951125057	-3.813228663763
C	-3.078381942181	2.316575042143	-0.066476568599
C	-4.314432545070	2.371146206210	-0.739253448161
C	-2.739590886516	1.161765643086	0.662571947269
C	-4.795995941750	0.114260037779	-0.006103632783
C	-5.161278306868	1.275801563007	-0.711329836968
C	-3.596714119032	0.065717377494	0.703999104081
H	-3.279850699879	-0.840382009041	1.207940219917
C	-5.768652611210	-0.937455881746	-0.311347224739
C	-6.731811279030	-0.412854326154	-1.198204168880
C	-5.811742267545	-2.277596982276	0.077524583052
C	-6.834080210848	-3.088758258128	-0.423782341726
C	-7.788173956917	-2.571050195626	-1.307826709700
C	-7.741513252535	-1.226053944065	-1.697647820272
H	-4.554445186659	3.238534352445	-1.346645151713
H	-1.785952505096	1.103854599666	1.168938129649
H	-5.061355942545	-2.683518226293	0.751129647827
H	-8.481178573915	-0.830984615484	-2.389907985832
H	-8.569681303392	-3.218376854425	-1.696609602737
H	-6.884175249385	-4.133812589612	-0.130128821103
C	-6.470378034326	1.068589272258	-1.467267011249
C	-6.314815173356	1.381869114566	-2.968161732141
H	-5.434975144697	0.886457001728	-3.383653272712
H	-7.198031375021	1.058237320424	-3.529919885786

H	-6.186524687896	2.458051784255	-3.125525875248
C	-7.602570556827	1.931712561400	-0.864457561568
H	-7.370229369779	2.997140012922	-0.976128608847
H	-8.551598174273	1.732270853148	-1.375801247336
H	-7.733400834269	1.717064305311	0.201007029174
C	3.355329507818	-3.967681692127	-3.493063867087
C	3.127637131576	-5.294117578489	-3.922323327776
C	3.309430254796	-2.931010907475	-4.446580045711
C	2.919316276091	-4.517244198001	-6.208755493832
C	2.922770296291	-5.563502617596	-5.267915260419
C	3.095127897316	-3.199167538891	-5.795167625281
H	3.084049700730	-2.389480503759	-6.514787155586
C	2.685015290270	-5.077606306263	-7.540191359750
C	2.562319273019	-6.475042376169	-7.419821121605
C	2.557094438802	-4.451785637730	-8.781327544116
C	2.295807338580	-5.240894020559	-9.903553423556
C	2.170614628190	-6.630532339667	-9.786560630150
C	2.306531863147	-7.255882482398	-8.540685284642
H	3.117531653378	-6.102320636316	-3.195516843699
H	3.477053252160	-1.908593197291	-4.136592013561
H	2.656739546241	-3.373956361086	-8.867350512535
H	2.204982256969	-8.335295737255	-8.455374175088
H	1.963575972322	-7.229014952822	-10.669724599157
H	2.184061135640	-4.771614611397	-10.877421918505
C	2.722197443586	-6.910565024747	-5.964504584192
C	1.460146159290	-7.642597628937	-5.471840061161
H	0.564291888863	-7.045807527007	-5.663189687369
H	1.343659846279	-8.600833159379	-5.991427009360
H	1.521926196644	-7.842796441190	-4.395761733661
C	3.962885650267	-7.810817438011	-5.779754323281
H	4.094932454598	-8.070632038686	-4.722715548807
H	3.852571749579	-8.741281070405	-6.348640982568
H	4.868626261041	-7.301791908673	-6.124417924551

C	-5.506692908626	-5.272223364136	-2.941901694263
H	-6.158865938339	-6.014525776639	-2.469067750534
H	-5.325001080890	-4.476071357380	-2.218671153913
H	-6.071216081734	-4.842540456841	-3.777485881191
C	-0.393335285028	-5.396994230924	-3.252021944679
H	-0.411948459627	-4.410302505364	-2.798876089192
H	0.182645728965	-6.037751746288	-2.575280258275
H	0.173893461048	-5.323108005492	-4.185010897644
C	-4.813004908757	-2.011065627751	-4.129993850537
H	-5.127873801932	-1.173644507116	-3.498966892568
H	-5.253615648157	-2.891031642626	-3.663283064680
C	-5.365454611125	-1.831572115828	-5.520935332423
C	-5.441266010462	-0.562501560176	-6.118093743799
C	-5.854412354061	-2.930288013982	-6.241031987486
C	-5.979189167288	-0.404997340296	-7.394701937709
H	-5.042847388547	0.299040579796	-5.595306503633
C	-6.397769951784	-2.773858428318	-7.517063651969
H	-5.802530783555	-3.923403718959	-5.801391269481
C	-6.461293515506	-1.509024709740	-8.101780485636
H	-6.017675691160	0.585982200084	-7.839824788315
H	-6.766677947292	-3.643286365797	-8.054775099433
H	-6.881408157459	-1.384528996802	-9.096177364685
C	-2.180387981148	-1.681328844956	-7.211652313112
H	-2.069637545045	-1.905526516685	-8.278582563776
H	-3.244000643462	-1.560658727454	-7.001131851541
H	-1.687370932429	-0.723796600187	-7.013721587848
C	-0.085808470978	-2.974437257313	-6.820894292358
H	0.483498138530	-2.055557337973	-6.655040063573
H	0.405252309181	-3.773673268619	-6.259633524596
H	-0.026409500086	-3.229765107066	-7.881894953216
C	-2.341046629269	-4.766858436564	-7.893143088887
C	-1.362959370397	-5.565697231038	-8.336633244820
H	-0.489796578966	-5.801095122375	-7.733166825211

H	-1.385909124673	-6.001864583757	-9.332608590564
C	-3.560589517883	-4.473167656131	-8.731703226246
H	-4.456704539845	-4.901811641493	-8.262815564265
H	-3.752720000616	-3.397874880626	-8.812337406619
H	-3.471385278548	-4.890176605686	-9.740539565668

B

O	1.275821484946	2.706396457456	-3.003943303326
O	-0.162939432542	1.779527633714	-1.144397512182
O	2.185649047442	-1.103461196360	-0.147968807628
O	3.490949714895	-1.210888247585	-2.341273941996
N	-0.706398457396	1.213942495430	-3.694490949950
S	0.012950070406	-3.189061030125	-1.535171313445
S	-1.047177987693	1.979523742592	-5.038352122467
O	-0.868937111354	-2.286343761422	-0.775277916560
O	-0.641263843624	-4.075322387822	-2.549277234681
O	0.058093980458	2.717584497694	-5.662955634667
O	-1.856423532818	1.084423046669	-5.889336470875
N	1.319303272056	-2.569694606095	-2.125374798827
P	1.980839864035	-1.139569035030	-1.772950012921
N	1.339799947132	0.138189077989	-2.365415655782
P	0.439171442537	1.383550862969	-2.604932688783
Si	-2.508776230925	-1.726476067766	-4.606664066307
C	-3.657833704341	-1.513060000180	-6.067394136043
H	-3.057437072038	-0.869902837029	-6.718640109200
H	-3.698691033837	-2.462241988487	-6.610256967606
C	-2.920309668946	-0.835643577707	-3.036312830522
H	-2.179360902979	-1.027513149332	-2.259724873005
H	-2.919782352196	0.238694801743	-3.214272932808
H	-3.905952751762	-1.119526272417	-2.657081288583
O	-2.639892869348	-3.545049669315	-4.088163836189
H	-1.882565087359	-3.786842040332	-3.446181772049
C	-5.082707136856	-0.908231378869	-5.980369477388

C	-3.841072081897	-4.293780161326	-3.867111044204
C	-5.646740042501	-0.848342088003	-7.441501193939
H	-6.592623529096	-0.286149261940	-7.412507507032
H	-5.904485724289	-1.873756770683	-7.736995563049
C	-5.636106508128	-5.585364287058	-4.760387782512
C	-6.254515484177	-5.576369621203	-3.515213118168
C	-5.619435976283	-4.977037973445	-2.428286927540
C	-4.381703628813	-4.340618453336	-2.572544649131
C	-4.403189601384	-4.948786495591	-4.966560775470
H	-6.104296972440	-6.092801482660	-5.600185673528
H	-7.219091223003	-6.058126093410	-3.382391483632
H	-6.081233267321	-4.996580766490	-1.444999724849
C	-0.790648943856	3.018299634761	-0.993904304663
C	2.100198014145	3.328904035950	-2.075694659192
C	3.106731817248	-0.204994845053	0.405211083156
C	4.375320057011	-2.076593499927	-1.681941839503
C	-2.203986575187	3.028664175868	-0.786309281824
C	4.646727816800	-3.337600729623	-2.273866360862
C	3.510616614986	3.199363395165	-2.247123349522
C	2.611630908388	0.811158249588	1.272759508324
C	-0.647651627430	7.867209518829	-1.234292078693
C	0.025725284613	6.666011195668	-1.249809678012
C	-0.658327391171	5.440873780117	-1.021328981530
C	-2.073450050176	5.483920623314	-0.816918289492
C	-2.735204744086	6.741943311268	-0.782036696710
C	-2.039973903091	7.911055447252	-0.983245171006
H	-0.105326126246	8.789523460131	-1.422901978390
H	1.089613656504	6.648410763799	-1.454301891355
C	-0.003033125553	4.162964926336	-1.023354598126
C	-2.791892115500	4.277152761923	-0.660774191514
H	-3.808991077722	6.758820429875	-0.611072233945
H	-3.852038276925	4.343595260174	-0.437772948398
C	4.060651406566	6.073751407304	1.668476491832

C	4.584419684938	5.402732382394	0.587338501688
C	3.745549178364	4.688402575878	-0.310612660408
C	2.326535035241	4.725969477195	-0.116982929598
C	1.820448226632	5.402472301569	1.026949603725
C	2.664931856179	6.052161143571	1.900600485489
H	5.376507993884	3.902377990846	-1.476921025887
H	5.657061946998	5.382223195562	0.416311299776
C	4.296365403477	3.924581376242	-1.368586542272
C	1.487845300888	4.068631242450	-1.080266015999
H	0.752057891250	5.406846949397	1.209157968584
H	2.254119069641	6.558146863436	2.769827248268
C	7.608091442995	1.589800713881	0.639792697584
C	6.751946270003	0.597781424093	0.215414037238
C	5.376261404206	0.619717235813	0.574025218372
C	4.891061539406	1.705666282845	1.368993786765
C	5.809000861096	2.690565066593	1.823150782594
C	7.136462879873	2.639979333612	1.464474671031
H	8.653448703777	1.563159769455	0.345011855680
H	7.117806346267	-0.209454698610	-0.410069023480
C	4.455486586604	-0.389935730142	0.146665028042
C	3.520590304853	1.750447849516	1.725163681153
H	5.433313722759	3.499008673380	2.442137219029
H	3.181586438652	2.532583824942	2.398386353947
C	7.440697454454	-4.427623003434	1.577871262509
C	6.938481872005	-4.736051254365	0.335543748366
C	6.080895263082	-3.833928321724	-0.350960809739
C	5.762803505479	-2.570744171930	0.245965717677
C	6.272917534235	-2.299972307467	1.546150552709
C	7.090308619841	-3.201758085938	2.191146357075
H	5.751763440219	-5.142243747517	-2.039483983310
H	7.179816765382	-5.683759995567	-0.139808681847
H	6.008618957155	-1.373543094761	2.040395023799
H	7.464847419703	-2.970324687791	3.184511400135

C	5.509870493316	-4.178720010986	-1.599076004080
C	4.904544240658	-1.667123730563	-0.473099293122
H	-2.557158272136	8.866354717776	-0.965748238807
H	4.715334273238	6.609121567493	2.350762343766
H	7.825313620503	3.405922610441	1.810370965348
H	8.092146707502	-5.126300003609	2.095459533721
C	0.616219699554	-4.456287804737	-0.301853118128
F	1.365851354247	-3.858981627653	0.631598729484
F	-0.439086068647	-5.035476153559	0.287111666103
F	1.345960506895	-5.396511323949	-0.911670797208
C	1.224446543794	0.780367225480	1.796281667006
C	0.681105022938	-0.435326490854	2.260072676063
C	0.479549030408	1.962931486675	1.936681702701
C	-1.339278788914	0.735442424870	2.919461239634
C	-0.578248513962	-0.447945756527	2.833897947273
C	-0.803023113565	1.948328749403	2.484819758975
H	-1.386974061899	2.863772849569	2.527282279565
C	-2.679974728207	0.387199138337	3.397680092265
C	-2.717598711812	-0.999994718695	3.650093532830
C	-3.832367620692	1.163885325778	3.515487015122
C	-5.028793305829	0.542202660954	3.881567378001
C	-5.067925665655	-0.832921670350	4.135349688267
C	-3.906841589174	-1.610002263798	4.028016676105
H	1.249020217877	-1.354399736583	2.159649928447
H	0.898529909815	2.893593158251	1.570580738643
H	-3.807835502057	2.227158915269	3.291843660521
H	-3.945635204228	-2.680004397933	4.219197121278
H	-6.009850147353	-1.305460127434	4.399166286111
H	-5.940508540771	1.128880261873	3.957237038395
C	-1.341035175598	-1.626566490624	3.434749111135
C	-0.720498830419	-2.001411789823	4.805227721680
H	-0.696569898942	-1.135351214273	5.474326189647
H	-1.307628515709	-2.792633478686	5.285928466720

H	0.305020865583	-2.364791402179	4.672621578574
C	-1.357472543784	-2.871454098259	2.530864196920
H	-0.348778256214	-3.283819726151	2.431920641467
H	-1.994123584277	-3.651820104236	2.964252738528
H	-1.711557901286	-2.646860421857	1.523469811127
C	4.105368667152	2.309696159017	-3.278885860256
C	3.505516861812	2.111366171287	-4.540996991803
C	5.298408709733	1.619305476525	-2.982100763866
C	5.284466382022	0.566126390879	-5.143227121917
C	4.078195423481	1.228154569226	-5.440162526212
C	5.897619720899	0.763134703291	-3.904629869108
H	6.815641598554	0.241765701577	-3.644563068011
C	5.645759473318	-0.274431723808	-6.290748949257
C	4.641530164193	-0.140955883660	-7.272026696446
C	6.733698543528	-1.124794639880	-6.499276985160
C	6.813346294224	-1.836564333067	-7.698495874561
C	5.821257418495	-1.698537902083	-8.676692792397
C	4.727792873157	-0.848661870020	-8.465495668890
H	2.593531491515	2.628757550418	-4.809218257718
H	5.747217223851	1.729858705535	-2.001111750439
H	7.501686676398	-1.239194728412	-5.738413431719
H	3.952911532122	-0.751137718013	-9.222169086088
H	5.899001313996	-2.258051925771	-9.605256444759
H	7.651495199312	-2.505863461521	-7.872850821024
C	3.532700124739	0.789071245453	-6.793926211437
C	3.293380473626	1.974285553634	-7.748399178807
H	4.206554089315	2.563271958302	-7.884545644750
H	2.965565850449	1.613816136479	-8.730417065064
H	2.507912160520	2.625750118836	-7.350545441613
C	2.216589886351	-0.002727737827	-6.601787099935
H	1.437280145886	0.661693484209	-6.218577100963
H	1.873665370806	-0.417682300120	-7.555110327702
H	2.350692935616	-0.832313743528	-5.900539291489

C	-2.279142852459	3.282473739386	-4.504644379598
F	-1.676426788276	4.222884934022	-3.770449084562
F	-2.829020374749	3.848038989368	-5.583702877205
F	-3.256505823015	2.721095641799	-3.771232439331
C	-3.040179982934	1.808614548193	-0.629834422407
C	-4.411175402296	1.861767938421	-0.975434337844
C	-2.547617620209	0.630540258173	-0.036221933636
C	-4.759403947877	-0.311846998833	0.038588539652
C	-5.257915380825	0.820135883229	-0.634103700615
C	-3.397434901897	-0.420820349555	0.298348267001
H	-2.992319715224	-1.301773925311	0.777508977158
C	-5.875376032219	-1.211060258154	0.336541961585
C	-7.054893584033	-0.647452590062	-0.188455533768
C	-5.913909794268	-2.420614049681	1.033317392221
C	-7.136492855335	-3.085043217841	1.162101445708
C	-8.304026732396	-2.541018260608	0.612888241475
C	-8.268578818962	-1.311495150592	-0.057346181116
H	-4.799099713739	2.722713172119	-1.511715404492
H	-1.497730220715	0.537186659447	0.194319620485
H	-5.012887668489	-2.821147287315	1.488364426793
H	-9.181644518598	-0.883815011633	-0.464917362831
H	-9.247336996608	-3.070104731289	0.719483251490
H	-7.183438509467	-4.027471369995	1.701959637223
C	-6.768847498401	0.707929437410	-0.832554215375
C	-7.164603448083	0.725136658750	-2.321137495558
H	-6.694038739626	-0.099831838919	-2.861308724510
H	-8.251263265270	0.629434471625	-2.431910714079
H	-6.854545657144	1.663085455941	-2.795237255240
C	-7.499745688870	1.837297681174	-0.073208380192
H	-7.241267194461	2.813972122086	-0.498913847246
H	-8.586393909804	1.711255656863	-0.143006745729
H	-7.218624659228	1.837294584722	0.985065002136
C	4.033927055496	-3.775376054355	-3.547976732353

C	3.400711173667	-5.028604436676	-3.605875774264
C	4.152277224717	-2.999113800568	-4.710736664259
C	3.145590536730	-4.755255591692	-6.001027750845
C	2.949495441790	-5.504676379415	-4.825383428808
C	3.723863641270	-3.486174237313	-5.944664036419
H	3.860678998418	-2.887745654065	-6.839111537171
C	2.665836713267	-5.545617747559	-7.137139513423
C	2.110769510544	-6.745381835019	-6.646315881206
C	2.713667697735	-5.288907610693	-8.507440274875
C	2.188557327179	-6.237845714667	-9.387726792404
C	1.615052062570	-7.419241987454	-8.902990315462
C	1.574852201390	-7.678567342676	-7.525577647243
H	3.257348211439	-5.597509761748	-2.691727857660
H	4.614406371888	-2.022523021056	-4.648780167148
H	3.142901171416	-4.362557237313	-8.879465746916
H	1.139900904631	-8.604160181830	-7.155163667277
H	1.204133061176	-8.144861537600	-9.600088105372
H	2.215778600254	-6.052864407187	-10.458377110455
C	2.245122586437	-6.823109284897	-5.125797127739
C	0.878116429707	-6.896734375001	-4.410988199565
H	0.237071975237	-6.057055167591	-4.690814536894
H	0.359536466828	-7.829066586372	-4.664354686873
H	1.010054119159	-6.856645614467	-3.324047922766
C	3.115058922902	-8.029774284504	-4.712205879050
H	3.270661105036	-8.035521878266	-3.627069410488
H	2.627642511620	-8.971377027843	-4.990752817529
H	4.094001102037	-7.991527439447	-5.200720271769
C	-3.744294212771	-5.035055394770	-6.321655350966
H	-3.649681096352	-6.085928057082	-6.619350076642
H	-4.349804158357	-4.538671611442	-7.089093942346
H	-2.749041197231	-4.593700524835	-6.341349057734
C	-3.646309690947	-3.838295783698	-1.357076062464
H	-4.322598156109	-3.804857681772	-0.502215807580

H	-2.815820283687	-4.508377655198	-1.108347328395
H	-3.217013313357	-2.846451261118	-1.479443464929
C	-0.696123696767	-1.810507655254	-5.132311675676
H	-0.121114122872	-2.387506285511	-4.401238399119
H	-0.313554230367	-0.791397858139	-5.091330850738
C	-0.561241359432	-2.400403897171	-6.514492398304
C	-0.695013973828	-1.600707600314	-7.660628710768
C	-0.374385564665	-3.778125369133	-6.680820828919
C	-0.668328227439	-2.173721803080	-8.932499909453
H	-0.833423288915	-0.529560157196	-7.546039509597
C	-0.357145968759	-4.355146973973	-7.950256045916
H	-0.239581205501	-4.399992937672	-5.800968616737
C	-0.508827701459	-3.553483228670	-9.082458856213
H	-0.781003962780	-1.538339963125	-9.807268868701
H	-0.205261962902	-5.424719865280	-8.051827208742
H	-0.487332483265	-4.000941184998	-10.072608856763
C	-6.026151318619	-1.813023967449	-5.166047806478
H	-7.040134401453	-1.394507551224	-5.144142569025
H	-6.087262620997	-2.820767280408	-5.589479532598
H	-5.689496062307	-1.917234549627	-4.128099167294
C	-5.082254290380	0.495421903206	-5.341812621779
H	-4.882480918098	0.445669094672	-4.269870396071
H	-4.330960848289	1.152240203197	-5.777750665765
H	-6.066680655974	0.964004118539	-5.463694570021
C	-4.749429242437	-0.264519597088	-8.516304095118
C	-4.238786608260	-1.049081130602	-9.472747730602
H	-4.425853663208	-2.119975571148	-9.499434990658
H	-3.600783595431	-0.652069403597	-10.258920956993
C	-4.440162713025	1.215412698588	-8.479846418735
H	-5.325344628824	1.803722418682	-8.207930096797
H	-3.662916820362	1.442640506799	-7.740303822359
H	-4.078386097277	1.567159819097	-9.451651526629

B'

O	1.067312860151	2.523325185775	-2.608358243039
O	-0.253049196884	2.044507919499	-0.496252655044
O	1.698722139671	-1.141438115790	0.438640869032
O	2.654406963493	-1.540079179838	-1.913286969218
N	-1.331944163005	1.476868650968	-2.781477940047
S	-0.635389280841	-3.040178764796	-0.079320670866
S	-1.652660052087	1.657975253589	-4.317805454183
O	-1.211892048278	-1.983415680817	0.756666577585
O	-1.599667108014	-4.043681693267	-0.645550599552
O	-0.499513604624	1.518841028330	-5.219997627137
O	-2.890933198808	0.939033110213	-4.667773757130
N	0.372399192885	-2.602555001636	-1.208279892447
P	1.269422536149	-1.249931797154	-1.128290567036
N	0.709810733494	0.043963390445	-1.759332248310
P	0.030422231410	1.434044214218	-1.972677409132
Si	-3.083877272681	-2.436973511738	-3.730999776868
C	-1.788393380304	-2.239630910325	-5.079050962135
H	-1.739501795242	-1.143504267514	-5.142979292148
H	-0.838512472247	-2.528518081199	-4.605985618448
C	-2.703183125626	-1.327390705039	-2.295010135139
H	-3.129076184071	-1.713360959859	-1.366452915697
H	-1.630225035050	-1.196243873939	-2.161698231180
H	-3.119284257342	-0.334901397147	-2.467350396495
O	-2.803745172192	-4.010582464569	-2.798426511768
H	-2.269017234896	-3.893703438634	-1.899597674824
C	-1.844303295017	-2.795986055184	-6.527245648111
C	-2.854354358693	-5.391987904687	-3.140980867854
C	-1.882437132032	-4.352770134221	-6.472140567988
H	-2.700387572417	-4.654193785179	-5.806684305748
H	-0.955405329154	-4.680039166893	-5.986858687158
C	-4.117734952338	-7.390445793133	-3.420610692263
C	-2.970829784690	-8.028575764092	-3.888203217414

C	-1.747639688855	-7.358690125341	-3.895466823147
C	-1.656308055267	-6.019968910897	-3.497524862297
C	-4.079049916426	-6.052317958484	-3.005370224854
H	-5.055466831050	-7.935053105834	-3.347863159772
H	-3.021542232938	-9.064744447898	-4.210845229355
H	-0.844411431245	-7.877017472407	-4.202250359556
C	-0.707060827157	3.366782577388	-0.462992921735
C	2.046637940140	3.111100329508	-1.807557578498
C	2.832534874336	-0.433993803492	0.826486787910
C	3.538472445075	-2.506362000905	-1.426584199544
C	-2.097476411925	3.590624356038	-0.257032544042
C	3.758416296755	-3.683973395537	-2.207414151007
C	3.403008552554	2.754641831621	-2.059508683296
C	2.643070569174	0.700766510230	1.664266843837
C	0.083275341197	8.107194178232	-1.150850019521
C	0.580840853026	6.824053744673	-1.096808378871
C	-0.253735970793	5.729058092444	-0.742751108786
C	-1.639898301461	5.987573595510	-0.488595221748
C	-2.116784621801	7.326018231365	-0.534421947154
C	-1.275774799770	8.366512644736	-0.853433728526
H	0.739318445778	8.926917101088	-1.430461337098
H	1.621694512368	6.639652011082	-1.335898807709
C	0.219450582645	4.375301168665	-0.665637087441
C	-2.519141316970	4.906559516176	-0.236416525830
H	-3.168648381148	7.508525413011	-0.327689097036
H	-3.569446904008	5.120268804571	-0.057059828617
C	4.735786443687	5.652505065792	1.638633831850
C	5.055834655267	4.827301764083	0.585678305489
C	4.042693920899	4.255651183712	-0.231217987886
C	2.672466856177	4.588968372033	0.012558783391
C	2.375748782802	5.429962788489	1.122231731430
C	3.378155109020	5.940743403654	1.916866624051
H	5.414226355384	3.124968152304	-1.450021979614

H	6.091115968227	4.576711135286	0.372076354675
C	4.367938593590	3.353660767352	-1.270531280765
C	1.660315161499	4.028272511784	-0.839858001829
H	1.341892596860	5.662389131120	1.348558803688
H	3.123214153280	6.573002918512	2.762781117970
C	7.587884456303	0.432127534425	0.458476081512
C	6.516183660622	-0.393898126411	0.199573336461
C	5.219849997732	-0.088275399489	0.698265032354
C	5.049165847697	1.110315716652	1.463884884056
C	6.182337159509	1.921769009378	1.740331825076
C	7.424942735155	1.596026535068	1.247114678824
H	8.566438306305	0.186342397136	0.055518266804
H	6.652493838357	-1.282833223347	-0.405930192683
C	4.069734919402	-0.903822190802	0.427270267425
C	3.764872487620	1.453469898240	1.955224539430
H	6.039261068856	2.819355039071	2.334342366882
H	3.665523439940	2.327072163904	2.593072100320
C	6.560803686758	-5.362994764603	1.435425198815
C	5.996724233566	-5.510711875300	0.189751469277
C	5.195007629222	-4.481410192943	-0.374941917211
C	5.000894559550	-3.265854430299	0.353731927189
C	5.585578271767	-3.150788060669	1.644017367184
C	6.342261759609	-4.173999962693	2.171298581048
H	4.855039346283	-5.518167073041	-2.222754496766
H	6.145970211129	-6.424593677878	-0.380354154399
H	5.420509580895	-2.249361563953	2.222405286978
H	6.772064039242	-4.068442231941	3.163518149107
C	4.604750956482	-4.629867677688	-1.651589847670
C	4.199405224434	-2.233183446047	-0.240721194680
H	-1.654043303454	9.384255770602	-0.893152708468
H	5.518723644357	6.076816700055	2.261274889249
H	8.281193790353	2.230995529672	1.457674736111
H	7.165385468898	-6.159177934612	1.860786830035

C	0.324831432960	-4.167875358403	1.073877844415
F	1.239104546606	-3.480412250400	1.765157712009
F	-0.526217891823	-4.735629896803	1.935038467754
F	0.939920870007	-5.117103216645	0.362175345854
C	1.330462251488	1.008202943509	2.285188010259
C	0.586130102926	-0.004974159533	2.922848524491
C	0.871095453466	2.333902639353	2.334166265279
C	-1.040686118319	1.657974500651	3.618627302684
C	-0.572687642444	0.327739727068	3.607298737428
C	-0.319039607696	2.665363962761	2.977715768935
H	-0.676586196212	3.691625017065	2.969344543807
C	-2.299653943913	1.704151636565	4.365627581883
C	-2.568625099208	0.414529246668	4.864954141796
C	-3.181926758934	2.759410440875	4.601129087130
C	-4.338141968223	2.514766069758	5.345654038023
C	-4.604571247261	1.235414577997	5.846904113221
C	-3.717892653322	0.177818334615	5.608647286892
H	0.928437824763	-1.034106489792	2.886193631612
H	1.443454864757	3.104157572297	1.833626413897
H	-2.978628154442	3.750765046553	4.204198073829
H	-3.932710775165	-0.815043508791	5.996777679815
H	-5.510046265941	1.060353625067	6.421973217412
H	-5.039244626073	3.323535040212	5.534252510850
C	-1.462865982698	-0.562808704692	4.474379120459
C	-0.677717782778	-1.019613456046	5.727711931107
H	-0.275865813806	-0.160590103958	6.274742205998
H	-1.330700189334	-1.584188559896	6.403407742353
H	0.158311144547	-1.666646487265	5.438047211596
C	-2.016591320814	-1.793904905967	3.736492302650
H	-1.208594487973	-2.450819777278	3.401668797640
H	-2.671459916736	-2.368284622562	4.402620647095
H	-2.587345680172	-1.501272315702	2.854205604512
C	3.806524768910	1.811529321140	-3.132288108773

C	3.271969954733	1.897418116519	-4.433123137872
C	4.817822648730	0.869619223182	-2.864540114703
C	4.799816900807	0.149221021284	-5.154053308209
C	3.780395081371	1.081828202181	-5.433146914908
C	5.313776128061	0.033701335846	-3.861921079702
H	6.079716156408	-0.701197207849	-3.627422383599
C	5.103690404993	-0.592540534630	-6.380117747374
C	4.283888557785	-0.097224223660	-7.413661871790
C	5.969053874825	-1.663325880249	-6.607887658462
C	6.006373576864	-2.240069316460	-7.879297159166
C	5.196144301902	-1.747445814846	-8.908566342865
C	4.331835258801	-0.668882866927	-8.679698639445
H	2.475011009801	2.600913079933	-4.648338947575
H	5.204835478280	0.783900658866	-1.855595549288
H	6.584053584023	-2.059013196246	-5.803860171189
H	3.697734544354	-0.295061061407	-9.480384286886
H	5.227445273816	-2.215503144968	-9.888532350736
H	6.661873381494	-3.085560222153	-8.069439199152
C	3.394375975048	1.035425467882	-6.910006070450
C	3.745882753583	2.366746772790	-7.611540922704
H	4.806808726765	2.608590444010	-7.488979437106
H	3.528011357760	2.302887168860	-8.683998535986
H	3.152363060921	3.186284587977	-7.191231245825
C	1.898841121601	0.720437736202	-7.107729737845
H	1.273651022530	1.533691606222	-6.734349826722
H	1.677243055158	0.572989196629	-8.171586884008
H	1.605106371813	-0.182302642775	-6.565491028681
C	-2.124582239425	3.467946397084	-4.396426611276
F	-1.174057457280	4.215381838194	-3.821628173567
F	-2.267452930483	3.844805139683	-5.671226389438
F	-3.283747085590	3.680021585190	-3.753537838571
C	-3.068458413160	2.471812810940	-0.191409305756
C	-4.232228098171	2.561699175027	-0.978377439707

C	-2.830612202947	1.304617545796	0.557453447360
C	-4.844567023669	0.316869982585	-0.307997194832
C	-5.102842580630	1.487281366477	-1.044226856310
C	-3.718926039483	0.233131695536	0.512114862094
H	-3.489907047672	-0.675855391228	1.058049737029
C	-5.844510008887	-0.689732152468	-0.674875605789
C	-6.711192551896	-0.130186710769	-1.637745599210
C	-5.999866586292	-2.015440494997	-0.264057456714
C	-7.032745819085	-2.776731782624	-0.822013398094
C	-7.887857815635	-2.224902168723	-1.782884186641
C	-7.730364502728	-0.893913928429	-2.192279193248
H	-4.392509730335	3.438446095579	-1.596209958213
H	-1.931166159832	1.220730263371	1.152570895523
H	-5.329827317320	-2.447929036562	0.474979257072
H	-8.394076385927	-0.472012968959	-2.943096828911
H	-8.675214000080	-2.833596458741	-2.218664879804
H	-7.169066458937	-3.808751951185	-0.510453558854
C	-6.342979773892	1.325562493550	-1.917746650027
C	-6.024709958077	1.576216836422	-3.406171868902
H	-5.174401523697	0.980290157043	-3.745432961702
H	-6.897111309622	1.345768682503	-4.029902398466
H	-5.758514440653	2.625724907476	-3.570930881339
C	-7.475080897157	2.270401002711	-1.455346536330
H	-7.171843376365	3.316274805803	-1.580311615201
H	-8.382108576158	2.104318221734	-2.048442511634
H	-7.717798228057	2.105285754158	-0.400599209257
C	3.247811339277	-3.872041938226	-3.591666758673
C	3.098150797843	-5.175634818426	-4.121099856935
C	3.070209518383	-2.773924644609	-4.457296476001
C	2.743736625364	-4.245720476711	-6.330228334925
C	2.859826140845	-5.357807389205	-5.475327707274
C	2.819663094715	-2.955459670206	-5.814735840217
H	2.740877931338	-2.096006510054	-6.468528687206

C	2.585176416908	-4.717176599042	-7.705290521285
C	2.603645541538	-6.125295819903	-7.697569187808
C	2.435867161897	-4.008321128995	-8.898822583209
C	2.298950441282	-4.726259636889	-10.088775313931
C	2.314116574407	-6.126343333284	-10.083846379742
C	2.467688827525	-6.833999200416	-8.884919948288
H	3.182836835625	-6.038370511070	-3.465696853781
H	3.179412107789	-1.766933121578	-4.082287871998
H	2.429473366749	-2.923040725589	-8.897972922726
H	2.474533630069	-7.921416949029	-8.887796226966
H	2.202984034515	-6.669137278383	-11.018695031551
H	2.175000165923	-4.193306994000	-11.027643017192
C	2.762361129924	-6.659379906950	-6.275070932582
C	1.534314824592	-7.504266055048	-5.875941093553
H	0.605365058096	-6.947522139778	-6.034473551141
H	1.485299521244	-8.415890339276	-6.482633727776
H	1.595523702750	-7.801653515515	-4.822080525959
C	4.050695652634	-7.495169417102	-6.125633389171
H	4.176140530315	-7.827040488632	-5.088144649079
H	4.010630040745	-8.384923199037	-6.764513512537
H	4.930536654502	-6.908104383804	-6.407469921482
C	-5.282126756803	-5.393554950255	-2.376506594854
H	-5.885207224010	-6.142532143729	-1.853542197617
H	-4.984477672338	-4.630065175812	-1.654583451084
H	-5.929520052838	-4.909441773321	-3.114284793069
C	-0.323415190071	-5.330936870801	-3.361410356075
H	-0.364252238755	-4.266897183044	-3.591396346495
H	0.034210137929	-5.404664483388	-2.327819735937
H	0.424493069928	-5.783919615669	-4.010803320516
C	-4.937128610814	-2.286988361352	-4.016280632044
H	-5.037469458082	-1.193872775581	-4.042276547279
H	-5.372914847704	-2.562561096993	-3.045675392447
C	-5.769634198648	-2.872259144517	-5.131292656989

C	-6.755436418260	-2.071130644570	-5.728979792589
C	-5.644861453922	-4.196594191101	-5.573461940479
C	-7.589880912796	-2.574624224051	-6.726228865858
H	-6.864601219950	-1.039026831906	-5.405435377252
C	-6.484745791411	-4.707953305020	-6.564238318264
H	-4.872988821981	-4.836068445999	-5.158127314371
C	-7.461730054110	-3.900241187089	-7.146078125047
H	-8.339992366059	-1.929598455403	-7.176581820611
H	-6.360373796859	-5.736882185398	-6.890352777712
H	-8.110382433826	-4.296119728492	-7.922707830937
C	-3.047743335480	-2.202831245928	-7.279612716106
H	-3.008915512918	-2.475889257878	-8.339880431271
H	-4.001733833837	-2.550845925816	-6.879104824843
H	-3.033456162359	-1.108654028668	-7.211502410829
C	-0.558381257825	-2.332185682065	-7.239801962923
H	-0.504823378724	-1.237772388236	-7.258147102694
H	0.332364874228	-2.706044004815	-6.724602974836
H	-0.529273255683	-2.699754714539	-8.270466307314
C	-2.043289312193	-5.097619793302	-7.784872015801
C	-0.988415545980	-5.449276748659	-8.528225470146
H	0.027052944834	-5.210632397322	-8.234318289341
H	-1.103825108432	-5.988449034751	-9.465542856030
C	-3.449237317624	-5.479715889168	-8.178020783673
H	-3.878102690424	-6.162131801600	-7.430161931714
H	-4.116649724479	-4.612427104047	-8.215323881106
H	-3.477196351091	-5.981513626956	-9.150962513032

CD experiments for determination of the absolute configuration

Method:

The CD-spectra of (*R*)-**4b** (c = 1.0 x 10^{-4} M), (*S*)-**4b** (c = 1.0 x 10^{-4} M) and (*R*)-**4d** (c = 1.0 x 10^{-4} M) were recorded in hexane (HPLC grade) at 20 °C and compared with the corresponding TD-DFT calculated CD spectra of the possible conformers. After a UV correction of −20 nm to −24 nm and a correction of the σ-value of 0.3 eV, the CD characteristics of the calculated spectra (blue curves) were in good agreement with the experimental spectra (red curves), thus allowing the assignment of the absolute configuration of both

enantiomers of chiral silicon compounds **4b**, and the absolute configuration of **4d** that was generated from the catalytic reaction could be assigned to be *R*, using (*S*, *S*)-IDPi as catalyst.

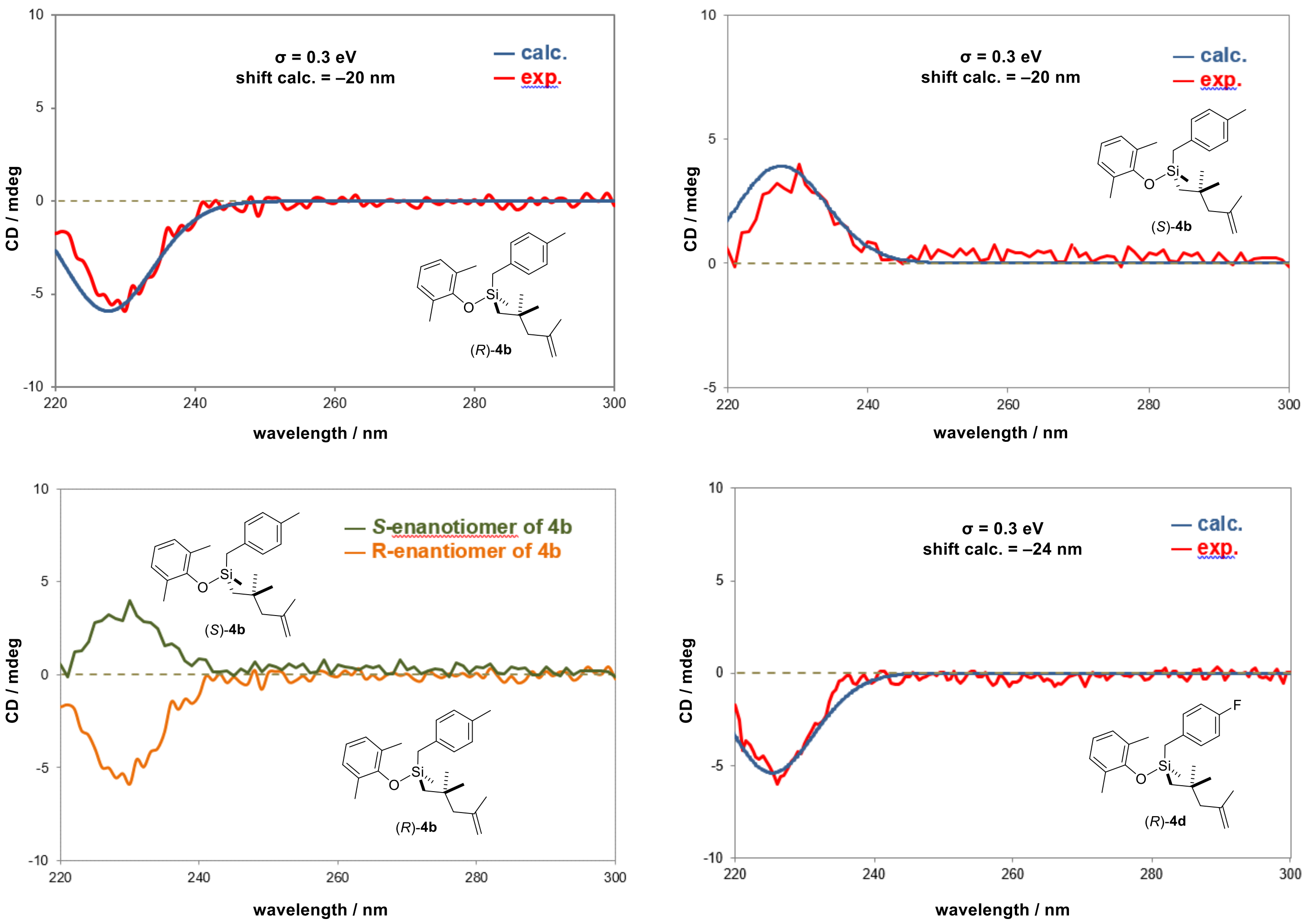

Figure S33. Comparison between the experimental CD-spectra (red curves) and the calculated spectra (blue curves).

CD spectrum calculation

Density Functional Theory (DFT) calculations were performed on the Max-Planck-Institut für Kohlenforschung computer cluster using the ORCA program package (Version 5.0-Stable).[42] Structural optimizations and frequency calculations to identify all of the stationary points as minima (zero imaginary frequencies) and to obtain thermal and entropic correction were performed with the B3LYP functional[43,44] with D3 dispersion correction[45] and Becke-Johnson damping (BJ)[46] along with RI approximation, utilizing the def2/J auxiliary basis set[47] and the def2-SVP basis set[48] on all atoms. The libint2 library was used for the computation of 2-el integrals.[49] Tight SCF convergence and geometry optimization criterions were chosen.

The CD spectrum was computed by time-dependent density functional theory (TD-DFT, NROOTS = 25) at B3LYP/TZVP level, solvent effects of hexane were taken into account using the conductor-like polarized continuum model (CPCM).[50] The CD spectrum was created using Multiwfn[51] with a 0.3 eV half-width at half-height.

Calculated coordinates:

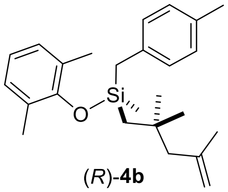

(*R*)-**4b**

C	2.75206400	-1.94094000	0.57172000
C	1.42299500	-1.69594400	0.92926900
C	0.36024300	-2.20025900	0.16643600
C	0.67915800	-2.96523800	-0.96933000
C	2.00481200	-3.20566100	-1.32523800
C	3.07011900	-2.70047600	-0.56132400
C	4.50027800	-2.97576700	-0.95395600
C	-1.07094800	-1.99712500	0.58943400
Si	-1.75899600	-3.50704600	1.50369800
C	-3.56618300	-3.14885900	1.92188700
C	-0.62779000	-3.88874300	2.95117000
O	-1.64035200	-4.74110400	0.35181300
C	-2.36677200	-5.83760200	0.00166900
C	-3.43092200	-5.71217200	-0.91323700
C	-1.99625400	-7.09162700	0.52961700
C	-4.16482200	-6.86242300	-1.23691700
C	-2.75302500	-8.21415100	0.17598100
C	-3.84266600	-8.10449900	-0.69094100
C	-3.78054300	-4.38210400	-1.52785100
C	-0.81718900	-7.19583000	1.45868300
C	-4.37082100	-3.96076000	2.96577000
C	-5.88798300	-3.60294400	2.85795000
C	-3.91548000	-3.58995300	4.38725600
C	-4.17498600	-5.46957500	2.75651300
C	-6.51169300	-3.69242800	1.47712100
C	-6.88202300	-4.85970100	0.93048400
C	-6.72319500	-2.38636800	0.75271500
H	3.55810500	-1.52912800	1.18623400
H	1.20801700	-1.09814700	1.82008300

H	-0.12754600	-3.38593500	-1.57367800
H	2.21969900	-3.80257100	-2.21695500
H	4.70339100	-2.64959000	-1.98780200
H	4.72796700	-4.05463500	-0.90785800
H	5.20837100	-2.45594300	-0.29132500
H	-1.72088600	-1.80739900	-0.28148500
H	-1.16026400	-1.12144000	1.25439600
H	-4.06550900	-3.22816800	0.94438200
H	-3.61291900	-2.07584800	2.18645200
H	-0.58644400	-3.02432200	3.63476500
H	0.39194700	-4.07370200	2.57870200
H	-0.94996800	-4.76579300	3.53038200
H	-4.99926100	-6.77438700	-1.93851100
H	-2.47712200	-9.18935800	0.58719100
H	-4.42639900	-8.98953800	-0.95528400
H	-2.89923300	-3.72971200	-1.60299500
H	-4.54088500	-3.85106000	-0.93096700
H	-4.20121900	-4.51706900	-2.53555500
H	-1.06273200	-6.82547800	2.46871300
H	0.02702400	-6.58867400	1.09811500
H	-0.48733000	-8.23984400	1.56205900
H	-6.43376900	-4.26310000	3.55296200
H	-6.01991900	-2.57594700	3.23887500
H	-2.86189800	-3.85632000	4.55547800
H	-4.51822600	-4.12110100	5.14206200
H	-4.02444700	-2.50818700	4.57068000
H	-3.12402900	-5.75549000	2.91294800
H	-4.45427200	-5.78377700	1.74321700
H	-4.78107400	-6.04518000	3.47504400
H	-6.77062400	-5.80814000	1.46129600
H	-7.31780400	-4.90497500	-0.07207100
H	-5.78630800	-1.81022000	0.67324300
H	-7.43042800	-1.74814000	1.31239100

H -7.12593400 -2.53659100 -0.26012000

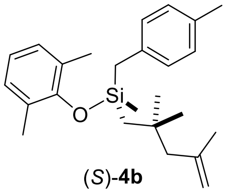

(*S*)-**4b**

C -2.75206400 1.94094000 -0.57172000

C -1.42299500 1.69594400 -0.92926900

C -0.36024300 2.20025900 -0.16643600

C -0.67915800 2.96523800 0.96933000

C -2.00481200 3.20566100 1.32523800

C -3.07011900 2.70047600 0.56132400

H -3.55810500 1.52912800 -1.18623400

H -1.20801700 1.09814700 -1.82008300

H 0.12754600 3.38593500 1.57367800

H -2.21969900 3.80257100 2.21695500

C -4.50027800 2.97576700 0.95395600

H -4.70339100 2.64959000 1.98780200

H -4.72796700 4.05463500 0.90785800

H -5.20837100 2.45594300 0.29132500

C 1.07094800 1.99712500 -0.58943400

H 1.72088600 1.80739900 0.28148500

H 1.16026400 1.12144000 -1.25439600

Si 1.75899600 3.50704600 -1.50369800

C 3.56618300 3.14885900 -1.92188700

H 4.06550900 3.22816800 -0.94438200

H 3.61291900 2.07584800 -2.18645200

C 0.62779000 3.88874300 -2.95117000

H 0.58644400 3.02432200 -3.63476500

H -0.39194700 4.07370200 -2.57870200

H 0.94996800 4.76579300 -3.53038200

O 1.64035200 4.74110400 -0.35181300

C 2.36677200 5.83760200 -0.00166900

C 3.43092200 5.71217200 0.91323700

C 1.99625400 7.09162700 -0.52961700

C 4.16482200 6.86242300 1.23691700

C 2.75302500 8.21415100 -0.17598100

C 3.84266600 8.10449900 0.69094100

H 4.99926100 6.77438700 1.93851100

H 2.47712200 9.18935800 -0.58719100

H 4.42639900 8.98953800 0.95528400

C 3.78054300 4.38210400 1.52785100

H 2.89923300 3.72971200 1.60299500

H 4.54088500 3.85106000 0.93096700

H 4.20121900 4.51706900 2.53555500

C 0.81718900 7.19583000 -1.45868300

H 1.06273200 6.82547800 -2.46871300

H -0.02702400 6.58867400 -1.09811500

H 0.48733000 8.23984400 -1.56205900

C 4.37082100 3.96076000 -2.96577000

C 5.88798300 3.60294400 -2.85795000

H 6.43376900 4.26310000 -3.55296200

H 6.01991900 2.57594700 -3.23887500

C 3.91548000 3.58995300 -4.38725600

H 2.86189800 3.85632000 -4.55547800

H 4.51822600 4.12110100 -5.14206200

H 4.02444700 2.50818700 -4.57068000

C 4.17498600 5.46957500 -2.75651300

H 3.12402900 5.75549000 -2.91294800

H 4.45427200 5.78377700 -1.74321700

H 4.78107400 6.04518000 -3.47504400

C 6.51169300 3.69242800 -1.47712100

C 6.88202300 4.85970100 -0.93048400

H 6.77062400 5.80814000 -1.46129600

H 7.31780400 4.90497500 0.07207100

C 6.72319500 2.38636800 -0.75271500

H 5.78630800 1.81022000 -0.67324300

H 7.43042800 1.74814000 -1.31239100

H 7.12593400 2.53659100 0.26012000

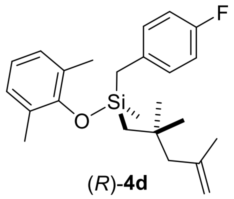

(*R*)-**4d**

C 2.76371200 -1.92893800 0.57889300

C 1.4327300 -1.68914600 0.93050600

C 0.36886700 -2.20294200 0.17140300

C 0.68093100 -2.97701100 -0.96013100

C 2.00419100 -3.22403100 -1.32797900

C 3.03248900 -2.69635800 -0.55047000

F 4.30884700 -2.93191300 -0.89862100

C -1.06124000 -1.99807400 0.59835100

Si -1.75498700 -3.51074100 1.50630000

C -3.56305000 -3.15294500 1.91738500

C -0.62678300 -3.89765500 2.95486500

O -1.63013700 -4.73669000 0.34597500

C -2.36021600 -5.83336300 -0.00139000

C -3.42845300 -5.70610400 -0.91098700

C -1.98887300 -7.08757500 0.52545200

C -4.16599900 -6.85545000 -1.23031300

C -2.74925600 -8.20887700 0.17585100

C -3.84313200 -8.09763800 -0.68549800

C -3.78188400 -4.37680400 -1.52541300

C -0.80610200 -7.19447600 1.44949600

C -4.36884400 -3.96406500 2.96113600

C -5.88561500 -3.60553600 2.85155200

C -3.91491700 -3.59290800 4.38298800

C -4.17373900 -5.47307600 2.75233800

C -6.50785600 -3.69472800 1.47014100

C -6.88039000 -4.86191500 0.92477300

C -6.71629200 -2.38899000 0.74427400

H 3.59246600 -1.52707000 1.16514300

H 1.21762700 -1.08637600 1.81727800

H -0.12803600 -3.40483300 -1.55538000

H 2.25027000 -3.82288300 -2.20712000

H -1.71098500 -1.80038900 -0.27079200

H -1.14625700 -1.12487300 1.26685600

H -4.05968000 -3.23567500 0.93879000

H -3.61193600 -2.07941800 2.17935700

H -0.58424900 -3.03590700 3.64172400

H 0.39302500 -4.08570100 2.58390800

H -0.95185500 -4.77572100 3.53077600

H -5.00403000 -6.76636700 -1.92745100

H -2.47299500 -9.18445800 0.58582400

H -4.42957300 -8.98182300 -0.94655000

H -2.90911400 -3.71096500 -1.57501200

H -4.56531000 -3.86219600 -0.94415400

H -4.17514100 -4.51096400 -2.54432900

H -1.04877800 -6.83117100 2.46279700

H 0.03595200 -6.58410900 1.08953300

H -0.47389800 -8.23841200 1.54540700

H -6.43214900 -4.26575200 3.54583500

H -6.01765200 -2.57857600 3.23261100

H -2.86330100 -3.86455700 4.55501700

H -4.52244300 -4.11956800 5.13705100

H -4.01884000 -2.51031300 4.56443500

H -3.12284200 -5.75981300 2.90818600

H -4.45386600 -5.78785200 1.73943900

H -4.77948300 -6.04787700 3.47172200

H -6.77172900 -5.80953500 1.45763600

H -7.31559600 -4.90795700 -0.07797200

H -5.77952000 -1.81226100 0.66804400

H -7.42605600 -1.75094600 1.30097300

H -7.11543000 -2.53976100 -0.26990600

12. References

1. Zhang, G.; Li, Y.; Wang, Y.; Zhang, Q.; Xiong, T.; Zhang, Q., Asymmetric Synthesis of Silicon-Stereogenic Silanes by Copper-Catalyzed Desymmetrizing Protoboration of Vinylsilanes. *Angew. Chem. Int. Ed*. **2020**, *59*, 11927–11931.

2. Bae, H. Y.; Höfler, D.; Kaib, P. S.; Kasaplar, P.; De, C. K.; Döhring, A.; Lee, S.; Kaupmees, K.; Leito, I.; List, B., Approaching Sub-PPM-Level Asymmetric Organocatalysis of a Highly Challenging and Scalable Carbon–Carbon Bond Forming Reaction. *Nat. Chem*. **2018**, *10*, 888–894.

3. Gatzenmeier, T.; Kaib, P.S.; Lingnau, J.B.; Goddard, R.; List, B., The Catalytic Asymmetric Mukaiyama–Michael Reaction of Silyl Ketene Acetals with α, β-Unsaturated Methyl Esters. *Angew. Chem. Int. Ed*. **2018**, *57*, 2464–2468.

4. Fulmer, G.R.; Miller, A.J.; Sherden, N.H.; Gottlieb, H.E.; Nudelman, A.; Stoltz, B.M.; Bercaw, J.E.; Goldberg, K.I., NMR Chemical Shifts of Trace Impurities: Common Laboratory Solvents, Organics, and Gases in Deuterated Solvents Relevant to the Organometallic Chemist. *Organometallics* **2010**, *29*, 2176–2179.

5. Hoffman, R. E., Standardization of Chemical Shifts of TMS and Solvent Signals in NMR Solvents. *Magn. Reson. Chem*. **2006**, *44*, 606–616.

6. Sainz, M. F.; Souto, J. A.; Regentova, D.; Johansson, M. K. G.; Timhagen, S. T.; Irvine, D. J.; Buijsen, P.; Konig, C. E.; Stockman, R. A.; Howdle, S. M., A Facile and Green Route to Terpene Derived Acrylate and Methacrylate Monomers and Simple Free Radical Polymerisation to Yield New Renewable Polymers and Coatings. *Polym. Chem.* **2016**, *7*, 2882–2887.

7. Andersen, C.; Ferey, V.; Daumas, M.; Bernardelli, P.; Guerinot, A.; Cossy, J., Introduction of Cyclopropyl and Cyclobutyl Ring on Alkyl Iodides through Cobalt-Catalyzed Cross-Coupling. *Org. Lett.* **2019**, *21*, 2285–2289.

8. Taber, D.F.; Paquette, C.M.; Gu, P.; Tian, W., Cyclohexanones by Rh-Mediated Intramolecular C–H Insertion. *J. Org. Chem*. **2013**, *78*, 9772–9780.

9. Rendler, S.; Oestreich, M., Conclusive Evidence for an S_N2-Si Mechanism in the $B(C_6F_5)_3$-Catalyzed Hydrosilylation of Carbonyl Compounds: Implications for the Related Hydrogenation. *Angew. Chem. Int. Ed.* **2008**, *47*, 5997–6000.

10. Zhan, G.; Teng, H.-L.; Luo, Y.; Lou, S.-J.; Nishiura, M.; Hou, Z., Enantioselective Construction of Silicon-Stereogenic Silanes by Scandium-Catalyzed Intermolecular Alkene Hydrosilylation. *Angew. Chem. Int. Ed.* **2018**, *57*, 12342–2346.

11. Shintani, R.; Maciver, E. E.; Tamakuni, F.; Hayashi, T., Rhodium-Catalyzed Asymmetric Synthesis of Silicon-Stereogenic Dibenzooxasilines via Enantioselective Transmetalation. *J. Am. Chem. Soc.* **2012**, *134*, 16955–16958.

12. Jeon, M.; Han, J.; Park, J., Transformation of Silanes into Silanols using Water and Recyclable Metal Nanoparticle Catalysts. *ChemCatChem* **2012**, *4*, 521–524.

13. Wang, Z.-C.; Wang, M.; Gao, J.; Shi, S.-L.; Xu, Y., ^{n}BuLi-promoted *anti*-Markovnikov Selective Hydroboration of Unactivated Alkenes and Internal Alkynes. *Org. Chem. Front.* **2019**, *6*, 2949–2953.

14. Schwengers, S.A.; De, C.K.; Grossmann, O.; Grimm, J.A.; Sadlowski, N.R.; Gerosa, G.G.; List, B., Unified Approach to Imidodiphosphate-Type Brønsted Acids with Tunable Confinement and Acidity. *J. Am. Chem. Soc.* **2021**, *143*, 14835–14844.

15. Seidel, R. W.; Goddard, R.; Nöthling, N.; Lehmann, C. W., In Situ Cryocrystallization and Solid-State Structures of Furfural and Some Derivatives. *CrystEngComm* **2019**, *21*, 3295–3303.

16. Buchsteiner, M.; Martinez-Rodriguez, L.; Jerabek, P.; Pozo, I.; Patzer, M.; Nöthling, N.; Lehmann, C. W.; Fürstner, A., Catalytic Asymmetric Fluorination of Copper Carbene Complexes: Preparative Advances and a Mechanistic Rationale. *Chem. Eur. J.* **2020**, *26*, 2509–2515.

17. Patzer, M.; Nöthling, N.; Goddard, R.; Lehmann, C. W., Absolute Configuration of In Situ Crystallized (+)-γ-Decalactone. *Chemistry* **2021**, *3*, 578–584.

18. F. Krupp, W. Frey, C. Richert, Absolute Configuration of Small Molecules by Co-Crystallization. *Angew. Chem. Int. Ed.* **2020**, *59*, 15875–15879.

19. C. Richert, F. Krupp, M.-I. Picher, W. Frey, B. Plietker, Determining the Relative Configuration of Propargyl Cyclopropanes by Co-Crystallization. *Synlett* **2020**, *32*, 350–353.

20. E. Christoforides, A. Papaioannou, K. Bethanis, Crystal Structure of the Inclusion Complex of Cholesterol in β-Cyclodextrin and Molecular Dynamics Studies. *Beilstein J. Org. Chem.* **2018**, *14*, 838–848.

21. A. Sala, Z. Hoossen, A. Bacchi, M. R. Caira, Two Crystal Forms of a Hydrated 2:1 β-Cyclodextrin Fluconazole Complex: Single Crystal X-ray Structures, Dehydration Profiles, and Conditions for Their Individual Isolation. *Molecules* **2021**, *26*, 1–14.

22. C. R. Clough, J. B. Greco, J. S. Figueroa, P. L. Diaconescu, W. M. Davis, and C. C. Organic Nitriles from Acid Chlorides: An Isovalent N for (O)Cl Exchange Reaction Mediated by a Tungsten Nitride Complex.Cummins, *J. Am. Chem. Soc.* **2004**, *126*, 7742–7743.

23. Inokuma, Y.; Yoshioka, S.; Ariyoshi, J.; Arai, T.; Hitora, Y.; Takada, K.; Matsunaga, S.; Rissanen, K.; Fujita, M., X-Ray Analysis on the Nanogram to Microgram Scale Using Porous Complexes. *Nature* **2013**, *495*, 461–466.

24. Dubey, R.; Yan, K.; Kikuchi, T.; Sairenji, S.; Rossen, A.; Goh, S. S.; Feringa, B. L.; Fujita, M., Absolute Configuration Determination from Low ee Compounds by the Crystalline Sponge Method. Unusual Conglomerate Formation in a Pre-Determined Crystalline Lattice. *Angew. Chem. Int. Ed.* **2021**, *60*, 11809–11813.

25. Kabsch, W., Integration, Scaling, Space-Group Assignment and Post-Refinement. *Acta Crystallogr., Sect. D: Biol. Crystallogr.* **2010**, *66*, 133–144.

26. Macrae, C. F.; Sovago, I.; Cottrell, S. J.; Galek, P. T.; McCabe, P.; Pidcock, E.; Platings, M.; Shields, G. P.; Stevens, J. S.; Towler, M., Mercury 4.0: From Visualization to Analysis, Design and Prediction. *J. Appl. Crystallogr*. **2020**, *53*, 226–235.

27. System, C. S., Rigaku Oxford Diffraction. CrysAlisPro Software System: **2021**, Rigaku Corporation, Oxford, UK.

28. Rosenberger, L.; von Essen, C.; Khutia, A.; Kühn, C.; Georgi, K.; Hirsch, A. K.; Hartmann, R. W.; Badolo, L., Crystalline Sponge Affinity Screening: A Fast Tool for Soaking Condition Optimization without the Need of X-ray Diffraction Analysis. *Eur. J. Pharm. Sci.* **2021**, *164*, 105884.

29. Flack, H. D.; Bernardelli, G., The Use of X-ray Crystallography to Determine Absolute Configuration. *Chirality* **2008**, *20*, 681–690.

30. Maeda, S.; Harabuchi, Y.; Takagi, M.; Taketsugu, T.; Morokuma, K., Artificial force induced reaction (AFIR) method for exploring quantum chemical potential energy surfaces. *Chem. Rec.* **2016**, *16*, 2232–2248.

31. Maeda, S.; Ohno, K.; Morokuma, K., Systematic exploration of the mechanism of chemical reactions: the global reaction route mapping (GRRM) strategy using the ADDF and AFIR methods. *Phys. Chem. Chem. Phys.* **2013**, *15*, 3683–3701.

32. Grimme, S.; Bannwarth, C.; Shushkov, P., A robust and accurate tight-binding quantum chemical method for structures, vibrational frequencies, and noncovalent interactions of large molecular systems parametrized for all spd-block elements (Z = 1–86). *J. Chem. Theory Comput.* **2017**, *13*, 1989–2009.

33. Bannwarth, C.; Ehlert, S.; Grimme, S., GFN2-xTB—An accurate and broadly parametrized self-consistent tight-binding quantum chemical method with multipole electrostatics and density-dependent dispersion contributions. *J. Chem. Theory Comput.* **2019**, *15*, 1652–1671.

34. Maeda, S.; Harabuchi, Y.; Takagi, M.; Saita, K.; Suzuki, K.; Ichino, T.; Sumiya, Y.; Sugiyama, K.; Ono, Y., Implementation and performance of the artificial force induced reaction method in the GRRM17 program. *J. Comput. Chem.* **2018**, *39*, 233−250.

35. Neese, F., WIREs Comput. Mol. Sci. *Wiley Interdiscip. Rev. Comput. Mol. Sci.* **2012**, *2*, 73−78.

36. Frisch, M.; Trucks, G.; Schlegel, H.; Scuseria, G.; Robb, M.; Cheeseman, J.; Scalmani, G.; Barone, V.; Petersson, G.; Nakatsuji, H., Gaussian 16 revision a. 03. 2016; gaussian inc. *Wallingford CT* **2016**, *2*.

37. Becke, A., Density-functional thermochemistry. III. The role of exact exchange. *J. Chem. Phys*. **1993**, *98*, 5648−5652.

38. Lee, C.; Yang, W.; Parr, R. G., Development of the Colle-Salvetti correlation-energy formula into a functional of the electron density. *Phys. Rev. B* **1988**, *37*, 785–789.

39. Chai, J.-D.; Head-Gordon, M., Long-range corrected hybrid density functionals with damped atom–atom dispersion corrections. *Phys. Chem. Chem. Phys.* **2008**, *10*, 6615–6620.

40. Cossi, M.; Rega, N.; Scalmani, G.; Barone, V., Energies, structures, and electronic properties of molecules in solution with the C-PCM solvation model. *J. Comput. Chem.* **2003**, *24*, 669–681.

41. Pettersen, E. F.; Goddard, T. D.; Huang, C. C.; Couch, G. S.; Greenblatt, D. M.; Meng, E. C.; Ferrin, T. E., UCSF Chimera—a visualization system for exploratory research and analysis. *J. Comput. Chem.* **2004**, *25*, 1605–1612.

42. Neese, F., The ORCA program system. *WIREs Comput. Mol. Sci.* **2012**, *2*, 73–78.

43. Becke, A. D., Density-functional thermochemistry. III. The role of exact exchange. *J. Chem. Phys*. **1993**, *98*, 5648–5652.

44. Lee, C.; Yang, W.; Parr, R. G., Development of the Colle-Salvetti correlation-energy formula into a functional of the electron density. *Phys. Rev. B* **1988**, *37*, 785–789.

45. Grimme, S.; Antony, J.; Ehrlich, S; Krieg, H., A consistent and accurate ab initio parametrization of density functional dispersion correction (DFT-D) for the 94 elements H–Pu. *J. Chem. Phys.* **2010**, *132*, 154104.

46. Grimme, S.; Ehrlich, S.; Goerigk, L., Effect of the damping function in dispersion corrected density functional theory. *J. Comput. Chem.* **2011**, *32*, 1456–1465.

47. Weigend, F., Accurate Coulomb-fitting basis sets for H to Rn. *Phys. Chem. Chem. Phys.* **2006**, *8*, 1057–1065.

48. Weigend, F; Ahlrichs, R., Balanced basis sets of split valence, triple zeta valence and quadruple zeta valence quality for H to Rn: Design and assessment of accuracy. *Phys. Chem. Chem. Phys.* **2005**, *7*, 3297–3305.

49. Valeev, E. F., Libint: A library for the evaluation of molecular integrals of many-body operators over Gaussian functions, *http://libint.valeyev.net/*

50. Barone, V.; Cossi, M., Quantum calculation of molecular energies and energy gradients in solution by a conductor solvent model. *J. Phys. Chem. A* **1998**, *102*, 1995–2001.

51. Lu, T.; Chen, F., Multiwfn: A multifunctional wavefunction analyzer. *J. Comput. Chem.* **2012**, *33*, 580-592.

13. Copies of NMR spectra

benzyl(methyl)bis(2-methylallyl)silane 1a:

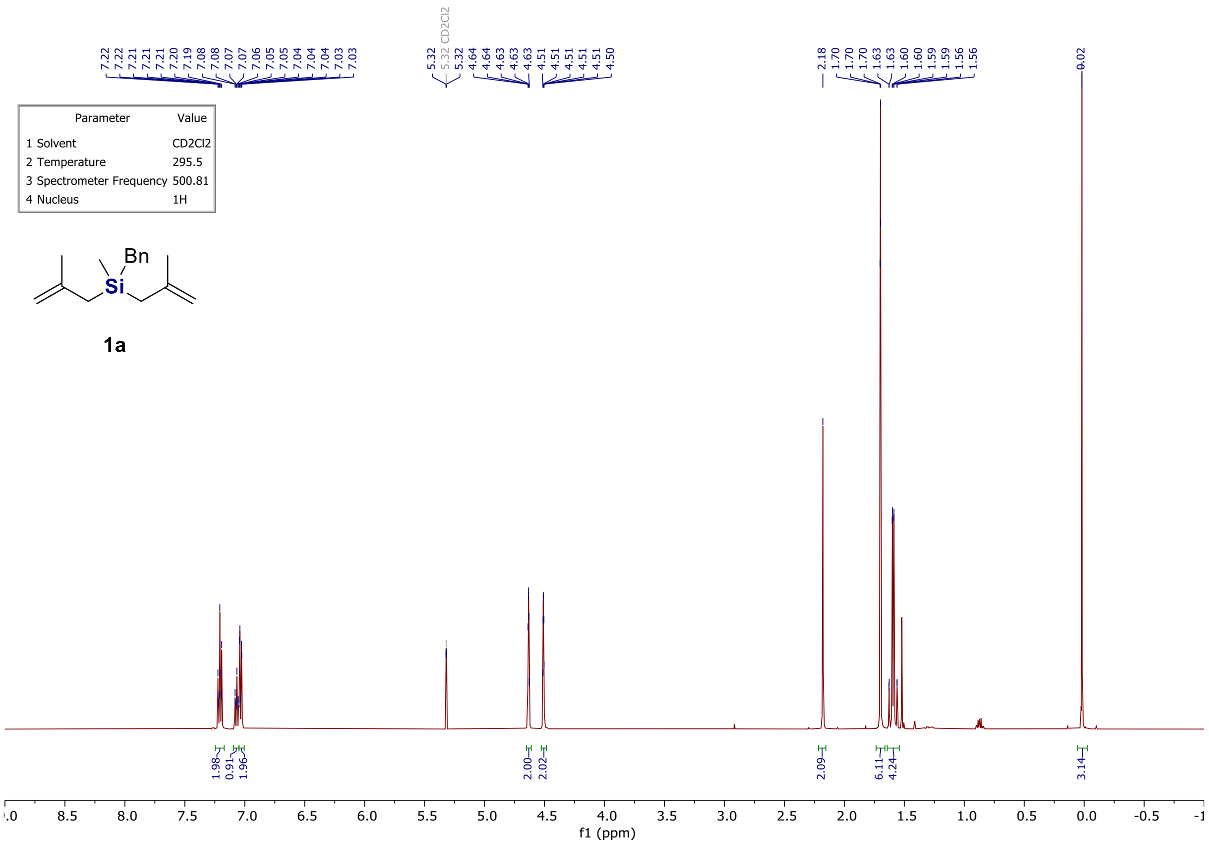

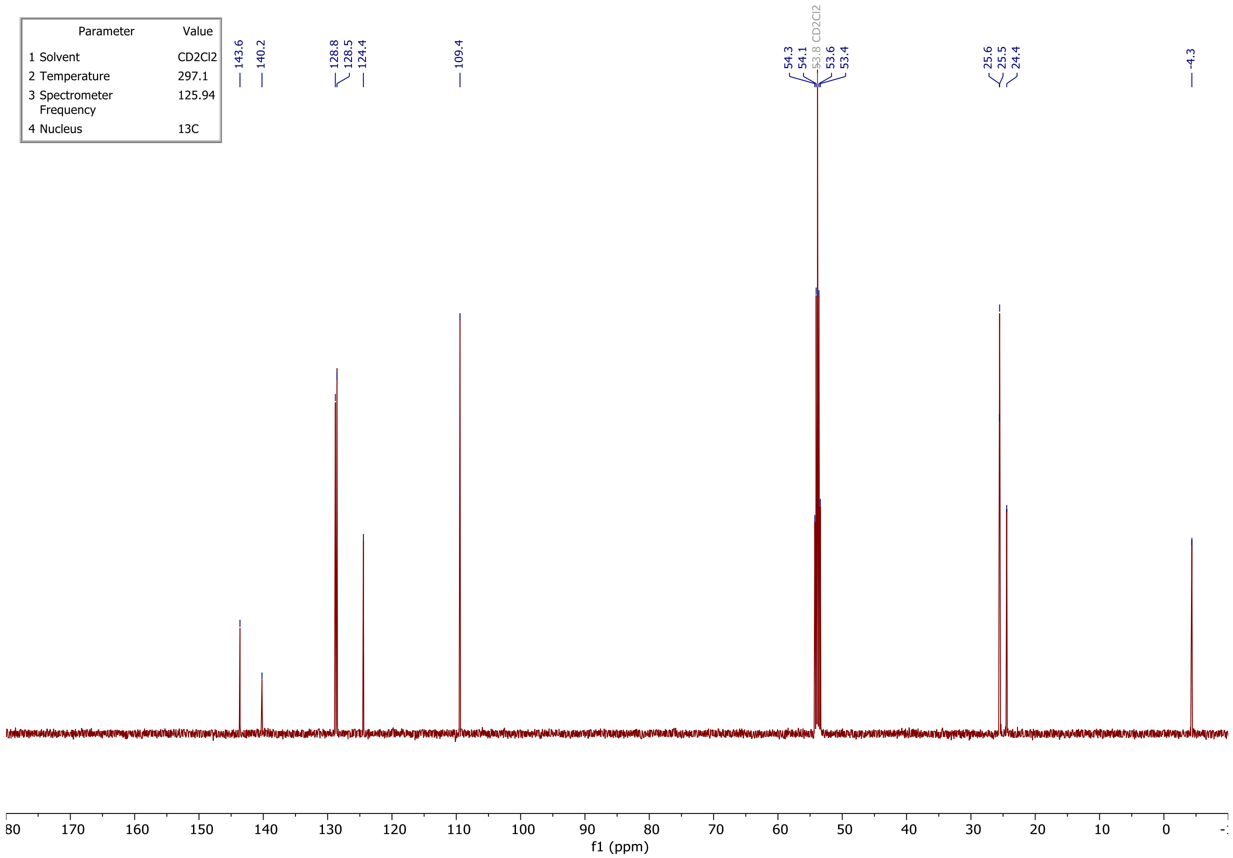

methylbis(2-methylallyl)(4-methylbenzyl)silane 1b

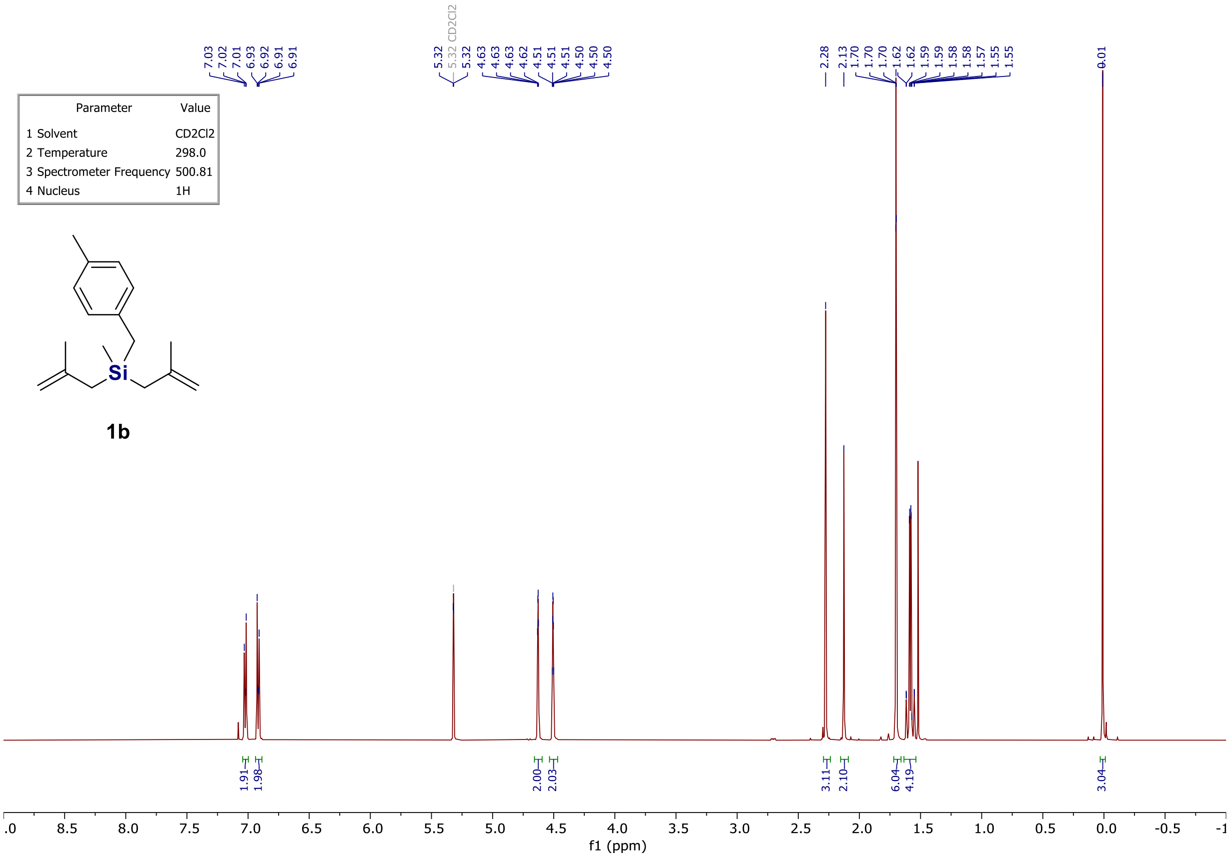

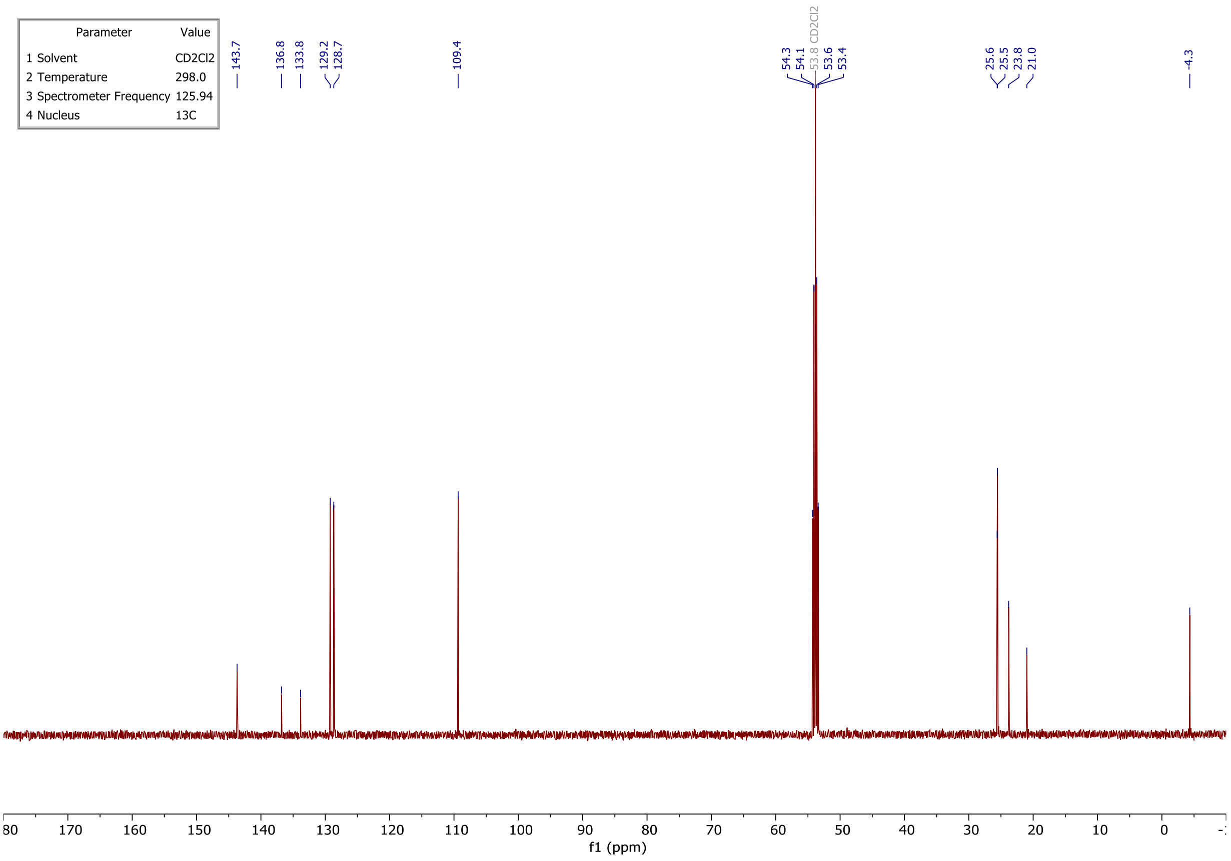

(4-methoxybenzyl)(methyl)bis(2-methylallyl)silane 1c

Parameter	Value
1 Solvent	CD2Cl2
2 Temperature	298.0
3 Spectrometer Frequency	500.81
4 Nucleus	1H

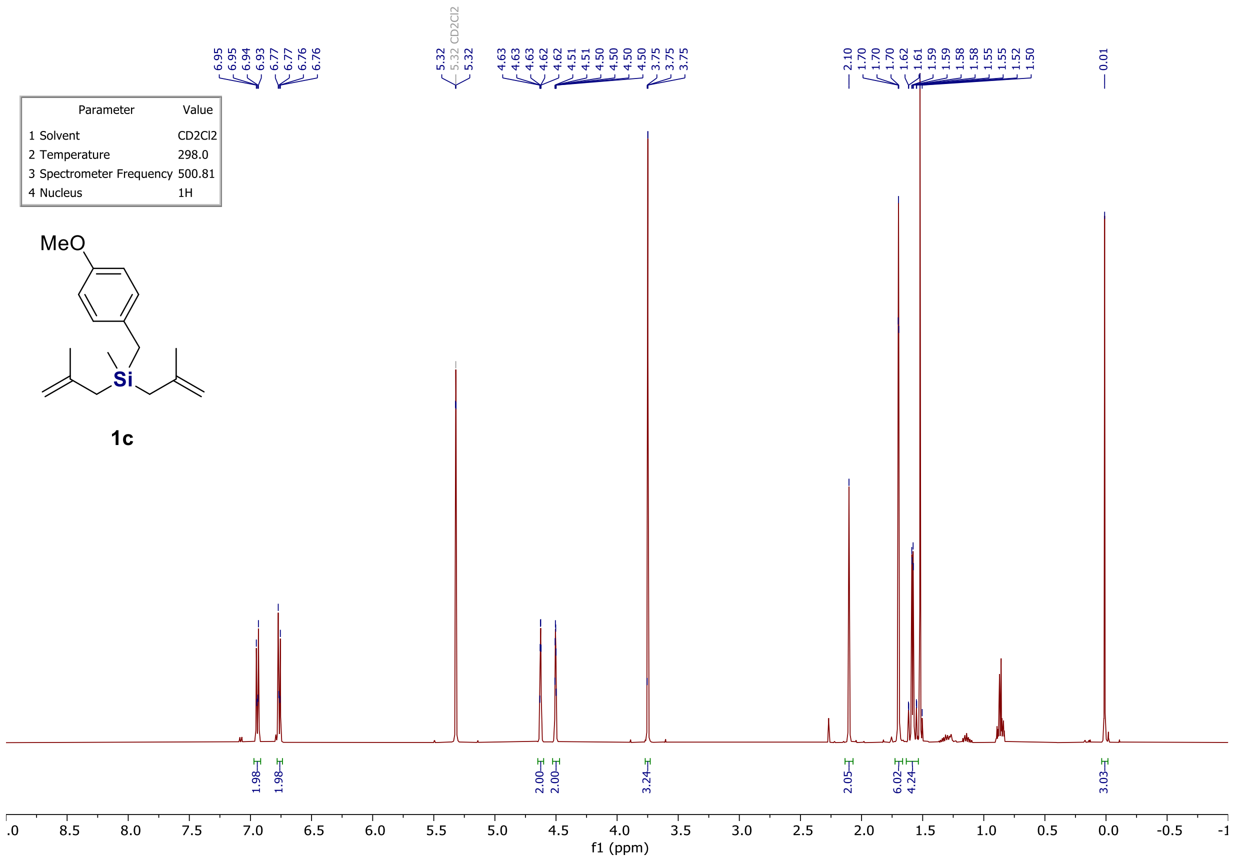

Parameter	Value
1 Solvent	CD2Cl2
2 Temperature	298.0
3 Spectrometer Frequency	125.94
4 Nucleus	13C

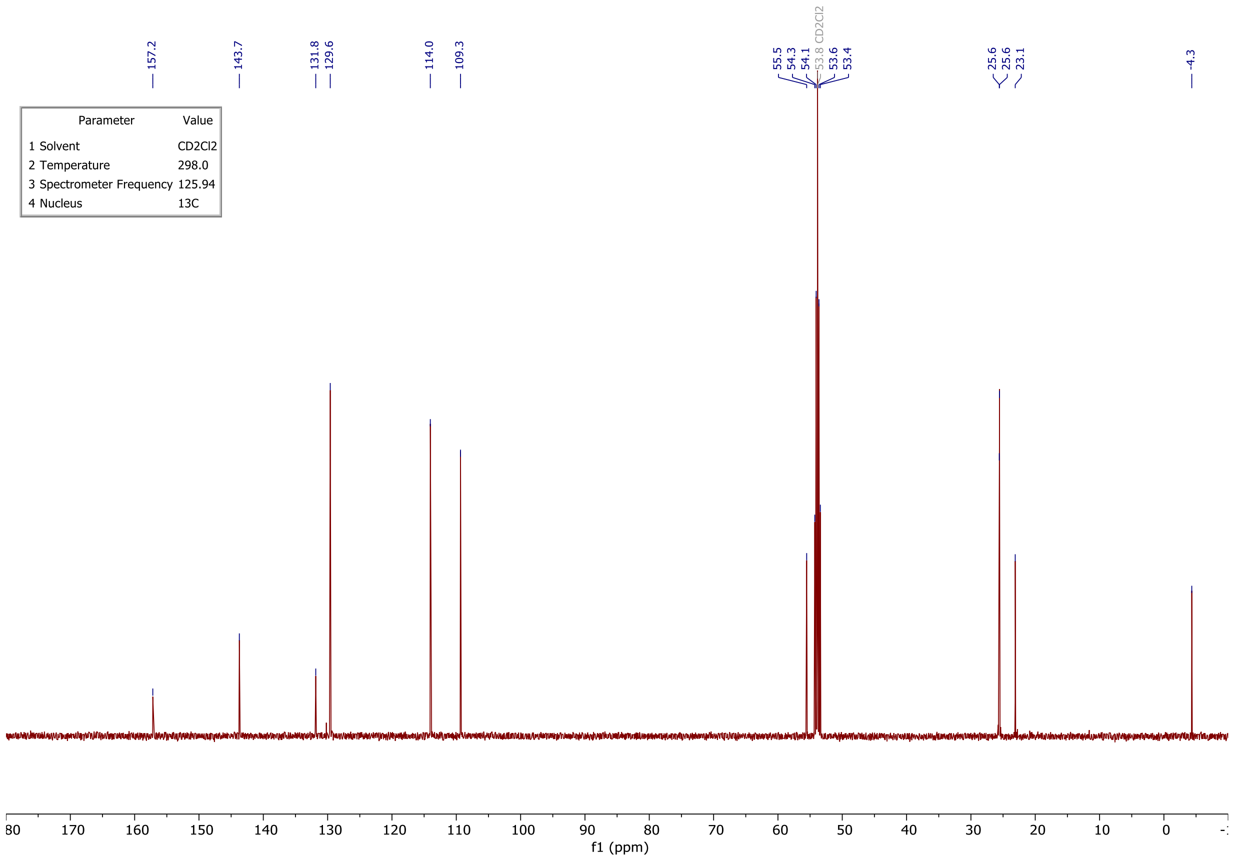

(4-fluorobenzyl)(methyl)bis(2-methylallyl)silane 1d

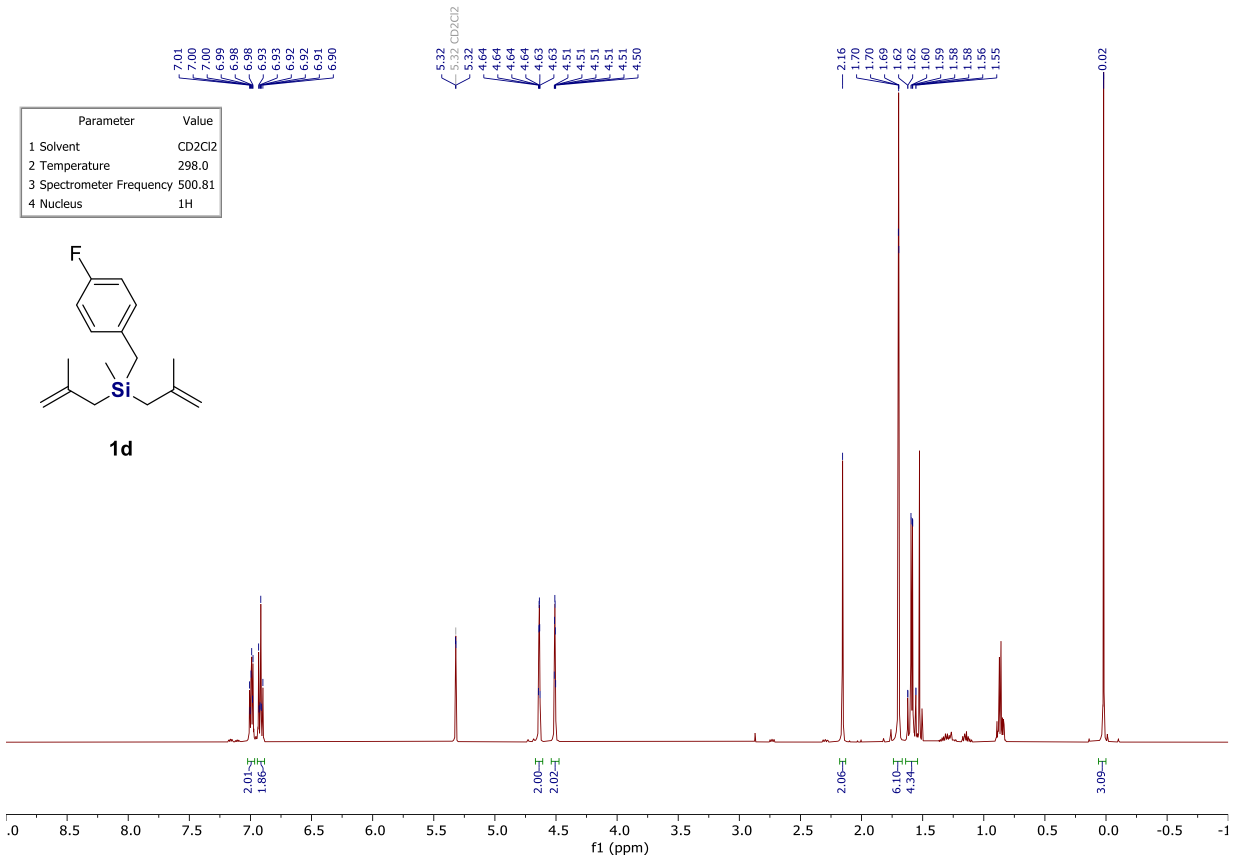

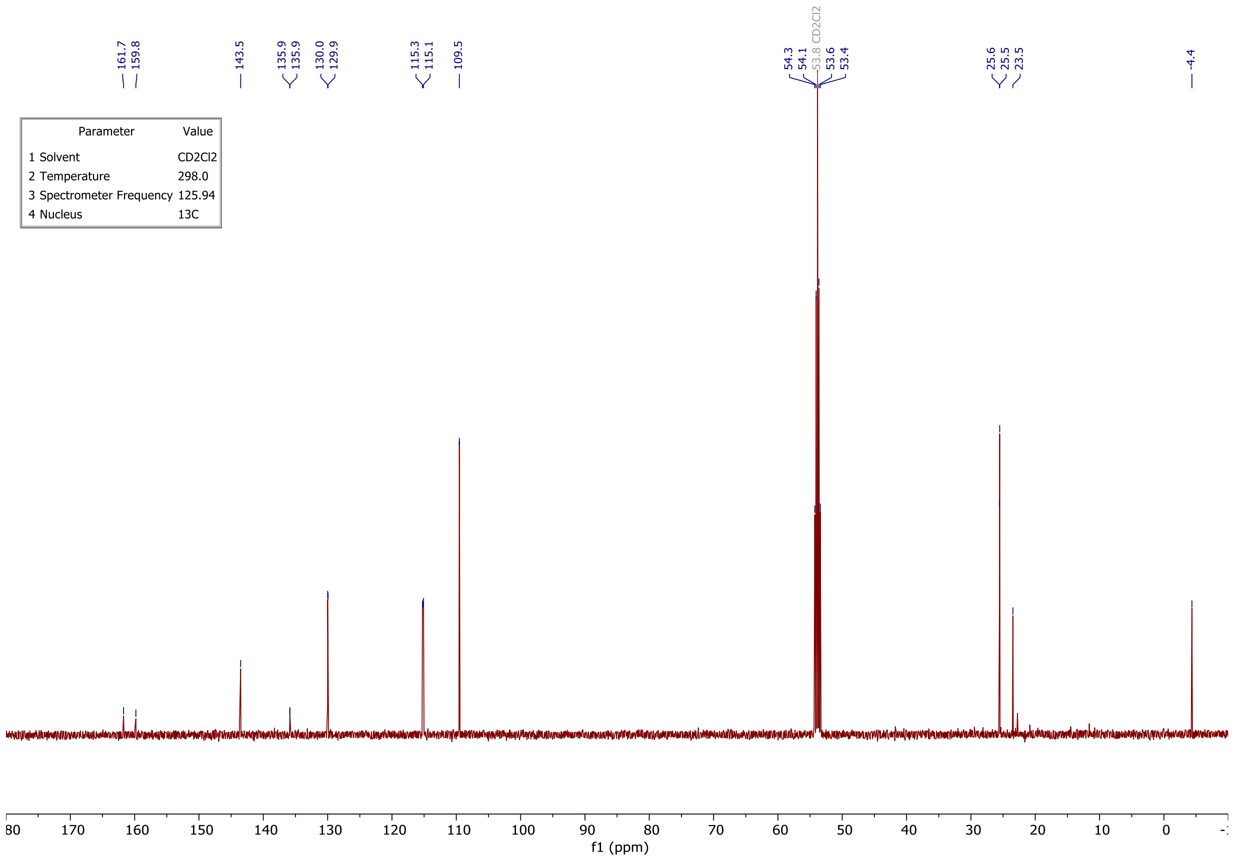

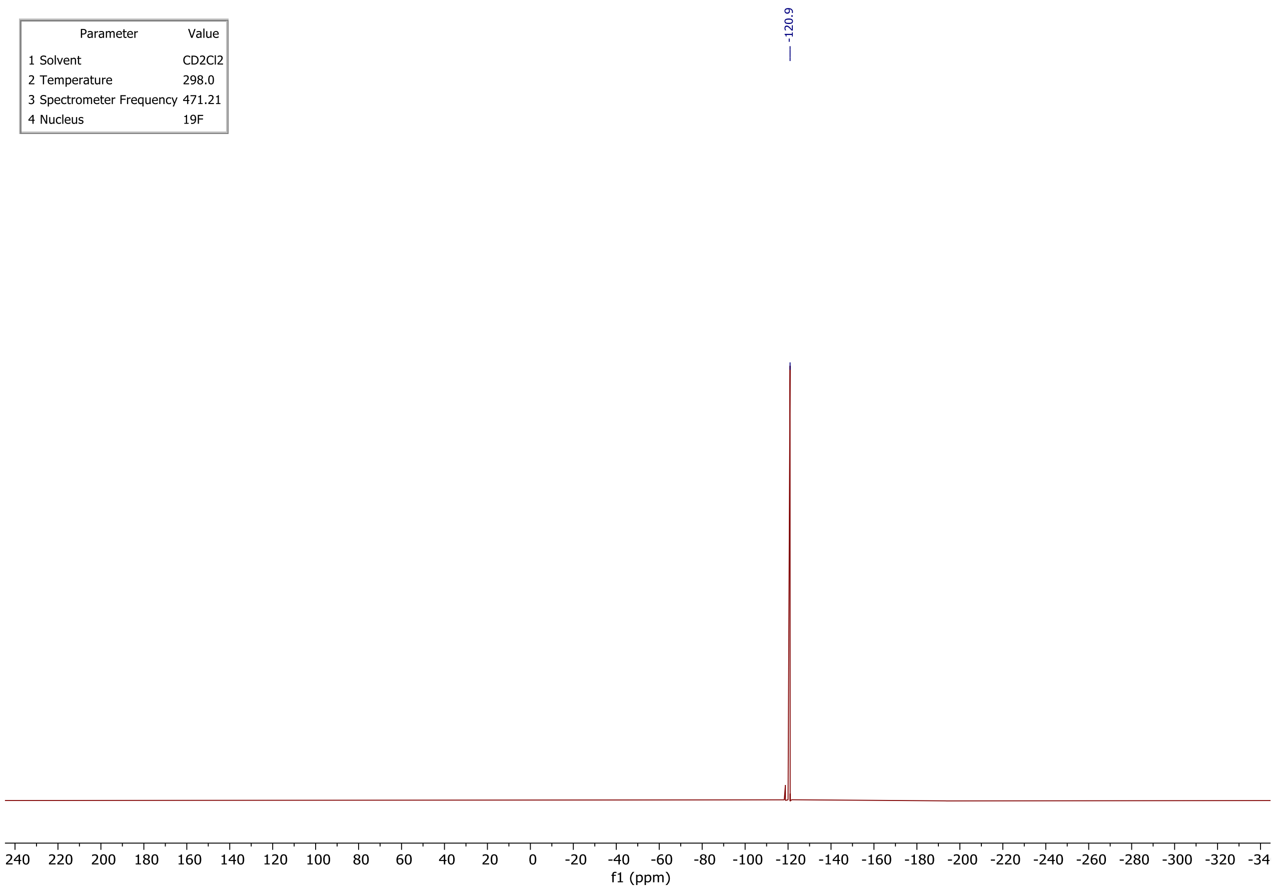

methylbis(2-methylallyl)(3-methylbenzyl)silane 1e

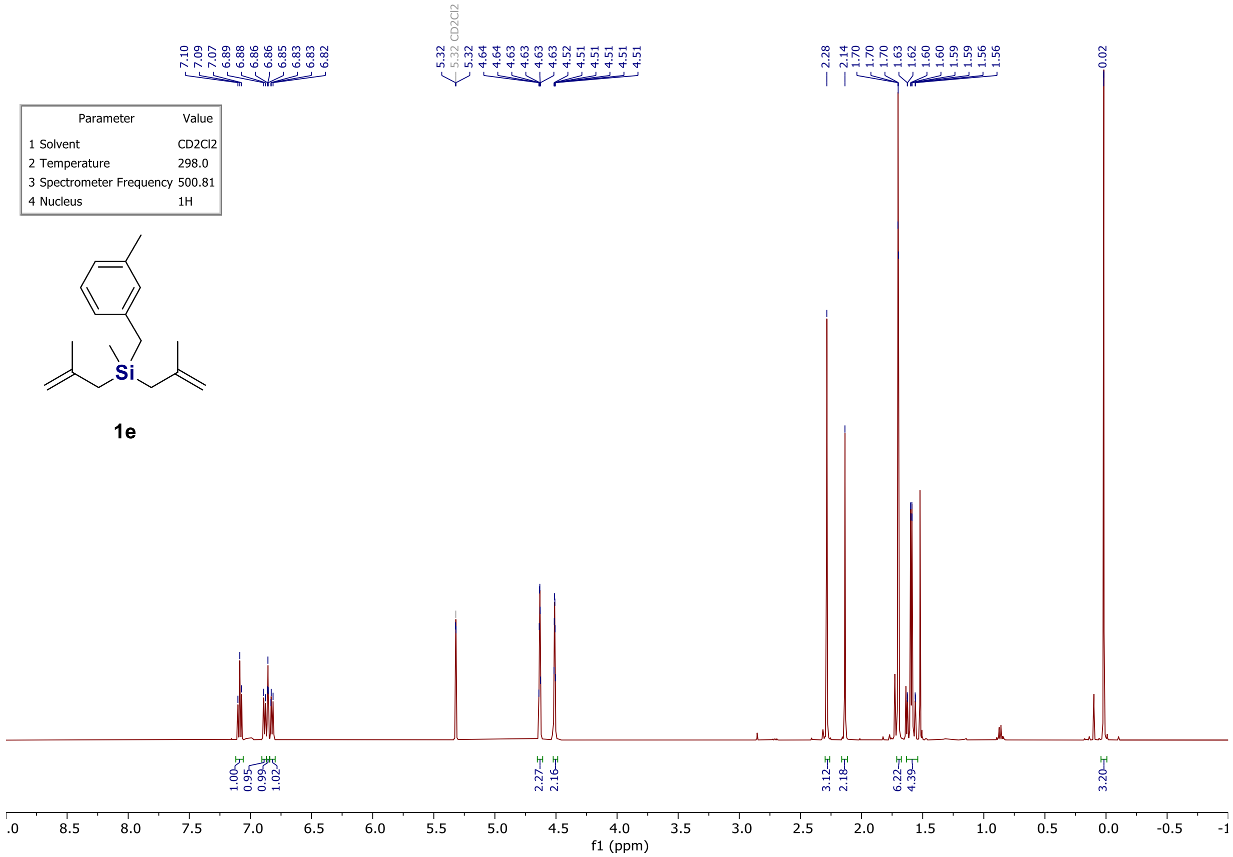

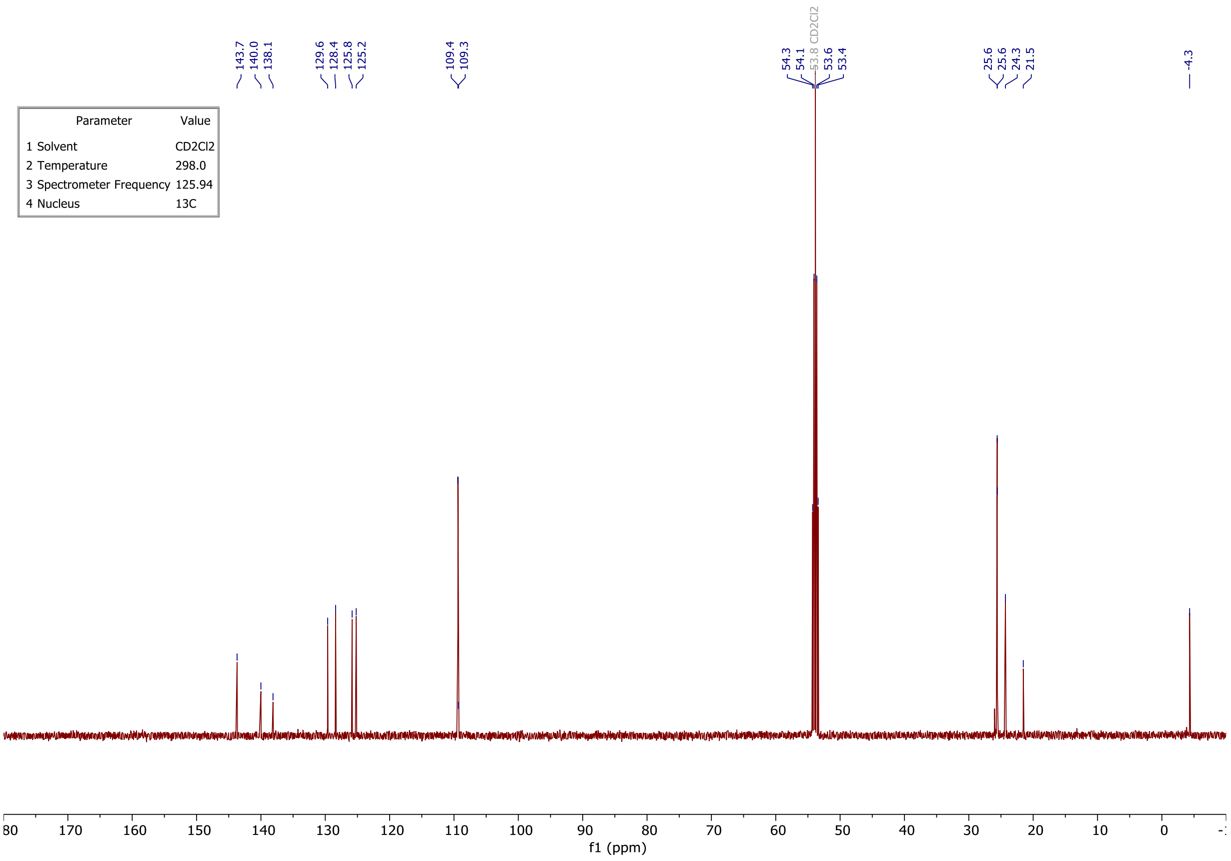

(3-methoxybenzyl)(methyl)bis(2-methylallyl)silane 1f

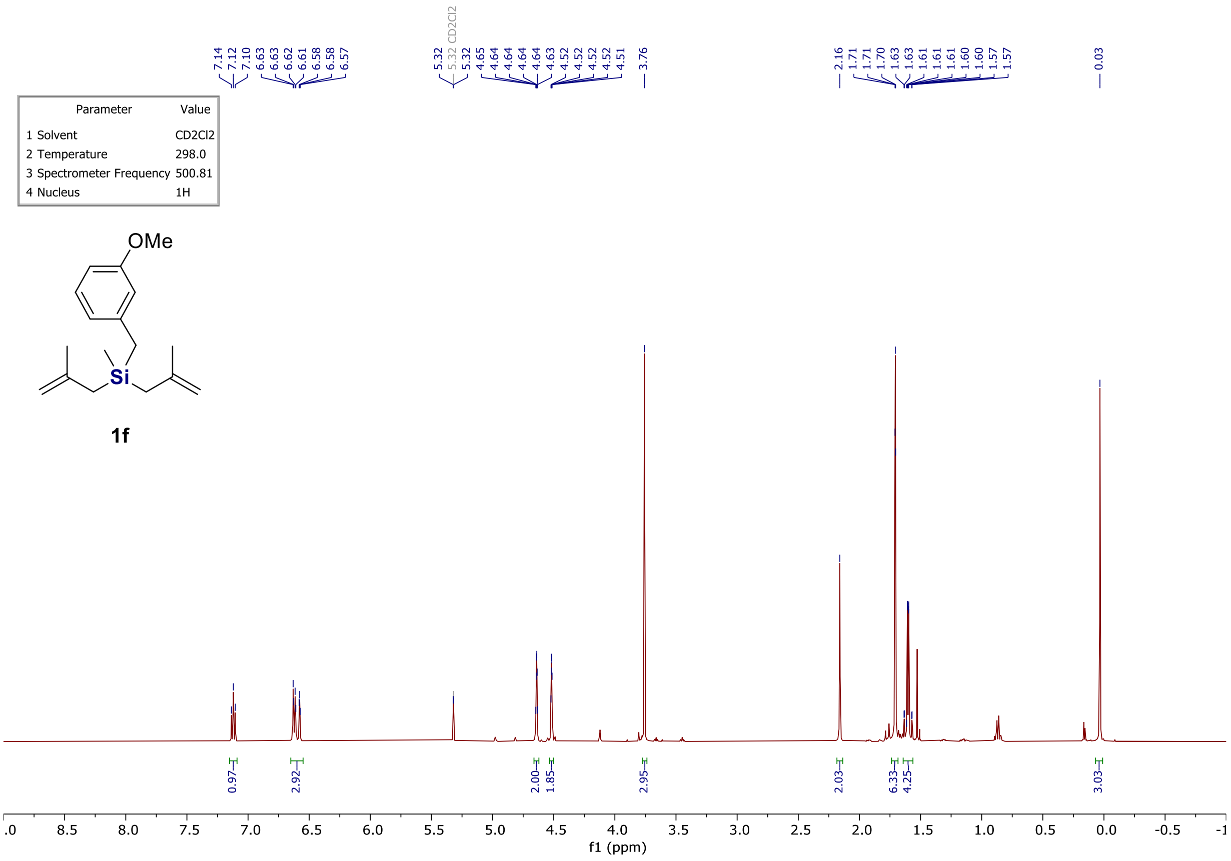

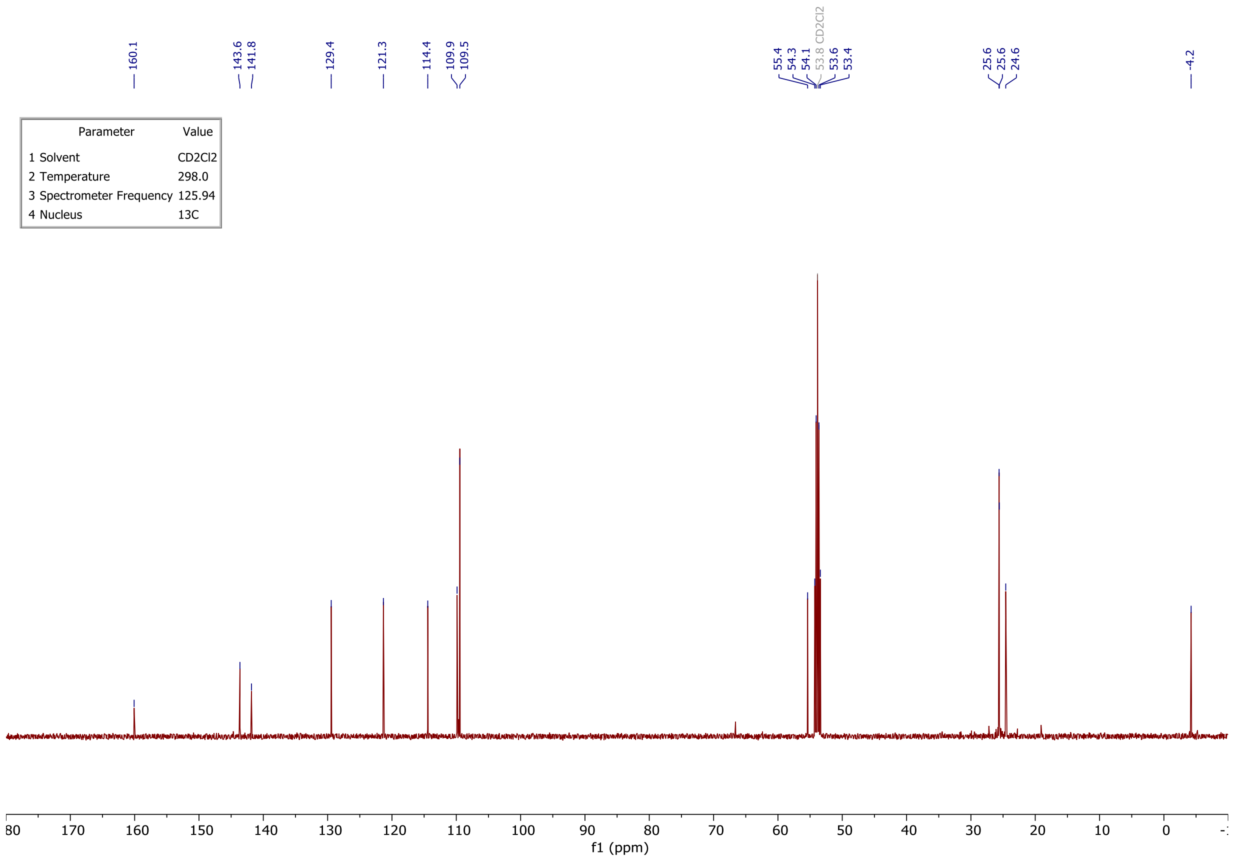

(3-fluorobenzyl)(methyl)bis(2-methylallyl)silane 1g

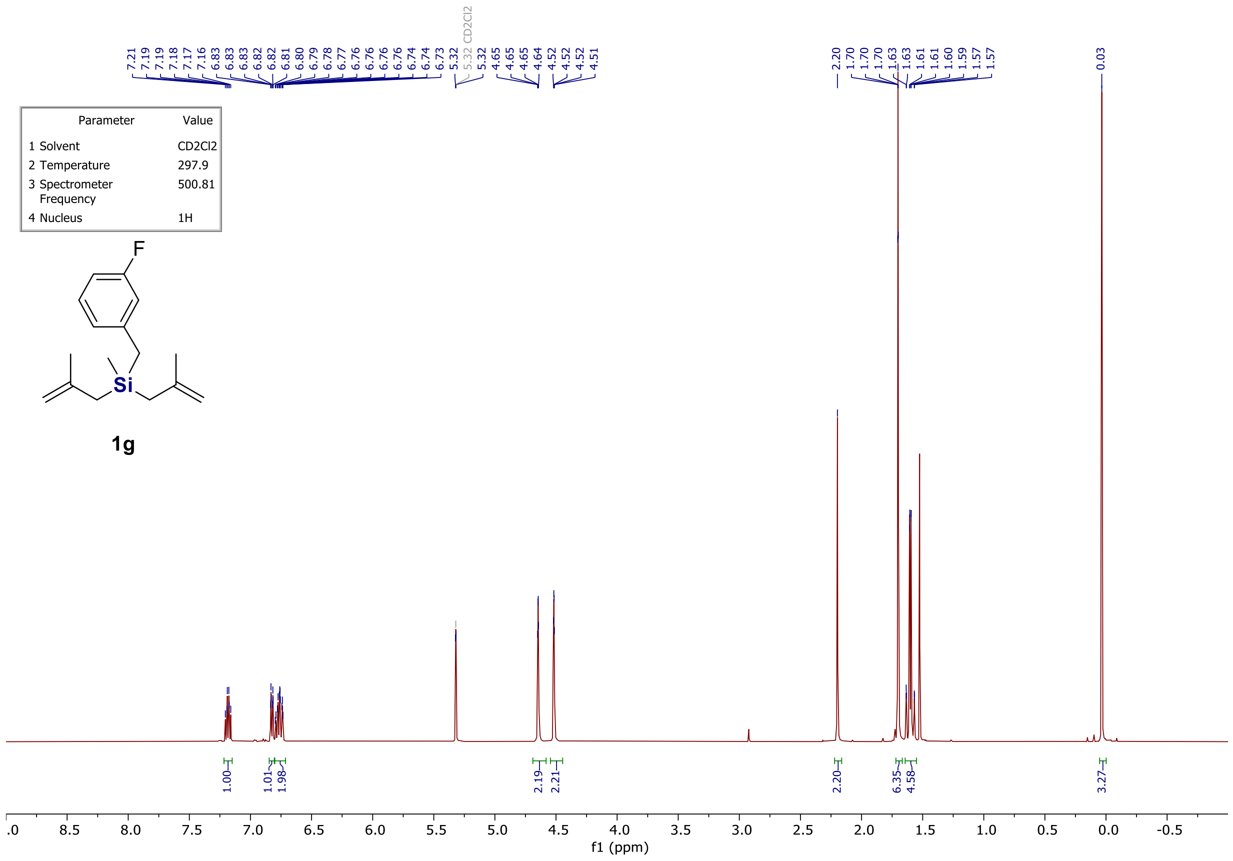

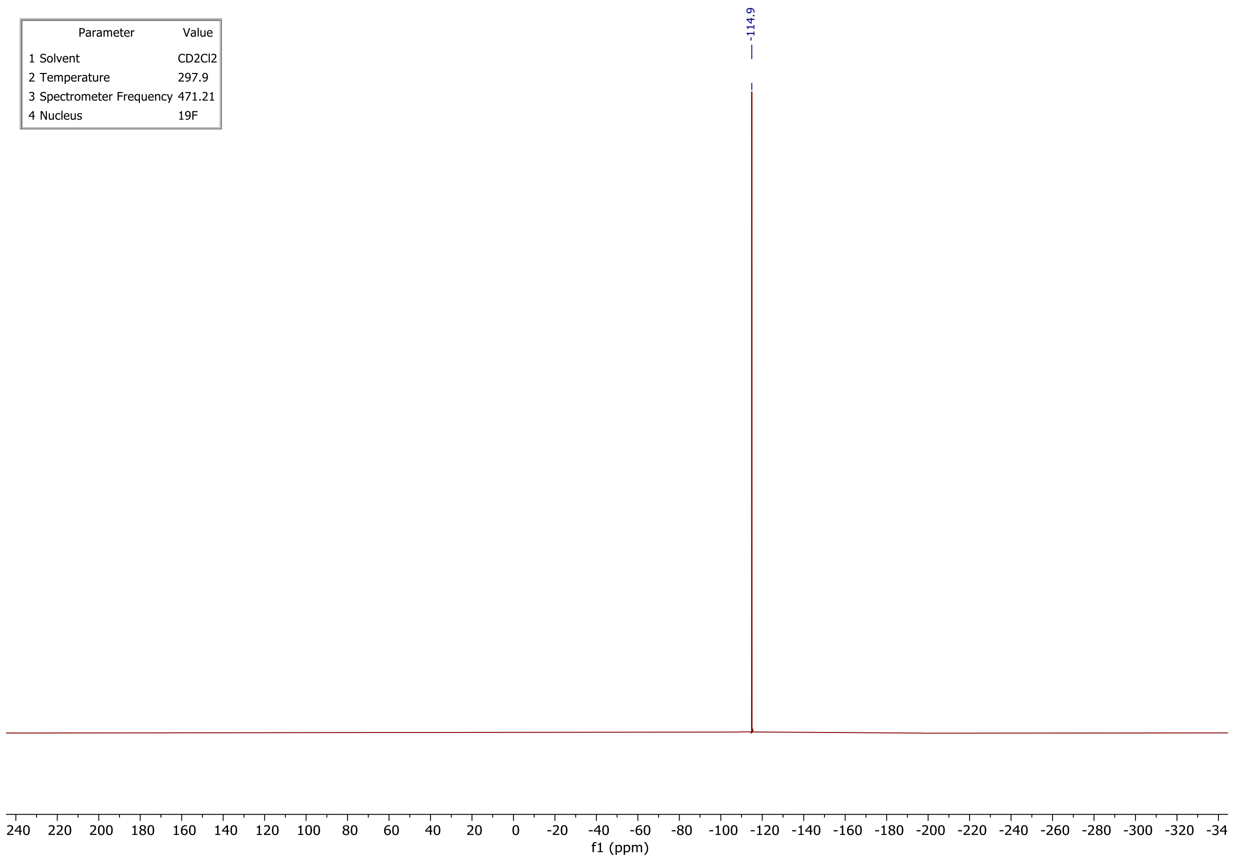
-114.9
Parameter Value
1 Solvent CD2Cl2
2 Temperature 297.9
3 Spectrometer Frequency 471.21
4 Nucleus 19F
240 220 200 180 160 140 120 100 80 60 40 20 0 -20 -40 -60 -80 -100 -120 -140 -160 -180 -200 -220 -240 -260 -280 -300 -320
f1 (ppm)

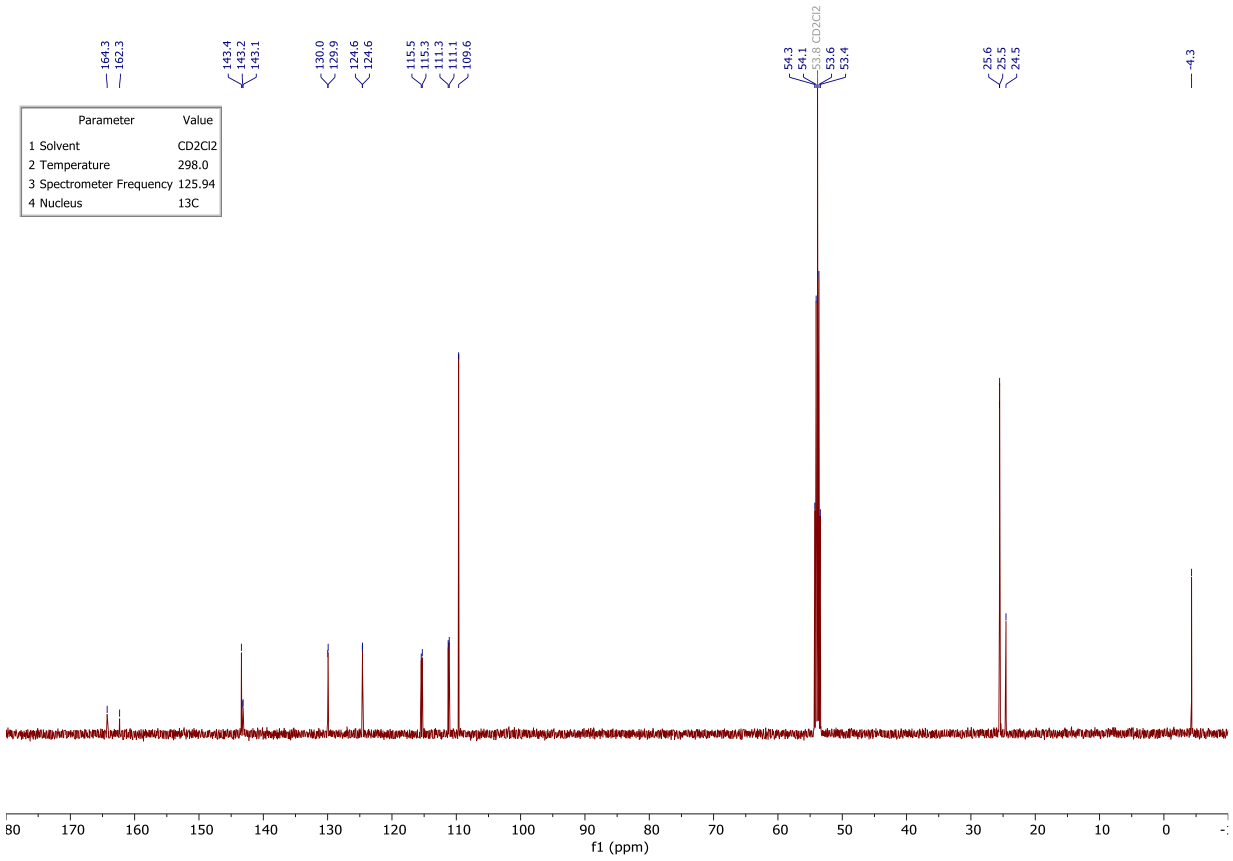
164.3
162.3
143.4
143.2
143.1
130.0
129.9
124.6
124.6
115.5
115.3
111.3
111.1
109.6
54.3
54.1
53.8 CD2Cl2
53.6
53.4
25.6
25.5
24.5
-4.3
Parameter Value
1 Solvent CD2Cl2
2 Temperature 298.0
3 Spectrometer Frequency 125.94
4 Nucleus 13C
170 160 150 140 130 120 110 100 90 80 70 60 50 40 30 20 10 0
f1 (ppm)

methylbis(2-methylallyl)(2-methylbenzyl)silane 1h

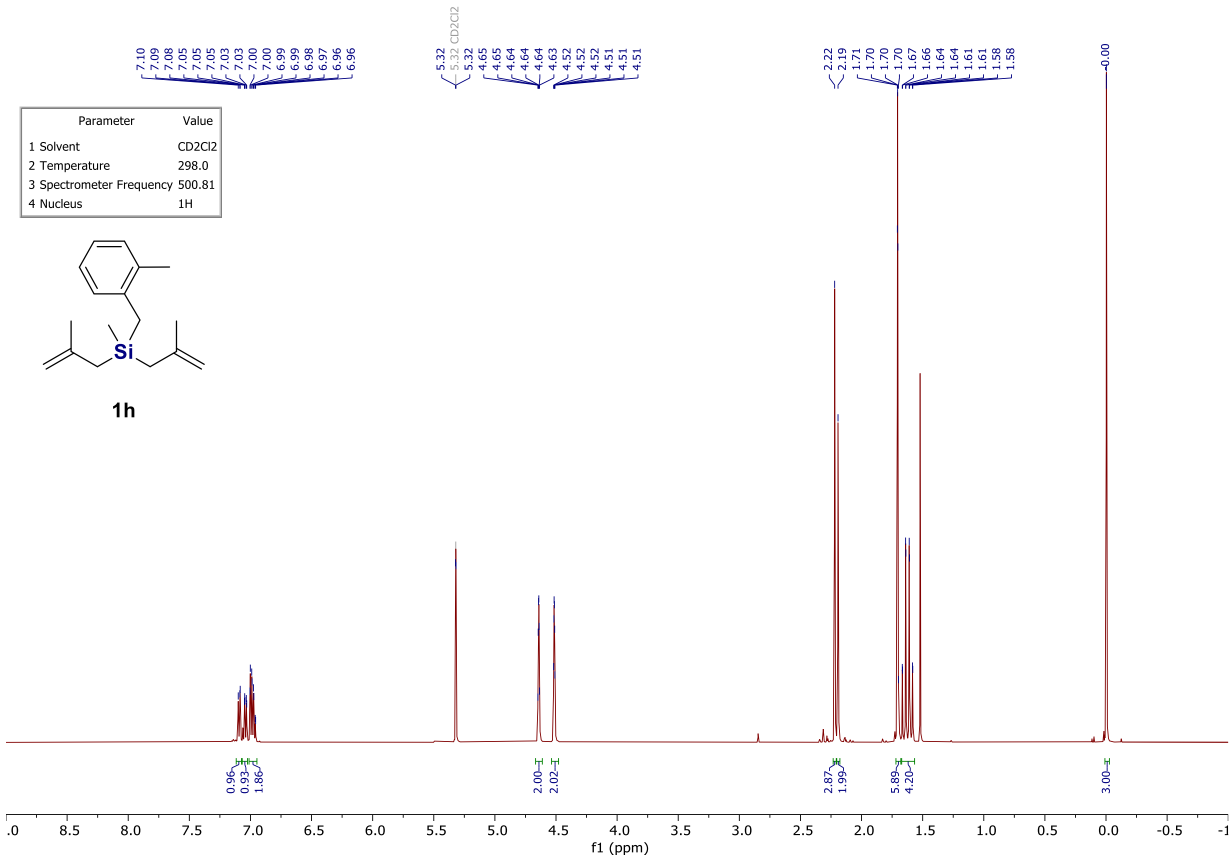

Parameter	Value
1 Solvent	CD2Cl2
2 Temperature	298.0
3 Spectrometer Frequency	500.81
4 Nucleus	1H

1h

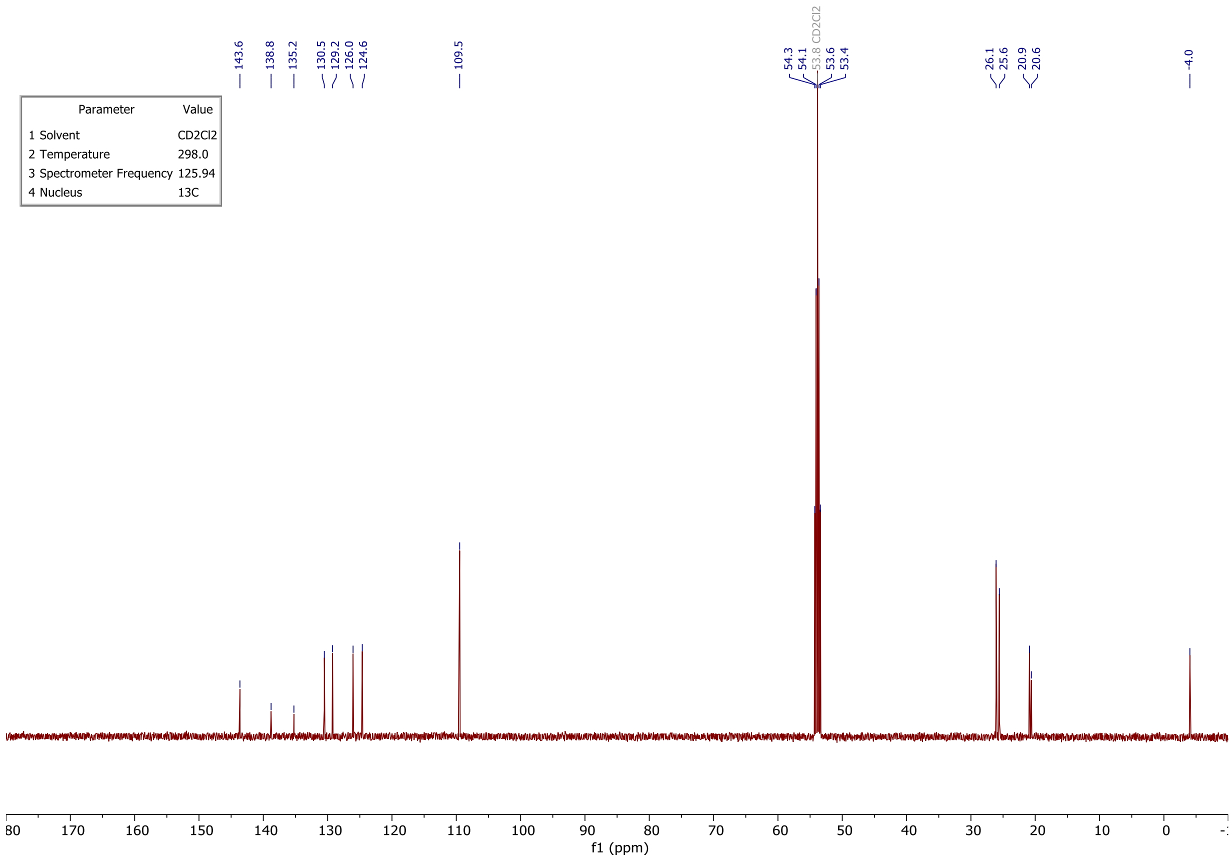

Parameter	Value
1 Solvent	CD2Cl2
2 Temperature	298.0
3 Spectrometer Frequency	125.94
4 Nucleus	13C

(2-fluorobenzyl)(methyl)bis(2-methylallyl)silane 1i

Parameter	Value
1 Solvent	CD2Cl2
2 Temperature	297.9
3 Spectrometer Frequency	500.81
4 Nucleus	1H

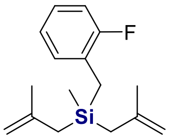

1i

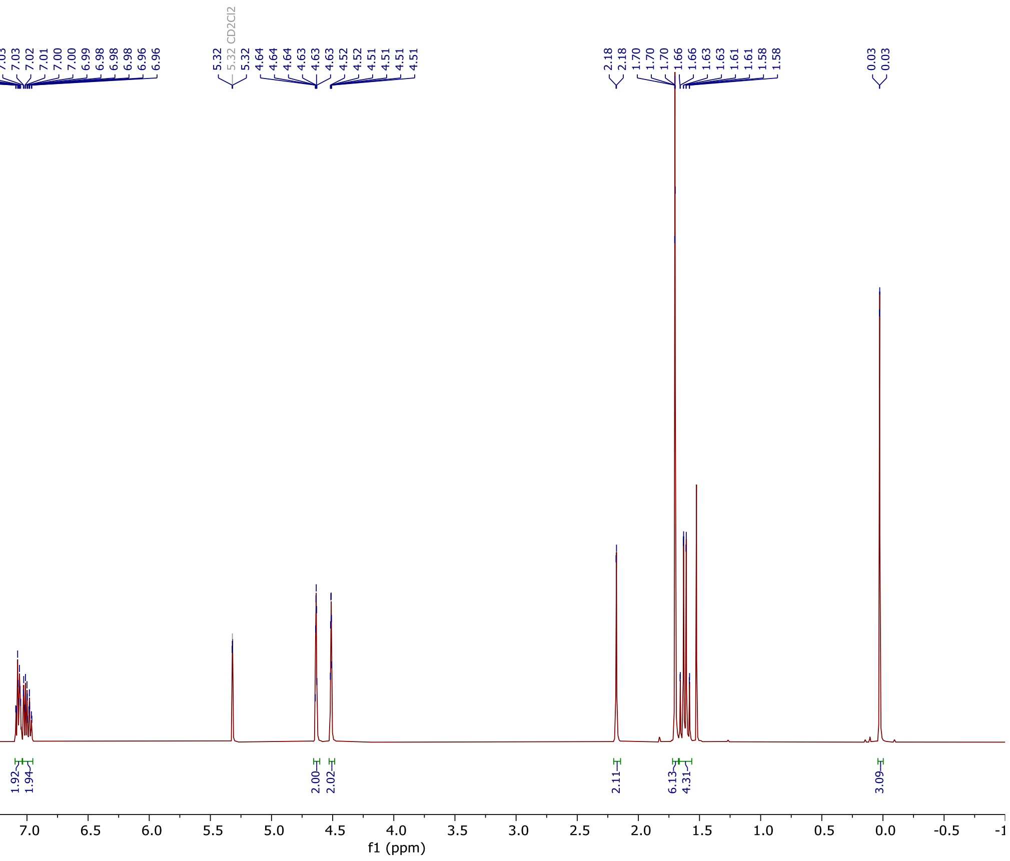

Parameter	Value
1 Solvent	CD2Cl2
2 Temperature	298.0
3 Spectrometer Frequency	471.21
4 Nucleus	19F

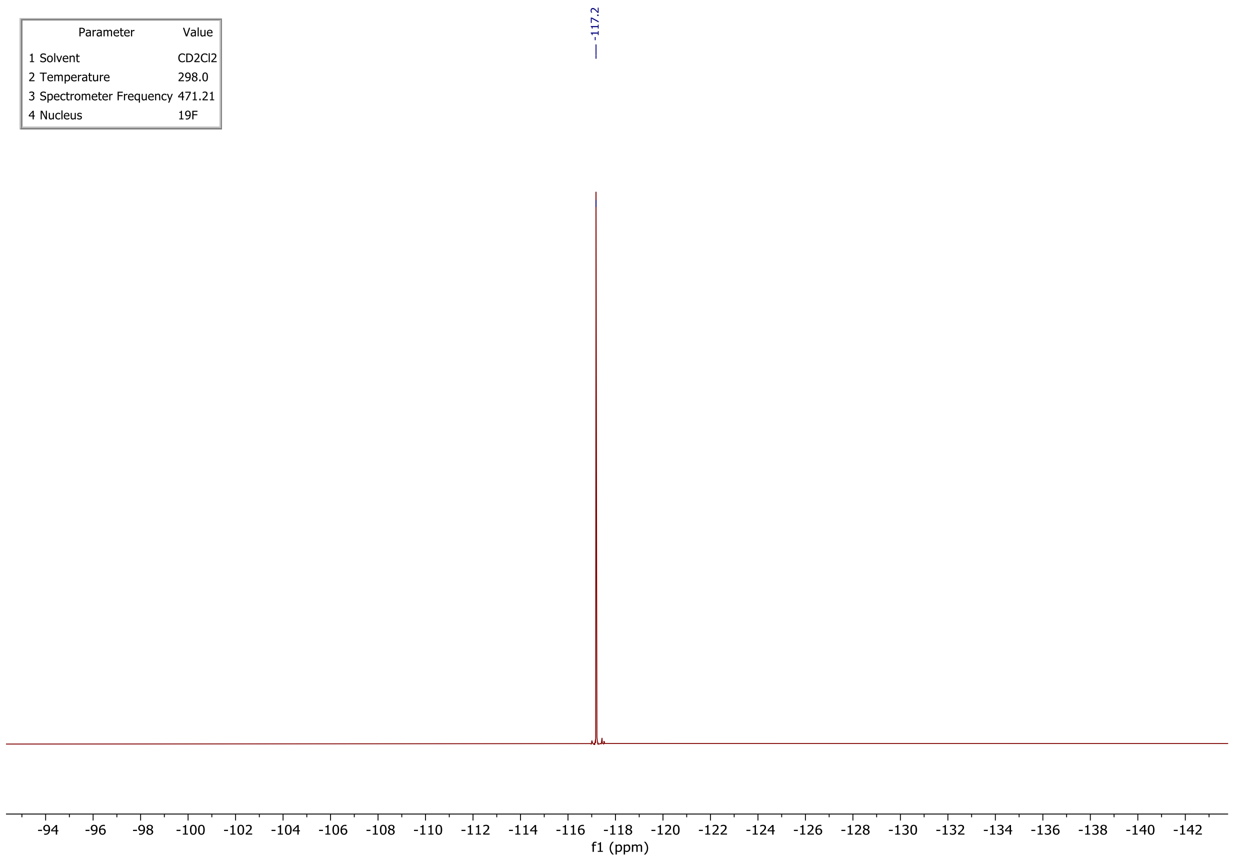

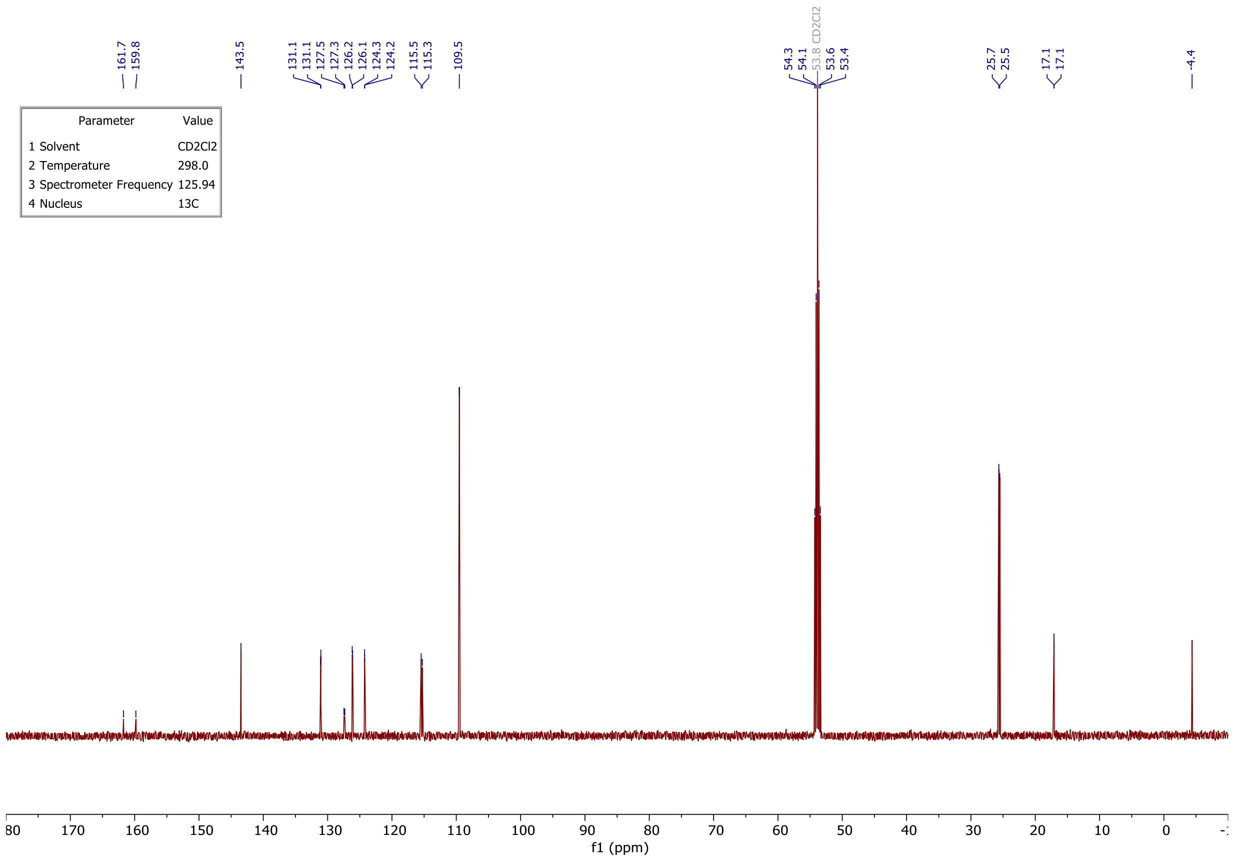

(3,4-dimethylbenzyl)(methyl)bis(2-methylallyl)silane 1j

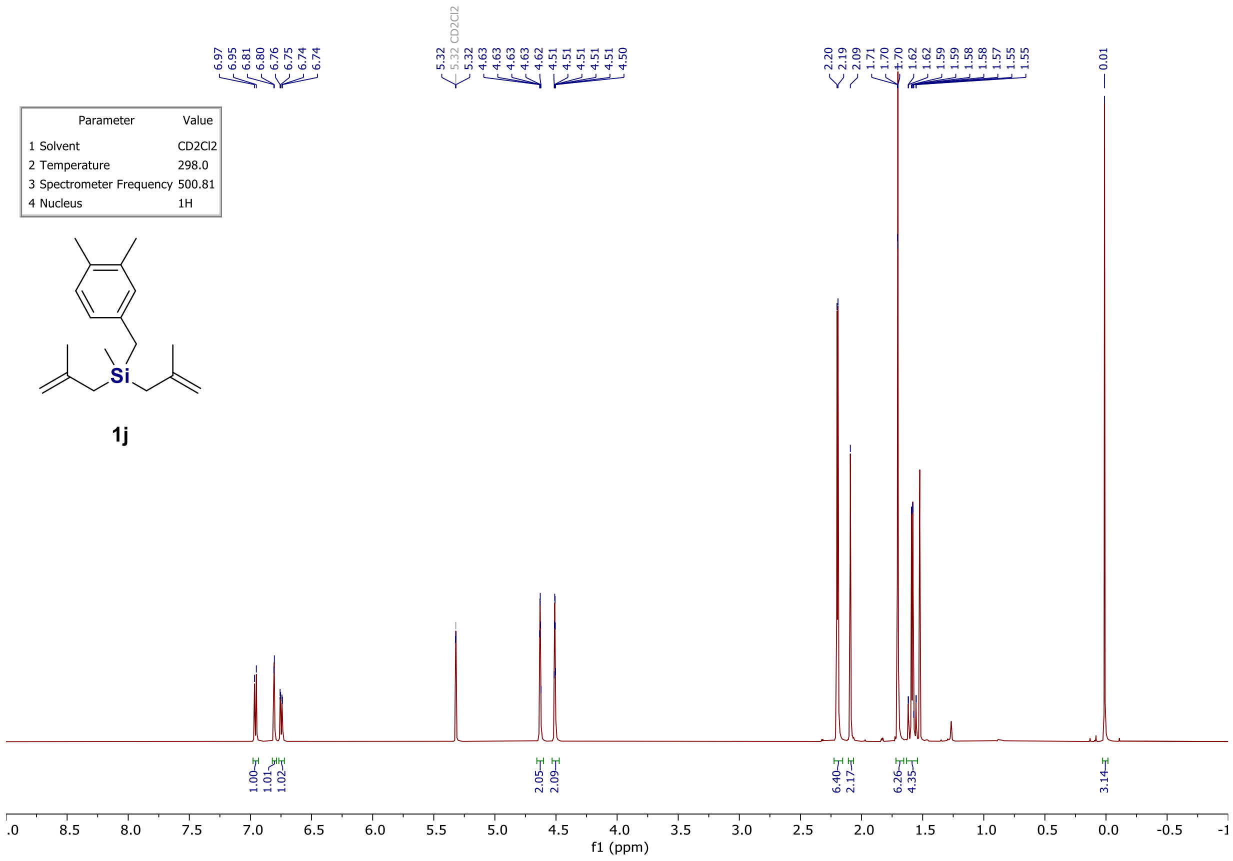

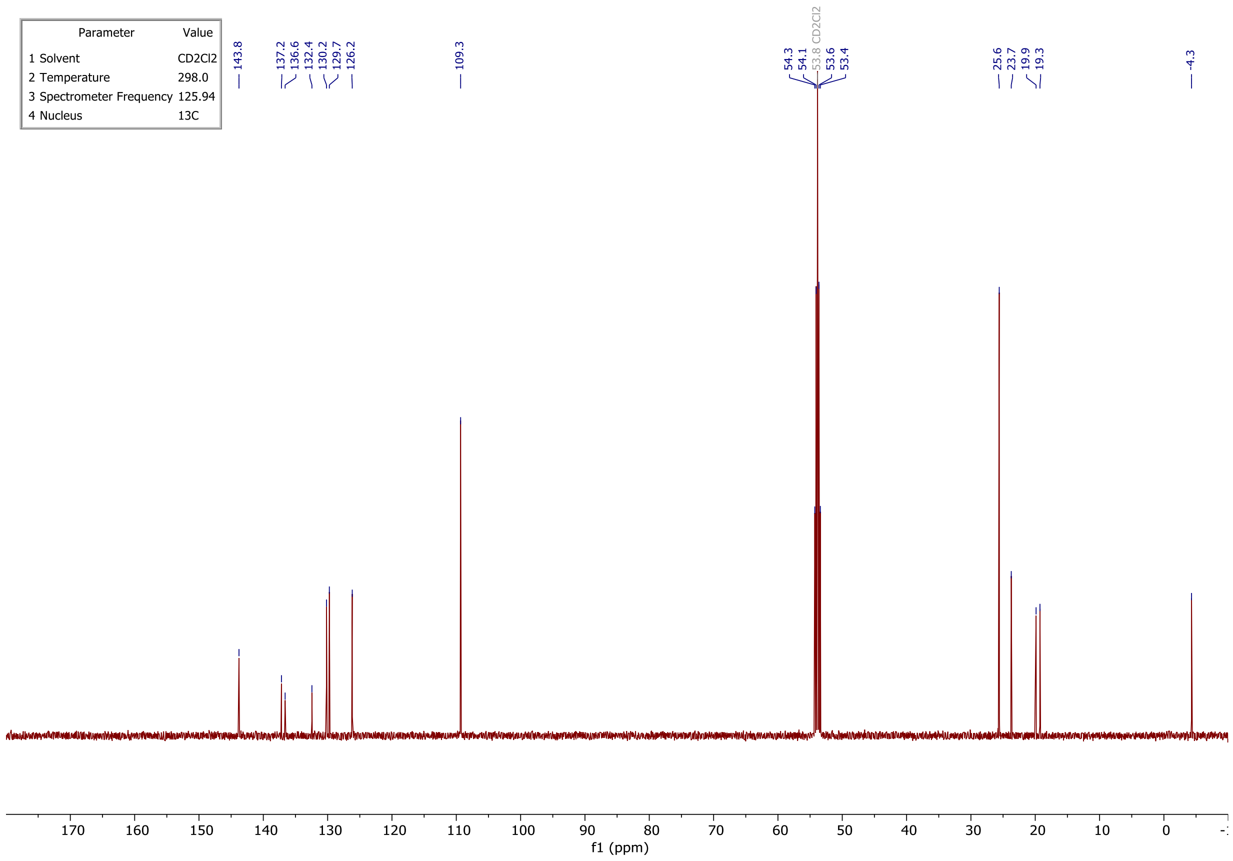

methylbis(2-methylallyl)(naphthalen-2-ylmethyl)silane 1k

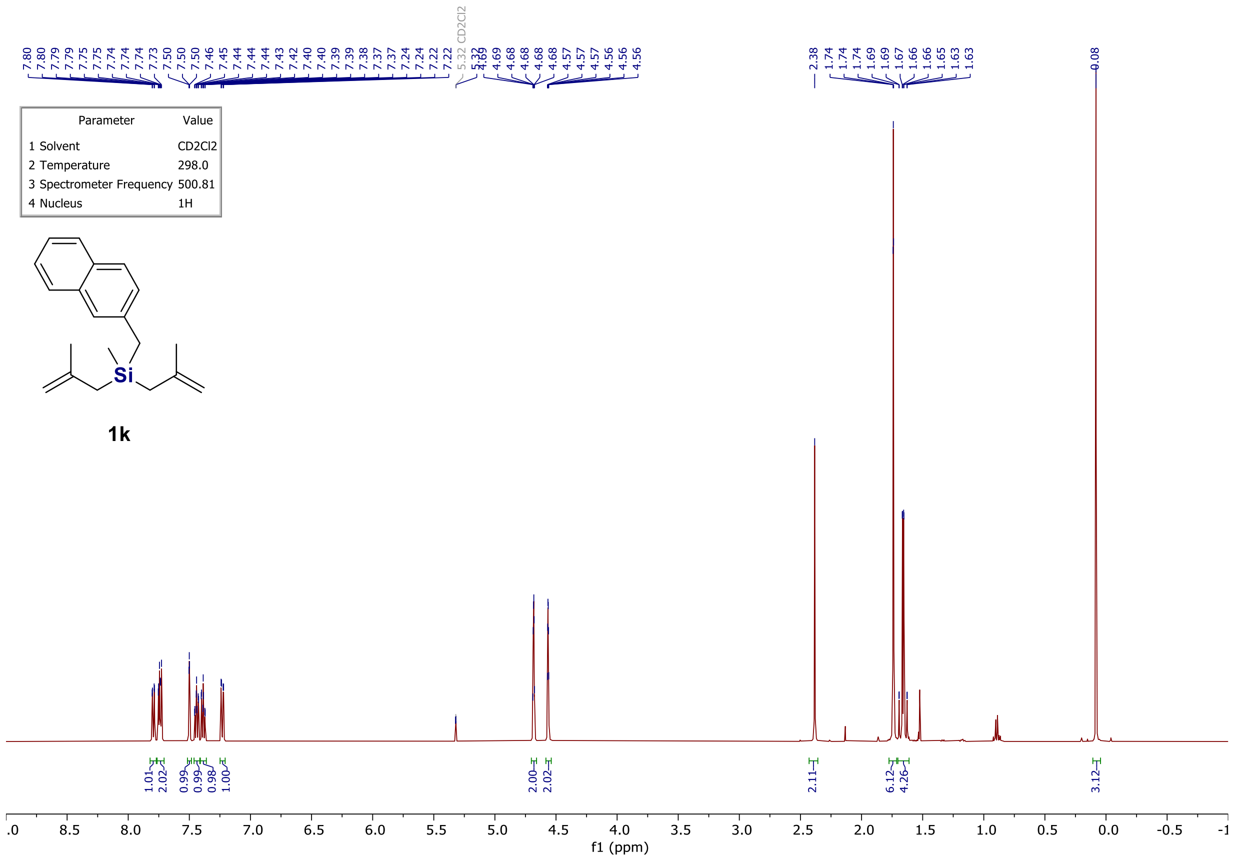

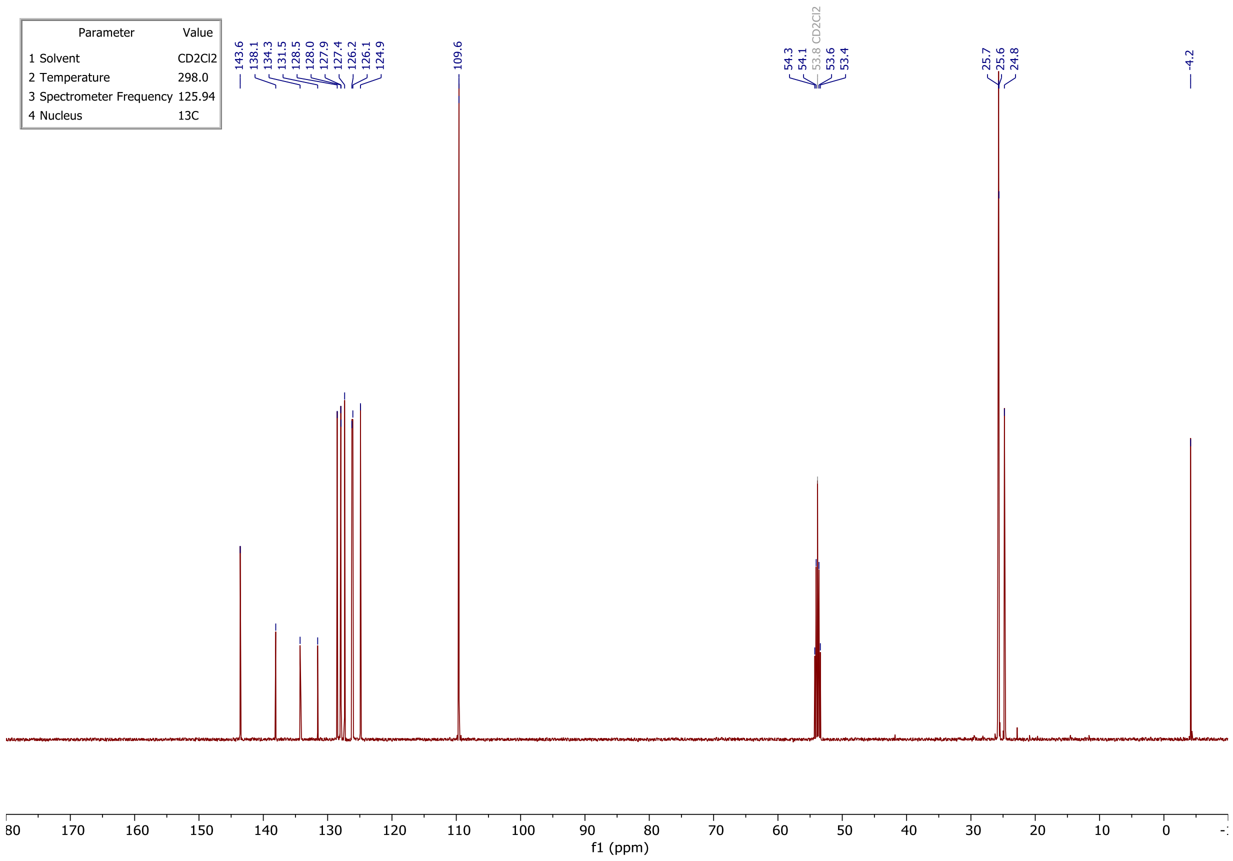

methylbis(2-methylallyl)(phenyl)silane 1l

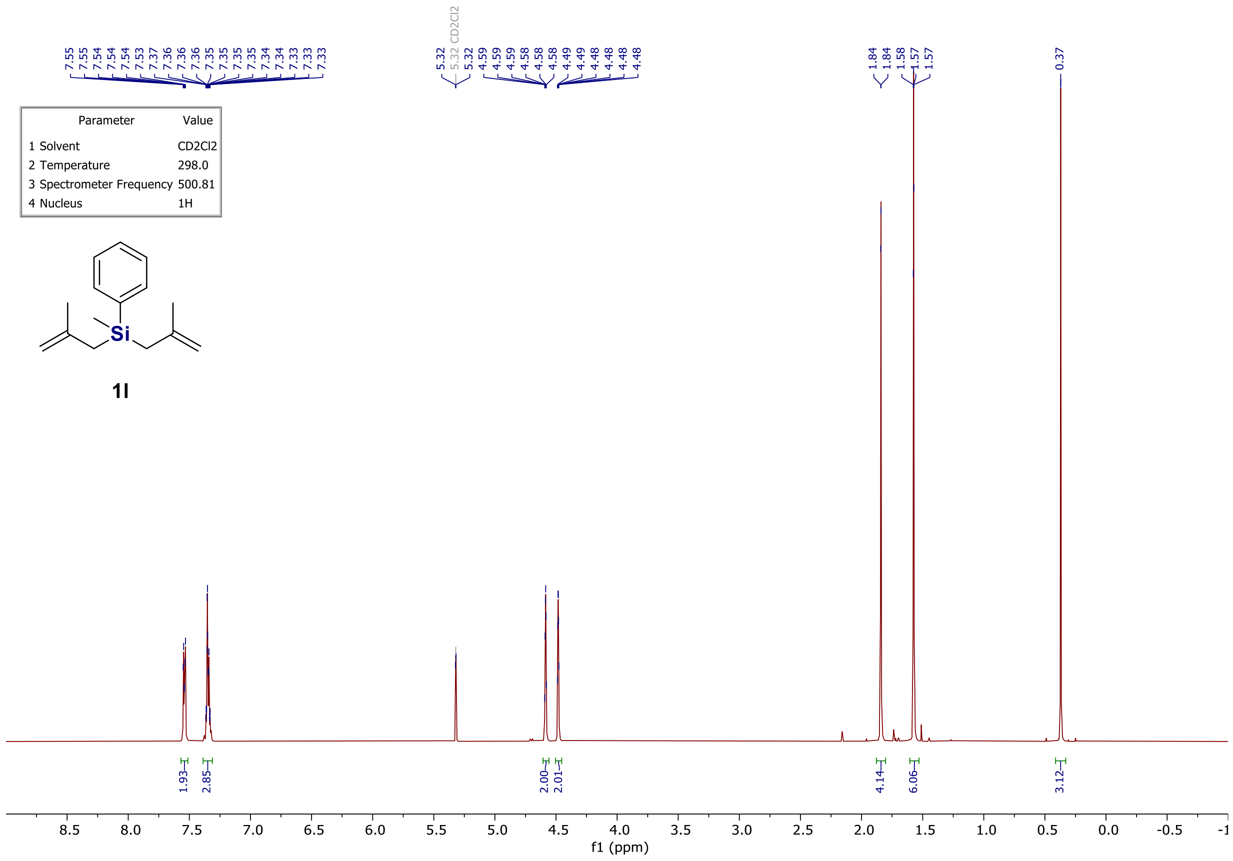

1l

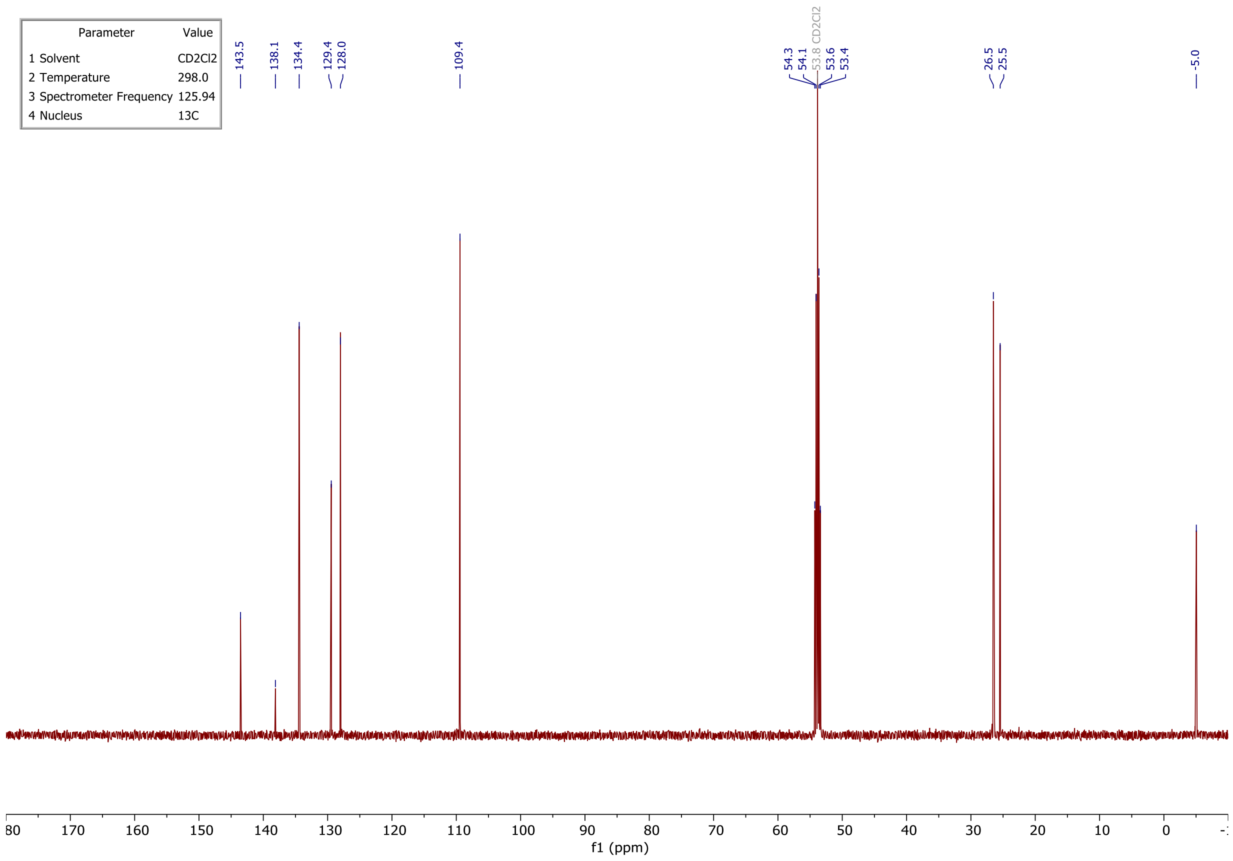

methylbis(2-methylallyl)(p-tolyl)silane 1m

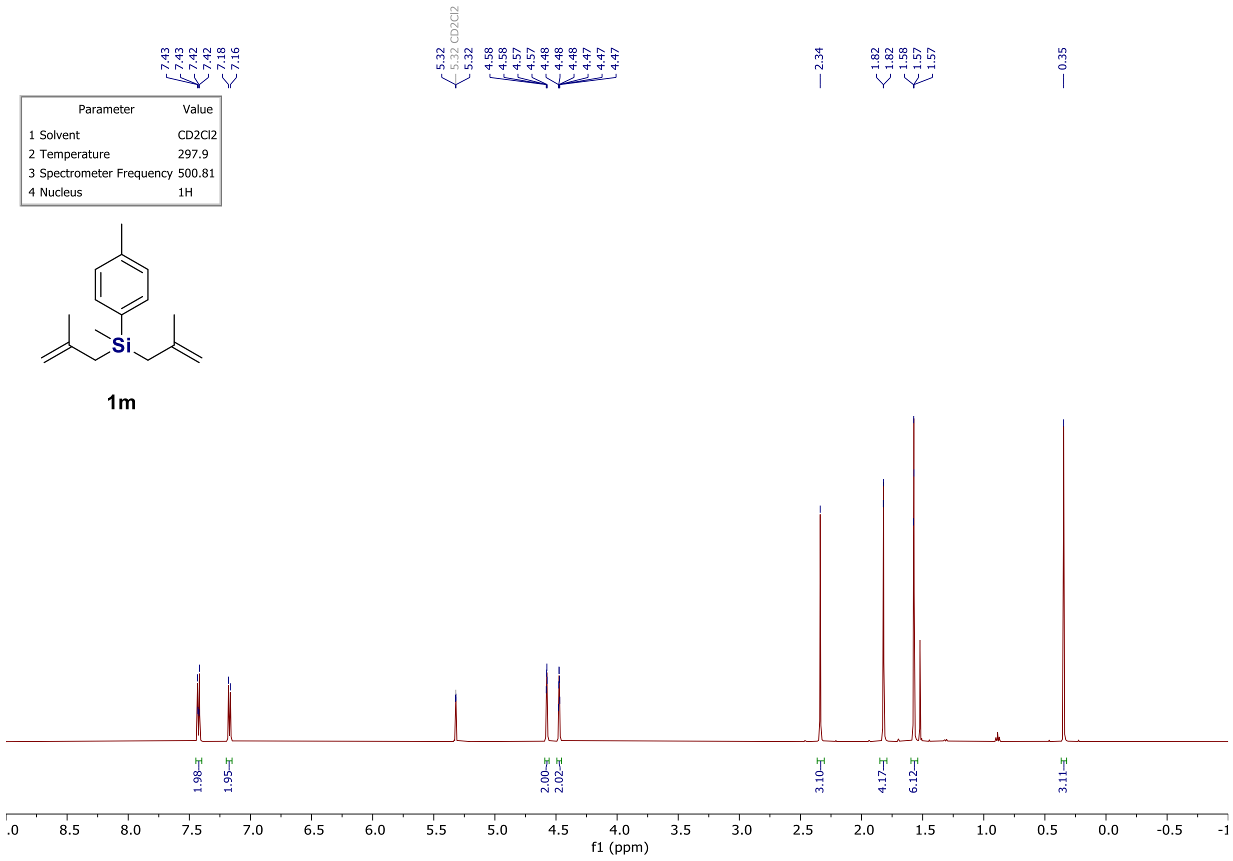

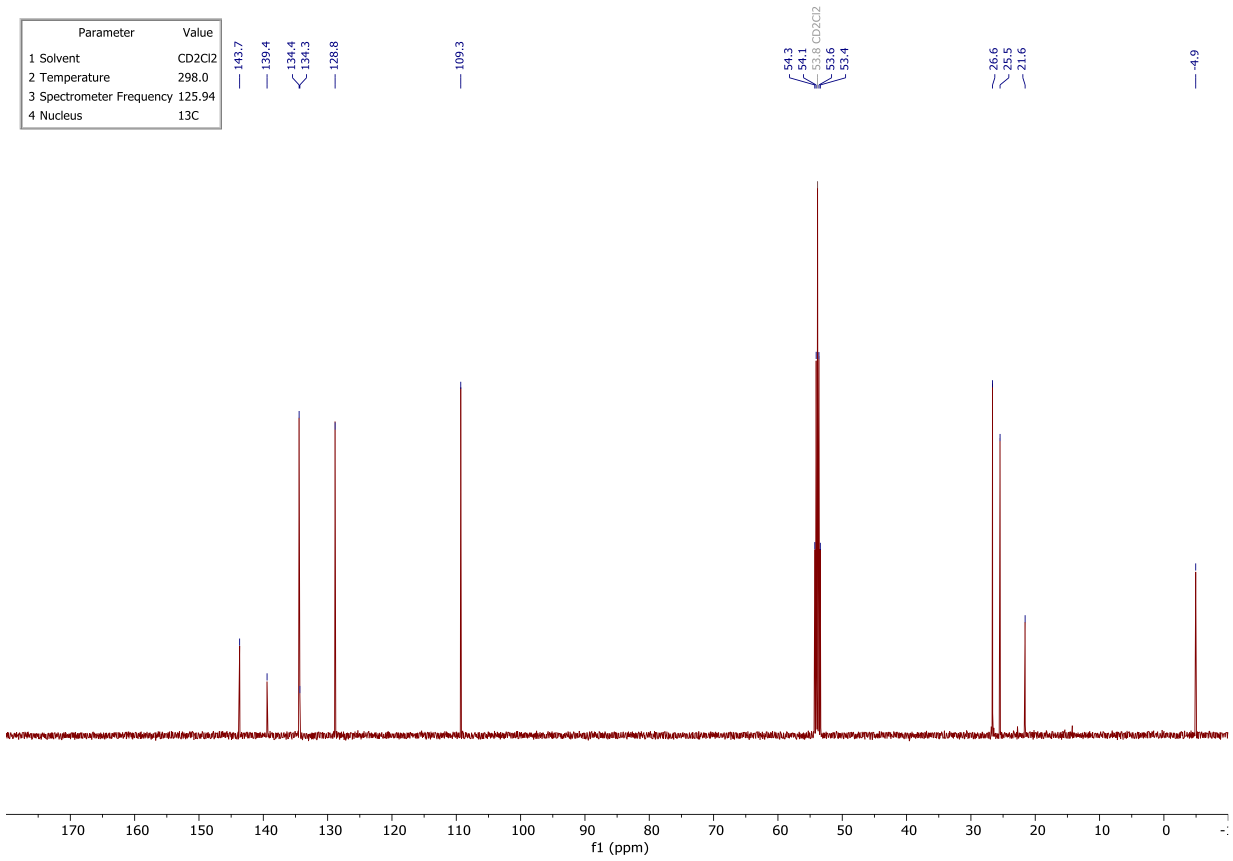

(4-fluorophenyl)(methyl)bis(2-methylallyl)silane 1n

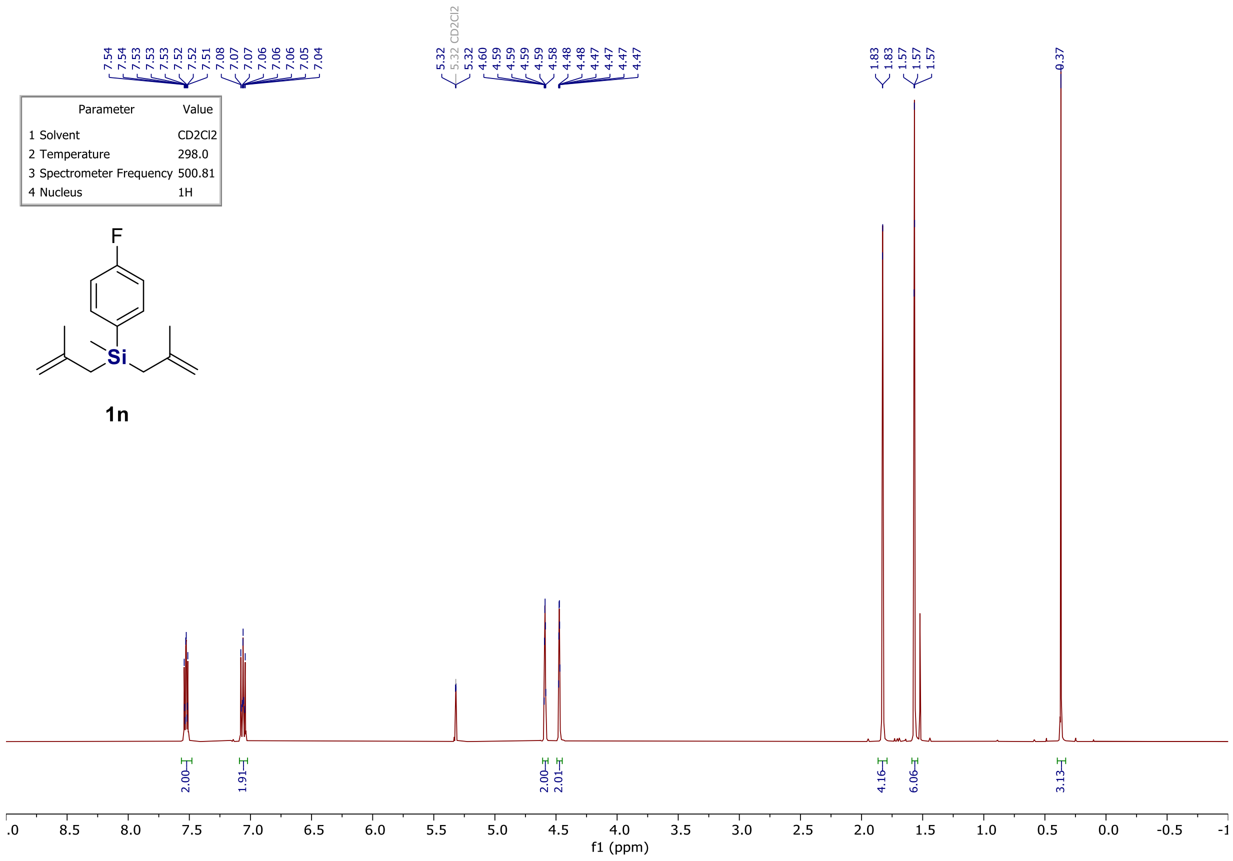

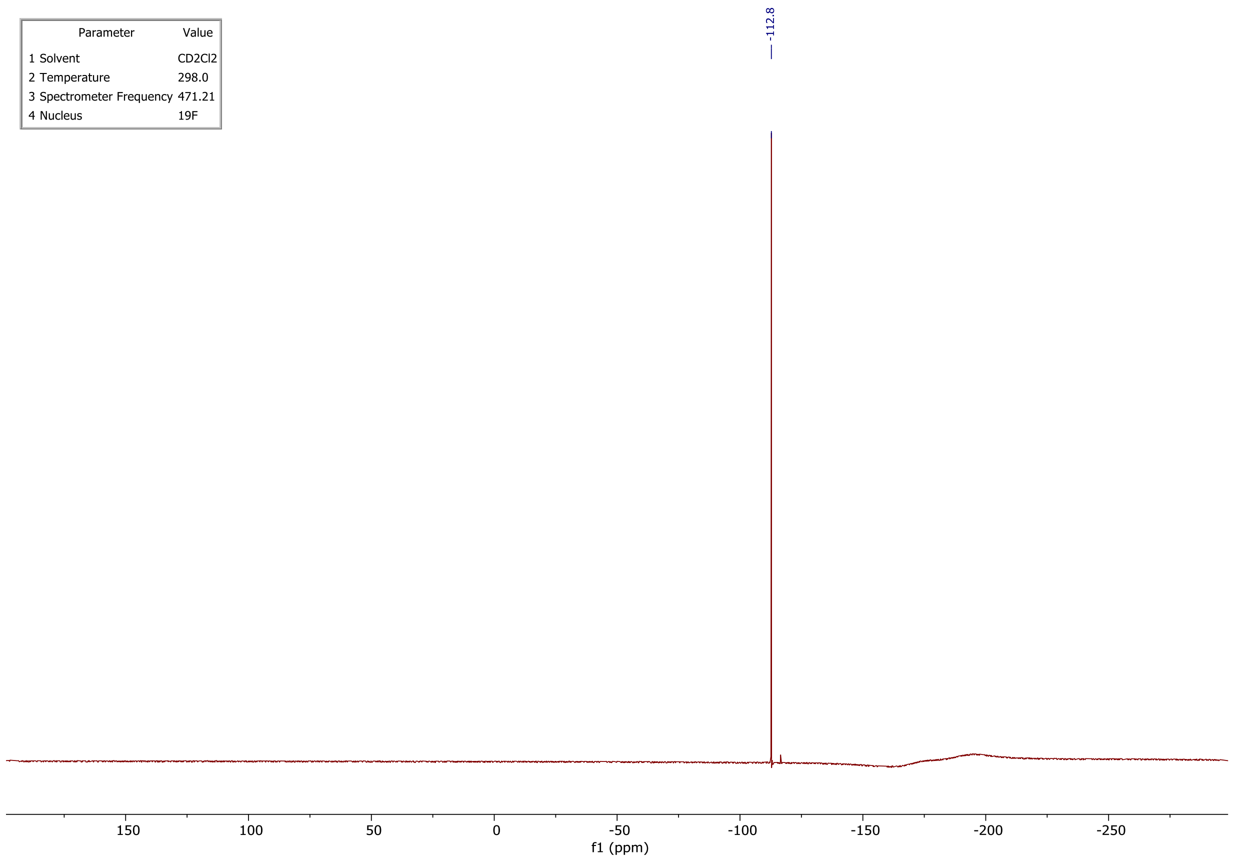
Parameter Value
1 Solvent CD2Cl2
2 Temperature 298.0
3 Spectrometer Frequency 471.21
4 Nucleus 19F
-112.8
150 100 50 0 -50 -100 -150 -200 -250
f1 (ppm)

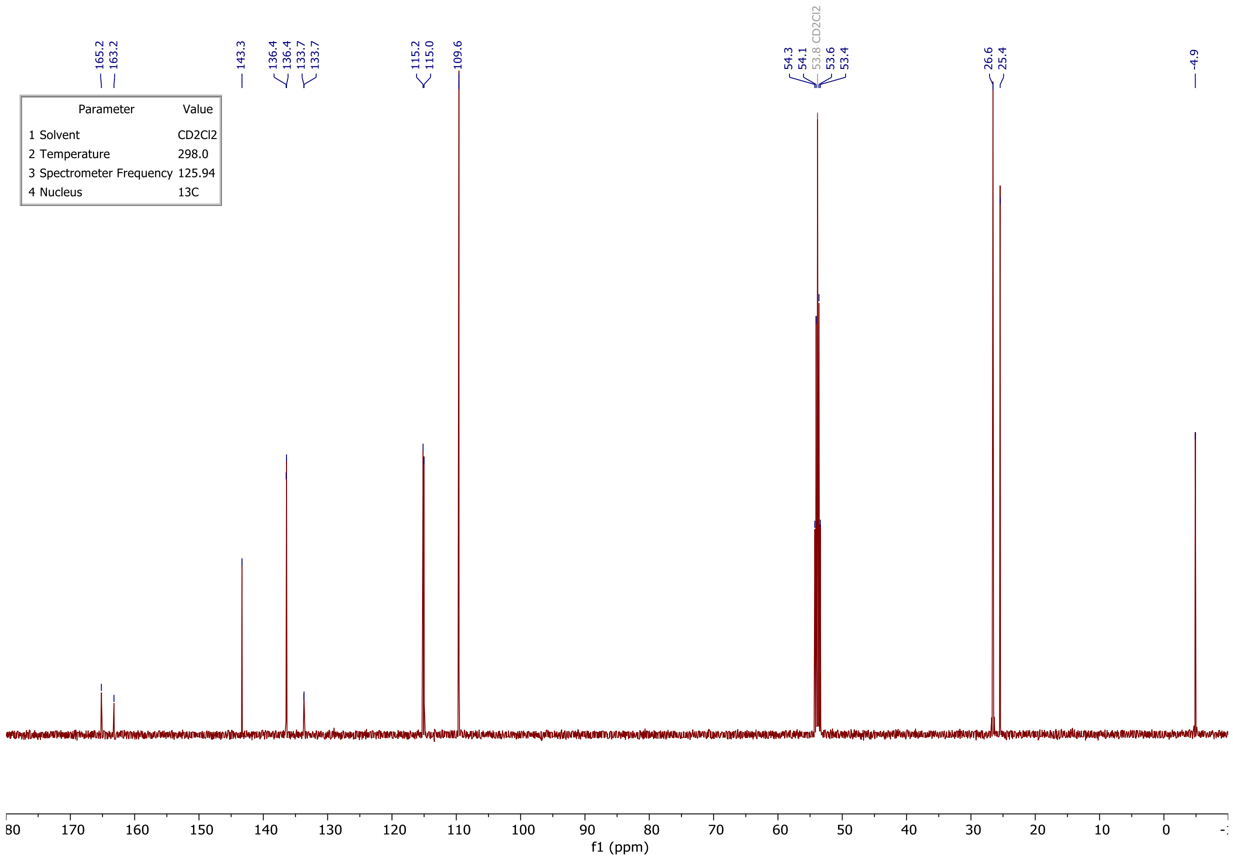
165.2
163.2
143.3
136.4
136.4
133.7
133.7
115.2
115.0
109.6
54.3
54.1
53.8 CD2Cl2
53.6
53.4
26.6
25.4
-4.9
Parameter Value
1 Solvent CD2Cl2
2 Temperature 298.0
3 Spectrometer Frequency 125.94
4 Nucleus 13C
80 170 160 150 140 130 120 110 100 90 80 70 60 50 40 30 20 10 0
f1 (ppm)

methylbis(2-methylallyl)(thiophen-2-yl)silane 1o

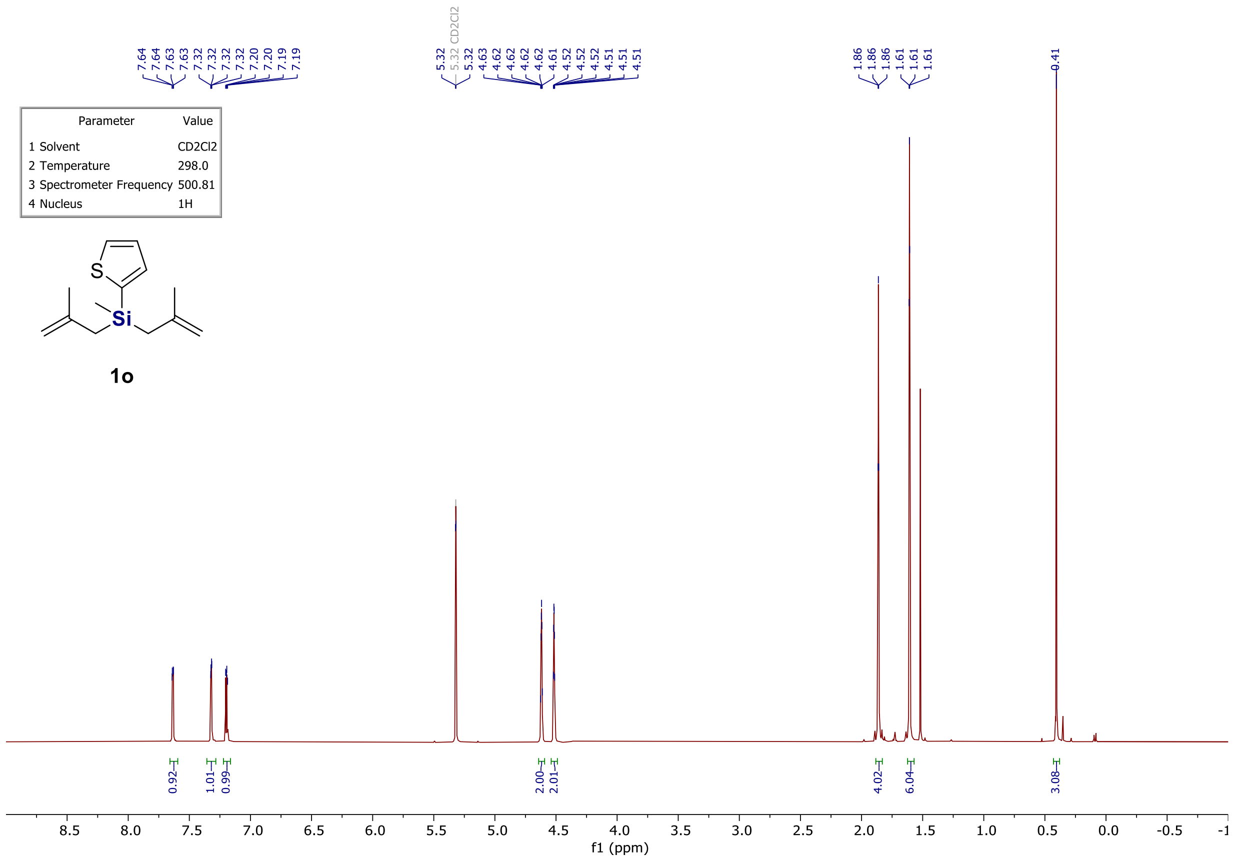

Parameter	Value
1 Solvent	CD2Cl2
2 Temperature	298.0
3 Spectrometer Frequency	500.81
4 Nucleus	1H

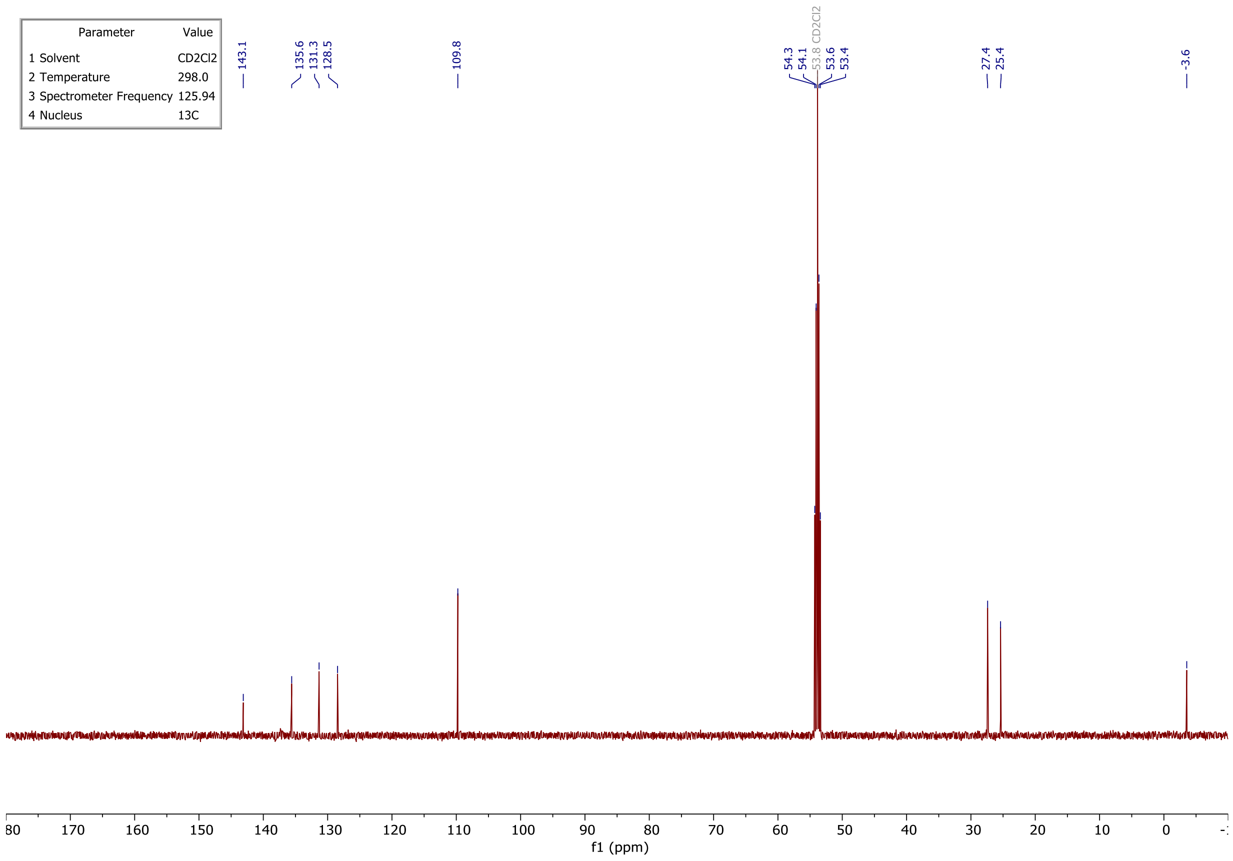

Parameter	Value
1 Solvent	CD2Cl2
2 Temperature	298.0
3 Spectrometer Frequency	125.94
4 Nucleus	13C

methyltris(2-methylallyl)silane 1p

Parameter	Value
1 Solvent	CD2Cl2
2 Temperature	294.9
3 Spectrometer Frequency	500.81
4 Nucleus	1H

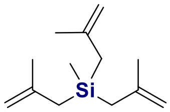

1p

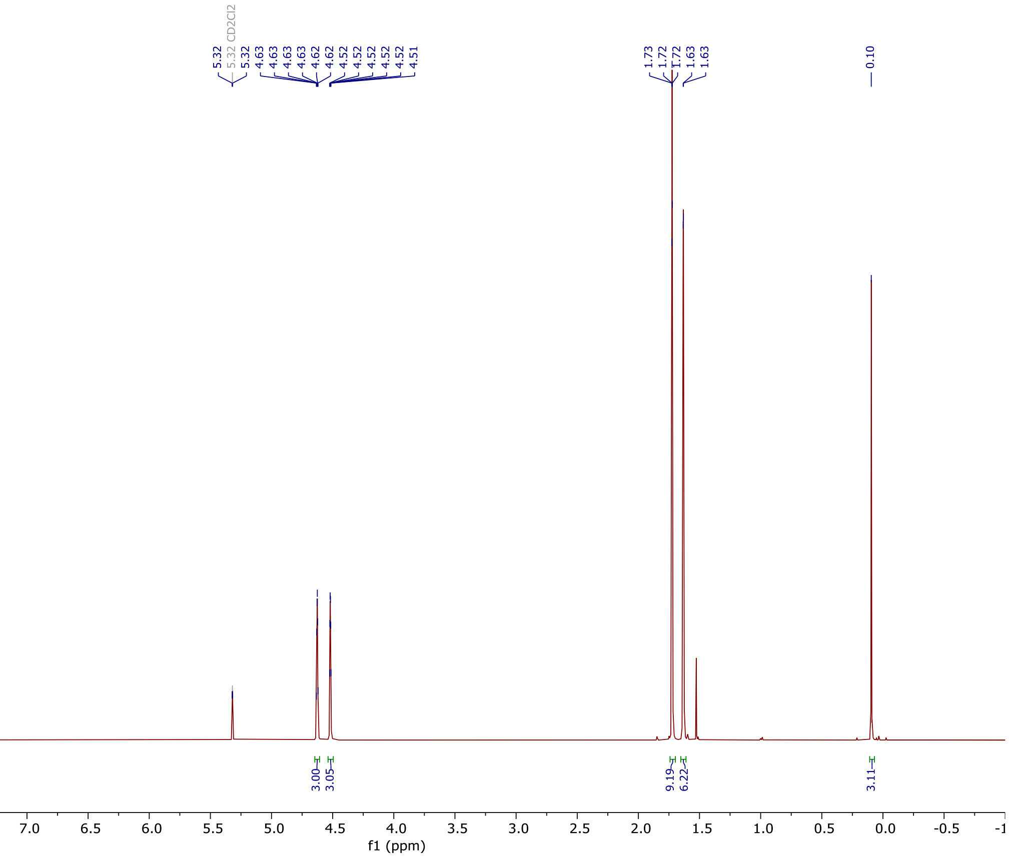

Parameter	Value
1 Solvent	CD2Cl2
2 Temperature	297.1
3 Spectrometer Frequency	125.94
4 Nucleus	13C

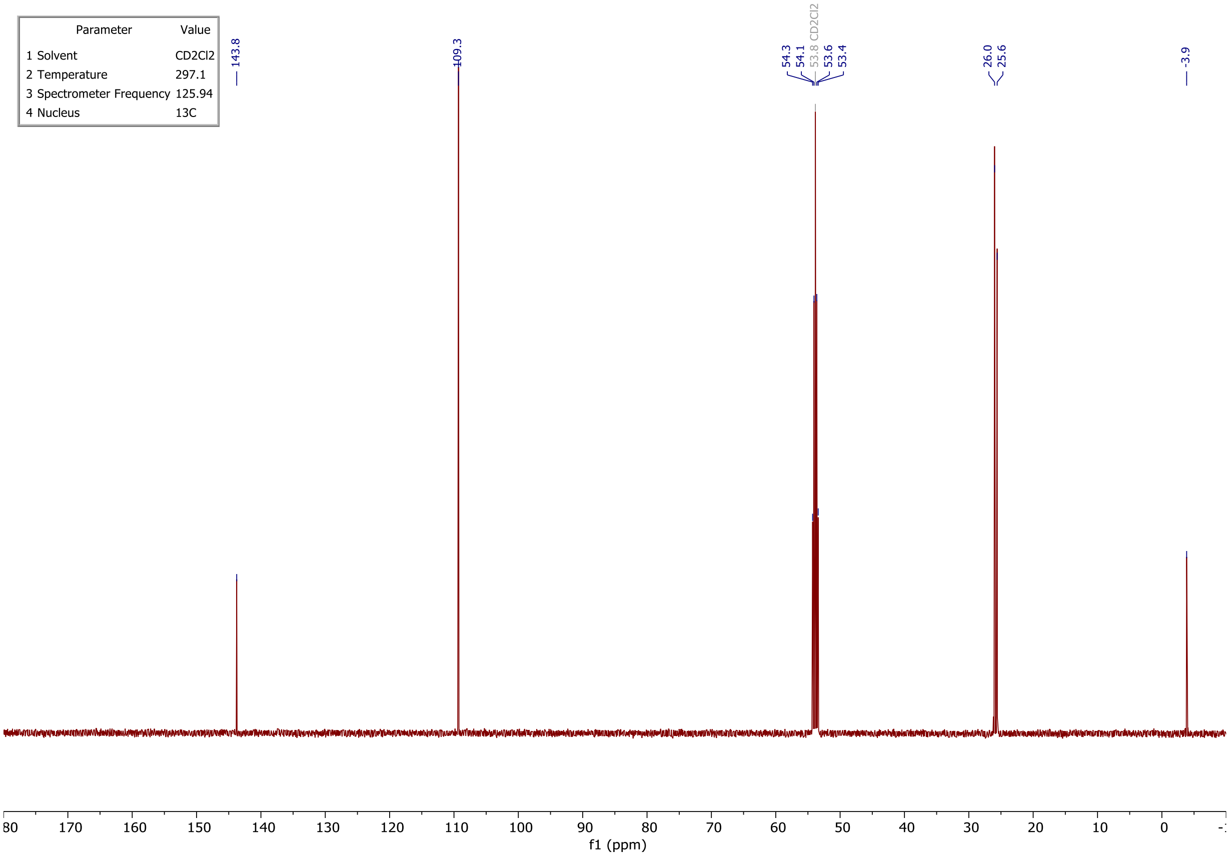

benzyl(ethyl)bis(2-methylallyl)silane 1q

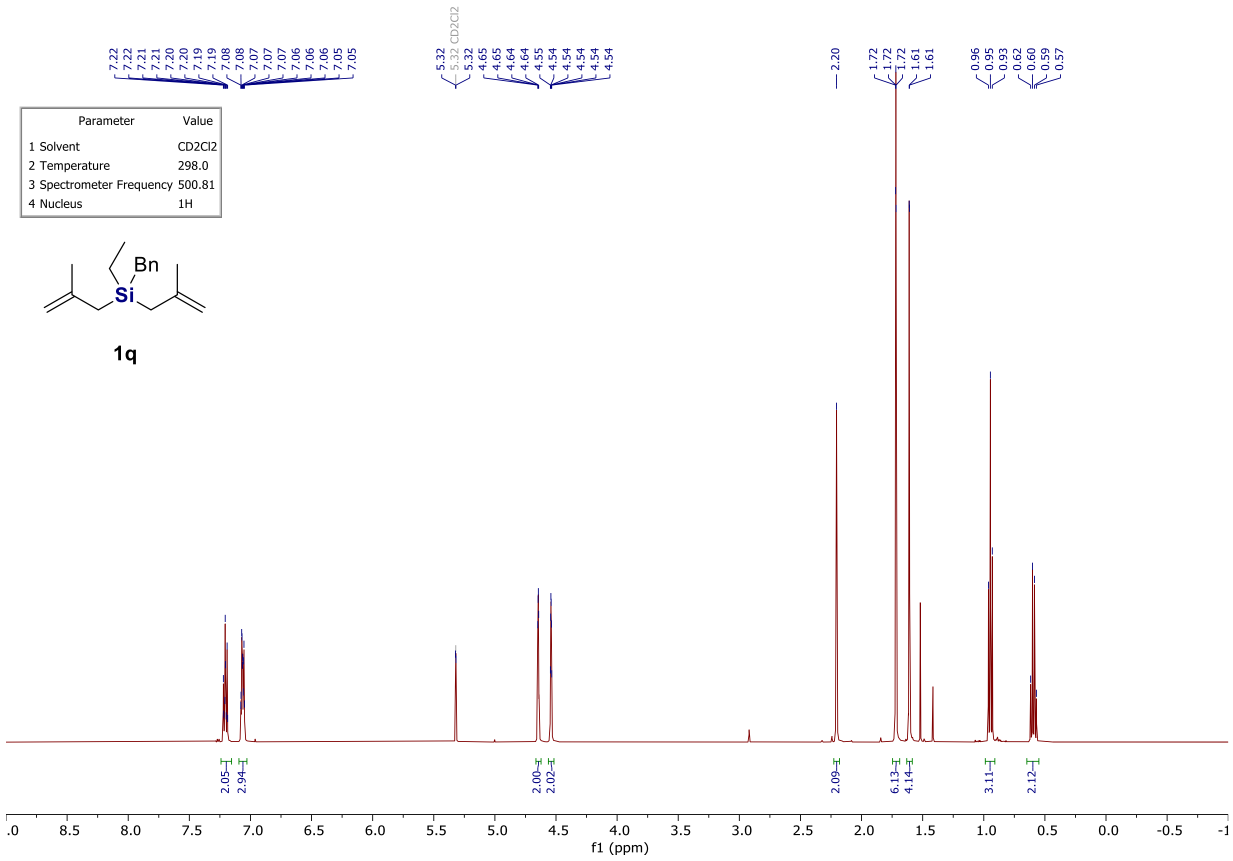

Parameter	Value
1 Solvent	CD2Cl2
2 Temperature	298.0
3 Spectrometer Frequency	500.81
4 Nucleus	1H

1q

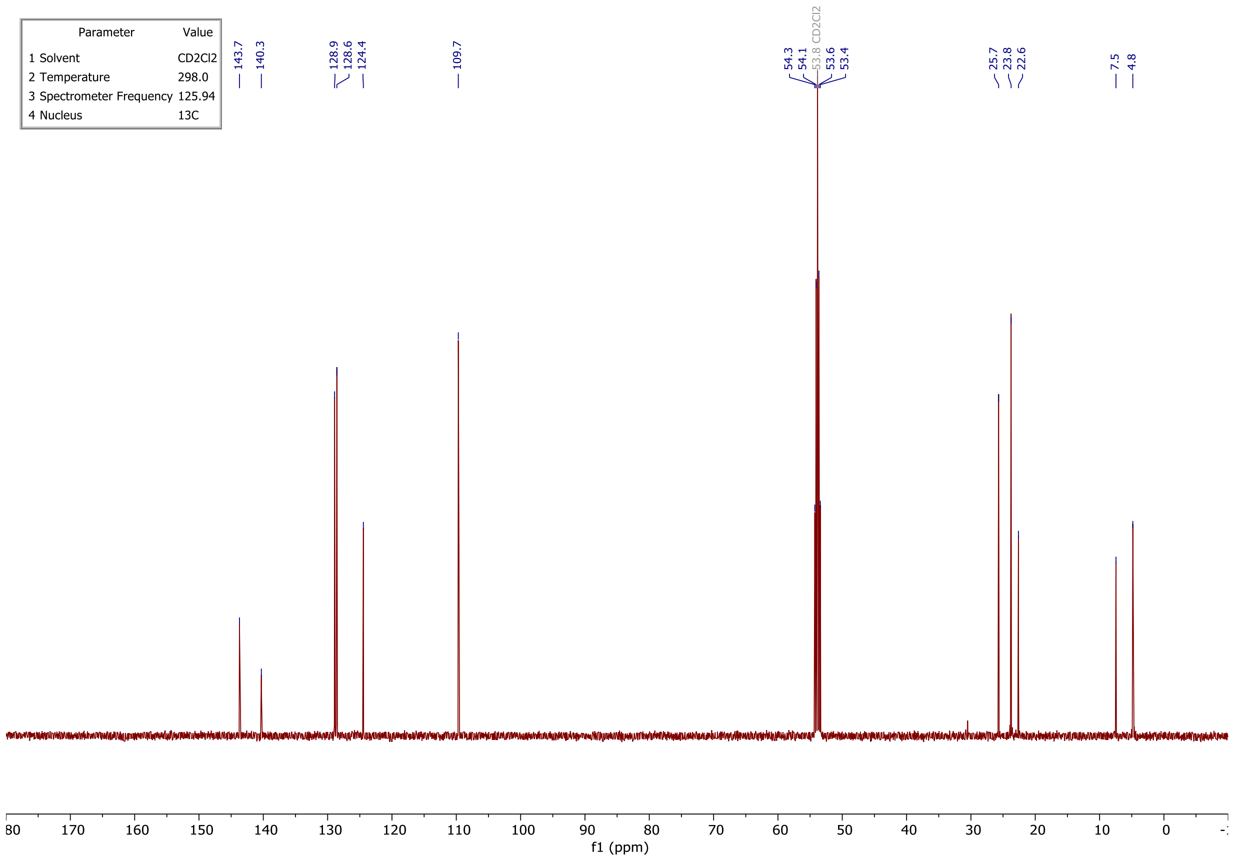

Parameter	Value
1 Solvent	CD2Cl2
2 Temperature	298.0
3 Spectrometer Frequency	125.94
4 Nucleus	13C

benzyl(2,6-dimethylphenoxy)(methyl)(2,2,4-trimethylpent-4-en-1-yl)silane 4a

Parameter	Value
1 Solvent	CD2Cl2
2 Temperature	298.0
3 Spectrometer Frequency	500.81
4 Nucleus	1H

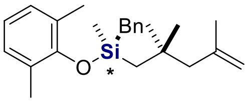

4a

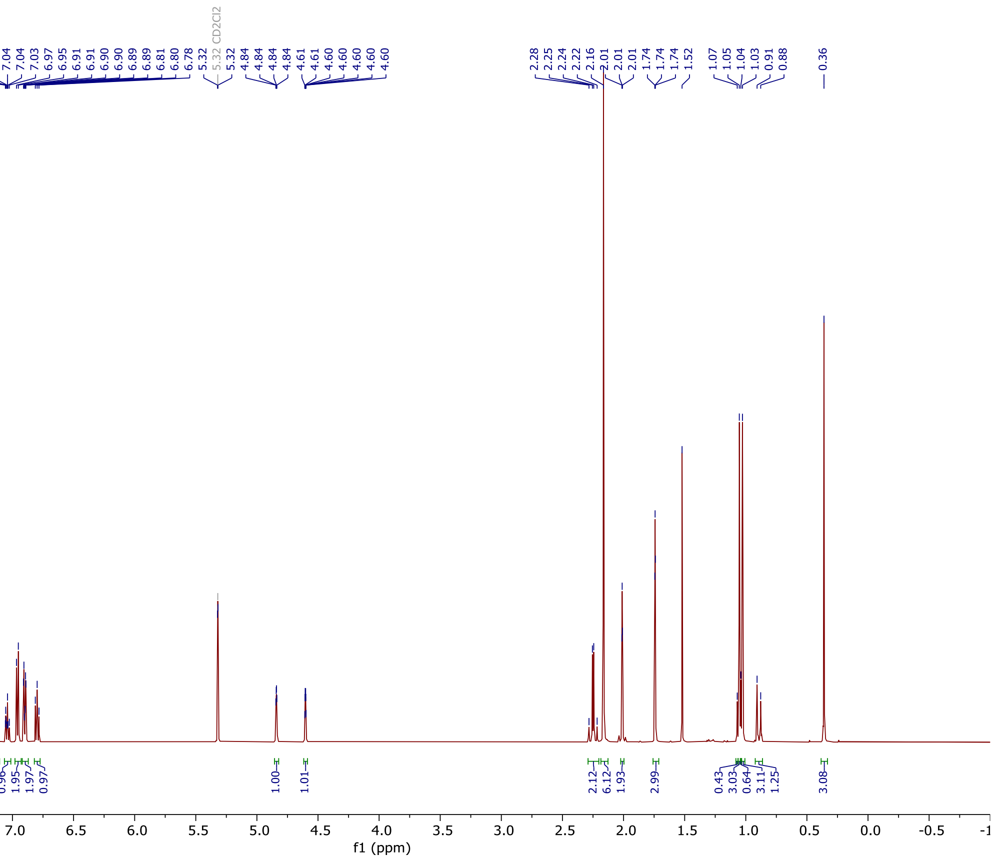

Parameter	Value
1 Solvent	CD2Cl2
2 Temperature	298.0
3 Spectrometer Frequency	125.94
4 Nucleus	13C

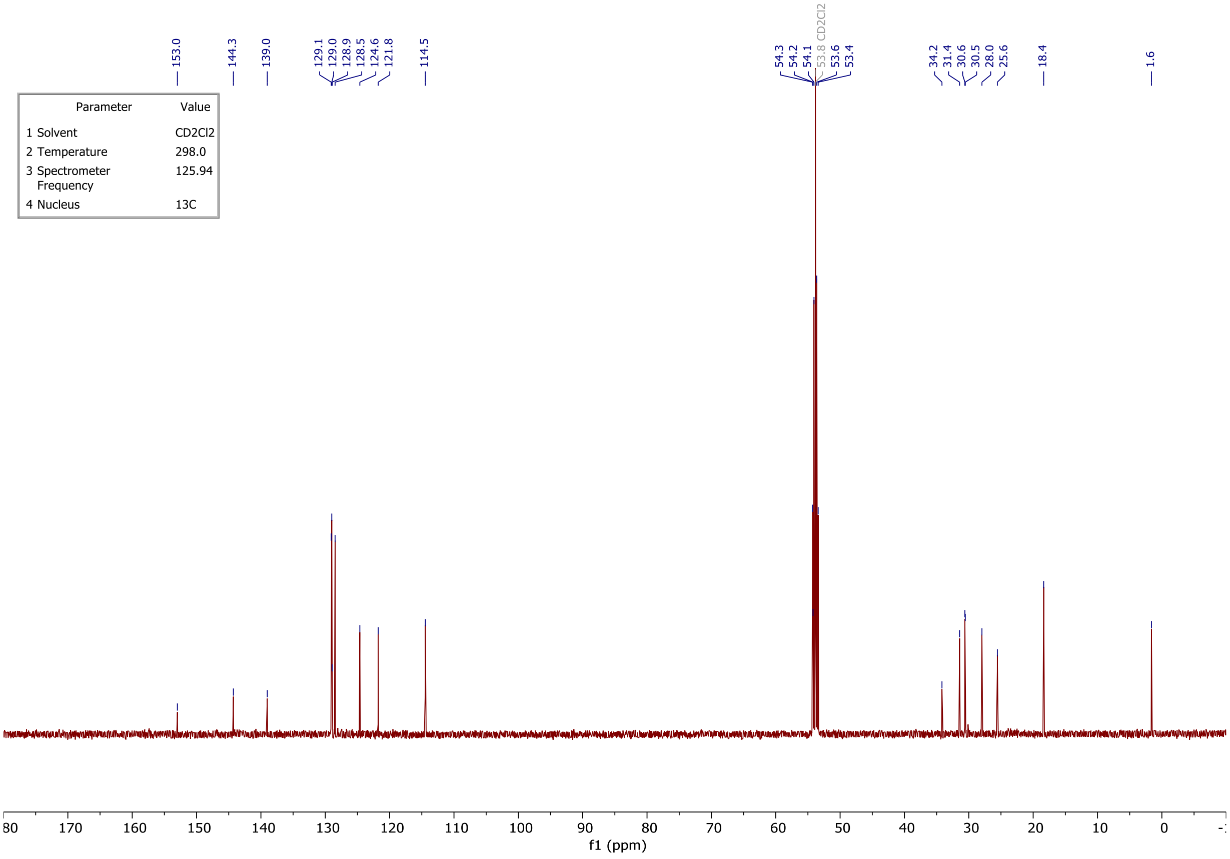

(*R*)-(2,6-dimethylphenoxy)(methyl)(4-methylbenzyl)(2,2,4-trimethylpent-4-en-1-yl)silane 4b

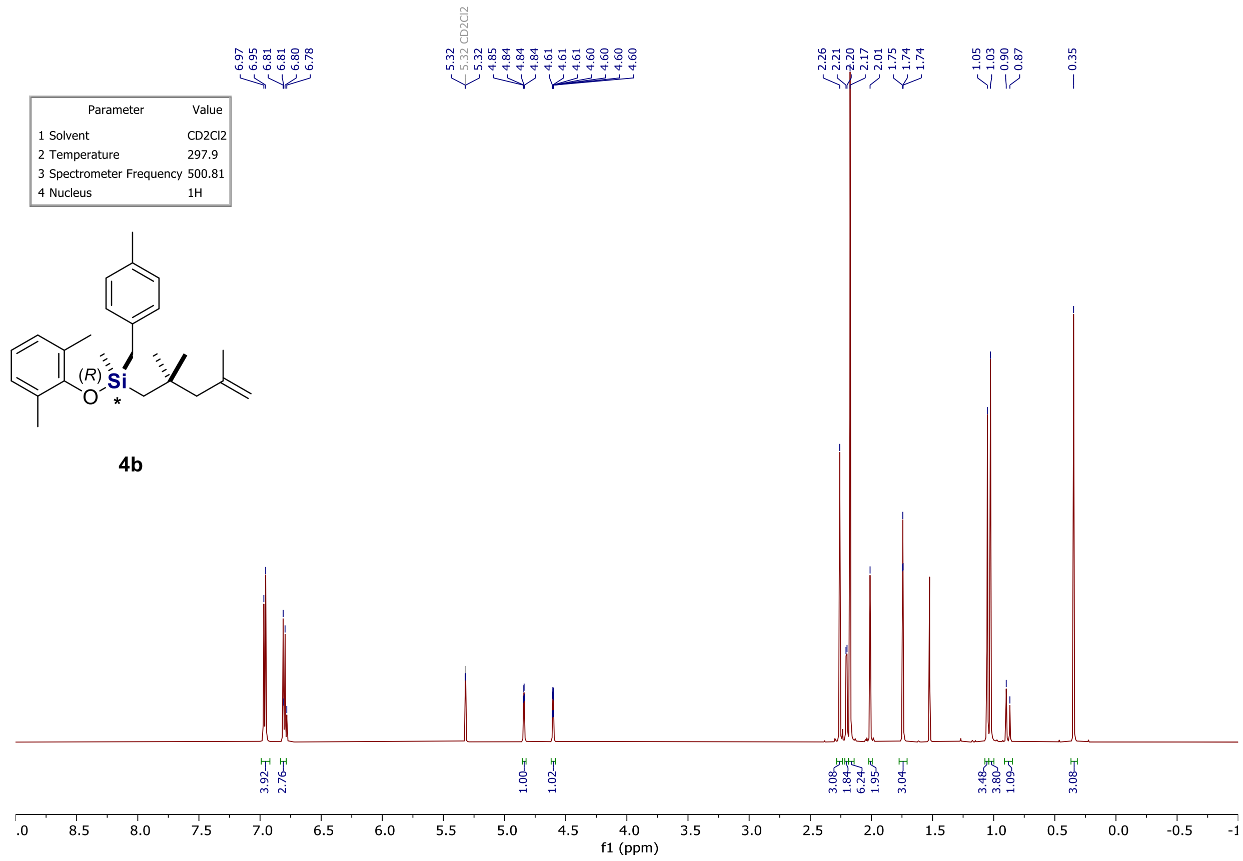

4b

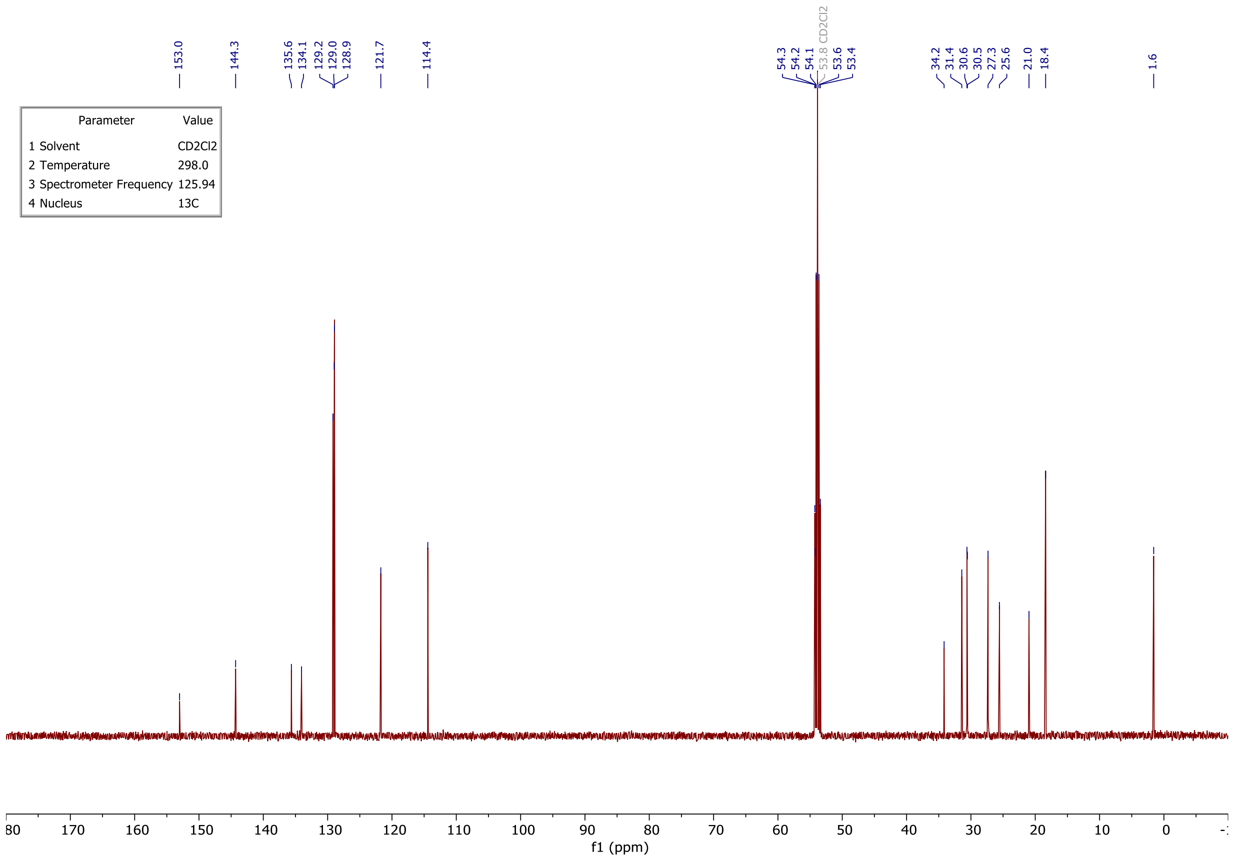

(2,6-dimethylphenoxy)(4-methoxybenzyl)(methyl)(2,2,4-trimethylpent-4-en-1-yl)silane 4c

Parameter	Value
1 Solvent	CD2Cl2
2 Temperature	297.9
3 Spectrometer Frequency	500.81
4 Nucleus	1H

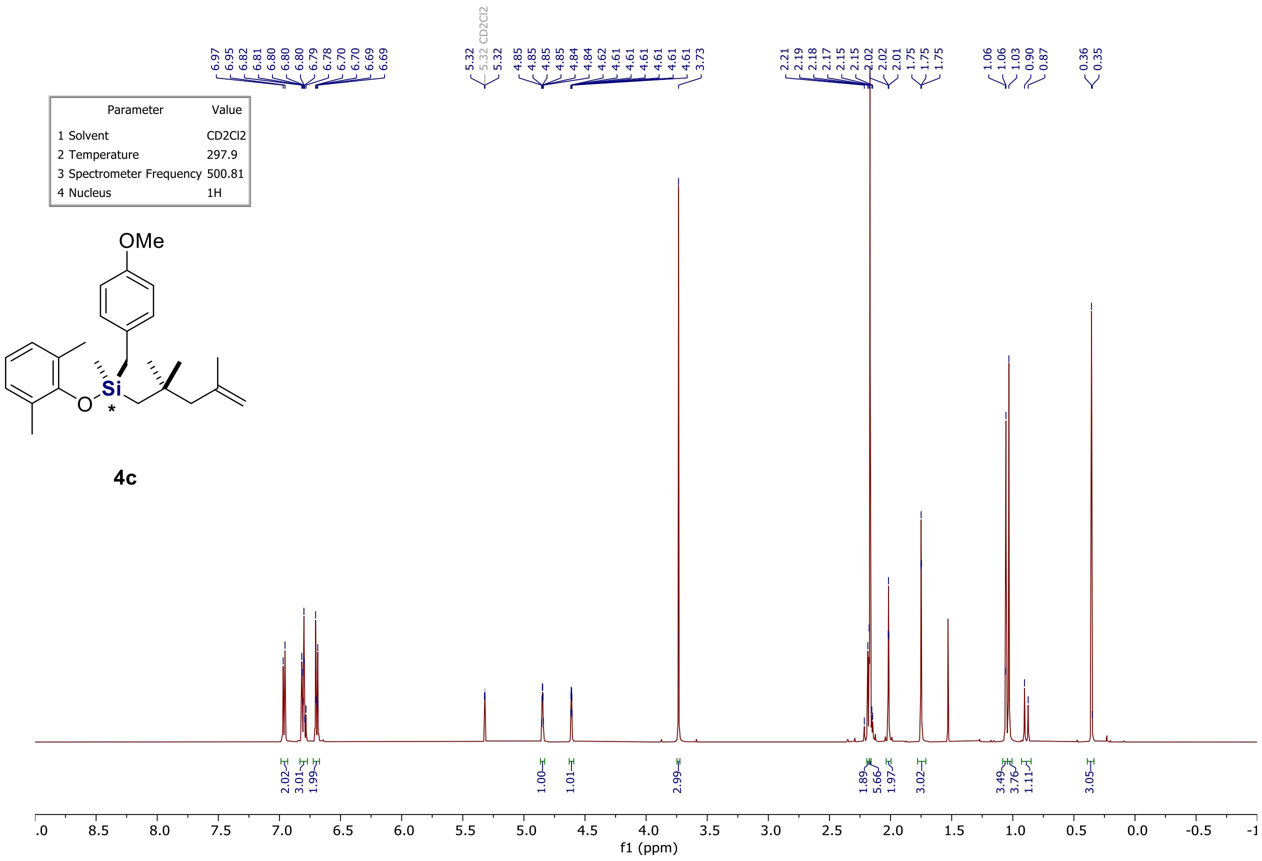

4c

Parameter	Value
1 Solvent	CD2Cl2
2 Temperature	298.0
3 Spectrometer Frequency	125.94
4 Nucleus	13C

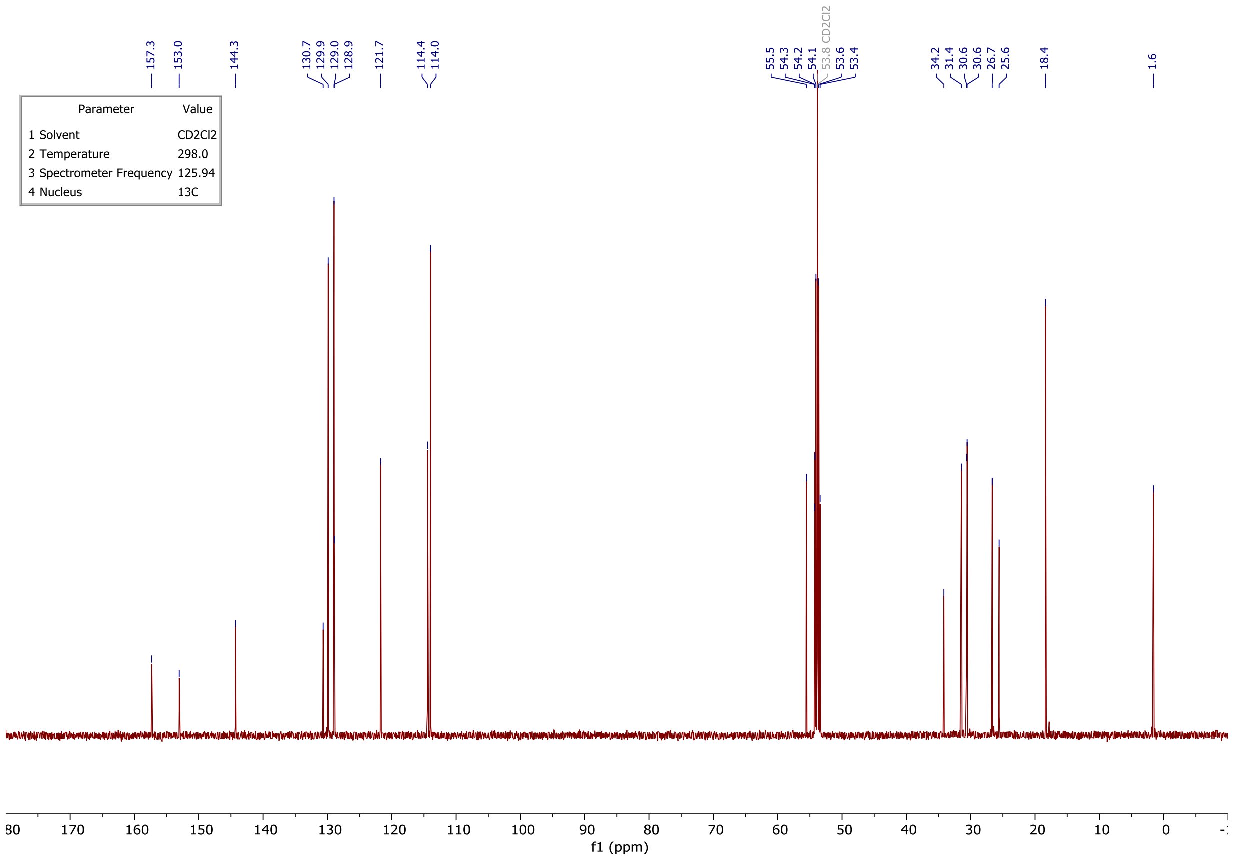

(2,6-dimethylphenoxy)(4-fluorobenzyl)(methyl)(2,2,4-trimethylpent-4-en-1-yl)silane 4d

Parameter	Value
1 Solvent	CD2Cl2
2 Temperature	298.0
3 Spectrometer Frequency	500.81
4 Nucleus	1H

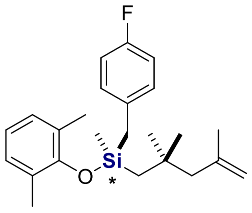

4d

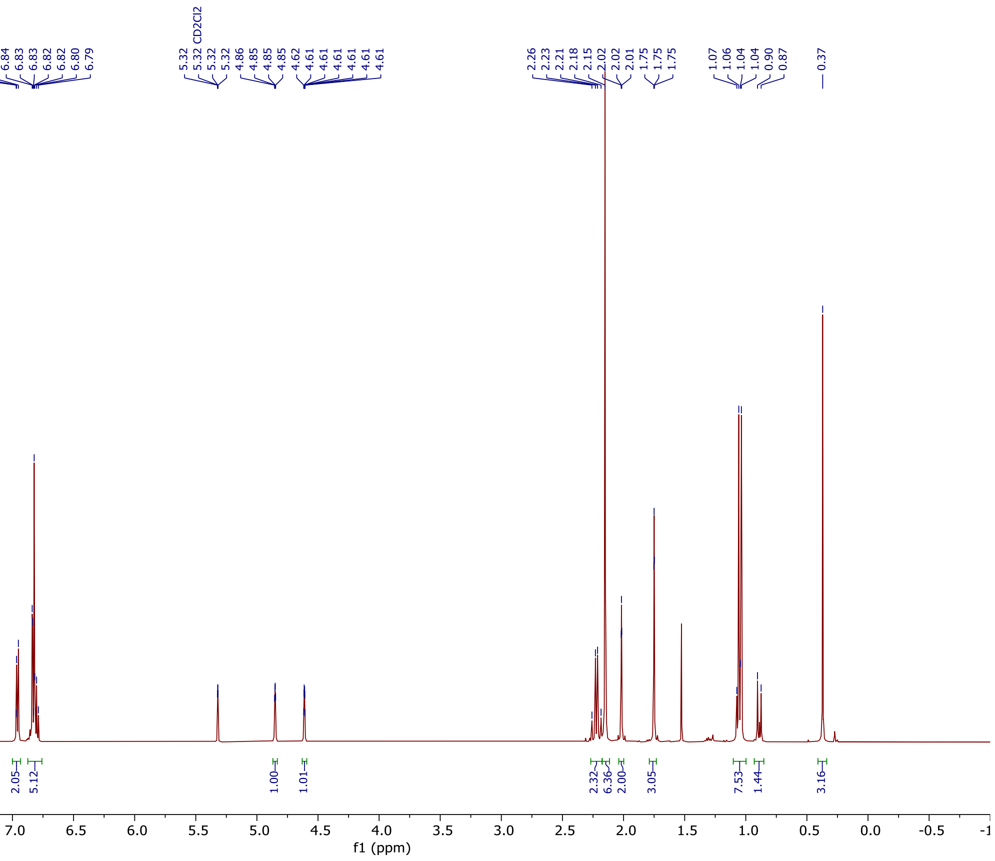

Parameter	Value
1 Solvent	CD2Cl2
2 Temperature	298.0
3 Spectrometer Frequency	471.21
4 Nucleus	19F

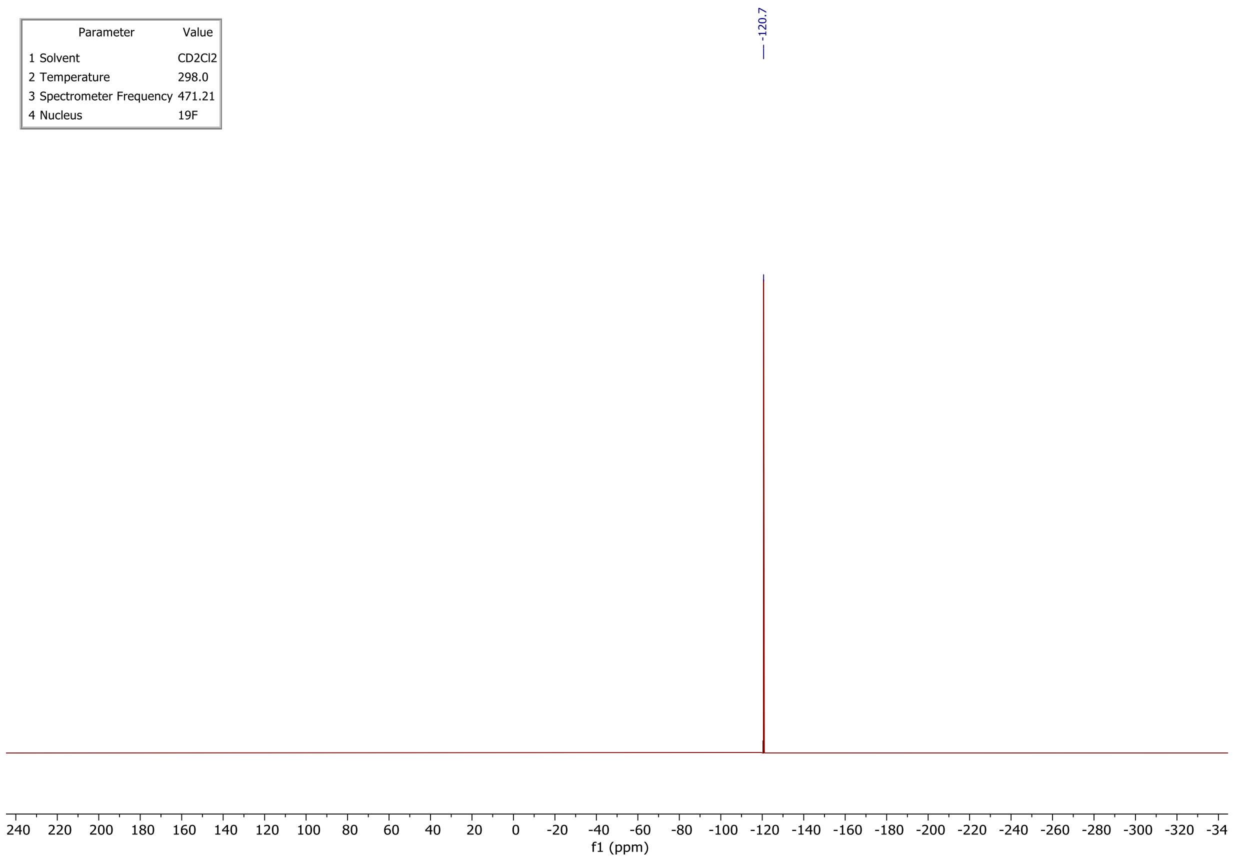

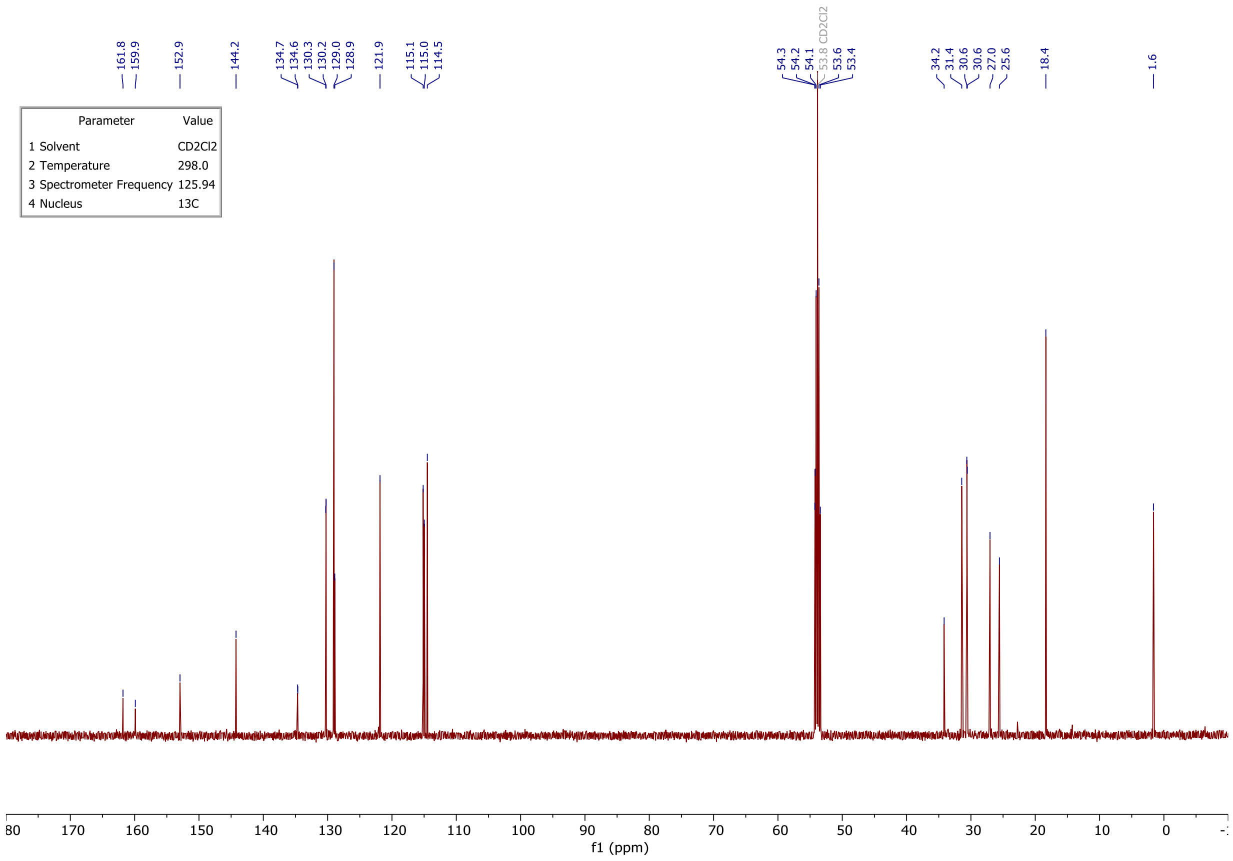

(2,6-dimethylphenoxy)(methyl)(3-methylbenzyl)(2,2,4-trimethylpent-4-en-1-yl)silane 4e

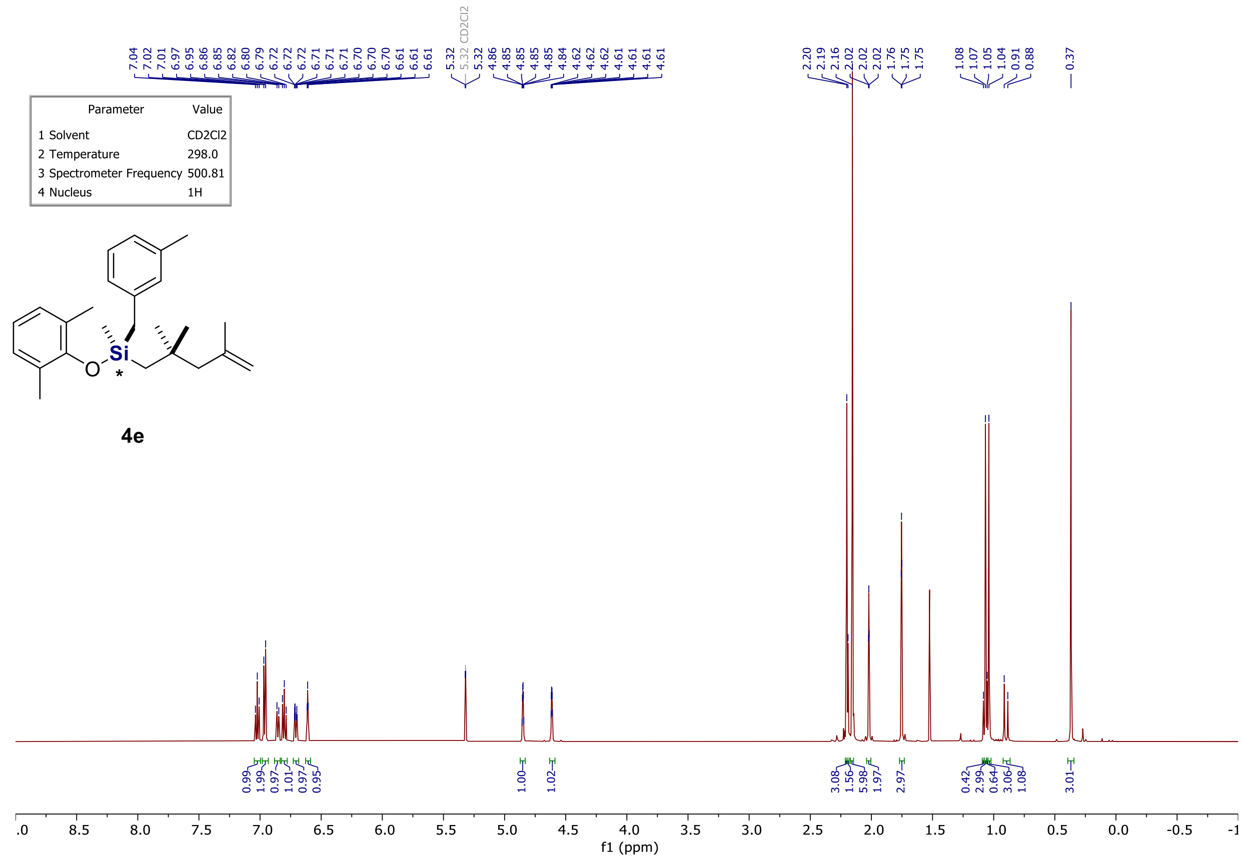

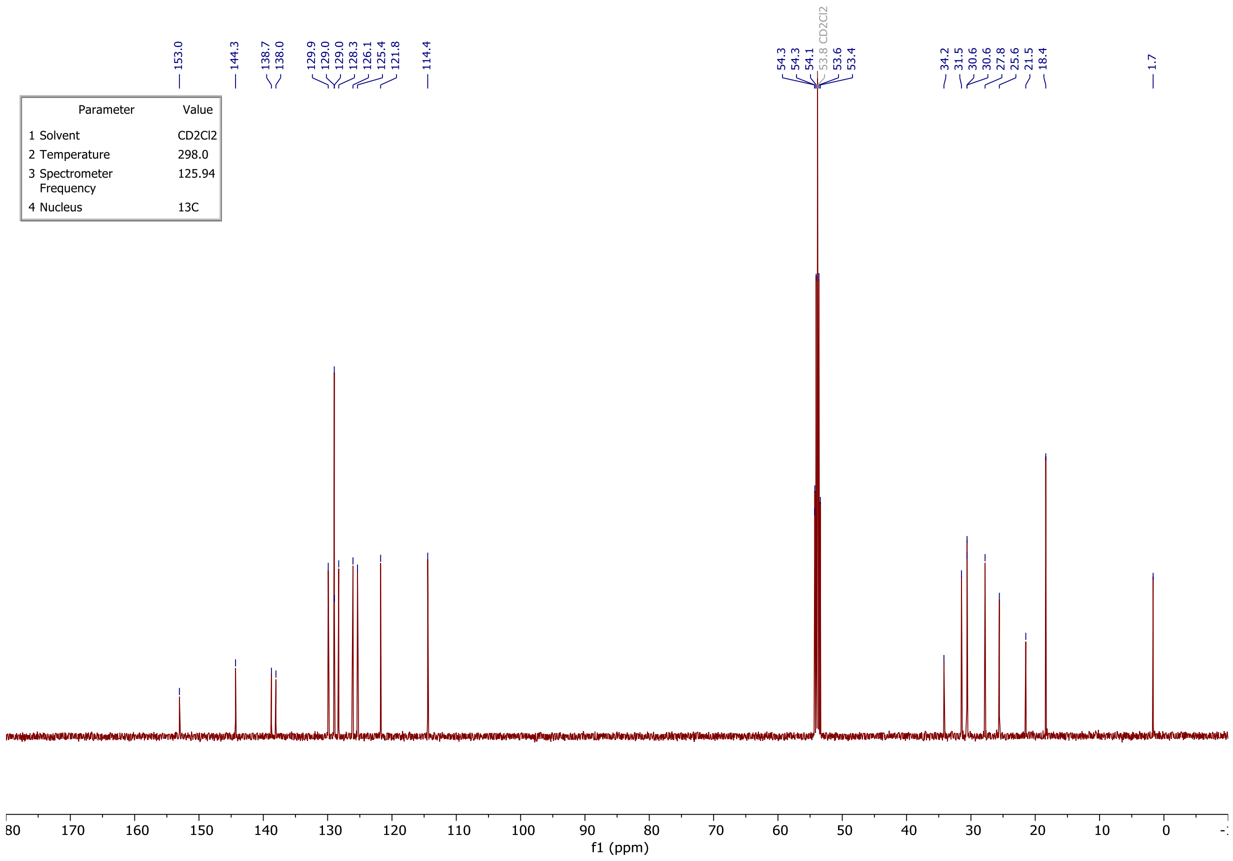

(2,6-dimethylphenoxy)(3-methoxybenzyl)(methyl)(2,2,4-trimethylpent-4-en-1-yl)silane 4f

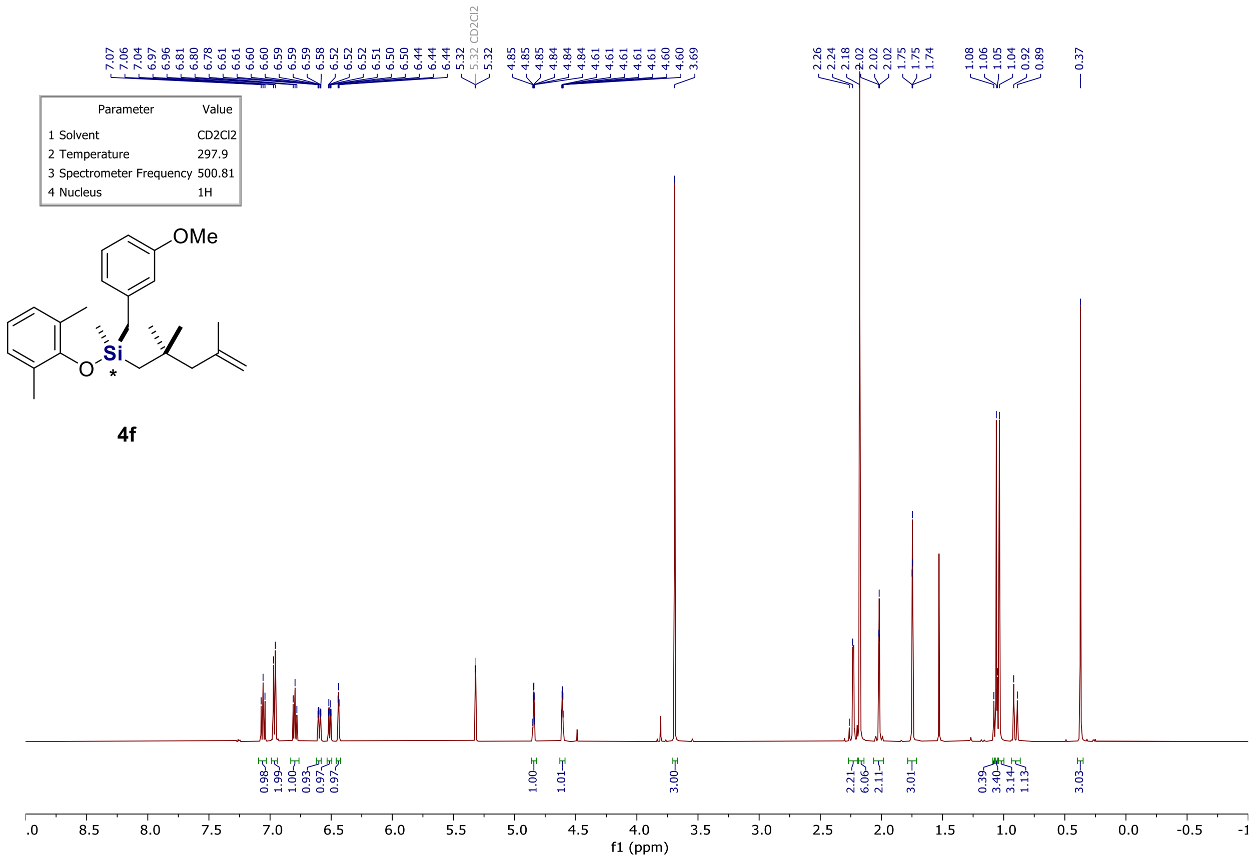

4f

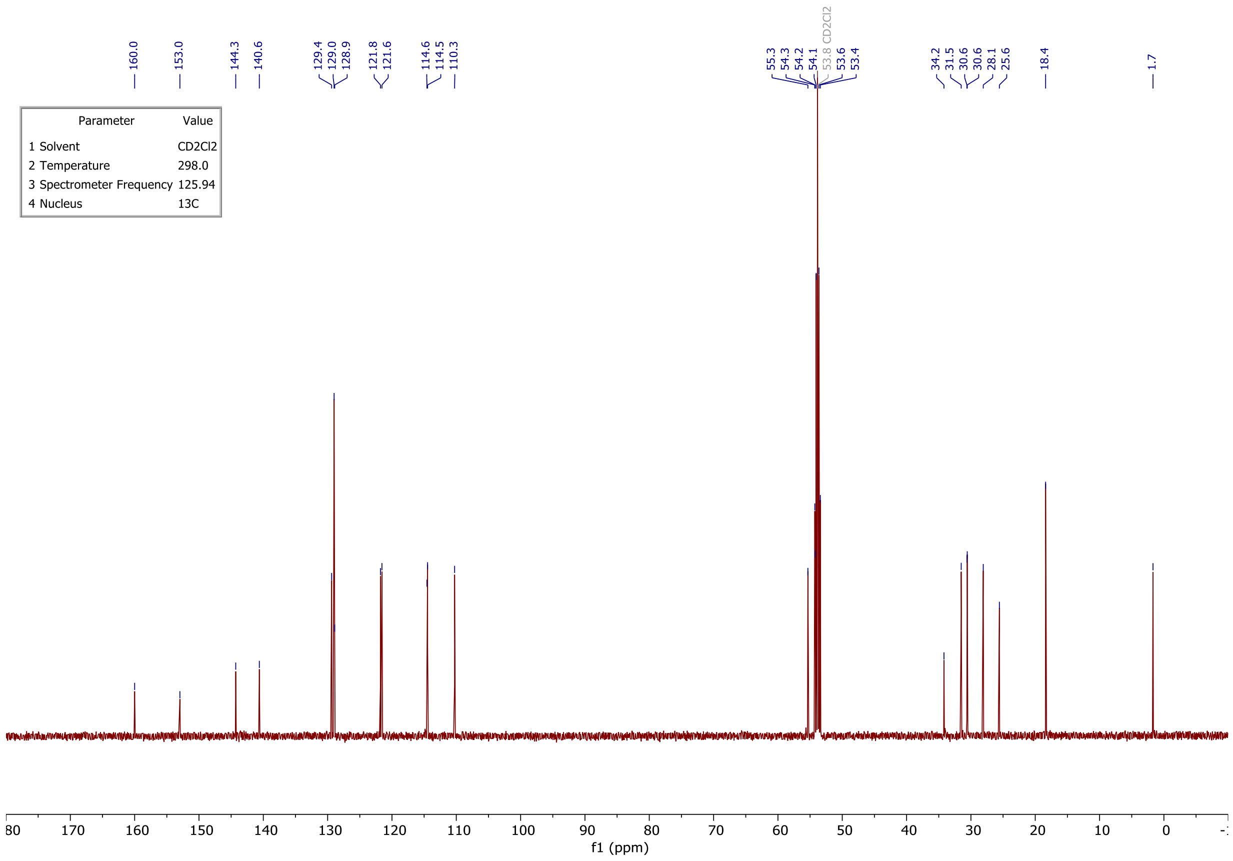

(2,6-dimethylphenoxy)(3-fluorobenzyl)(methyl)(2,2,4-trimethylpent-4-en-1-yl)silane 4g

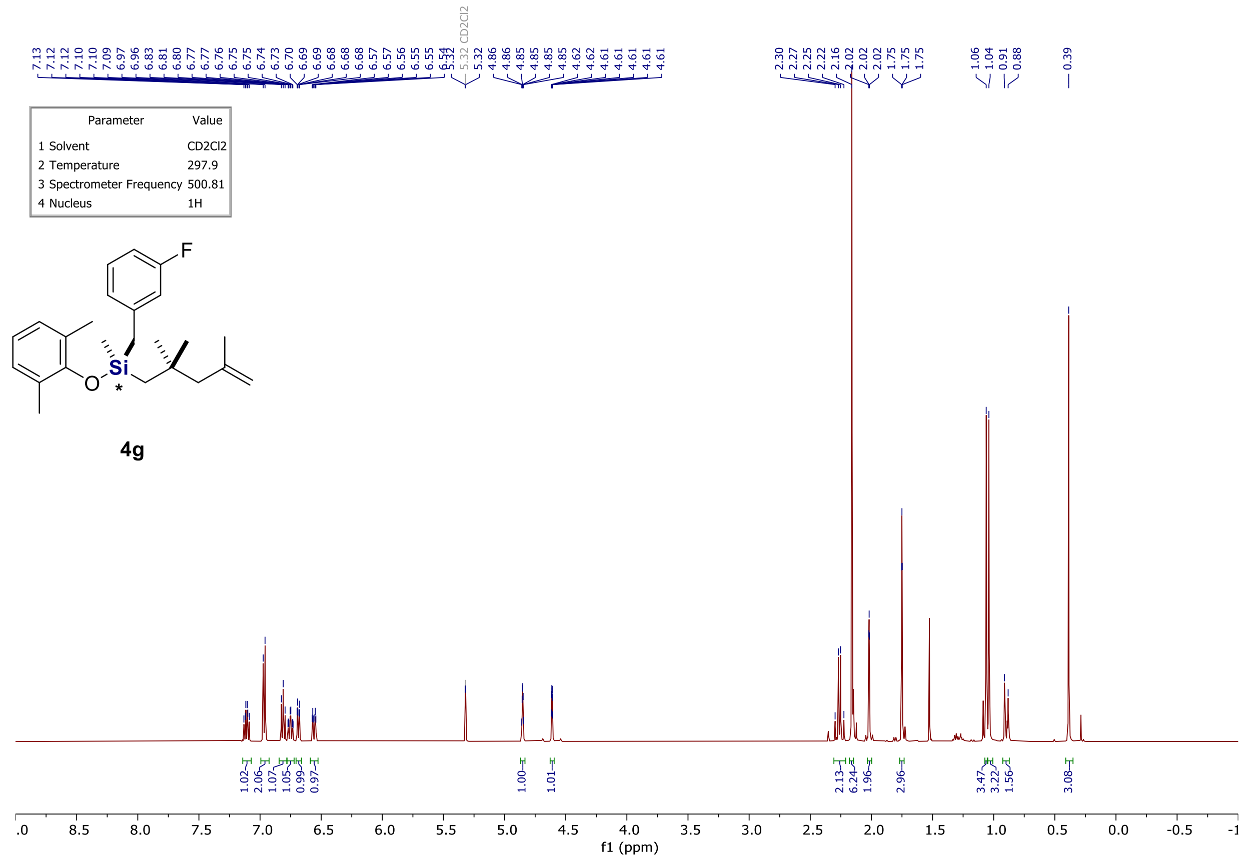

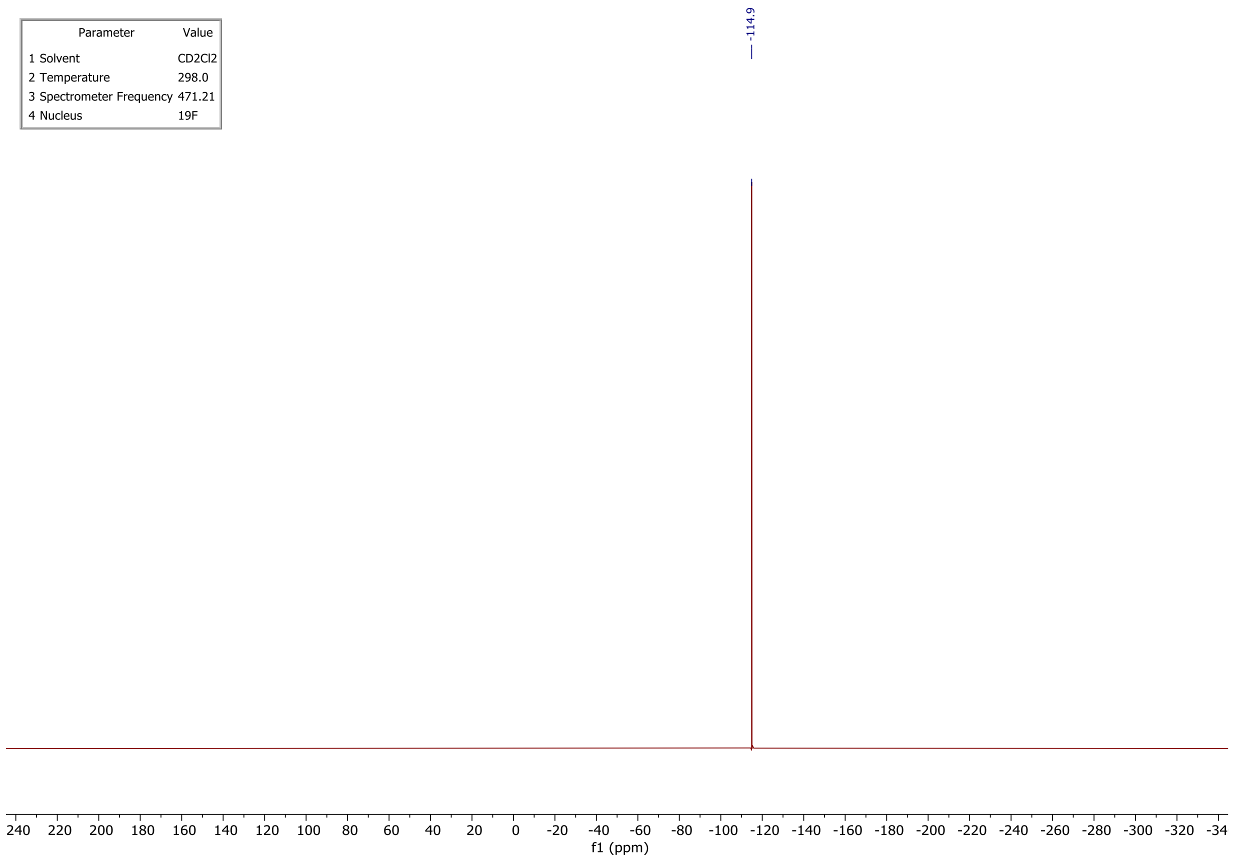

-114.9
Parameter Value
1 Solvent CD2Cl2
2 Temperature 298.0
3 Spectrometer Frequency 471.21
4 Nucleus 19F
f1 (ppm)

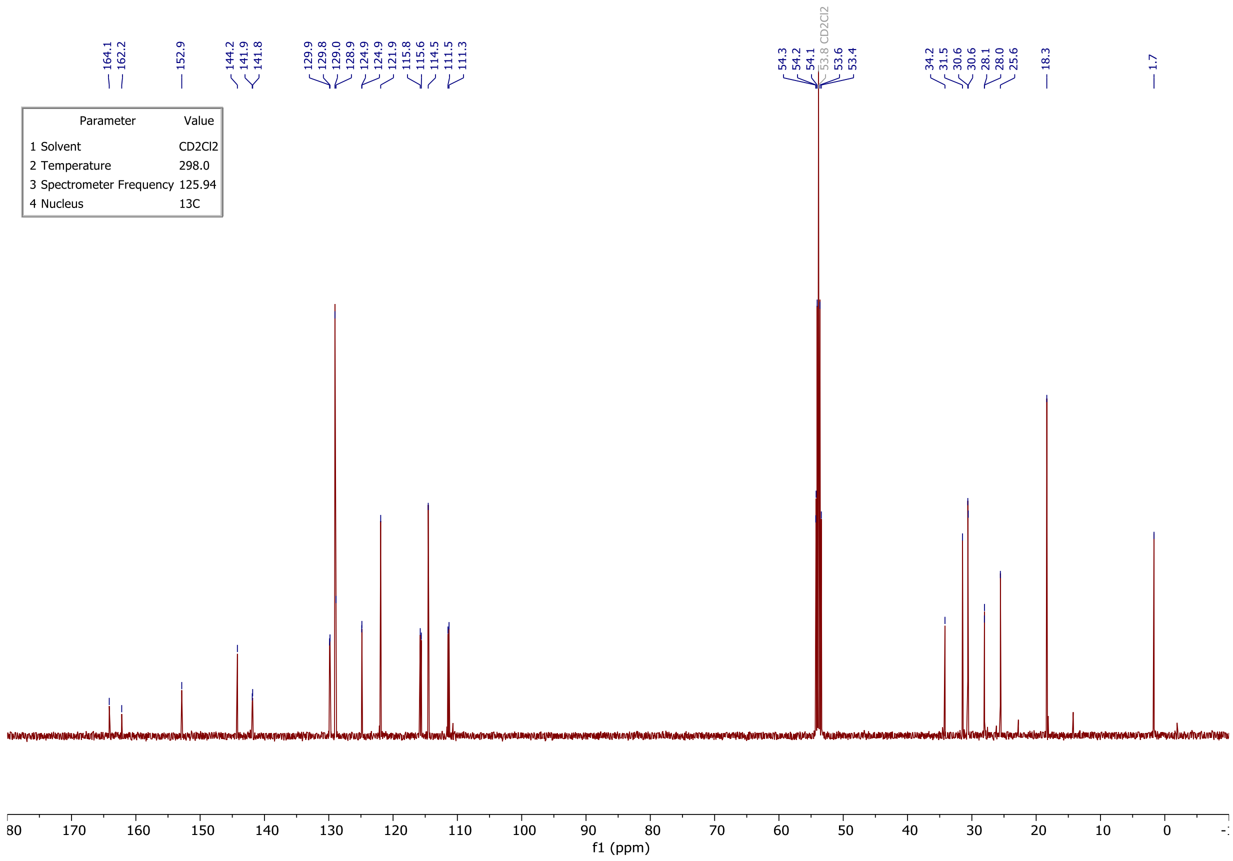

164.1
162.2
152.9
144.2
141.9
141.8
129.9
129.8
129.0
128.9
124.9
124.9
121.9
115.8
115.6
114.5
111.5
111.3
54.3
54.2
54.1
53.8 CD2Cl2
53.6
53.4
34.2
31.5
30.6
30.6
28.1
28.0
25.6
18.3
1.7
Parameter Value
1 Solvent CD2Cl2
2 Temperature 298.0
3 Spectrometer Frequency 125.94
4 Nucleus 13C
f1 (ppm)

(2,6-dimethylphenoxy)(methyl)(2-methylbenzyl)(2,2,4-trimethylpent-4-en-1-yl)silane 4h

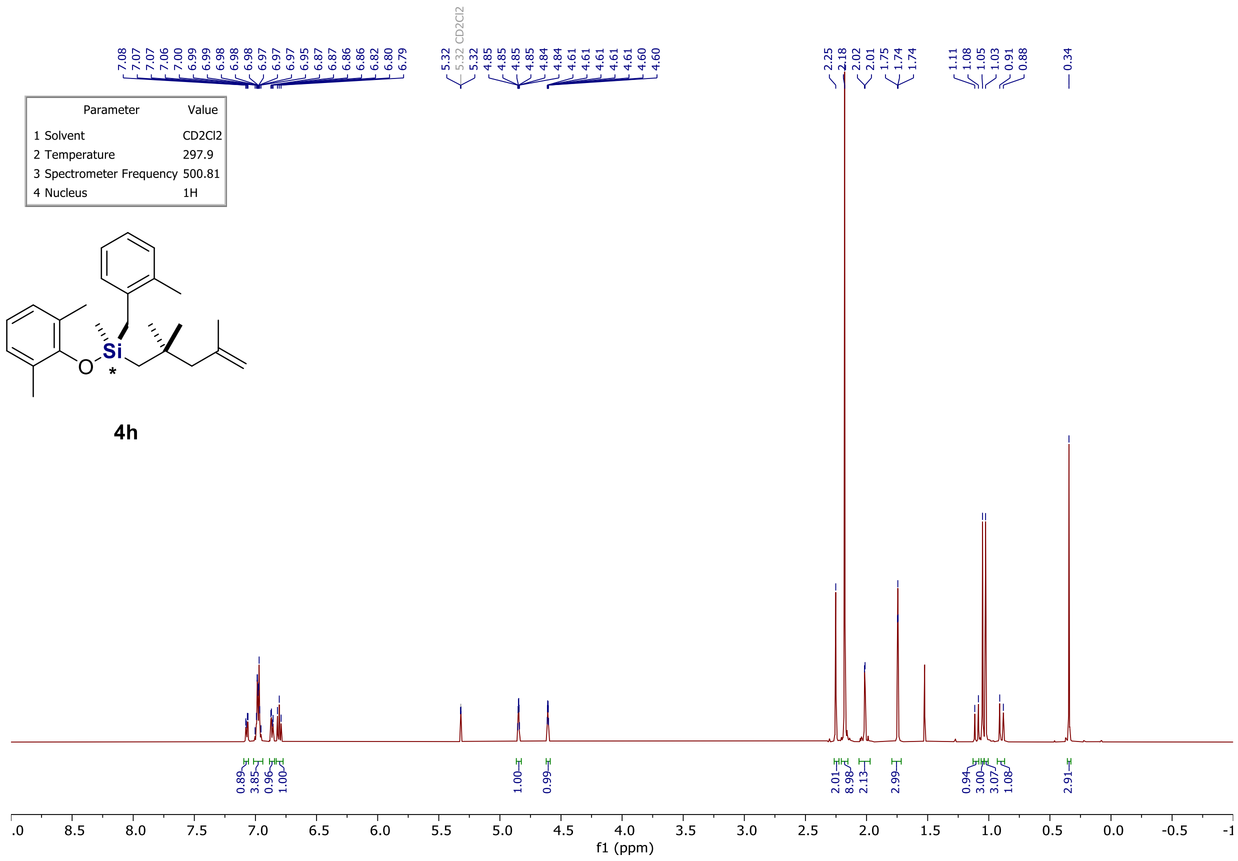

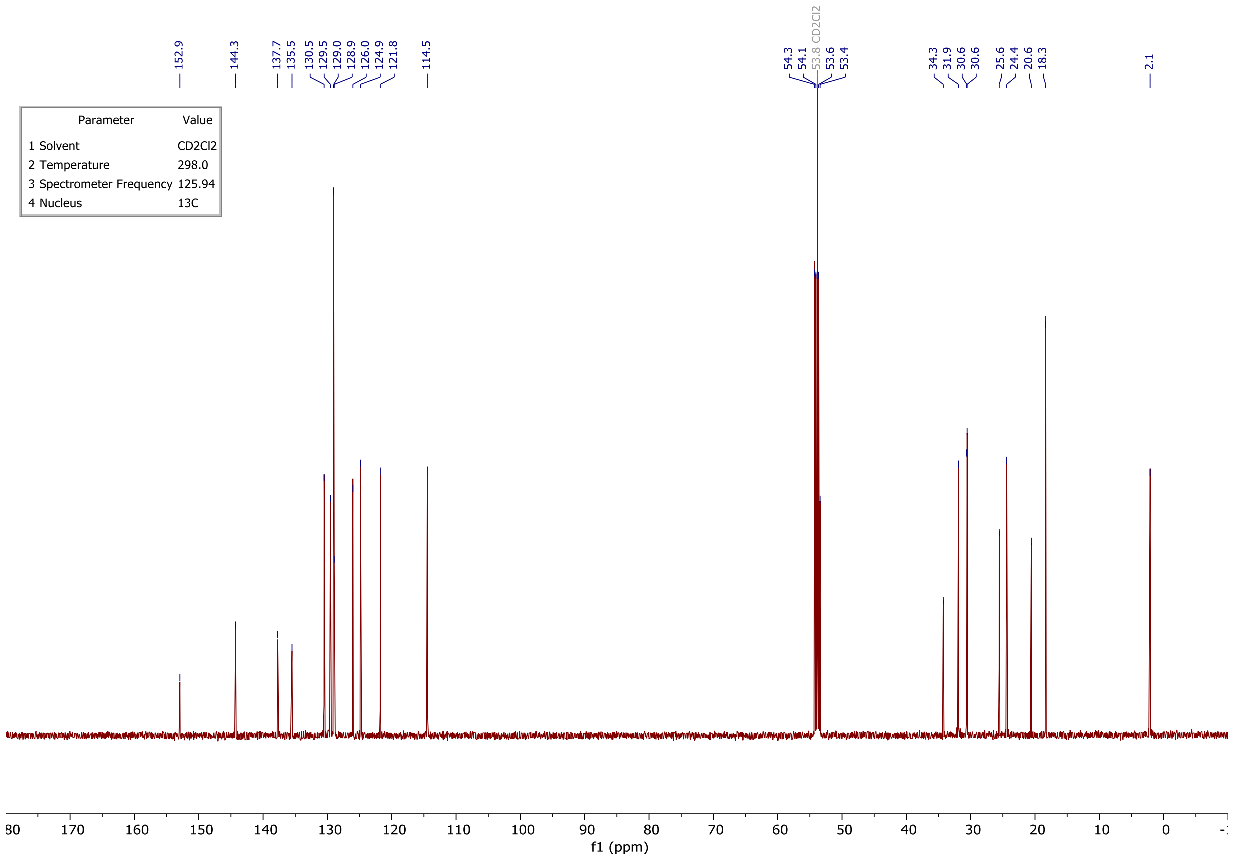

(2,6-dimethylphenoxy)(2-fluorobenzyl)(methyl)(2,2,4-trimethylpent-4-en-1-yl)silane 4i

Parameter	Value
1 Solvent	CD2Cl2
2 Temperature	298.0
3 Spectrometer Frequency	500.81
4 Nucleus	1H

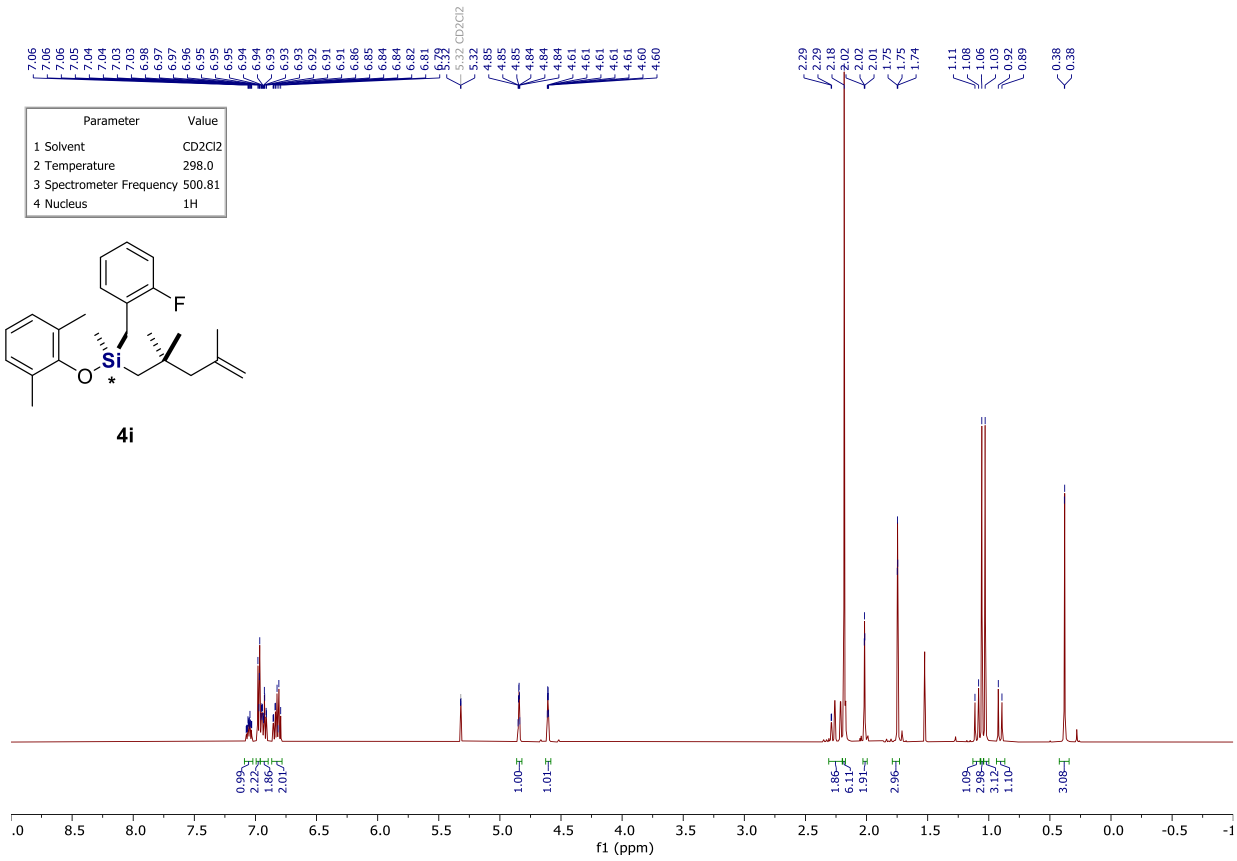

4i

Parameter	Value
1 Solvent	CD2Cl2
2 Temperature	298.0
3 Spectrometer Frequency	471.21
4 Nucleus	19F

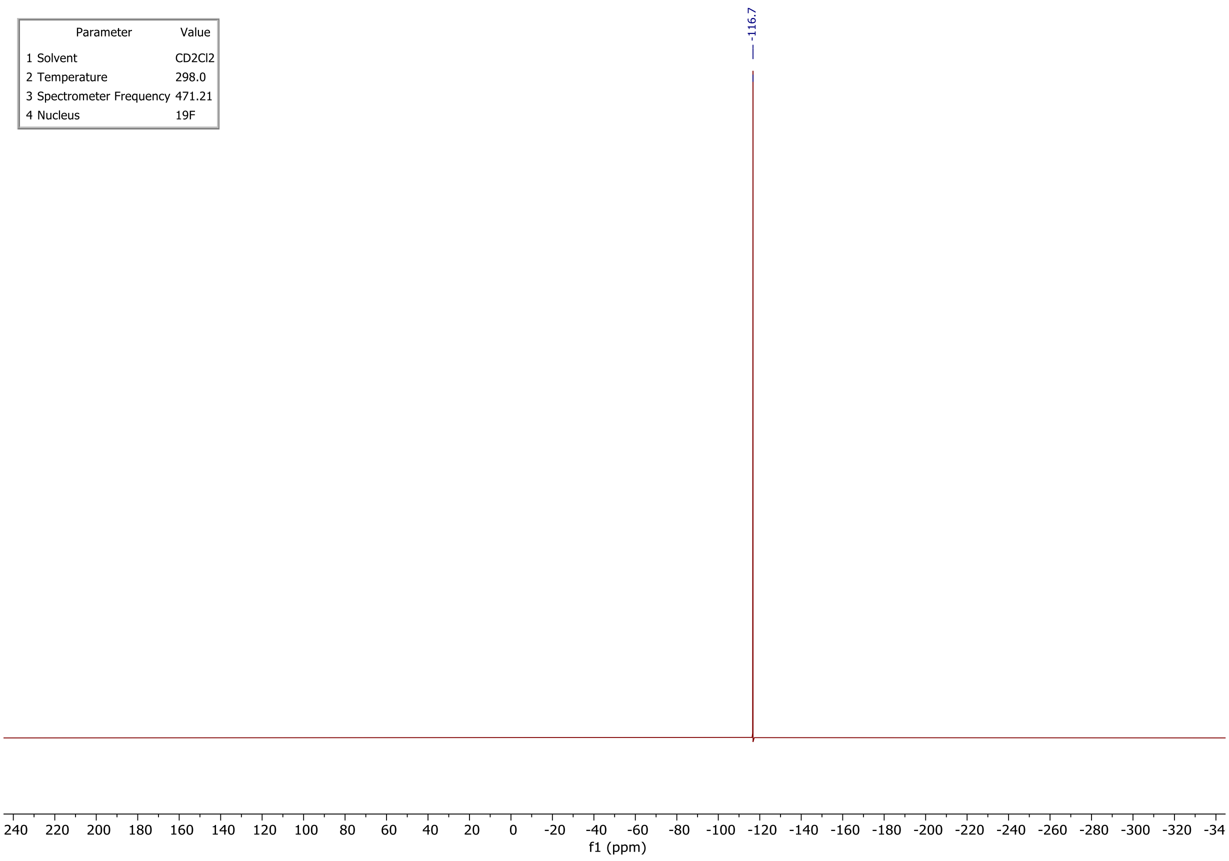

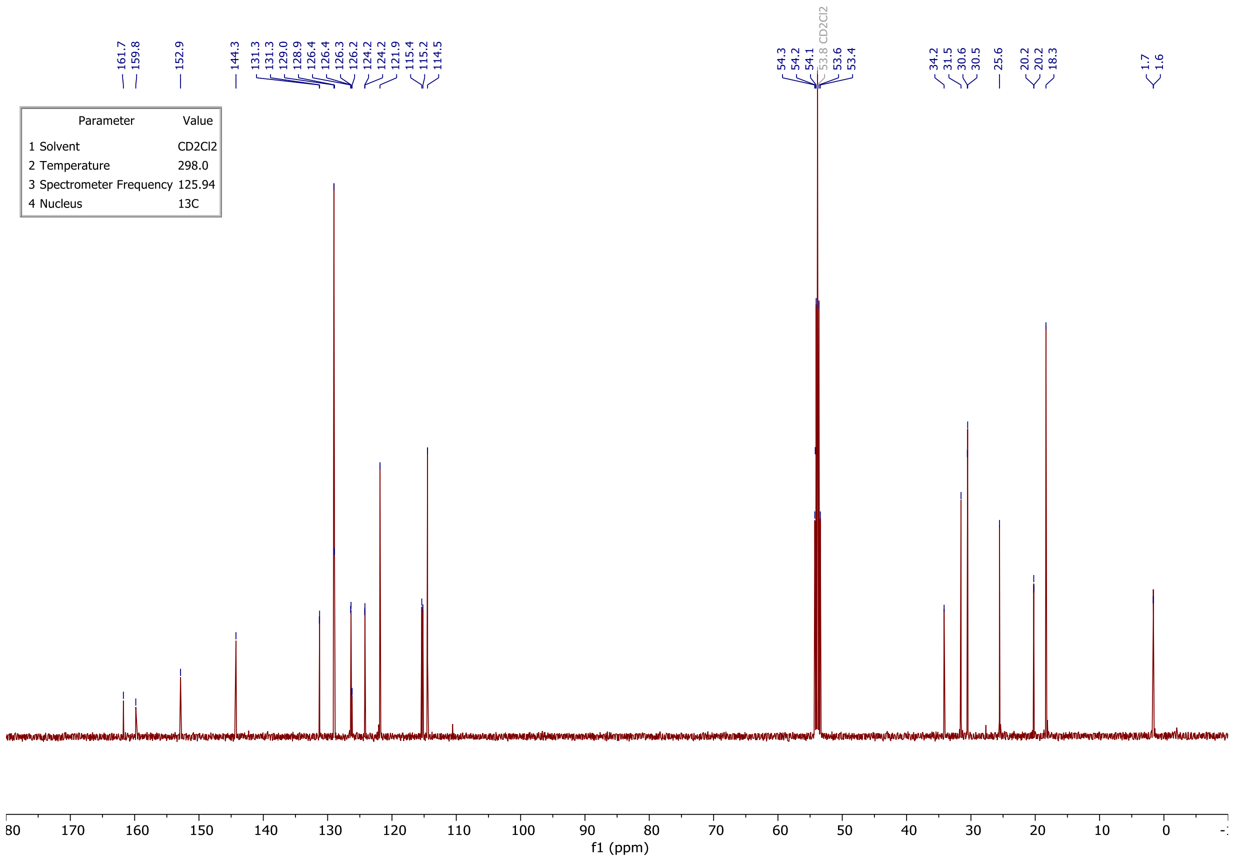

(3,4-dimethylbenzyl)(2,6-dimethylphenoxy)(methyl)(2,2,4-trimethylpent-4-en-1-yl)silane 4j

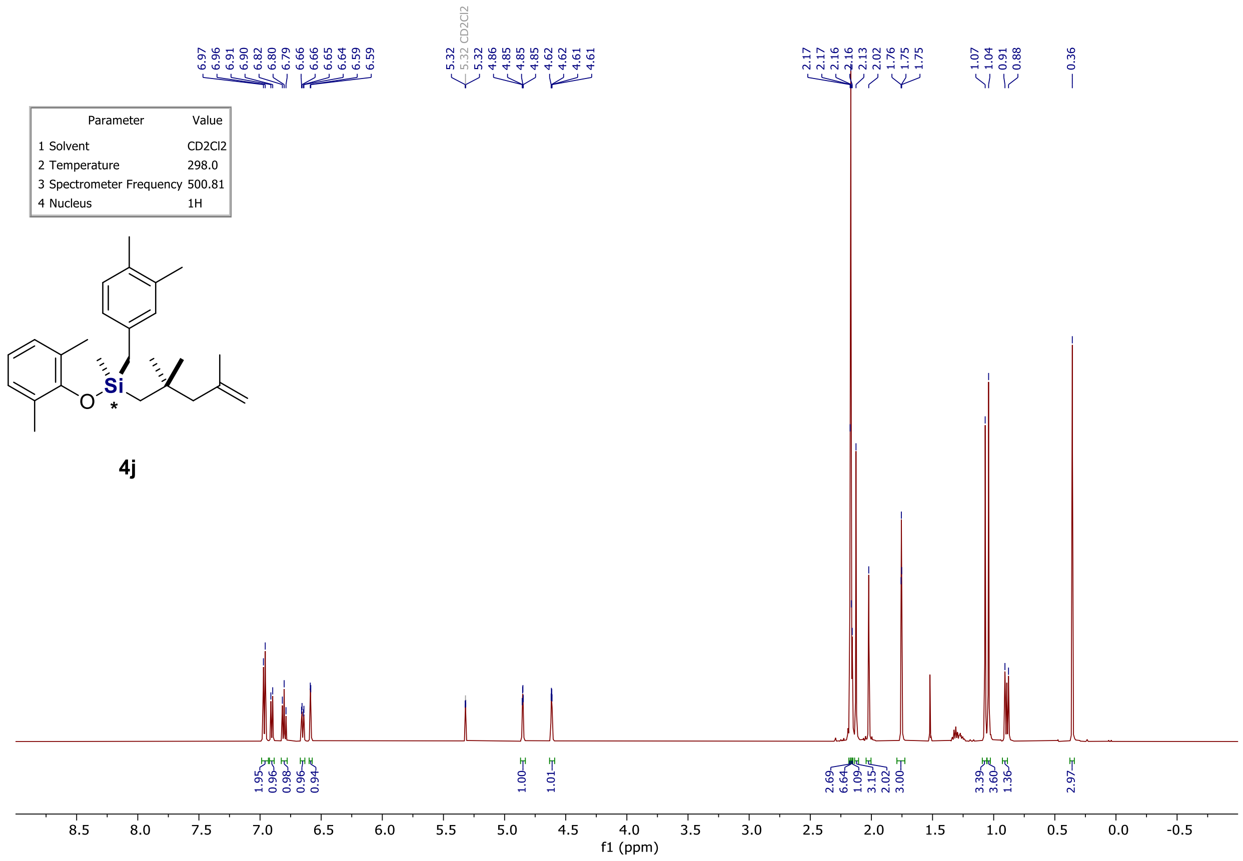

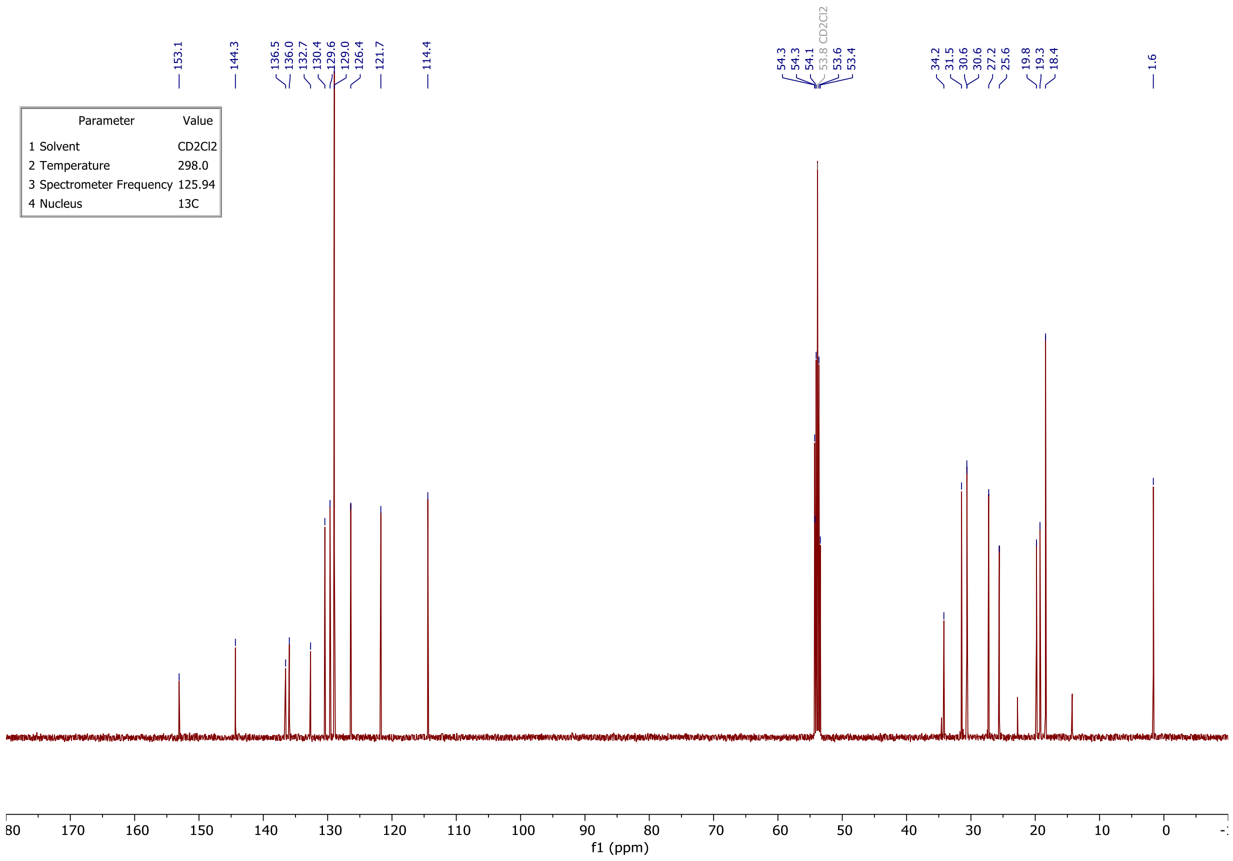

(2,6-dimethylphenoxy)(methyl)(naphthalen-2-ylmethyl)(2,2,4-trimethylpent-4-en-1-yl)silane 4k

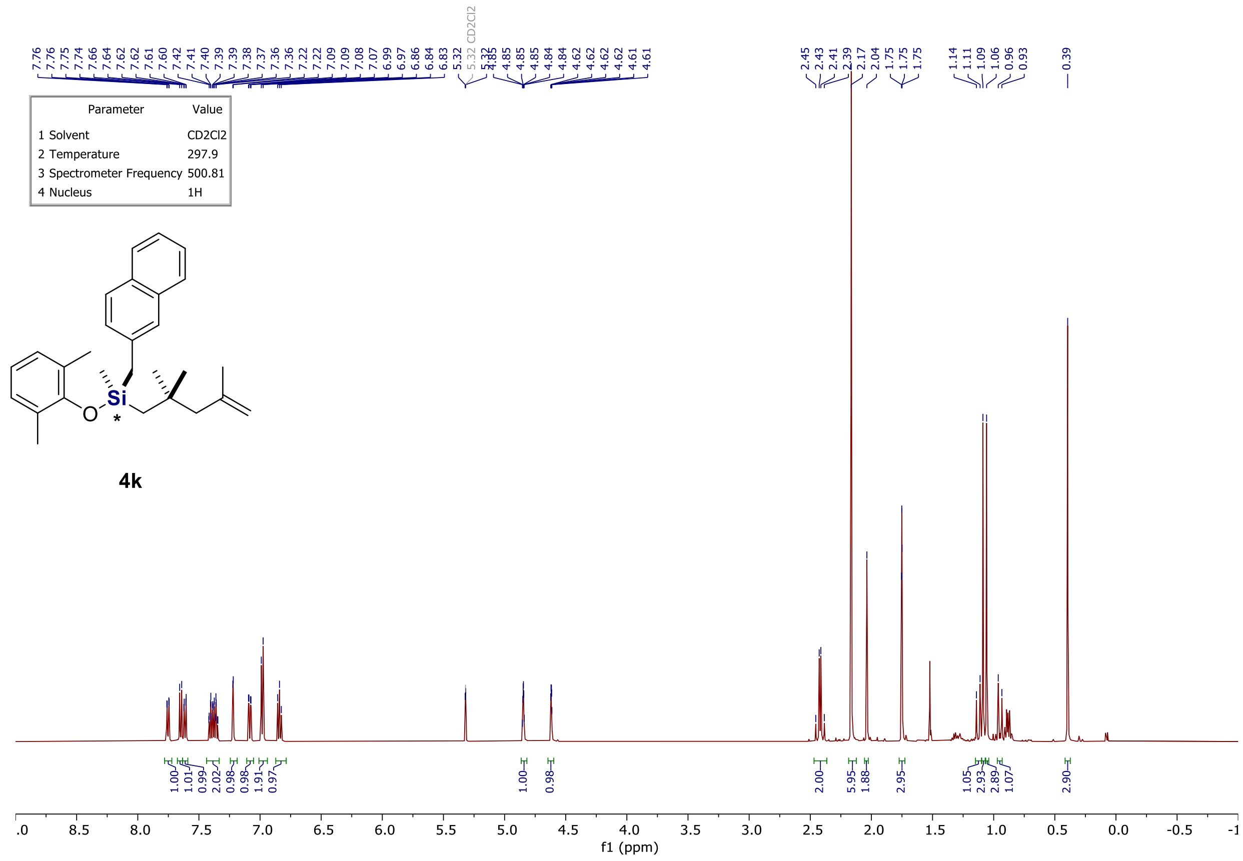

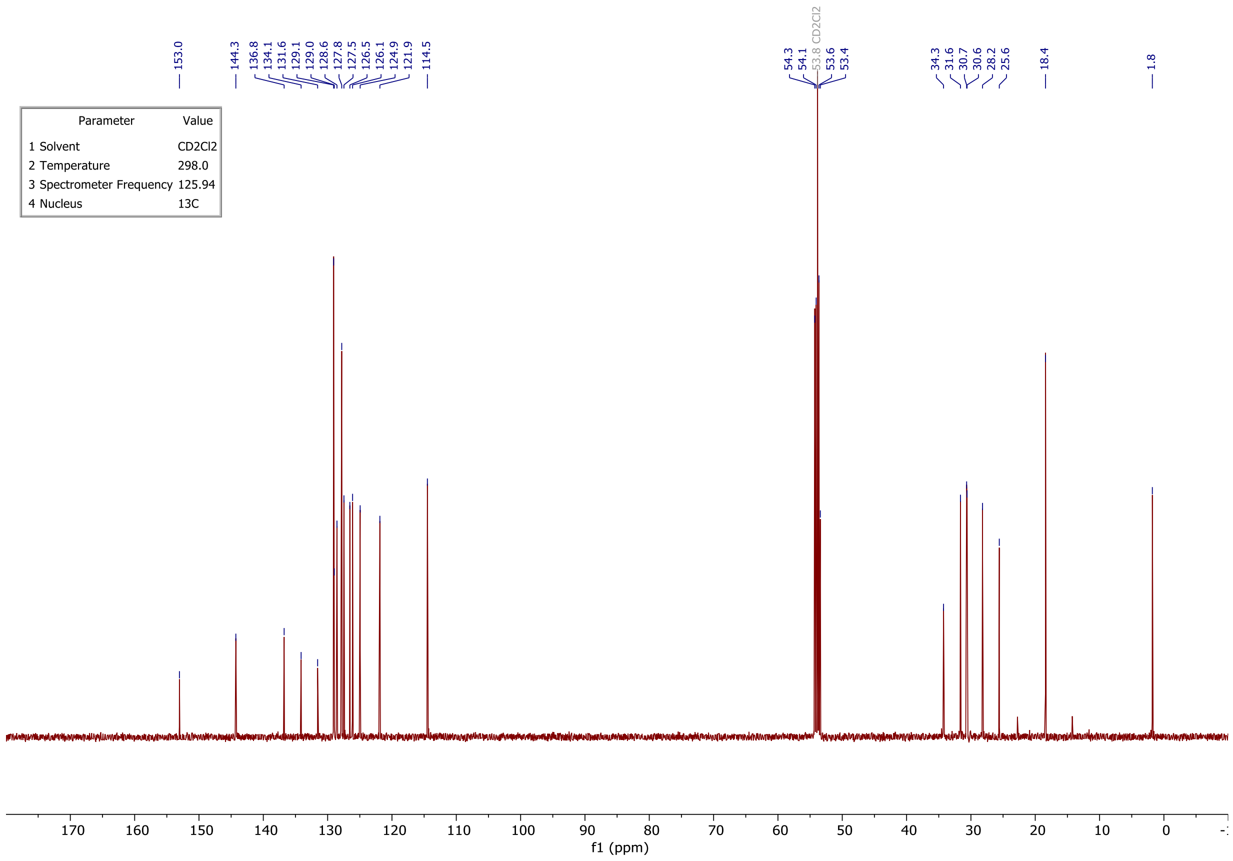

Parameter	Value
1 Solvent	CD2Cl2
2 Temperature	298.0
3 Spectrometer Frequency	125.94
4 Nucleus	13C

(S)-(2,6-dimethylphenoxy)(methyl)(phenyl)(2,2,4-trimethylpent-4-en-1-yl)silane 4l

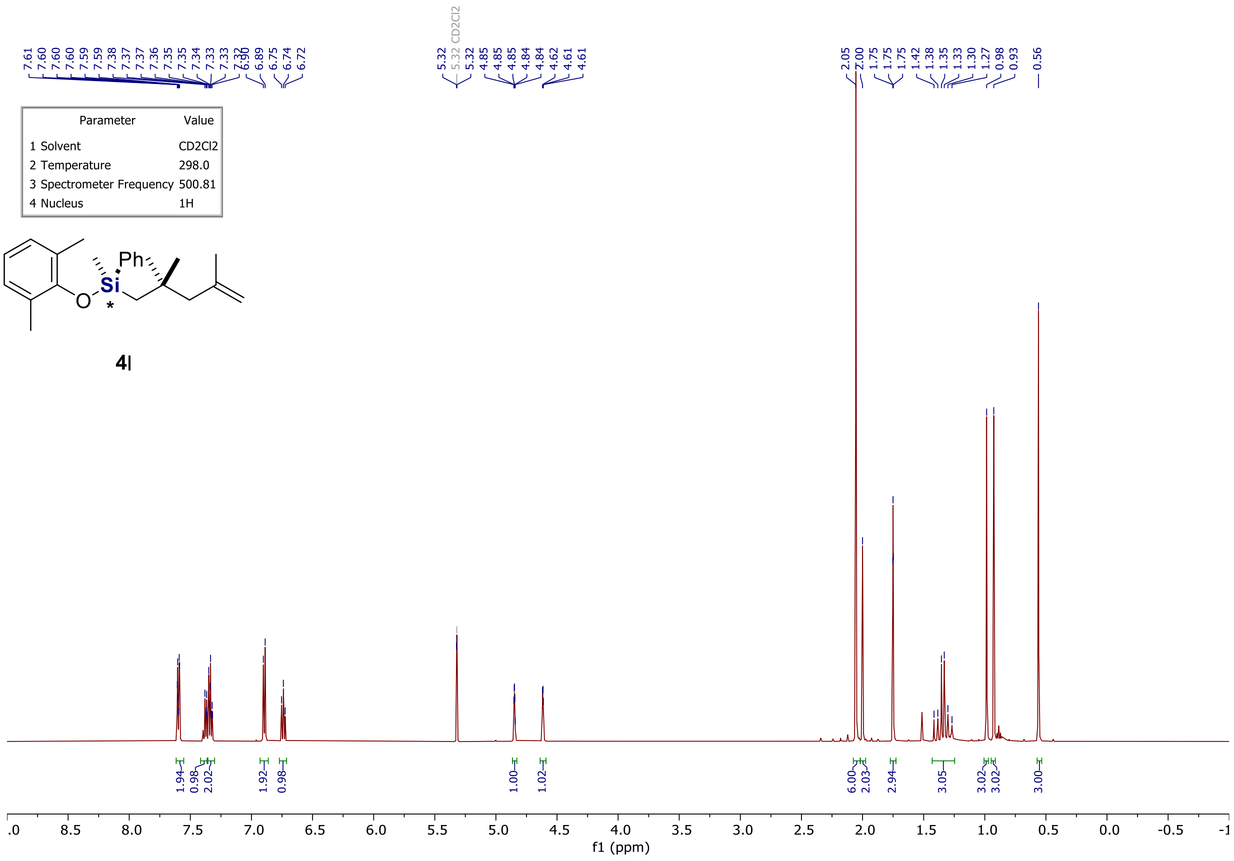

Parameter	Value
1 Solvent	CD2Cl2
2 Temperature	298.0
3 Spectrometer Frequency	500.81
4 Nucleus	1H

4l

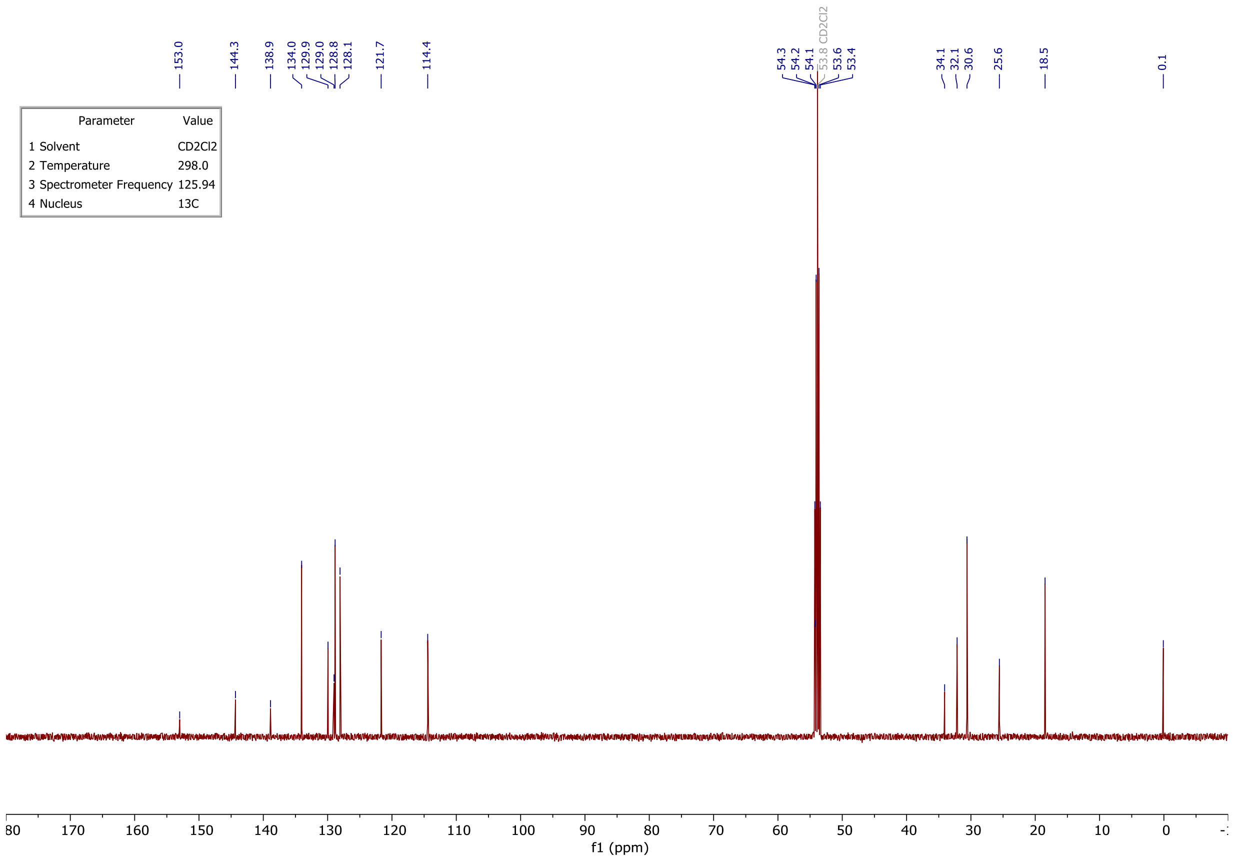

(S)-(2,6-dimethylphenoxy)(methyl)(p-tolyl)(2,2,4-trimethylpent-4-en-1-yl)silane 4m

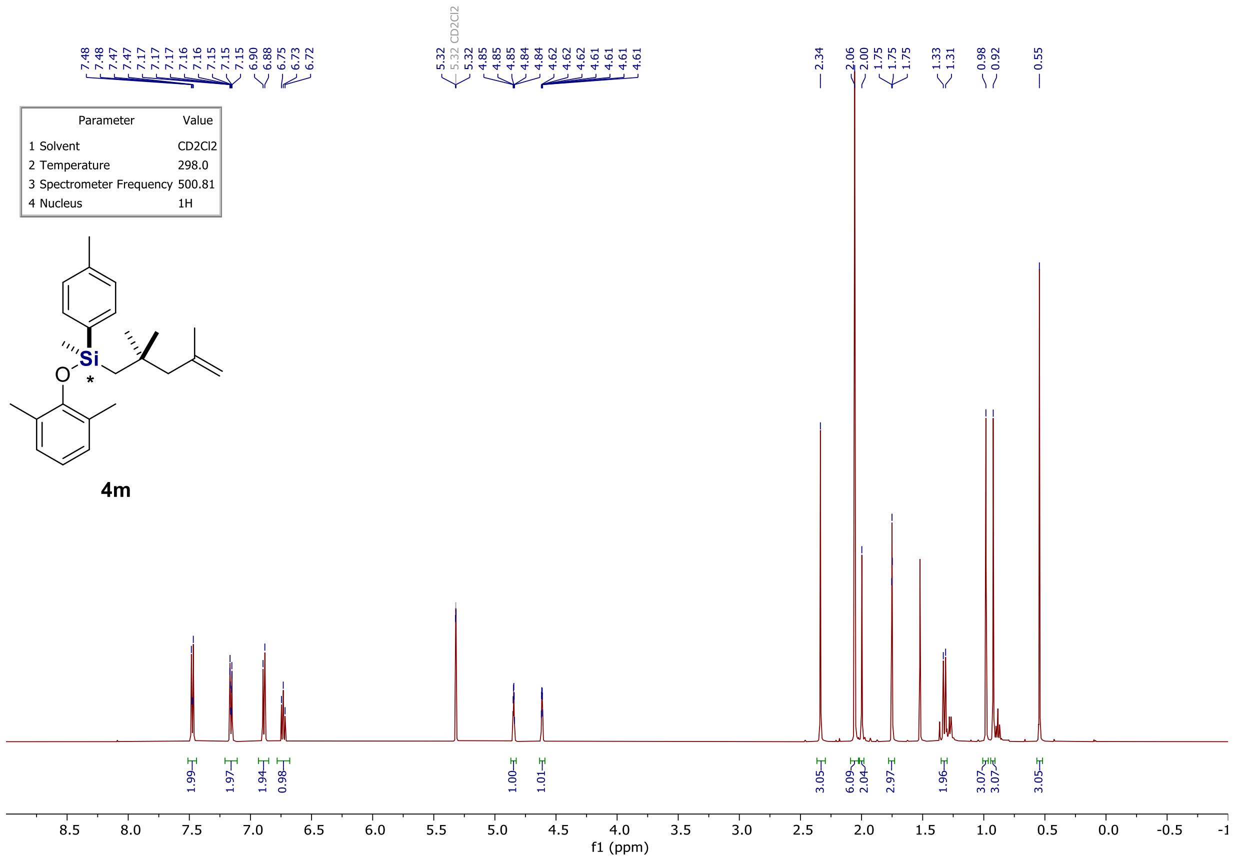

4m

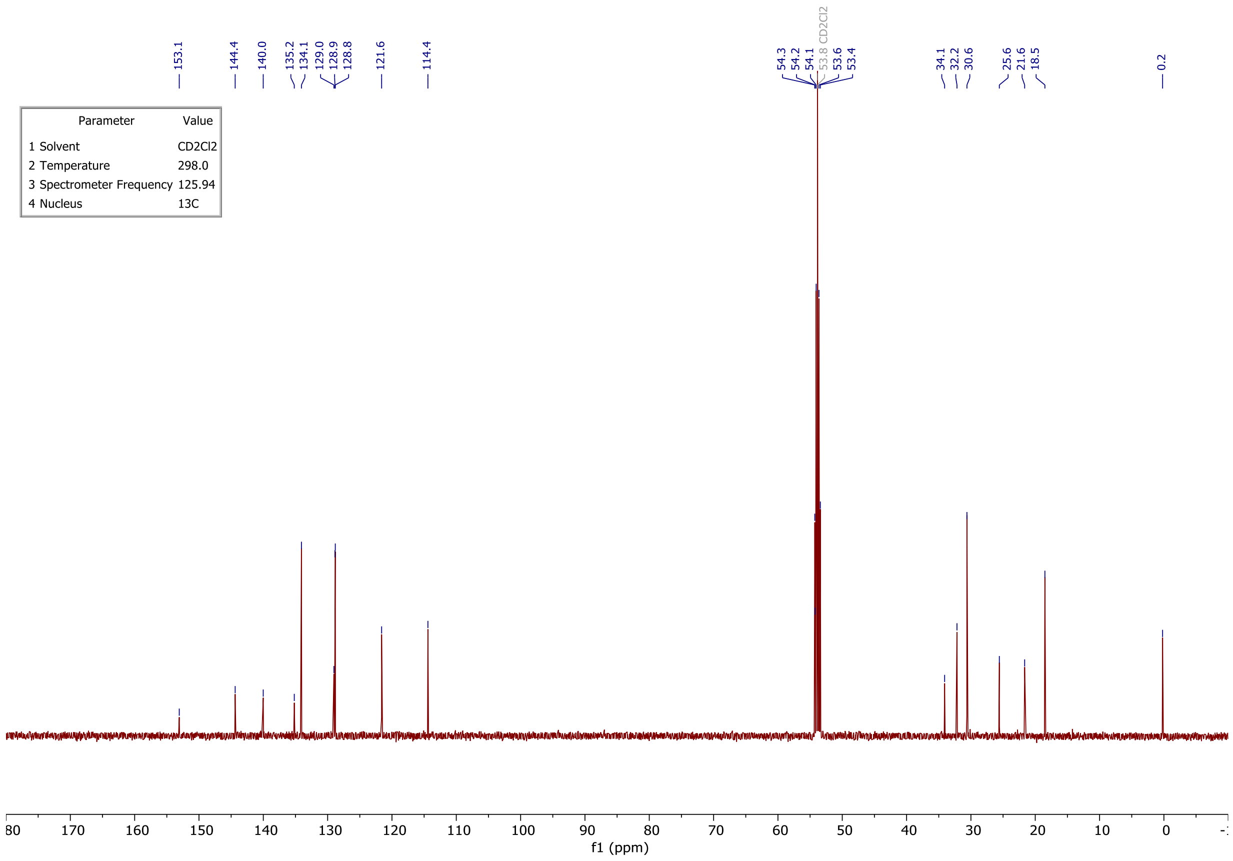

(2,6-dimethylphenoxy)(4-fluorophenyl)(methyl)(2,2,4-trimethylpent-4-en-1-yl)silane 4n

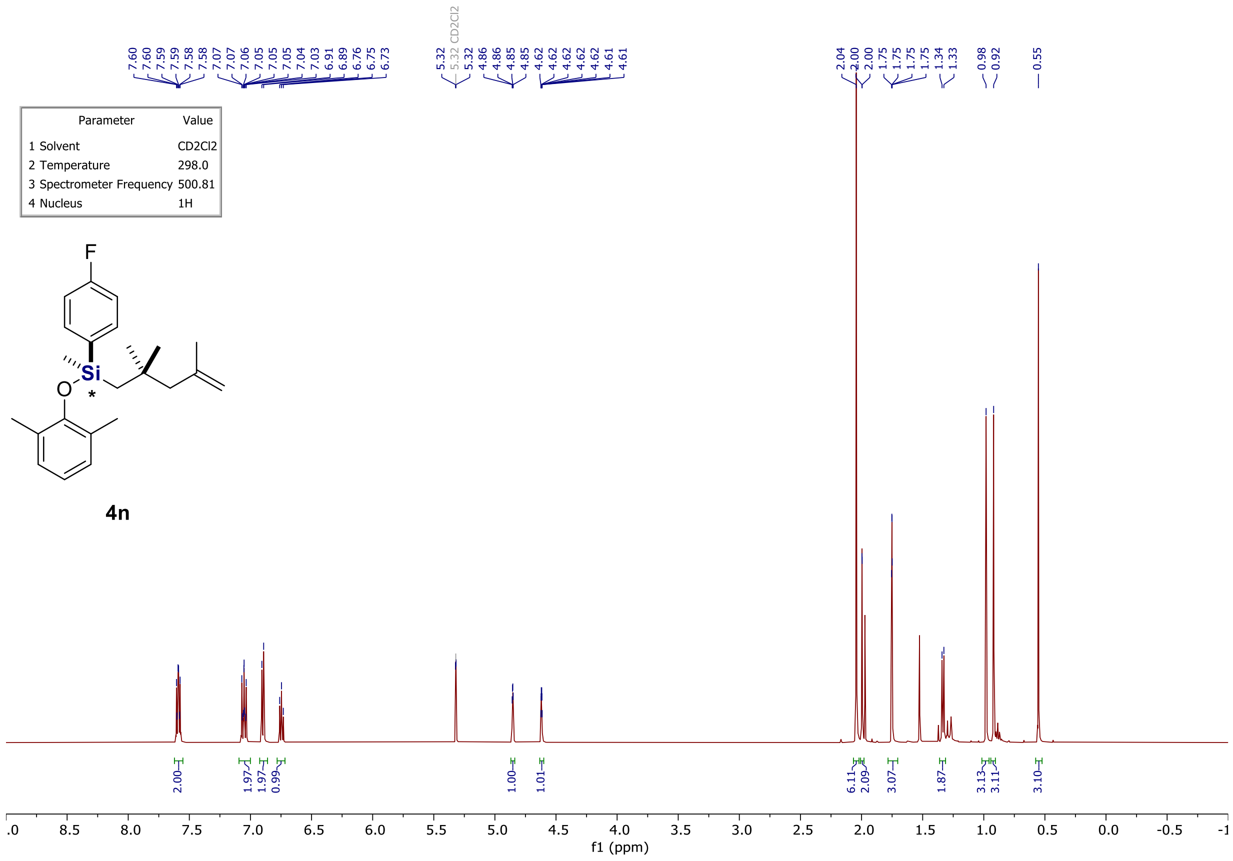

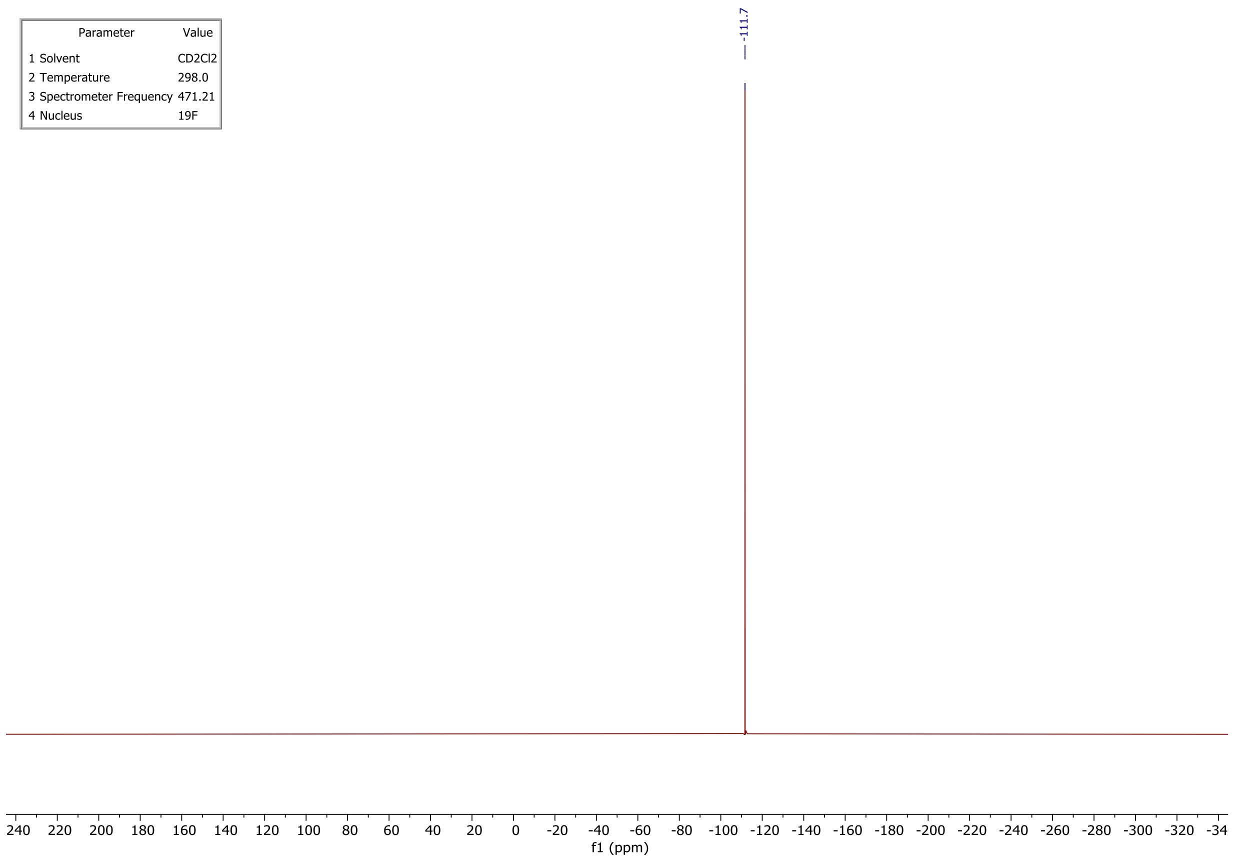

Parameter Value
1 Solvent CD2Cl2
2 Temperature 298.0
3 Spectrometer Frequency 471.21
4 Nucleus 19F
-111.7
240 220 200 180 160 140 120 100 80 60 40 20 0 -20 -40 -60 -80 -100 -120 -140 -160 -180 -200 -220 -240 -260 -280 -300 -320 -34
f1 (ppm)

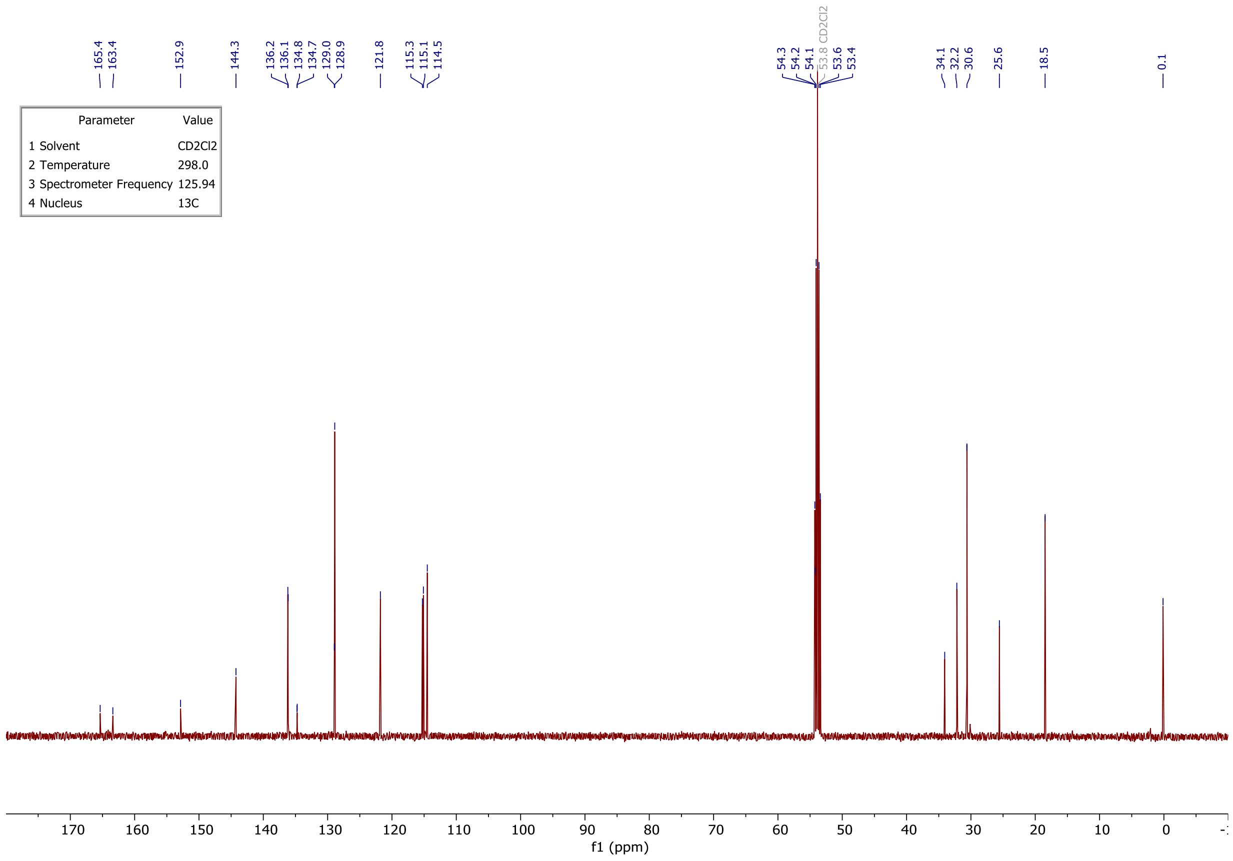

165.4
163.4
152.9
144.3
136.2
136.1
134.8
134.7
129.0
128.9
121.8
115.3
115.1
114.5
54.3
54.2
54.1
53.8 CD2Cl2
53.6
53.4
34.1
32.2
30.6
25.6
18.5
0.1
Parameter Value
1 Solvent CD2Cl2
2 Temperature 298.0
3 Spectrometer Frequency 125.94
4 Nucleus 13C
170 160 150 140 130 120 110 100 90 80 70 60 50 40 30 20 10 0
f1 (ppm)

(2,6-dimethylphenoxy)(methyl)(thiophen-2-yl)(2,2,4-trimethylpent-4-en-1-yl)silane 4o

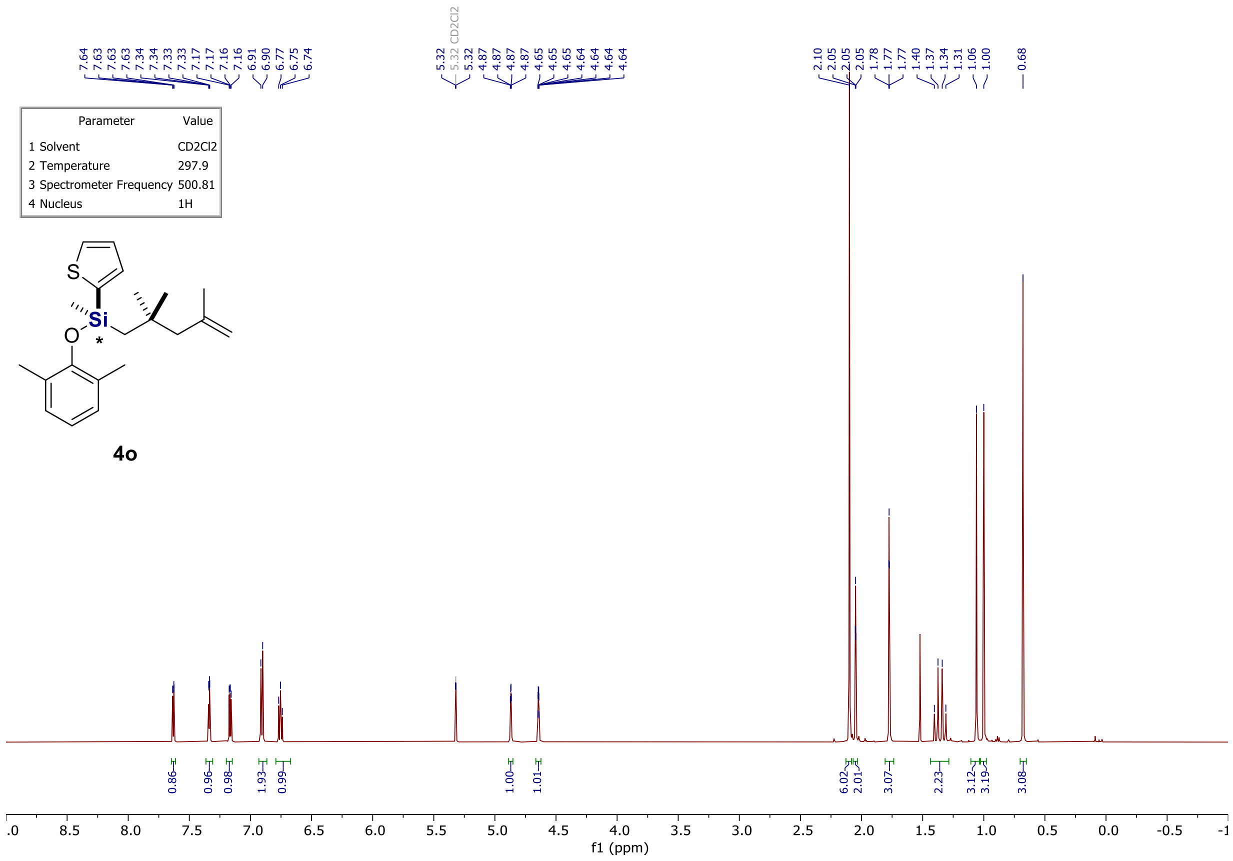

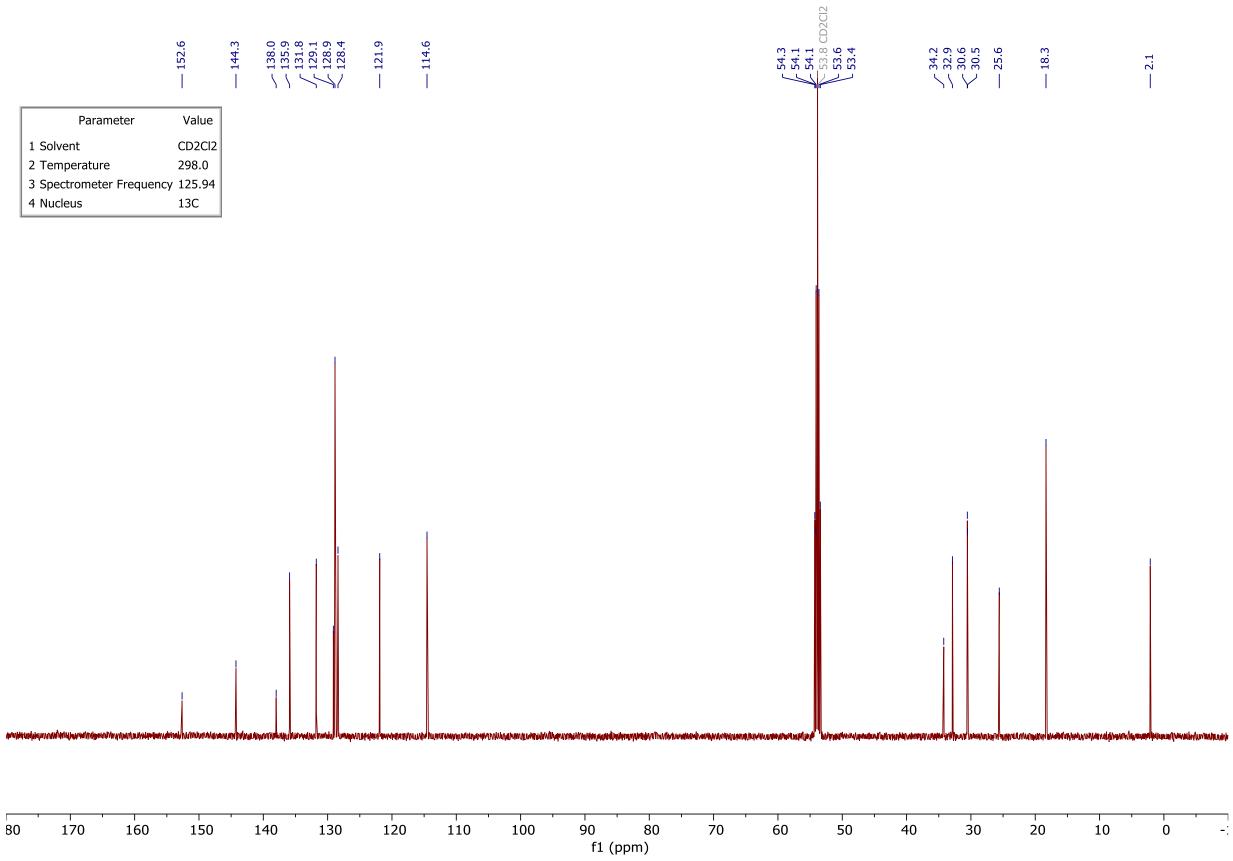

(2,6-dimethylphenoxy)(methyl)(2-methylallyl)(2,2,4-trimethylpent-4-en-1-yl)silane 4p

Parameter	Value
1 Solvent	CD2Cl2
2 Temperature	297.9
3 Spectrometer Frequency	500.81
4 Nucleus	1H

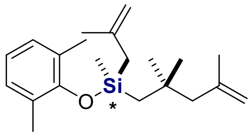

4p

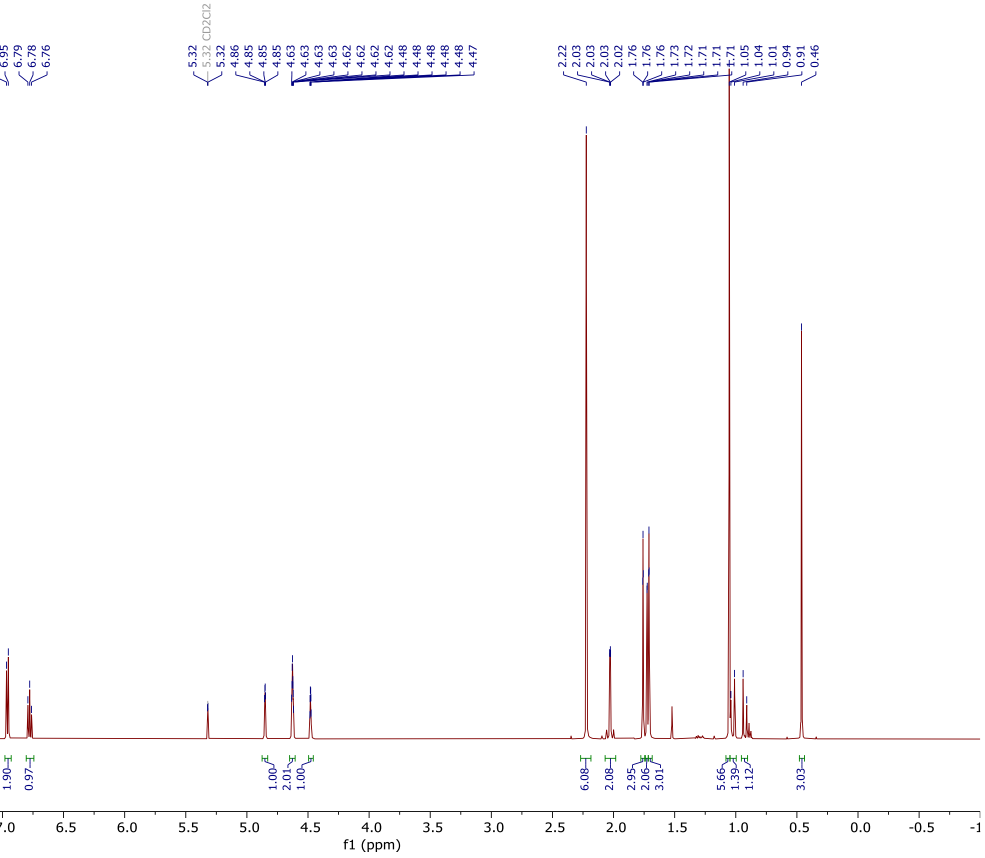

Parameter	Value
1 Solvent	CD2Cl2
2 Temperature	298.0
3 Spectrometer Frequency	125.94
4 Nucleus	13C

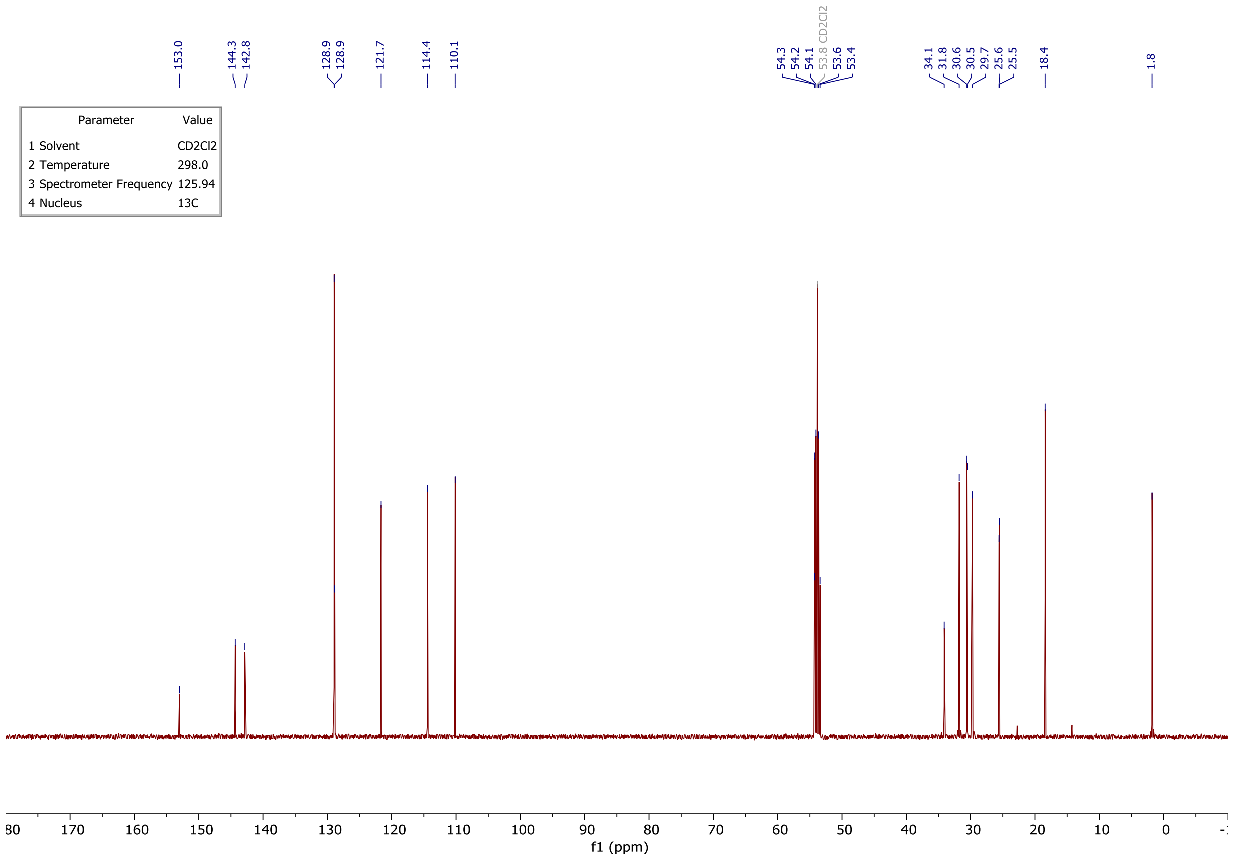

benzyl(2,6-dimethylphenoxy)(ethyl)(2,2,4-trimethylpent-4-en-1-yl)silane 4q

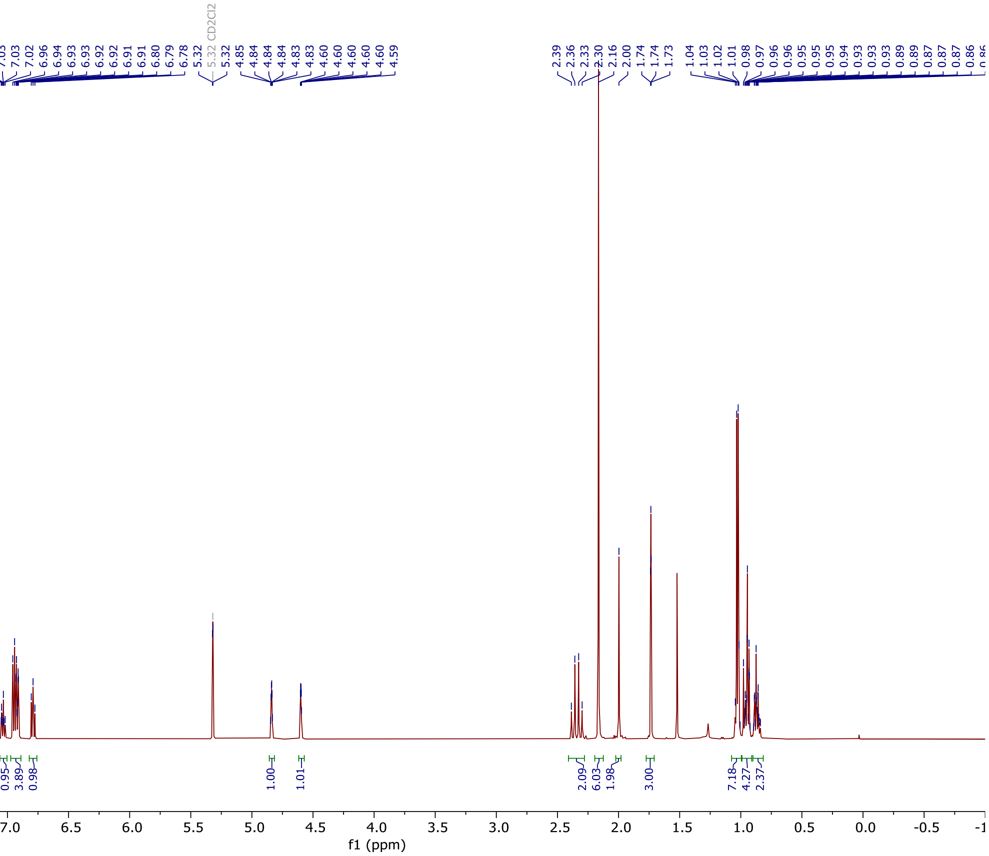

Parameter	Value
1 Solvent	CD2Cl2
2 Temperature	297.9
3 Spectrometer Frequency	500.81
4 Nucleus	1H

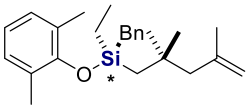

4q

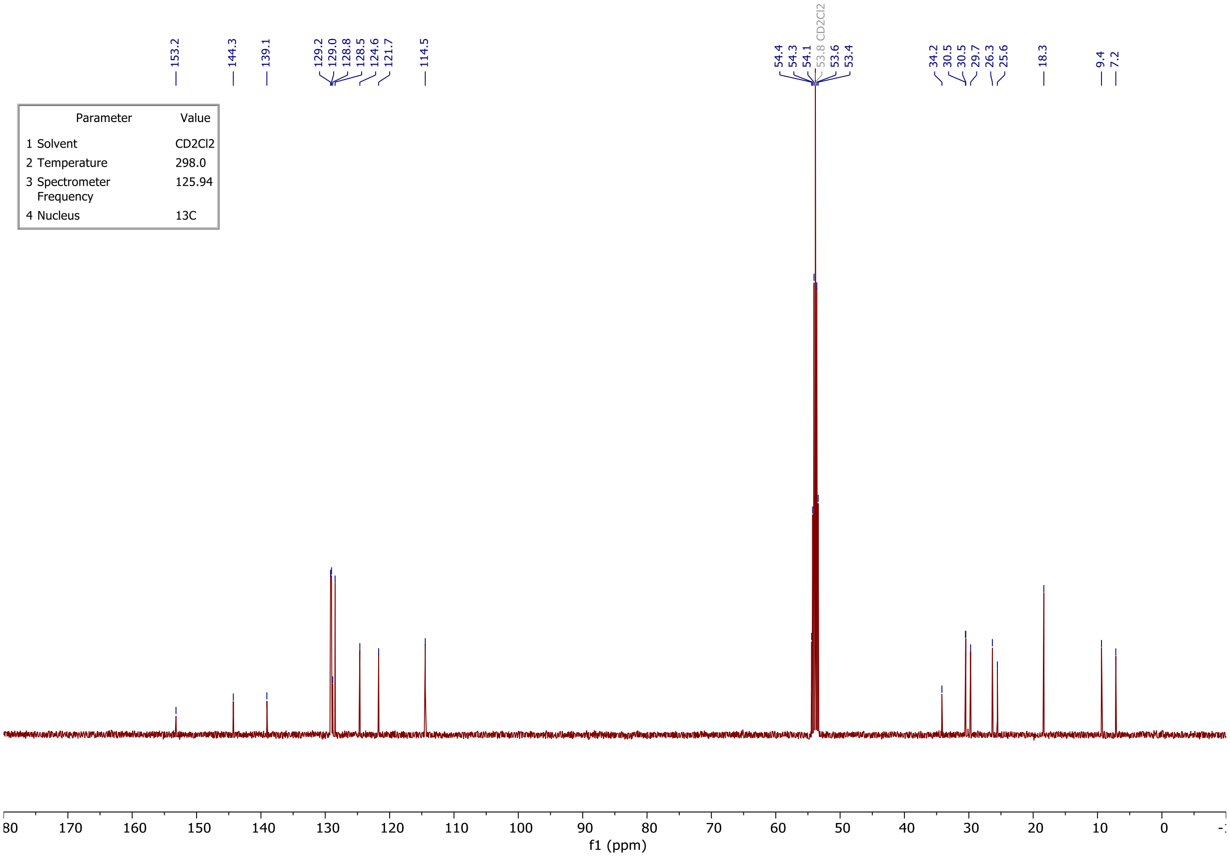

Parameter	Value
1 Solvent	CD2Cl2
2 Temperature	298.0
3 Spectrometer Frequency	125.94
4 Nucleus	13C

(2,6-dimethoxyphenoxy)(ethyl)(methyl)(2,2,4-trimethylpent-4-en-1-yl)silane

Parameter	Value
1 Solvent	CD2Cl2
2 Temperature	297.9
3 Spectrometer Frequency	500.81
4 Nucleus	1H

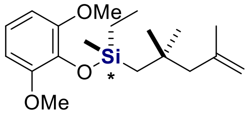

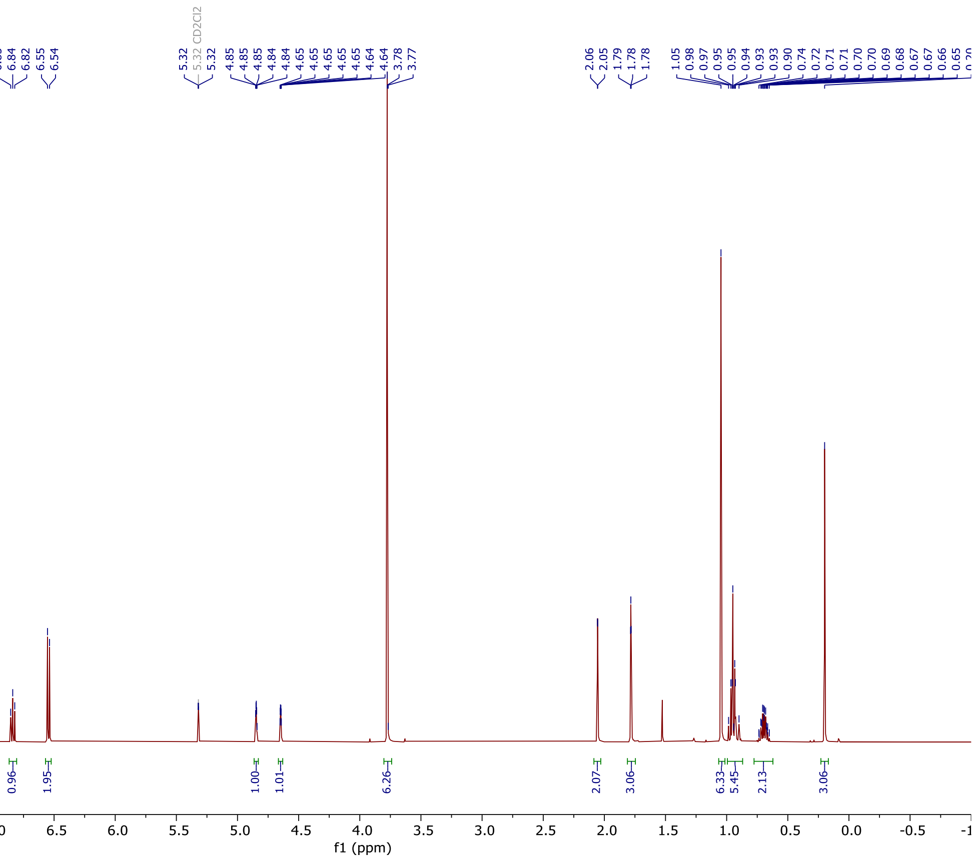

Parameter	Value
1 Solvent	CD2Cl2
2 Temperature	298.0
3 Spectrometer Frequency	125.94
4 Nucleus	13C

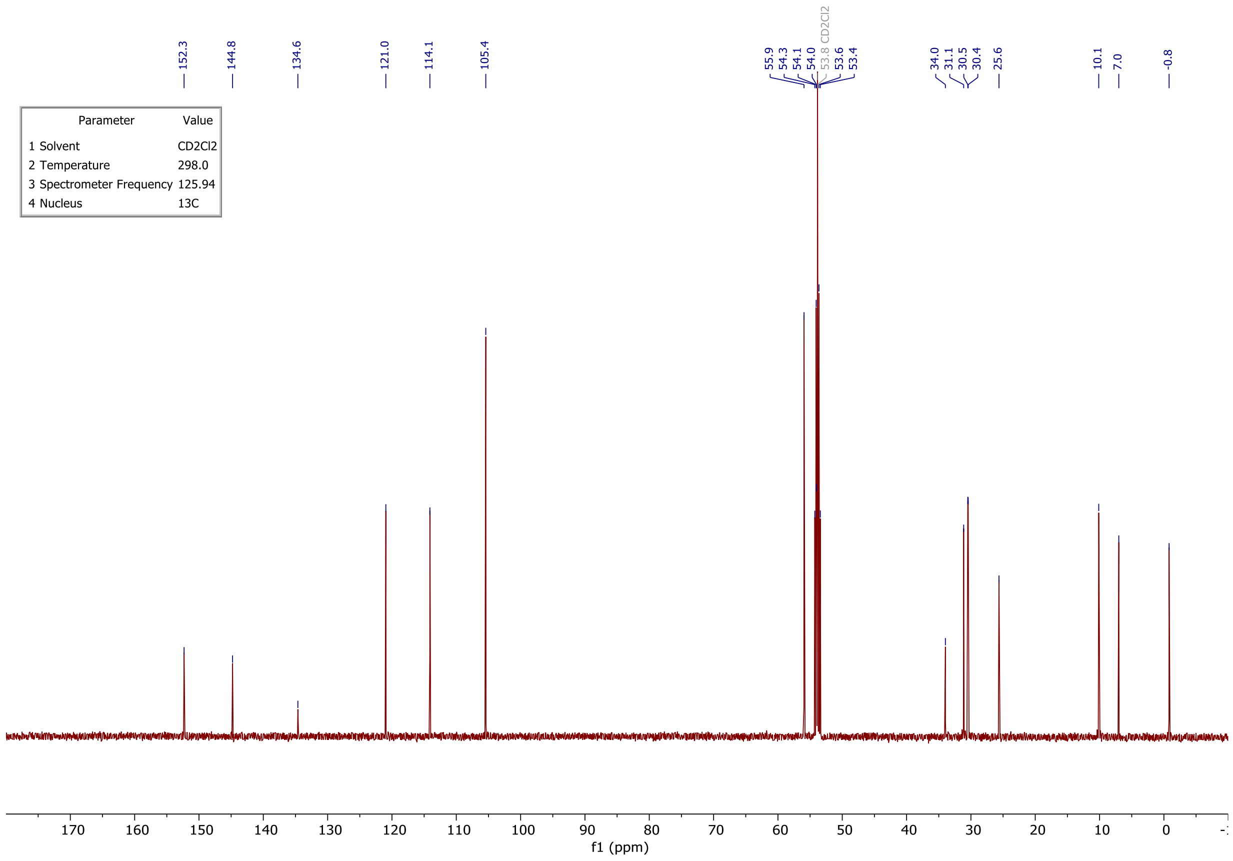

butyl(2,6-dimethoxyphenoxy)(methyl)(2,2,4-trimethylpent-4-en-1-yl)silane

Parameter	Value
1 Solvent	CD2Cl2
2 Temperature	297.9
3 Spectrometer Frequency	500.81
4 Nucleus	1H

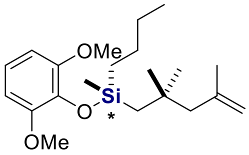

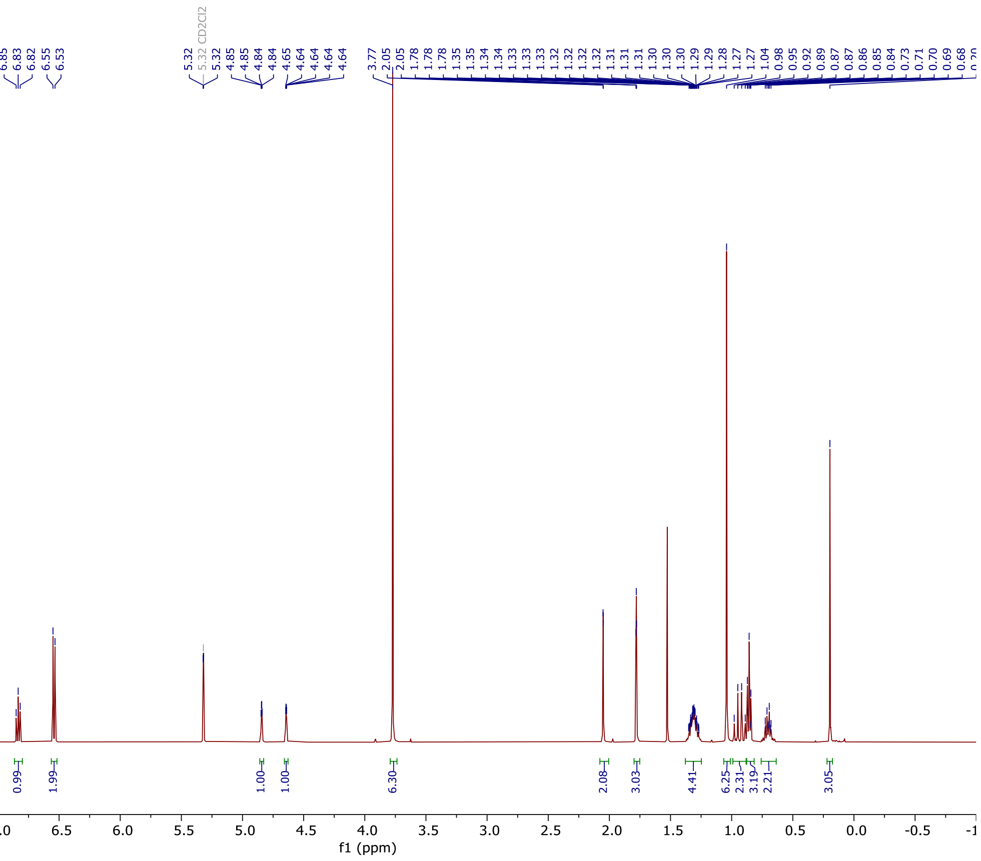

Parameter	Value
1 Solvent	CD2Cl2
2 Temperature	298.0
3 Spectrometer Frequency	125.94
4 Nucleus	13C

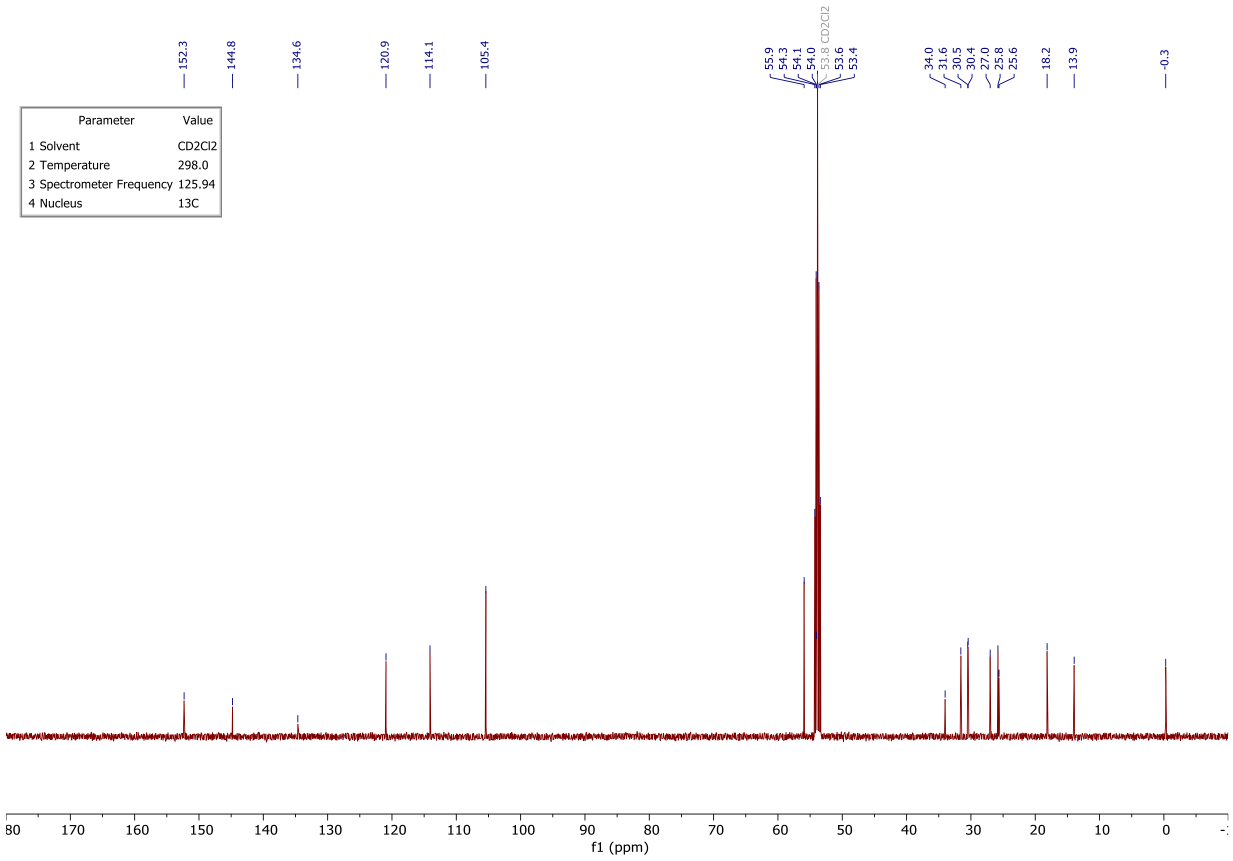

(2,6-dimethoxyphenoxy)(methyl)(pentyl)(2,2,4-trimethylpent-4-en-1-yl)silane

Parameter	Value
1 Solvent	CD2Cl2
2 Temperature	298.0
3 Spectrometer Frequency	500.81
4 Nucleus	1H

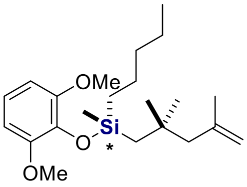

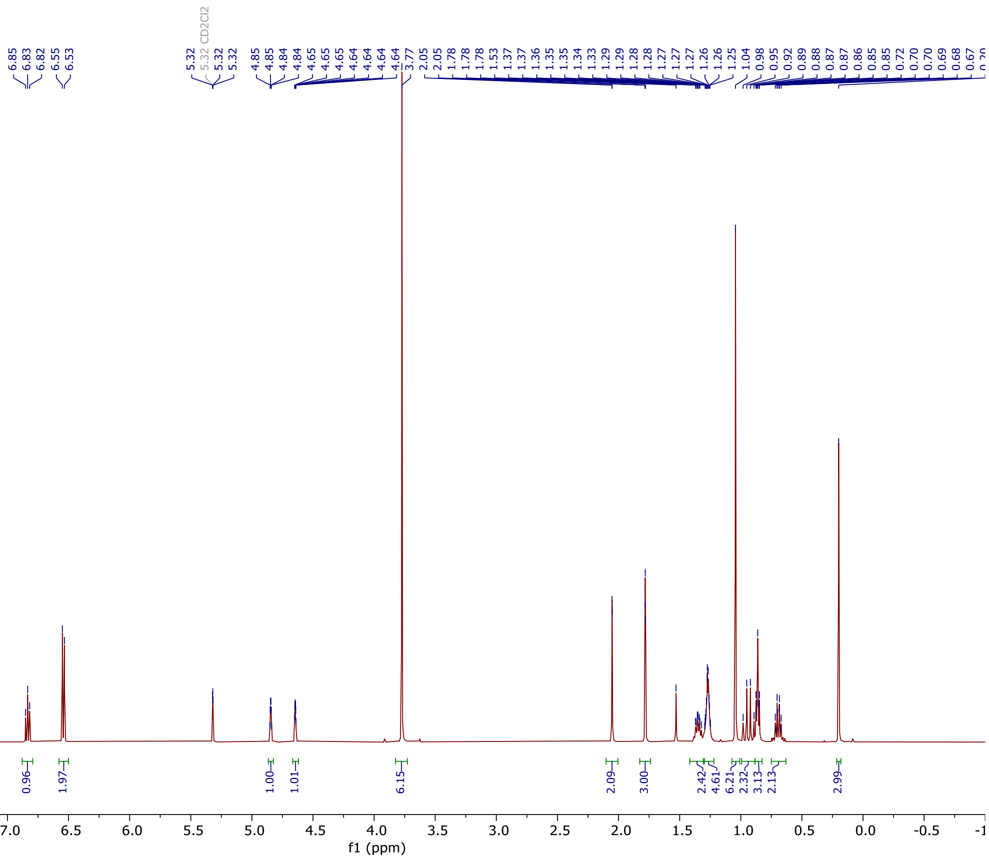

Parameter	Value
1 Solvent	CD2Cl2
2 Temperature	298.0
3 Spectrometer Frequency	125.94
4 Nucleus	13C

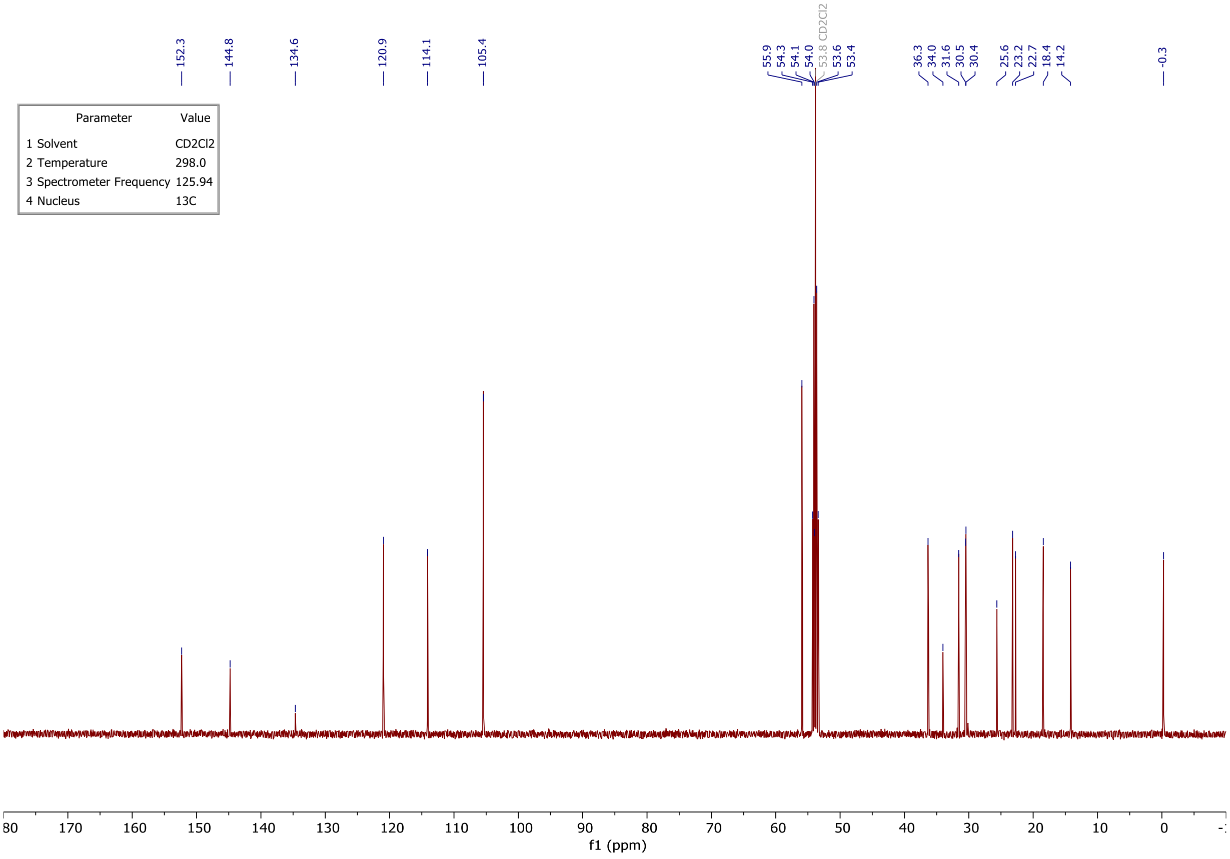

(S)-(2,6-dimethoxyphenoxy)(hexyl)(methyl)(2,2,4-trimethylpent-4-en-1-yl)silane

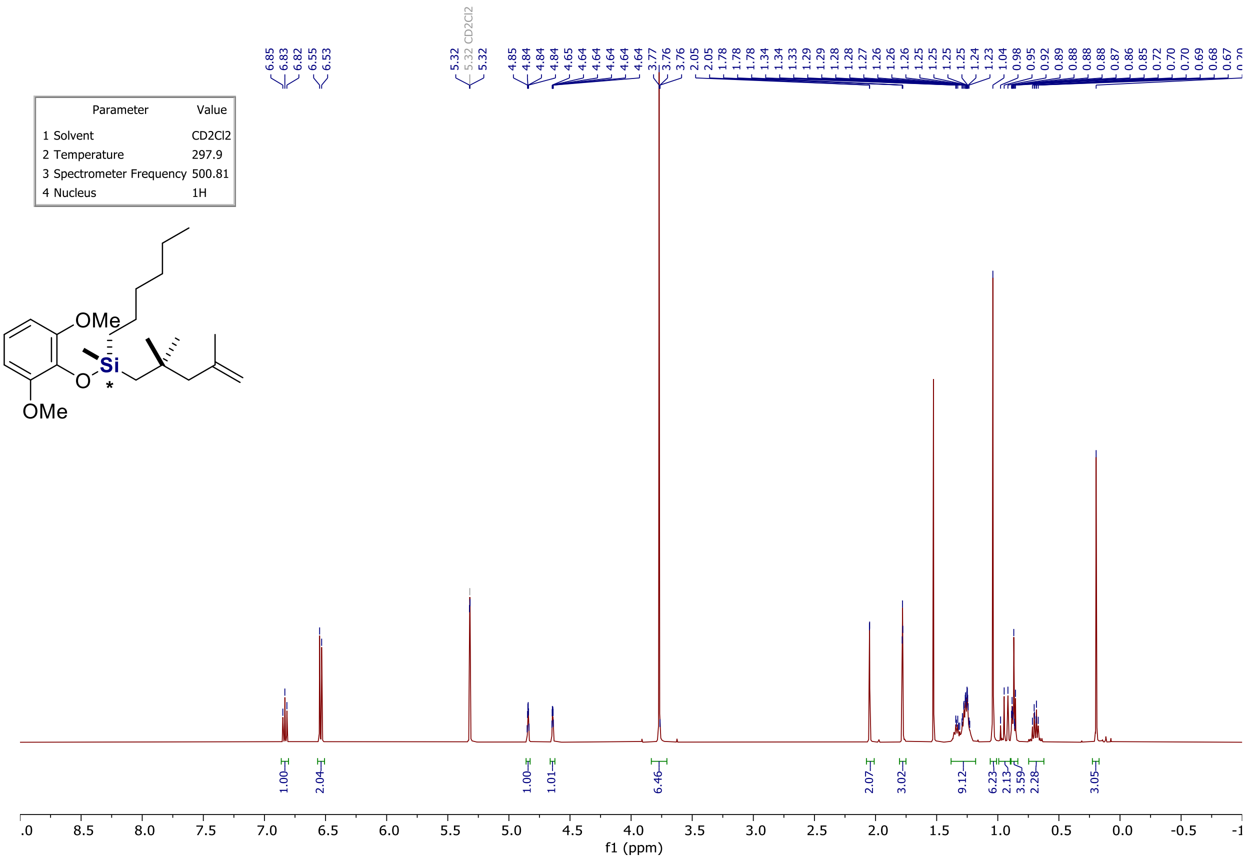

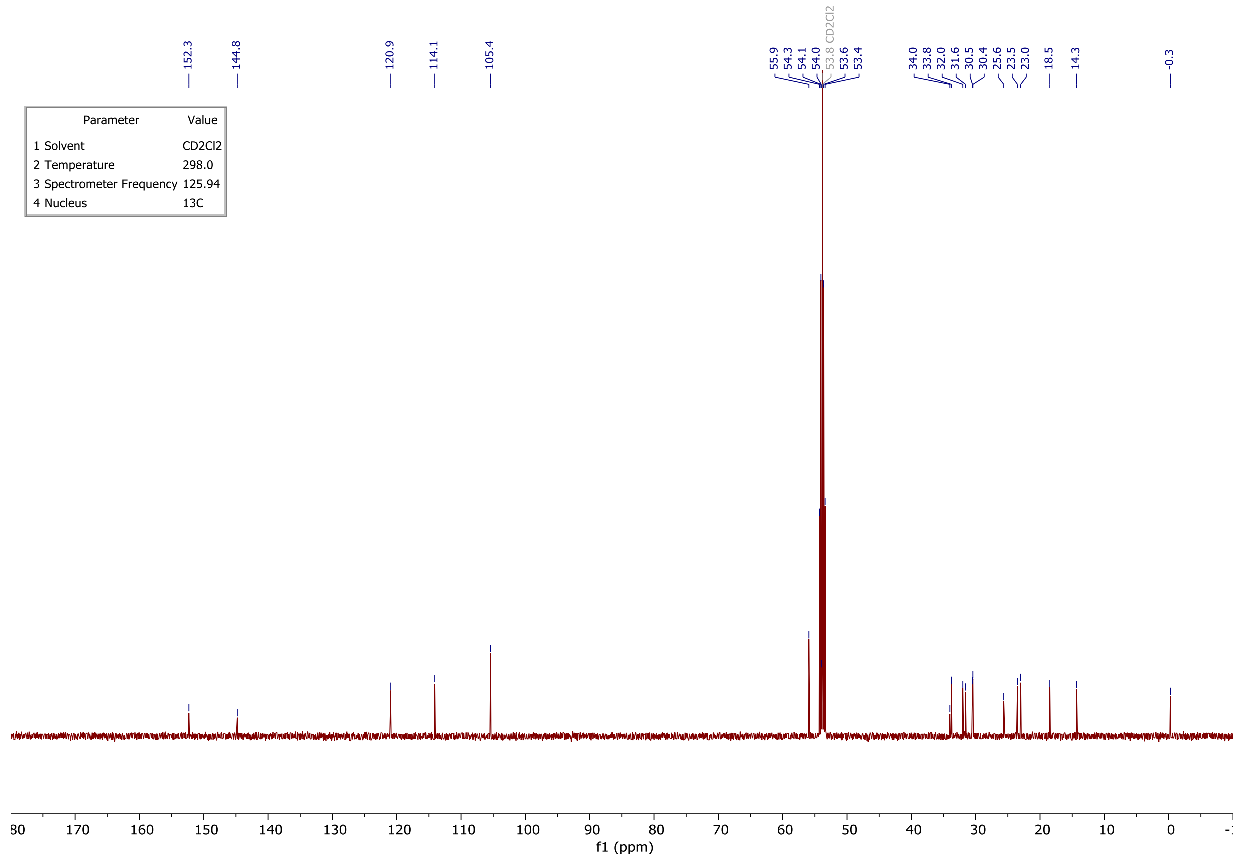

benzyl(2,6-dimethylphenoxy)(methyl)silyl)-2,4,4-trimethylpentan-1-ol 6

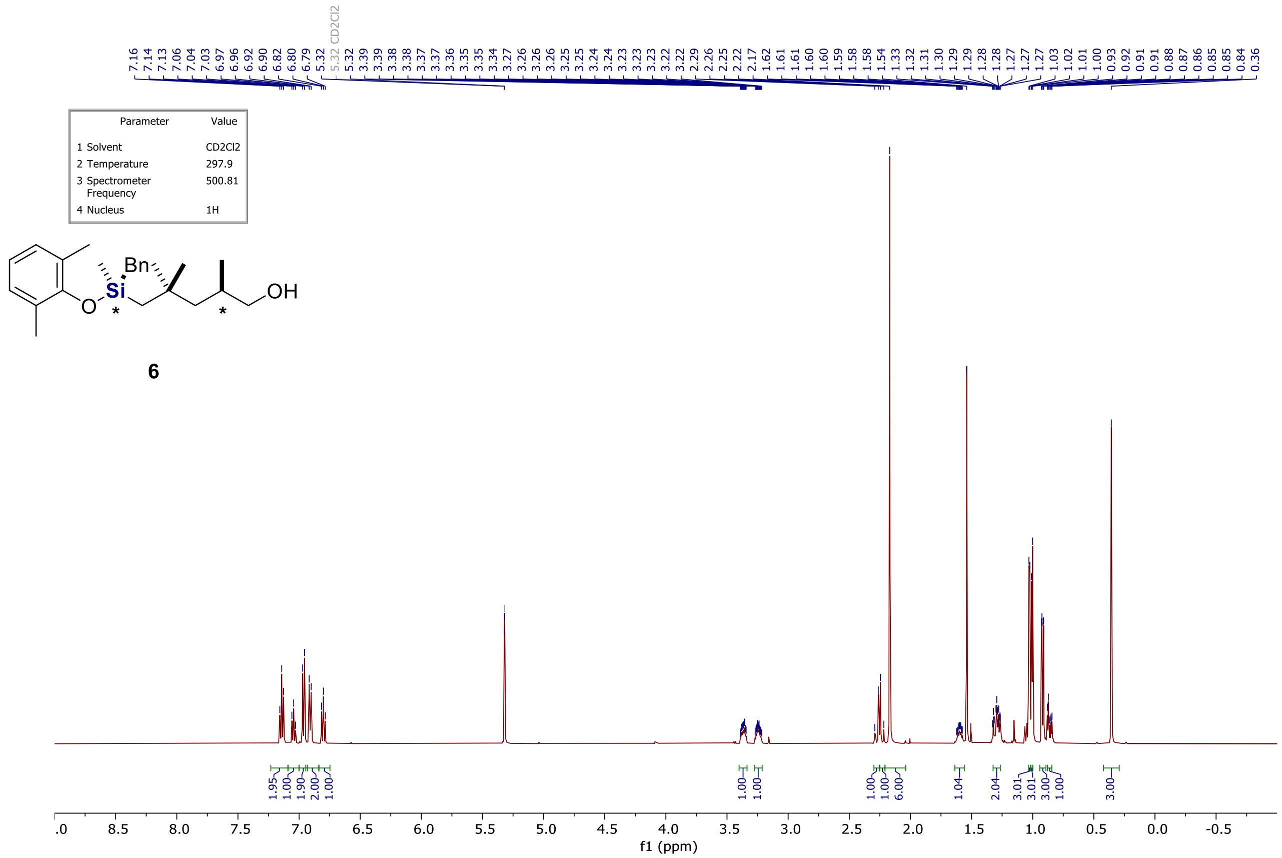

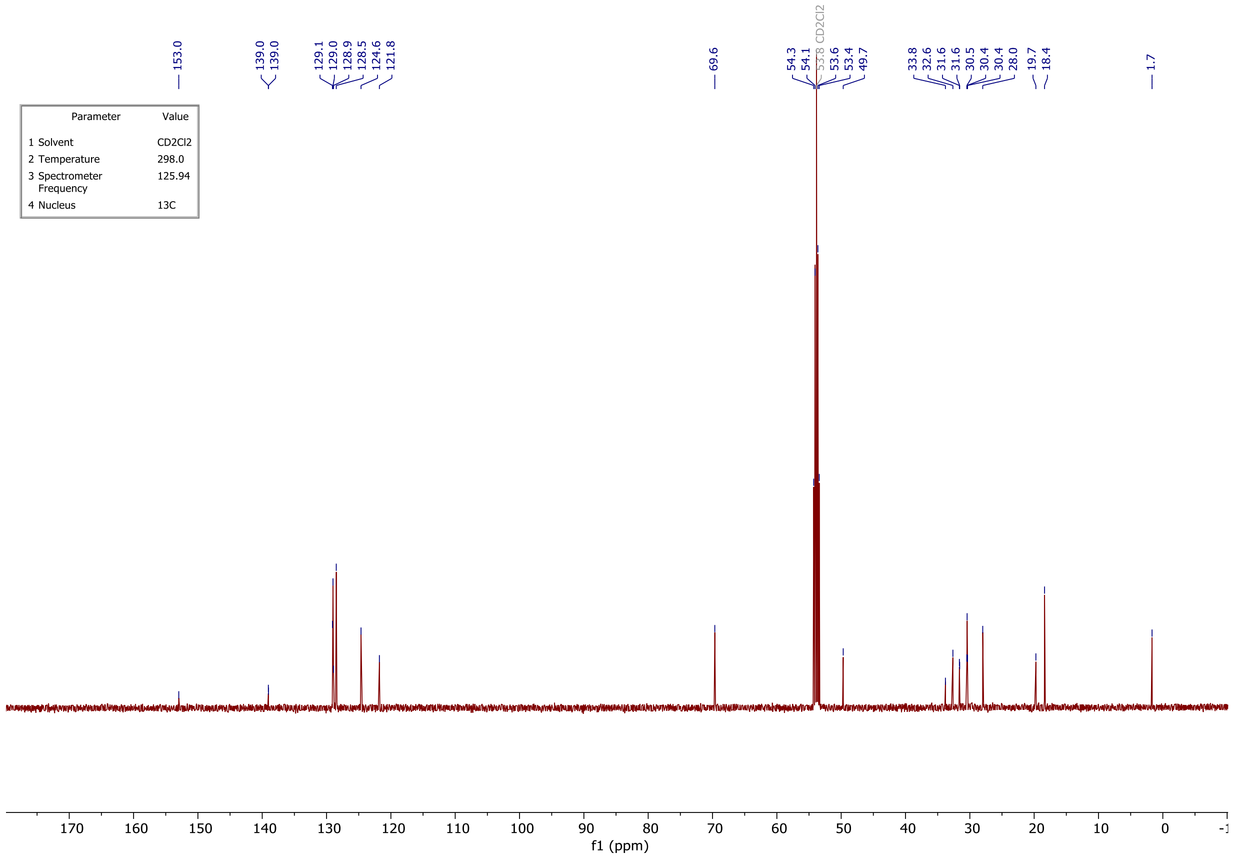

benzyl(2,2-dimethyl-3-(1-methylcyclopropyl)propyl)(2,6-dimethylphenoxy)(methyl)silane 7

Parameter	Value
1 Solvent	CD2Cl2
2 Temperature	298.0
3 Spectrometer Frequency	500.81
4 Nucleus	1H

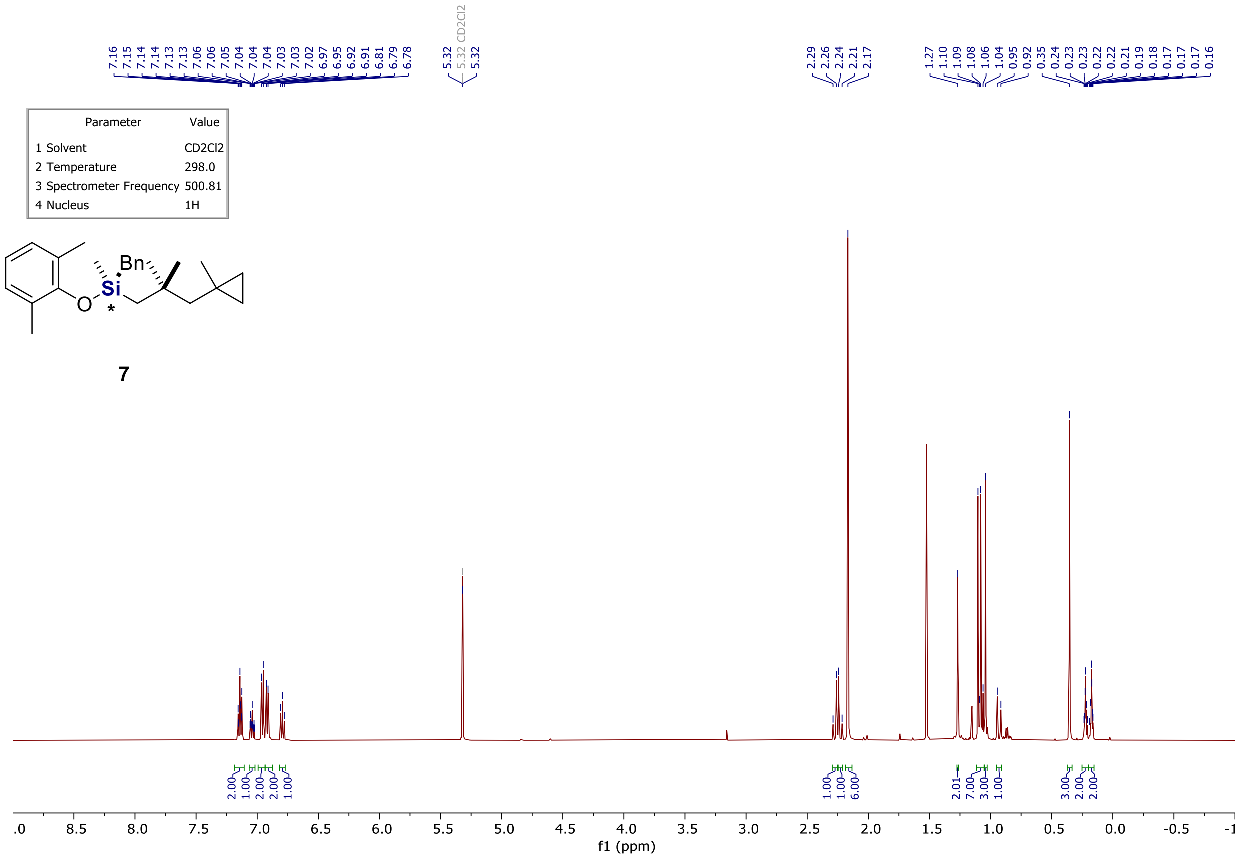

Parameter	Value
1 Solvent	CD2Cl2
2 Temperature	298.0
3 Spectrometer Frequency	125.94
4 Nucleus	13C

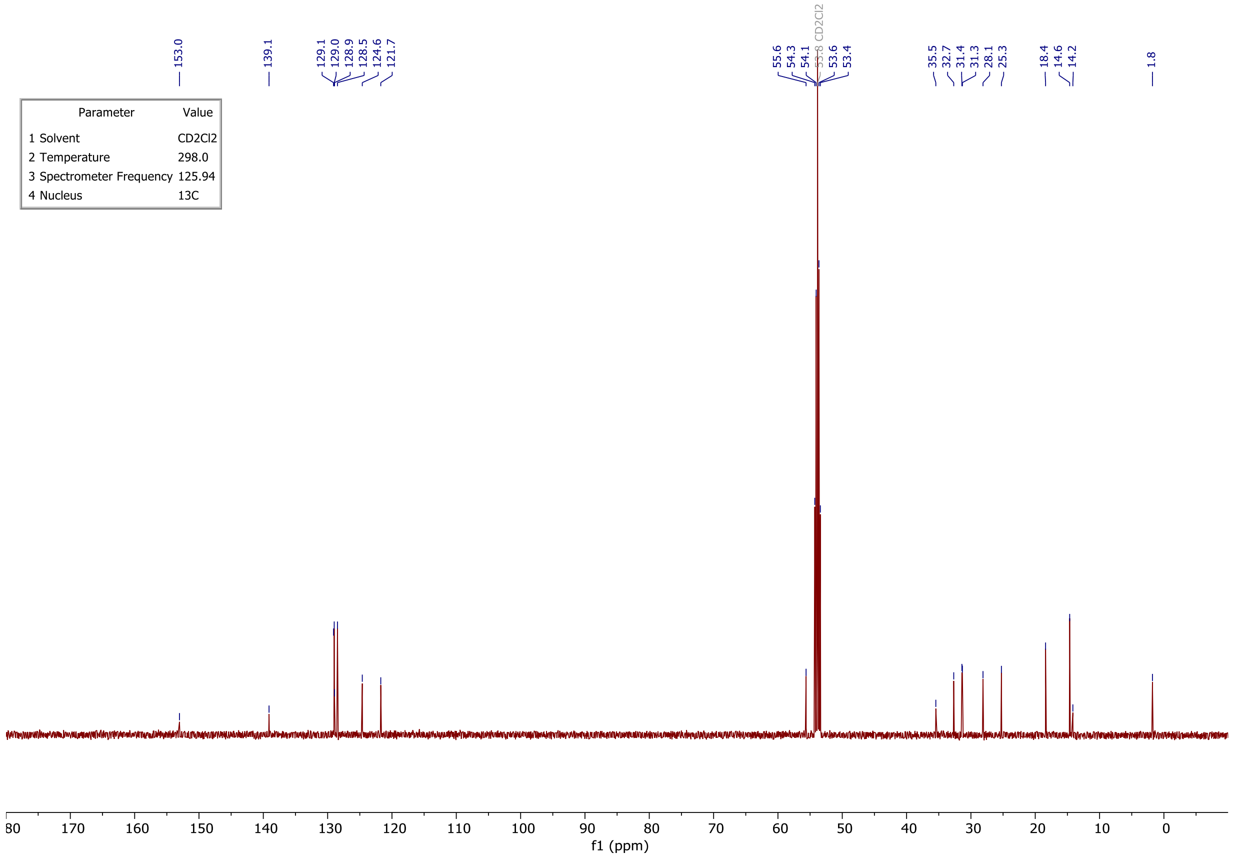

benzyl(2,2-dimethyl-3-(2-methyloxiran-2-yl)propyl)(2,6-dimethylphenoxy)(methyl)silane 8

Parameter	Value
1 Solvent	CD2Cl2
2 Temperature	298.0
3 Spectrometer Frequency	500.81
4 Nucleus	1H

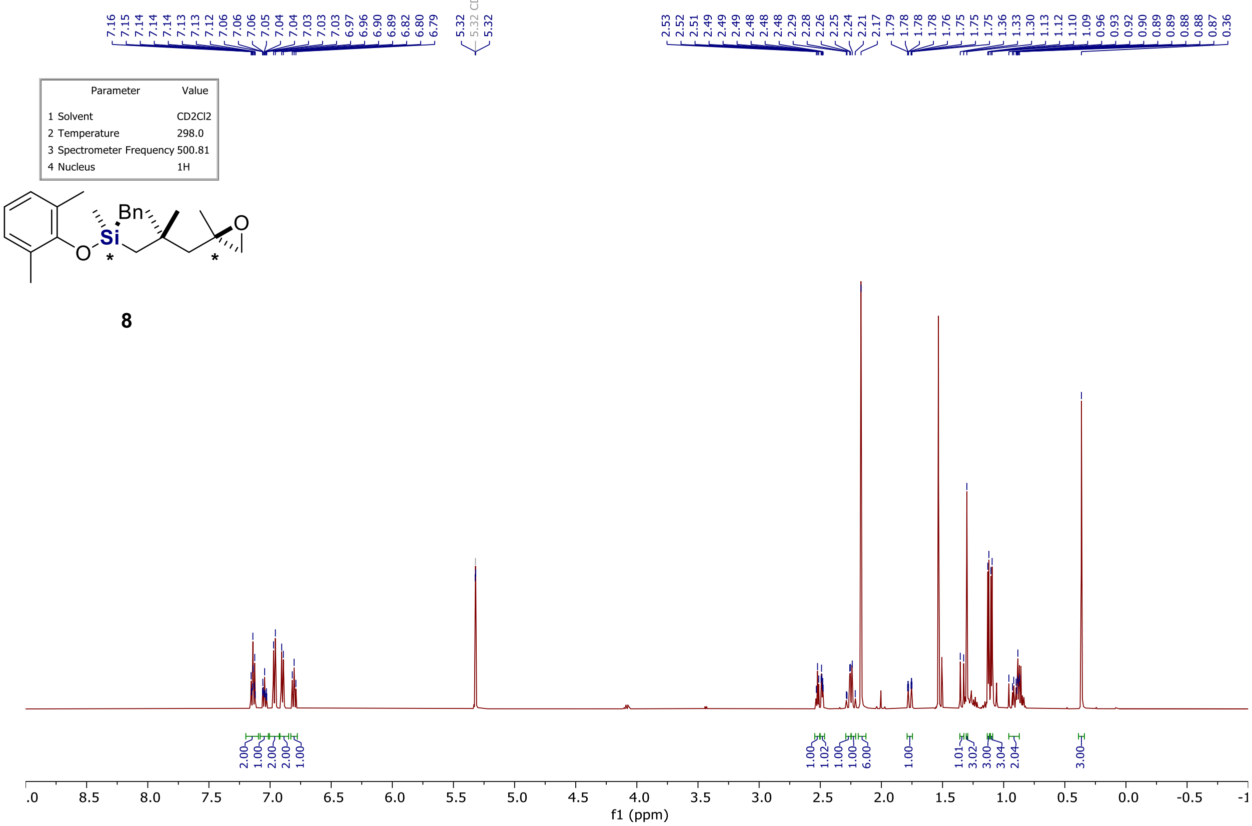

Parameter	Value
1 Solvent	CD2Cl2
2 Temperature	298.0
3 Spectrometer Frequency	125.94
4 Nucleus	13C

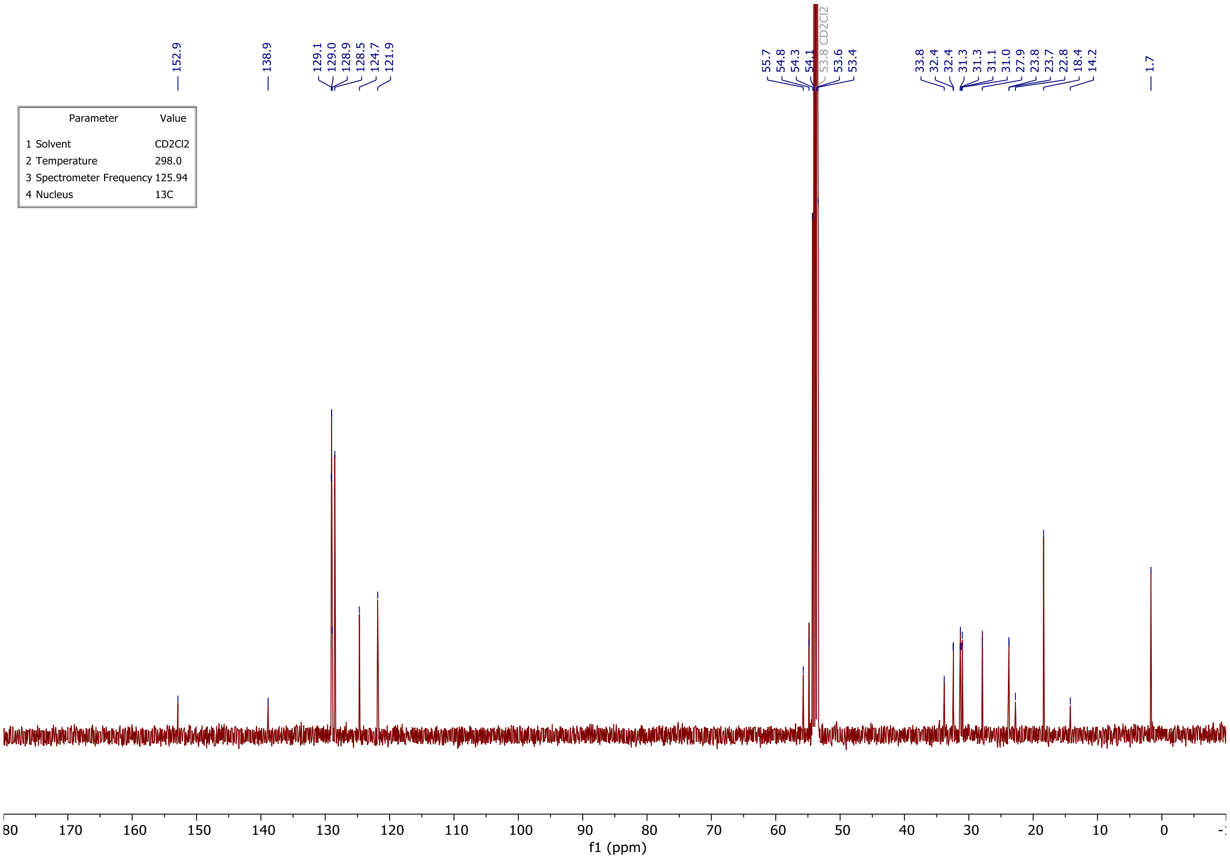

benzyl(2,6-dimethylphenoxy)(methyl)(2,2,4-trimethylpentyl)silane 9

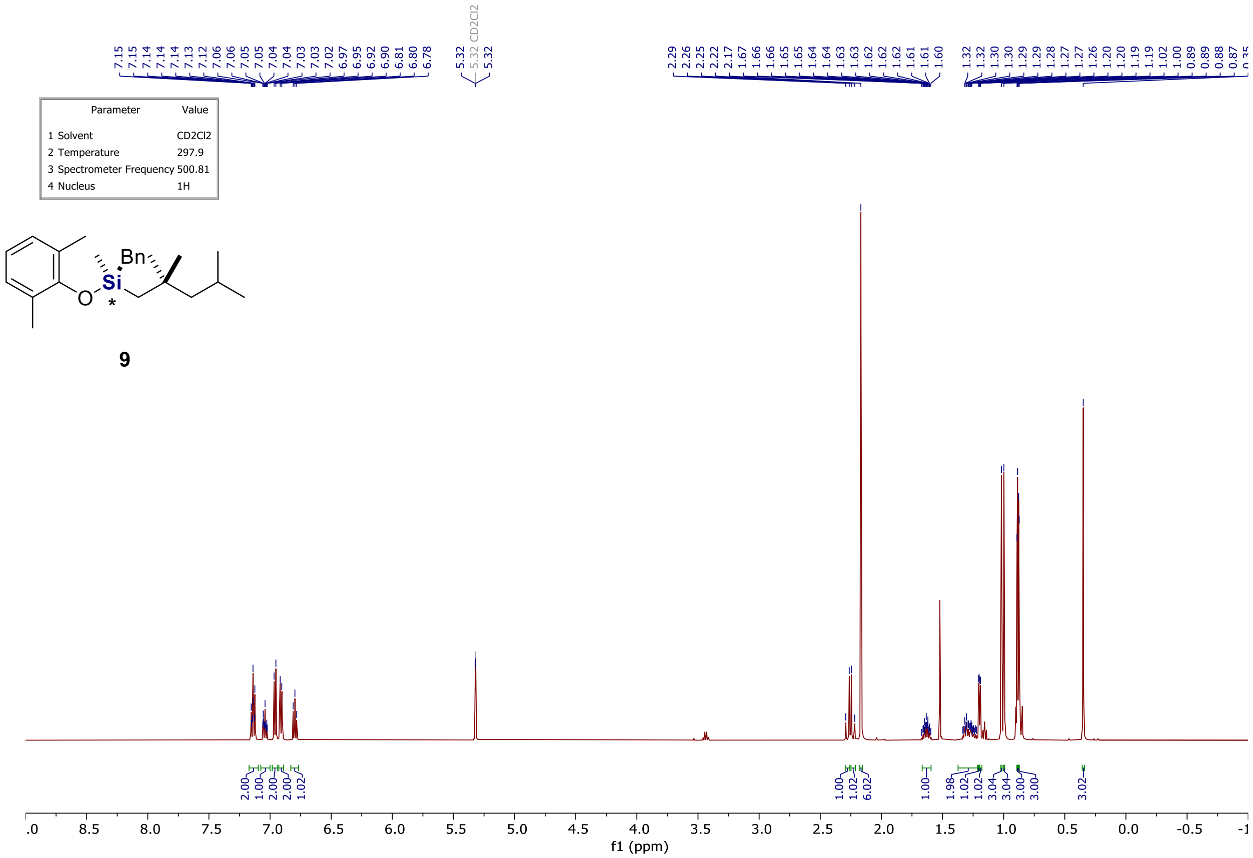

Parameter	Value
1 Solvent	CD2Cl2
2 Temperature	297.9
3 Spectrometer Frequency	500.81
4 Nucleus	1H

9

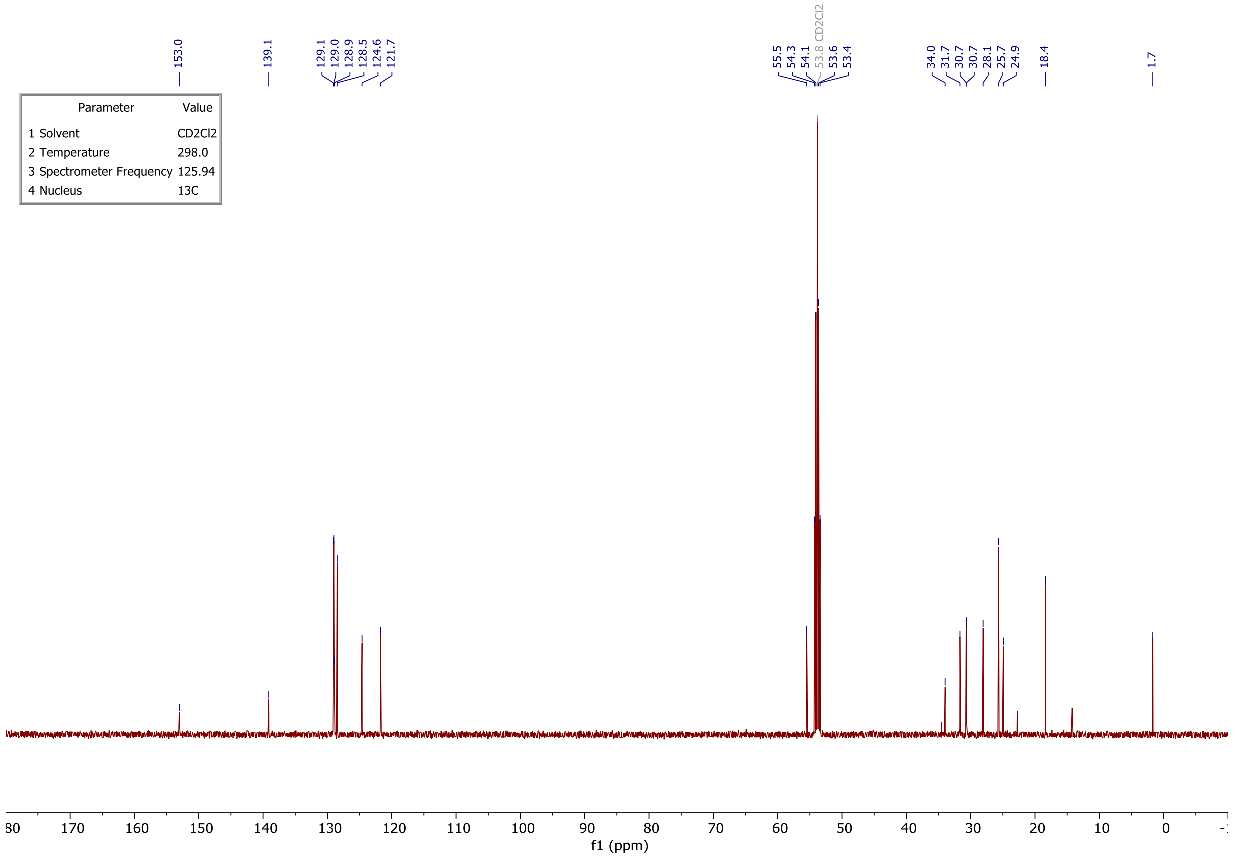

Parameter	Value
1 Solvent	CD2Cl2
2 Temperature	298.0
3 Spectrometer Frequency	125.94
4 Nucleus	13C

benzyl(methyl)(2,2,4-trimethylpentyl)silane 10

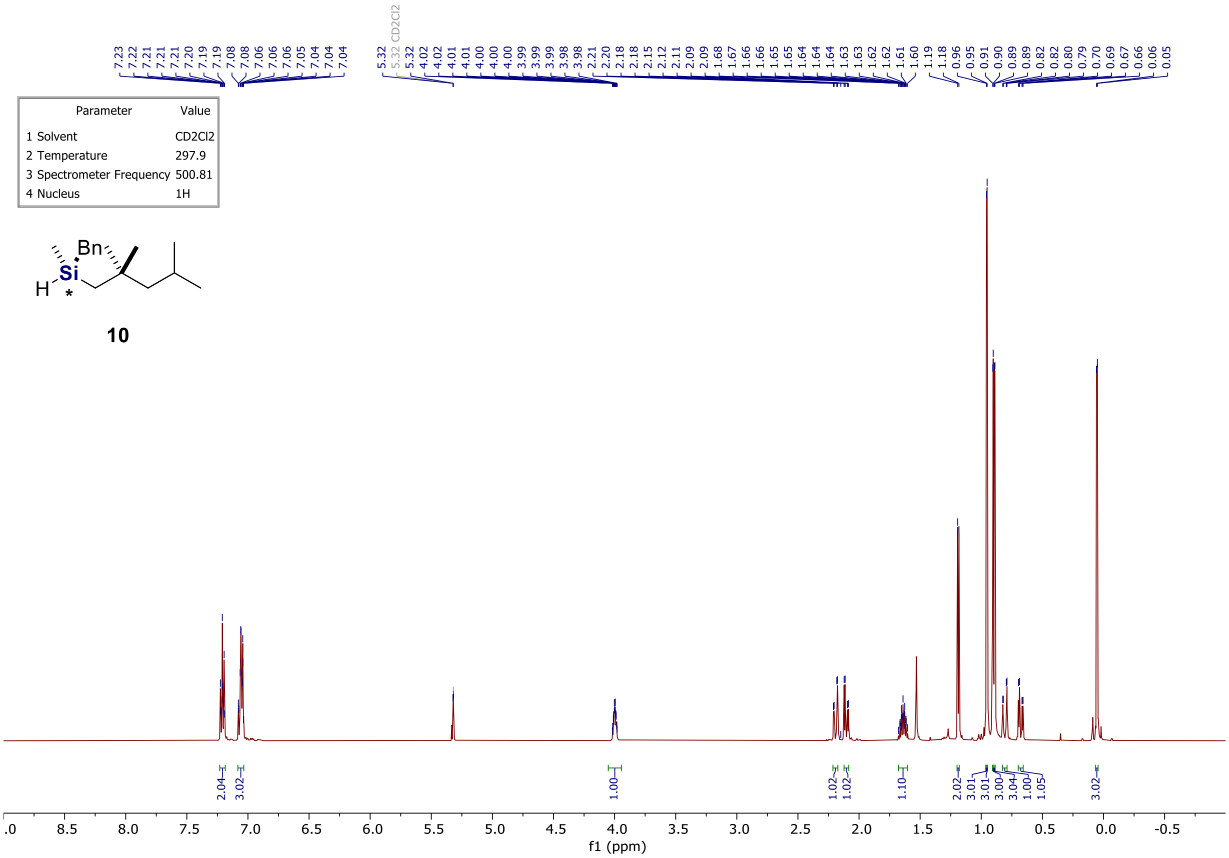

Parameter	Value
1 Solvent	CD2Cl2
2 Temperature	297.9
3 Spectrometer Frequency	500.81
4 Nucleus	1H

10

Parameter	Value
1 Solvent	CD2Cl2
2 Temperature	298.0
3 Spectrometer Frequency	125.94
4 Nucleus	13C

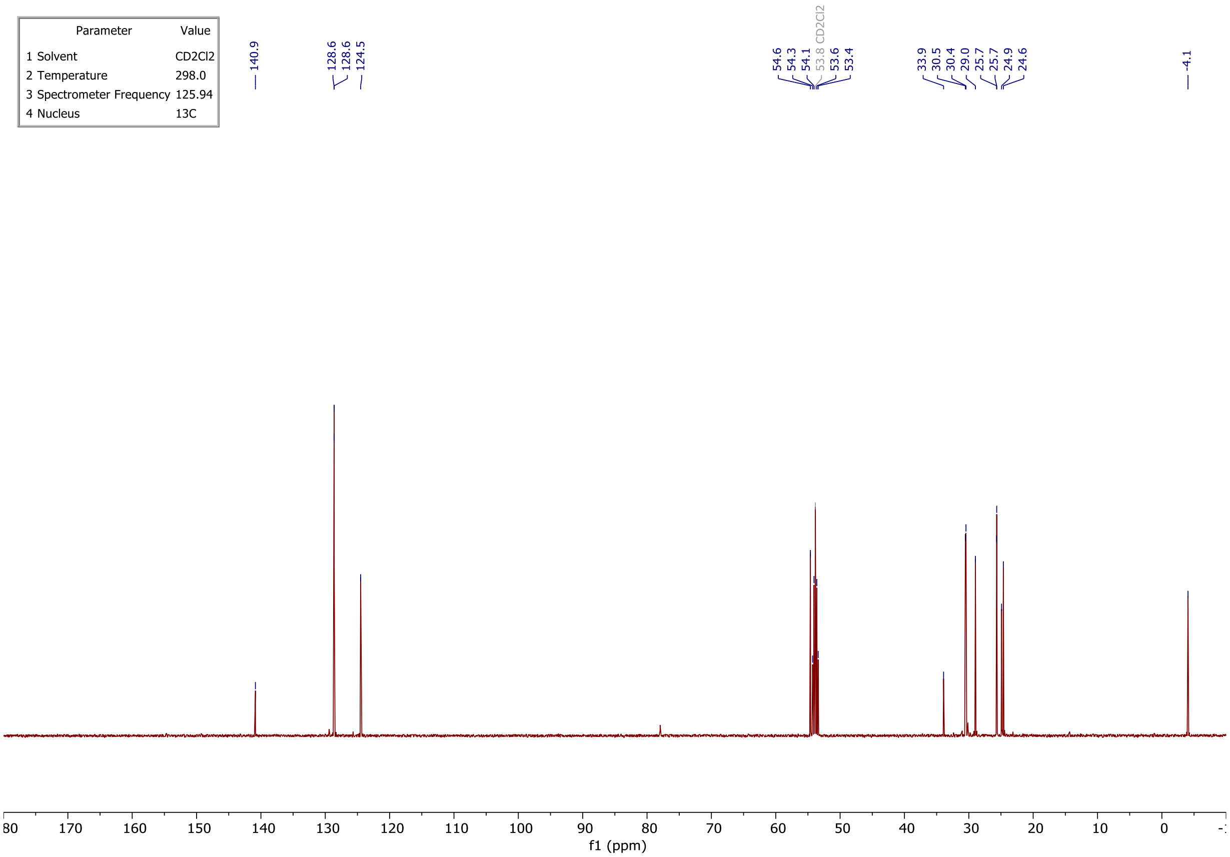

benzyl(methyl)(octyl)(2,2,4-trimethylpentyl)silane 11

Parameter	Value
1 Solvent	CD2Cl2
2 Temperature	297.9
3 Spectrometer Frequency	500.81
4 Nucleus	1H

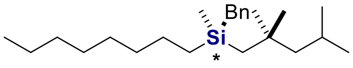

11

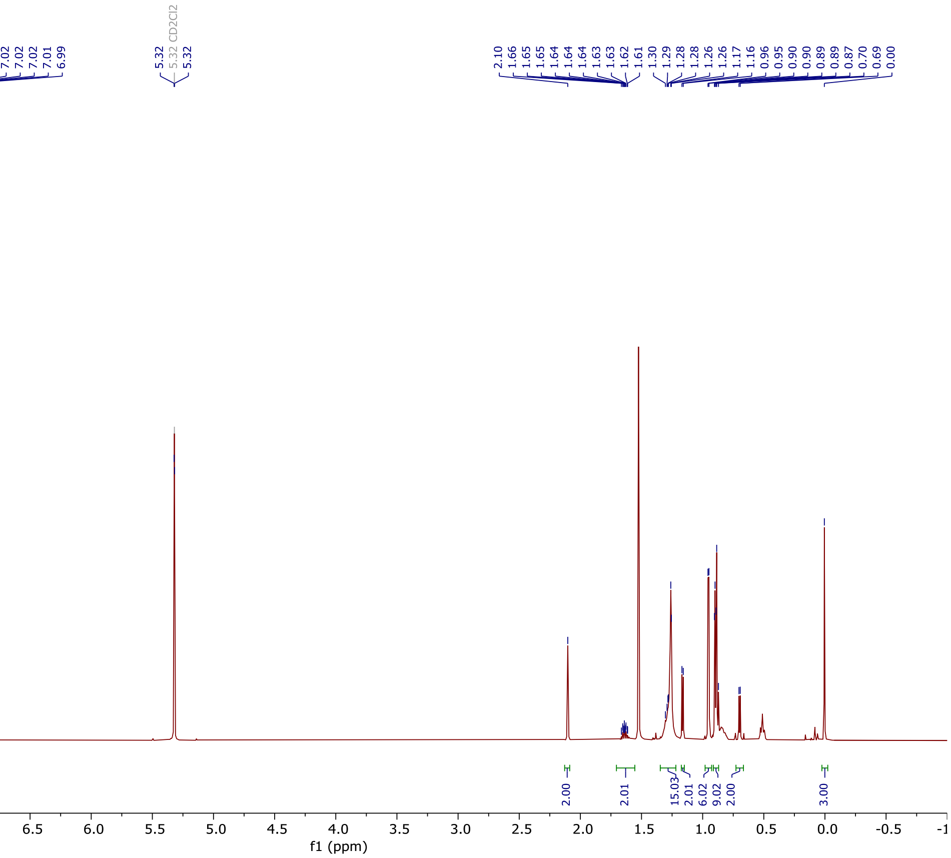

Parameter	Value
1 Solvent	CD2Cl2
2 Temperature	298.0
3 Spectrometer Frequency	125.94
4 Nucleus	13C

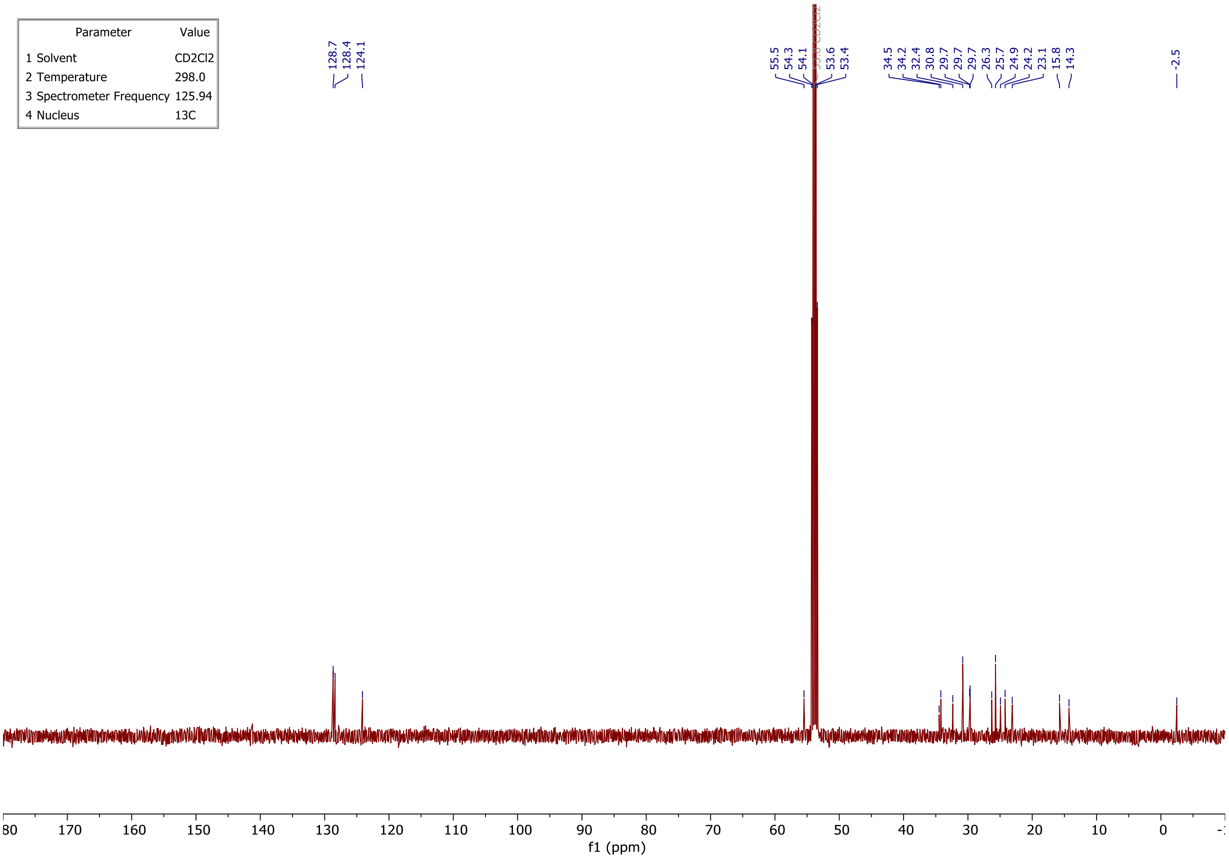

benzyl(butyl)(methyl)(2,2,4-trimethylpent-4-en-1-yl)silane 12

Parameter	Value
1 Solvent	CD2Cl2
2 Temperature	297.9
3 Spectrometer Frequency	500.81
4 Nucleus	1H

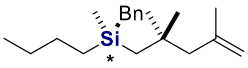

12

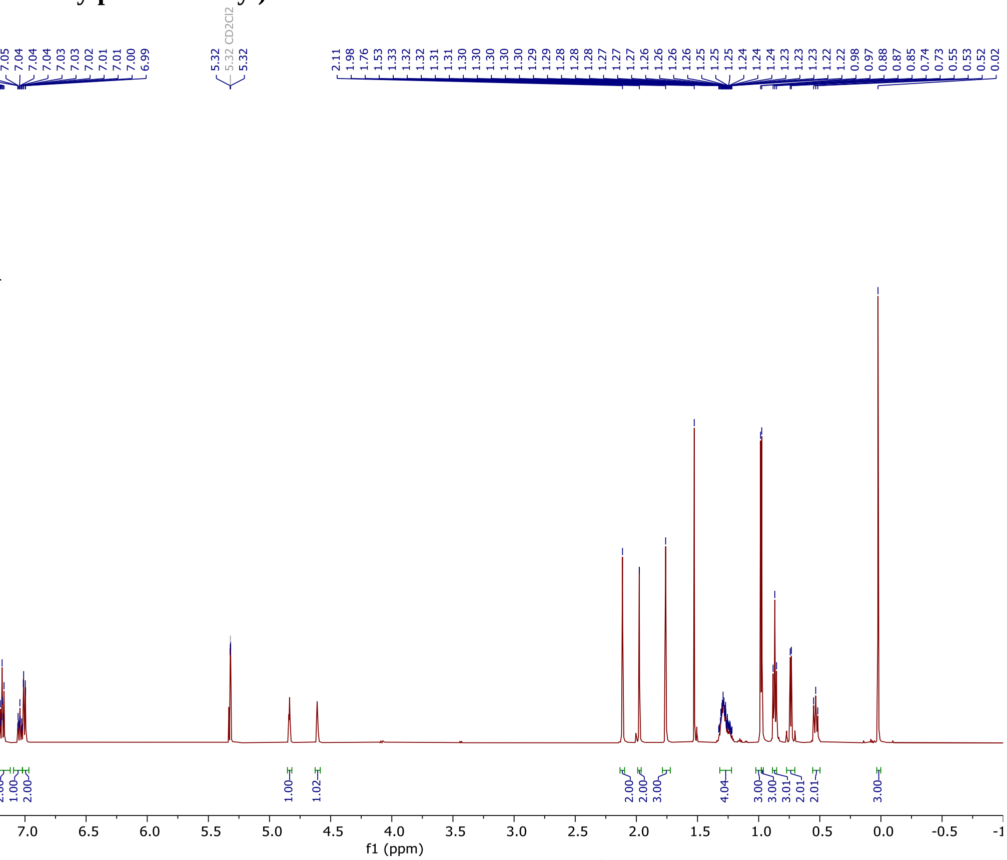

Parameter	Value
1 Solvent	CD2Cl2
2 Temperature	298.0
3 Spectrometer Frequency	125.94
4 Nucleus	13C

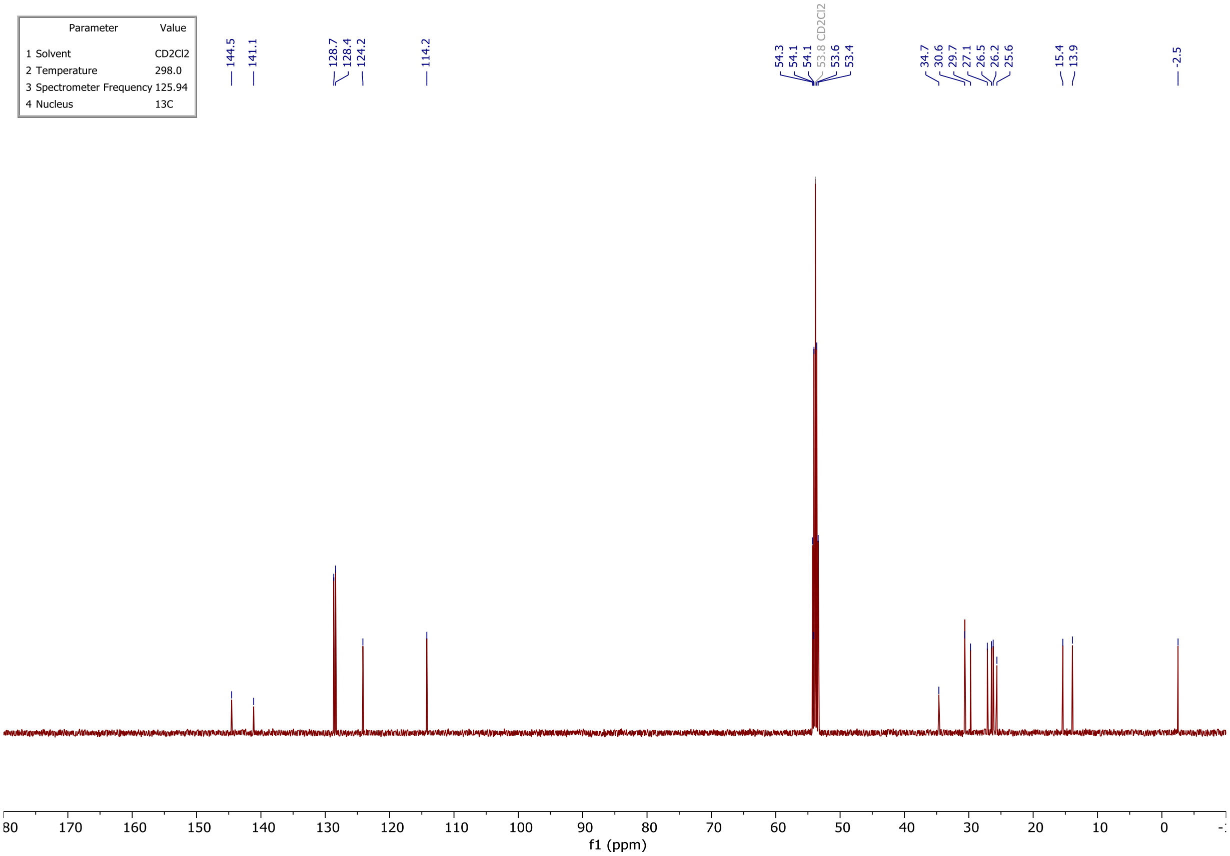

benzyl(methyl)(2,2,4-trimethylpent-4-en-1-yl)silane 13

Parameter	Value
1 Solvent	CD2Cl2
2 Temperature	298.0
3 Spectrometer Frequency	500.81
4 Nucleus	1H

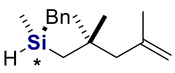

13

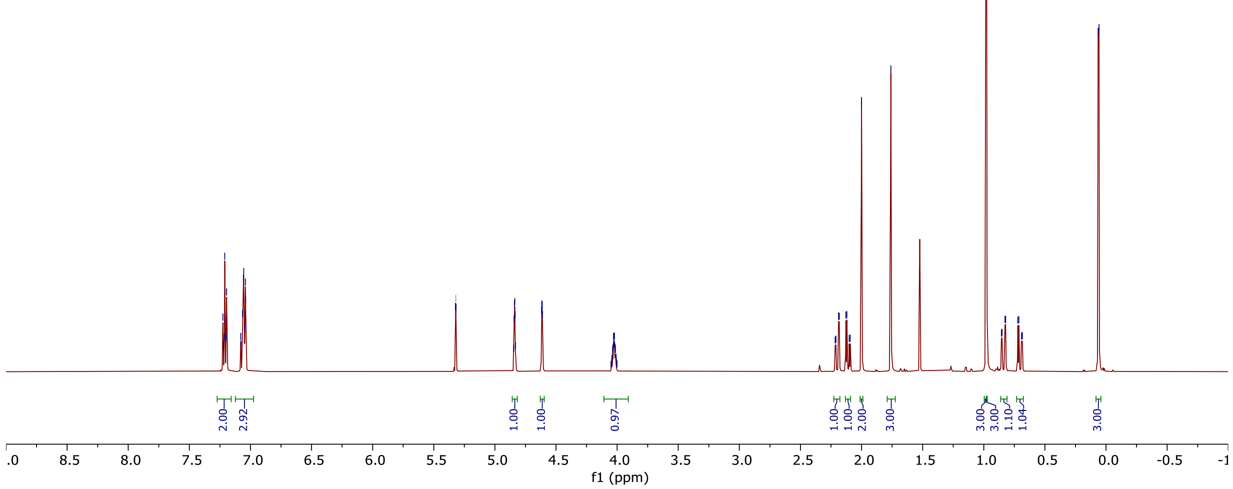

Parameter	Value
1 Solvent	CD2Cl2
2 Temperature	298.0
3 Spectrometer Frequency	125.94
4 Nucleus	13C

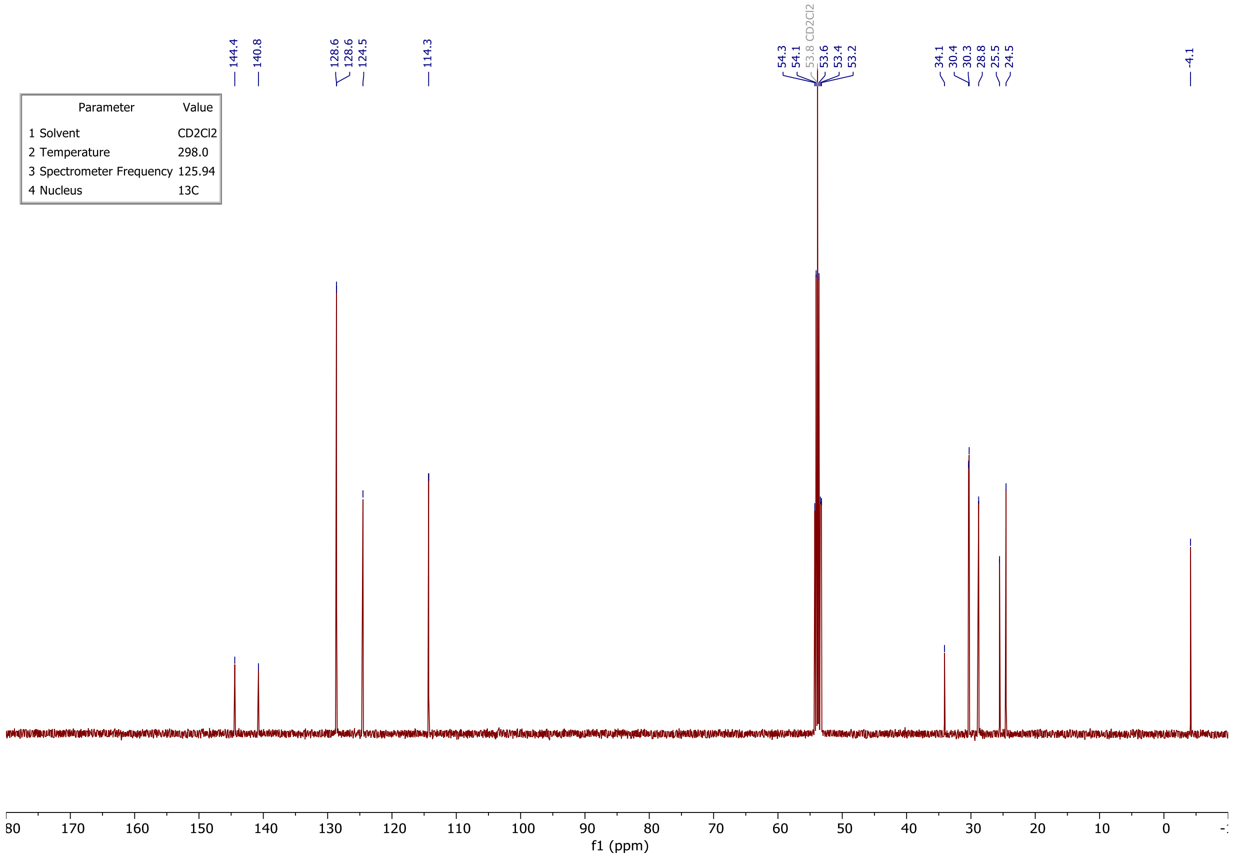

1-benzyl-1,3,3,5-tetramethylsilinane 14

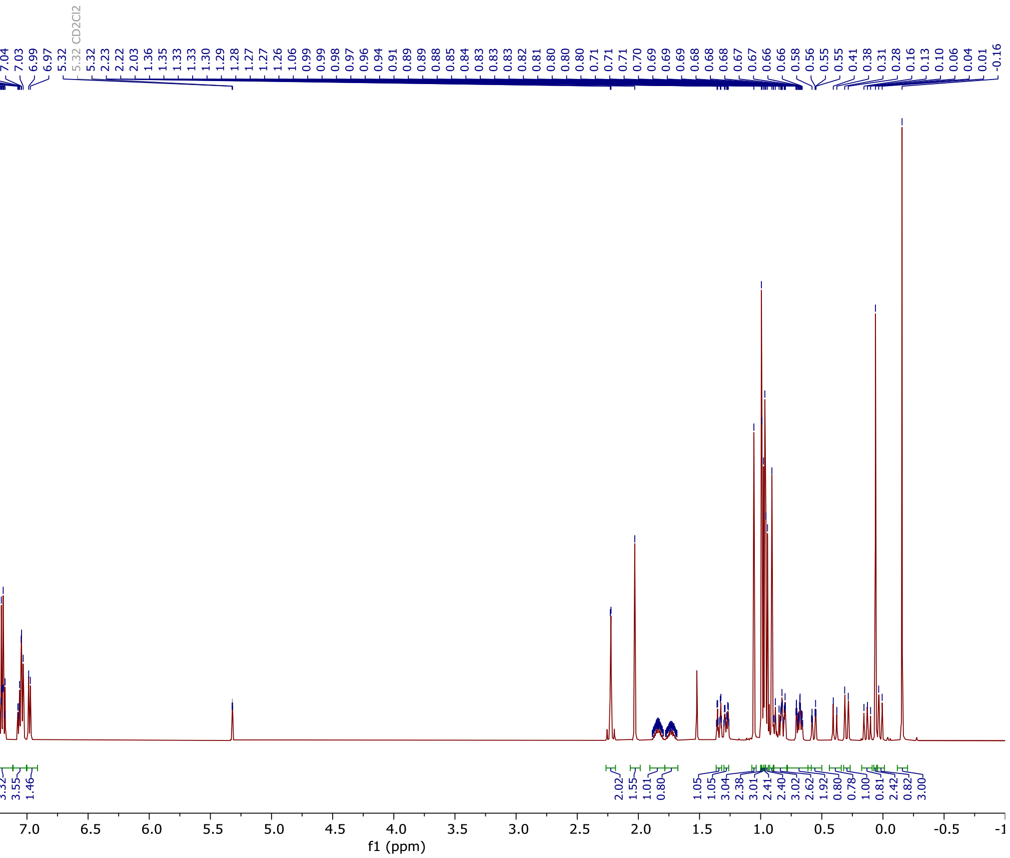

Parameter	Value
1 Solvent	CD2Cl2
2 Temperature	297.9
3 Spectrometer Frequency	500.81
4 Nucleus	1H

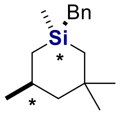

14

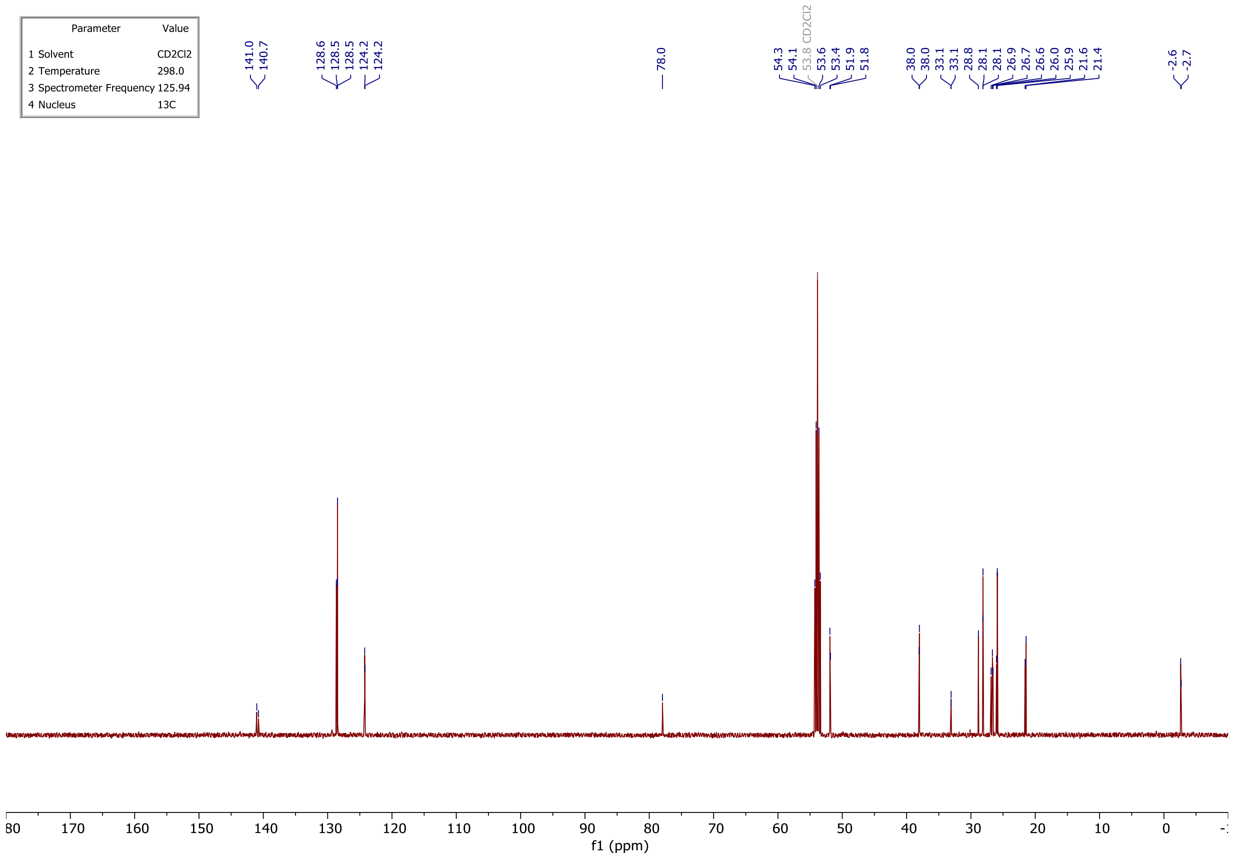

Parameter	Value
1 Solvent	CD2Cl2
2 Temperature	298.0
3 Spectrometer Frequency	125.94
4 Nucleus	13C

benzyl(methyl)(2,2,4-trimethylpent-4-en-1-yl)silanol 15

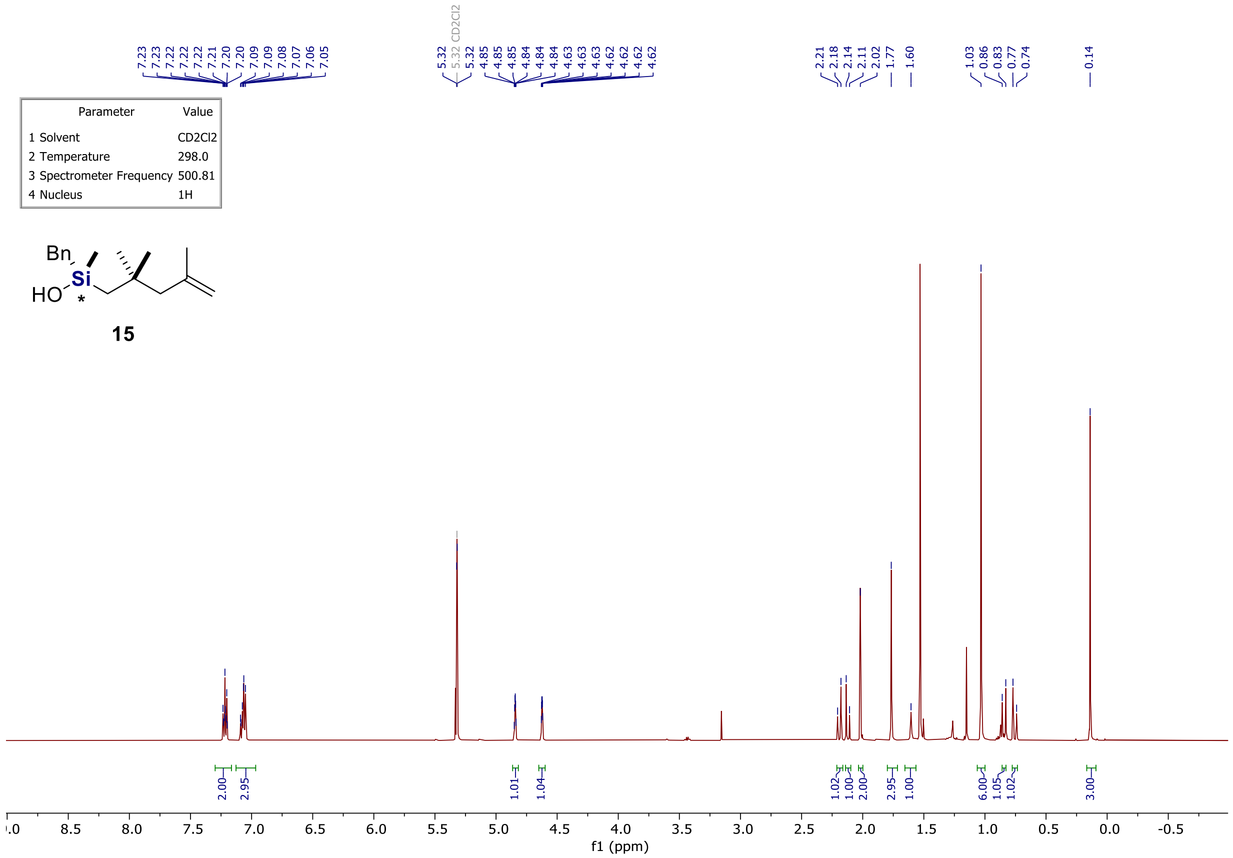

15

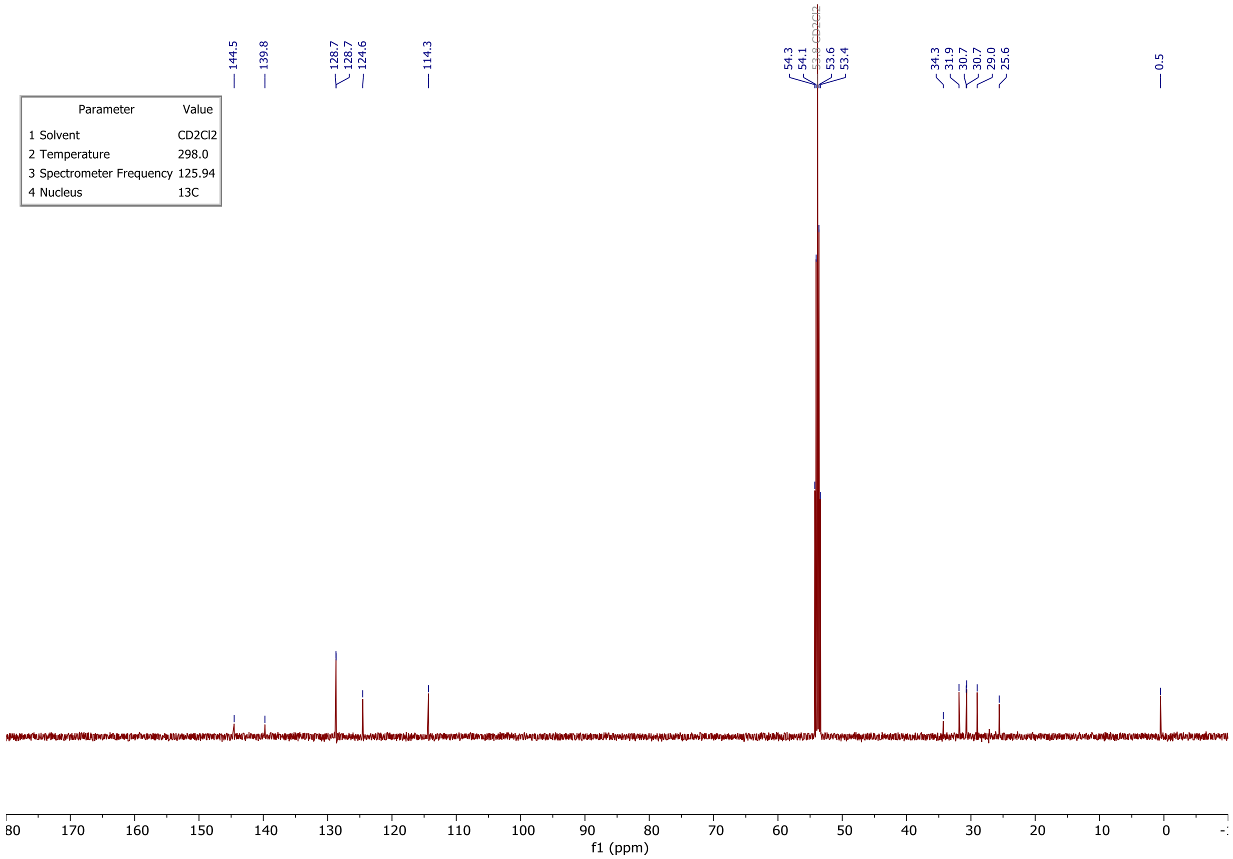

benzyl(2,6-dimethylphenoxy)(methyl)(2,2,4-trimethyl-5-(4,4,5,5-tetramethyl-1,3,2-dioxaborolan-2-yl)pentyl)silane 16

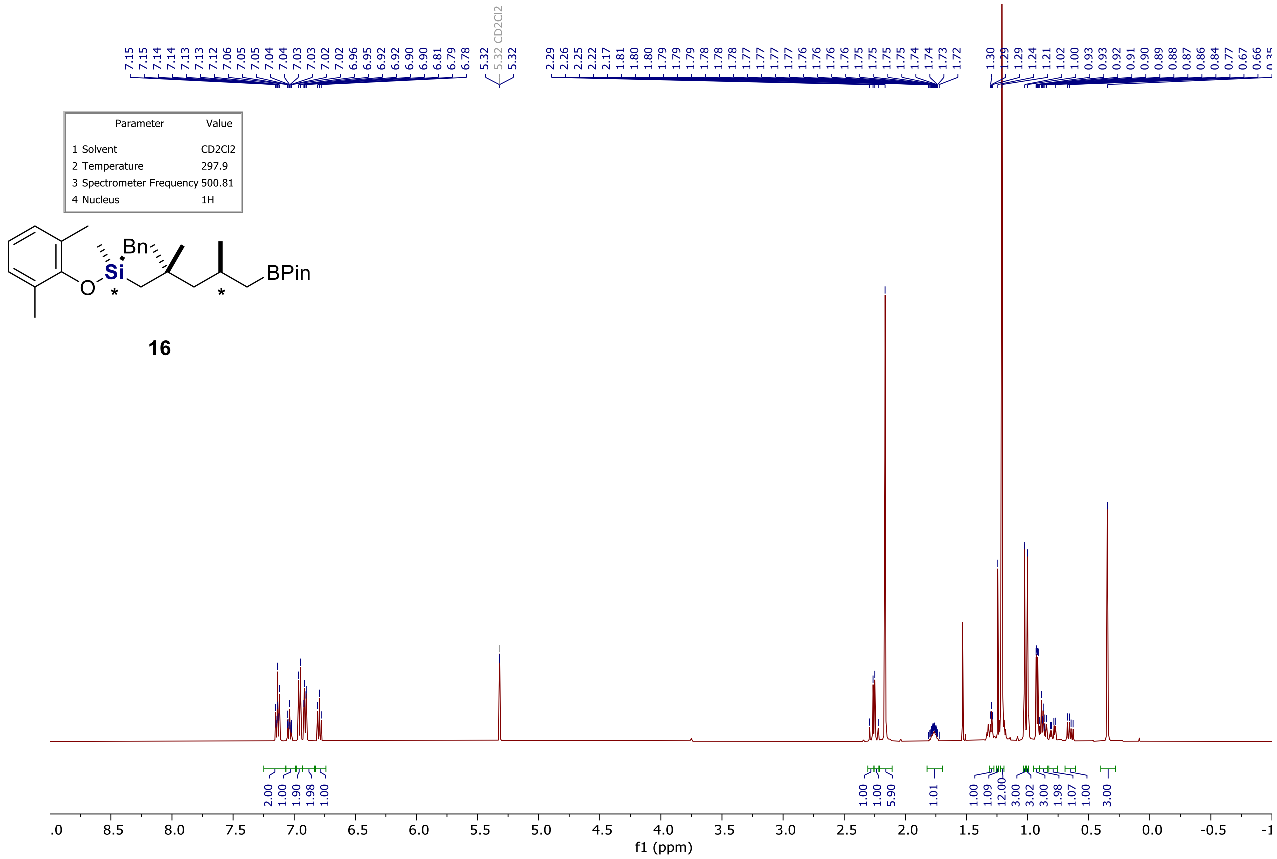

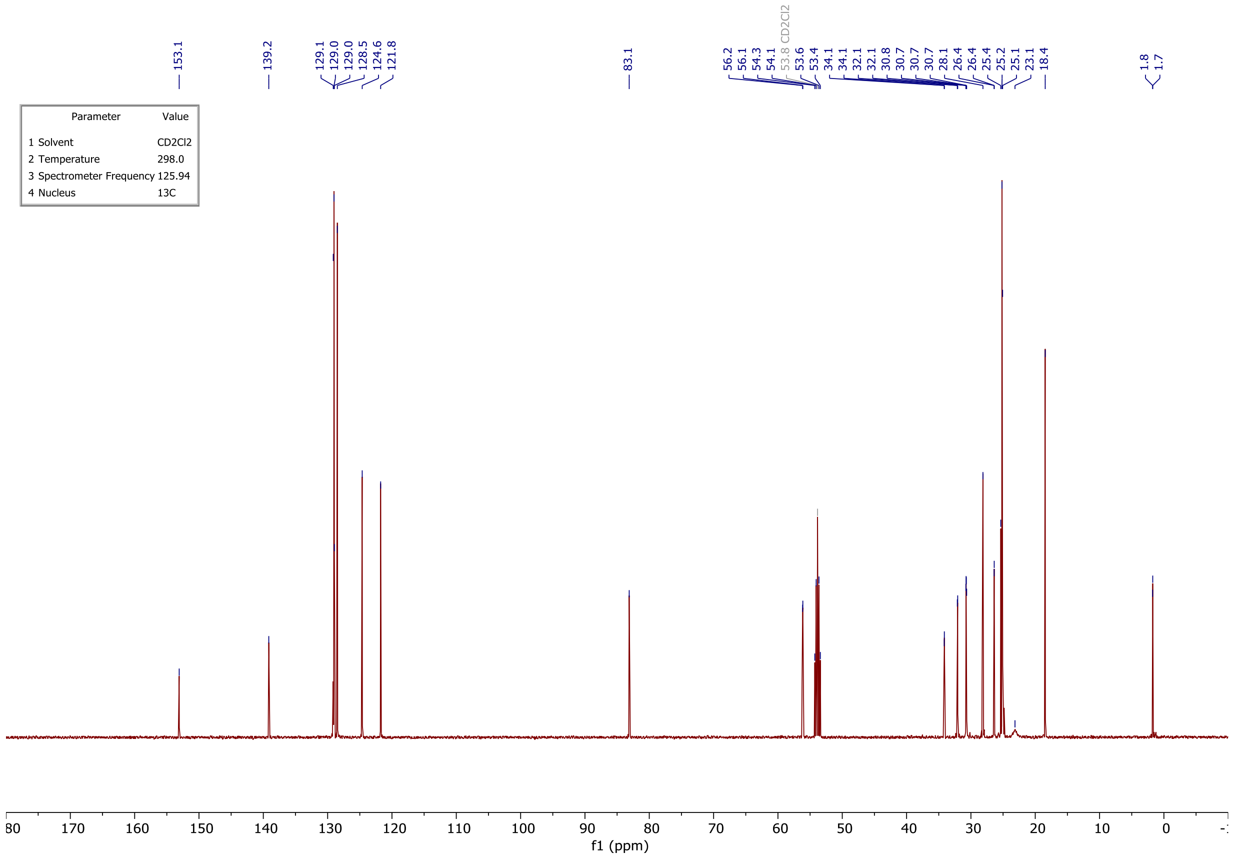

benzyl(2,6-dimethylphenoxy)(methyl)(2-methylallyl)silane 5a

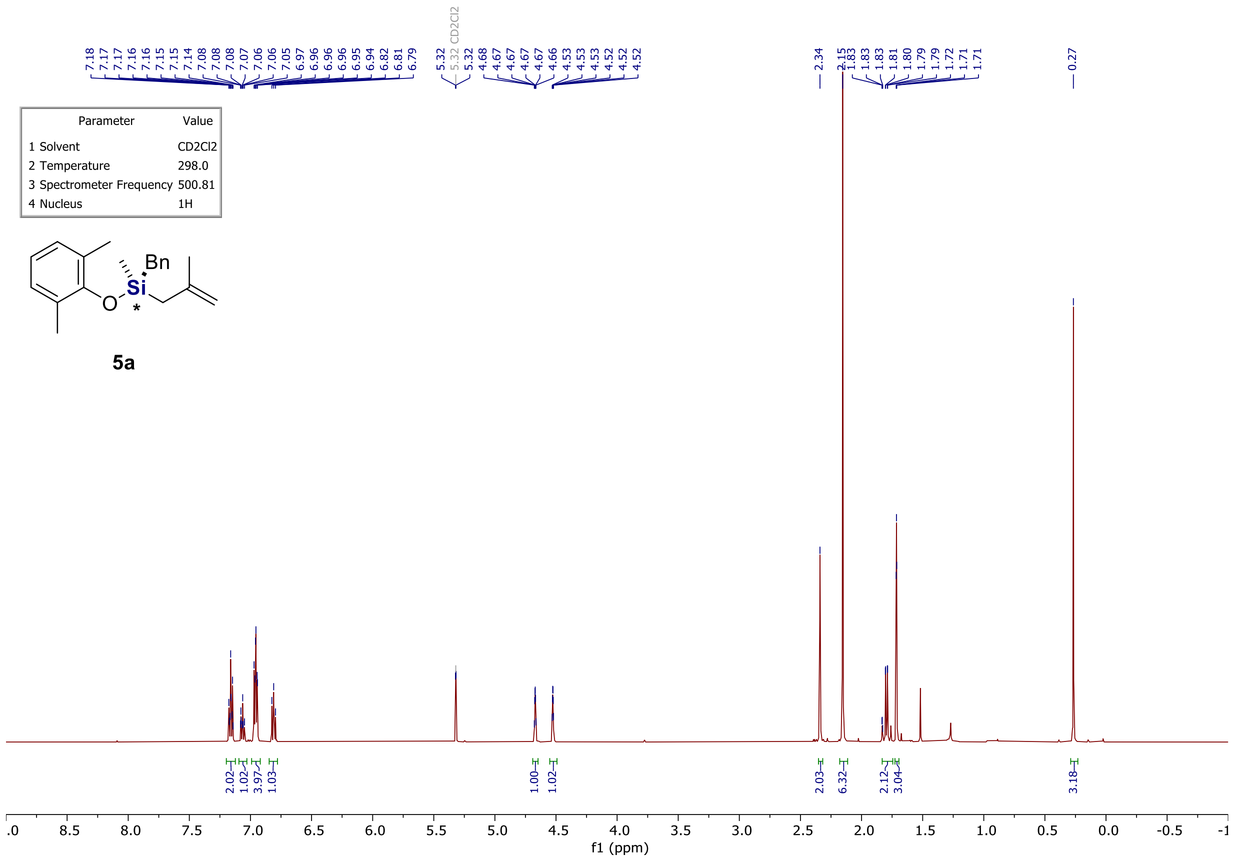

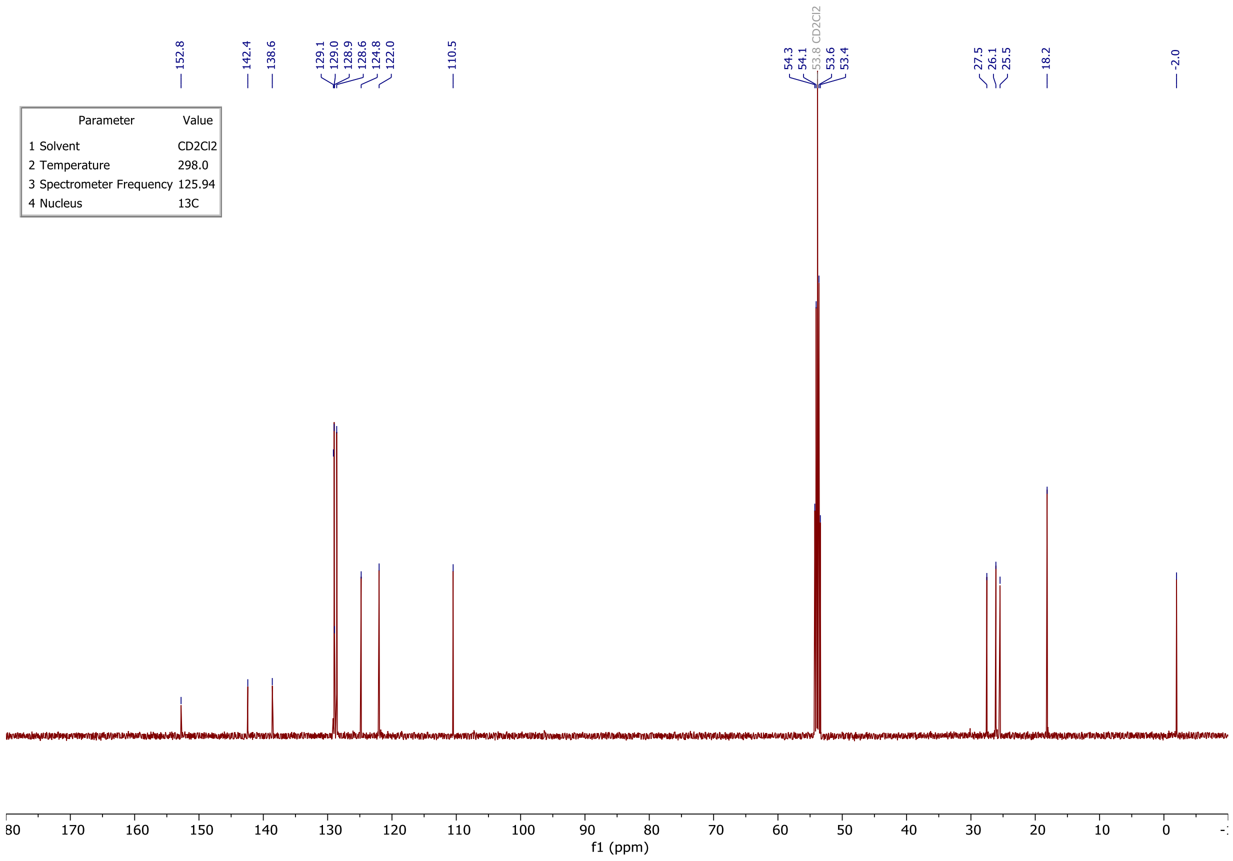

(2,6-dimethylphenoxy)(methyl)(2-methylallyl)(phenyl)silane 5b

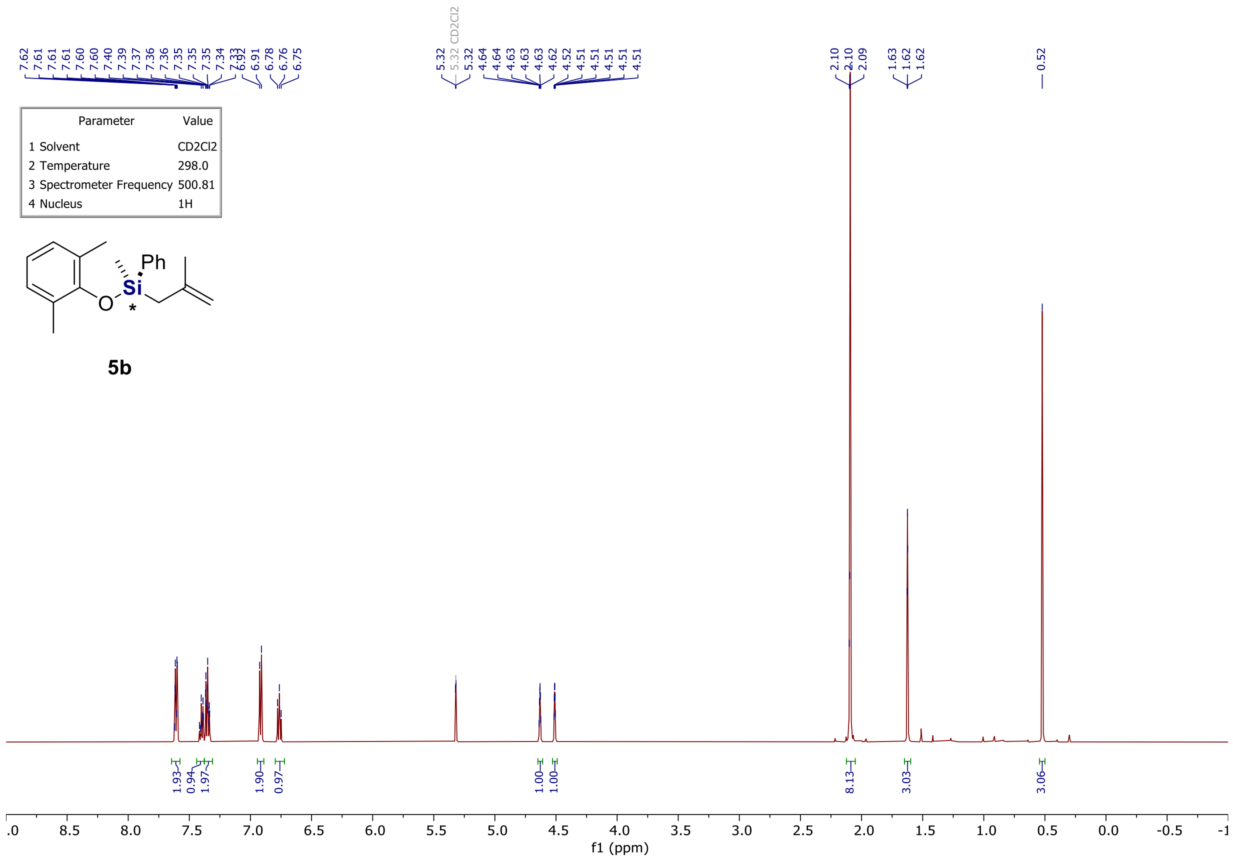

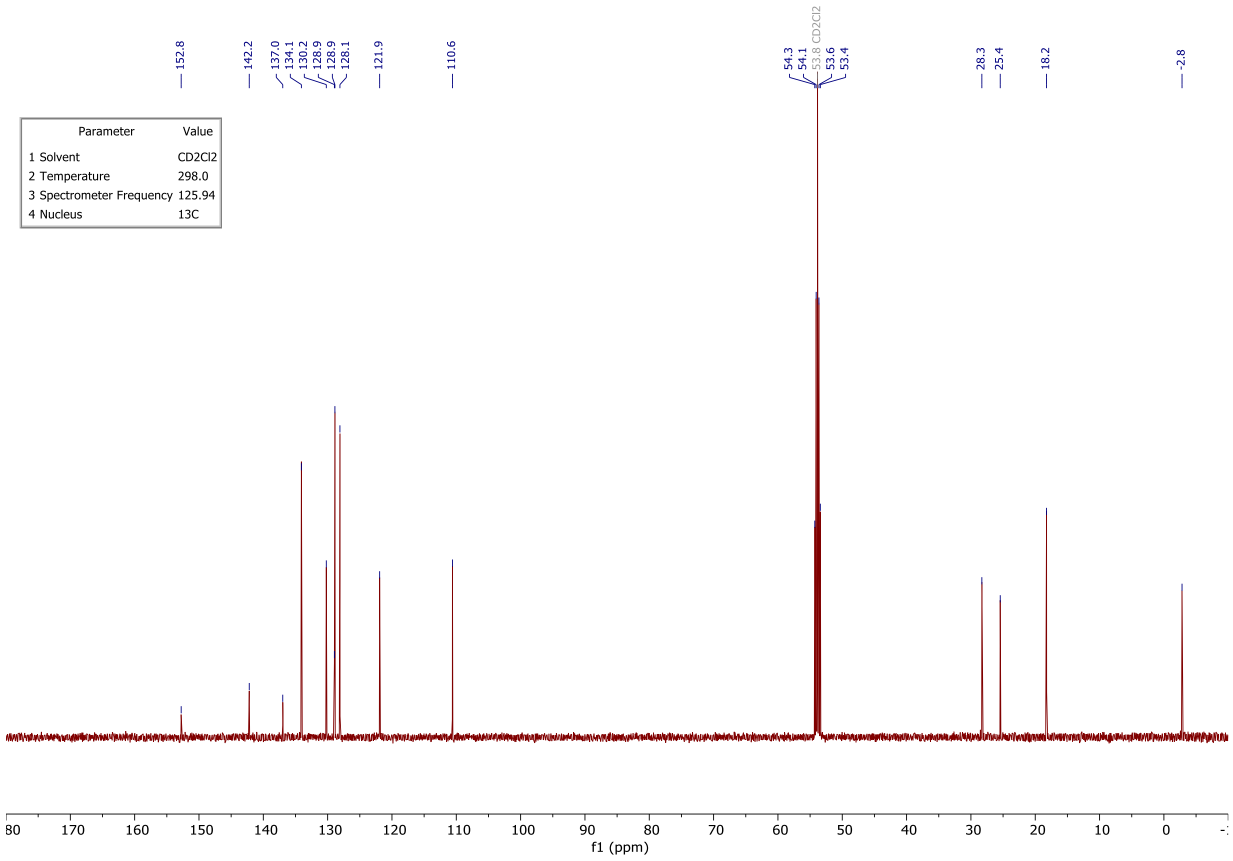

1-benzyl-1,3,3-trimethyl-5-methylenesilinane 17a

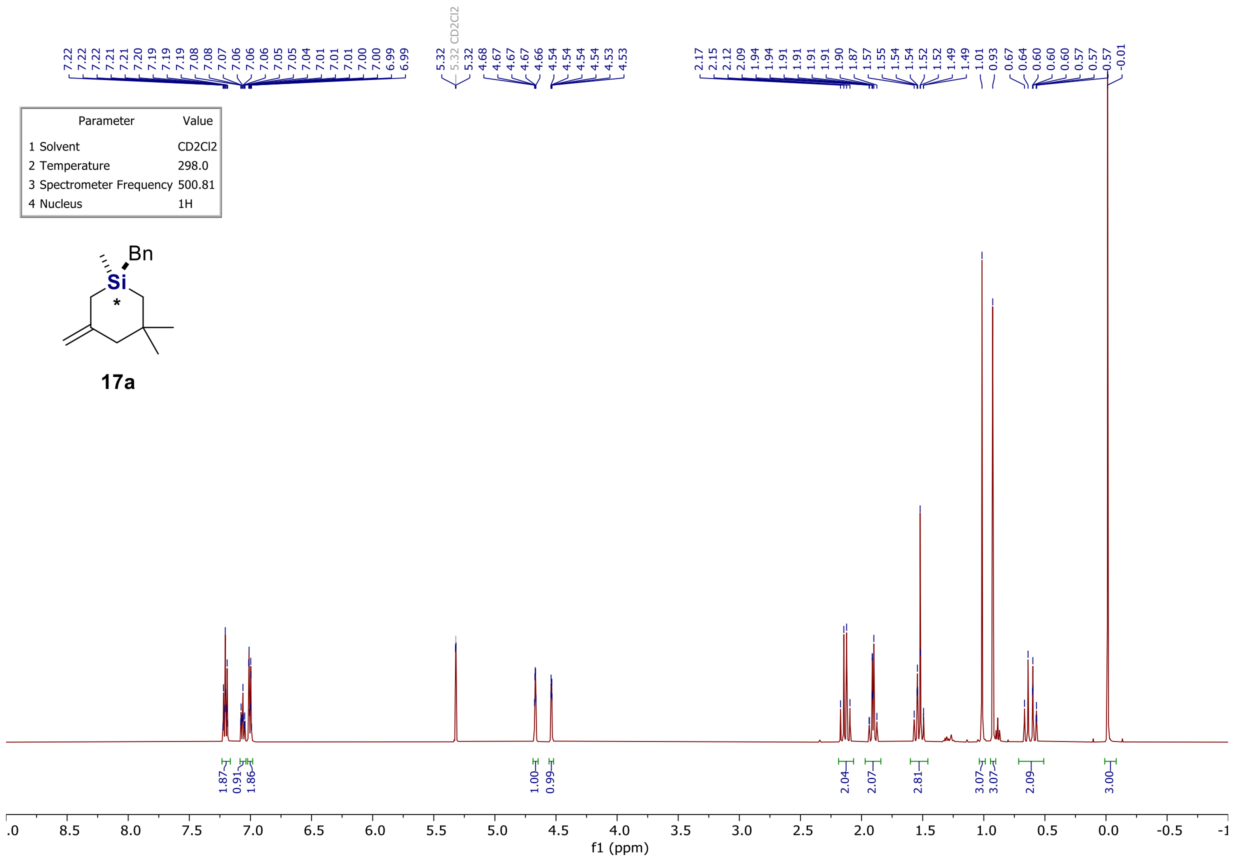

Parameter	Value
1 Solvent	CD2Cl2
2 Temperature	298.0
3 Spectrometer Frequency	500.81
4 Nucleus	1H

17a

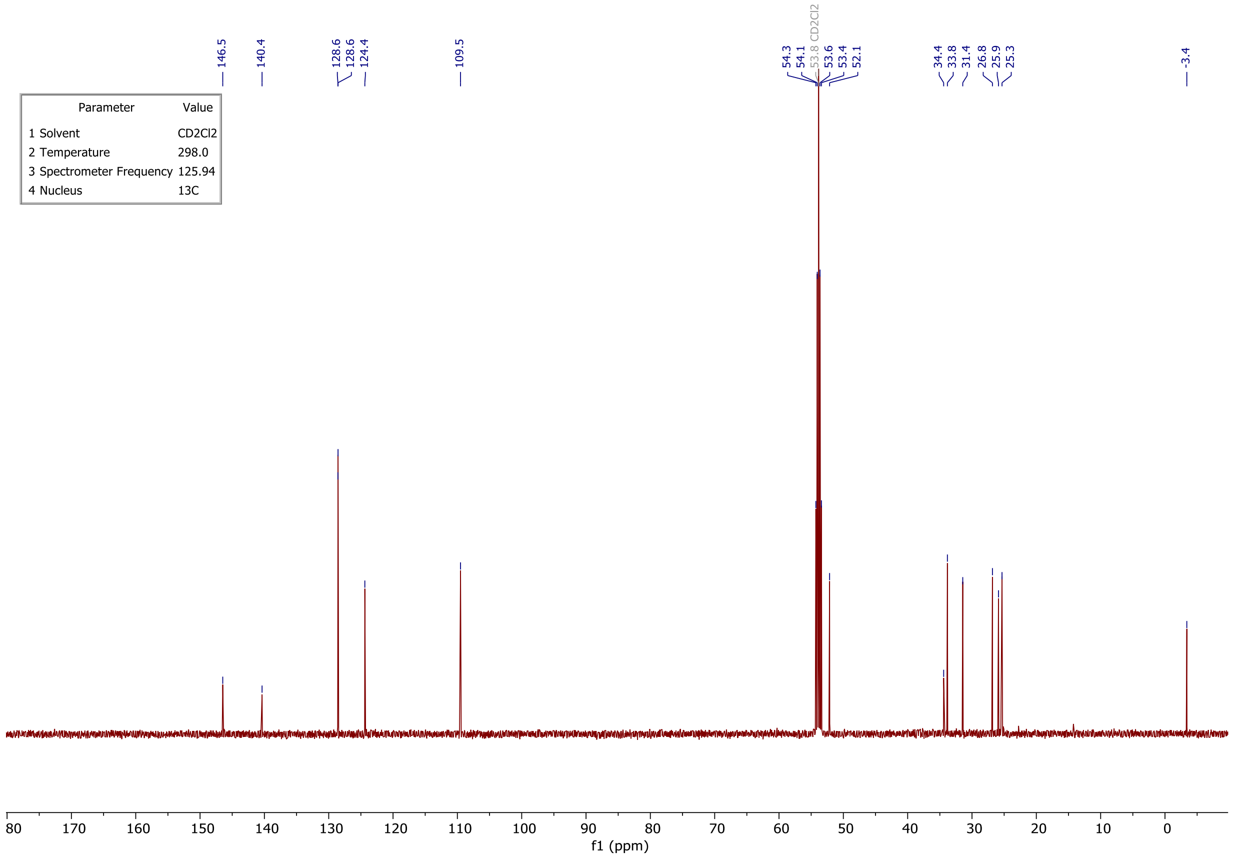

Parameter	Value
1 Solvent	CD2Cl2
2 Temperature	298.0
3 Spectrometer Frequency	125.94
4 Nucleus	13C

1,3,3-trimethyl-5-methylene-1-phenylsilinane 17b

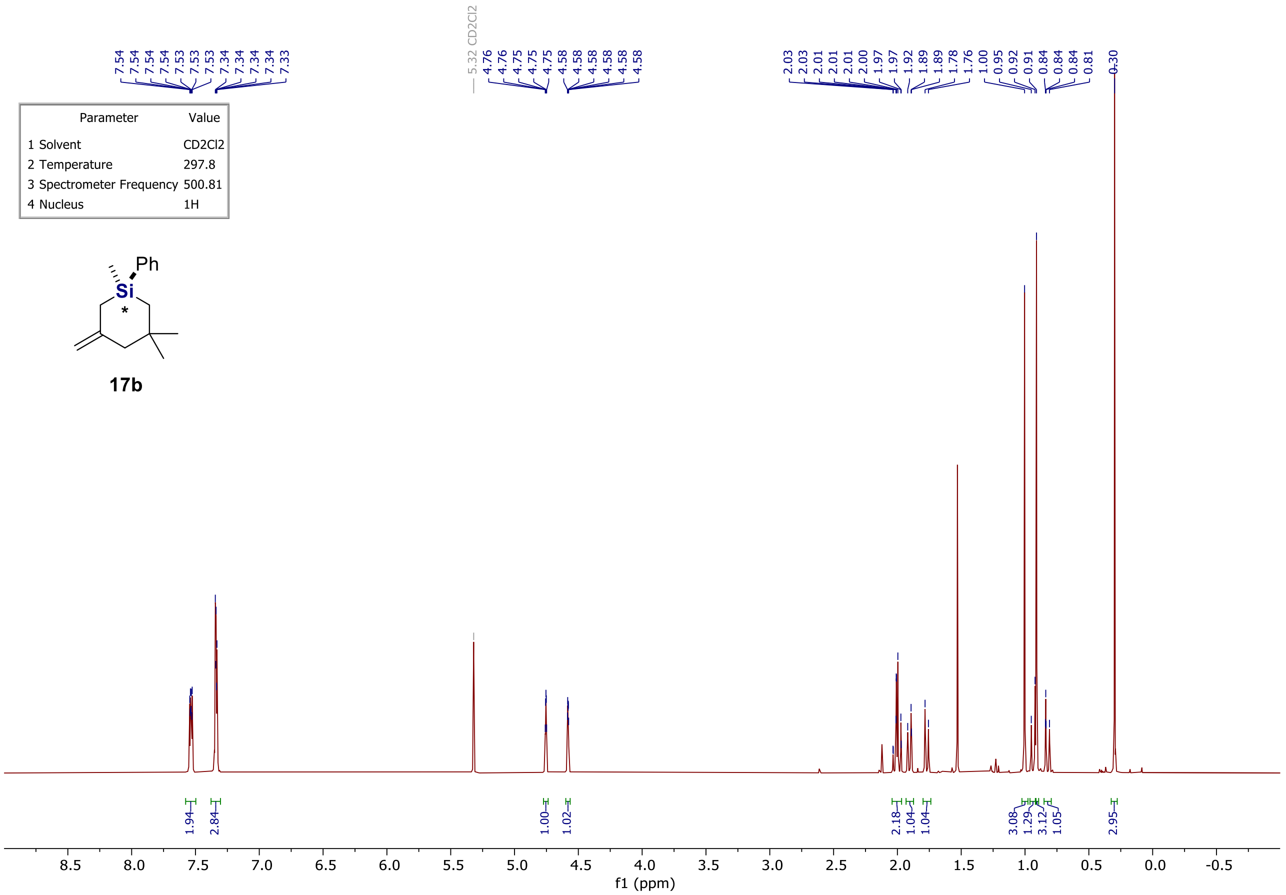

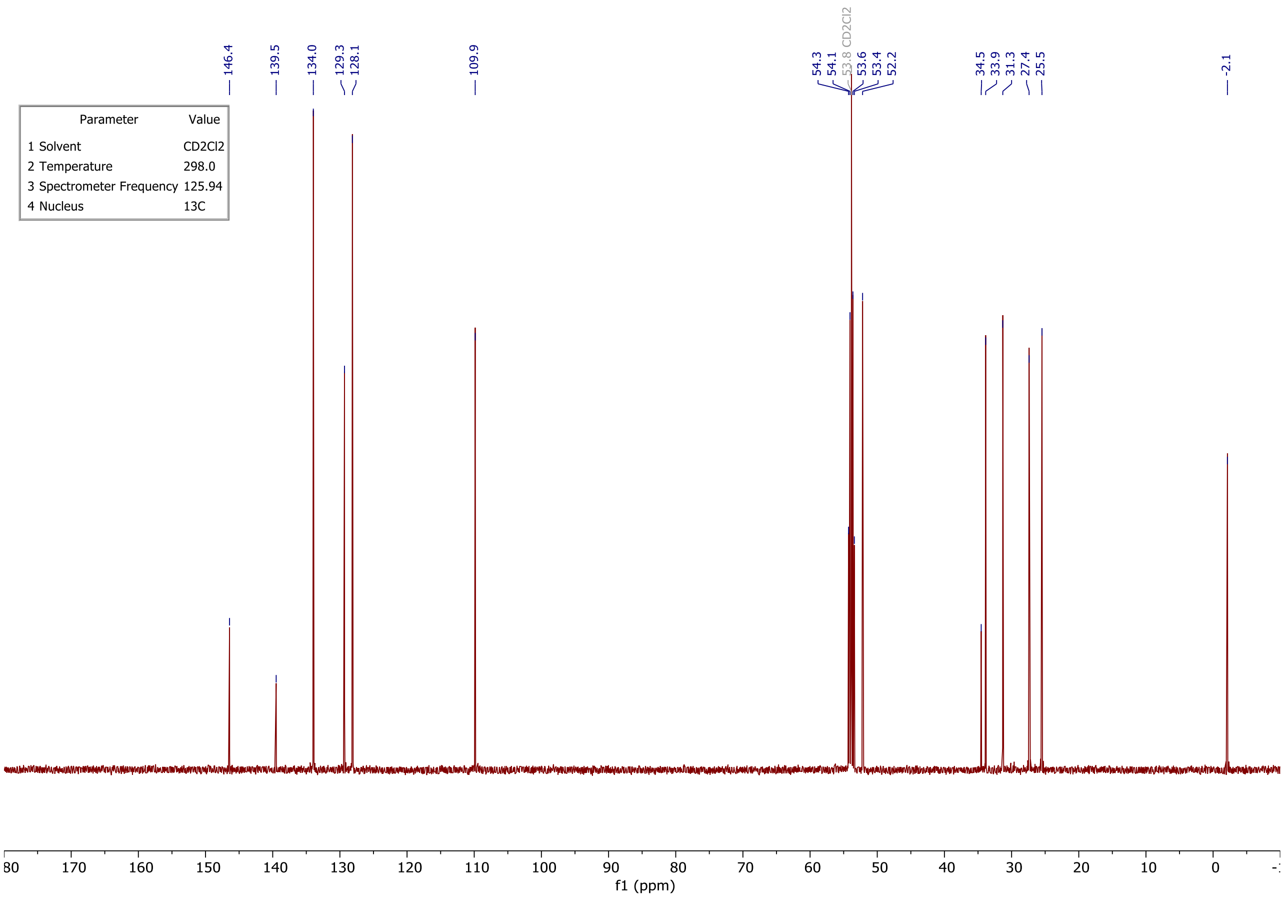

***i*IDP**

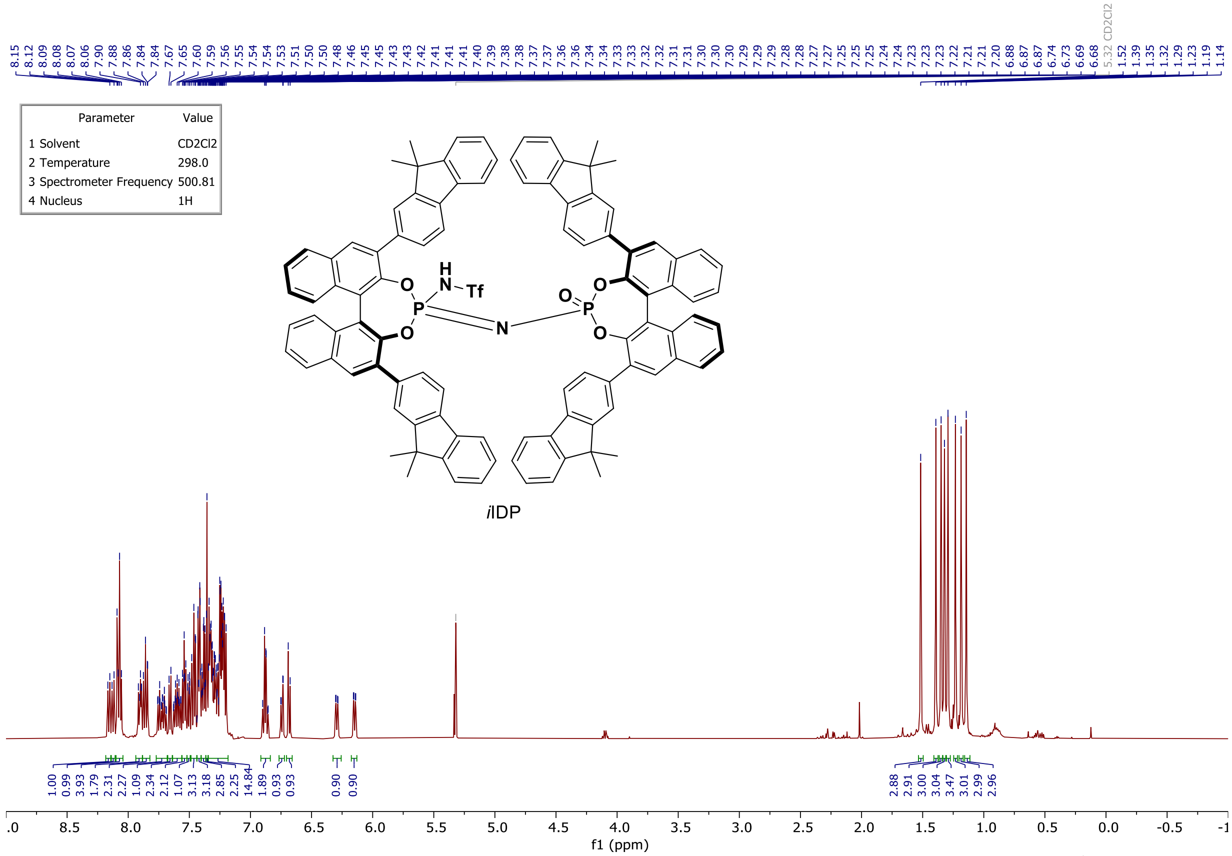
8.15 8.12 8.09 8.08 8.07 8.06 7.90 7.88 7.86 7.84 7.84 7.67 7.65 7.60 7.59 7.56 7.55 7.54 7.54 7.53 7.51 7.50 7.50 7.48 7.46 7.45 7.45 7.43 7.42 7.41 7.41 7.41 7.40 7.39 7.38 7.38 7.37 7.37 7.36 7.36 7.34 7.34 7.33 7.33 7.32 7.32 7.31 7.30 7.30 7.29 7.29 7.29 7.28 7.28 7.27 7.27 7.25 7.25 7.25 7.24 7.24 7.23 7.23 7.23 7.22 7.22 7.21 7.21 7.20 6.88 6.87 6.74 6.73 6.69 6.68 5.32 CD2Cl2 1.52 1.39 1.35 1.32 1.29 1.23 1.19 1.14
Parameter Value
1 Solvent CD2Cl2
2 Temperature 298.0
3 Spectrometer Frequency 500.81
4 Nucleus 1H
H
N
Tf
O
P
O
N
O
P
O
O
*i*IDP
1.00 0.99 3.93 1.79 2.31 2.27 1.09 2.34 2.12 1.07 3.13 3.18 2.85 2.25 14.84 1.89 0.93 0.93 0.93 0.90 0.90
2.88 2.91 3.00 3.04 3.47 3.01 2.99 2.96
8.5 8.0 7.5 7.0 6.5 6.0 5.5 5.0 4.5 4.0 3.5 3.0 2.5 2.0 1.5 1.0 0.5 0.0 -0.5
f1 (ppm)

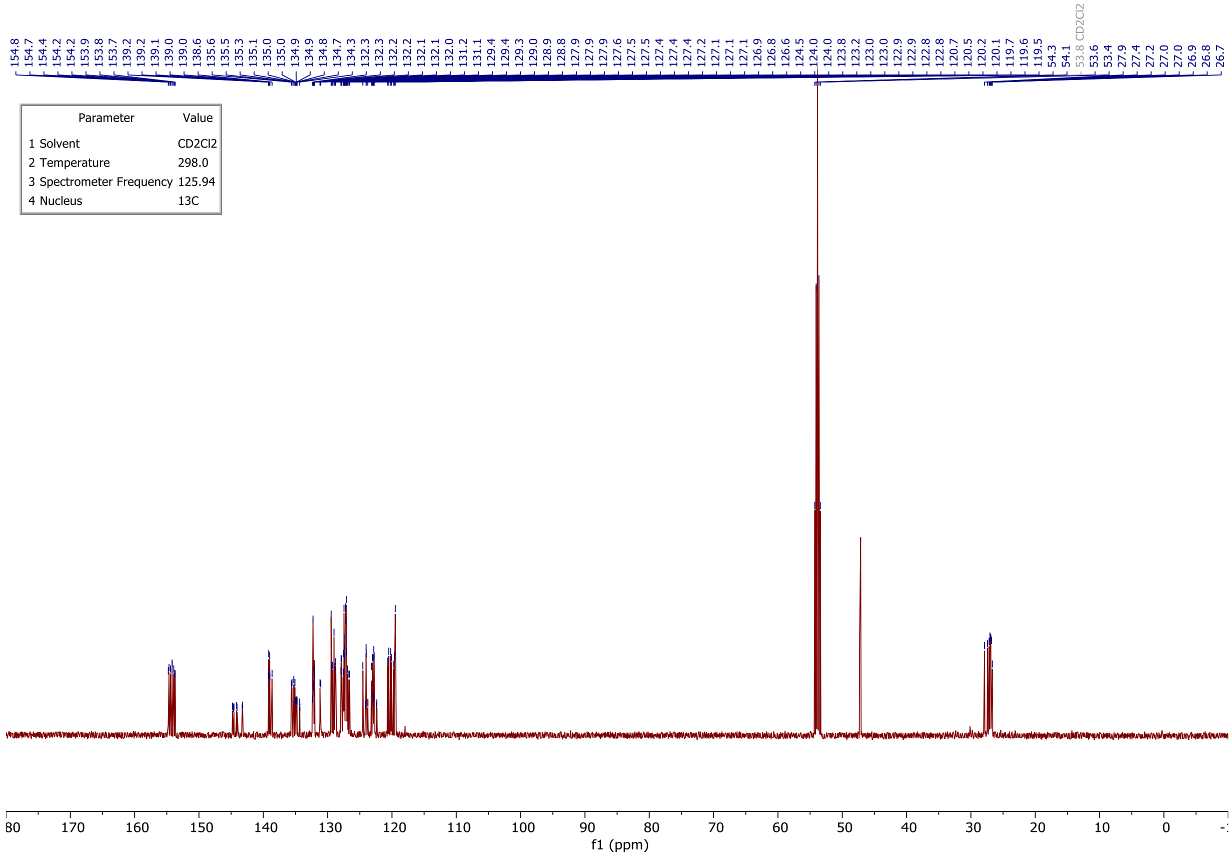
154.8 154.7 154.4 154.2 154.2 153.9 153.8 153.7 139.2 139.2 139.1 139.0 139.0 138.6 135.6 135.5 135.3 135.1 135.0 135.0 134.9 134.9 134.8 134.7 134.3 132.3 132.3 132.2 132.2 132.1 132.1 132.0 131.2 131.1 129.4 129.3 129.0 128.9 128.8 127.9 127.9 127.9 127.6 127.5 127.5 127.4 127.4 127.2 127.1 127.1 126.9 126.8 126.6 124.5 124.0 124.0 123.8 123.2 123.0 122.9 122.9 122.8 122.8 120.7 120.5 120.2 120.1 119.7 119.6 119.5 54.3 54.1 53.8 CD2Cl2 53.6 53.4 27.9 27.4 27.2 27.0 27.0 26.9 26.8 26.7
Parameter Value
1 Solvent CD2Cl2
2 Temperature 298.0
3 Spectrometer Frequency 125.94
4 Nucleus 13C
170 160 150 140 130 120 110 100 90 80 70 60 50 40 30 20 10 0
f1 (ppm)

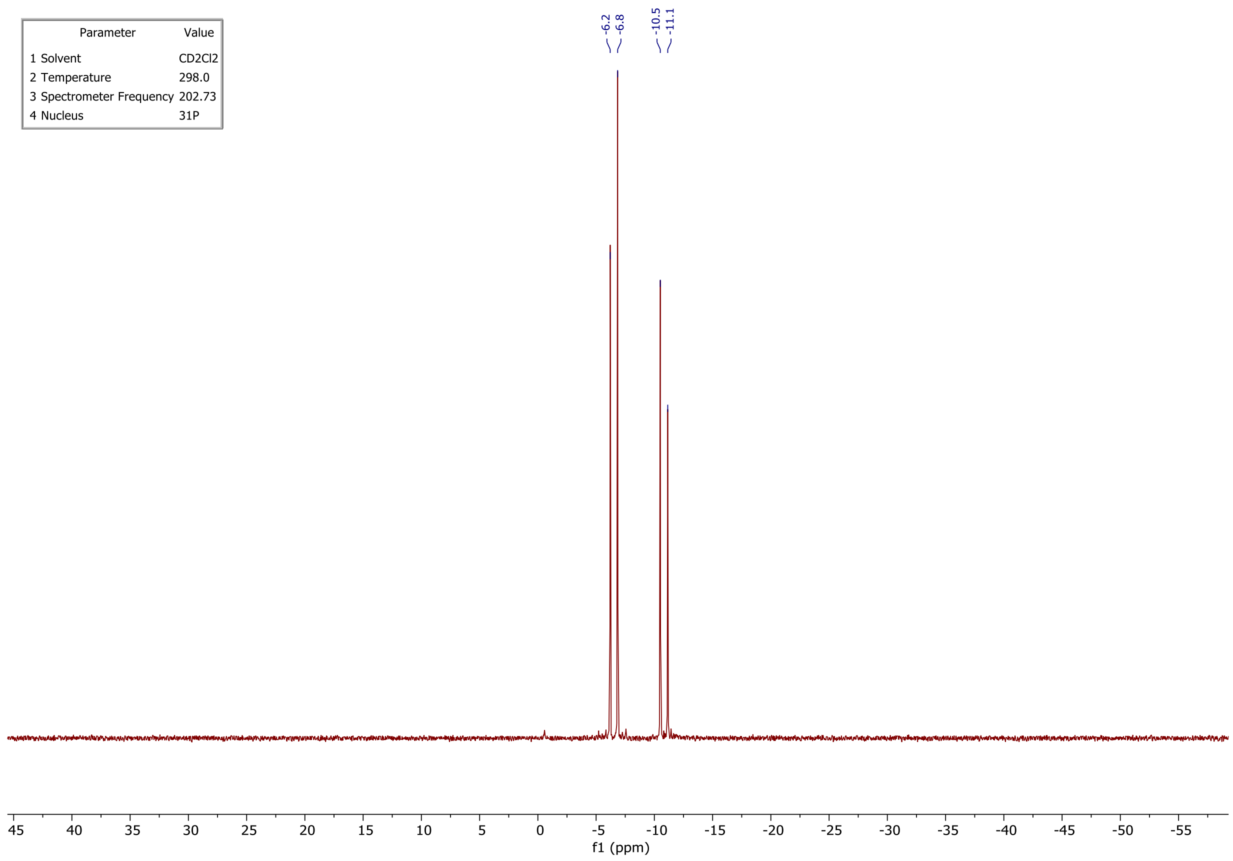
Parameter Value
1 Solvent CD2Cl2
2 Temperature 298.0
3 Spectrometer Frequency 202.73
4 Nucleus 31P
-6.2
-6.8
-10.5
-11.1
45 40 35 30 25 20 15 10 5 0 -5 -10 -15 -20 -25 -30 -35 -40 -45 -50 -55
f1 (ppm)

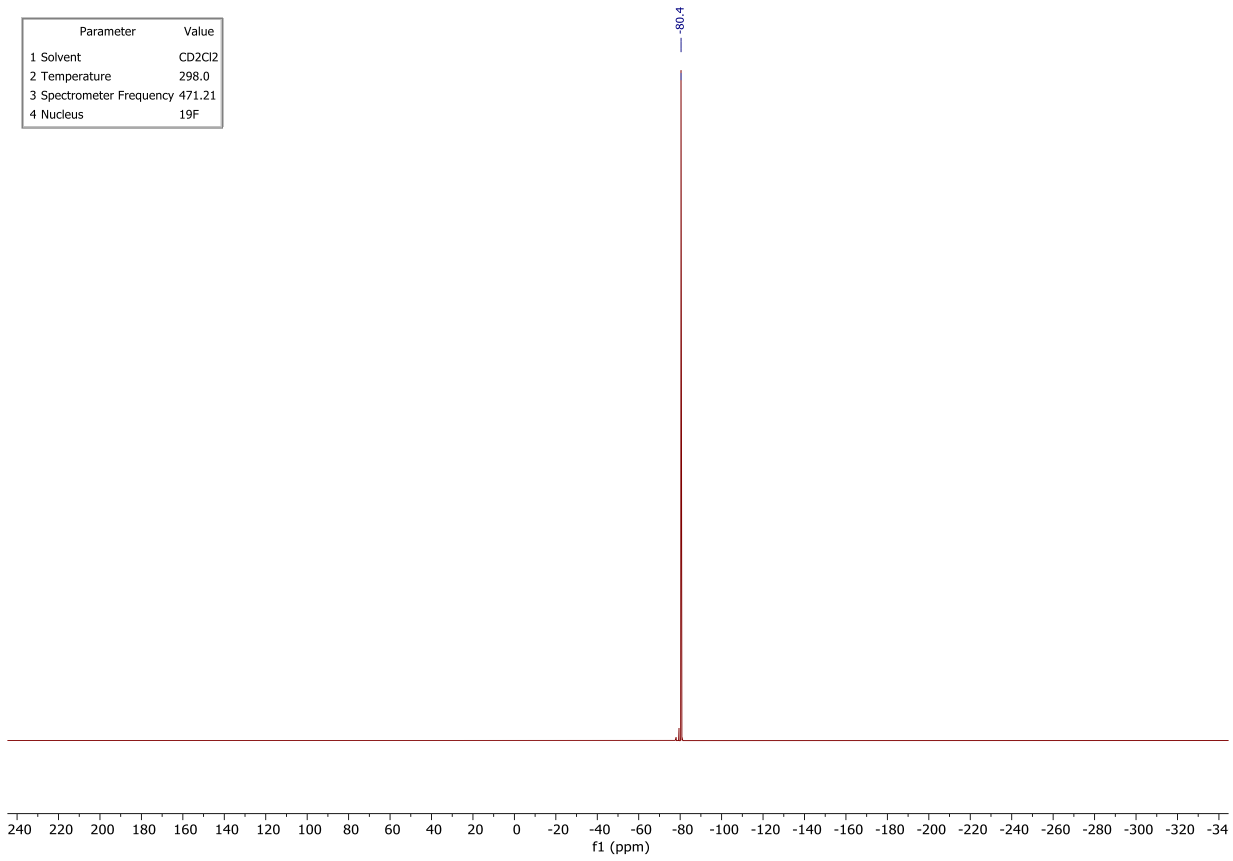
Parameter Value
1 Solvent CD2Cl2
2 Temperature 298.0
3 Spectrometer Frequency 471.21
4 Nucleus 19F
-80.4
240 220 200 180 160 140 120 100 80 60 40 20 0 -20 -40 -60 -80 -100 -120 -140 -160 -180 -200 -220 -240 -260 -280 -300 -320 -34
f1 (ppm)

IDP

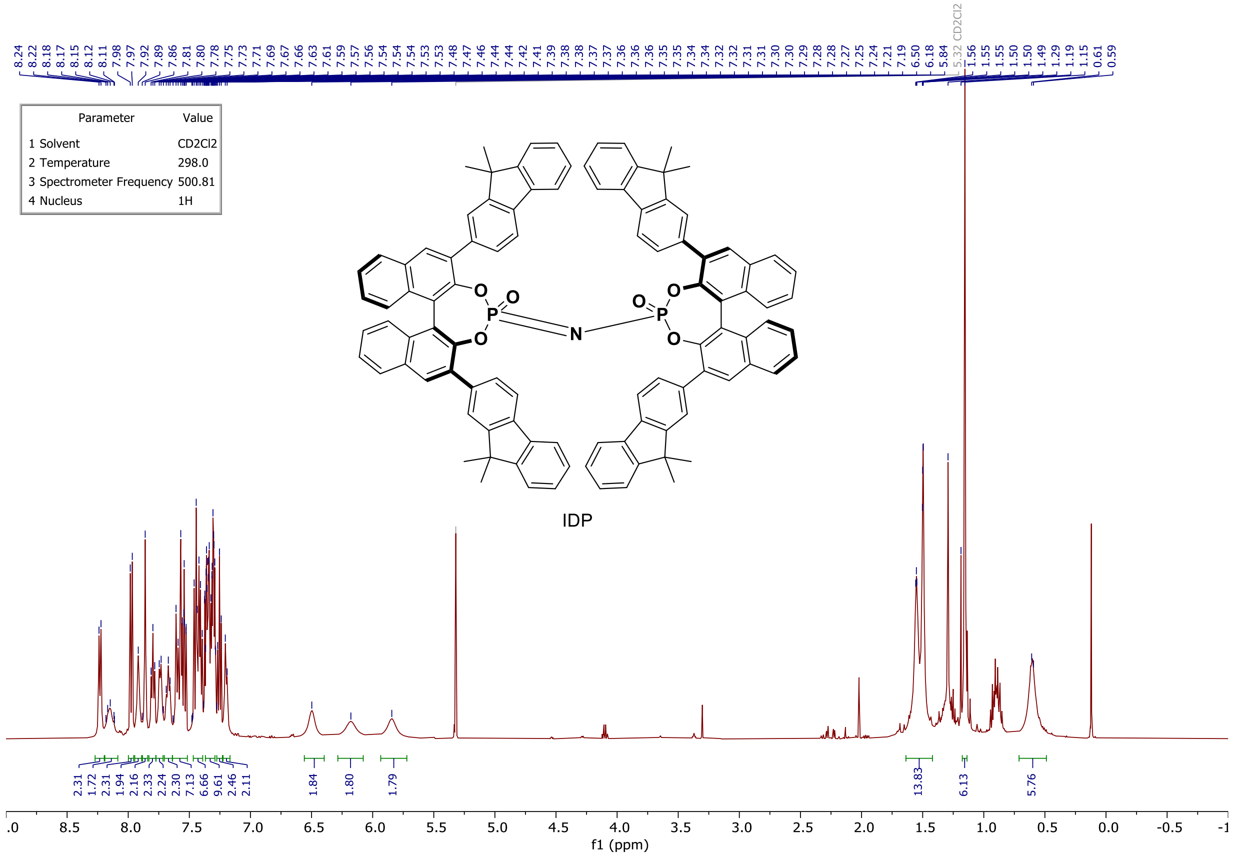
Parameter Value
1 Solvent CD2Cl2
2 Temperature 298.0
3 Spectrometer Frequency 500.81
4 Nucleus 1H
IDP
f1 (ppm)

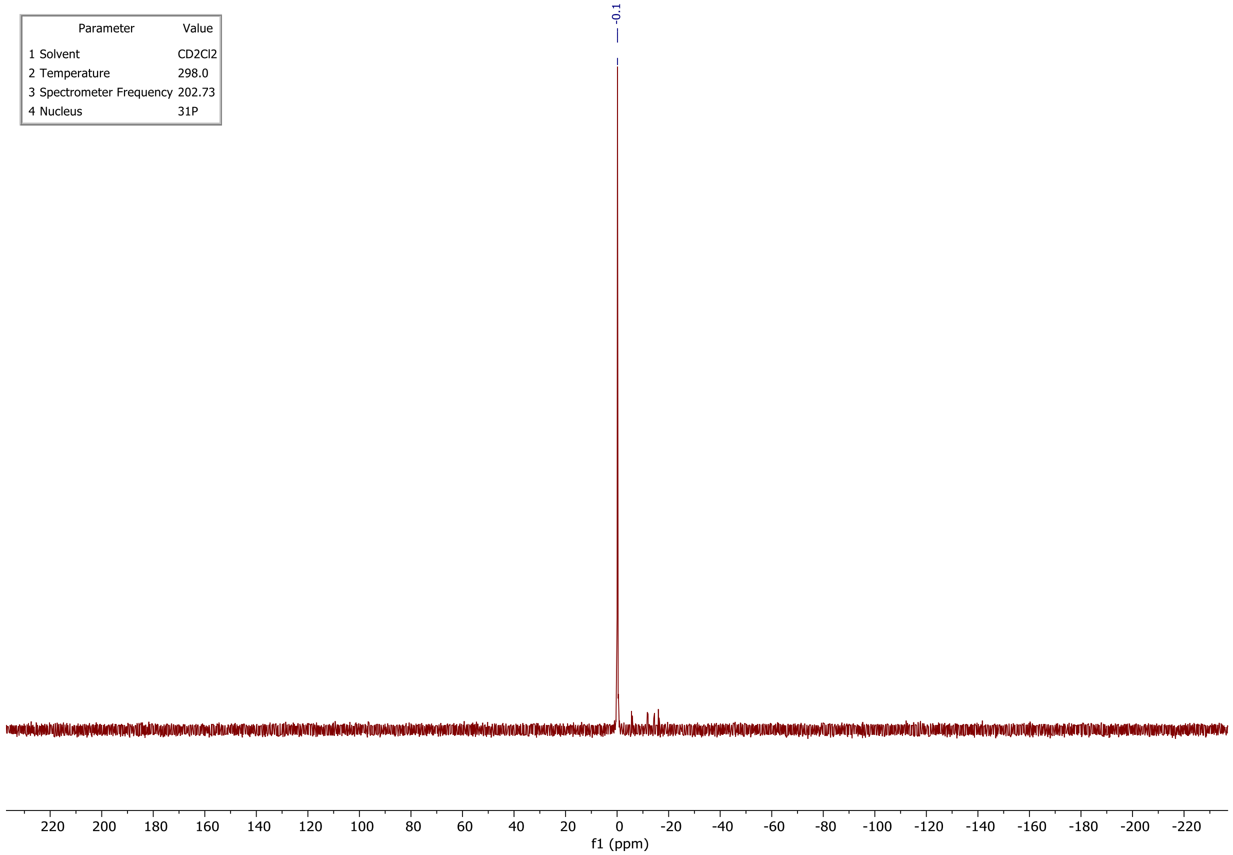
Parameter Value
1 Solvent CD2Cl2
2 Temperature 298.0
3 Spectrometer Frequency 202.73
4 Nucleus 31P
-0.1
f1 (ppm)

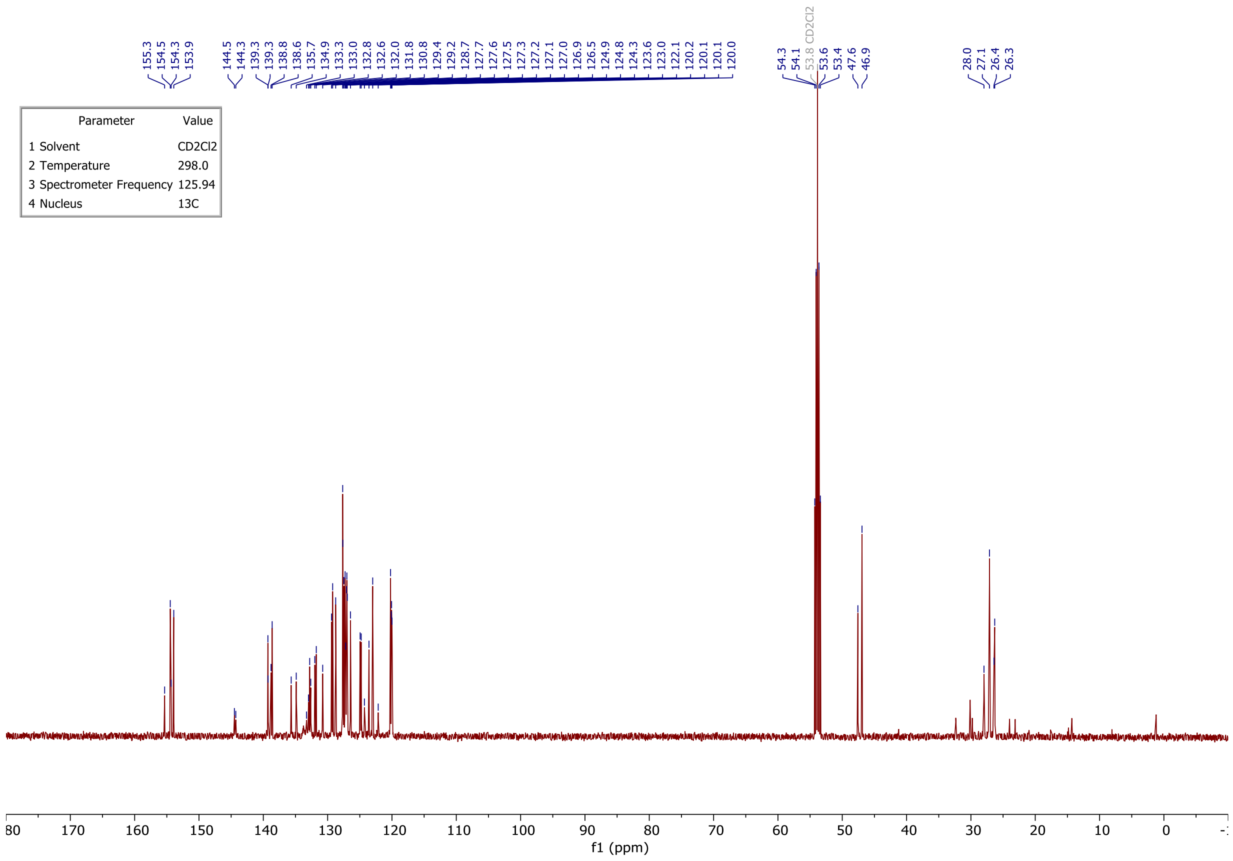
155.3
154.5
154.3
153.9
144.5
144.3
139.3
139.3
138.8
138.6
135.7
134.9
133.3
133.0
132.8
132.6
132.0
131.8
130.8
129.4
129.2
128.7
127.7
127.6
127.5
127.3
127.2
127.1
127.0
126.9
126.5
124.9
124.8
124.3
123.6
123.0
122.1
120.2
120.1
120.1
120.0
54.3
54.1
53.8 CD2Cl2
53.6
53.4
47.6
46.9
28.0
27.1
26.4
26.3
Parameter Value
1 Solvent CD2Cl2
2 Temperature 298.0
3 Spectrometer Frequency 125.94
4 Nucleus 13C
f1 (ppm)

14. Copies of HPLC traces

HPLC column: OD-3, *n*-Heptane = 100, 0.5 mL/min, 298 K, 220 nm.

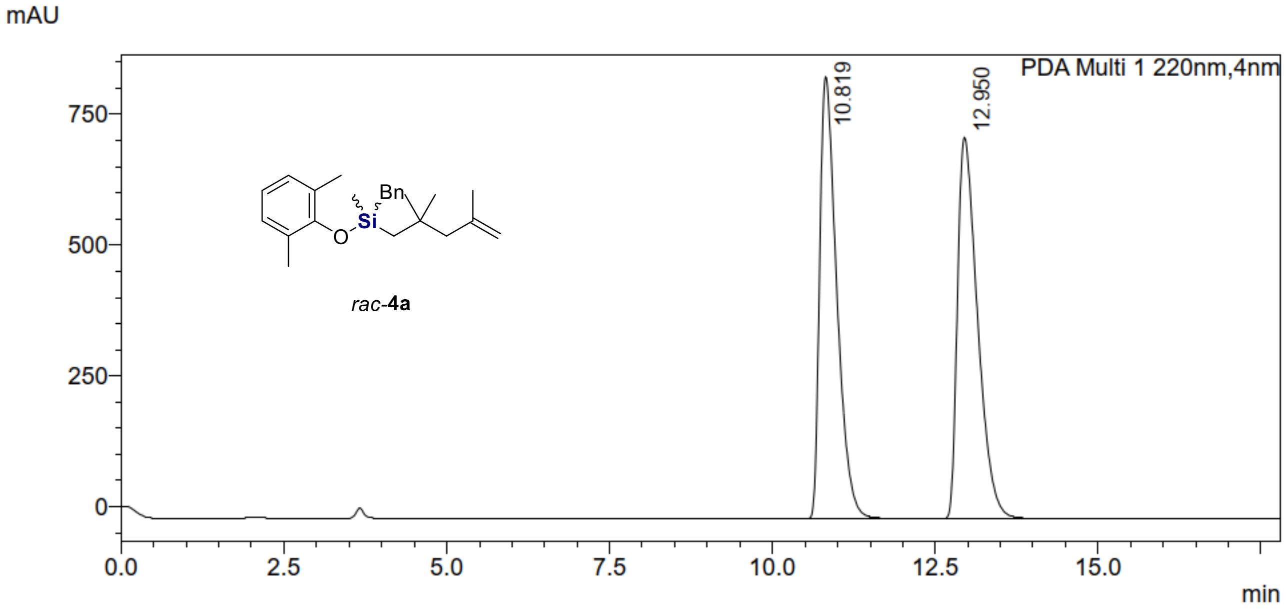

Peak #	t_R/min	% peak area
1	10.8	49.59
2	13.0	50.41
Total		100

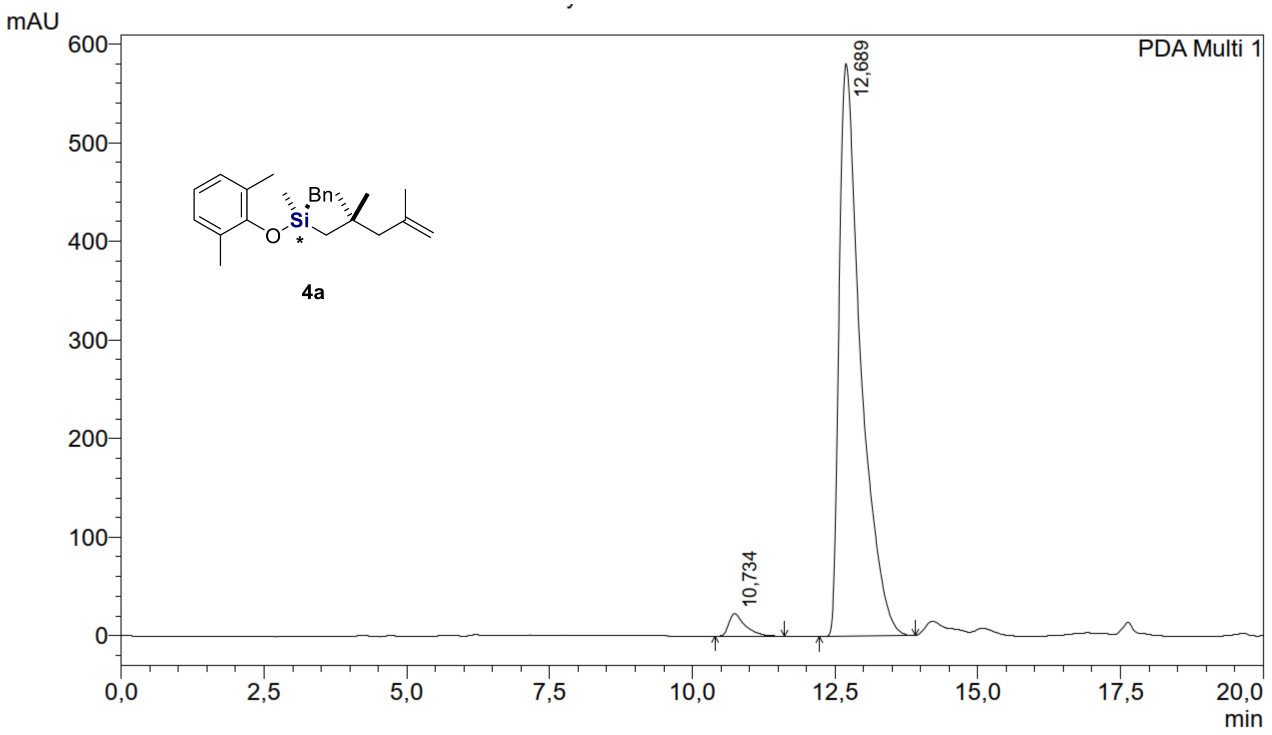

Peak #	t_R/min	% peak area
1	10.7	2.96
2	12.7	97.04
Total		100

HPLC column: OD-3, *n*-Heptane = 100, 0.5 mL/min, 298 K, 220 nm.

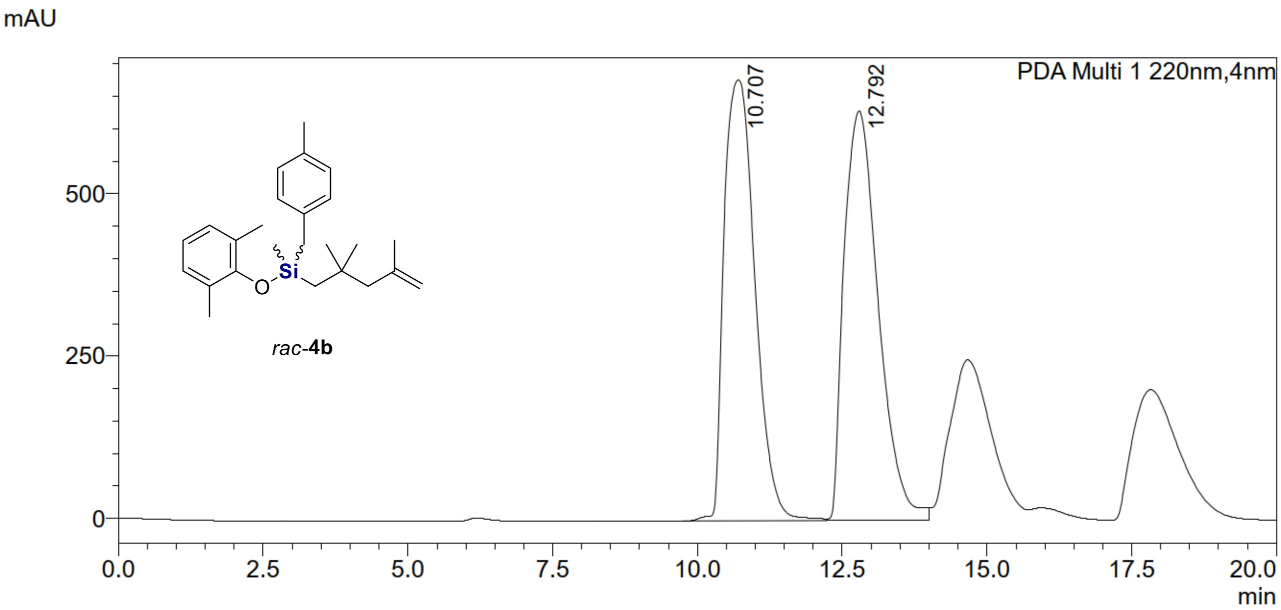

Peak #	t_R/min	% peak area
1	10.7	49.30
2	12.8	50.70
Total		100

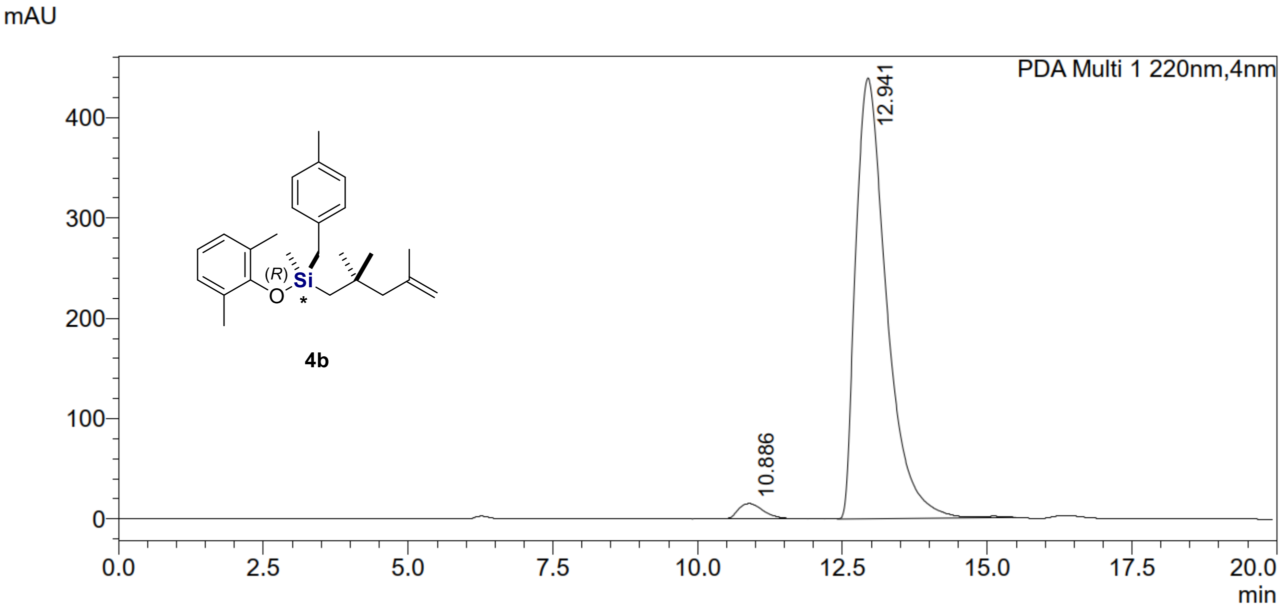

Peak #	t_R/min	% peak area
1	10.9	2.77
2	12.9	97.23
Total		100

HPLC column: IB-3, *n*-Heptane = 100, 0.5 mL/min, 298 K, 220 nm.

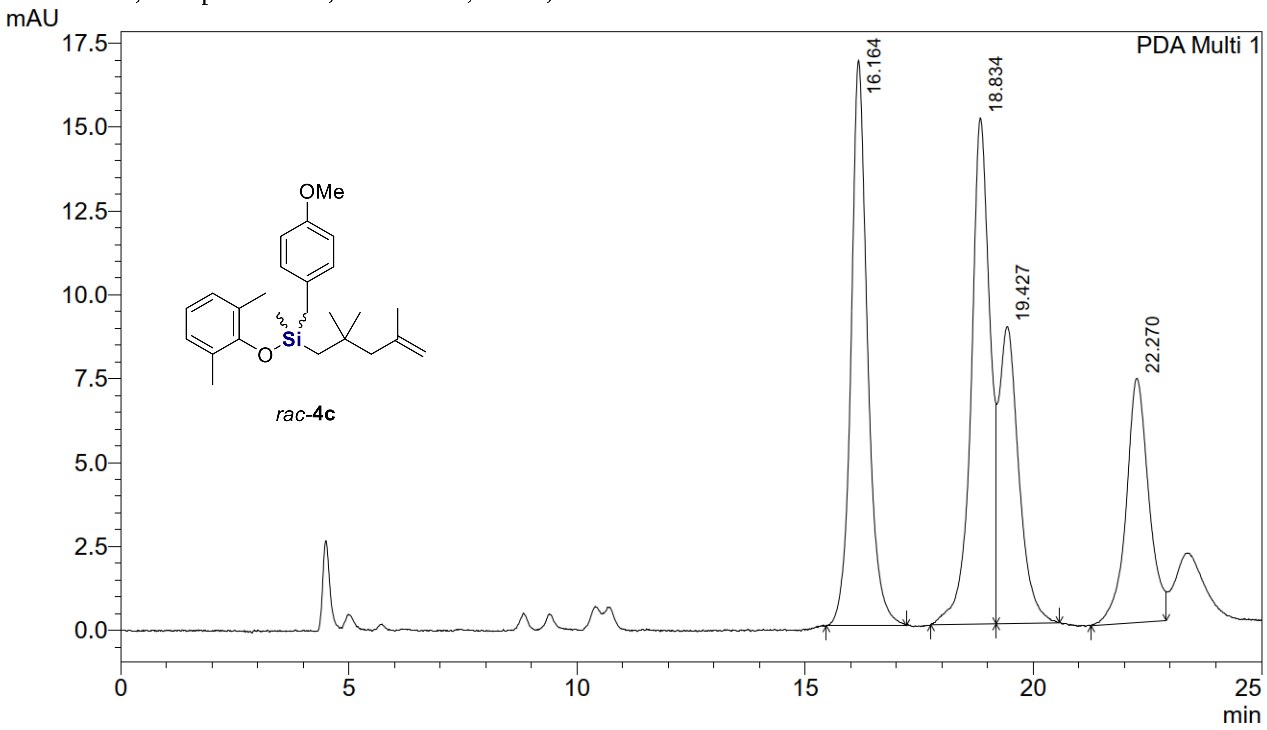

Peak #	t_R/min	% peak area
1	16.2	30.74
2	18.8	31.55
3	19.4	19.50
4	22.3	18.21
Total		100

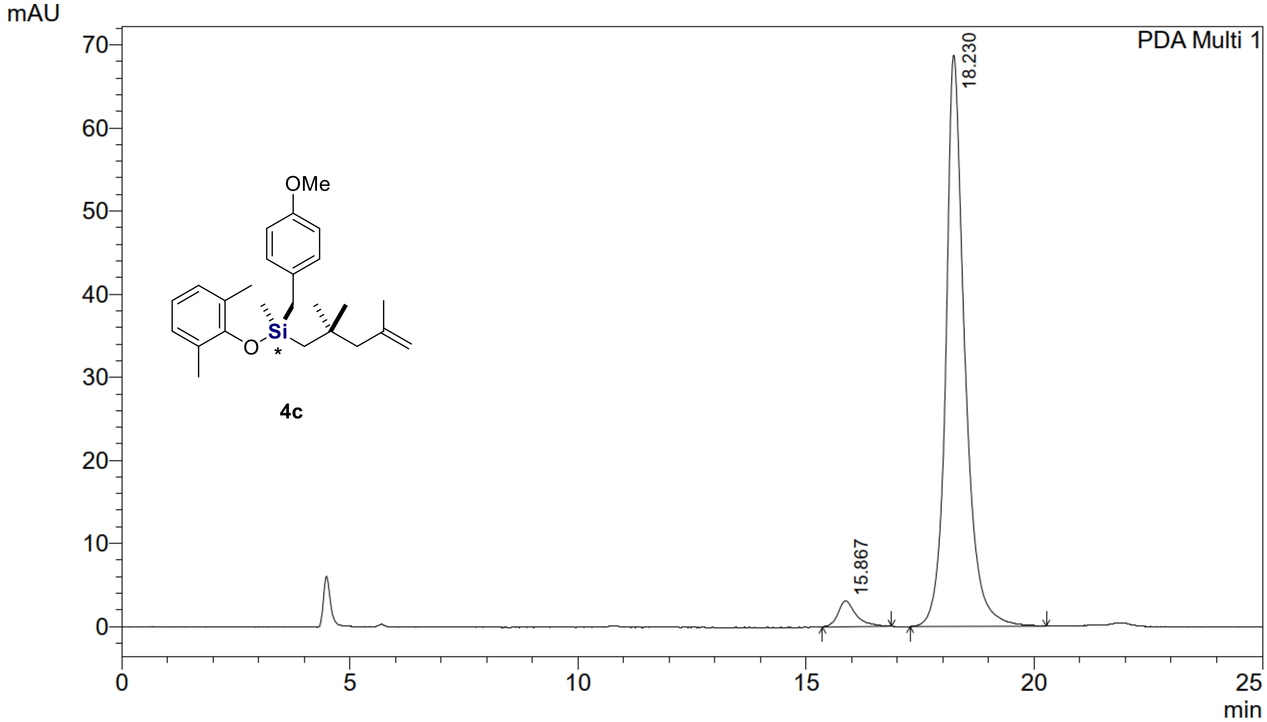

Peak #	t_R/min	% peak area
1	15.9	4.22
2	18.2	95.78

Total		100

HPLC column: OD-3, *n*-Heptane = 100, 0.5 mL/min, 298 K, 220 nm.

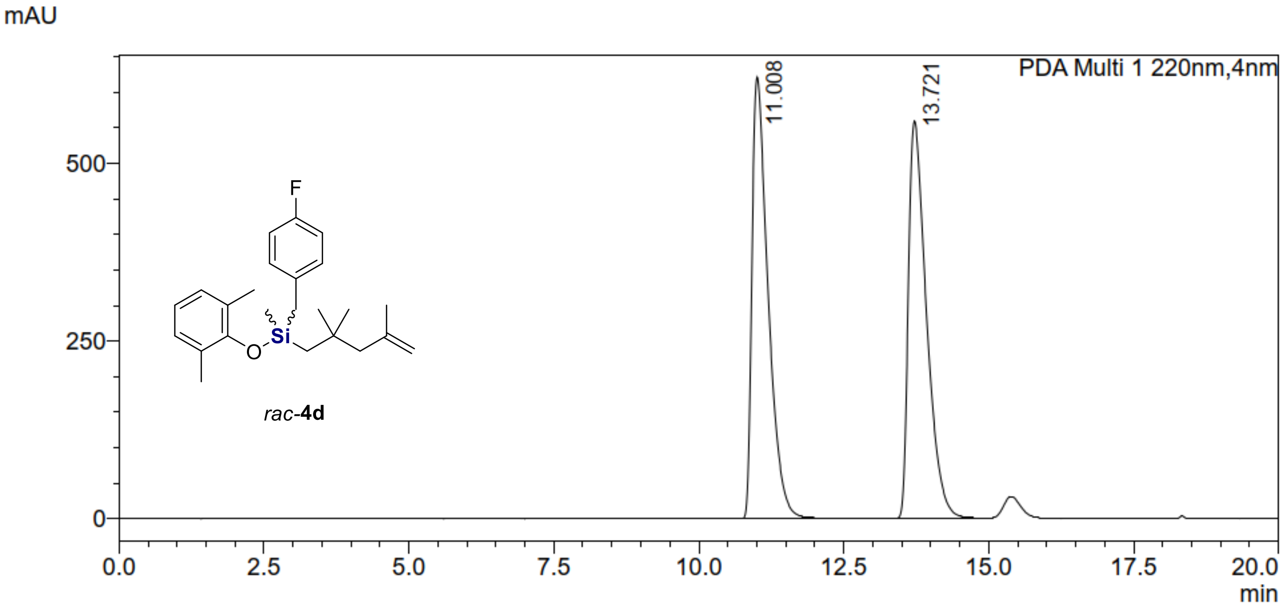

Peak #	t_R/min	% peak area
1	11.0	49.75
2	13.7	50.25
Total		100

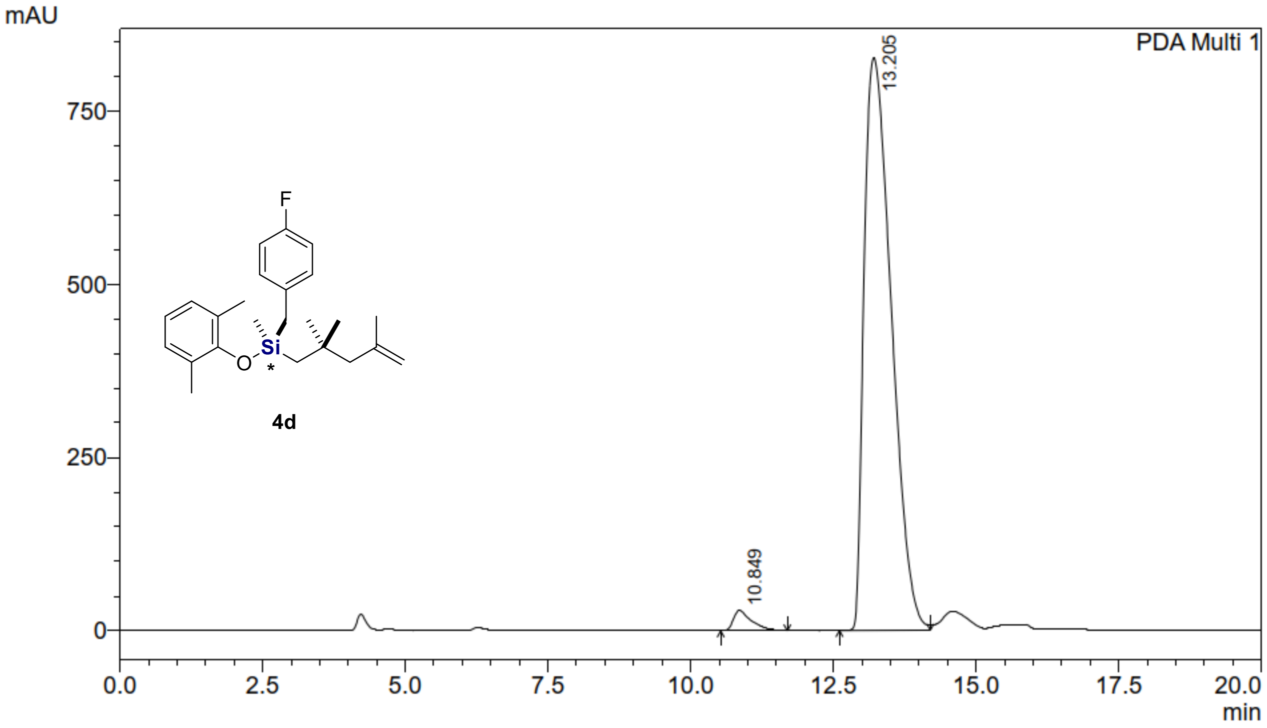

Peak #	t_R/min	% peak area
1	10.8	2.33
2	13.2	97.67
Total		100

HPLC column: OD-3, *n*-Heptane = 100, 0.5 mL/min, 298 K, 220 nm.

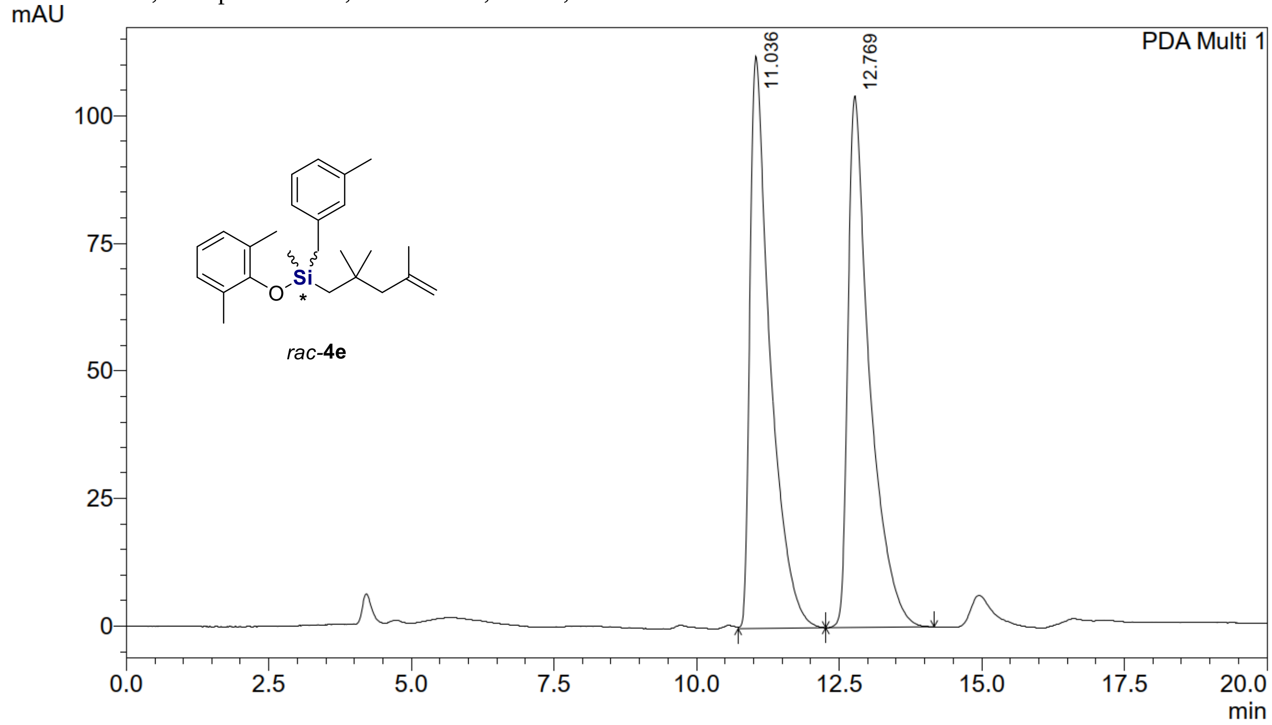

Peak #	t_R/min	% peak area
1	11.0	50.01
2	12.8	49.99
Total		100

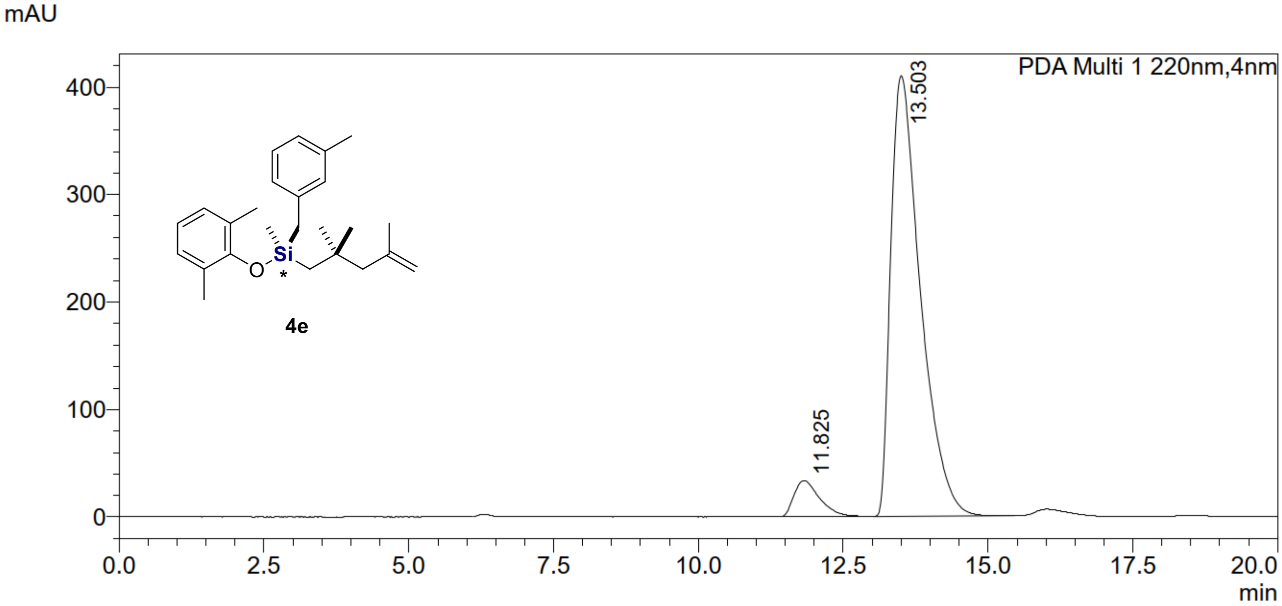

Peak #	t_R/min	% peak area
1	11.8	6.79
2	13.5	93.21
Total		100

HPLC column: IB-3N, *n*-Heptane = 100, 1.0 mL/min, 298 K, 220 nm.

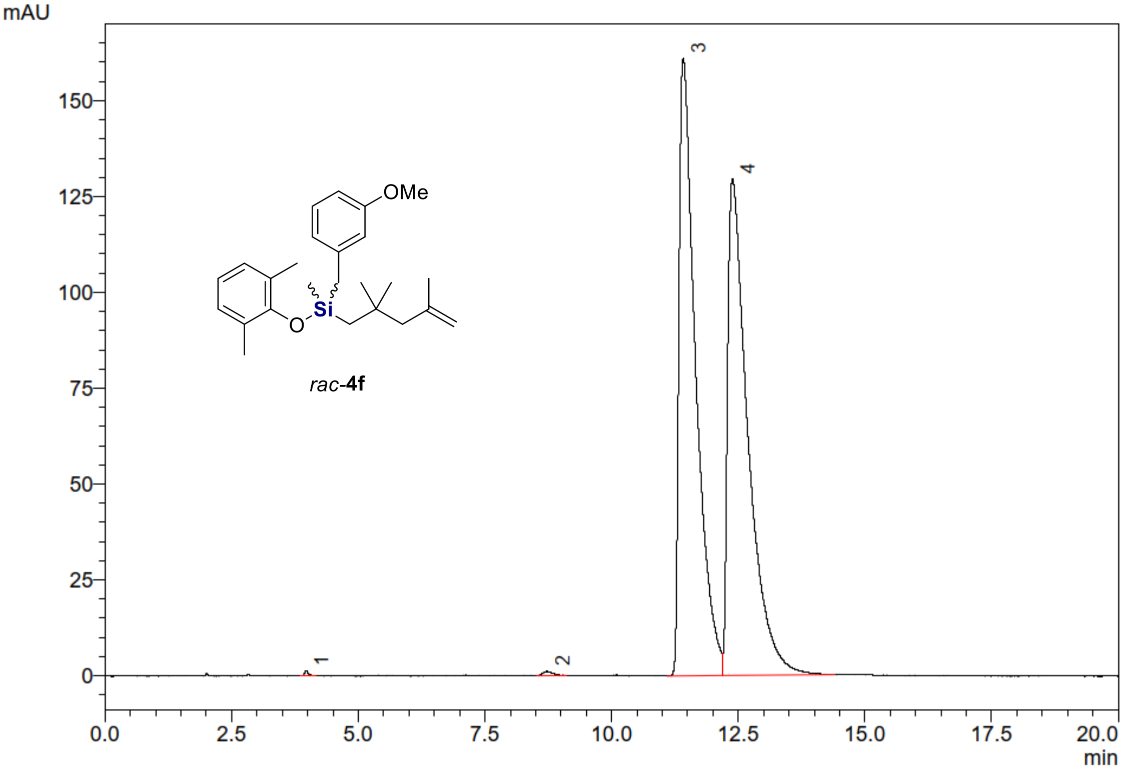

Peak #	t_R/min	% peak area
3	11.4	50.42
4	12.4	49.58
Total		100

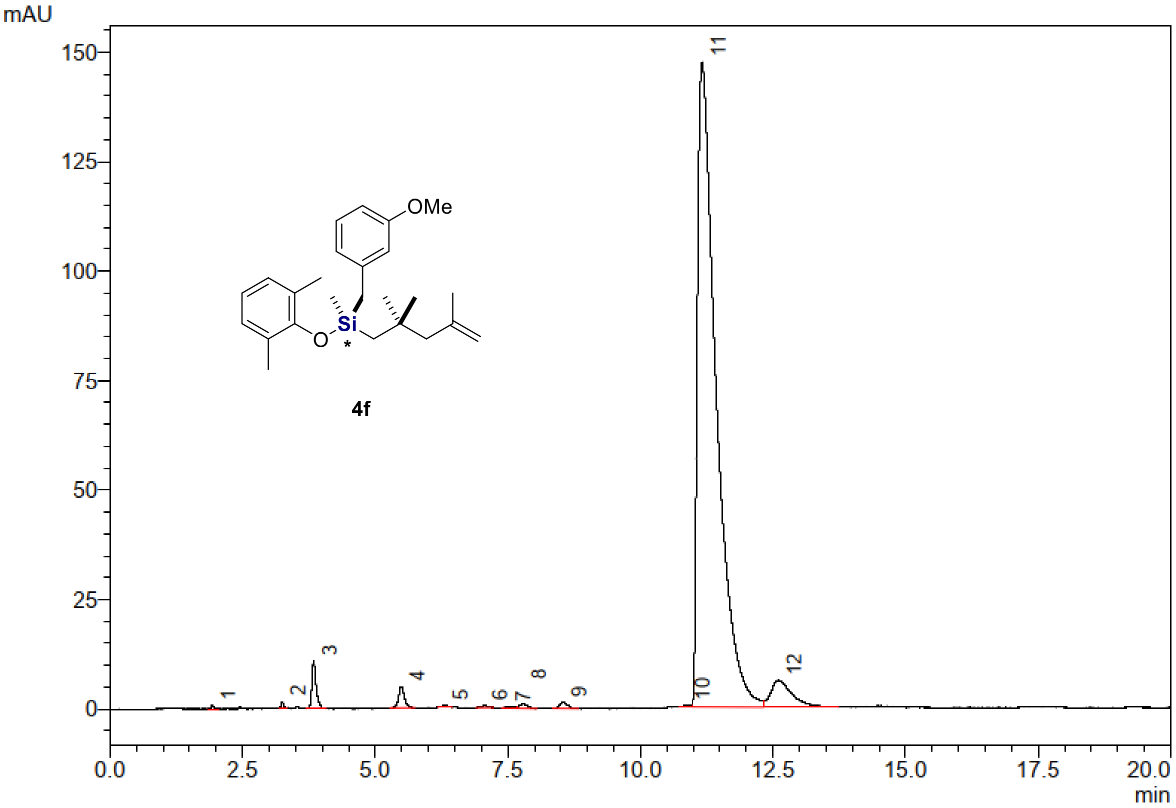

Peak #	t_R/min	% peak area
11	11.2	4.52
12	12.6	95.48
Total		100

HPLC column: OD-3, *n*-Heptane = 100, 0.5 mL/min, 298 K, 220 nm.

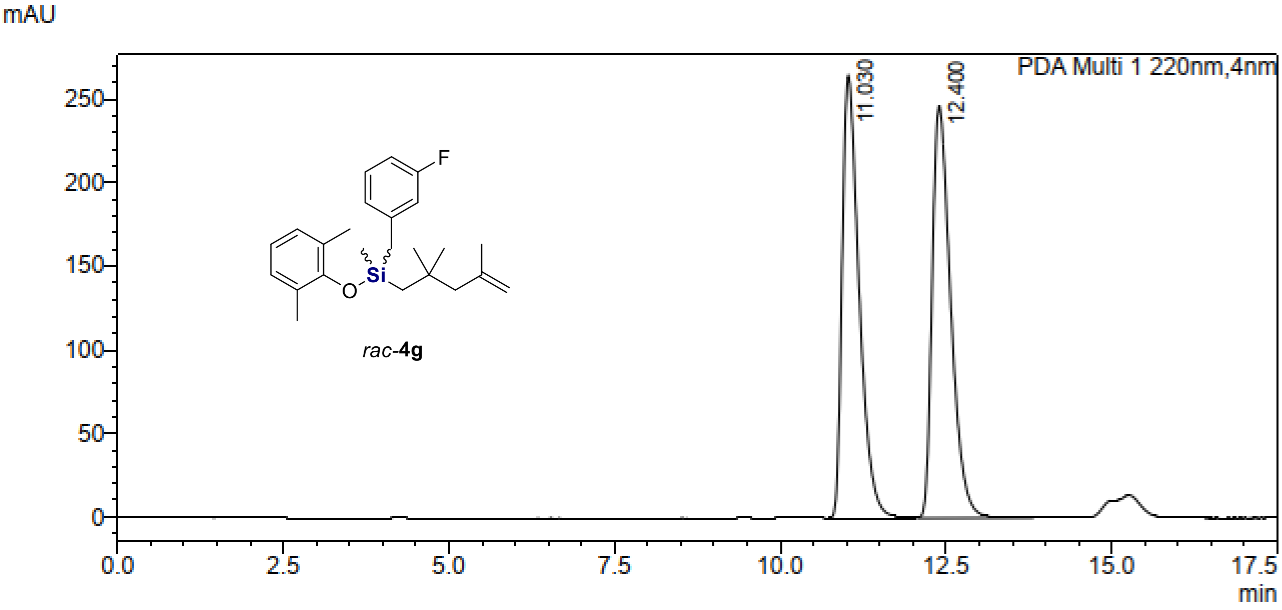

Peak #	t_R/min	% peak area
1	11.0	49.60
2	12.4	50.40
Total		100

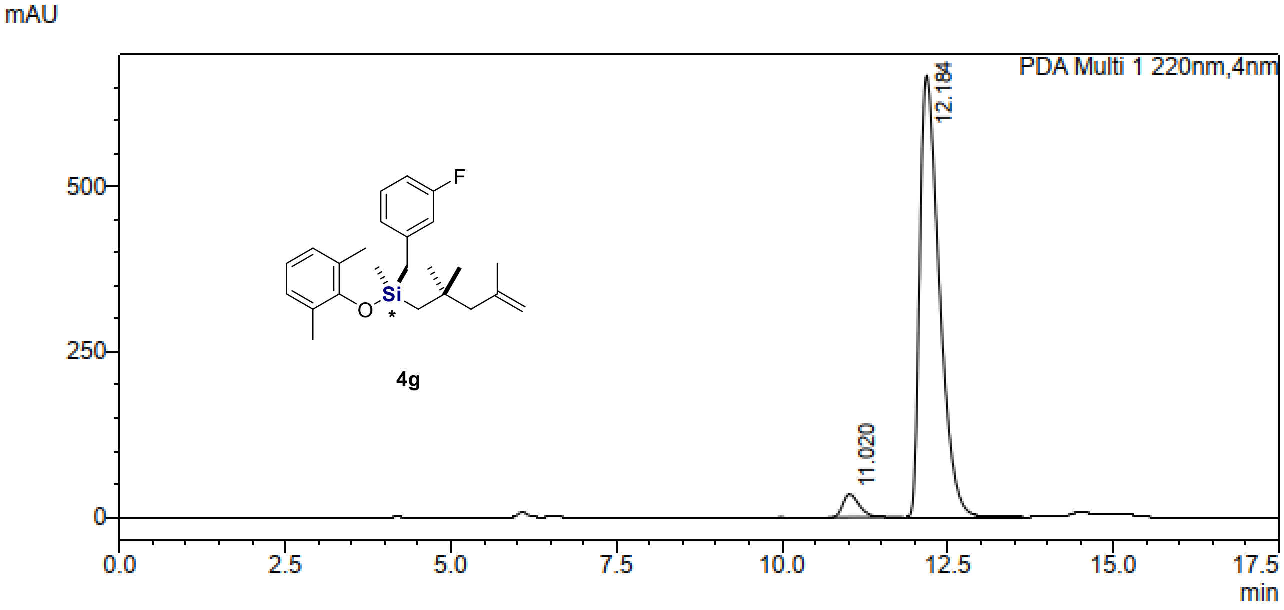

Peak #	t_R/min	% peak area
1	11.0	4.25
2	12.2	95.76
Total		100

HPLC column: OD-3, *n*-Heptane = 100, 0.5 mL/min, 298 K, 220 nm.

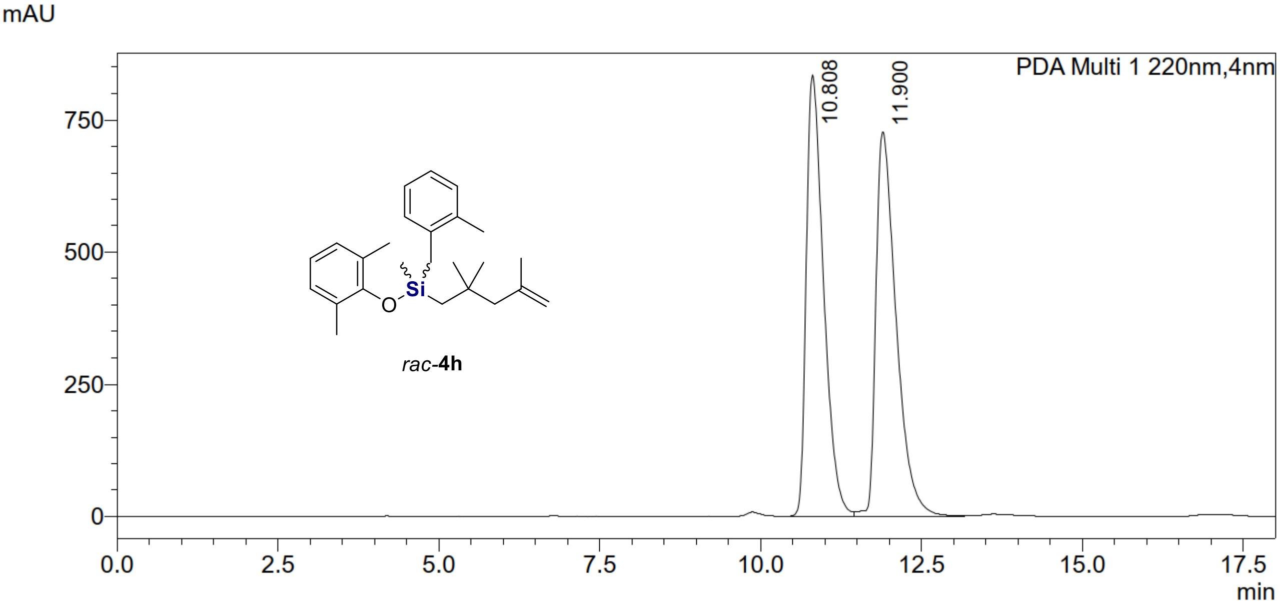

Peak #	t_R/min	% peak area
1	10.8	49.48
2	11.9	50.52
Total		100

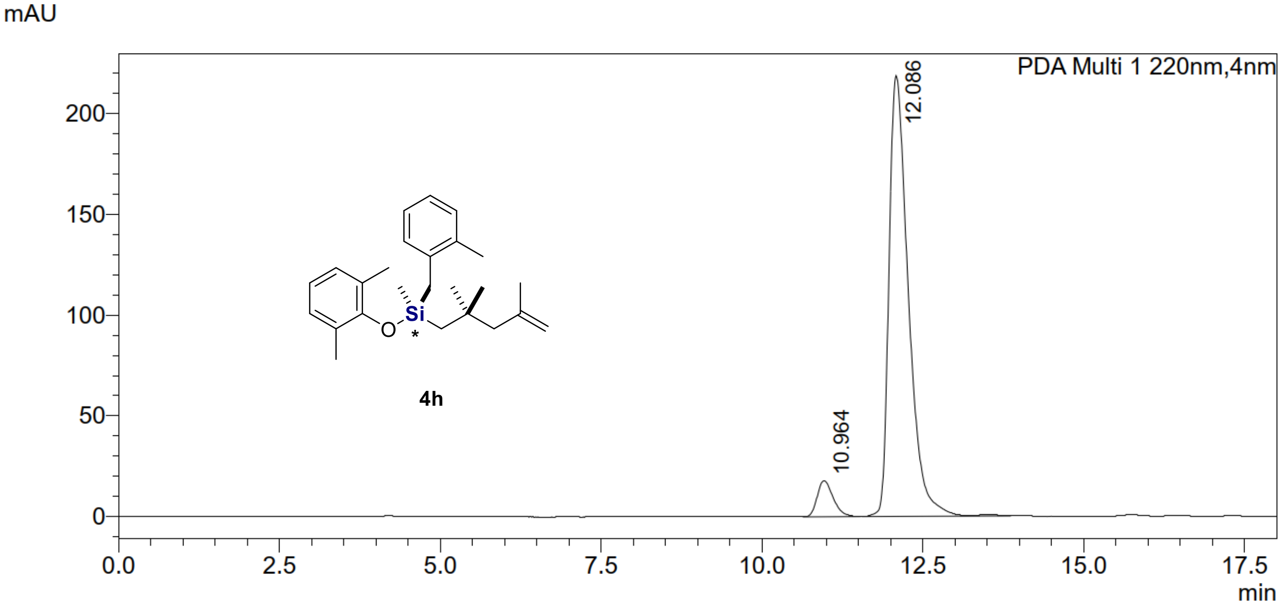

Peak #	t_R/min	% peak area
1	11.0	6.33
2	12.1	93.67
Total		100

HPLC column: OD-3, *n*-Heptane = 100, 0.5 mL/min, 298 K, 220 nm.

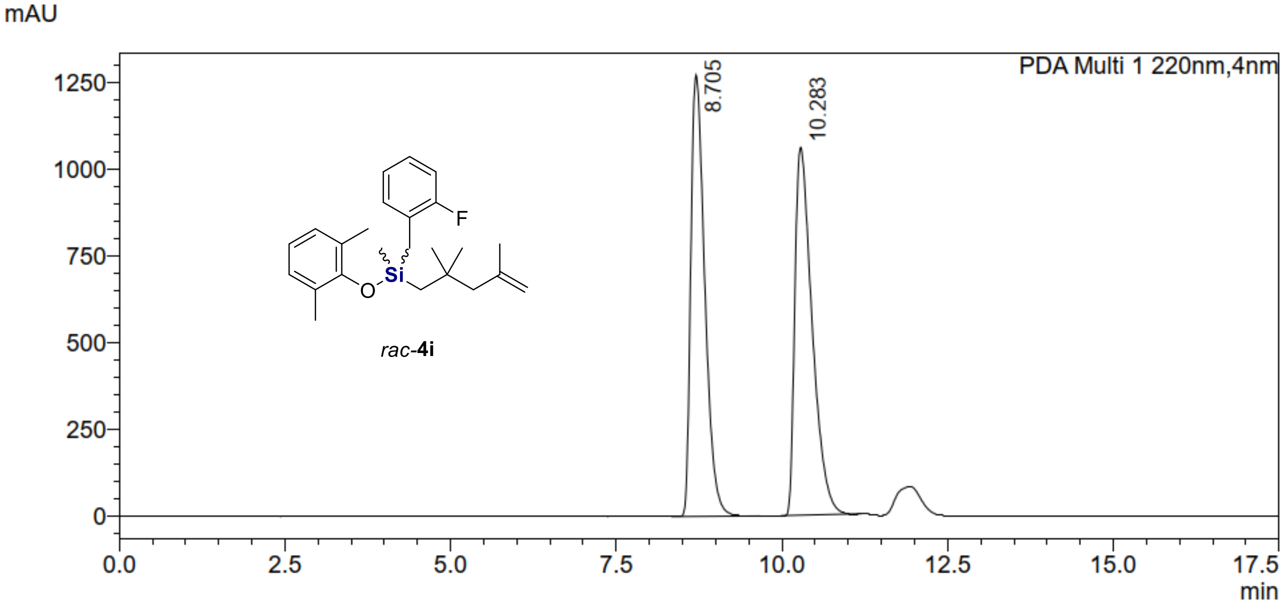

Peak #	t_R/min	% peak area
1	8.7	49.34
2	10.3	50.66
Total		100

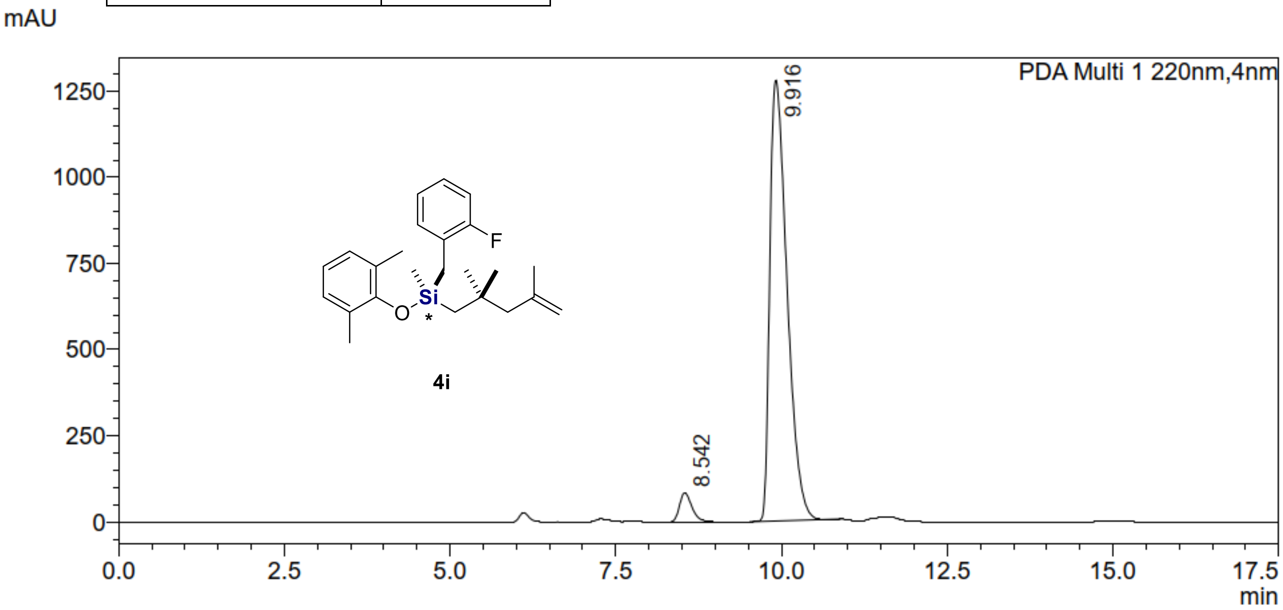

Peak #	t_R/min	% peak area
1	8.5	4.61
2	9.9	95.39
Total		100

HPLC column: OD-3, *n*-Heptane = 100, 0.5 mL/min, 298 K, 220 nm.

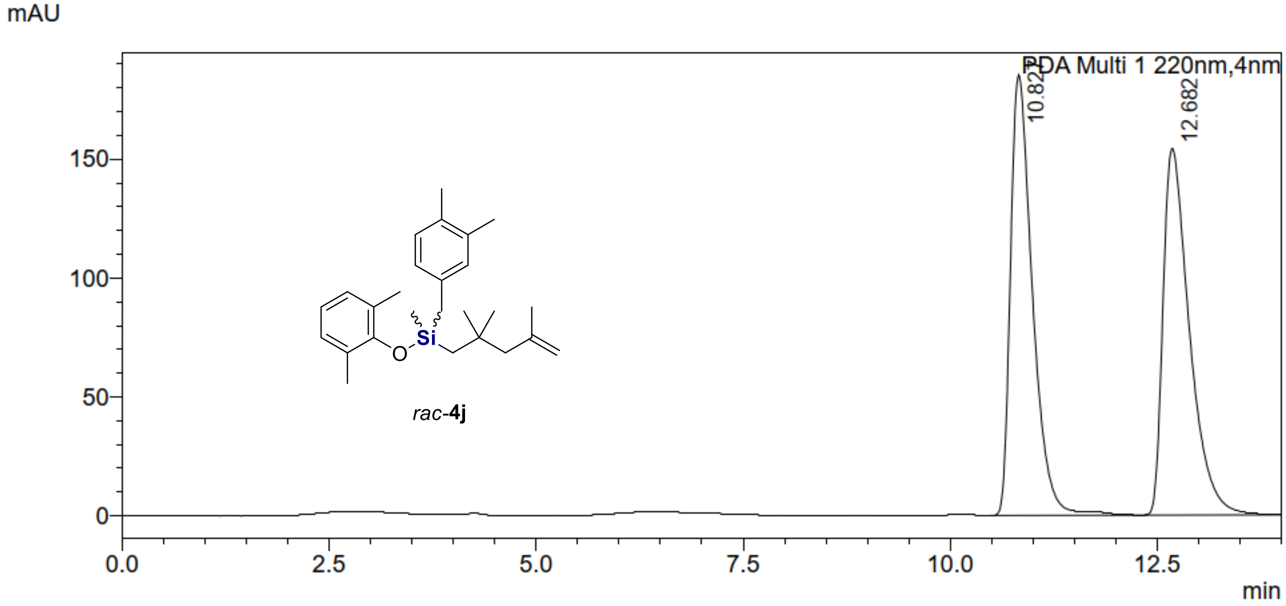

Peak #	t_R/min	% peak area
1	10.8	49.97
2	12.7	50.03
Total		100

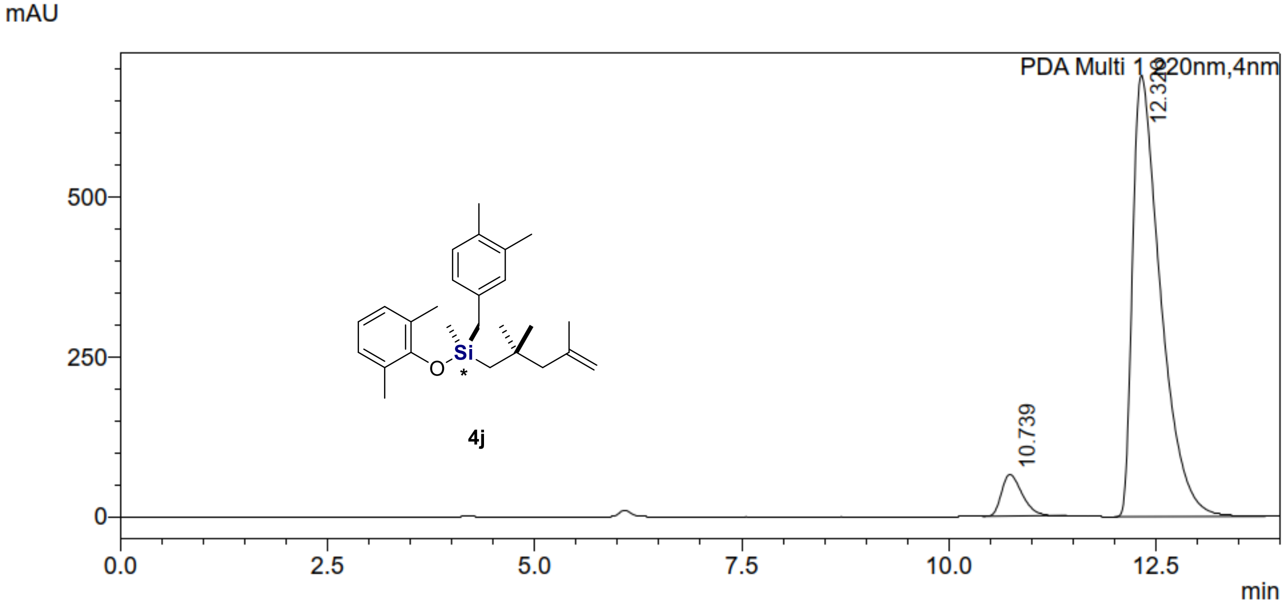

Peak #	t_R/min	% peak area
1	10.7	6.52
2	12.3	93.48
Total		100

HPLC column: IB-3, *n*-Heptane = 100, 0.5 mL/min, 298 K, 220 nm.

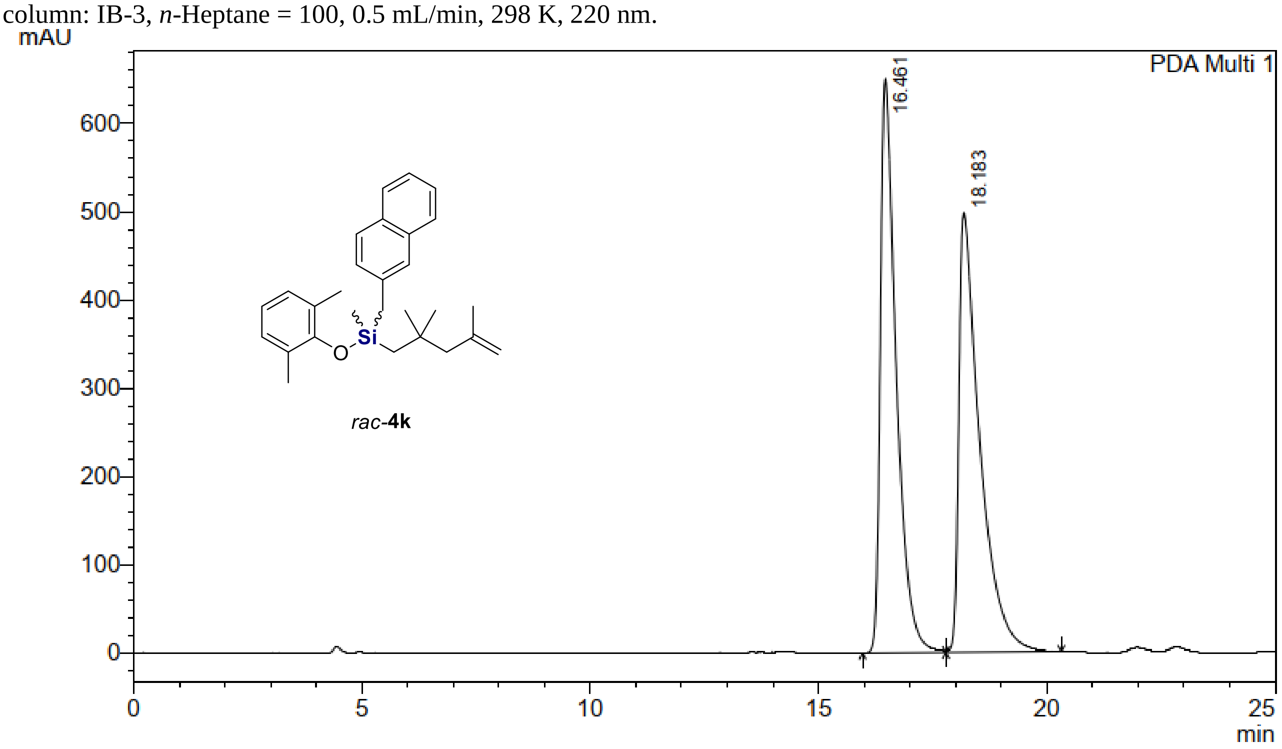

Peak #	t_R/min	% peak area
1	16.5	49.88
2	18.2	50.12
Total		100

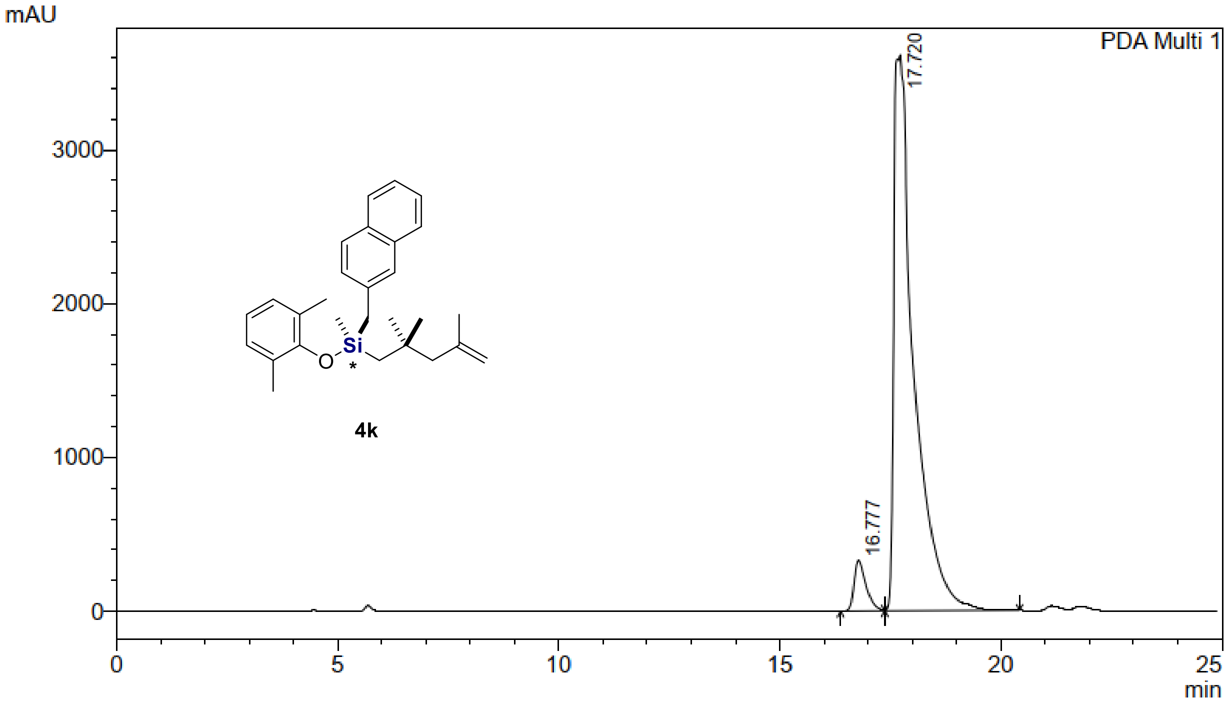

Peak #	t_R/min	% peak area
1	16.8	5.21
2	17.7	94.79
Total		100

HPLC column: OJ-3R, MeOH/Water = 80:20, 1.0 mL/min, 298 K, 190 nm

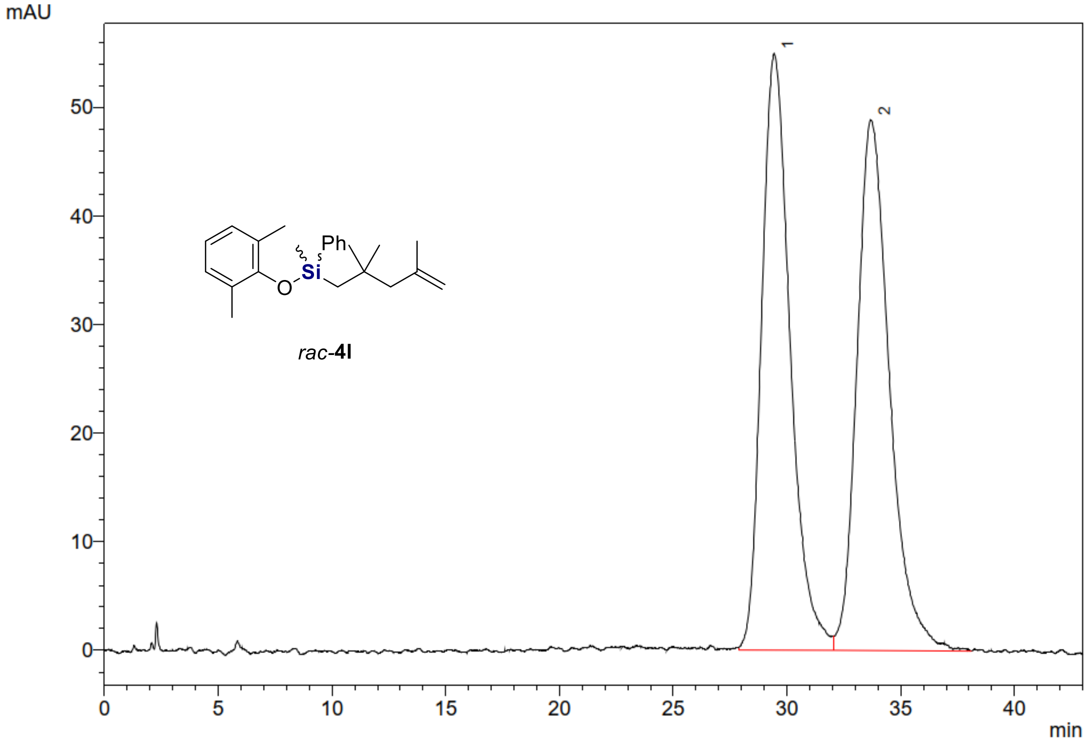

Peak #	t_R/min	% peak area
1	29.4	49.70
2	33.7	50.30
Total		100

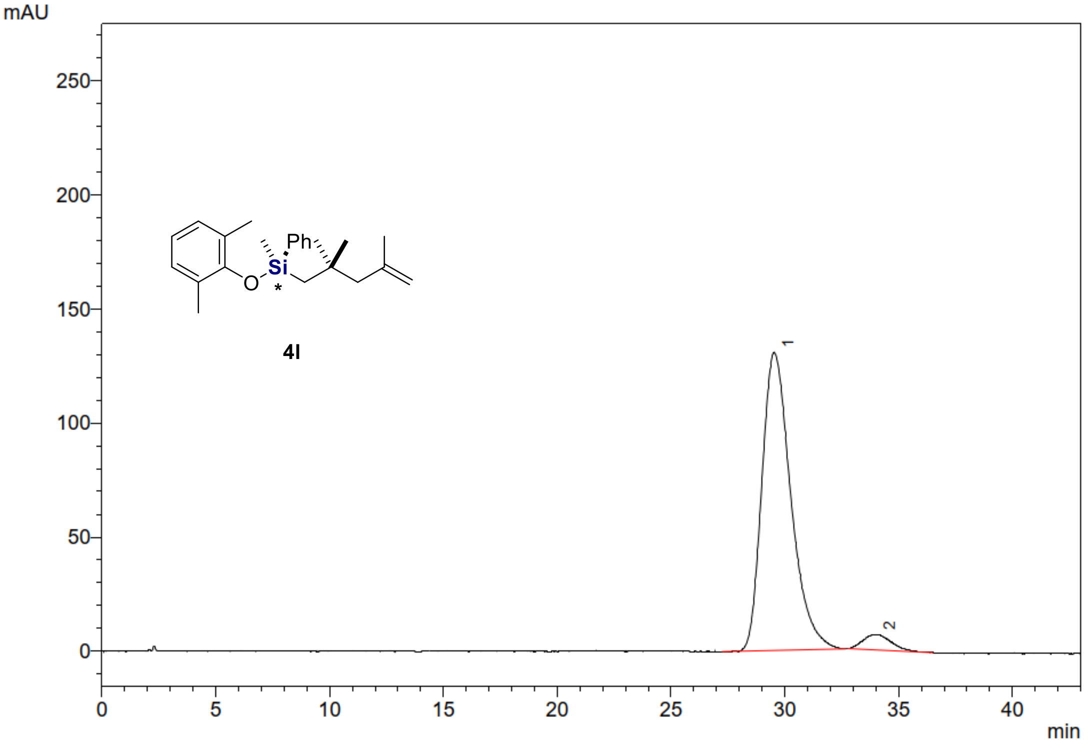

Peak #	t_R/min	% peak area
1	29.5	95.26
2	34.0	4.74
Total		100

HPLC column: OJ-3R, MeOH/Water = 80:20, 1.0 mL/min, 298 K, 220 nm

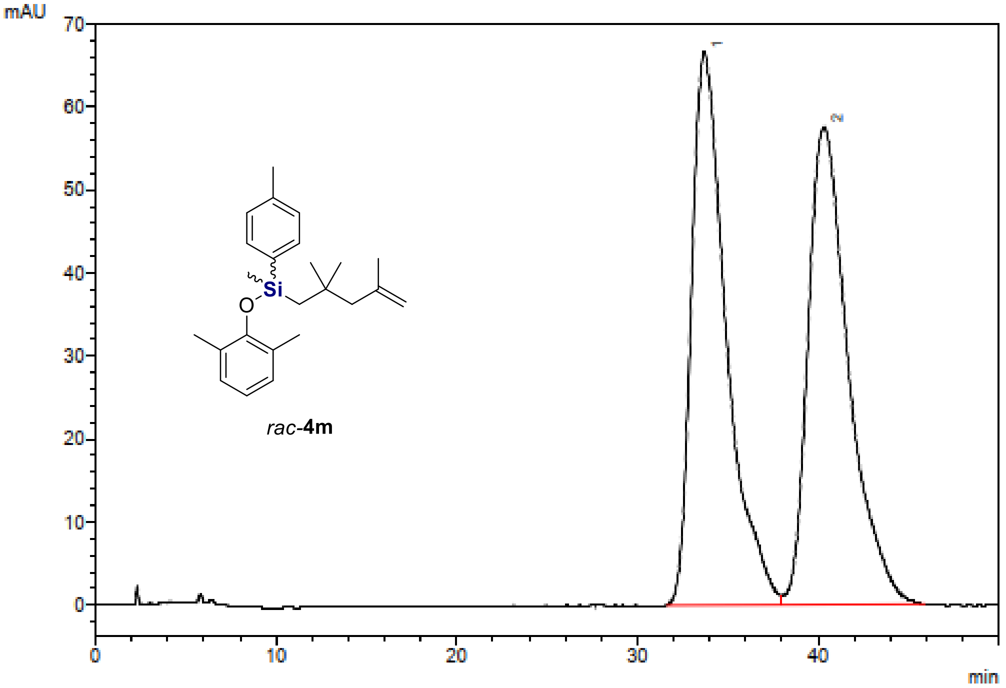

Peak #	t_R/min	% peak area
1	33.7	49.97
2	40.4	50.03
Total		100

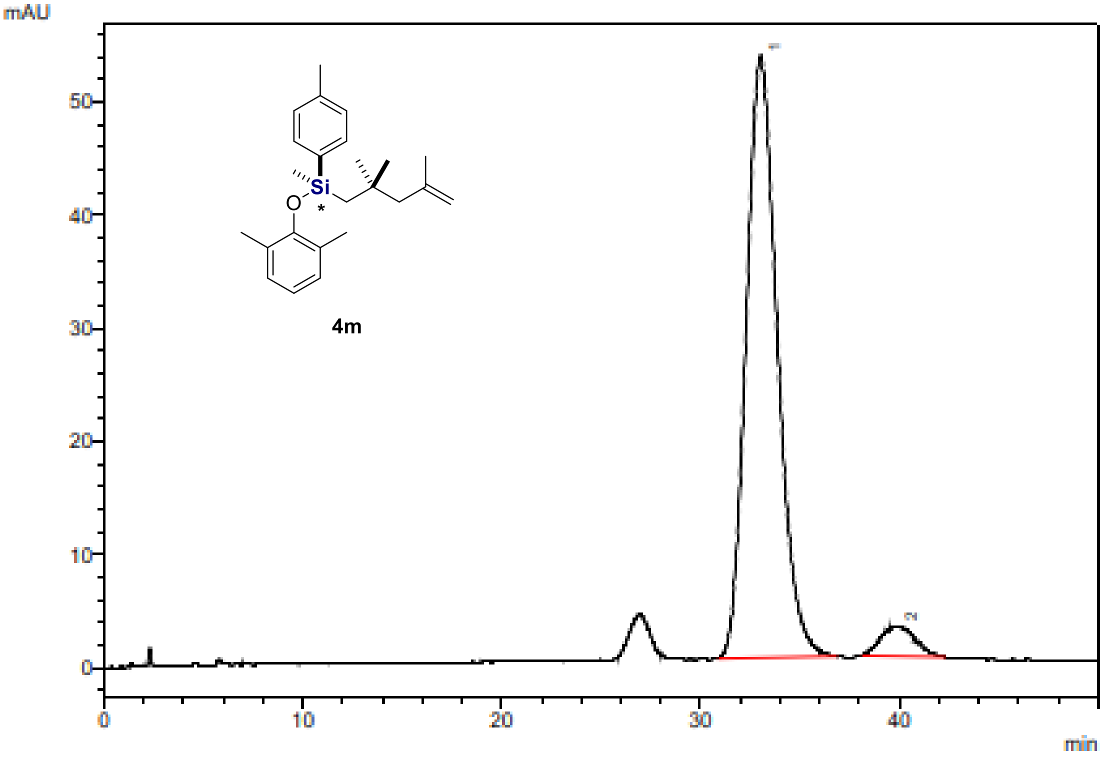

Peak #	t_R/min	% peak area
1	33.0	94.79
2	39.9	5.21
Total		100

HPLC column: OJ-3R, MeOH/Water = 80:20, 1.0 mL/min, 298 K, 220 nm

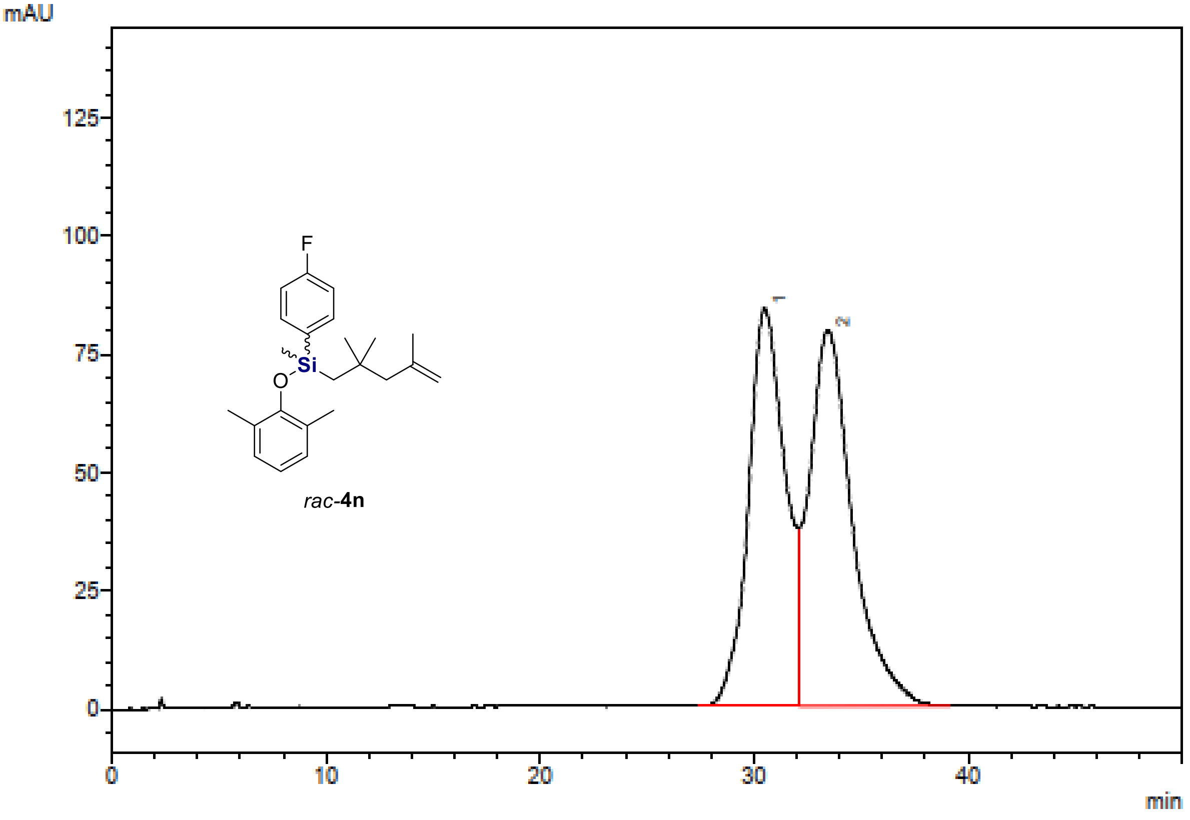

Peak #	t_R/min	% peak area
1	30.5	47.23
2	33.4	52.77
Total		100

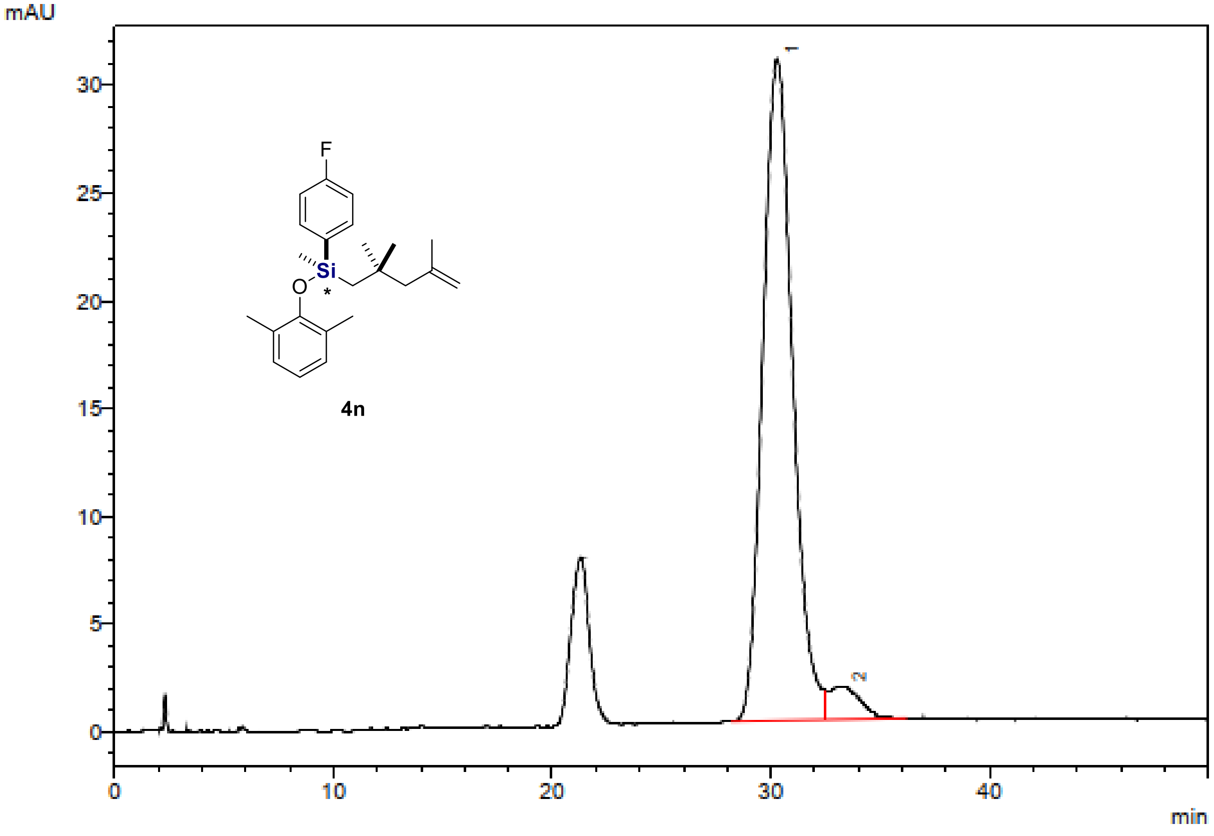

Peak #	t_R/min	% peak area
1	30.3	94.96
2	33.4	5.04
Total		100

HPLC column: OD-3, *n*-Heptane = 100, 0.5 mL/min, 298 K, 220 nm.

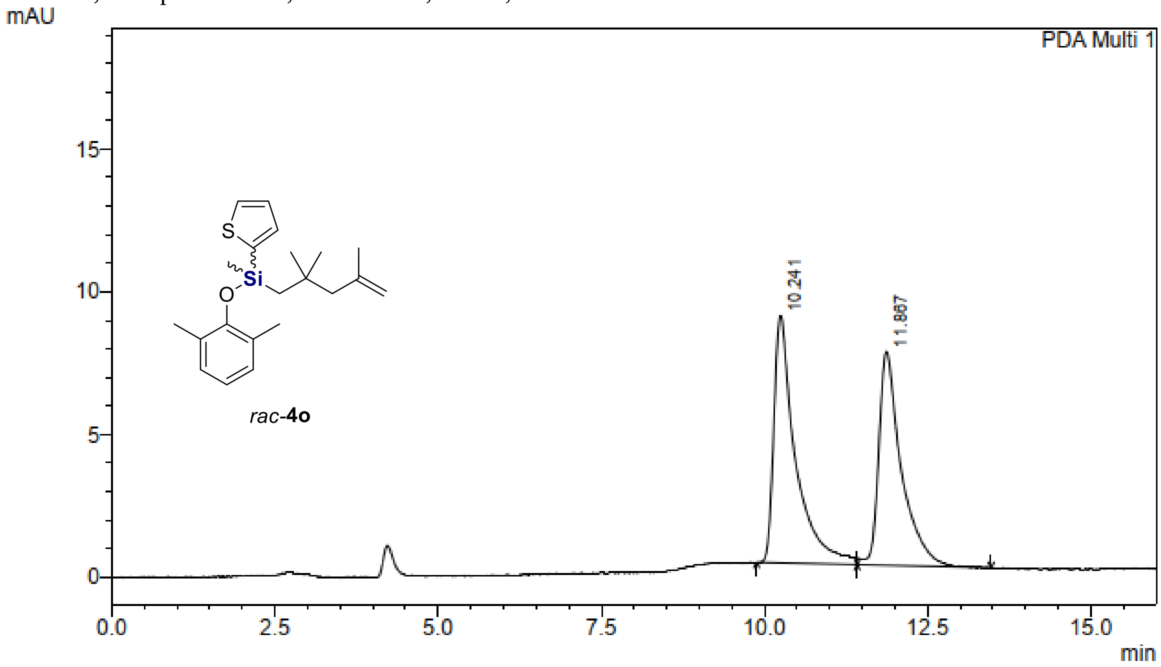

Peak #	t_R/min	% peak area
1	10.2	52.72
2	11.9	47.28
Total		100

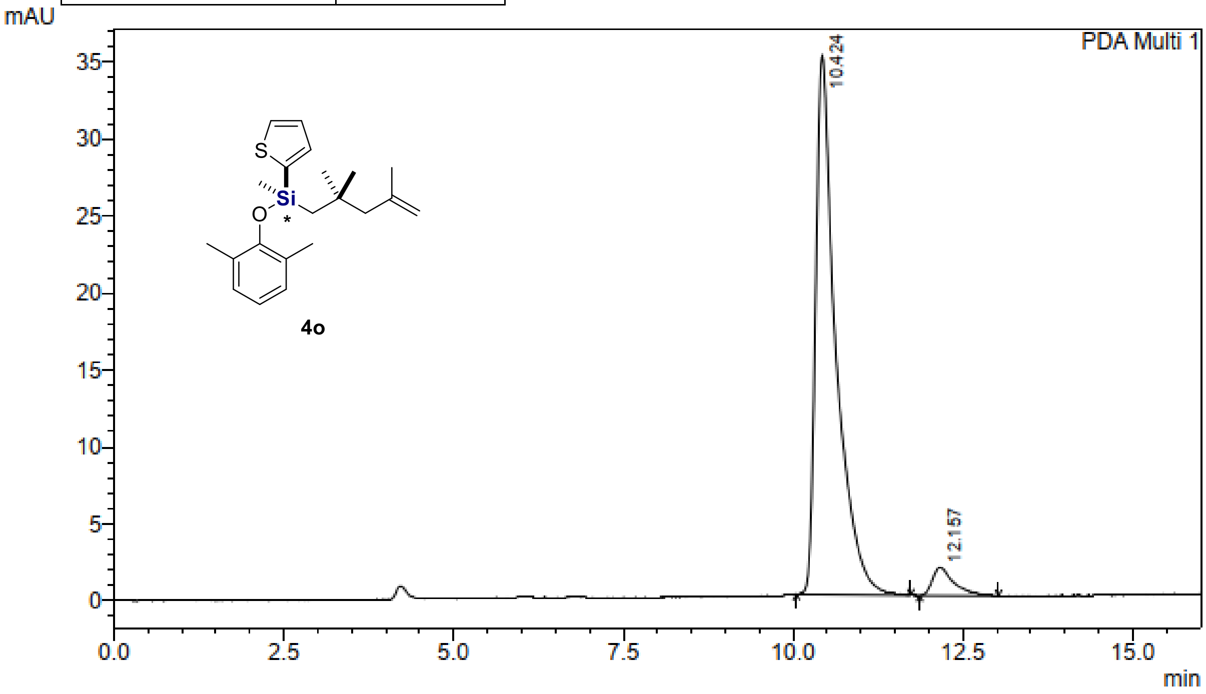

Peak #	t_R/min	% peak area
1	10.4	94.74
2	12.2	5.26
Total		100

HPLC column: OD-3, *n*-Heptane = 100, 0.5 mL/min, 298 K, 220 nm

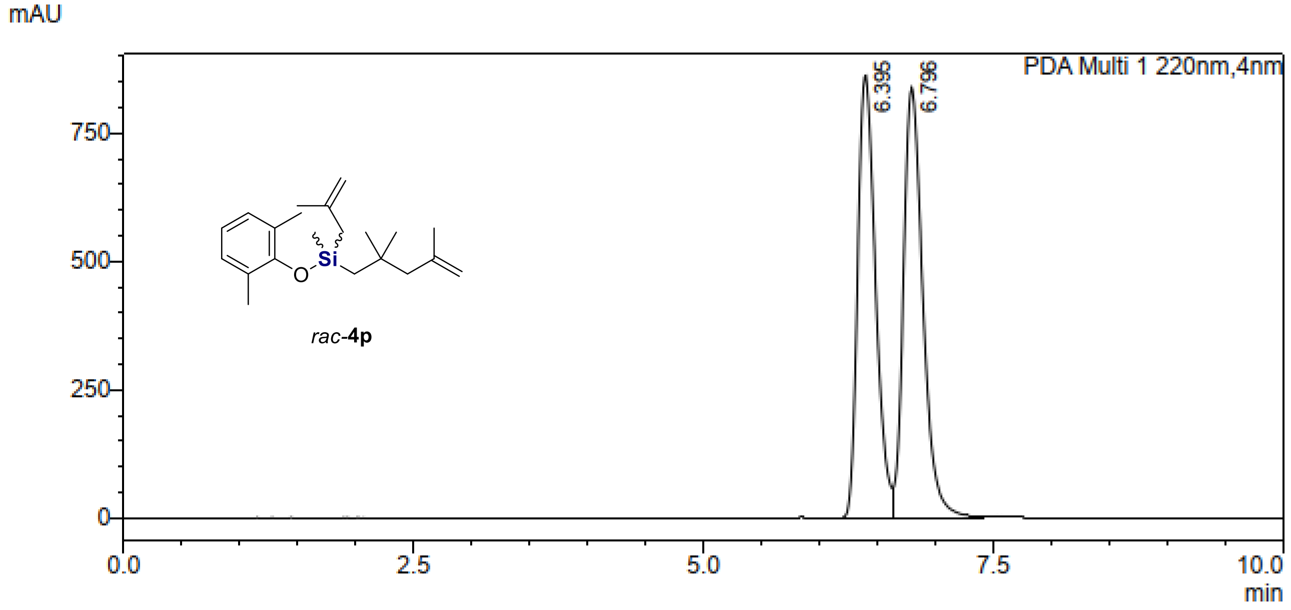

Peak #	t_R/min	% peak area
1	6.4	48.68
2	6.8	51.32
Total		100

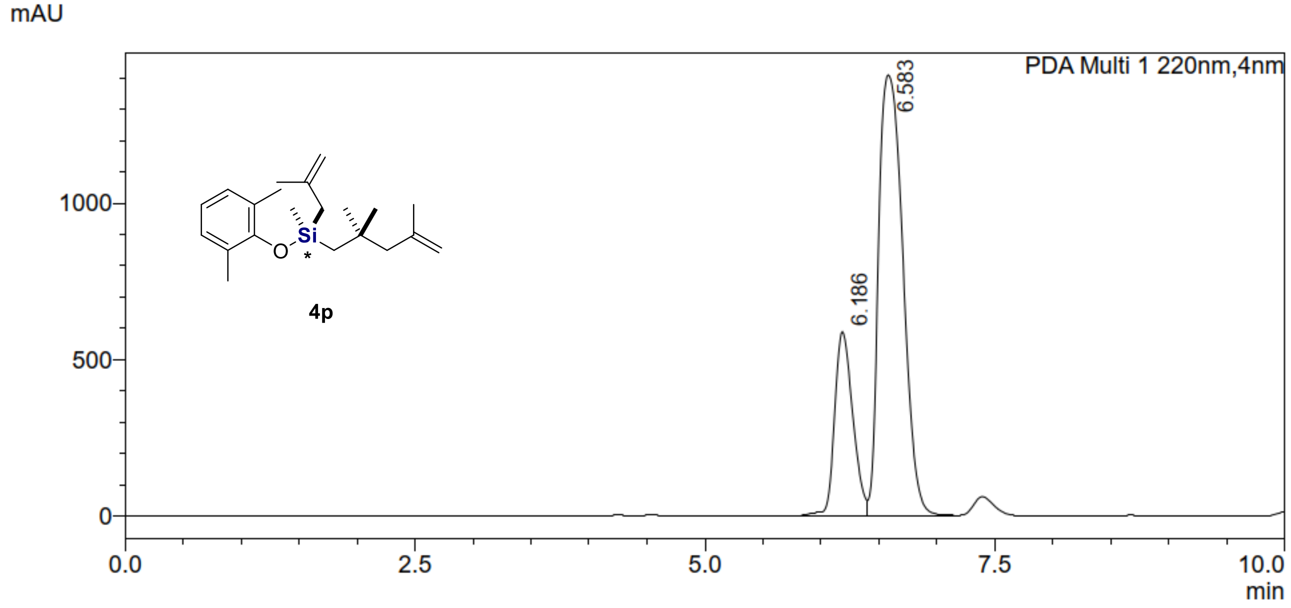

Peak #	t_R/min	% peak area
1	6.2	23.95
2	6.6	76.05
Total		100

HPLC column: OD-3, *n*-Heptane = 100, 1.0 mL/min, 293 K, 220 nm

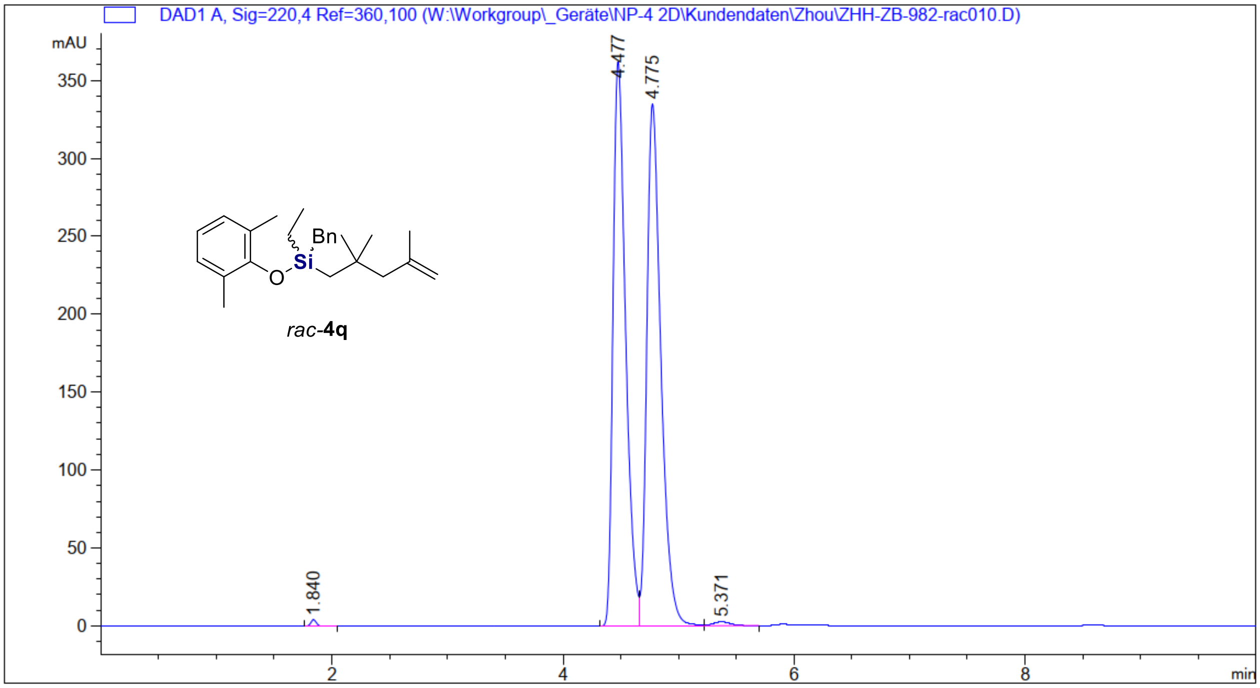

Peak #	t_R/min	% peak area
1	4.5	49.12
2	4.8	50.88
Total		100

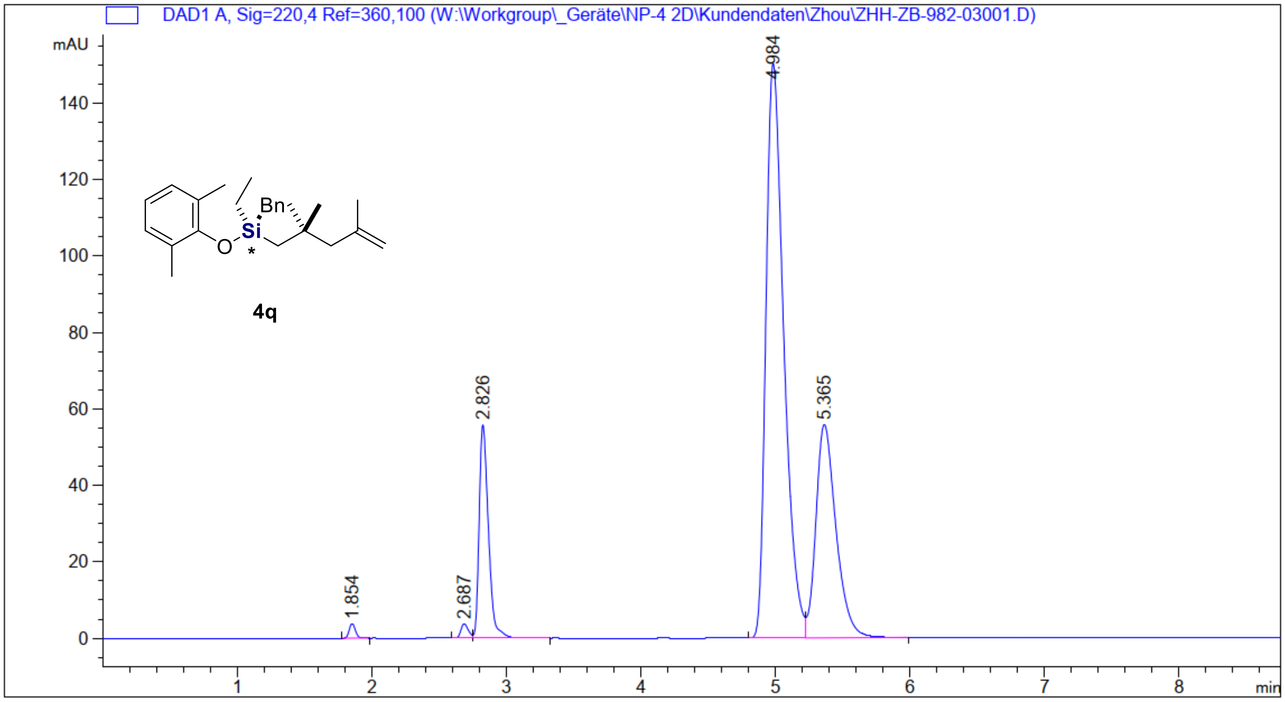

Peak #	t_R/min	% peak area
1	5.0	70.5
2	5.4	29.5
Total		100

HPLC column: AD-3, *n*-Heptane:*i*-PrOH = 99.5:0.5, 1.0 mL/min, 298 K, 224 nm.

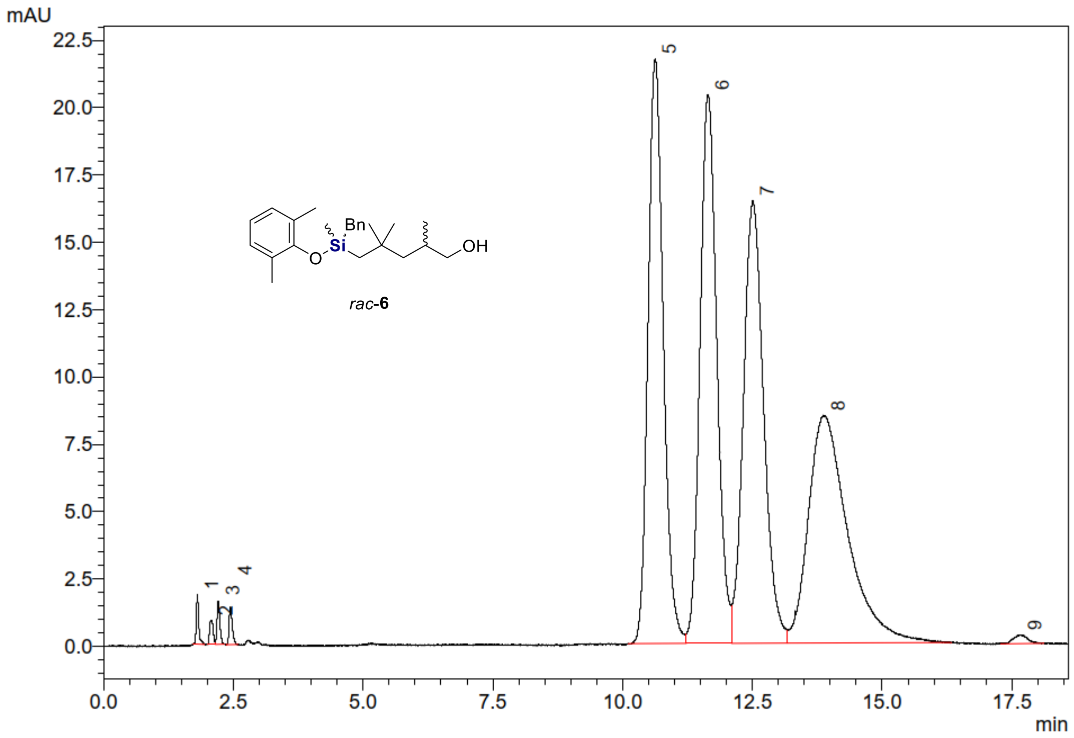

Peak #	t_R/min	% peak area
5	10.6	24.78
6	11.6	25.52
7	12.5	24.80
8	13.9	24.90
Total		100

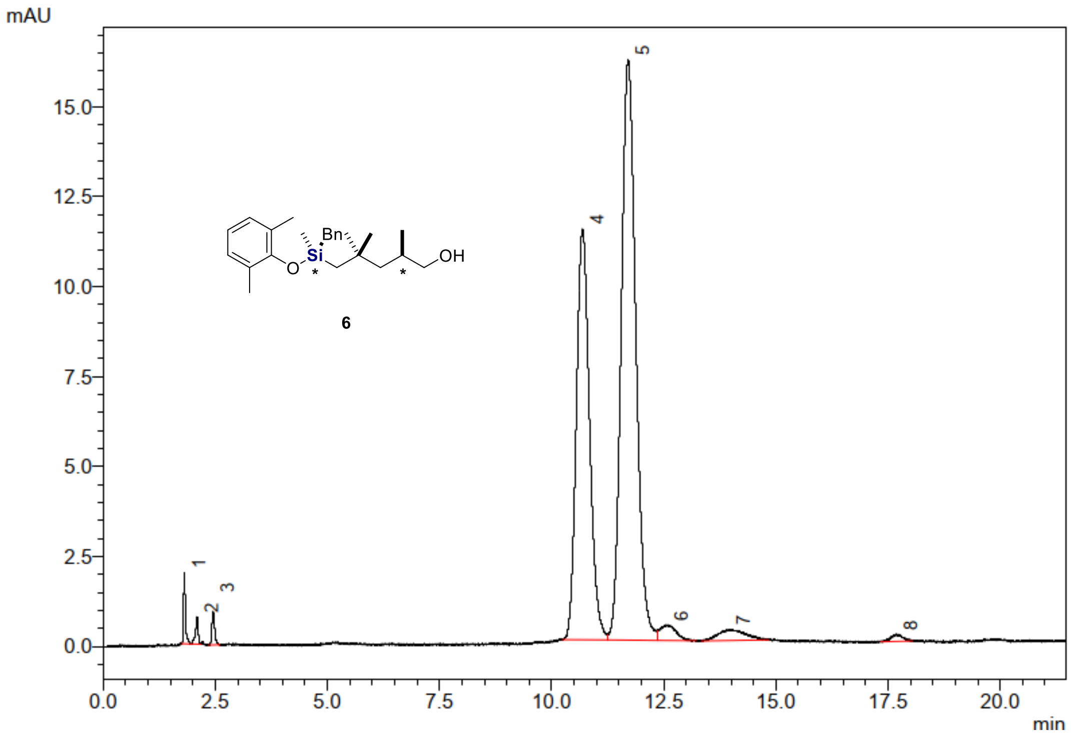

Peak #	t_R/min	% peak area
4	10.7	37.44

5	11.7	58.79
6	12.6	1.71
7	14.0	2.06
Total		100

HPLC column: IB-3, *n*-heptane, 1.0 mL/min, 298 K, 220 nm.

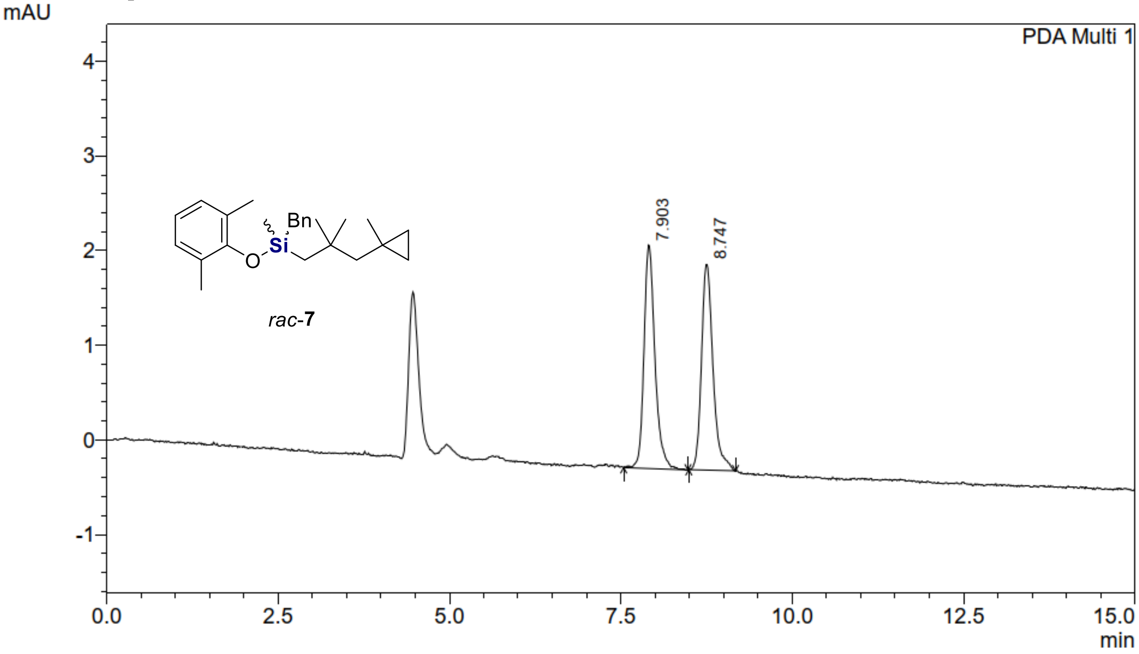

Peak #	t_R/min	% peak area
1	7.9	50.76
2	8.7	49.24
Total		100

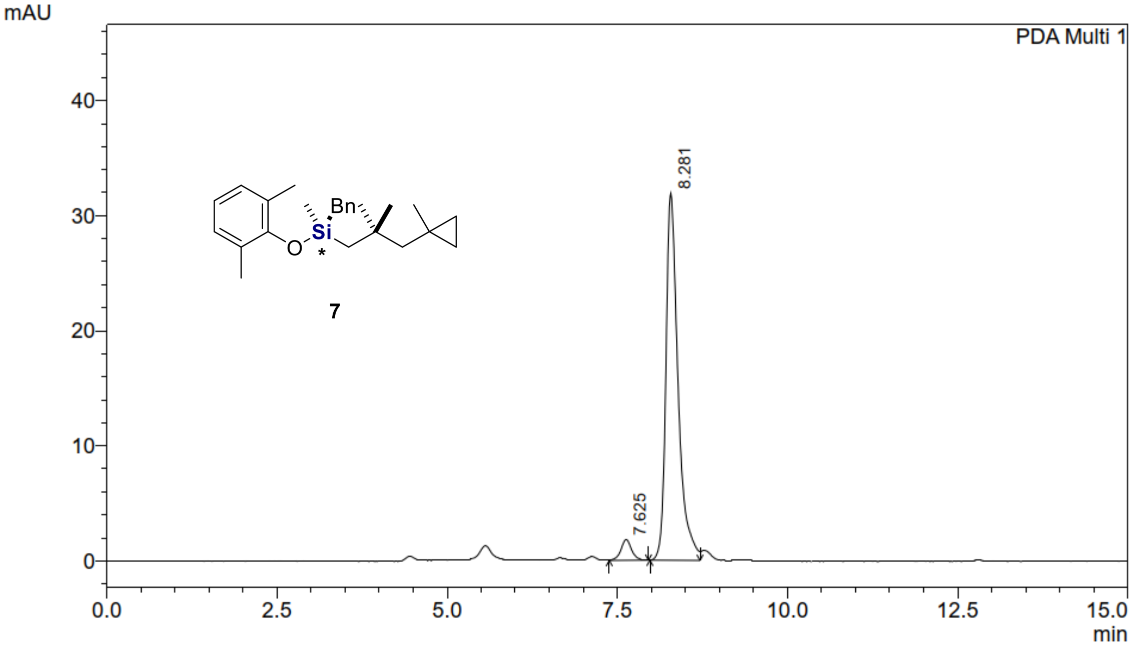

Peak #	t_R/min	% peak area
1	7.6	4.81
2	8.3	95.19
Total		100

SFC column: OJ-3, CO_2:*i*-PrOH = 98:2, 1.0 mL/min, 313 K, 220 nm.

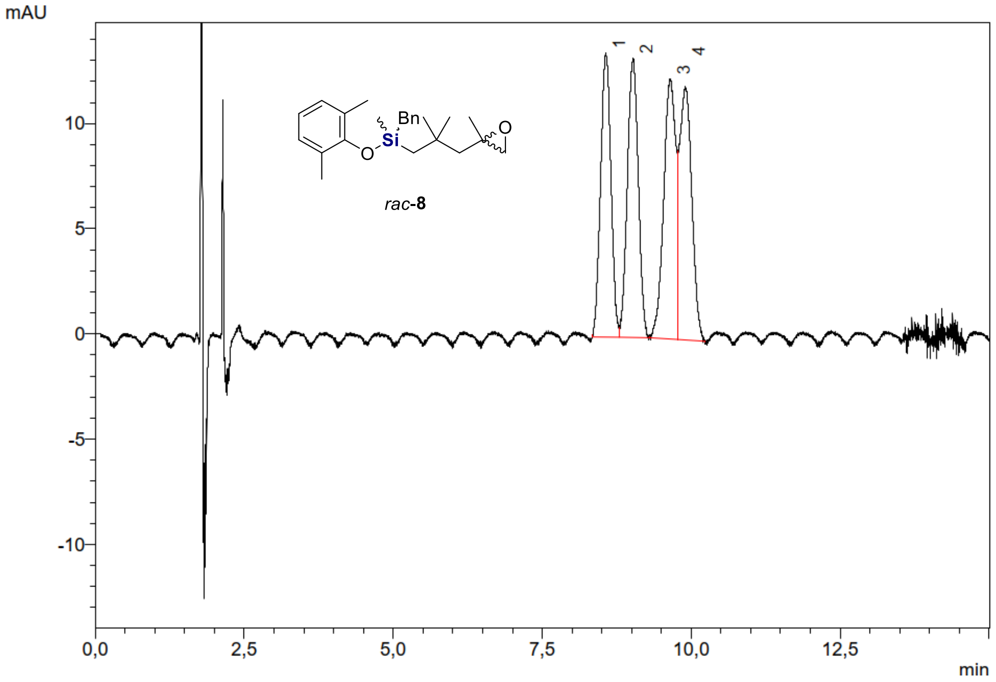

Peak #	t_R/min	% peak area
1	8.6	23.98
2	9.0	24.97
3	9.7	25.68
4	9.9	25.37
Total		100

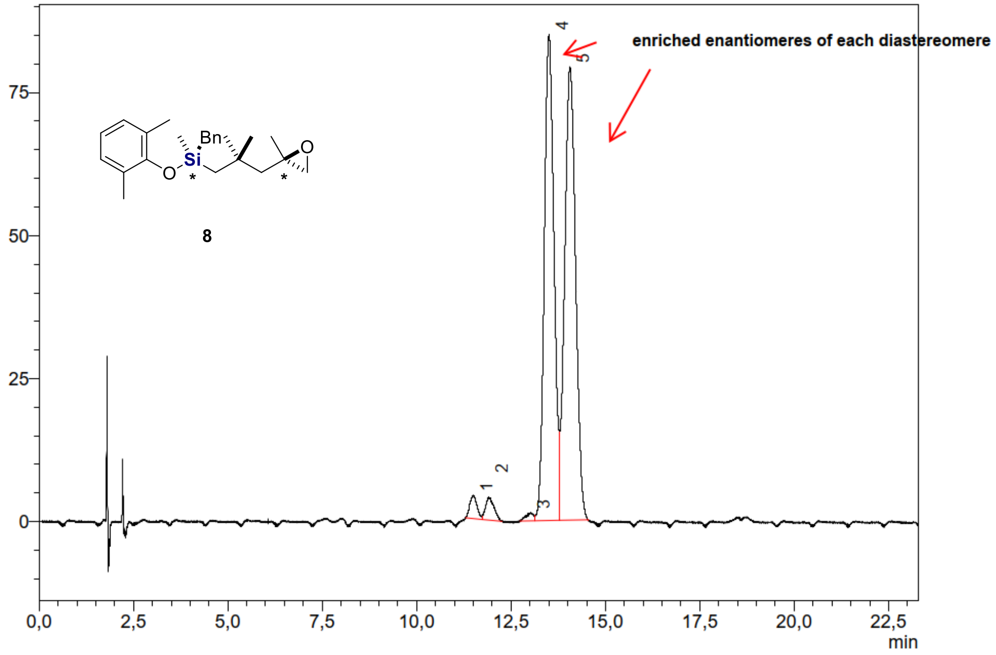

Peak #	t_R/min	% peak area
1	8.6	1.69
2	9.0	1.91
4	9.7	47.16
5	9.9	49.24
Total		100

HPLC column: OD-3, *n*-heptane, 1.0 mL/min, 298 K, 220 nm.

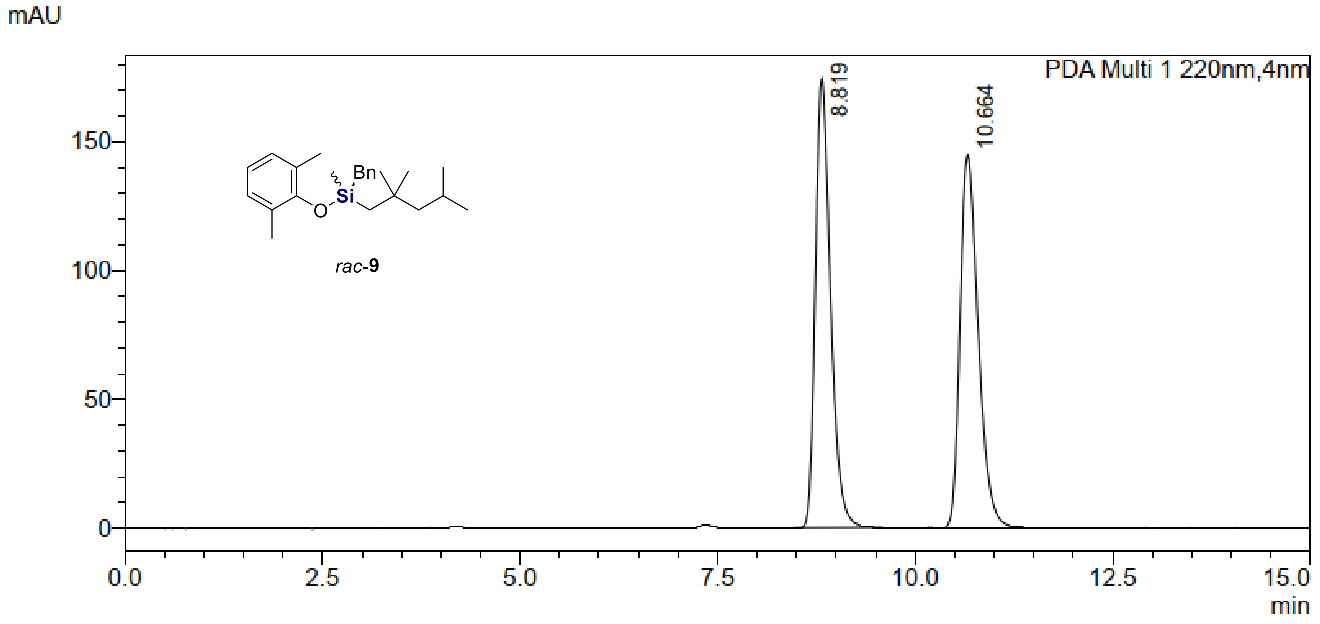

Peak #	t_R/min	% peak area
1	8.8	50.10
2	10.7	49.90
Total		100

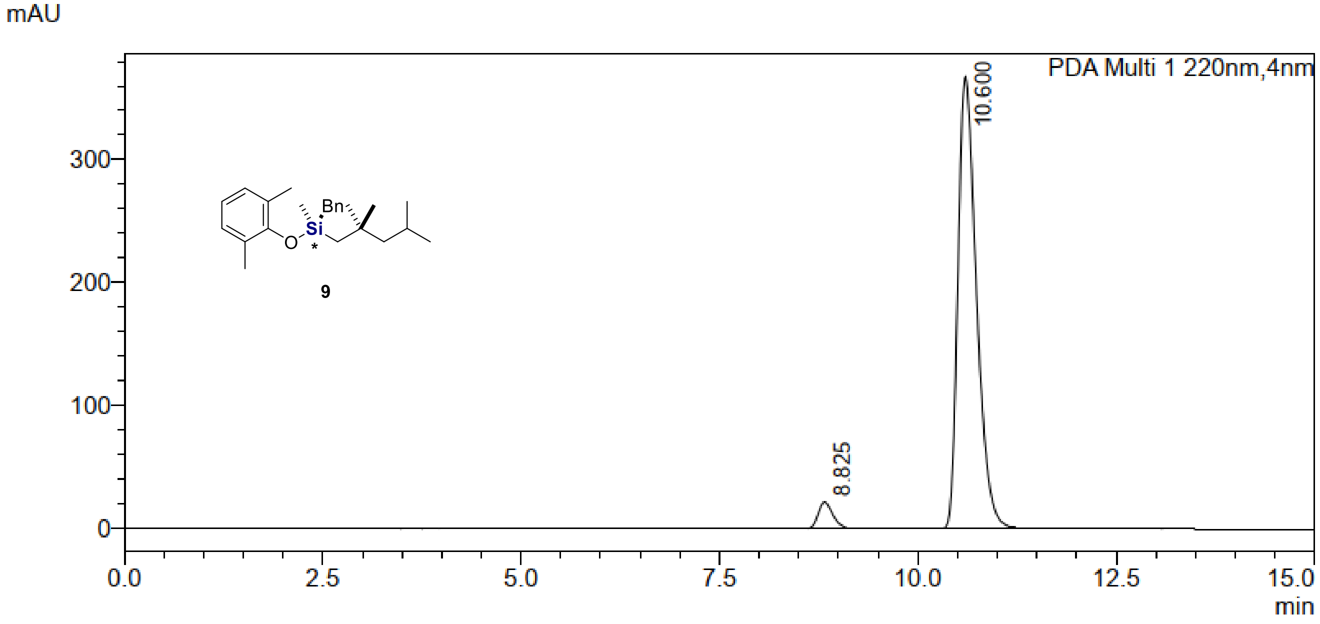

Peak #	t_R/min	% peak area
1	8.8	4.57
2	10.6	95.43
Total		100

HPLC column: OJ-3R, Acetonitrile:Water = 70:30, 1.0 mL/min, 298 K, 220 nm.

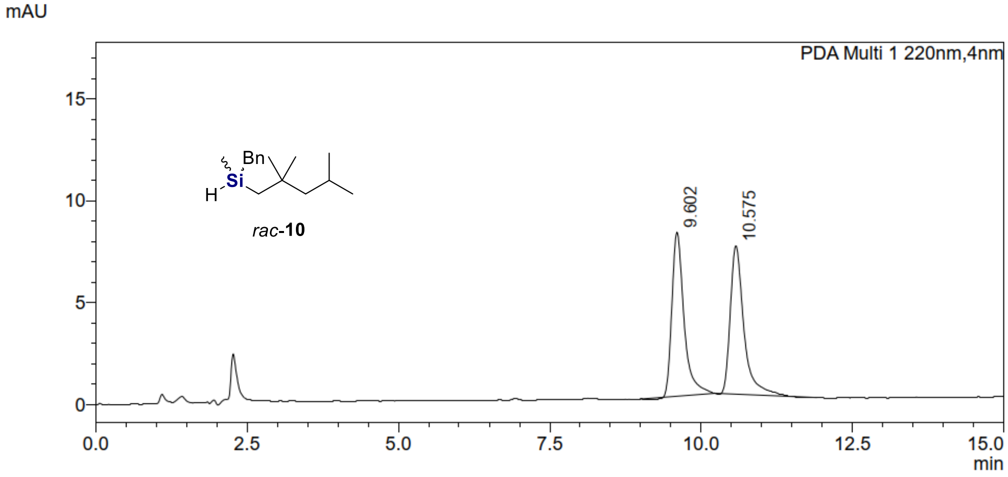

Peak #	t_R/min	% peak area
1	9.6	49.56
2	10.6	50.44
Total		100

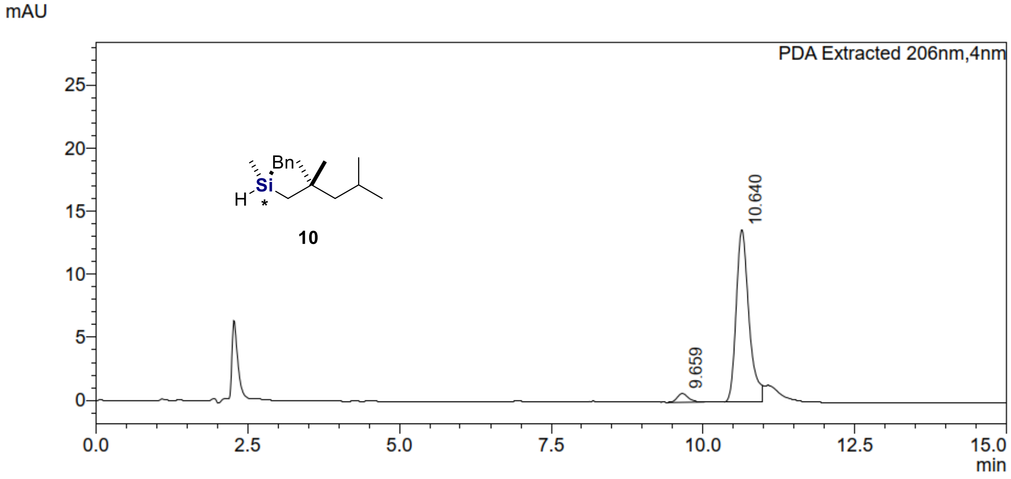

Peak #	t_R/min	% peak area
1	9.7	4.06
2	10.6	95.94
Total		100

HPLC column: OJ-3R, MeOH:Water = 85:15, 1.0 mL/min, 298 K, 220 nm.

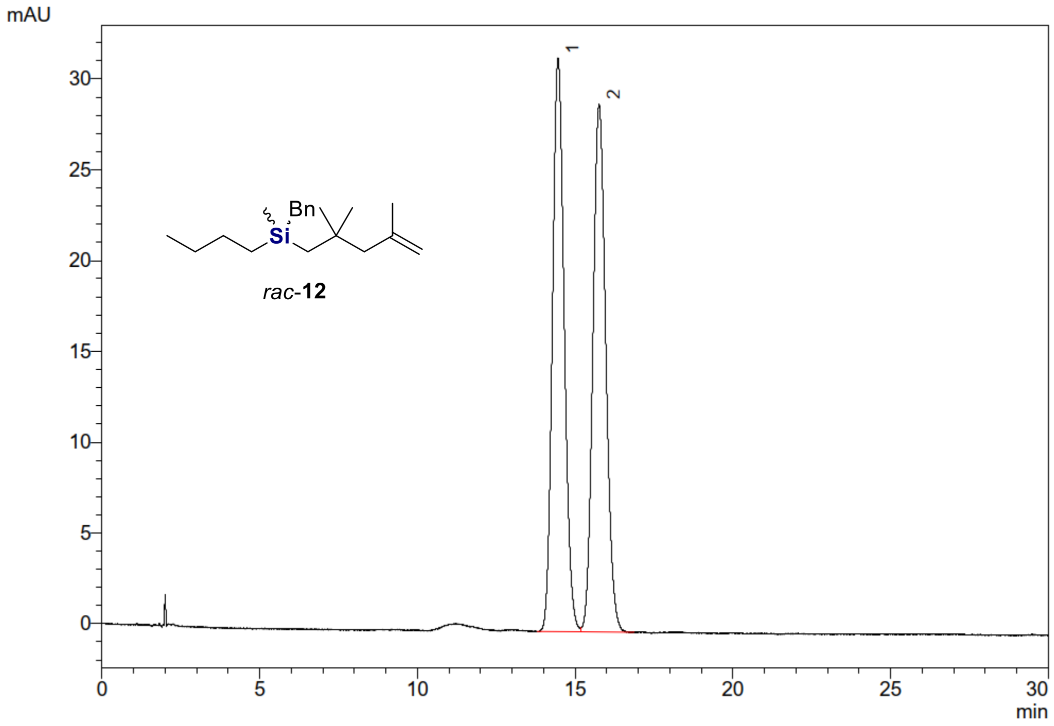

Peak #	t_R/min	% peak area
1	14.5	49.92
2	15.8	50.08
Total		100

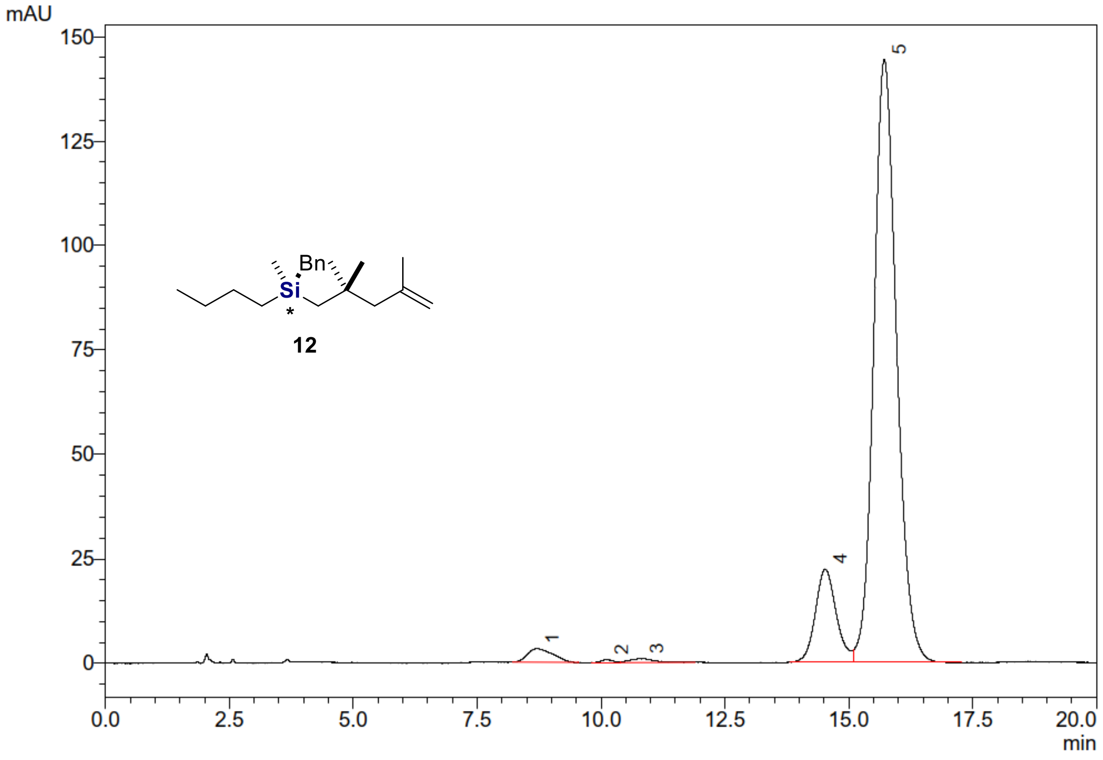

Peak #	t_R/min	% peak area
4	14.5	12.44
5	15.7	87.56
Total		100

HPLC column: OJ-3R, MeOH:Water = 85:15, 1.0 mL/min, 298 K, 220 nm.

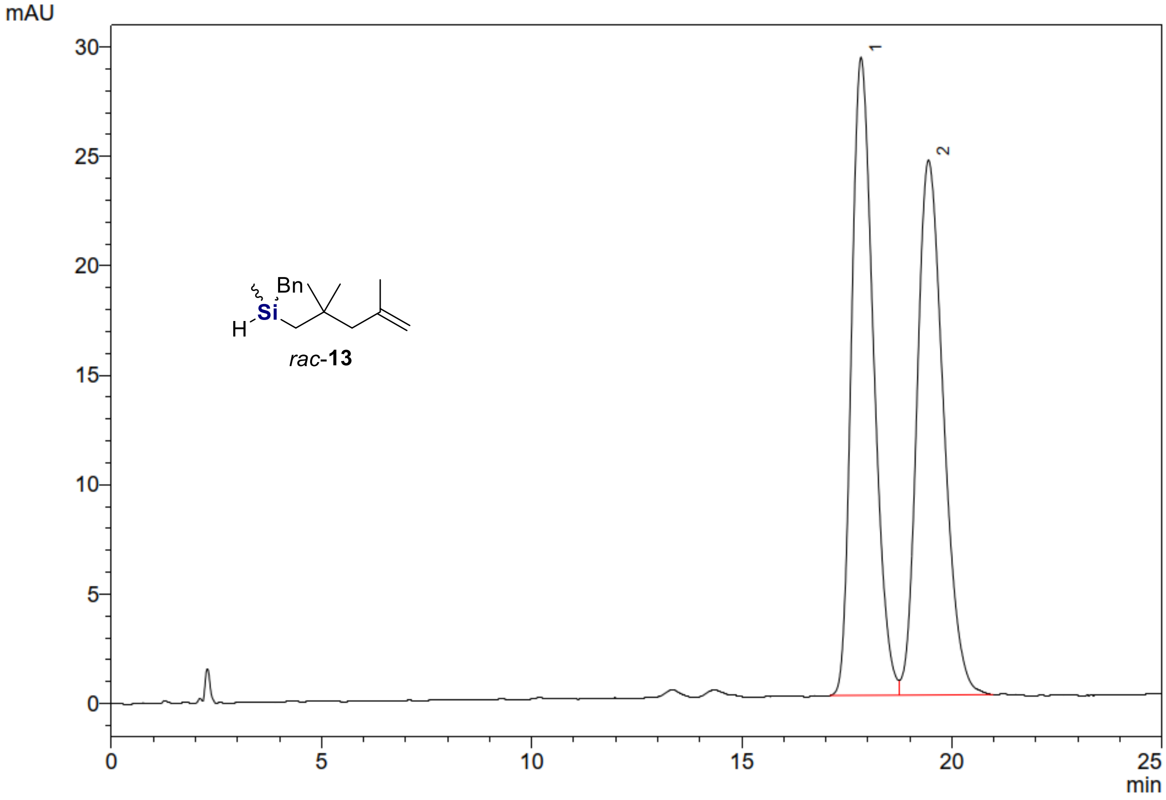

Peak #	t_R/min	% peak area
1	17.8	49.95
2	19.5	50.05
Total		100

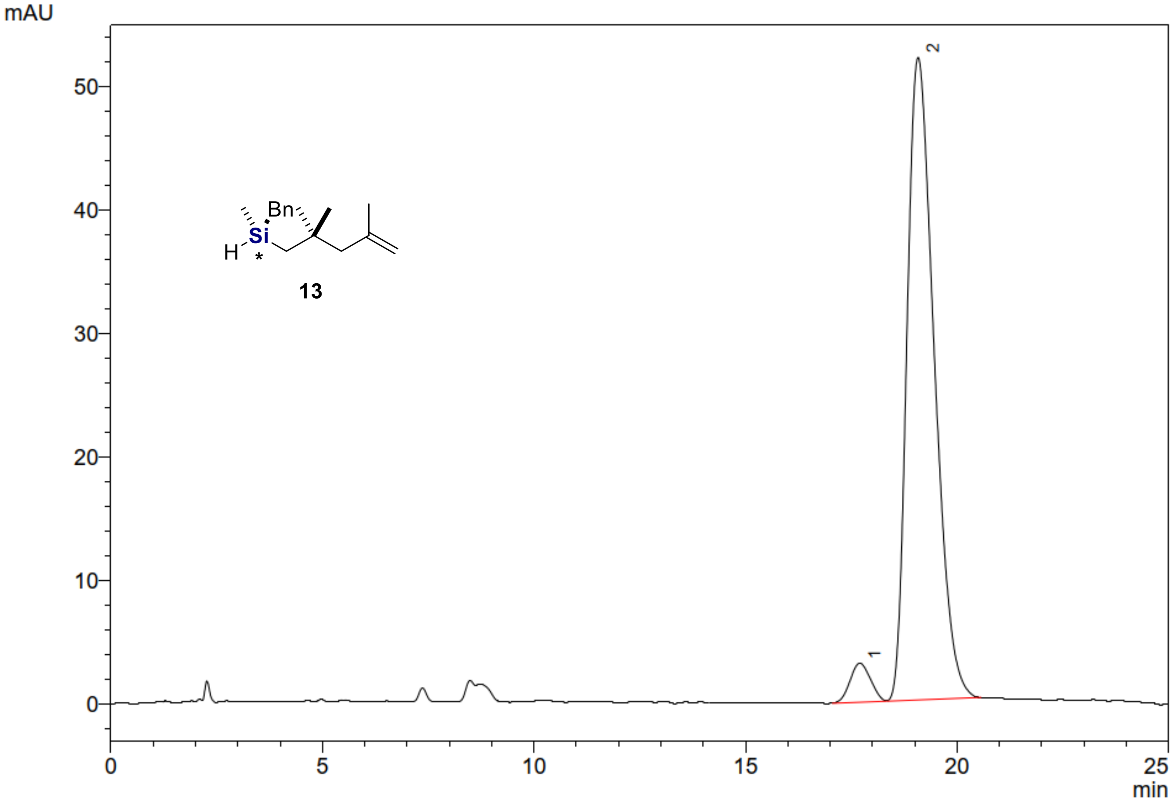

Peak #	t_R/min	% peak area
1	17.1	4.37
2	19.1	95.63
Total		100

HPLC column: OJ-3R, Acetonitrile:Water = 65:35, 1.0 mL/min, 298 K, 220 nm.

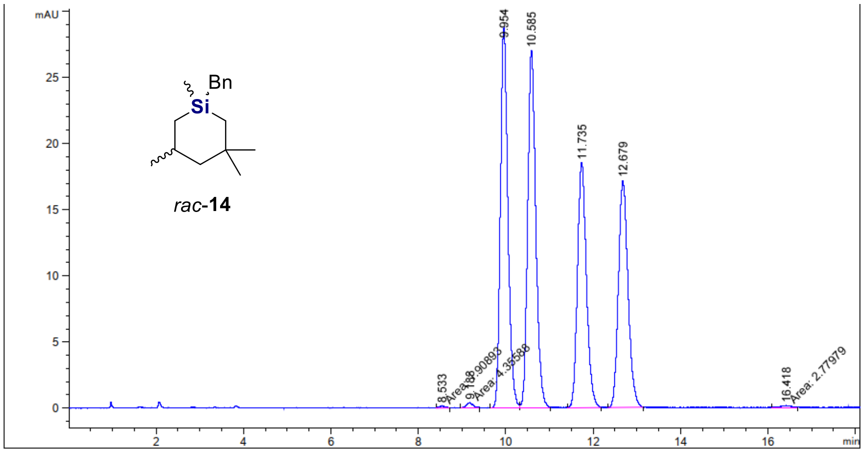

Peak #	t_R/min	% peak area
1	9.9	31.04
2	10.6	29.71
3	11.7	20.36
4	12.7	18.89
Total		100

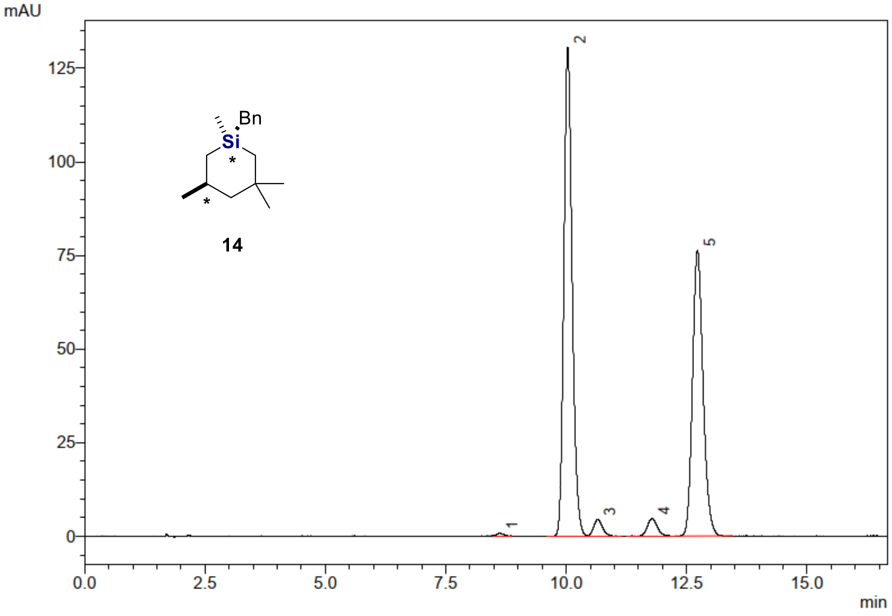

Peak #	t_R/min	% peak area
2	9.9	54.83
3	10.6	2.01
4	11.7	2.35
5	12.7	40.81
Total		100

HPLC column: IE-3, *n*-Heptane:*i*-PrOH = 99:1, 1.0 mL/min, 298 K, 220 nm.

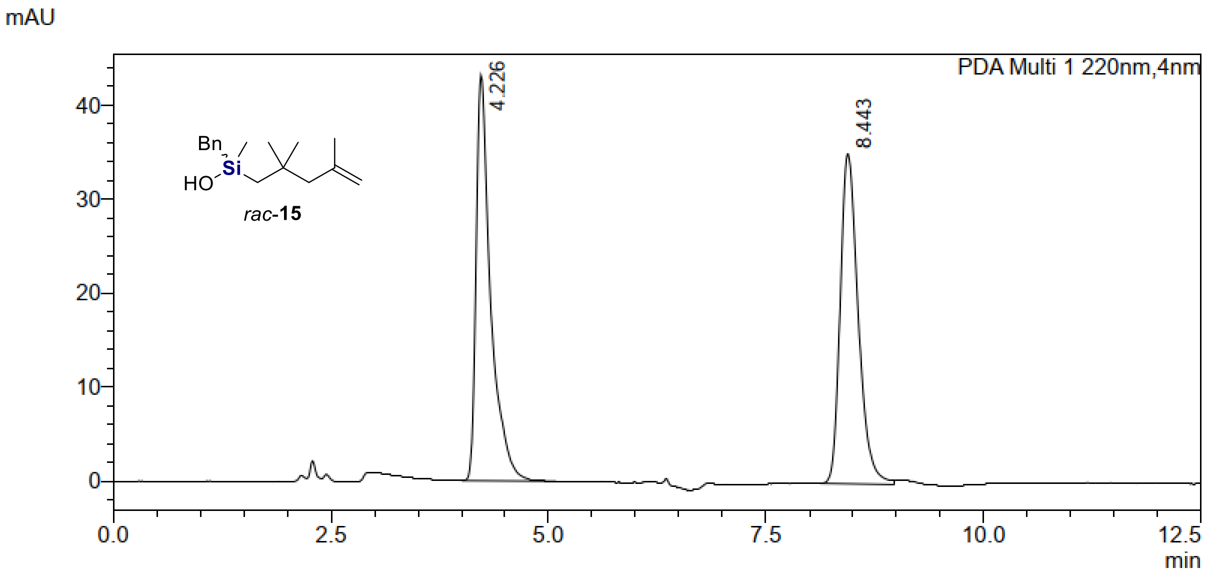

Peak #	t_R/min	% peak area
1	4.2	49.80
2	8.4	50.20
Total		100

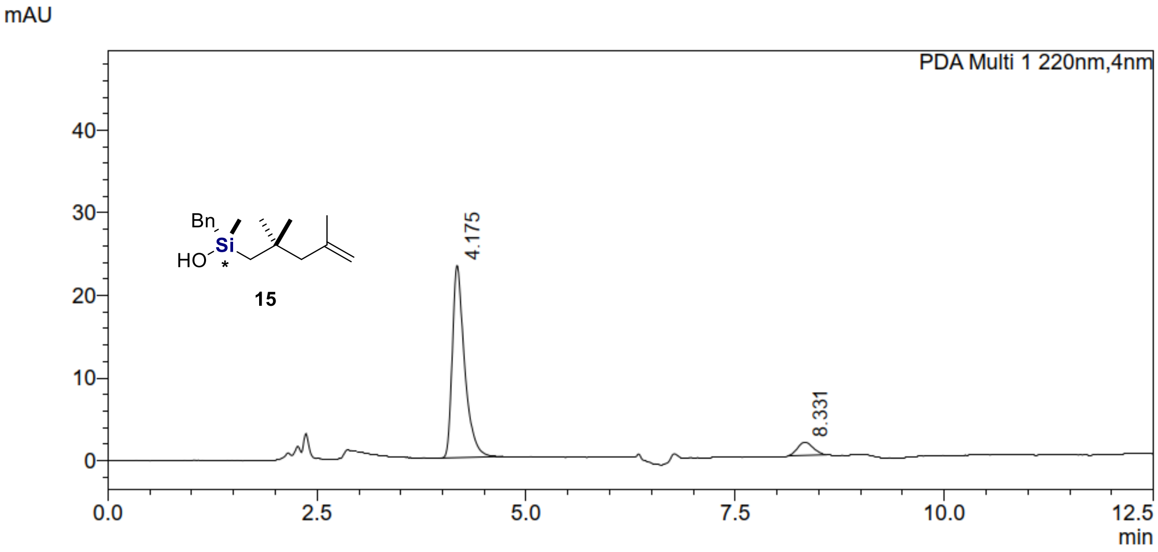

Peak #	t_R/min	% peak area
1	4.2	91.90
2	8.3	8.10
Total		100

HPLC column: OJ-3R, Acetonitrile:H_2O = 50:50, 1.0 mL/min, 298 K, 220 nm.

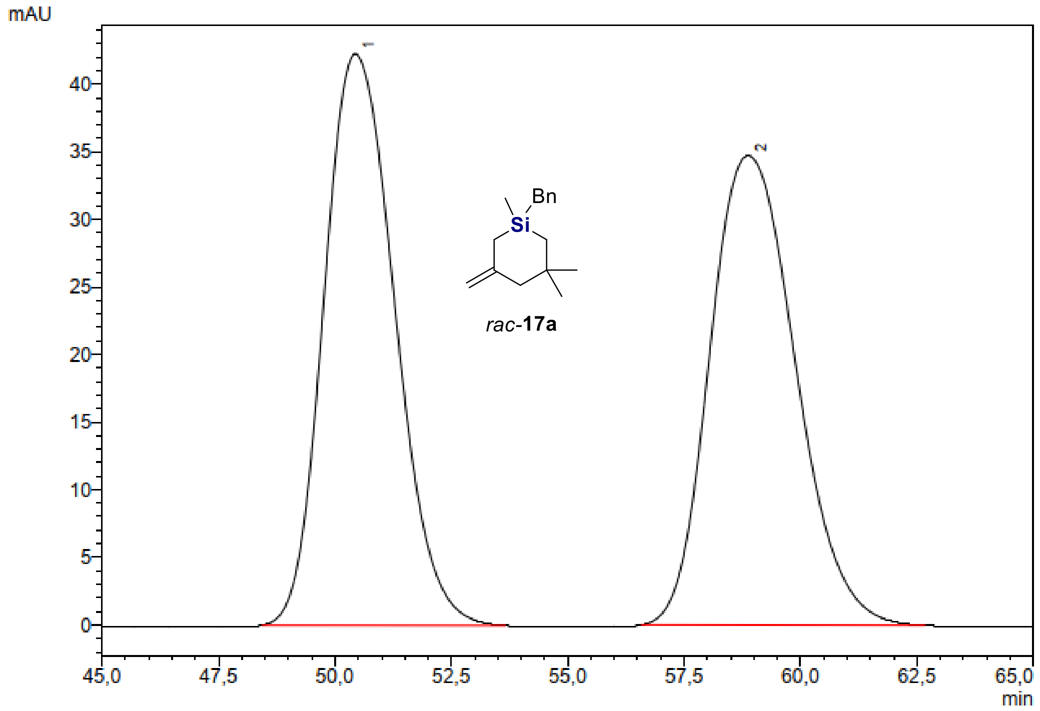

Peak #	t_R/min	% peak area
1	50.4	50.15
2	58.9	49.85
Total		100

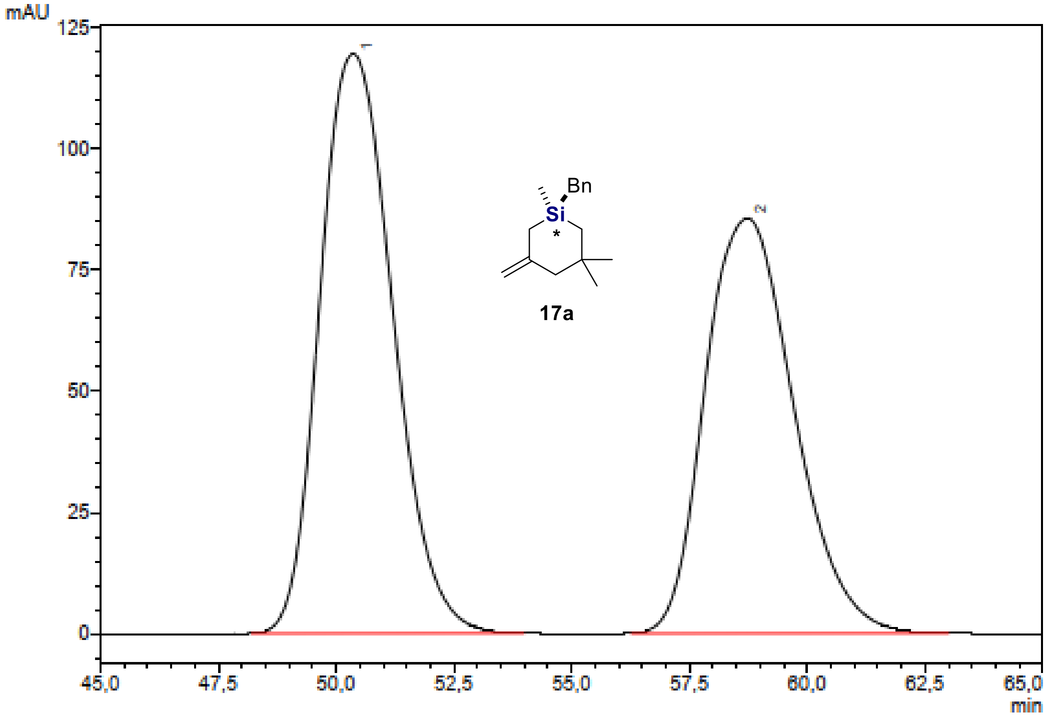

Peak #	t_R/min	% peak area
1	50.4	53.47
2	58.7	46.53
Total		100

LC traces of IDPi catalysts

Purity of the freshly prepared IDPi Catalyst **3a** is 98.18%. Method: 50 mm Zorbax SB300-C8 3.5 µm, 4.6 mm i.D. Acetonitril / 1 % TFA = 75:25, 1 mL / min, 5.7 MPa, 308 K, UV, 254 nm.

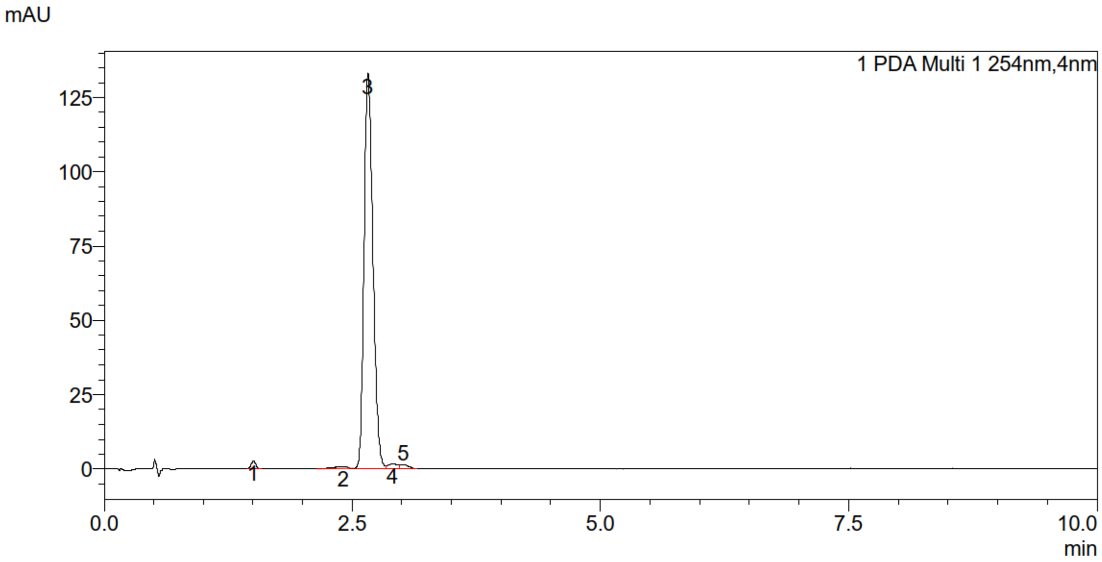

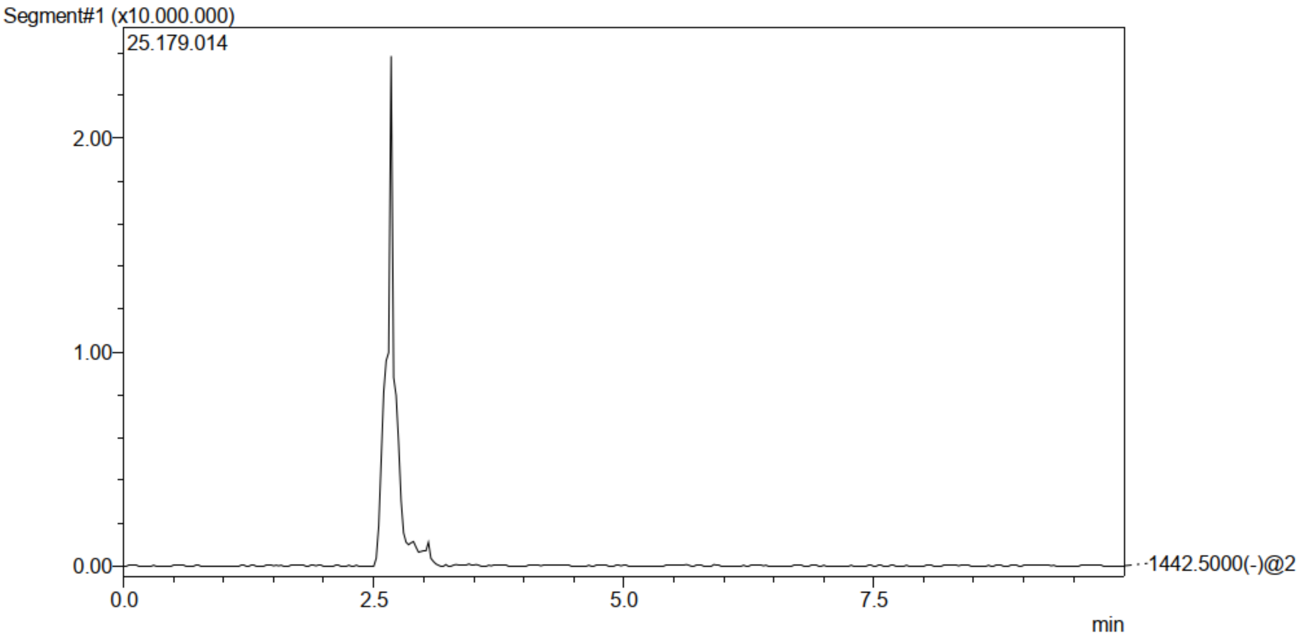

PDA Ch1 254nm

Peak#	Ret. Time	Area%	Name
1	1.50	1.08	
2	2.41	0.74	
3	2.66	96.12	Product
4	2.91	1.21	Isomer
5	3.02	0.85	Isomer
Total		100.00	

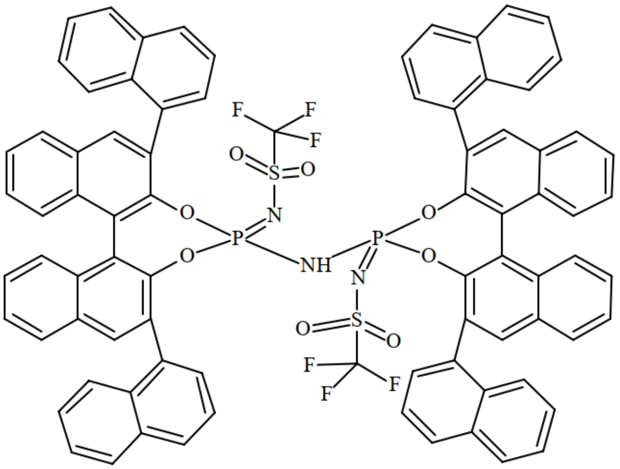

M = 1443

m/z 1442: M - H

Retention Time: 2.700 min
Base Mass: 1442
DUIS Negative

Peak 3 negativ

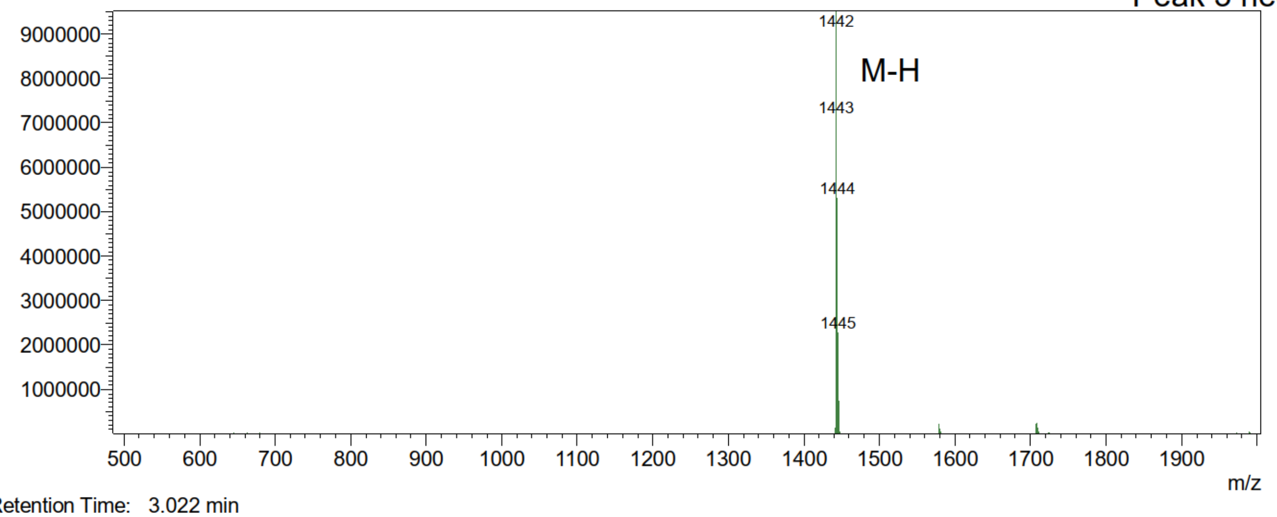

Retention Time: 3.022 min
Base Mass: 1442
DUIS Negative

Peak 4/5 negativ

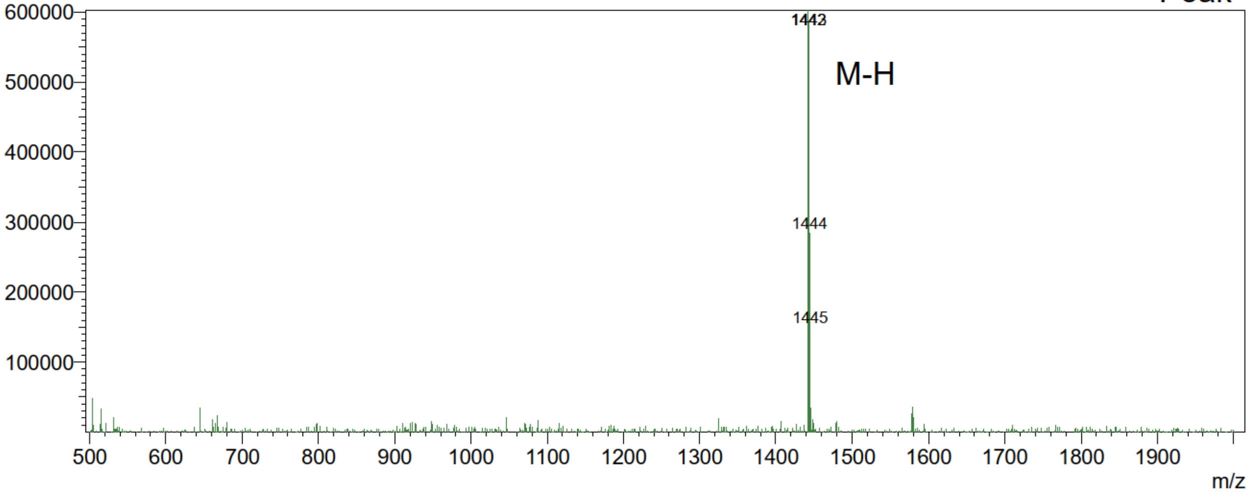

Purity of the previously prepared IDPi Catalyst **3e** is 96.03% due to slight decomposition. Method: 50 mm Zorbax 300SB-C8, 4.6 mm i.D., Acetonitril / 1 % TFA = 75:25, 1.0 mL / min, 5.9 MPa, 308 K, UV, 220 nm.

However, we couldn't exclude the influence of impurities (including $TfNH_2$) generated from decomposition on enantiocontrol, due to variations in the decomposition extent of different catalyst batches. Further mechanistic studies are currently ongoing in our lab.

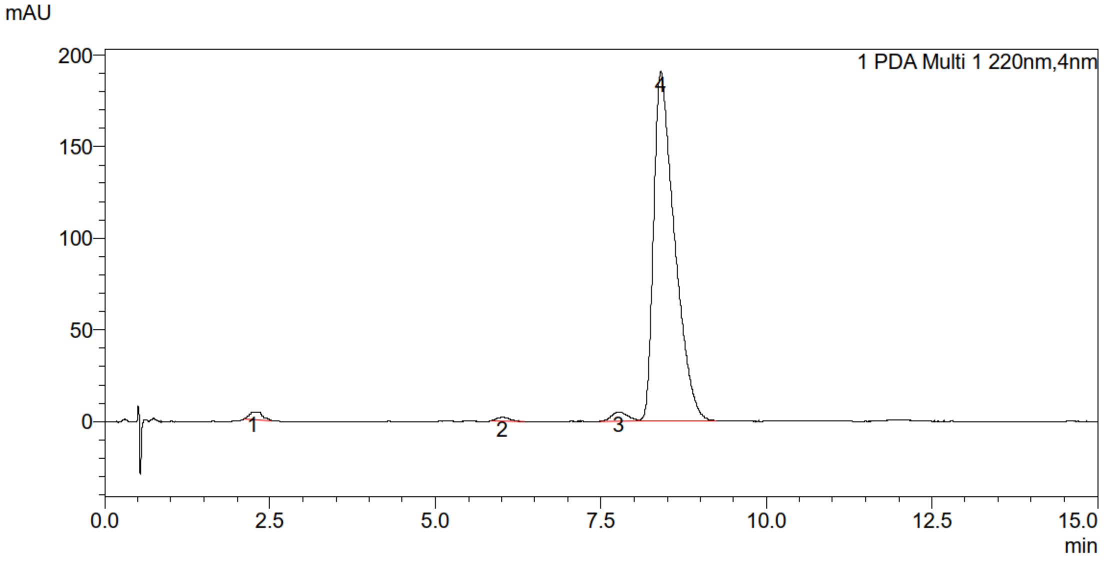

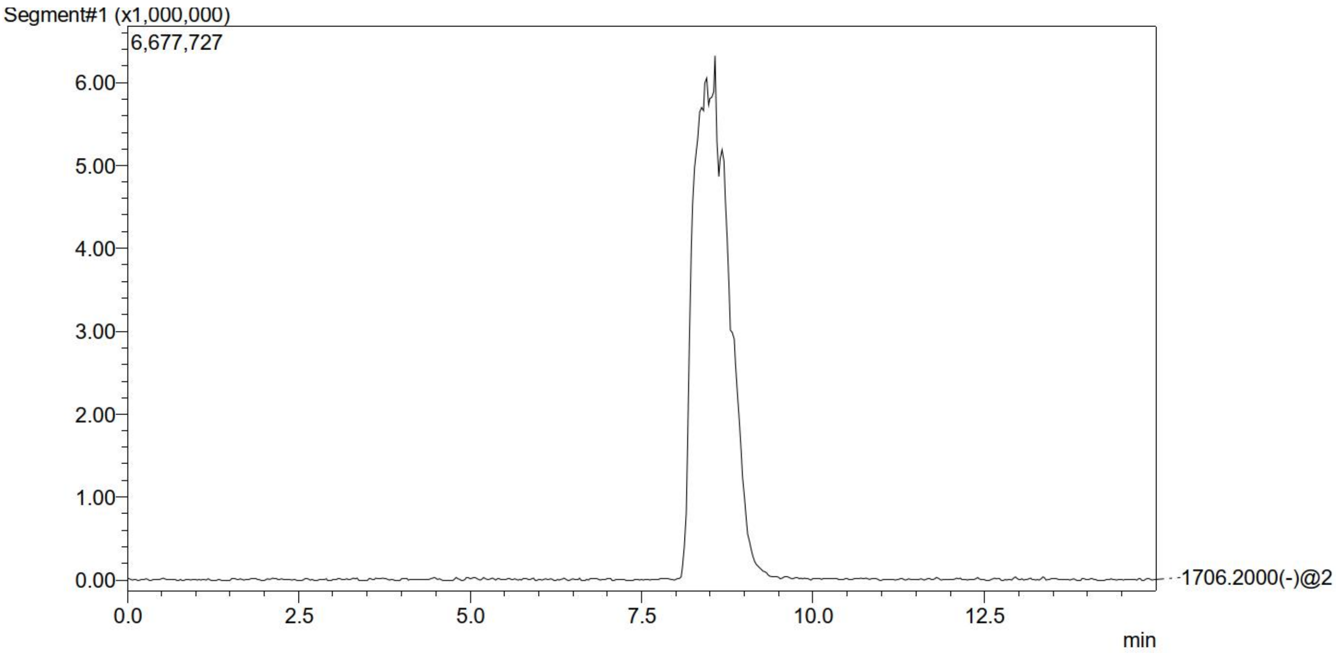

PDA Ch1 220nm

Peak#	Ret. Time	Area%	
1	2.25	1.31	
2	6.01	0.65	
3	7.77	2.01	
4	8.40	96.03	product
Total		100.00	

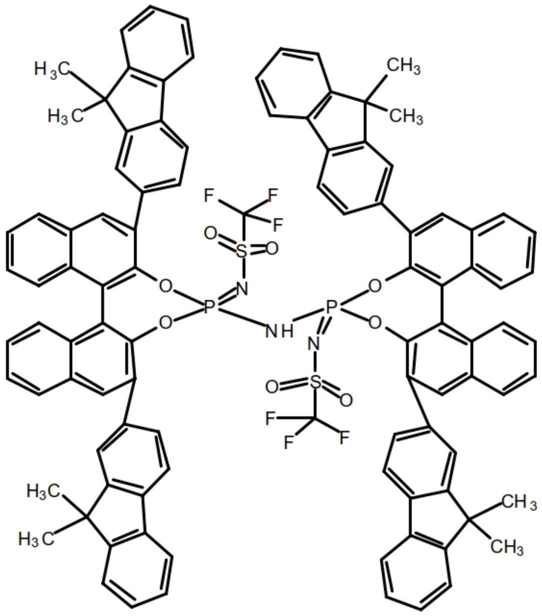

M = 1707

m/z 1706: M - H

Retention Time: 5.990 min
Base Mass: 1590
DUIS Negative

peak 2

negative

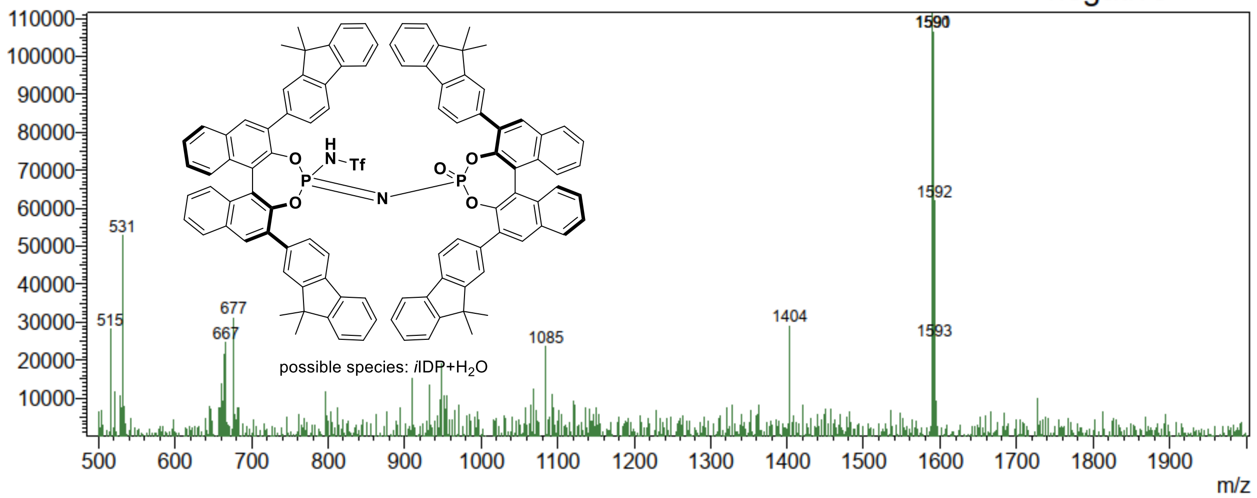

Retention Time: 7.772 min
Base Mass: 1576
DUIS Negative

peak 3

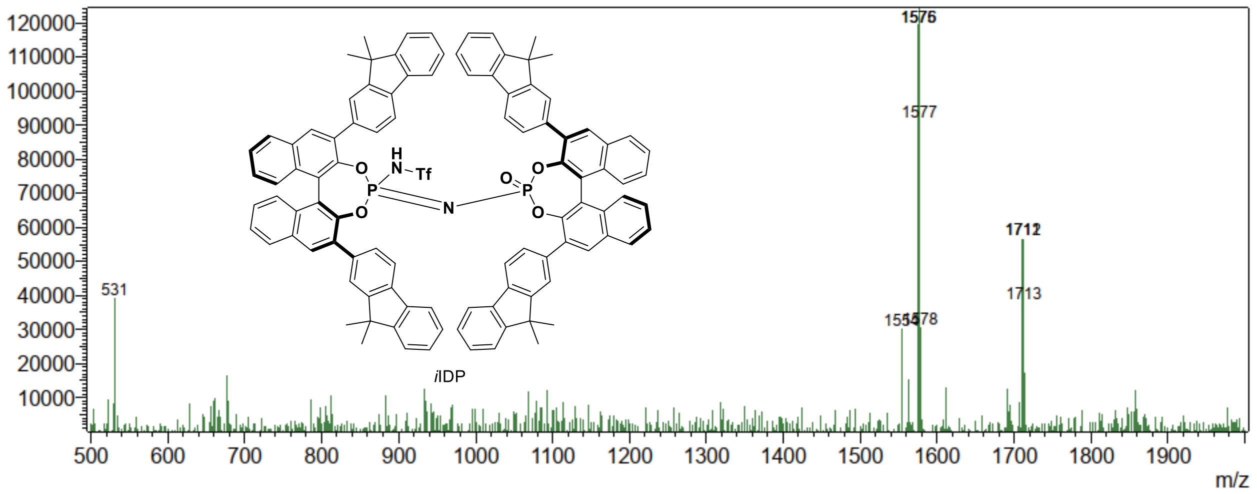

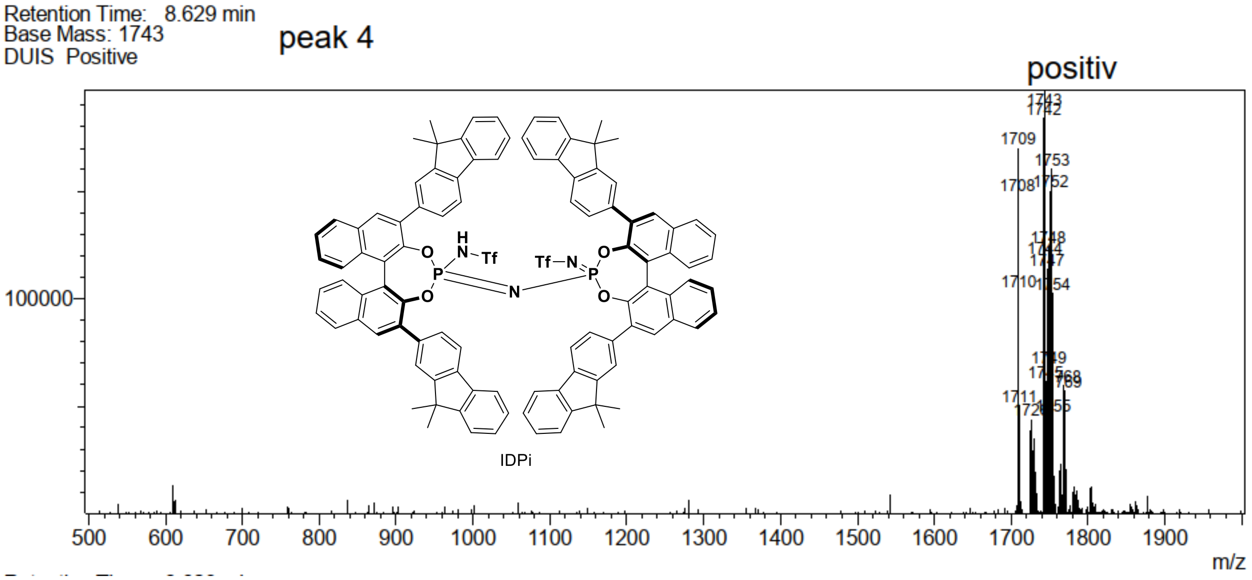
Retention Time: 8.629 min
Base Mass: 1743
DUIS Positive
peak 4
positiv
IDPi
m/z

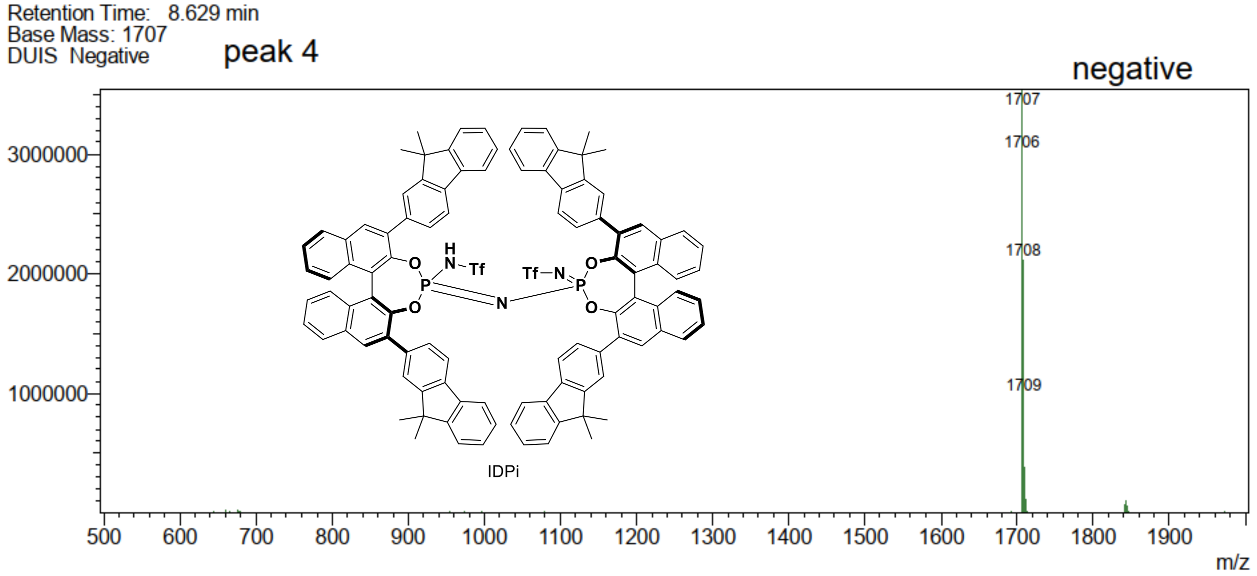
Retention Time: 8.629 min
Base Mass: 1707
DUIS Negative
peak 4
negative
IDPi
m/z

In some cases, the starting material and desired products were hard to separate from the mixture with side products, so prep LC technic was quite necessary.

For example silicon starting material **1j** was purified by Prep LC after column chromatography.

Method: 250 mm ChiralART Cellulose SB, 5 µm, 20 mm i.D.. iso-Hexane, 20 mL/min, 5.2 MPa, 298, 220 nm.

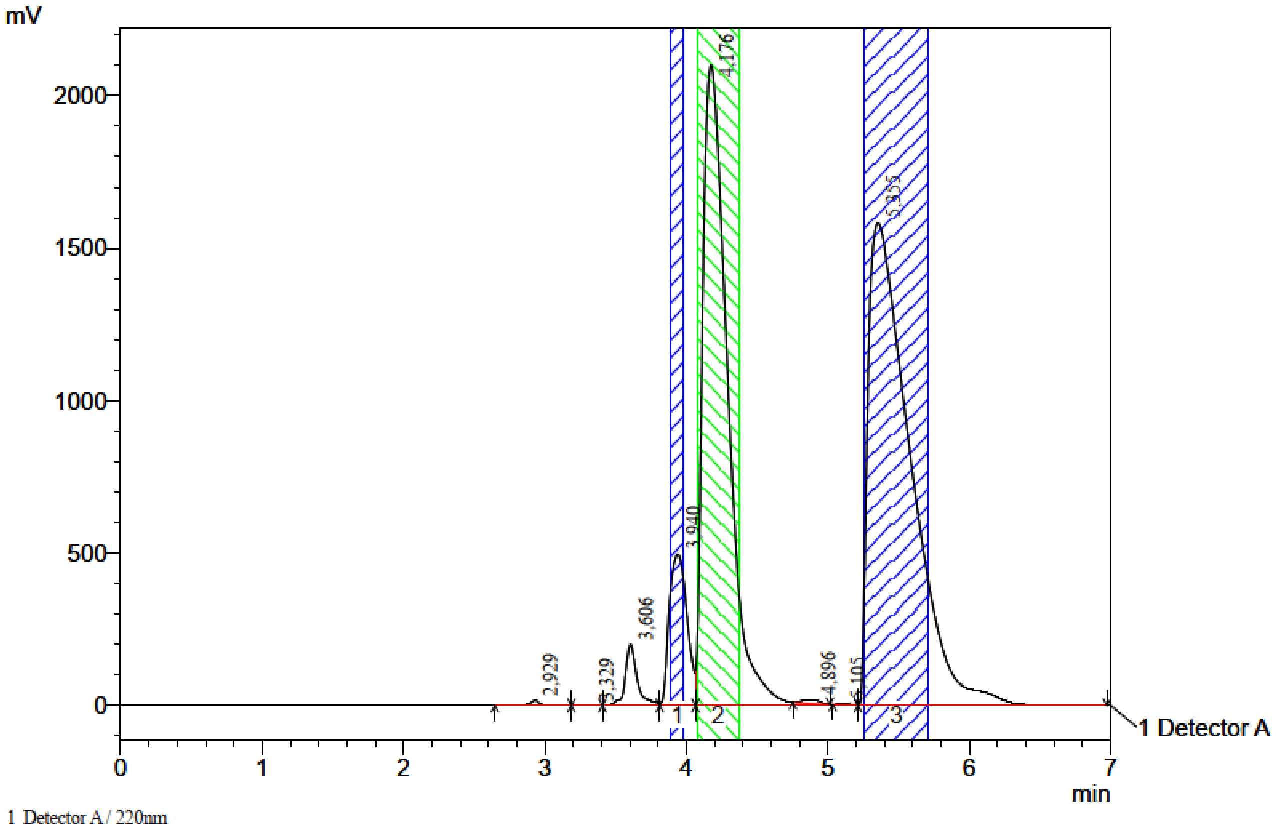

PeakTable

Detector A 220nm

Peak#	Ret. Time	Area %	k'	Name
1	2,93	0,13	0,00	
2	3,33	0,01	0,14	
3	3,61	1,82	0,23	
4	3,94	6,76	0,35	
5	4,18	40,05	0,43	
6	4,90	0,14	0,67	
7	5,10	0,02	0,74	
8	5,36	51,08	0,83	
Total		100,00		

In order to get significant amount of the minor enantiomer of compound **4b** for soaking experiments of co-crystal studies, we separated the racemic sample of compound **4b** by Prep LC.

Method: 250 mm Chiral OD-H, 5 µm, 20 mm i.D.. iso-Hexane, 12.5 mL/min, 3.6 MPa, 298, 220 nm.

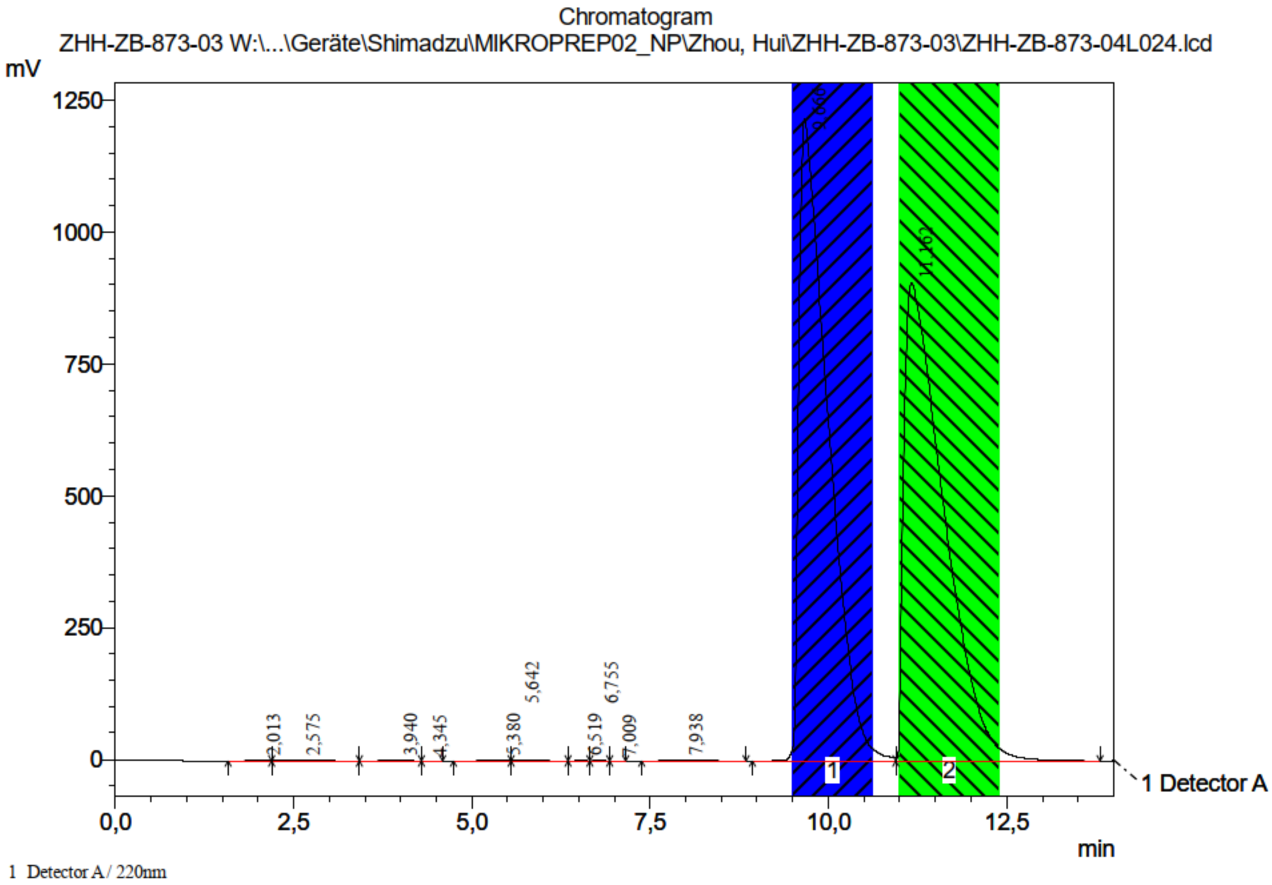

PeakTable

Detector A 220nm

Peak#	Ret. Time	Area %	k'	Name
1	2,01	0,01	0,00	
2	2,57	0,06	0,28	
3	3,94	0,02	0,96	
4	4,34	0,00	1,16	
5	5,38	0,03	1,67	
6	5,64	0,04	1,80	
7	6,52	0,01	2,24	
8	6,75	0,01	2,35	
9	7,01	0,00	2,48	
10	7,94	0,05	2,94	
11	9,67	49,67	3,80	
12	11,16	50,08	4,54	
Total		100,00		

After separation, the pure enantiomers were again confirmed by HPLC analysis, the traces are shown below:

Method: HPLC column: IB-N3, *n*-heptane, 0.5 mL/min, 298 K, 220 nm.

Racemate traces:

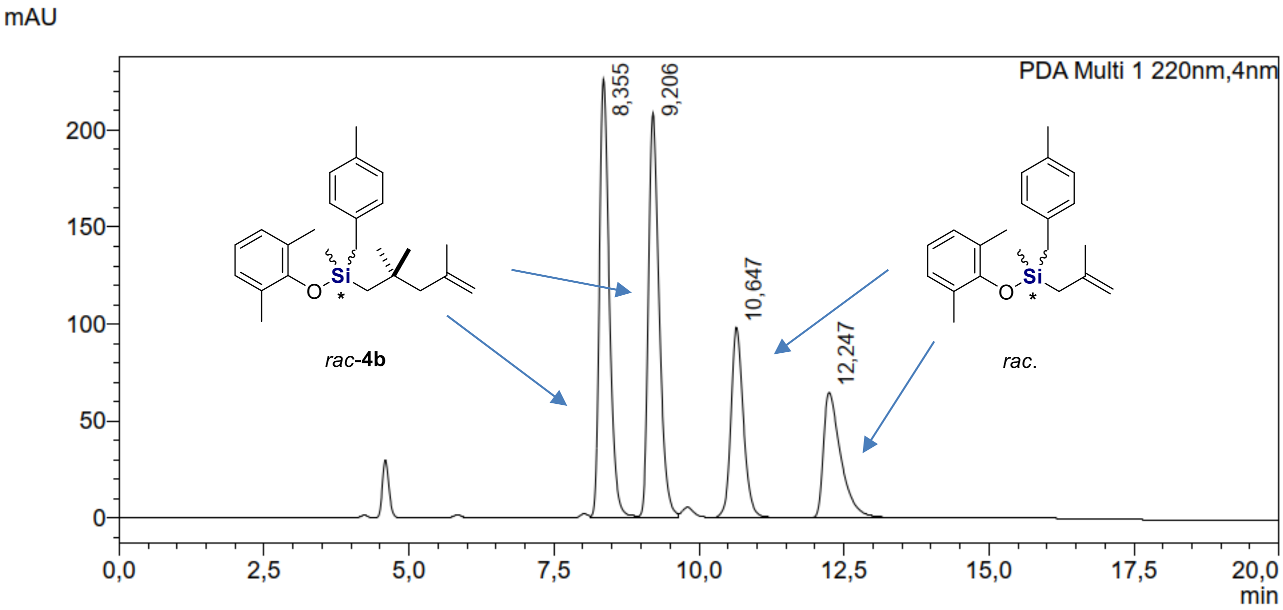

PDA Ch1 220nm

Peak#	Ret. Time	Area%
1	8,355	32,722
2	9,206	32,975
3	10,647	17,901
4	12,247	16,402
Total		100,000

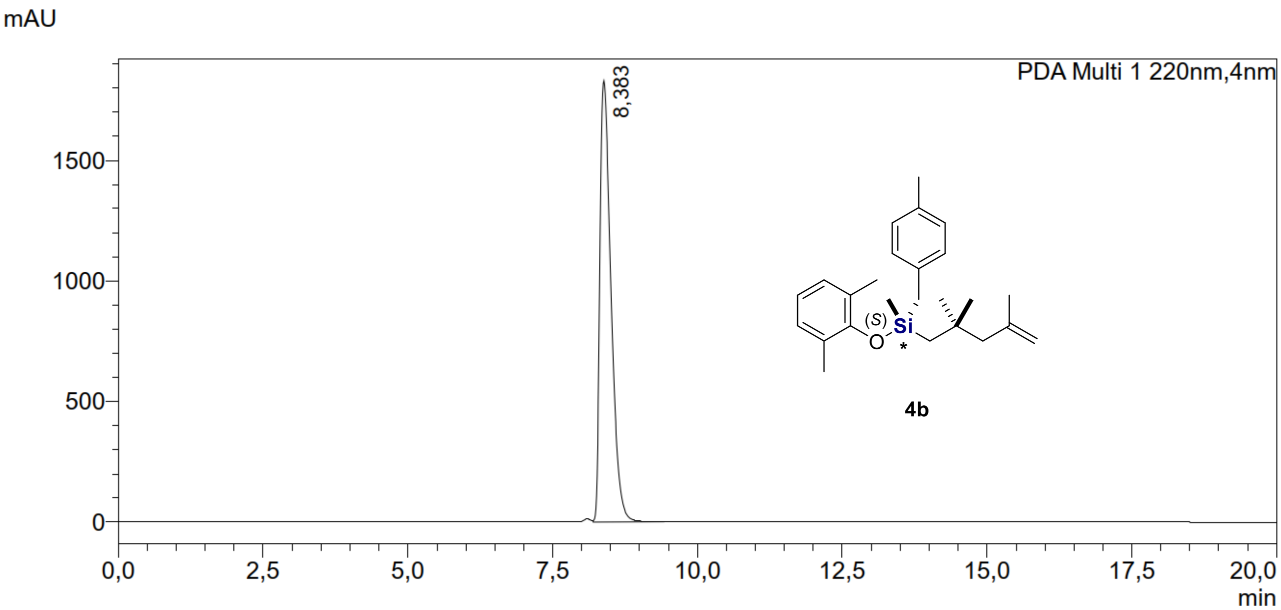

PDA Ch1 220nm

Peak#	Ret. Time	Area%
1	8,383	100,000
Total		100,000

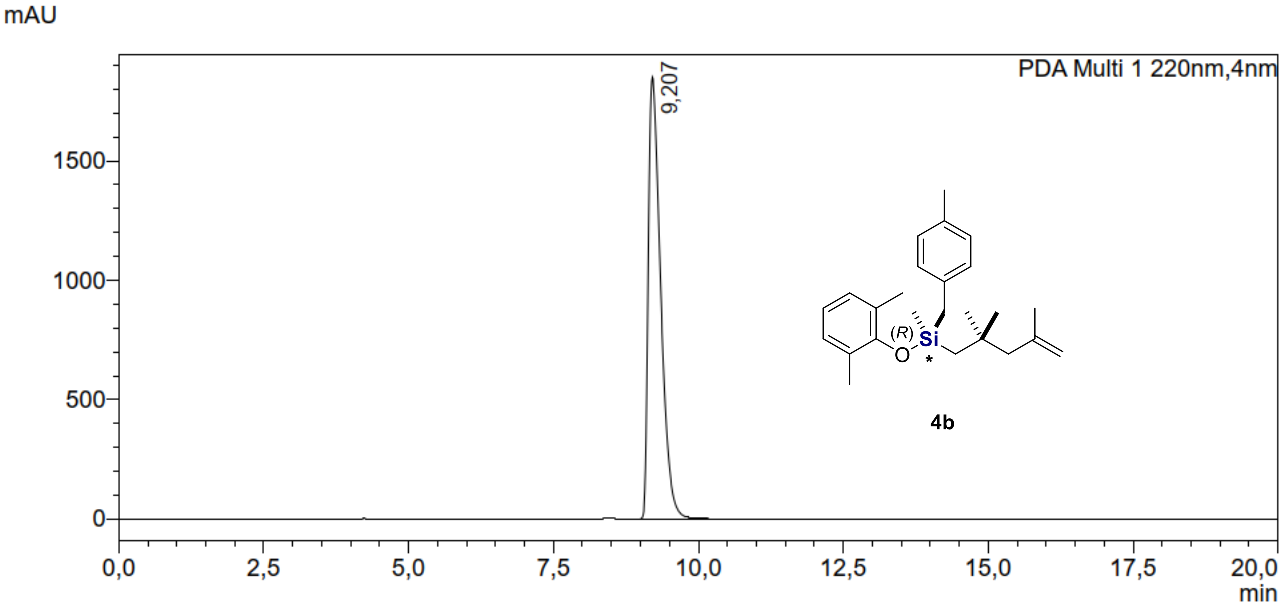

PDA Ch1 220nm

Peak#	Ret. Time	Area%
1	9,207	100,000
Total		100,000
